# Can Gene Expression Analysis in Zero-Time Biopsies Predict Kidney Transplant Rejection?

**DOI:** 10.3389/fmed.2022.793744

**Published:** 2022-03-30

**Authors:** Eva Vonbrunn, Miriam Angeloni, Maike Büttner-Herold, Janina Müller-Deile, Katharina Heller, Erik Bleich, Stefan Söllner, Kerstin Amann, Fulvia Ferrazzi, Christoph Daniel

**Affiliations:** ^1^Department of Nephropathology, Institute of Pathology, Friedrich-Alexander-University Erlangen-Nuremberg and University Hospital, Erlangen, Germany; ^2^Institute of Pathology, Friedrich-Alexander-University Erlangen-Nuremberg and University Hospital, Erlangen, Germany; ^3^Department of Nephrology and Hypertension, Friedrich-Alexander-University Erlangen-Nuremberg and University Hospital, Erlangen, Germany

**Keywords:** kidney transplantation, zero-time biopsy, mRNA expression, inflammation, outcome

## Abstract

Zero-time biopsies are taken to determine the quality of the donor organ at the time of transplantation. Histological analyses alone have so far not been able to identify parameters that allow the prediction of subsequent rejection episodes or graft survival. This study investigated whether gene expression analyses of zero-time biopsies might support this prediction. Using a well-characterized cohort of 26 zero-time biopsies from renal transplant patients that include 4 living donor (LD) and 22 deceased donor (DD) biopsies that later developed no rejection (Ctrl, *n* = 7), delayed graft function (DGF, *n* = 4), cellular (T-cell mediated rejection; TCMR, *n* = 8), or antibody-mediated rejection (ABMR, *n* = 7), we analyzed gene expression profiles for different types of subsequent renal transplant complication. To this end, RNA was isolated from formalin-fixed, paraffin-embedded (FFPE) sections and gene expression profiles were quantified. Results were correlated with transplant data and B-cell, and plasma cell infiltration was assessed by immunofluorescence microscopy. Both principal component analysis and clustering analysis of gene expression data revealed marked separation between LDs and DDs. Differential expression analysis identified 185 significant differentially expressed genes (adjusted *p* < 0.05). The expression of 68% of these genes significantly correlated with cold ischemia time (CIT). Furthermore, immunoglobulins were differentially expressed in zero-time biopsies from transplants later developing rejection (TCMR + ABMR) compared to non-rejected (Ctrl + DGF) transplants. In addition, immunoglobulin expression did not correlate with CIT but was increased in transplants with previous acute renal failure (ARF). In conclusion, gene expression profiles in zero-time biopsies derived from LDs are markedly different from those of DDs. Pre-transplant ARF increased immunoglobulin expression, which might be involved in triggering later rejection events. However, these findings must be confirmed in larger cohorts and the role of early immunoglobulin upregulation in zero-biopsies needs further clarification.

## Introduction

At the time point of kidney transplantation, many transplant centers routinely collect zero-time biopsies for determining graft quality, as a reference for later biopsies, and for gathering information with the potential to predict the later outcome after transplantation. However, the benefits of this practice are still under debate. Rathore et al. described that zero-time biopsies provide information regarding the general condition of the kidneys of the donors and that interstitial fibrosis and acute tubular injury in living donors (LDs) were significantly associated with allograft dysfunction (1). However, another study revealed that a mild degree of subclinical pathologic findings did not affect graft function after LD kidney transplantation (2). There are also contradictory results and discrepant conclusions in studies that investigated deceased donor (DD) kidneys. While Tavakkoli et al. showed no relationship between histological findings and graft survival in DDs (3), others reported that histological findings predict early graft function (4). Even the use of various standard immunohistological examinations in zero-time biopsies did not reveal useful markers that could indicate graft outcome. In a previous study involving a cohort of living and DDs, we showed that glomerular immune reactivity is a frequent finding in zero-time biopsies. Yet it does not have an impact on graft function not on survival (5). Accordingly, similar studies could not identify immunohistochemical parameters that are predictive of rejection or graft outcome (6).

Given that histological and immunohistochemical analyses have not yet identified parameters that definitely predict subsequent rejection episodes or graft survival, other analytic methods should be considered. Some gene expression analyses have shown that differences in the transcriptome of kidneys of the donors reflect graft function (7). Furthermore, a better understanding of the molecular mechanisms influencing graft outcome might be discovered by analyzing differential gene expression patterns in zero-time biopsies (8).

Here, we tested the hypothesis that expression profiles of transplant-related genes in kidneys of the donors can predict subsequent graft outcomes. To this aim, we examined the gene expression profiles of 26 zero-time biopsies from renal transplant patients who developed no rejection or dysfunction (Ctrl, *n* = 7), delayed graft function (DGF, *n* = 4), T-cell mediated rejection (TCMR, *n* = 8), or antibody-mediated rejection (ABMR, *n* = 7). For expression analysis, RNA was isolated from formalin-fixed, paraffin-embedded (FFPE) sections and quantified with the NanoString Human Organ Transplant panel measuring the expression of nearly 800 genes. Genes with remarkable expression profiles regarding the different outcome groups were further analyzed.

## Materials and Methods

### Renal Tissue Specimens

In this study, zero-time biopsies collected from renal grafts before transplantation between 2015 and 2019 from the Department of Nephrology at the FAU Erlangen-Nuremberg, Germany were included. To identify differences in gene expression in time-zero biopsies, 26 carefully selected FFPE specimens of archived donor kidney biopsies (from the Department of Nephropathology, University Hospital, Erlangen, Germany) were used to evaluate characteristic mRNA expression profiles in zero-time biopsies from patients who later developed DGF (*n* = 4), ABMR (*n* = 7), and TCMR (*n* = 8; 1 LD, 7 DDs) within the first 2.5 years. Zero-time biopsies were taken as a protocol biopsy immediately before implantation of the graft into the recipient. Biopsies from patients with borderline changes or other co-morbidities, such as viral infection, immunoglobulin A (IgA)-nephropathy, or other immune-complex glomerulonephritis, were excluded. Neither patients from the control group nor from the DGF group developed a rejection or a borderline reaction at later stages. Zero-time biopsies from kidneys without signs of renal dysfunction or rejection after 1 year from transplantation served as controls (*n* = 7; 3 LDs, 4 DDs). DGF was defined as impaired renal function necessitating dialysis within the first 10 days post-transplantation and lack of rejection. For the ABMR group, we included zero-time biopsies that later developed ABMR (active type II) with evidence of donor-specific antibodies (DSAs). Zero-time biopsies for the TCMR group were included if cases were developed later on after transplantation acute type IA, IB, or IIA TCMR without signs of ABMR. The study groups, characteristics of patients, and Banff classification are described for donors (Table 1 and Supplementary Data 1) and for recipients (Supplementary Datas 2, 3) (9). The Ethics Committee of the Friedrich-Alexander-University approved the use of archival material, waiving the need for retrospective consent for the use of archived rest material (Re.-No. 4415).

**TABLE 1:** Kidney donor characteristics.

	Ctrl	DGF	TCMR	ABMR	Total
Donor *n* = 26	7	4	8	7	26
Men (%)	2 (29%)	1 (25%)	4 (50%)	3 (43%)	10 (38%)
Age at transplant (years)	49 ± 18	49 ± 9	54 ± 7	52 ± 9	51 ± 12
Body mass index (kg/m^2^)	27 ± 3	27 ± 2	29 ± 8	29 ± 3	28 ± 5
Serum creatinine (mg/dl)	1.19 ± 0.96	3.02 ± 2.13	1.36 ± 0.53	2.54 ± 2.13	1.89 ± 1.67
Smoker	0	1	2	1	4
Diabetes	0	1	0	0	1
Hypertension	1	2	3	3	9
CAD	1	1	2	0	4
Sepsis	0	1	3	1	5
ARF	1	4	5	5	15
Deceased (%)	4 (57%)	4 (100%)	7 (88%)	7 (100%)	22 (85%)
Reanimation	2	4	5	3	14
CIT (min)	313 ± 289	795 ± 146	680 ± 280	813 ± 215	635 ± 322

*Ctrl, control; DGF, delayed graft function; TCMR, T-cell mediated rejection; ABMR, antibody-mediated rejection; CAD, coronary artery disease; ARF, acute kidney injury; CIT, cold ischemia time. Ranges are stated as mean ± SD.*

### Multiplex mRNA Expression Analysis by NanoString

For expression analysis, RNA was isolated from 15 μm sections using the RNeasy FFPE Kit (Qiagen, Venlo, Netherlands). RNA concentration and purity were measured with a NanoDrop Spectrophotometer (Thermo Fisher Scientific, Waltham, MA, United States), and isolates with a 260/280 nm absorbance ratio below 1.4 were excluded. All samples had a volume of 25 μl H_2_O containing 111–393 ng mRNA and were concentrated using a Savant SPD111 SpeedVac (Thermo Fisher Scientific) at 35°C for 24 min to a volume of 2–3 μl. According to the recommendations of manufacturer, after a hybridization and preparation step, gene expression was analyzed with the NanoString nCounter FLEX Analysis System (NanoString Technologies, Seattle, WA, United States) using the nCounter Banff Human Organ Transplant (B-HOT) panel, containing 760 genes and 10 internal reference genes (10).

### Analysis of NanoString Gene Expression Data and Statistics

Analysis of NanoString gene expression raw data (Supplementary Data 4) was performed relying on the DESeq2 package v. 1.34.0 (11) within R. v. 4.1.2/Bioconductor v. 3.14 environment (12, 13). Positive/negative controls were excluded from the analysis, and the estimation of size factors was based on housekeeping genes. Differential expression analysis was performed that includes as factors donor type (living/deceased) and later rejection (yes/no) of the samples. A gene was considered differentially expressed if its (Benjamini–Hochberg) adjusted *p* was lower than 0.05. Principal component analysis was based on the variance-stabilized transformed counts of the top 100 variable genes. The expression heatmap was generated using standardized variance-stabilized transformed counts relying on the Complex Heatmap package v. 2.10.0. In order to perform functional enrichment analysis, first gene symbols associated with the NanoString nCounter^®^ Human Organ Transplant Panel were mapped to Entrez Gene Ids relying on the biomaRt package v. 2.44.4 (14, 15). Functional enrichment analysis of differentially expressed genes was then performed relying on the enrichGO function of the clusterProfiler package v. 3.16.1 (16) within the R environment v.4.0.3, using Entrez Gene Ids as identifiers. As background for the analysis, the set of uniquely mapped Entrez Gene Ids was employed. Pathways were considered significantly enriched if their associated adjusted value of *p* was <0.05. Dot plots of the expression of single genes were generated using normalized count data relying on ggplot2 v.3.3.5.

To test for correlation between expression and CIT, Spearman’s rank correlation analysis was performed. Wilcoxon rank-sum test was used to assess differences in expression of selected genes between ARF and no ARF, an abundance of CD20- and CD138-positive cells between rejection and no rejection group, and to test for differences in age of the donor. A *p***<** 0.05 was considered statistically significant (*p**<** 0.05; ^**^p**<** 0.01; ^***^p**<** 0.001; ^****^p**<** 0.0001).

### Immunofluorescence Double Staining

For immunofluorescence microscopy, 2 μm sections of FFPE kidney biopsies were utilized for the staining procedure. After antigen retrieval in target retrieval solution pH 6 (DAKO Deutschland, Hamburg, Germany) for 2.5 min in a pressure cooker, sections were blocked in 1% bovine serum albumin diluted 1:50 in 50 mM Tris pH 7.4. Sections were then incubated overnight at room temperature with a mouse monoclonal antibody (IgG2a) against human CD20cy (clone L26; M0755 Dako Deutschland) together with a mouse monoclonal antibody (IgG1) against human CD138 (clone B-A38, MSK063, Zytomed Systems GmbH, Berlin, Germany), both diluted in blocking solution. After washing with 50 mM Tris pH 7.4, sections were incubated for 30 min with secondary antibodies: goat anti-mouse IgG1 conjugated with Alexa488 (Dianova GmbH, Hamburg, Germany) and goat anti-mouse IgG2a conjugated with Alexa633 (Life Technologies, Carlsbad, CA, United States). After additional washing, stained slides were covered with VECTASHIELD Vibrance antifade mounting medium containing 4′,6-diamidino-2-phenylindole (DAPI) for nuclear staining (Vector Laboratories, Burlingame, CA, United States) and imaged on the slide-scanner Axio Scan.Z1 (Zeiss, Oberkochen, Germany).

Images of the scanned slides were evaluated with the software QuPath version 0.2.3 (17). Scanned slides were annotated manually for CD20- and CD138-positive cells, while CD138-positive tubular epithelial cells were excluded. Section area was determined by outlining the biopsies using the polygon tool. Finally, the number of CD20- and CD138-positive cells was calculated per section area.

### Injury Scores

Histopathological changes were graded according to the Banff classification score of 2013 and 2017 for renal transplant biopsies in the process of routine diagnosis (18, 19). Selected clinical parameters from the time point of biopsy collection were retrospectively investigated. In addition, transplantation relevant parameters, such as cold ischemia time (CIT) and data of the donors (i.e., age, hypertension, serum creatinine, reanimation, sepsis, and acute renal failure (ARF) events before renal transplantation and renal inflammation) were included for correlation analysis with the results of the gene expression analysis.

## Results

### Gene Expression in Zero-Time Biopsies Is Dependent on Donor Type

**FIGURE 1:**
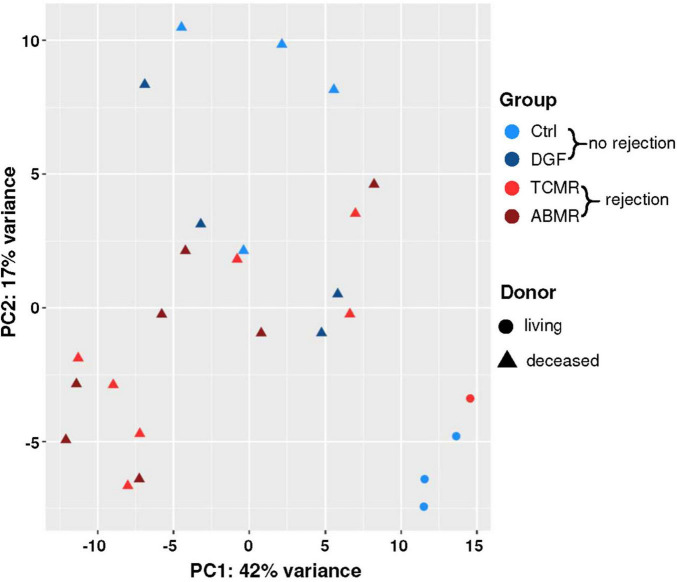
Principal component analysis (PCA) plot of NanoString expression data. The plot of the first two principal components (PC1 and PC2), calculated from the expression data of the 100 genes with the highest variance across all 26 samples. Axis labels report the percentage of total variance explained by each component. Biopsies without later rejection were indicated with blue symbols [Ctrl = light blue; delayed graft function (DGF) = dark blue] and those with later rejection with red symbols [T-cell mediated rejection (TCMR) = bright red, antibody-mediated rejection (ABMR) = dark red]; living donors were denoted using circles and deceased donors using triangles.

**FIGURE 2:**
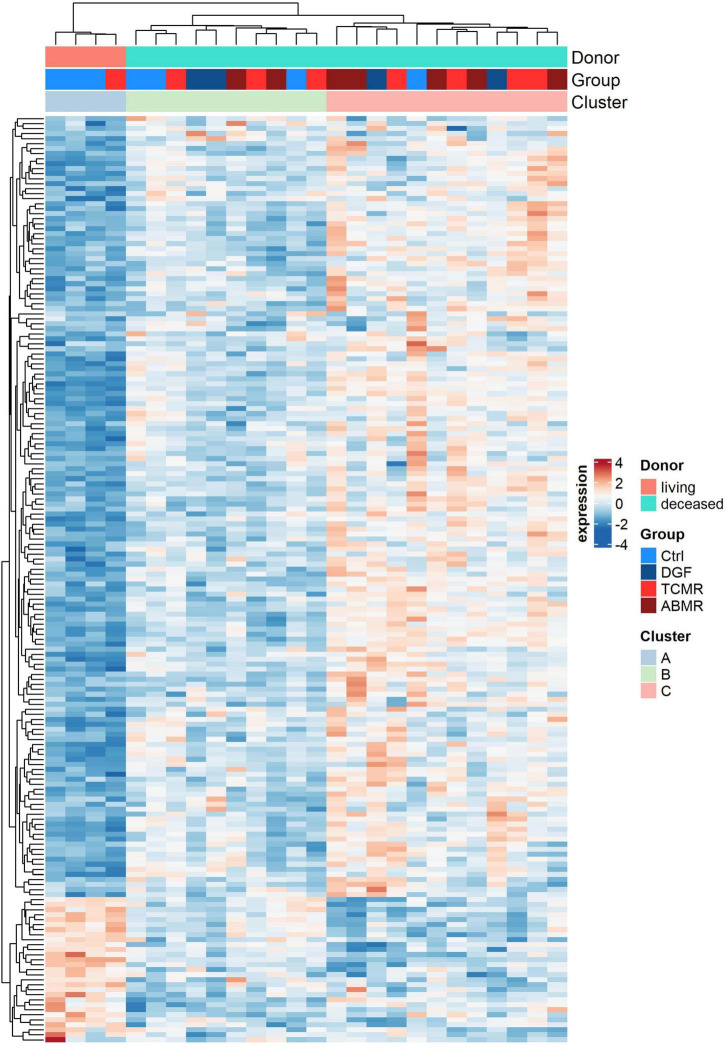
Expression heatmap. Expression heatmap of the 185 genes of the NanoString Human Organ Transplant Panel differentially expressed (adjusted *p* < 0.05) between zero-time biopsies of living (light red) and deceased (turquoise) donors. Two additional color-coded bars on top show sample outcome and membership into three identified clusters. In the heatmap red denotes upregulated genes and blue downregulated genes.

Histopathologic examination of the zero-time biopsies revealed no significant differences in kidney grafts with different outcomes (Supplementary Data 1). Therefore, we investigated whether differences in gene expression may predict complications, such as DGF, TCMR, or ABMR. The expression profiles of transplantation-related genes were analyzed in 26 zero-time biopsies that later developed DGF, TCMR, ABMR or had normal renal function throughout 1 year after transplantation and showed no signs of rejection (Ctrl) using a NanoString B-HOT panel. Expression analysis revealed that irrespective of the complication group, the greatest differences were observed when comparing donor types. Indeed, principal component analysis of expression data showed a clear separation between biopsies from LDs and those from DDs (Figure 1). Furthermore, differential expression analysis including factors “donor” and “later rejection (yes/no)” of the samples, revealed 185 differentially expressed genes between DD and LD biopsies (adjusted *p* < 0.05) (Figure 2 and Supplementary Data 5). Instead, only 11 genes were differentially expressed (adjusted *p* < 0.05) in samples later showing a rejection (TCMR or ABMR) versus samples showing no rejection (Ctrl or DGF).

### Genes Differentially Expressed in Zero-Time Biopsies From DD Versus LD Were Enriched in the Pathway “Post-translational Protein Modification”

**FIGURE 3:**
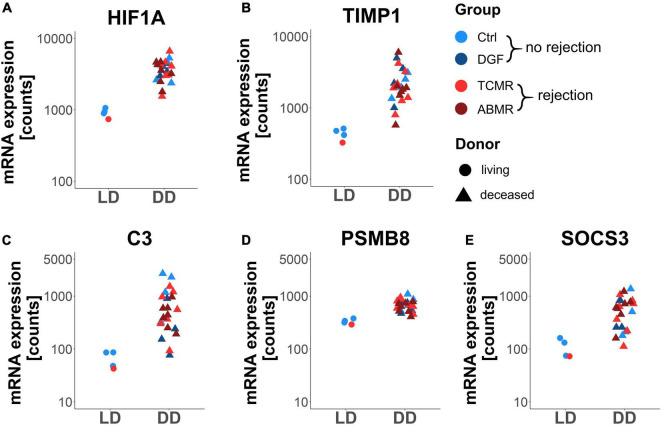
Expression of the top 5 differentially expressed genes between deceased (DD) and living donors (LD) associated with the enriched pathway “post-translational protein modification”. Dot plots showing gene expression (normalized counts) of the genes HIF1A **(A)**, TIMP1 **(B)**, C3 **(C)**, PSMB8 **(D)**, and SOCS3 **(E)** in zero-time biopsies of living (LD) and deceased (DD) donors. Adjusted *p* < 0.05 for all shown genes.

In order to further characterize the differentially expressed genes, we performed functional enrichment analysis using Gene Ontology Biological process terms and the set of NanoString B-HOT panel genes as background. Interestingly, considering the 185 differentially expressed genes between DD and LD, only “GO: 0043687 post-translational protein modification” was significantly enriched (adjusted *p* < 0.05). Fifteen of the 185 genes were assigned to this pathway (Table 2). Among them, HIF1A, TIMP1, C3, PSMB8, and SOCS3 were the genes with the highest fold changes and showed higher expression in DD regardless of the group (Figure 3). In addition to being involved in post-translational modifications, these genes are also involved in many other pathways, such as hypoxia-induced processes (GO: 0061418, GO: 0001666, and GO: 0071456; e.g., HIF1A, PSMB8, PSME1, and PSME2), extracellular matrix organization (GO: 0030198, GO: 0043062; TIMP1, TNC, VCAN, and FN1), complement-dependent processes (e.g., GO: 0006956, C3) and pathways related to ubiquitinylation (GO: 0016567; PSMB8, PSME1, PSME2, PSMB10, SOCS3, ASB15, and KLHL13) (Supplementary Data 6). Almost all of the 15 genes were also found in the stress response pathway (GO: 0006950). However, none of these other pathways were significantly enriched. Since one of the major differences between DD and LD was the duration of the CIT [median CIT (range) (min); LD: 0 (0–157) vs. DD: 769 (402–1,080)], we assessed the correlation between CIT and the expression levels of the 15 genes of the enriched “post-translational modification” pathway (Table 2) and with the top 20 of the 185 differentially expressed genes between DD and LD (Table 3). In both cases, the majority of genes showed significant correlations with CIT. Furthermore, 125 (67.6%) of the 185 differentially expressed genes of the comparison of DD vs. LD were significantly correlated with CIT, indicating that CIT was an important stimulator/regulator of these genes.

**TABLE 2:** Genes differentially expressed between deceased (DD) and living donors (LD) associated with the significantly enriched pathway (adjusted *p* = 0.01) “GO: 0043687 post-translational protein modification”.

Gene name	Significance DD vs LD (adj. *p*-value)	DD vs LD (log2 Fold change)	Corr. Coeff. with CIT (Spearman’s rank correlation)
HIF1A	7.08e-12	2.06	0.628***
TIMP1	5.02e-08	2.55	0.401*
C3	8.94e-08	3.78	0.420*
PSMB8	5.81e-06	1.09	0.583**
SOCS3	2.66e-05	2.41	0.413*
ASB15	7.77e-05	–2.35	−0.524**
PSME2	1.89e-04	0.71	0.513**
PSMB10	4.44e-04	1.06	0.640***
APOL1	0.004	0.82	0.614***
TNC	0.004	1.84	0.570**
VCAN	0.010	1.62	0.674***
APOE	0.011	–1.12	–0.382
PSME1	0.028	0.38	0.489*
FN1	0.029	1.10	0.327
KLHL13	0.045	0.51	0.141

CIT, cold ischemia time. *p < 0.05. **p < 0.01. ***p < 0.001.

**TABLE 3:** Correlation of the top 20 differentially expressed genes between deceased (DD) and living donors (LD)with cold ischemia time (CIT).

Gene name	Significance DD vs LD (adj. *p*-value)	DD vs LD (log2 Fold change)	Corr. Coeff. with CIT (Spearman’s rank correlation)
SERPINA3	1.56e-20	7.42	0.239
ALDH3A2	4.72e-15	–1.66	−0.544**
OSMR	9.08e-14	2.24	0.604**
S100A9	4.21e-13	4.13	0.395*
HIF1A	7.08e-12	2.11	0.628***
LTF	4.60e-10	4.95	0.267
BCL3	5.15e-10	2.4	0.660***
PLAAT	3.74e-09	2.48	0.483*
RARRES1	4.28e-09	4.13	0.489*
C1QB	5.26e-09	2.25	0.636***
JAK1	5.26e-09	0.668	0.413*
CD163	6.40e-09	2.80	0.462*
IFITM3	2.42e-08	1.92	0.379
C1S	2.52e-08	1.90	0.573**
FPR1	2.52e-08	2.41	0.521**
SOD2	3.13e-08	2.82	0.473*
BCL6	4.86e-08	2.14	0.458*
TIMP1	5.02e-08	2.55	0.401*
STAT3	5.07e-08	1.08	0.439*
S100A8	6.55e-08	4.08	0.284

CIT, cold ischemia time. *p < 0.05. **p < 0.01. ***p < 0.001.

### The Expression Profiles of DDs Were Associated With the Age of the Donors

Hierarchical clustering of the expression of the 185 differentially expressed genes between DD and LD (Figure 2) suggested grouping samples into three clusters: one (cluster A) of LD samples and two (clusters B and C) of DD samples. Most of the genes that were expressed at lower levels in cluster A were expressed more strongly in cluster B and even more strongly in cluster C. With regard to outcome, it is noticeable that more samples belonged to the Ctrl group in cluster B than in cluster C (Table 4). In contrast, the proportion of samples with ABMR was higher in cluster C (Table 4). CIT was significantly lower in DD cluster B compared to cluster C. (Table 4). No significant differences were observed between clusters B and C for other clinical parameters that include the occurrence of ARF, sepsis, hypertension, or the need for reanimation (Table 4). However, the age of donors in cluster B was significantly higher, with median age of 25 years higher than that of samples in cluster C (Table 4).

**TABLE 4:** Distribution of outcome and clinical parameters of samples belonging to the two DD clusters (B, C) of Figure 2.

		Deceased donors (DD)	
		Cluster B	Cluster C
Outcome	Ctrl	3/10 (30%)	1/12 (8.3%)
	DGF	2/10 (20%)	2/12 (16.7%)
	TCMR	3/10 (30%)	4/12 (33.3%)
	ABMR	2/10 (20%)	5/12 (41.7%)
	Rejection	5/10 (50%)	9/12 (75%)
Clinical parameters	ARF	6/10 (60%)	9/12 (75%)
	Reanimation	8/10 (80%)	6/12 (50%)
	Sepsis	2/10 (20%)	3/12 (25%)
	Hypertension	5/10 (50%)	4/12 (33.3%)
	CIT *[min]	609 (402; 1008)	868 (480; 1080)
	Donor age (**)	61 (36; 76)	36 (25; 65)

The distribution of donor outcome and categorical clinical parameters are provided in terms of proportion with respect to the total number of samples within each cluster (with the associated percentage within brackets). For quantitative clinical parameters, the median value (with minimum and maximum values within brackets) is reported. Ctrl, control; DGF, delayed graft function; TCMR, T-cell mediated rejection; ABMR, antibody-mediated rejection; ARF, acute renal failure; CIT, cold ischemia time. *p < 0.05; **p < 0.01 (Wilcoxon rank-sum test).

### Immunoglobulin Genes Were Upregulated in Zero-Time Biopsies From Patients Later on Developing Rejection

**FIGURE 4:**
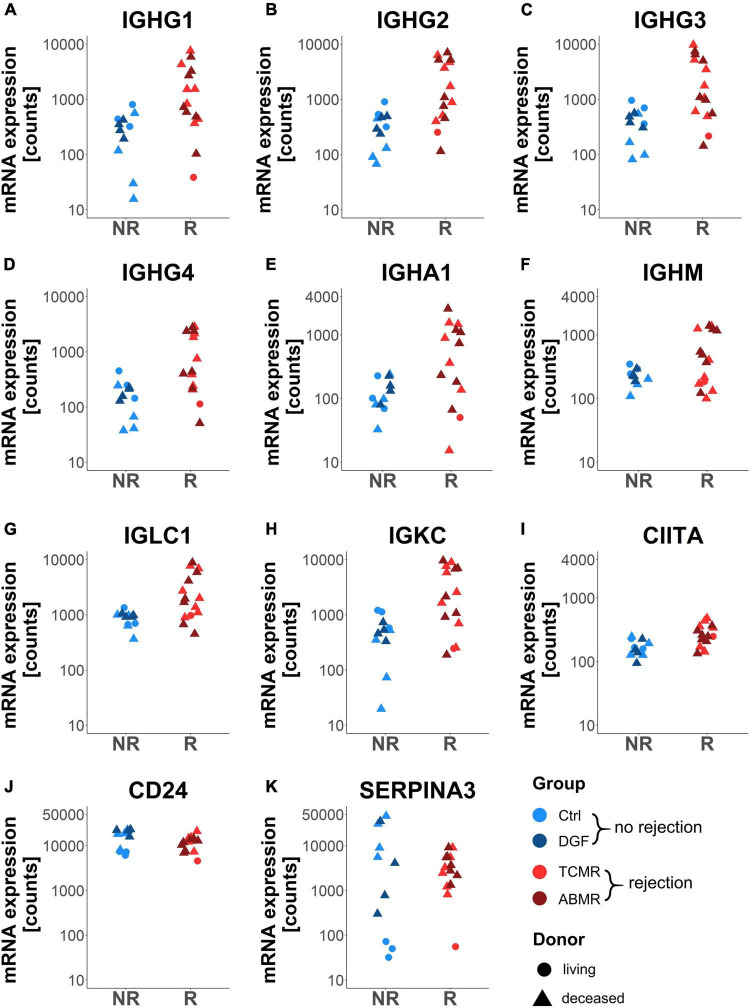
Dot plots of genes differentially expressed in zero-time biopsies of patients later developing rejection (R) versus no rejection (NR). Dot plots showing gene expression of the genes IGHG1-4 **(A–D)**, IGHA1 **(E)**, IGHM **(F)**, IGLC1 **(G)**, IGKC **(H)**, CIITA **(I)**, CD24 **(J)**, and SERPINA3 **(K)** in zero-time biopsies. Adjusted *p* < 0.05 for all shown genes.

While still adjusting for donor type (DD vs. LD), differential expression analysis was performed to compare samples developing rejection events later on to samples without rejection (controls and cases that developed DGF). In contrast to the high number of differentially expressed genes between DD and LD, only 11 genes were significantly differentially expressed in zero-time biopsies with later rejection (Table 5 and Figure 4). Interestingly, 8 of these belonged to genes coding for immunoglobulin chains, i.e., four IgG heavy chains (Figures 4A–D), one IgA heavy chain (Figure 4E), one IgM heavy chain (Figure 4F), an immunoglobulin lambda chain (Figure 4G), and kappa light chain (Figure 4H). With lower fold change CIITA (Figure 4I), coding for a protein involved in transactivation of class II major histocompatibility complex (MHCII) was differentially expressed. All immunoglobulin chains and CIITA were more strongly expressed in transplants with later rejection events. In contrast, CD24 (Figure 4J), coding for a surface protein expressed on mature granulocytes and B-cells and modulating growth and differentiation signals to these cells, and SERPINEA3 (Figure 4K), coding for a serine protease inhibitor, were expressed at a lower level in the rejection group compared to the no-rejection group. None of the genes differentially expressed in rejection vs. no-rejection groups significantly correlated with CIT (Table 5). Functional pathway enrichment analysis for the genes differentially expressed in the rejection vs. no-rejection group resulted in significant enrichment of 62 pathways (adjusted *p* < 0.05), with the top ones being: GO: 0006911 phagocytosis, engulfment; GO: 0010324 membrane invagination; GO: 0099024 plasma membrane invagination; GO: 0006958 complement activation, classical pathway; GO: 0008037 cell recognition; GO: 0002455 humoral immune response mediated by immunoglobulin (Supplementary Data 7).

**TABLE 5:** Correlation of the differentially expressed genes between rejection (R) and no-rejection (NR) with cold ischemia time (CIT).

Gene name	Significance R vs NR (adj. *p*-value)	R vs NR (log2 Fold change)	Corr. Coeff. with CIT (Spearman’s rank correlation)
IGHG2	6.73e-06	3.01	0.288
IGHG3	6.73E-06	2.98	0.286
IGKC	6.73E-06	3.11	0.284
IGHG4	7.74E-06	2.86	0.297
IGHA1	0.00064	2.36	0.364
IGHG1	0.00064	2.85	0.275
IGLC1	0.00065	1.78	0.364
IGHM	0.016	1.47	0.232
CD24	0.033	–0.60	0.266
SERPINA3	0.036	–1.78	0.239
CIITA	0.048	0.70	0.302

### CD20-Positive B-Cells and Plasma Cells Are More Abundant in Zero-Time Biopsies From Renal Transplants Experiencing Later Rejection Episodes

**FIGURE 5:**
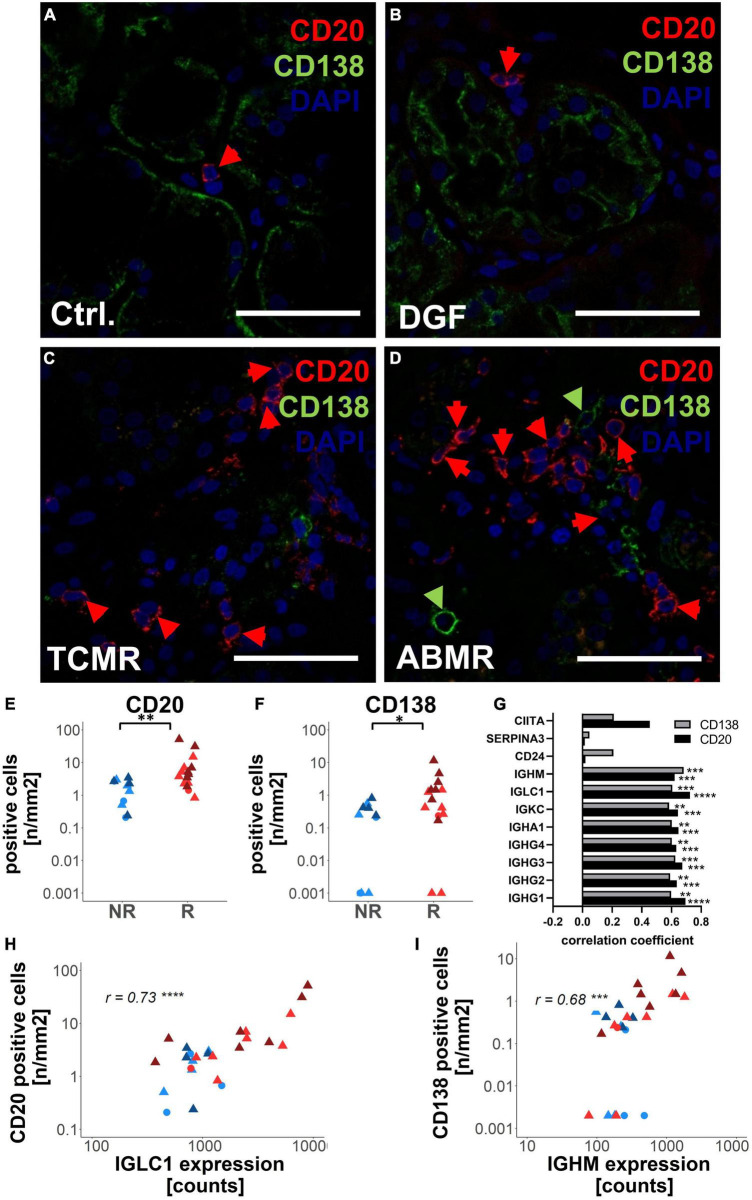
Analysis of antibody-producing cells in zero-time biopsies with the different later outcomes. CD20-positive B-cells and CD138-positive plasma cells were analyzed using immunofluorescence microscopy in zero-time biopsies of Ctrl **(A)**, delayed graft function (DGF) **(B)**, T-cell mediated rejection (TCMR) **(C)**, and antibody-mediated rejection (ABMR) **(D)**. Examples of CD20-positive cells were marked by red arrows and CD138-positive cells by green arrows. The numbers of CD20-positive cells **(E)** and CD138-positive **(F)** per biopsy area were shown in biopsies that later on developed rejection (R) or no rejection (NR). Correlation between the expression levels of differentially expressed genes between rejection and no rejection was shown **(G)**. Histograms for correlation of the number of CD20-positive cells with the gene expression level of IGLC1 **(H)** and numbers of CD138-positive cells with gene expression levels of IGHM **(I)** in zero-time biopsies were shown. Scale bar represents 50 μm; r: Spearman’s rank correlation coefficient; *p < 0.05; **p < 0.01; ***p < 0.001; ****p < 0.0001.

Since immunoglobulins were expressed by mature B cells and plasma cells, we evaluated the abundance of CD20-positive B-cells and CD138-positive plasma cells in FFPE sections of the zero-time biopsies using immunofluorescence microscopy. While in zero-time biopsies from Ctrl and DGF, CD20- and CD138-positive cells were detected only sporadically (Figures 5A,B), in TCMR, and in ABMR, these cells often occurred locally clustered (Figures 5C,D). Consequently, the number of CD20- (Figure 5E) and CD138-positive cells (Figure 5F) per section area was significantly higher in samples from transplants with later rejection events. In addition, the expression of immunoglobulins correlated well with numbers of CD20- and CD138-positive cells (Figure 5G). The highest correlation with the number of CD20-positive B-cells was found for the expression of IGLC1 (Figure 5H) and with the number of CD138-positive cells for the expression of IGHM (Figure 5I).

### Genes Associated With Later Rejection Were More Strongly Expressed in Kidneys of Donors With Pre-transplant ARF

**FIGURE 6:**
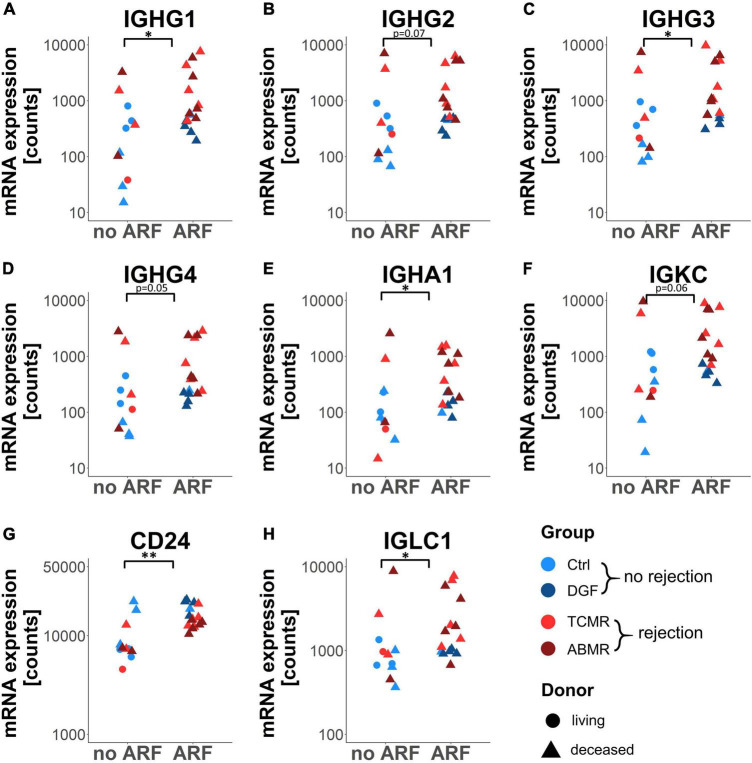
Dot plot of eight selected genes differentially expressed between rejection and no rejection grouped according to the presence of acute renal failure (ARF). Dot plots showing gene expression of the genes IGHG1–4 **(A–D)**, IGHA1 **(E)**, IGKC **(F)**, CD24 **(G)**, and IGLC1 **(H)** in zero-time biopsies. *p < 0.05; ^**^p < 0.01.

Since genes differentially expressed in rejection vs. no-rejection groups did not correlate with CIT, we investigated whether gene expression was correlated with other pre-transplant parameters. No correlation could be detected between gene expression levels and the need for reanimation or with the occurrence of sepsis in the donors (data not shown). In contrast, gene expression levels of 5 differentially expressed genes (i.e., IGHG1, IGHG3, IGHA1, CD24, and IGLC1) were significantly associated with the occurrence of ARF prior to the explanation of the organs of donors and three more genes (i.e., IGHG2, IGHG4, and IGKC) showed a strong tendency toward this association (Figure 6).

## Discussion

After kidney transplantation, impaired kidney function due to DGF or rejection events, such as TCMR and ABMR, occurs on regular basis. At worst, this can lead to loss of the transplant. Graft loss due to these complications partly depends on factors related to the recipient but possibly also on the organs of donors. Suri et al. estimated that characteristics of donors account for 35–45% of the variability of early graft function (20). In the past, various studies could not produce consistent results on the predictive value of histological changes that were already present in the organs of donors at the time of transplantation with regard to later complications or graft loss (1–4). In our small collective of zero-time biopsies, analysis of histological changes using Banff classification failed to detect changes that can be used as predictors for the later outcomes. The investigation of mRNA expression profiles, with the aid of multiplex analyses, such as microarrays or NanoString analyses, enables the recording of a huge number of parameters simultaneously. While most existing studies compare the gene expression profiles of healthy grafts with rejection biopsies (9, 21–25), there are so far only a few that examine zero-time biopsies using either few pre-selected genes (8, 26) or multiplex arrays (7, 27–29). Earlier gene expression studies using zero-time biopsies focused on differences between LDs and DDs (29), gene expression profiles in biopsies with histological changes (27) or DGF (7, 28). Although we included only a low number of zero-time biopsies derived from LDs in our study, we could confirm earlier findings showing a differential gene expression pattern comparing DD vs. LD (29). The high number of 185 differentially regulated genes when comparing DD with LD indicates that the donor type significantly influences expression. The extent to which these differentially regulated genes influence the subsequent outcome cannot be determined on the basis of our study. Surprisingly, only one pathway was significantly enriched, suggesting that conditions in DD were complex and induced no specific pathway. Several studies on kidney transplantation could demonstrate that graft survival was improved in recipients receiving a transplant from an LD (30, 31). One major difference between LD and DD is the lack of CIT in LD. Our data clearly showed that the expression level of the majority of the genes differentially regulated in DD vs. LD correlated with the duration of CIT. The importance of the hypoxic trigger is supported by overexpression of HIF1A in DD, which is the gene with the 5th highest significance level, even if this transcription factor is mainly controlled by oxygen-dependent stabilization on protein level. HIF1A stabilization occurs by post-translational protein modification, in which the only pathway enriched in DD compared to LD is involved and which includes several proteasomal proteins. Earlier studies reported that prolonged CIT is a known risk factor for allograft loss (32). However, in our study, we searched for gene expression changes in zero-time biopsies that may predict a later complication. In contrast to previous studies, we specifically investigated zero-time biopsies from transplants that later developed either DGF, TCMR, or ABMR, since the number of transplants that developed DGF or rejection events was very low in consecutive cohorts under investigation in earlier studies (7). The comparison of all 4 different groups showed no significantly changed expression patterns for the respective complication groups DGF, TCMR, and ABMR, which may be also due to the low sample numbers per group. Within the DD samples, two different clusters could be identified showing different levels of upregulation compared to the LD group. Although one cluster contained more controls and the other more ABMR, the two clusters did not differ significantly in terms of outcome and clinical parameters. However, there was a significant difference in the age of donor. Since most of the genes monitored on the NanoString B-HOT panel are related to the immune system, the observed age-dependent differences might reflect the changes described as immunosenescence, which can affect graft outcome (33). However, in our pilot study, assignment to one of the two gene expression clusters B and C did not allow a clear prediction regarding the subsequent outcomes. Since we observed that the gene expression profile in DGF was similar to Ctrl and expression profiles in TCMR resembled those of ABMR, we decided to compare biopsies with and without future rejection episodes to assess whether gene expression in zero-time biopsies can predict future rejection. Only 11 genes were found to be differentially expressed between rejection and no rejection samples, 8 of which coded for immunoglobulin chains. Functional pathway enrichment analysis of these genes highlighted the enrichment of more than 60 pathways that are mainly associated with antibody-mediated responses, such as activation of the classical complement pathway or the antibody-mediated humoral immune response. Using immunofluorescence microscopy, we demonstrated that this increased expression of immunoglobulins in zero-time biopsies later developing rejection was due to an increased presence of CD20-positive B-cells and CD138-positive plasma cells. B-cell rich infiltrates in allograft biopsies were not associated with worse outcomes in types I and II acute rejection, but a possible contribution of B-cells to allograft rejection could not be excluded in a previous study (34). Another study reported the protective effects of intra-graft CD20-positive cells in cell-mediated rejection (35). Especially transitional B-cells, a subgroup of CD20-positive cells expressing CD24*^high^*, were found to play a protective role (36). Interestingly, in our gene expression analysis, CD24 was one of the differentially regulated genes in the rejection group and exhibited a lower expression compared to the no rejection group, thus indicating that protective B-cell subpopulations might be less abundant. However, a meta-analysis of the effects of CD20-positive B-cell infiltration during allograft rejection revealed an increased risk of graft loss (37). In principle, immunoglobulins play a role in complement-mediated immune response and ABMR (38). In ABMR, donor-specific antibodies attack the graft. However, in zero-time biopsies, only donor B- and plasma cells can be detected in the kidney, which would only attack the kidney if autoantibodies were built. Therefore, the meaning of increased immunoglobulin expression remains unclear. Studies investigating gene expression using microarray analysis on biopsy samples with established rejection also observed significantly increased upregulation of immunoglobulins (39). Interestingly, the expression of immunoglobulin was not triggered by hypoxia but associated with prior ARF. Clinical studies reported that the adjusted relative risk for DGF was increased by severity of ARF in the pre-transplantation of the kidneys of donors (40) but was not associated with long-term graft failure (41). However, ARF in pre-transplant kidneys is a potential trigger for the expression of rejection-promoting genes. However, other inducers not yet known cannot be excluded.

In our study, transplant zero-time biopsies that later developed DGF showed comparable gene expression to controls or even a slight downregulation of transplantation-relevant genes, confirming similar observations reported by Hauser et al. (28). However, microarray analysis followed by unsupervised analysis clustered zero-time biopsies into 3 different groups, one of which with a significantly higher incidence of subsequent DGF (7). This indicates that differences in gene expression may also play a role in the development of DGF, but may not have been included in our NanoString array, which is limited to 770 genes. This study is limited by the small number of biopsies examined per subsequent transplant complication. The differences observed in gene expression between rejection and no rejection are relatively minor and the type of rejection in this study cannot be clearly predicted. The restriction to 770 pre-selected genes carries the risk that other genes, which are important for later rejection, will not be detected. Furthermore, the ratio of transplants from LDs to DDs differed between groups and was highest in the control group, while the ABMR and DGF groups exclusively contained DDs. However, also adjusting for donor type, significantly differentially expressed genes between rejection and no rejection group could be identified.

In conclusion, this pilot study clearly showed that gene expression analysis can be performed using standard protocol zero-time biopsies without the need to take an extra biopsy for this purpose. We suggest that the expression of inflammation-associated genes in the transplant, already determined at the time of donation, at least in part influences the transplant outcome, which is already determined at the time of donation. Furthermore, the upregulation of immunoglobulin genes in zero-time biopsies may indicate an increased risk for subsequent rejection.

## Data Availability Statement

The original contributions presented in the study are included in the article/Supplementary Material. Further inquiries can be directed to the corresponding author.

## Ethics Statement

The studies involving human participants were reviewed and approved by The Ethics Committee of the Friedrich-Alexander-University Erlangen-Nürnberg, Erlangen, Germany. Written informed consent for participation was not required for this study in accordance with the national legislation and the institutional requirements.

## Author Contributions

EV collected and analyzed the data, performed the experiments, and wrote the manuscript. SS analyzed the data and performed the experiments. MB-H collected and analyzed the data and edited the manuscript. KA collected data and edited the manuscript. EB edited the manuscript. JM-D and KH collected the clinical data. FF and MA analyzed the expression data and wrote the manuscript. CD initiated the study, collected and analyzed the data, and wrote the manuscript. All authors contributed to the article and approved the submitted version.

## Conflict of Interest

The authors declare that the research was conducted in the absence of any commercial or financial relationships that could be construed as a potential conflict of interest.

## Publisher’s Note

All claims expressed in this article are solely those of the authors and do not necessarily represent those of their affiliated organizations, or those of the publisher, the editors and the reviewers. Any product that may be evaluated in this article, or claim that may be made by its manufacturer, is not guaranteed or endorsed by the publisher.
