# Supplementary material for: Can Gene Expression Analysis in Zero-Time Biopsies Predict Kidney Transplant Rejection?
Source: Front Med (Lausanne). 2022 Mar 30;9:793744. doi: 10.3389/fmed.2022.793744 (PMC9005644; doi:10.3389/fmed.2022.793744)
Supplement: Supplementary file 1 [file Table_1.pdf]

Supplementary data 1: Banff classification of zero-time biopsies

	Nr.	IFTA	g	i	ti	t	v	ptc	aah	cg	ci	ct	cv	mm
Ctrl	1	0	0	0	0	0	0	0	0	0	0	1	0	0
	2	0	0	1	0	0	0	0	0	0	0	1	0	0
	3	0	0	0	0	0	0	0	0	0	0	1		0
	4	1	0	0	0	0	0	0	0	0	1	1	1	0
	5	1	-	-	-	-	-	-	-	-	-	-	-	-
	6	0	0	0	0	0	0	0	0	0	0	1	0	0
	7	0	0	0	0	0	0	0	0	0	0	1	0	0
DGF	1	0	0	0	0	0	0	0	1	0	0	1	1	1
	2	0	-	-	-	-	-	-	-	-	-	-	-	-
	3	1	0	0	1	0	0	0	2	0	1	1	0	0
	4	0	0	0	0	0	0	0	0	0	0	0	0	0
TCMR	1	0	0	0	0	0	0	0	0	0	0	1	0	0
	2	0	0	0	1	0	0	0	0	0	0	1	0	0
	3	0	-	-	-	-	-	-	-	-	-	-	-	-
	4	1	0	0	1	0	0	0	2	0	1	1	2	0
	5	1	0	0	0	0	0	0	2	0	0	1	2	1
	6	0	0	0	0	0	0	0	0	0	0	1	0	0
	7	1	0	0	0	0	0	0	3	0	1	1	2	1
	8	0	0	0	0	0	0	0	1	0	0	1	2	1
ABMR	1	0	0	0	0	0	0	0	0	0	0	0	0	0
	2	0	-	-	-	-	-	-	-	-	-	-	-	-
	3	0	0	0	0	0	0	0	0	0	0	0	0	1
	4	0	0	0	0	0	0	0	0	0	0	0	0	0
	5	1	0	0	0	0	0	0	0	0	0	1	0	0
	6	1	0	0	0	0	0	0	0	0	0	1	0	0
	7	1	0	0	1	0	0	0	2	0	1	1		0

IFTA= Interstitial Fibrosis and Tubular Atrophy; g= Glomerulitis; i=Interstitial Inflammation; ti= Total Inflammation; t= Tubulitis; v= Intimal Arteritis; ptc= Peritubular Capillaritis; aah= Hyaline Arteriolar Thickening; cg= Glomerular Basement Membrane Double Contours; ci= Interstitial Fibrosis; ct= Tubular Atrophy; cv= Vascular Fibrous Intimal Thickening; mm= Mesangial Matrix Expansion
