# Supplementary material for: Can Gene Expression Analysis in Zero-Time Biopsies Predict Kidney Transplant Rejection?
Source: Front Med (Lausanne). 2022 Mar 30;9:793744. doi: 10.3389/fmed.2022.793744 (PMC9005644; doi:10.3389/fmed.2022.793744)
Supplement: Supplementary file 2 [file Table_2.pdf]

Supplementary data 2: Kidney Recipient characteristics

	Ctrl	DGF	TCMR	ABMR	Total
**Recipient** n=**28**	7	4	8	7	26
Men (%)	7 (100%)	3 (75%)	2 (29%)	4 (57%)	16 (62%)
Age at transplant (years)	56 ±13	51 ± 24	47 ± 14	47 ± 14	50 ± 15
Body mass index (kg/m²)	25 ± 6	24 ± 4	26 ± 4	23 ± 4	24 ± 4
Smoker	3	1	1	1	6
Diabetes	2	1	0	2	5
Hypertension	7	3	5	5	20
CAD	1	2	1	1	5
Diagnosis (days after Tx)	401 ± 35	15 ± 6	493 ± 223	127 ± 139	296 ± 236
Serum Creatinine Diagnosis (mg/dl)	1.24 ± 0.26	5.15 ± 3.43	2.95 ± 2.20	3.70 ± 1.95	3.01 ± 2.36
eGFR (0.5 year)	70 ± 18.8	74.2 ± 11.2	54 ± 13	38.1 ± 16.1	59.1 ± 20.9
eGFR (1 year)	68.6 ± 19.6	71.3 ± 18	48.5 ± 20.9	39 ± 18.7	56.9 ± 23.5
eGFR (2years)	68.1 ± 19.7	75.1 ± 20.3	30.7 ± 18.2	47.6 ± 11.2	55.4 ± 26.3

Ctrl= control, DGF= delayed graft function, TCMR= T-cell mediated rejection, ABMR= antibody-mediated rejection, CAD= coronary artery disease, Tx= Transplantation; Ranges are stated as mean ± SD
