# Supplementary material for: Can Gene Expression Analysis in Zero-Time Biopsies Predict Kidney Transplant Rejection?
Source: Front Med (Lausanne). 2022 Mar 30;9:793744. doi: 10.3389/fmed.2022.793744 (PMC9005644; doi:10.3389/fmed.2022.793744)
Supplement: Supplementary file 3 [file Table_3.pdf]

Supplementary data 3: Banff classification of follow-up biopsies at time-point of diagnosis

	Nr.	Baseline disease	Diagnostic findings	IFTA	g	i	ti	t	v	ptc	aah	cg	ci	ct	cv	mm
Ctrl	1	Diabetic Nephropathy	no dysfunction or rejection	0	0	0	0	0	0	0	0	0	0	1	0	0
	2	IgAN	no dysfunction or rejection	0	0	0	0	0	0	0	0	0	0	1	0	0
	3	Diabetic Nephropathy	no dysfunction or rejection	0	0	0	0	0	0	0	0	0	0	0	0	0
	4	Unknown	no dysfunction or rejection	1	0	1	1	0	0	0	0	0	1	1	0	0
	5	Nephrocalcinosis	no dysfunction or rejection	1	0	0	1	0	0	0	2	0	1	1	3	0
	6	hypert. ischemic Nephropathy	no dysfunction or rejection	1	0	0	0	0	0	0	0	0	1	1	0	0
	7	ADPKD	no dysfunction or rejection	0	0	1	1	0	0	0	0	0	0	1	2	0
DGF	1	FSGS	no evidence for rejection	0	0	0	0	0	0	0	0	0	0	1	0	0
	2	Unknown	no evidence for rejection	0	0	0	0	0	0	0	1	0	0	0	0	1
	3	IgAN	no evidence for rejection	0	0	1	1	0	0	0	2	0	0	1	0	0
	4	Cystinosis	no evidence for rejection	0	0	1	1	0	0	0	0	0	0	1	0	0
TCMR	1	Vesicoureteral Reflux	chronic active IB & acute IB	3	0	3	3	3	0	1	2	0	3	3	3	3
	2	Bilateral shrunken kidney	acute IB	2	0	2	2	3	0	0	2	0	2	2	1	0
	3	Unknown	acute IB	2	0	3	3	3	0	1	2	0	2	2	3	0
	4	Granulomatosis w. Polyangiitis	acute IA	1	0	2	2	2	0	0	2	1	1	1	2	1
	5	Hypertensive Nephropathy	acute IIA		0	3	3	3	1	0	2	0			3	0
	6	Hypertensive Nephropathy	chronic active IB & acute IA	3	0	2	2	2	0	0	2	0	3	3	1	0
	7	Vesicoureteral Reflux	acute IA	1	0	2	2	2	0	0	1	0	1	1	0	0
	8	Benign Nephrosclerosis/ hypertensive Nephropathy	acute IIA	2	0	2	2	3	0	0	1					
ABMR	1	Glomerulosclerosis	acute Type II	0	1	1	1	1	0	1	0	0	0	1	0	0
	2	IgAN	acute Type II	1	1	1	1	0	0	2	2	0	1	1		1
	3	aHUS	acute Type II	0	0	0	0	0	0	2	0	0	0	1	0	0
	4	Diabetic-hypertensive NP	acute Type II	1	1	1	1	1	0	1	0	0	1	1	1	0
	5	Nephrosclerosis	acute Type II	0	0	0	0	0	0	2	0	0	0	1	0	0
	6	IgAN	acute Type II	0	0	0	0	0	0	1	0	0	0	0	0	0
	7	ADPKD	chronic active & acute Type II	1	2	1	2	1	0	1		3	1	1	2	2

IFTA= Interstitial Fibrosis and Tubular Atrophy; g= Glomerulitis; i=Interstitial Inflammation; ti= Total Inflammation; t= Tubulitis; v= Intimal Arteritis; ptc= Peritubular Capillaritis; aah= Hyaline Arteriolar Thickening; cg= Glomerular Basement Membrane Double Contours; ci= Interstitial Fibrosis; ct= Tubular Atrophy; cv= Vascular Fibrous Intimal Thickening; mm= Mesangial Matrix Expansion

Data already published in Vonbrunn et al. Multiplex gene analysis reveals T-cell and antibody-mediated rejection-specific upregulation of complement in renal transplants (9)
