# Supplementary material for: Can Gene Expression Analysis in Zero-Time Biopsies Predict Kidney Transplant Rejection?
Source: Front Med (Lausanne). 2022 Mar 30;9:793744. doi: 10.3389/fmed.2022.793744 (PMC9005644; doi:10.3389/fmed.2022.793744)
Supplement: Supplementary file 4 [file Table_4.pdf]

Description	Positive POS_A	Positive POS_B	Positive POS_C	Positive POS_D	Positive POS_E	Positive POS_F
Ctrl.	44887	16999	4171	963	155	71
Ctrl.	51111	17813	4408	1079	148	87
Ctrl.	40312	13967	3681	898	100	83
Ctrl.	58063	19332	5022	1142	181	91
Ctrl.	53068	17239	4460	1073	166	97
Ctrl.	52249	17443	4471	1138	168	80
Ctrl.	55480	17938	4571	1117	151	66
DGF	54658	18399	4599	1113	183	86
DGF	76071	23487	6194	1400	234	113
DGF	62084	18684	4751	1127	175	96
DGF	59007	19560	4799	1211	187	69
TCMR	53703	18532	4581	1056	165	82
TCMR	61546	21179	5301	1235	197	121
TCMR	40900	13494	3341	828	122	74
TCMR	43961	15572	3895	926	137	84
TCMR	55365	18438	4800	1162	152	87
TCMR	59405	18988	5003	1168	169	84
TCMR	57047	18696	5106	1227	176	96
TCMR	41937	13987	3380	894	132	53
ABMR	63618	21251	5479	1244	187	129
ABMR	67403	23229	5888	1354	219	115
ABMR	65430	22570	5540	1268	199	92
ABMR	80233	24553	6241	1544	238	131
ABMR	78264	24500	6404	1486	242	109
ABMR	78500	23972	6192	1521	234	118
ABMR	55355	18054	4431	1035	149	68

Negative<br>NEG_A	Negative<br>NEG_B	Negative<br>NEG_C	Negative<br>NEG_D	Negative<br>NEG_E	Negative<br>NEG_F	Negative<br>NEG_G
11	21	16	5	25	29	3
11	16	16	13	27	31	9
17	12	15	9	12	41	14
10	20	16	8	18	31	7
15	18	13	8	17	33	17
12	18	18	14	18	35	16
5	13	11	8	10	13	10
12	25	11	10	30	32	6
19	19	12	17	21	36	19
21	21	14	18	20	33	18
3	12	10	12	13	14	10
16	28	15	15	21	30	14
15	32	8	10	16	47	9
17	13	12	12	18	35	7
20	16	19	12	18	32	14
17	21	14	11	14	22	19
12	20	20	7	13	28	11
15	22	24	13	11	36	16
4	4	12	4	5	7	8
18	18	25	15	22	40	11
7	28	20	12	15	49	7
17	20	19	6	16	43	10
25	22	27	15	13	38	25
20	25	20	11	23	48	19
7	28	22	14	20	58	44
2	10	14	6	8	10	9

Negative	Housekeeping	Housekeeping	Housekeeping	Housekeeping	Housekeeping	Housekeeping
NEG_H	ABCF1	G6PD	GUSB	NRDE2	OAZ1	POLR2A
7	160	147	386	143	2257	576
16	318	210	958	225	4074	1080
15	669	396	1175	377	5203	1478
19	224	154	372	81	2142	499
12	506	399	1018	220	4885	1308
19	197	123	477	155	1777	669
13	163	125	506	119	1534	545
13	507	501	1284	287	5649	1467
16	367	236	694	207	2875	985
19	805	601	1430	437	7088	1955
15	163	167	325	97	855	403
22	370	289	976	242	3215	1084
14	438	280	928	234	4310	1211
15	862	511	1920	478	7705	2450
26	430	445	1212	461	3044	1603
19	196	121	541	146	2005	628
24	130	39	232	51	840	320
22	274	196	757	157	2431	730
19	292	273	715	166	2530	825
15	323	256	639	180	2855	779
17	107	123	226	59	1142	315
12	317	181	591	152	2920	971
36	234	225	475	142	1560	846
27	463	289	690	243	3996	1172
33	88	62	153	45	588	227
21	290	250	716	156	2829	847

Housekeeping	Housekeeping	Housekeeping	Housekeeping	Housekeeping	Housekeeping	Endogenous
PPIA	SDHA	STK11IP	TBC1D10B	TBP	UBB	ABCA1
3019	724	77	175	175	6376	180
5574	1977	119	290	324	13006	388
7958	2516	225	276	510	13226	384
3225	1109	83	144	149	6875	58
7607	2009	125	287	344	10747	487
2800	1601	78	152	161	5901	188
2728	973	60	121	157	5371	173
10118	2419	175	356	361	12303	610
4918	1894	115	280	217	9589	492
13712	3832	257	450	542	14924	643
2177	651	72	120	94	2135	182
5454	1478	175	302	326	8042	603
7604	2086	131	229	339	11989	278
11590	4703	279	451	645	19271	653
9530	1954	290	278	565	5034	371
2810	1159	54	151	151	6013	214
1347	353	39	109	66	2341	102
4313	1054	112	169	202	5237	271
6512	1338	92	180	208	7064	502
3905	1312	108	158	164	7053	282
2383	549	33	78	93	2382	123
5645	1414	112	187	196	8365	208
4074	966	133	165	187	4273	401
6861	2078	115	243	273	10335	281
1327	237	42	83	66	1164	49
4536	1146	93	231	192	6975	211

Endogenous ABCB1	Endogenous ABCC2	Endogenous ABCE1	Endogenous ACKR1	Endogenous ACTA2	Endogenous ACVR1	Endogenous ACVRL1
585	177	405	67	551	288	332
2182	912	556	13	1253	680	500
1262	417	941	27	4099	790	909
554	511	266	18	531	304	298
1500	478	633	93	3316	598	637
1081	504	314	22	941	346	256
889	424	240	7	586	256	251
1567	471	710	209	5888	679	1065
899	300	403	34	1975	335	422
1773	557	1118	133	3291	946	1099
356	162	212	14	1201	307	172
1418	275	725	142	4416	579	730
1272	567	605	33	1788	720	485
2247	1124	1122	117	4457	950	2913
919	945	1000	348	3120	648	1061
915	543	237	10	850	304	197
261	117	143	107	269	127	138
793	473	411	108	799	334	511
1060	572	578	20	2607	542	450
886	1220	328	26	746	408	273
293	266	178	19	309	109	156
699	332	451	174	1388	405	316
420	347	338	451	3254	379	869
1069	577	496	26	1090	477	952
130	137	125	27	438	99	142
944	321	383	116	1512	356	410

Endogenous ADAM8	Endogenous ADAMDEC1	Endogenous ADAMTS1	Endogenous ADGRL4	Endogenous ADORA2A	Endogenous AGER	Endogenous AGR2
14	15	3516	218	81	23	84
32	16	580	204	56	26	80
43	19	1191	316	69	40	63
29	11	1006	105	42	17	25
41	16	2031	254	72	35	47
27	13	394	94	27	22	40
21	9	188	110	19	25	16
39	8	7536	399	87	57	149
27	12	927	249	84	23	25
61	19	3540	462	155	31	91
23	10	1955	91	34	10	34
38	18	3807	278	96	39	61
33	19	1345	190	66	21	55
40	19	2301	492	132	75	115
67	15	9385	746	127	185	572
20	8	211	126	25	24	20
23	11	762	116	24	16	18
40	9	3124	240	63	34	168
22	2	1452	166	26	20	100
29	11	3257	171	48	26	122
27	15	1387	97	24	13	79
28	12	3742	202	40	24	78
44	14	1527	208	58	43	92
44	17	923	292	65	44	33
41	15	902	83	38	30	45
39	7	2421	180	38	27	100

Endogenous AGR3	Endogenous AGT	Endogenous AHR	Endogenous AICDA	Endogenous AIM2	Endogenous AIRE	Endogenous AKR1C3
19	1187	176	33	54	35	995
17	548	213	3	26	24	1350
22	8678	596	12	25	14	1312
13	2286	170	9	18	15	604
16	1461	532	7	29	20	699
7	426	134	6	12	20	454
12	242	73	8	14	13	501
32	1036	596	17	33	24	820
15	1007	325	8	29	20	495
33	1212	380	17	26	27	1102
5	1584	144	6	14	20	284
30	546	332	9	25	31	540
16	2185	262	8	19	15	1084
27	3419	701	14	19	27	687
68	1858	533	7	106	27	586
13	593	153	3	18	21	446
13	721	80	4	12	20	146
36	630	321	9	26	10	537
21	752	231	2	20	9	936
21	1747	234	6	15	13	934
14	418	131	9	23	12	302
22	2782	247	10	27	14	671
18	1238	292	11	28	29	418
23	1803	352	10	30	33	660
30	491	99	8	25	37	367
18	1712	340	10	37	15	545

Endogenous ALAS1	Endogenous ALDH3A2	Endogenous ALOX15	Endogenous ALOX5	Endogenous ANKRD1	Endogenous ANKRD22	Endogenous ANXA1
178	654	17	115	44	36	362
239	4680	8	90	17	6	1015
672	2725	18	320	14	7	970
308	680	7	84	14	5	323
516	1601	10	250	16	9	1119
179	2050	6	51	11	8	368
196	1918	4	41	7	3	384
581	1568	12	325	20	17	1397
271	1214	9	137	19	8	608
798	2612	8	314	32	12	1490
215	364	7	86	12	12	494
384	1301	11	264	34	9	1108
502	2066	4	118	20	7	566
797	3709	17	339	30	25	1348
486	1236	14	587	36	35	2590
177	2563	4	55	12	7	338
104	292	6	55	9	4	168
275	867	8	397	17	7	1168
293	1155	2	182	16	7	556
562	1336	7	84	13	7	357
148	352	10	68	25	8	316
313	849	12	170	17	9	483
270	571	8	315	30	6	817
476	1856	11	126	22	13	518
75	190	9	59	16	8	166
315	972	3	267	6	11	1073

Endogenous AOAH	Endogenous APOE	Endogenous APOL1	Endogenous APOL2	Endogenous APOLD1	Endogenous AQP1	Endogenous AQP2
16	5215	360	300	150	3713	121
40	22151	354	276	67	26744	707
48	23426	754	576	146	8065	1162
11	11980	303	221	83	5763	456
39	16062	750	480	405	20017	1952
20	14542	164	182	226	13445	783
14	14754	243	160	73	10957	451
78	10908	1077	650	242	24637	322
23	10599	449	410	73	23159	1462
35	13992	1263	696	193	42841	5826
22	8982	244	140	68	1071	67
86	6721	686	416	112	14746	444
27	25932	448	513	151	12509	888
67	32882	1007	805	321	37300	2142
73	21387	1016	533	378	3645	331
13	24769	181	178	125	13024	614
12	4631	171	178	43	1925	214
93	6927	608	337	115	10685	427
49	9107	365	384	129	13370	915
37	20347	352	296	84	15914	430
21	1947	184	143	58	1876	171
47	9060	500	439	111	7700	305
58	4494	800	428	184	3714	818
13	23591	539	467	142	12068	1358
14	1432	190	179	36	472	93
69	15999	634	318	127	7374	539

Endogenous AREG	Endogenous ARG1	Endogenous ARG2	Endogenous ARHGDIB	Endogenous ARRB2	Endogenous ASB15	Endogenous ATF3
10	14	638	658	63	60	182
10	22	570	890	99	369	563
15	25	2935	1846	188	101	105
6	22	517	401	61	106	92
16	15	1029	1619	158	37	114
10	21	376	406	57	164	450
7	8	736	380	55	205	121
20	26	1063	2184	165	56	207
10	20	1460	902	80	61	116
21	20	4061	1899	204	76	244
11	15	648	498	48	17	138
16	15	375	1783	144	26	192
11	18	987	853	142	235	99
27	19	1678	2007	231	125	361
64	36	1278	2197	191	46	395
5	21	360	416	57	352	122
3	13	131	365	32	17	73
4	22	365	1128	146	25	161
16	17	682	912	88	41	90
10	12	1558	727	123	252	125
12	18	297	320	36	32	65
14	14	302	783	88	53	162
24	25	406	1189	113	39	357
16	33	755	809	122	94	109
12	45	382	225	29	49	113
18	15	1057	1153	142	90	234

Endogenous ATM	Endogenous ATXN3	Endogenous AXL	Endogenous B2M	Endogenous B3GAT1	Endogenous BASP1	Endogenous BATF
166	205	120	11562	19	197	22
331	362	210	13729	28	196	9
569	641	404	23595	35	172	31
183	195	86	7776	25	66	7
464	518	305	28664	21	162	26
166	254	141	5495	29	63	7
167	174	102	6846	13	77	6
328	516	670	20935	26	287	23
305	354	210	10158	38	210	9
604	710	604	19028	39	588	28
110	171	166	4585	25	49	9
399	397	396	18679	41	437	27
294	364	223	18337	23	158	12
711	790	695	25934	26	187	39
544	673	427	33470	31	156	29
155	187	109	5092	19	74	7
88	116	68	2950	24	122	12
310	256	275	16625	30	239	24
265	170	185	16039	18	106	5
233	314	179	6006	22	98	9
90	95	112	4358	24	39	7
284	321	171	15401	35	245	22
217	239	339	9011	32	207	28
277	385	250	14096	30	105	15
88	82	64	1907	51	30	4
275	254	244	14155	15	292	22

Endogenous BATF3	Endogenous BAX	Endogenous BCL2	Endogenous BCL2A1	Endogenous BCL2L1	Endogenous BCL2L11	Endogenous BCL3
37	522	160	41	485	50	410
32	692	269	16	1319	140	260
35	1075	365	17	1608	217	791
19	416	131	7	749	51	566
37	1198	458	24	1959	136	619
21	371	258	10	834	101	84
15	275	166	5	607	86	83
45	1302	509	33	2291	180	1428
31	847	406	13	1243	117	513
49	1386	655	14	2805	274	1147
30	401	139	19	733	52	564
47	779	427	21	1452	113	906
31	810	221	11	1579	135	831
47	1894	563	16	3393	418	973
120	1296	528	57	1369	249	976
20	333	186	7	857	81	94
16	310	75	6	494	17	513
32	759	325	10	979	89	874
16	586	243	9	1186	118	418
26	638	303	10	1818	58	978
22	326	119	17	380	33	222
38	645	213	25	929	74	1130
51	556	215	12	1327	77	687
28	761	254	10	1435	136	923
29	160	53	11	199	29	204
27	830	269	10	941	72	1275

Endogenous BCL6	Endogenous BDNF	Endogenous BIRC3	Endogenous BK VP1	Endogenous BK large T Ag	Endogenous BLK	Endogenous BLNK
227	33	155	14	4	7	195
79	11	28	9	5	9	228
513	12	84	13	10	18	481
155	9	65	13	1	6	133
435	40	101	9	7	15	282
82	4	34	9	2	9	151
29	8	25	6	5	9	117
567	22	87	17	4	14	332
185	15	68	18	10	18	245
429	44	97	7	9	21	433
396	6	68	9	2	11	95
248	19	72	12	6	14	317
241	12	58	9	2	11	183
683	24	94	9	11	24	424
591	25	184	12	9	77	577
44	6	26	7	5	13	115
84	8	65	13	6	10	71
196	12	87	12	4	13	177
158	7	30	4	1	10	200
200	11	58	10	7	8	212
104	16	45	17	4	12	122
291	14	122	15	5	12	224
267	16	154	21	4	31	155
324	18	55	19	5	15	202
172	9	13	12	8	6	106
214	5	83	7	5	18	162

Endogenous BMP2	Endogenous BMP4	Endogenous BMP6	Endogenous BMP7	Endogenous BMPER	Endogenous BMPR1A	Endogenous BMPR1B
143	101	61	94	14	372	102
98	277	65	140	21	557	177
141	222	142	230	16	877	218
36	98	28	64	21	277	86
93	262	67	116	15	826	138
81	201	60	111	23	330	139
69	171	65	141	15	247	135
140	203	323	161	25	793	180
52	205	205	137	22	542	290
195	465	1025	287	36	1358	391
44	83	47	48	18	292	71
131	309	292	120	19	715	153
79	152	34	139	22	478	157
258	544	308	543	21	1405	390
318	166	205	114	37	809	264
34	141	53	73	22	297	88
21	84	52	13	22	185	42
164	153	46	63	33	410	87
99	104	57	121	11	359	137
39	97	124	56	15	505	130
41	60	26	45	11	170	88
79	122	93	98	9	420	91
90	176	89	65	31	394	124
111	214	46	205	17	548	177
31	89	39	17	38	131	31
100	125	43	79	31	387	84

Endogenous BRWD1	Endogenous BST2	Endogenous BTG2	Endogenous BTK	Endogenous BTLA	Endogenous C1QA	Endogenous C1QB
278	492	455	49	31	131	296
568	783	680	31	12	169	161
759	1216	548	51	20	540	933
222	438	452	31	7	148	251
549	983	554	53	21	568	1061
345	346	1102	23	13	79	64
256	418	273	22	8	59	65
549	948	903	44	12	776	1352
423	707	461	43	21	390	685
833	1320	799	31	15	628	912
149	282	255	20	8	126	186
444	841	584	58	35	539	628
419	483	484	21	10	406	616
1038	1536	1082	53	27	777	1140
951	1194	938	68	72	203	328
280	334	336	21	10	99	95
81	143	213	36	11	114	166
316	808	444	39	22	651	775
367	290	344	28	11	380	720
266	321	428	38	16	372	432
139	113	187	25	11	106	172
335	556	522	29	15	430	678
350	527	416	61	36	529	851
443	881	504	34	13	346	395
84	142	101	58	13	59	101
288	845	610	48	24	917	1223

Endogenous C1S	Endogenous C3	Endogenous C3AR1	Endogenous C5	Endogenous C5AR1	Endogenous C9	Endogenous CALHM6
1038	1908	96	55	86	153	59
439	119	92	109	87	29	43
1726	4469	136	110	223	231	86
501	235	52	40	122	150	18
1990	1887	141	94	117	135	67
212	64	54	49	58	17	16
135	30	19	68	35	19	11
1583	436	166	120	177	110	85
669	176	91	45	105	79	30
1959	193	151	149	135	142	70
411	453	35	14	71	67	19
1706	1603	125	131	108	46	100
978	546	104	102	111	179	34
2505	270	213	205	245	167	113
873	578	96	87	336	267	54
136	32	36	55	46	23	21
274	189	36	10	30	56	37
1425	1419	126	75	127	59	87
873	968	78	55	106	68	55
516	195	77	55	94	57	33
220	232	64	30	53	50	15
1067	630	103	40	84	94	31
1045	325	122	52	150	60	65
814	358	86	80	133	129	35
131	124	37	19	58	27	21
1494	972	124	56	115	94	50

Endogenous CARD16	Endogenous CARD8	Endogenous CASP1	Endogenous CASP3	Endogenous CASP4	Endogenous CASP8	Endogenous CAV1
39	188	114	142	352	159	56
30	155	90	193	248	237	146
22	254	108	271	353	354	172
18	120	40	83	125	146	38
34	294	96	208	376	401	151
11	100	47	101	101	145	68
15	96	35	94	97	139	47
23	302	115	383	435	478	228
29	151	68	165	224	278	97
33	288	135	310	471	611	231
13	67	35	94	159	133	47
30	252	137	242	365	442	161
22	208	70	156	240	335	117
37	551	92	393	429	647	301
40	609	160	436	674	677	217
31	103	46	74	115	162	66
25	50	39	46	91	101	33
50	205	132	190	301	326	91
15	120	51	161	261	301	111
23	143	45	152	198	238	33
15	84	29	75	100	96	44
38	164	71	159	316	249	83
19	109	70	182	234	339	128
29	103	68	178	168	268	144
13	45	61	50	92	73	57
26	204	95	171	253	251	59

Endogenous CCL13	Endogenous CCL15	Endogenous CCL18	Endogenous CCL19	Endogenous CCL2	Endogenous CCL20	Endogenous CCL21
36	201	47	97	489	49	241
26	281	10	27	932	18	125
22	518	12	30	355	25	108
17	285	15	16	306	10	62
36	442	17	264	903	76	958
19	192	11	19	138	11	66
18	160	17	15	143	19	54
49	351	18	47	2266	54	273
40	191	27	25	683	46	105
50	468	24	35	4037	56	143
11	126	13	65	339	10	154
56	273	34	323	1822	24	1414
33	591	20	38	699	16	168
33	770	24	175	611	65	876
33	147	34	121	946	103	360
30	145	24	14	205	28	116
33	93	27	68	673	17	120
35	277	34	218	1577	79	1584
15	282	7	27	554	15	207
23	648	24	61	555	14	108
21	115	28	35	274	29	38
32	300	21	152	2217	27	355
42	139	58	1287	1138	48	2200
40	321	36	61	333	23	295
57	47	34	33	152	38	36
38	367	14	83	1674	47	483

Endogenous CCL22	Endogenous CCL3/L1	Endogenous CCL4	Endogenous CCL5	Endogenous CCR1	Endogenous CCR10	Endogenous CCR2
27	65	27	48	67	57	55
11	45	2	83	89	29	39
19	24	6	69	79	32	63
9	48	6	17	51	14	19
19	34	5	60	97	39	84
13	27	4	22	79	16	14
4	16	1	22	53	15	14
17	48	10	82	138	40	56
15	36	9	32	62	32	34
15	25	7	52	100	43	52
12	15	1	19	42	21	27
12	21	6	125	133	32	124
10	12	8	33	97	37	26
27	35	10	80	94	54	88
26	48	10	88	110	32	60
13	34	5	46	52	19	21
8	19	7	44	50	32	22
4	52	7	176	175	27	51
3	17	3	48	72	12	25
10	34	6	45	57	23	29
10	42	6	19	34	20	30
11	30	7	75	158	23	48
16	42	13	105	121	45	36
13	27	8	37	71	32	46
14	38	5	10	46	23	40
8	36	6	120	138	25	55

Endogenous CCR3	Endogenous CCR4	Endogenous CCR5	Endogenous CCR6	Endogenous CCR7	Endogenous CD14	Endogenous CD160
40	20	62	46	25	324	41
17	5	29	30	20	449	11
15	8	70	34	13	568	17
10	7	36	18	12	264	8
17	16	68	38	18	1120	11
12	8	27	11	11	129	10
10	6	29	18	20	169	6
18	12	54	37	12	994	21
13	6	39	26	23	834	14
22	12	60	42	19	1552	18
8	7	32	7	16	151	10
11	11	100	46	19	956	19
18	8	27	27	13	651	15
15	12	62	45	22	815	18
31	19	48	60	26	482	26
13	5	26	23	22	239	13
16	7	44	18	9	295	10
10	15	69	44	33	893	21
8	9	31	19	12	418	7
14	10	22	22	16	353	6
26	11	30	22	18	174	18
25	18	60	19	14	936	12
23	27	81	32	32	657	16
18	16	51	27	34	480	20
16	8	76	15	45	109	25
11	23	56	41	15	1417	14

Endogenous CD163	Endogenous CD19	Endogenous CD1D	Endogenous CD2	Endogenous CD207	Endogenous CD209	Endogenous CD22
198	22	26	23	11	74	53
108	9	32	21	21	77	16
2165	6	56	24	20	44	32
296	5	16	18	22	60	28
1489	11	52	23	19	188	28
100	6	12	13	11	35	12
43	3	10	18	6	28	23
1492	12	44	20	23	112	46
335	11	25	20	18	207	46
631	9	54	28	23	165	36
419	8	20	16	12	40	23
473	11	39	41	18	267	45
652	5	43	21	19	130	29
2672	19	62	16	39	157	61
2354	38	64	36	22	183	141
70	5	17	20	20	47	22
99	4	12	20	8	75	10
609	8	33	40	3	362	32
1171	4	21	10	9	57	7
286	7	21	11	10	85	20
236	7	14	23	13	54	20
448	4	20	31	18	119	28
1435	17	52	62	10	247	86
984	11	25	20	15	90	25
127	5	21	46	10	68	15
909	13	35	34	14	377	50

Endogenous CD24	Endogenous CD244	Endogenous CD247	Endogenous CD27	Endogenous CD274	Endogenous CD276	Endogenous CD28
13028	28	57	25	39	173	34
10144	18	27	14	21	208	31
43523	15	35	23	44	353	17
5901	8	12	7	18	107	12
29926	21	33	12	40	502	37
5526	10	12	8	15	108	10
3875	9	20	9	14	98	10
42330	16	29	10	47	621	18
18345	21	30	10	15	302	34
55155	17	32	12	50	608	36
11181	4	11	7	15	147	6
28079	27	32	30	25	497	51
10898	17	16	8	21	204	35
43118	26	38	20	46	536	40
24311	25	66	54	44	460	36
3478	6	19	8	19	100	17
4377	18	13	15	14	87	13
11434	20	43	19	17	267	81
15361	9	19	3	14	236	15
10597	11	26	6	27	341	14
5036	24	13	8	16	99	9
12688	19	29	17	20	216	36
11512	22	45	32	33	286	75
10738	21	27	20	28	222	15
4078	23	9	13	18	53	9
7035	12	42	38	14	242	36

Endogenous CD34	Endogenous CD38	Endogenous CD3D	Endogenous CD3E	Endogenous CD3G	Endogenous CD4	Endogenous CD40
85	25	49	40	30	88	97
228	41	28	26	8	143	162
325	24	28	34	9	152	196
109	20	13	15	7	47	96
166	57	20	36	12	199	128
112	24	16	13	8	53	96
146	33	7	13	8	56	75
321	46	15	25	16	200	215
139	57	20	18	12	85	146
435	68	21	29	15	134	244
57	18	11	18	11	34	84
179	60	32	61	22	272	181
227	38	19	24	9	125	116
458	43	33	40	23	280	250
284	77	48	46	28	160	404
143	31	13	17	12	39	90
41	24	11	16	12	54	46
204	49	47	56	29	241	130
114	40	13	23	9	149	125
146	23	15	21	13	79	120
64	16	8	19	9	30	82
106	42	34	49	21	131	159
183	68	50	71	33	143	123
276	39	13	27	19	110	146
32	64	10	19	11	18	76
145	39	49	50	24	261	189

Endogenous CD40LG	Endogenous CD44	Endogenous CD45R0	Endogenous CD45RA	Endogenous CD45RB	Endogenous CD46	Endogenous CD47
17	195	91	4	22	1394	456
12	559	124	19	36	3184	543
23	451	180	33	55	4762	900
15	143	47	11	17	1518	314
12	663	167	24	55	3314	887
4	390	63	15	12	1606	324
3	347	53	4	12	1639	357
13	1843	153	25	64	3762	1028
14	463	81	25	23	2775	546
11	1144	107	25	40	6184	1183
9	542	54	10	15	952	254
36	1150	264	29	63	2940	642
7	351	86	10	23	3566	531
13	728	178	23	50	6644	1077
25	1628	159	70	86	4812	1068
9	391	72	17	26	1461	237
16	272	71	12	19	608	153
27	913	210	27	67	2106	571
15	339	116	9	23	2708	491
4	550	67	14	31	1656	416
11	167	69	14	16	775	204
18	528	127	22	38	2248	491
24	923	197	57	44	1556	500
12	310	115	20	24	2886	600
10	215	71	24	18	419	146
26	565	214	41	81	1926	457

Endogenous CD48	Endogenous CD5	Endogenous CD55	Endogenous CD58	Endogenous CD59	Endogenous CD6	Endogenous CD68
50	18	166	203	7923	28	253
41	41	265	340	8085	15	565
59	54	470	770	19079	16	671
16	20	118	199	5850	8	204
67	40	308	535	12394	30	516
30	27	110	180	4631	15	270
16	20	160	153	4142	12	226
64	36	433	536	20976	17	680
28	27	311	280	8674	18	241
48	45	459	862	22246	19	654
34	12	190	162	6661	5	239
129	43	341	375	10428	47	508
20	20	241	412	8524	14	347
73	64	514	985	18810	25	629
136	36	542	840	22351	40	647
16	34	142	150	3936	21	392
40	24	63	94	3109	18	110
127	55	269	351	5622	43	517
37	12	262	423	8611	13	261
58	19	195	218	10616	12	525
18	20	93	137	3631	18	156
69	31	167	309	8361	26	523
102	72	215	283	9157	72	465
39	39	220	354	8461	15	500
36	45	102	97	2486	29	129
142	37	212	325	6039	26	650

Endogenous CD69	Endogenous CD7	Endogenous CD70	Endogenous CD72	Endogenous CD74	Endogenous CD79A	Endogenous CD80
52	13	43	44	9527	29	26
36	8	34	20	8186	20	1
37	36	22	26	25811	18	4
14	6	26	18	8665	13	1
51	14	34	36	21965	16	3
17	10	20	18	5953	8	1
8	5	16	11	4169	8	1
42	23	26	41	13412	26	1
23	9	37	27	8712	17	8
32	19	22	52	11502	25	1
18	13	15	15	3848	11	6
59	34	29	54	24832	31	10
15	13	21	24	15513	11	1
52	13	29	43	21643	34	1
233	33	47	33	19486	60	29
14	7	23	15	5378	15	2
14	5	31	32	6788	10	9
60	19	50	65	21545	26	22
33	7	12	19	9452	8	3
37	25	17	29	5737	15	2
18	7	23	24	2390	23	4
40	22	24	33	15168	19	7
117	35	53	39	12298	21	9
47	10	45	35	14144	23	12
21	17	76	19	1175	22	9
147	18	24	22	16433	17	11

Endogenous CD81	Endogenous CD82	Endogenous CD83	Endogenous CD84	Endogenous CD86	Endogenous CD8A	Endogenous CD8B
4065	102	205	74	28	47	53
6084	137	486	48	25	32	62
8693	303	784	91	37	60	22
2288	121	164	13	7	12	7
7499	202	423	101	41	40	21
2126	74	293	34	16	14	26
2751	76	312	14	6	8	70
8989	308	331	126	37	25	14
4525	286	483	55	18	27	18
13231	750	434	96	35	25	27
1532	73	154	19	7	17	10
6664	208	239	97	34	37	31
6005	198	431	56	18	19	14
12851	552	907	137	37	52	17
4803	318	364	136	36	50	18
2030	68	257	31	8	13	38
1067	85	304	20	13	34	20
3491	134	255	144	50	91	29
5833	151	205	64	16	24	25
2468	208	270	48	13	13	9
1397	73	98	31	17	14	12
4610	115	194	92	21	46	39
4812	208	147	86	30	89	22
4540	197	248	52	14	34	21
836	41	105	16	7	11	4
4329	151	573	128	31	65	14

Endogenous CD96	Endogenous CDH13	Endogenous CDH5	Endogenous CDKN1A	Endogenous CEACAM3	Endogenous CETP	Endogenous CFB
30	54	142	213	24	54	1887
28	47	182	355	11	96	356
31	48	154	1021	19	70	5522
13	28	67	496	12	35	890
42	49	182	735	20	85	3037
26	47	85	274	5	26	278
21	46	104	94	8	52	184
26	104	207	1067	14	72	1326
28	48	143	260	10	44	1021
26	100	403	989	21	33	1003
10	26	52	575	8	22	1196
46	44	212	338	17	135	4565
18	25	113	568	11	26	1675
30	115	318	1774	17	78	1718
75	133	352	1299	15	83	1419
19	27	90	135	16	45	158
21	31	53	179	6	17	879
70	68	169	508	16	39	2484
24	20	108	475	3	32	1714
18	23	84	429	16	50	462
17	21	51	201	9	23	283
31	43	91	450	13	38	1852
74	74	129	1039	9	61	1003
36	49	153	817	19	58	1142
37	24	49	240	15	17	143
59	31	110	484	9	29	1722

Endogenous CFH	Endogenous CFI	Endogenous CFLAR	Endogenous CGAS	Endogenous CH25H	Endogenous CHCHD10	Endogenous CHUK
468	2111	436	18	37	2900	319
611	2499	904	19	28	5040	539
894	5282	1802	38	20	11585	1024
393	2283	395	27	24	5170	309
1115	4297	908	35	17	9866	751
235	1284	502	16	31	4022	287
341	1071	444	8	15	2738	246
843	4349	1467	42	37	8700	723
668	2022	605	25	54	4970	523
1037	3668	1562	51	33	13932	1095
288	889	295	9	22	1944	271
1004	3887	767	50	40	4932	518
739	4671	825	31	34	8987	655
1754	5389	2377	53	16	14978	1387
920	4554	1332	65	17	4745	785
333	1277	463	20	31	3539	292
213	737	147	14	27	1546	139
740	2955	553	48	28	3887	341
772	3368	758	15	8	5268	364
227	2627	502	24	38	6537	539
171	639	228	23	36	1903	171
709	3104	505	27	50	5180	400
872	1510	611	40	47	2894	273
747	3341	854	30	49	7314	547
161	286	140	16	49	763	96
1416	2861	565	53	39	3939	411

Endogenous CIITA	Endogenous CITED4	Endogenous CLEC4C	Endogenous CMKLR1	Endogenous CMV UL83	Endogenous COL13A1	Endogenous COL1A1
176	102	8	93	10	19	252
232	98	12	115	12	31	259
248	114	16	200	14	33	431
140	51	12	92	10	30	156
202	146	16	269	9	50	895
118	84	8	65	7	19	128
146	36	7	61	2	9	79
279	215	10	316	11	50	2662
160	144	12	155	5	22	235
239	279	9	271	12	60	1729
114	74	2	65	5	11	404
452	152	18	277	10	26	1510
252	107	9	172	13	38	414
421	159	17	660	14	104	849
684	112	20	324	16	43	749
189	59	8	66	6	17	71
163	86	8	46	9	12	204
407	121	13	335	12	35	669
142	113	5	137	6	17	478
212	71	10	161	15	29	866
52	61	10	58	6	24	100
280	118	13	133	8	31	395
256	129	10	270	15	47	997
317	104	13	271	8	35	289
103	69	6	39	9	9	166
250	94	5	227	4	19	662

Endogenous COL3A1	Endogenous COL4A1	Endogenous COL4A3	Endogenous COL4A4	Endogenous COL4A5	Endogenous CPA3	Endogenous CR1
2122	2305	182	582	109	54	182
1772	1393	387	923	140	49	255
6118	6569	578	2061	236	39	182
801	1115	182	489	84	7	72
6294	3756	536	1684	205	33	228
709	795	176	591	103	17	108
505	619	176	471	96	12	233
12489	7614	493	1563	223	23	233
1664	2439	329	907	225	27	135
6517	6855	475	2321	355	37	156
1619	2238	221	465	76	13	64
8350	6949	316	1211	147	155	430
1854	2212	310	1151	130	27	180
4852	7179	868	2799	387	100	511
3434	7481	853	2683	254	108	198
512	520	229	565	98	14	95
709	929	80	259	42	12	43
2336	2370	294	631	98	151	471
2339	2696	366	884	143	45	195
2316	2634	264	670	113	24	95
377	693	128	332	67	19	77
2565	2374	247	871	95	26	126
3029	3162	279	834	95	62	198
1246	1660	464	1292	213	24	333
893	667	44	207	23	16	44
2864	1927	252	814	92	69	269

Endogenous CRHBP	Endogenous CRIP2	Endogenous CRP	Endogenous CSF1	Endogenous CSF2	Endogenous CSF2RB	Endogenous CSF3
215	189	11	90	11	29	23
524	376	15	134	9	32	20
888	594	42	170	10	25	16
267	275	13	84	5	20	7
238	598	10	170	14	25	18
324	247	14	76	3	18	15
400	203	12	71	8	22	10
484	738	13	231	13	32	23
462	499	18	109	13	36	21
445	1380	11	302	8	43	16
111	271	12	30	5	10	4
182	564	10	172	7	65	22
370	489	14	143	13	28	12
714	1269	6	339	9	26	16
516	797	19	230	13	42	21
167	243	19	71	2	20	16
28	133	12	27	18	22	11
447	381	16	161	7	51	19
263	590	14	81	5	24	7
147	301	10	54	6	22	11
65	145	13	39	11	19	17
154	386	9	135	19	38	28
201	561	31	219	16	42	21
456	564	28	225	15	37	13
35	119	43	31	20	45	26
285	314	14	122	5	47	6

Endogenous CSF3R	Endogenous CTLA4	Endogenous CTNNB1	Endogenous CTSL	Endogenous CTSS	Endogenous CTSW	Endogenous CX3CL1
25	34	1656	1128	204	33	148
29	10	2843	1815	303	26	286
40	13	4720	3598	545	23	286
24	8	1329	840	140	7	104
50	18	3375	1405	677	22	323
16	9	1509	804	95	10	172
15	4	1220	736	92	10	156
55	15	3882	1544	634	17	342
26	9	2576	590	268	19	343
34	11	5230	2082	571	22	540
42	6	989	752	187	12	72
44	11	2987	867	950	40	285
21	6	3057	1953	331	14	212
44	12	5306	2232	709	31	536
80	30	3746	3949	771	33	311
15	4	1374	741	68	12	158
21	13	696	321	126	15	80
48	26	2100	796	867	59	211
36	9	2267	960	314	18	112
37	11	1660	1013	267	12	104
32	8	700	465	117	10	61
19	19	2167	1268	433	25	199
43	20	1484	848	474	54	154
24	10	2794	1368	224	16	255
44	9	530	313	90	11	42
35	16	2139	981	827	50	190

Endogenous CX3CR1	Endogenous CXCL1/2	Endogenous CXCL10	Endogenous CXCL11	Endogenous CXCL12	Endogenous CXCL13	Endogenous CXCL14
111	620	53	54	229	16	8230
89	290	22	14	954	11	14229
73	462	19	12	661	18	7433
28	166	10	13	300	17	5006
88	594	64	13	1289	10	6637
43	95	6	11	822	11	7178
30	79	12	6	424	11	5668
103	903	53	46	649	22	7482
38	189	47	17	1078	10	4026
61	430	32	19	1635	16	10673
8	278	6	12	68	9	2304
188	586	15	23	2119	16	9528
54	199	15	13	630	15	9171
47	417	35	21	1886	31	11109
44	1938	45	30	235	13	2826
46	84	15	10	509	13	6443
52	151	17	17	446	12	3501
81	1201	14	14	744	14	9501
27	270	15	12	442	5	5955
56	329	22	18	319	13	9826
13	248	13	13	103	19	1687
83	1022	23	15	646	14	11262
64	603	13	13	1622	20	2027
18	146	21	20	617	17	5607
3	173	13	6	42	18	828
95	784	10	14	1076	12	7243

Endogenous CXCL16	Endogenous CXCL2	Endogenous CXCL5	Endogenous CXCL8	Endogenous CXCL9	Endogenous CXCR3	Endogenous CXCR4
245	458	29	67	60	21	65
152	315	6	31	39	16	140
334	202	7	20	47	11	205
131	86	8	9	20	6	59
268	231	5	48	43	19	238
94	99	3	5	20	12	97
74	57	4	10	52	10	83
544	729	19	109	59	8	162
213	89	10	27	35	11	131
487	297	7	47	42	16	128
96	219	7	33	30	7	59
529	239	4	38	55	40	251
218	177	7	9	25	9	63
393	348	9	19	37	17	378
389	1934	14	184	31	21	936
80	50	5	11	42	10	49
106	107	5	14	61	15	53
326	536	8	114	69	30	175
217	166	4	11	24	6	111
181	334	3	35	52	11	85
83	174	5	33	21	15	43
282	553	8	46	74	23	107
307	349	18	70	40	26	429
189	142	11	20	95	10	122
62	144	6	90	50	11	76
256	246	5	57	17	17	266

Endogenous CXCR5	Endogenous CXCR6	Endogenous DCAF12	Endogenous DDX50	Endogenous DEFB1	Endogenous DNMT1	Endogenous DNMT3A
28	54	165	306	2016	115	180
12	45	209	356	4089	170	348
18	42	383	618	6318	340	520
6	17	133	191	3207	112	131
10	40	312	425	2731	355	400
8	19	140	172	2710	91	206
8	17	98	176	3913	87	154
10	22	413	506	906	437	375
15	31	273	355	1341	201	276
16	21	472	737	8837	586	643
15	18	105	136	385	106	112
16	41	322	446	2687	335	425
12	17	255	320	8335	251	314
14	48	580	729	8083	526	773
40	53	311	546	3625	389	475
11	20	121	204	2671	99	181
20	29	67	107	675	75	88
9	42	199	280	1357	216	250
6	21	194	286	3611	188	239
6	28	182	244	739	138	176
15	24	88	115	444	118	96
5	42	204	317	2339	203	220
38	55	164	256	1970	181	197
8	44	253	364	9037	231	275
15	59	73	110	226	76	92
22	38	248	279	1675	256	223

Endogenous DUSP2	Endogenous EBI3	Endogenous EBV LMP2	Endogenous ECSCR	Endogenous EDA	Endogenous EEF1A1	Endogenous EGFR
5	16	4	133	21	45307	248
12	12	5	176	46	97818	382
12	26	8	231	55	102872	697
5	8	5	98	24	37132	216
10	37	10	257	63	75476	731
28	9	10	92	48	49250	274
8	10	3	105	28	38631	207
18	49	4	345	68	115035	733
10	7	2	151	41	59018	405
21	21	7	339	77	113450	1051
8	11	7	95	24	32075	195
14	16	10	233	48	87066	449
13	21	9	135	39	67921	543
11	39	5	244	74	149770	1132
29	18	3	524	57	62678	687
7	19	8	98	44	54298	220
4	7	3	87	17	18106	96
13	22	4	223	35	56229	357
11	16	2	119	29	53288	412
14	17	9	116	45	49490	316
5	18	5	67	15	20710	172
13	22	8	172	22	53173	346
24	27	9	207	52	40827	281
30	31	8	189	45	61224	506
6	10	9	39	35	10660	98
14	16	1	125	33	44309	293

Endogenous EGR1	Endogenous EHD3	Endogenous EMP3	Endogenous ENG	Endogenous EOMES	Endogenous EPAS1	Endogenous EPO
134	105	123	1302	10	907	373
646	537	194	2070	15	1407	11
95	571	352	2597	8	2063	8
180	209	117	867	15	582	104
234	365	470	2123	19	1878	7
1111	395	96	1049	10	843	15
218	549	102	1157	10	799	5
479	449	562	2804	13	2054	12
114	327	282	1939	14	1252	357
490	336	802	5324	11	2672	24
186	106	169	520	5	366	9
556	276	361	2434	19	1517	126
169	376	233	2078	19	1666	415
328	649	603	3535	14	3816	10
1223	289	464	3279	16	2069	23
278	253	113	1016	16	849	12
81	41	108	573	9	331	27
423	341	353	1845	21	1094	7
69	201	243	1156	5	1296	42
160	244	174	818	7	849	21
131	53	150	488	22	446	12
431	218	210	1637	20	928	78
293	175	396	1752	20	1366	5
188	392	209	1936	13	2286	11
75	44	108	314	20	173	17
416	215	360	2113	15	1138	15

Endogenous ERG	Endogenous ERRFI1	Endogenous EVA1C	Endogenous EZH2	Endogenous F3	Endogenous FABP1	Endogenous FADD
76	563	236	24	126	404	153
138	824	71	36	205	3494	292
199	5403	348	39	115	11512	525
78	168	59	25	65	2340	187
163	2508	190	50	140	1084	481
75	3137	48	15	117	2082	228
83	2181	54	24	187	2235	154
170	1439	259	90	200	1766	511
124	383	102	19	78	2105	305
314	1333	354	64	190	1799	618
56	762	83	20	66	297	178
153	355	118	66	63	81	351
94	2105	134	28	126	9718	421
282	2548	424	42	319	7322	765
308	4379	271	76	247	1977	353
76	2499	63	20	66	2274	225
25	85	56	21	21	98	117
115	242	171	44	112	207	248
100	1344	115	22	139	831	249
57	445	47	33	71	4302	343
31	270	47	32	34	263	138
80	359	203	38	79	1476	252
129	560	192	54	126	115	206
151	1387	179	47	194	4484	322
32	367	34	39	55	226	89
114	223	78	38	101	5254	272

Endogenous FAM30A	Endogenous FAS	Endogenous FASLG	Endogenous FCAR	Endogenous FCER1A	Endogenous FCER1G	Endogenous FCGR1A
54	165	25	38	17	136	90
28	287	11	10	80	98	54
20	278	11	5	14	347	147
16	134	5	13	8	107	74
24	351	6	30	60	389	106
17	92	3	7	27	46	25
4	94	7	6	19	53	16
30	496	12	19	14	501	220
28	221	5	11	11	263	77
38	453	9	21	23	348	105
13	146	3	11	3	166	48
40	319	9	21	112	433	215
22	175	9	14	15	228	81
32	357	10	15	40	340	231
74	653	13	48	13	273	114
18	146	2	8	14	48	27
15	62	9	3	33	116	62
17	354	13	20	79	376	202
15	190	5	8	5	227	72
17	269	9	10	12	152	50
25	95	5	11	6	111	41
33	199	9	14	22	252	174
31	332	10	25	19	312	118
35	145	10	14	23	219	121
23	71	6	9	8	48	17
34	226	14	20	100	437	99

Endogenous FCGR2A	Endogenous FCGR2B	Endogenous FCGR3A/B	Endogenous FCRL2	Endogenous FGD2	Endogenous FGFBP2	Endogenous FJX1
275	87	307	15	57	11	136
139	64	71	10	66	7	80
270	66	393	16	189	11	105
235	22	112	2	34	8	56
241	79	150	16	193	12	163
83	47	23	6	53	5	33
70	36	24	1	21	9	32
462	38	417	14	237	10	282
262	53	94	15	54	7	101
255	99	174	9	169	20	411
76	6	79	5	68	5	75
313	154	255	23	126	21	438
315	76	120	8	106	5	113
293	177	181	7	344	9	176
462	118	910	63	420	15	180
66	38	34	10	31	12	58
74	38	59	4	18	9	85
381	200	332	14	127	9	266
270	40	233	2	110	7	108
171	34	92	5	79	10	105
106	26	61	6	30	8	54
391	84	303	15	89	10	319
366	80	103	18	182	5	98
282	53	151	8	103	19	73
48	27	70	12	34	6	46
407	158	494	15	92	7	228

Endogenous FKBP1A	Endogenous FLT3	Endogenous FN1	Endogenous FOS	Endogenous FOSL1	Endogenous FOXO1	Endogenous FOXP3
553	56	458	787	49	212	26
616	31	513	1571	63	420	13
1212	39	591	571	53	725	11
642	18	319	1023	22	167	13
1347	30	923	900	46	497	12
522	22	314	3287	25	228	7
342	15	192	772	16	264	9
1876	69	3803	1813	103	589	10
1005	21	640	338	29	311	16
1846	27	1087	1031	104	758	13
658	27	447	561	52	235	7
1154	62	1657	1513	70	323	19
1179	93	602	973	49	470	11
1805	124	1220	1369	104	923	18
1668	115	1195	8569	85	847	15
480	27	217	661	18	230	5
493	19	156	252	30	118	8
969	17	432	1480	47	240	12
820	17	962	355	25	303	6
1061	28	710	823	64	281	14
449	22	142	414	46	167	13
902	17	522	1281	43	233	14
1099	57	887	673	72	356	13
1081	131	468	347	55	472	12
304	35	423	238	61	128	10
837	21	591	1373	40	260	11

Endogenous FPR1	Endogenous FYN	Endogenous GAPDH	Endogenous GATA3	Endogenous GBP1	Endogenous GBP2	Endogenous GBP4
118	220	4488	333	332	480	180
30	348	9433	916	291	177	302
210	620	15572	1291	531	1042	474
74	194	7853	379	143	139	94
116	396	13898	662	293	484	341
14	200	6153	509	140	76	164
17	222	4694	518	122	88	172
227	466	15367	626	341	597	393
67	304	6579	673	222	261	307
139	431	19491	1403	437	676	737
85	173	6579	223	131	344	59
125	357	7798	658	411	441	342
92	249	13362	523	249	239	238
116	839	20020	1414	558	327	474
288	568	16240	1071	312	896	259
12	190	5754	466	141	87	280
37	86	2818	159	114	133	95
143	317	6609	418	249	456	234
108	257	9535	600	163	233	139
55	272	15946	507	167	237	218
60	78	3648	219	99	132	66
150	200	7952	313	407	534	229
197	323	7509	388	324	421	188
100	365	11939	924	234	228	253
37	57	3323	84	67	167	51
127	306	7908	472	217	351	218

Endogenous GBP5	Endogenous GDF15	Endogenous GEMIN7	Endogenous GIMAP5	Endogenous GNG11	Endogenous GNLY	Endogenous GZMA
7	289	172	168	405	42	70
13	837	135	280	889	32	58
26	877	259	691	864	22	57
6	671	98	157	351	22	21
16	974	203	545	577	34	45
6	262	87	231	513	19	23
6	151	77	220	427	12	17
26	1185	238	588	1274	19	34
15	343	197	361	746	42	36
20	1667	340	726	1384	19	42
10	1085	72	174	298	26	16
37	1333	165	384	777	55	104
12	466	156	309	692	23	28
22	1638	360	853	1208	41	57
27	3882	217	837	1726	100	87
13	231	80	228	371	25	27
12	583	29	73	184	31	36
43	1221	120	296	694	53	127
17	767	136	320	527	18	37
15	1135	81	274	464	25	21
7	410	48	118	236	22	31
22	693	154	227	531	16	80
31	1822	100	435	517	42	82
10	516	186	427	671	26	34
5	334	59	55	187	65	37
29	1030	126	352	618	22	70

Endogenous GZMB	Endogenous GZMH	Endogenous GZMK	Endogenous HAVCR1	Endogenous HAVCR2	Endogenous HDAC3	Endogenous HDAC6
26	31	21	371	824	321	173
13	15	27	150	322	585	824
22	20	43	392	503	822	802
6	12	10	229	341	271	393
18	6	26	1375	823	795	485
8	9	24	130	173	360	631
6	7	6	32	110	315	458
20	18	19	618	301	789	572
13	11	26	318	135	500	429
12	12	27	1720	924	1083	839
9	7	14	84	143	241	208
22	18	32	1345	649	623	333
8	14	23	670	1017	599	845
18	14	33	1186	1136	1176	1292
41	23	29	1463	427	866	616
7	7	29	37	153	330	746
9	5	23	166	138	149	122
19	29	46	966	413	408	318
13	12	33	947	579	392	371
17	8	18	439	304	600	636
10	12	17	389	159	185	126
11	10	39	752	471	410	354
7	14	57	342	159	368	260
12	12	32	291	651	496	698
8	11	33	195	33	96	72
13	12	48	297	361	490	701

Endogenous HDC	Endogenous HEG1	Endogenous HFE	Endogenous HIF1A	Endogenous HK2	Endogenous HLA-A	Endogenous HLA-B
31	445	107	3804	90	10796	1912
18	973	112	1310	42	9796	1729
13	695	119	4636	59	12443	3347
6	304	67	1906	21	6196	1132
21	707	102	7185	47	22175	6460
13	390	57	666	11	4701	969
8	515	50	675	15	4325	1353
19	948	107	8133	64	11217	4216
18	443	100	3464	37	7422	1823
18	1196	139	8804	57	16169	2836
14	146	39	2045	13	4354	1489
41	868	107	6178	47	13072	4317
13	561	95	4569	21	8370	3172
29	1191	145	4533	87	18717	3525
68	767	96	7882	97	19146	3371
7	384	75	563	19	4598	1060
8	190	34	2252	25	2859	1096
65	1092	88	3957	21	9430	3490
6	397	63	3026	35	11741	2057
16	373	88	2526	27	4188	1475
10	153	33	1822	27	2346	803
12	578	63	4585	48	8162	2852
34	571	92	2988	70	7014	2750
12	832	98	2551	61	6661	2393
8	73	48	908	80	1437	687
17	649	72	4710	13	9216	2407

Endogenous HLA-C	Endogenous HLA-DMA	Endogenous HLA-DMB	Endogenous HLA-DPA1	Endogenous HLA-DPB1	Endogenous HLA-DQA1	Endogenous HLA-DQB1
3432	614	246	626	403	188	321
4233	462	181	1158	946	836	133
3703	1285	341	1740	934	13	1031
4733	508	111	515	297	250	80
4674	1021	286	1659	1336	705	112
3120	277	112	619	407	484	82
1466	291	93	507	425	221	231
8109	847	305	996	994	14	919
5578	378	268	543	507	416	86
4883	520	324	862	721	268	277
3642	183	43	180	193	72	42
3636	1102	369	2318	1991	19	1038
2771	626	220	620	703	252	613
4574	1000	280	1624	1540	545	267
4156	985	255	1655	968	14	445
2412	219	90	603	439	15	190
2184	309	95	539	370	17	211
3600	1129	359	2206	1708	641	472
2233	375	127	561	451	442	165
2729	383	105	506	571	16	266
1696	162	61	175	146	17	74
6552	953	316	1283	962	20	501
4800	396	219	885	1225	37	336
3620	742	276	848	762	28	243
766	72	30	115	71	38	49
3052	1035	302	1622	980	525	238

Endogenous HLA-DRA	Endogenous HLA-DRB1	Endogenous HLA-DRB3	Endogenous HLA-E	Endogenous HLA-F	Endogenous HLA-G	Endogenous HMGB1
3945	4400	2660	3017	105	78	764
3295	2668	2382	2727	160	62	1193
8457	1787	4891	5363	228	80	1806
2428	2169	2239	1920	93	28	534
5942	7217	4946	5016	325	128	1033
1782	1723	1558	1568	101	33	670
1512	504	1002	1479	71	17	615
4035	697	2393	5224	241	65	1673
2286	2677	2534	2760	97	32	965
3352	1280	2546	4976	210	124	2544
851	999	937	1726	78	20	442
8344	6592	6579	3280	289	80	1049
4431	2263	2196	3648	171	66	1246
6621	5342	4380	5084	221	102	1964
4855	1511	5125	5416	331	110	1597
1445	1352	1368	1642	74	41	540
1473	1688	1841	1324	59	18	188
6517	4265	4650	3161	217	50	798
3063	1322	1944	2822	118	30	1046
1363	263	1247	2222	113	19	461
715	547	698	915	59	21	348
5045	2928	3053	2986	173	59	945
3405	1633	3486	2804	163	42	871
3770	746	2762	3575	171	48	1301
274	150	344	622	40	29	196
5946	3999	4496	2657	139	24	931

Endogenous HNF1A	Endogenous HPRT1	Endogenous HSD11B1	Endogenous HSP90AA1	Endogenous HSPA12B	Endogenous HYAL1	Endogenous HYAL2
49	396	20	3577	69	638	474
381	507	22	7680	123	1506	572
340	1317	75	10107	179	2649	1153
184	341	14	6494	108	936	455
202	730	71	7501	180	1681	1262
186	246	15	3083	83	647	500
146	272	9	3412	114	608	343
214	729	142	7874	247	1377	1491
133	405	39	4326	118	1186	793
223	911	222	8944	390	2822	2012
104	233	29	1424	87	350	303
122	604	37	4939	153	1218	871
344	530	14	5880	141	2015	896
534	1096	71	13523	467	4450	2582
241	1142	32	4778	224	877	1858
245	227	10	3054	106	695	386
22	152	14	1259	55	271	230
145	312	41	3545	164	651	700
169	549	23	3425	93	954	886
288	313	21	5491	131	970	438
44	178	17	1716	63	307	301
88	504	39	6660	116	817	701
88	466	54	1650	199	555	929
235	545	36	6842	202	1286	1364
23	145	6	1702	67	127	161
192	329	29	4394	105	1126	794

Endogenous ICAM1	Endogenous ICAM2	Endogenous ICOS	Endogenous ICOSLG	Endogenous IDO1	Endogenous IER5	Endogenous IFI27
495	114	5	108	38	40	359
231	155	13	183	57	94	773
302	144	9	357	46	101	564
311	87	6	152	24	41	291
572	150	8	174	37	107	825
76	80	3	102	28	56	470
73	109	7	123	24	51	467
626	162	2	281	27	127	1423
272	156	10	155	39	59	636
684	285	10	532	35	135	1309
235	70	7	104	27	55	283
602	158	13	141	41	103	626
383	161	9	230	25	60	539
518	330	7	445	45	195	2011
908	344	18	340	96	231	1116
94	81	2	103	24	42	435
341	61	8	60	30	27	231
705	158	17	129	35	111	933
338	109	6	131	25	52	449
246	80	9	178	27	67	482
152	58	15	47	26	37	157
727	126	11	139	43	62	478
774	178	17	170	41	110	686
391	261	8	211	57	95	1092
73	84	9	62	57	23	106
557	113	6	209	33	69	515

Endogenous IFI30	Endogenous IFI44	Endogenous IFI6	Endogenous IFIT1	Endogenous IFITM1	Endogenous IFITM2	Endogenous IFITM3
287	89	341	108	2118	3389	7884
330	181	454	417	1139	1142	2694
764	242	535	215	6677	5213	23237
271	77	212	75	1566	1343	4696
858	246	1035	339	5006	3186	16468
214	122	259	229	906	783	2171
196	88	310	231	673	511	1449
612	313	1398	383	5659	5609	16575
302	149	758	329	1897	2156	5108
1088	429	1518	641	6007	3901	16085
214	72	2415	43	2927	2381	11244
472	220	623	284	3645	2745	10916
745	149	440	266	2593	1940	7776
878	436	1706	425	4808	3727	14244
574	393	2015	179	7391	5319	20712
165	106	277	161	763	534	1910
124	46	272	78	839	762	3268
455	218	612	238	3545	2747	8705
419	113	461	172	2487	2198	7328
285	85	349	150	1951	1243	6209
199	40	134	49	695	724	1878
410	128	382	176	2504	2949	5615
373	244	652	153	3851	3549	9442
493	149	470	163	2450	2419	6184
116	29	56	27	925	914	2408
420	211	678	127	2912	2723	6436

Endogenous IFNA1	Endogenous IFNAR1	Endogenous IFNAR2	Endogenous IFNG	Endogenous IFNGR1	Endogenous IFNGR2	Endogenous IGF1
37	539	600	15	387	741	76
10	801	309	12	615	611	102
17	1153	851	19	1287	1632	39
13	370	302	23	350	526	17
14	943	824	19	929	1253	251
7	273	195	15	337	360	92
6	406	156	12	271	316	69
18	1199	1102	14	1239	1394	62
13	753	412	26	492	674	90
19	1637	717	18	1170	1527	131
6	292	416	13	305	524	60
15	1025	903	24	703	1078	70
10	792	462	20	798	999	143
24	1523	1076	15	1548	1926	69
29	1052	1665	18	1322	1020	101
11	327	118	21	290	270	36
12	190	273	13	140	313	34
11	560	834	18	590	800	142
7	571	394	8	608	682	37
6	555	487	21	415	688	48
19	225	200	25	192	244	26
11	676	650	9	603	962	67
19	461	619	24	673	607	69
18	602	409	31	743	989	45
11	172	162	47	144	172	34
8	549	630	15	518	755	307

Endogenous IGF1R	Endogenous IGF2R	Endogenous IGFBP1	Endogenous IGFL1	Endogenous IGHA1	Endogenous IGHG1	Endogenous IGHG2
307	258	16	8	23	21	48
696	744	34	6	141	450	744
1143	1219	33	2	469	229	254
390	447	19	4	58	11	65
816	941	13	5	156	896	718
478	363	13	5	170	601	676
398	372	9	4	44	278	201
938	1053	23	7	413	500	900
901	698	36	10	183	406	337
1265	1436	17	3	335	1081	1199
383	266	16	3	40	97	119
502	650	3	5	1962	5748	6272
661	695	14	3	22	550	595
1384	1593	44	7	401	1290	1493
689	985	54	3	3000	14645	12125
483	402	18	8	38	29	192
150	197	5	8	304	517	1257
475	570	10	4	692	770	1603
491	680	6	1	364	1545	887
792	785	17	5	237	599	766
211	212	26	7	287	280	423
458	508	28	9	195	518	487
351	468	23	11	922	2269	4364
644	628	20	14	94	146	163
110	153	10	8	336	1662	1467
418	580	13	4	2607	3301	7127

Endogenous IGHG3	Endogenous IGHG4	Endogenous IGHM	Endogenous IGKC	Endogenous IGLC1	Endogenous IKBKB	Endogenous IKBKG
70	29	119	52	261	158	136
974	346	340	1689	934	231	239
324	129	361	686	1958	572	470
59	27	148	14	463	134	157
874	391	174	841	1551	493	313
715	336	219	849	1006	163	144
228	91	221	368	444	134	114
882	409	407	1335	1670	485	378
439	181	267	383	1063	291	246
1432	540	476	1355	2491	725	572
155	65	149	230	527	171	167
6953	2861	1834	10165	10338	390	281
731	307	148	378	1330	270	270
1773	704	385	2046	3239	783	534
18698	5496	780	17224	13285	808	560
164	86	141	187	740	168	130
1182	626	426	1998	923	75	58
1645	701	158	2396	1872	306	201
1056	389	216	1650	1379	243	164
995	408	379	932	1725	292	246
424	170	211	834	759	83	94
591	230	523	1150	719	220	182
4204	1984	1030	5714	4960	202	279
203	72	173	268	643	289	289
1830	669	330	1973	1167	85	49
7479	2822	1406	9738	8981	262	213

Endogenous IKZF1	Endogenous IKZF2	Endogenous IL10	Endogenous IL10RA	Endogenous IL10RB	Endogenous IL12A	Endogenous IL12B
43	69	9	75	148	13	44
43	92	11	68	114	16	24
97	120	12	109	196	12	22
22	45	8	34	87	12	14
112	106	9	119	158	18	25
35	60	11	25	75	11	4
14	41	5	28	63	11	5
97	130	21	110	235	17	20
37	110	11	80	203	15	18
76	151	17	118	270	16	7
34	32	4	24	98	11	5
107	107	14	174	170	21	11
42	108	18	84	134	12	17
98	169	13	181	322	16	15
203	145	39	201	254	22	24
20	62	4	35	68	9	12
30	48	5	25	67	11	19
125	95	21	214	149	14	21
55	62	6	84	136	4	7
48	76	12	85	131	11	14
31	31	14	20	47	21	25
72	79	10	79	170	17	15
160	63	27	178	140	21	23
52	127	9	71	153	21	19
28	49	4	23	72	15	18
113	101	19	169	149	12	15

Endogenous IL12RB1	Endogenous IL12RB2	Endogenous IL13	Endogenous IL15	Endogenous IL16	Endogenous IL17A	Endogenous IL17F
36	31	32	86	49	8	42
42	19	13	117	79	7	12
46	49	10	187	95	19	11
35	20	7	49	32	7	12
40	76	12	171	89	5	7
45	9	9	60	37	3	8
49	11	5	56	37	6	15
44	66	12	187	92	12	5
54	12	22	149	48	4	10
54	26	14	278	80	10	11
25	8	3	39	25	4	13
47	24	17	153	111	9	11
43	22	9	130	59	8	3
46	41	21	245	112	21	10
47	52	12	204	183	9	21
38	10	7	63	49	5	5
47	11	7	42	24	7	3
55	36	10	127	127	9	8
30	14	7	100	58	8	5
39	13	5	105	59	12	9
48	4	10	42	25	7	8
45	19	19	121	59	16	12
76	26	9	93	134	4	11
78	32	16	93	69	12	17
97	7	11	23	38	8	6
36	12	5	101	109	9	8

Endogenous IL17RA	Endogenous IL17RB	Endogenous IL17RC	Endogenous IL18	Endogenous IL18BP	Endogenous IL18RAP	Endogenous IL1A
120	1199	145	176	484	7	42
166	1673	371	267	106	13	14
391	3894	527	586	575	22	9
93	1638	154	143	224	7	5
360	3362	428	590	247	12	12
109	804	249	140	59	5	11
75	645	197	208	55	4	8
367	3148	483	598	178	8	7
200	5839	268	266	112	6	10
453	3103	529	541	280	14	8
112	608	172	132	141	7	9
269	1586	262	382	283	13	9
231	2431	458	375	137	13	9
479	6714	757	832	258	12	7
507	2056	318	862	281	18	18
92	824	263	154	60	3	8
58	547	94	57	134	6	14
173	1101	231	268	121	14	6
202	1560	193	367	101	7	2
150	3130	285	201	103	14	9
91	941	79	236	42	11	8
216	1004	221	259	259	10	14
233	1326	143	277	228	23	8
248	1586	334	391	256	8	6
61	213	38	96	47	5	12
185	3931	207	270	119	11	10

Endogenous IL1B	Endogenous IL1R1	Endogenous IL1R2	Endogenous IL1RAP	Endogenous IL1RL1	Endogenous IL1RN	Endogenous IL2
51	641	57	67	1098	34	23
52	300	48	84	178	19	12
10	802	212	79	2347	20	6
45	255	24	45	338	10	5
32	734	142	59	381	24	9
9	129	33	22	120	13	4
8	155	25	55	360	12	10
42	1171	264	64	689	26	10
19	322	59	31	115	27	7
27	989	366	74	286	47	10
20	238	116	24	606	10	7
32	725	162	54	45	21	8
21	475	36	47	521	13	11
23	1233	166	113	969	22	26
48	583	425	119	4312	33	25
23	114	35	17	107	22	8
17	153	51	20	20	25	14
39	487	43	46	110	18	9
10	463	112	44	947	9	4
20	200	76	31	142	12	7
69	113	53	19	211	15	12
48	604	73	53	121	22	22
42	488	92	31	208	27	5
20	488	54	67	1220	34	10
20	138	40	26	190	42	9
72	400	33	41	228	16	7

Endogenous IL21	Endogenous IL21R	Endogenous IL22	Endogenous IL23A	Endogenous IL23R	Endogenous IL27	Endogenous IL27RA
30	38	18	32	43	24	46
25	27	6	15	29	6	49
15	33	7	19	25	8	71
17	24	4	3	16	5	22
16	45	7	18	31	7	73
17	18	5	8	23	6	26
16	18	1	6	17	5	27
18	32	9	31	24	13	102
24	29	5	14	28	4	54
18	35	14	23	23	10	129
19	10	5	13	23	9	27
27	48	14	15	29	10	66
21	36	9	7	17	6	25
20	45	10	14	28	10	127
27	35	12	23	33	16	116
15	21	10	9	22	6	29
20	26	7	8	28	15	19
23	71	7	10	22	11	74
16	16	1	7	14	7	38
15	27	12	16	12	12	42
26	31	8	8	17	2	31
25	42	8	13	14	11	54
44	53	12	10	44	8	78
36	36	9	16	33	6	49
65	21	6	8	45	8	25
11	42	11	7	13	9	69

Endogenous IL2RA	Endogenous IL2RB	Endogenous IL2RG	Endogenous IL33	Endogenous IL4	Endogenous IL4R	Endogenous IL5
45	23	21	112	17	309	23
25	24	32	80	8	201	10
17	43	37	47	4	775	8
7	21	13	29	10	222	5
65	47	47	176	7	450	11
9	22	16	34	5	160	2
5	9	20	25	2	127	3
49	23	39	211	7	626	8
32	21	36	34	7	271	9
28	40	64	122	10	719	6
2	18	9	54	12	251	8
35	71	76	277	9	448	7
50	21	17	105	7	345	2
37	34	45	108	3	1103	7
42	54	103	80	16	932	11
7	26	24	41	4	139	2
8	15	19	63	9	167	3
43	72	87	134	5	419	6
26	27	26	86	3	265	8
17	21	30	44	9	203	4
6	21	16	28	8	124	4
41	38	53	120	11	355	11
47	73	78	142	8	484	9
31	39	25	68	11	553	10
13	39	19	21	11	89	5
42	67	103	113	5	305	3

Endogenous IL6	Endogenous IL6R	Endogenous IL6ST	Endogenous IL7	Endogenous IL7R	Endogenous IMPDH1	Endogenous IMPDH2
112	178	2630	33	30	167	536
192	257	3617	22	39	167	576
16	601	9421	21	143	416	786
14	201	2279	17	14	123	222
20	329	6422	26	129	332	736
10	487	2075	15	22	99	339
11	230	1941	11	29	87	241
58	374	6867	21	42	385	934
17	134	3114	6	25	216	325
23	360	8633	27	34	435	862
29	111	1723	17	40	160	264
78	202	5019	33	75	316	705
11	375	5414	26	21	200	534
21	685	11363	24	127	627	1135
110	702	5441	31	365	536	760
8	274	1987	19	28	64	231
13	54	1017	13	27	87	164
113	186	3012	37	95	229	497
10	281	3309	14	74	160	463
30	258	3223	15	56	196	278
31	88	1056	23	30	92	202
89	223	3218	35	50	150	530
19	218	3614	24	435	216	320
11	371	5788	34	60	227	461
10	65	673	20	26	75	143
78	154	2867	25	185	172	411

Endogenous INHBC	Endogenous INPP5D	Endogenous IRF1	Endogenous IRF4	Endogenous IRF6	Endogenous IRF7	Endogenous IRF8
31	93	107	58	182	73	62
30	108	162	37	336	94	59
31	194	203	31	568	195	161
24	62	83	29	179	89	47
23	225	165	19	332	187	92
21	75	47	22	183	79	32
21	64	73	28	147	32	33
38	194	253	42	401	246	100
25	142	139	31	230	172	58
28	247	262	52	504	224	168
22	49	77	37	112	80	35
29	261	223	101	279	139	211
21	91	156	26	295	138	78
24	286	241	40	653	308	127
22	422	374	149	378	206	237
24	63	72	37	209	56	37
34	50	100	29	78	64	36
33	217	144	55	159	124	148
10	89	111	28	249	100	78
29	95	144	38	181	197	55
20	49	48	33	73	78	62
27	115	210	41	253	108	117
45	197	187	89	143	159	153
50	109	167	50	344	155	55
60	50	57	73	64	45	63
24	181	154	91	206	110	150

Endogenous IRS1	Endogenous ISG15	Endogenous ISG20	Endogenous ITGA4	Endogenous ITGAM	Endogenous ITGAX	Endogenous ITGB2
176	43	100	46	18	46	58
282	43	33	55	22	33	94
492	77	75	77	38	44	201
156	35	33	33	13	18	68
396	178	104	111	59	63	200
210	41	20	36	9	26	37
165	34	19	23	13	17	29
455	356	98	97	50	68	166
326	140	46	58	25	34	92
960	380	101	83	46	73	157
145	84	37	24	21	31	42
374	104	71	136	58	97	191
284	62	43	52	17	32	104
786	153	85	123	42	54	197
403	96	161	118	53	104	181
220	47	27	40	14	30	46
104	53	32	37	17	21	40
294	161	71	81	56	63	143
171	60	40	51	27	20	142
178	61	50	45	32	40	59
122	36	21	26	14	36	56
250	81	49	95	30	52	161
163	125	106	105	26	57	177
252	62	44	60	26	39	111
109	21	37	29	11	28	56
222	95	73	90	65	40	194

Endogenous ITGB6	Endogenous JAK1	Endogenous JAK2	Endogenous JAK3	Endogenous JUN	Endogenous KAAG1	Endogenous KDR
435	948	162	115	361	22	343
139	1151	298	46	601	2	951
391	2750	490	98	339	8	1200
110	840	105	66	164	6	354
854	2380	353	129	306	11	522
58	710	157	40	711	5	329
48	515	164	17	205	2	482
4293	2759	324	178	386	12	656
210	1437	287	90	186	12	391
2133	3458	587	127	541	7	848
844	762	119	56	98	4	171
1743	1858	310	223	326	7	582
473	1529	276	91	199	5	613
729	4100	687	209	538	8	1070
2815	2243	500	268	2329	26	887
72	604	135	29	140	7	319
324	416	65	57	66	3	111
563	1165	242	198	359	4	449
861	1484	238	91	143	4	399
991	1166	168	101	117	8	357
271	525	57	37	106	8	134
953	1164	193	165	255	9	382
1068	1144	207	230	190	6	309
124	1577	282	124	243	18	759
330	306	59	55	109	10	71
525	1217	214	148	255	6	554

Endogenous KIR3DL1	Endogenous KIR3DL2	Endogenous KIR_Activating	Endogenous KIR_Activating	Endogenous KIR_Inhibiting	Endogenous KIR_Inhibiting	Endogenous KIT
139	38	5	58	5	5	60
26	18	6	18	9	12	108
24	17	15	17	11	12	244
18	19	9	18	4	8	106
32	26	11	23	8	9	274
1	11	7	14	3	5	83
13	10	7	8	7	2	86
27	19	12	12	10	15	284
35	14	7	21	9	6	197
35	19	12	19	9	18	241
22	10	8	12	13	9	109
22	19	15	20	6	15	96
27	10	8	12	4	8	127
32	18	14	18	11	10	331
69	12	13	27	19	5	490
7	8	9	9	9	8	69
22	8	8	11	5	6	32
15	18	14	20	7	9	111
14	7	15	6	5	9	195
17	13	11	11	9	5	90
21	16	13	28	10	11	129
31	16	13	17	7	12	127
18	23	16	25	8	16	140
44	27	13	20	10	12	203
18	16	10	21	6	17	36
18	14	6	10	1	3	107

Endogenous KITLG	Endogenous KLF2	Endogenous KLF4	Endogenous KLHL13	Endogenous KLRB1	Endogenous KLRC1	Endogenous KLRD1
610	193	118	376	14	25	22
566	451	223	337	23	11	9
1128	641	146	590	28	30	22
430	211	61	272	10	14	18
1170	553	180	513	52	15	17
309	499	161	247	19	5	17
289	251	70	147	17	17	9
2086	519	250	862	32	9	18
493	233	83	333	18	19	23
1479	681	360	883	23	15	17
625	161	70	126	7	11	11
1012	448	153	657	84	13	19
471	376	153	396	15	7	16
1594	1089	418	773	36	18	24
2617	585	377	663	77	25	29
256	183	56	145	12	8	16
178	96	49	101	18	14	17
609	931	421	367	57	10	20
793	283	98	322	29	9	7
781	261	79	253	26	11	17
310	115	66	125	7	10	18
540	313	150	388	21	14	14
697	590	231	231	62	13	13
552	695	233	318	21	10	18
208	130	49	60	7	18	19
631	342	144	315	55	9	16

Endogenous KLRF1	Endogenous KLRG1	Endogenous KLRK1	Endogenous KRT19	Endogenous KRT8	Endogenous LAG3	Endogenous LAIR1
13	9	56	273	2047	10	90
25	13	37	301	2700	16	64
14	14	46	505	7713	24	167
19	7	16	312	2631	4	46
19	24	51	519	6590	21	172
22	6	29	237	1764	12	42
12	10	24	151	1094	8	23
12	19	35	1000	9775	30	250
20	15	42	345	2606	10	90
26	14	40	1235	9983	26	128
21	4	29	167	3298	15	52
24	42	43	450	5277	12	218
15	5	30	410	5075	14	81
19	18	50	1560	8864	25	191
22	32	82	1446	13546	25	194
25	16	36	143	1452	10	44
32	9	26	93	1744	7	34
26	28	42	275	3168	28	269
13	9	33	485	5905	11	104
17	9	39	443	3684	10	80
22	15	38	161	1608	7	31
19	15	47	311	3496	13	152
33	27	55	566	4040	23	207
29	11	48	477	4400	15	98
48	18	35	121	1010	12	36
10	31	59	357	3256	19	162

Endogenous LAMP1	Endogenous LAP3	Endogenous LAYN	Endogenous LCK	Endogenous LCN2	Endogenous LCP2	Endogenous LDLR
1325	1918	69	28	162	67	289
4179	1454	83	23	34	101	296
6159	3204	68	31	596	165	346
2214	1495	38	7	30	60	285
4891	1904	81	31	217	165	481
2164	764	37	9	22	46	150
2078	785	31	9	17	56	101
5243	1955	84	16	462	207	489
3488	834	96	25	32	89	186
7408	2179	129	34	173	182	1505
1355	1179	26	13	356	51	178
3172	1887	93	34	882	234	244
5807	2037	55	19	60	75	495
9358	2778	113	24	86	268	1081
3285	2871	97	36	1091	266	608
2437	915	35	15	20	46	79
970	787	28	20	94	46	98
2777	1373	76	55	457	267	357
3418	1114	30	13	265	111	380
4130	1245	40	14	66	103	263
1199	344	41	16	138	46	78
2844	2096	58	28	327	127	335
1942	1295	70	56	254	212	533
4023	2753	54	25	44	104	391
625	188	25	14	68	24	127
3290	1445	71	32	68	184	470

Endogenous LEF1	Endogenous LGALS3	Endogenous LHX6	Endogenous LIF	Endogenous LILRB1	Endogenous LILRB2	Endogenous LILRB4
33	493	42	132	66	114	55
39	1188	29	109	30	44	42
73	1112	25	56	58	99	71
26	606	20	36	25	65	20
53	1437	35	149	64	169	84
23	581	12	37	13	20	16
19	798	12	28	18	48	18
117	1300	68	372	119	248	105
30	629	30	61	60	102	58
71	1841	71	249	47	124	50
39	289	12	116	24	83	24
54	792	57	649	99	199	161
47	1361	37	71	20	79	50
114	2001	101	178	60	127	68
142	1306	90	368	129	209	95
22	537	12	44	15	33	23
22	150	23	89	24	79	21
27	815	58	442	92	258	83
41	983	20	98	40	83	56
30	575	21	330	34	133	57
37	253	26	85	17	52	15
39	874	35	222	49	152	79
38	614	54	238	85	283	93
62	1121	42	47	28	65	46
14	140	24	91	17	35	22
43	872	51	575	60	152	124

Endogenous LOX	Endogenous LRP2	Endogenous LRRC32	Endogenous LST1	Endogenous LTA	Endogenous LTB	Endogenous LTBR
97	3281	97	62	34	34	405
189	5664	87	48	10	36	536
274	5074	128	96	9	53	1212
65	4699	87	33	7	22	636
246	5739	107	119	9	117	1540
94	3890	48	20	2	23	433
115	2484	60	20	7	25	243
274	6270	166	134	13	243	1261
53	3936	115	64	8	81	922
169	5363	203	100	14	477	2028
72	1311	55	31	3	28	551
215	3507	147	170	12	214	731
171	6645	114	63	11	33	1241
356	6802	147	124	13	163	2109
112	3264	151	138	24	343	1310
86	3198	62	25	4	34	393
11	1146	45	41	10	64	326
103	4156	107	138	13	172	710
160	3453	77	47	3	34	909
74	6291	66	73	12	82	599
26	1266	48	29	4	33	311
84	4135	128	86	9	118	739
143	1023	150	83	15	255	559
183	5075	149	39	16	32	1055
86	245	66	34	5	16	201
125	3509	104	111	15	175	850

Endogenous LTF	Endogenous LY96	Endogenous LYVE1	Endogenous MAF	Endogenous MALL	Endogenous MAP3K1	Endogenous MAPK11
1538	74	294	315	44	423	116
27	53	294	1419	12	708	81
6170	71	814	991	15	1003	178
1765	43	255	583	10	270	75
1392	117	1539	978	20	1043	174
54	18	141	687	10	314	60
24	28	99	880	7	259	38
2670	137	1061	734	23	1229	257
386	74	775	615	18	815	73
630	104	1491	1057	20	1772	214
707	41	246	279	3	253	72
3177	185	617	372	20	1400	106
385	76	643	1034	15	577	86
729	172	2270	1275	22	1487	273
4286	163	1630	552	25	1171	307
50	13	164	970	10	237	49
295	22	105	154	12	199	36
7617	175	636	570	14	940	127
2299	65	794	567	8	485	119
315	62	303	705	7	431	160
211	28	167	240	9	253	50
1756	109	669	424	13	514	113
528	102	909	269	15	444	108
188	59	736	1044	14	423	167
336	19	117	63	6	119	40
780	139	1542	813	8	715	78

Endogenous MAPK12	Endogenous MAPK13	Endogenous MAPK14	Endogenous MAPK3	Endogenous MAPK8	Endogenous MARCH8.	Endogenous MASP1
59	371	485	312	128	97	36
102	306	603	736	374	158	134
57	832	1121	842	454	288	42
56	288	366	245	124	71	25
123	609	815	854	331	240	95
57	169	338	403	183	68	117
24	125	288	344	175	76	51
106	696	939	853	390	219	131
50	351	581	569	247	137	102
179	757	1138	1277	517	249	142
33	213	298	232	134	93	12
45	532	713	758	282	166	105
93	605	738	572	354	149	65
133	954	1522	1375	541	275	136
126	662	1508	762	308	180	48
28	171	297	328	156	90	92
20	134	121	163	76	46	20
70	316	478	526	170	106	77
53	338	598	535	207	112	69
58	205	491	362	223	151	81
24	145	199	138	78	62	16
39	330	531	428	211	103	70
64	257	470	414	126	92	38
86	357	606	565	260	152	29
16	127	142	156	53	37	14
60	381	493	556	216	115	61

Endogenous MASP2	Endogenous MBP	Endogenous MCAM	Endogenous MCM6	Endogenous MEF2C	Endogenous MEGF11	Endogenous MEOX1
22	182	123	122	241	158	43
13	389	129	151	376	18	25
15	623	77	235	408	219	18
9	278	67	82	137	127	14
14	561	224	172	418	135	19
17	276	72	86	183	15	14
9	174	38	94	139	21	10
12	654	420	288	435	188	16
12	385	191	118	324	31	26
21	1021	409	198	578	56	26
9	186	87	71	117	80	18
15	512	268	201	515	133	44
13	458	181	132	266	131	21
9	1001	292	250	470	54	19
18	685	286	247	460	539	40
13	234	81	60	157	22	19
16	143	56	61	105	71	11
20	430	154	134	375	333	13
7	411	157	119	260	88	7
11	433	81	90	207	108	10
17	155	57	90	90	59	11
18	406	176	134	282	121	18
22	365	328	161	375	160	27
23	372	184	179	232	61	28
31	124	60	64	107	56	49
18	344	81	120	271	66	14

Endogenous MERTK	Endogenous MET	Endogenous MICA	Endogenous MICB	Endogenous MIF	Endogenous MIR155HG	Endogenous MME
152	471	182	150	2890	59	844
394	533	273	205	6679	23	5098
920	792	620	257	12779	25	2430
160	261	185	102	4676	11	2192
538	644	374	276	10924	22	1452
213	229	184	118	5075	7	3210
244	267	122	82	3356	13	3774
527	916	493	276	11160	40	1482
267	508	240	156	6178	25	964
933	1208	634	427	15011	49	1837
141	253	215	70	3814	26	490
444	692	348	194	6348	53	610
370	465	250	150	9390	23	2707
1707	843	1048	502	13400	40	4017
692	1286	497	368	8467	128	1195
230	218	322	123	5294	13	3456
55	100	103	64	2372	23	181
260	485	248	174	5175	20	903
409	464	129	128	5965	24	1225
306	400	279	145	5080	26	2503
137	199	113	57	2310	17	348
252	371	186	100	6172	19	1032
377	343	407	162	5463	39	397
540	351	239	175	7175	25	3786
71	199	65	43	1937	26	136
236	353	165	123	5085	21	1928

Endogenous MMP12	Endogenous MMP14	Endogenous MMP9	Endogenous MMRN2	Endogenous MPIG6B	Endogenous MRC1	Endogenous MS4A1
12	282	43	93	25	104	20
21	431	15	179	4	255	13
32	638	12	189	10	305	10
10	205	10	165	7	108	6
13	660	44	227	9	523	23
10	212	11	119	5	151	15
6	139	10	115	2	101	7
14	988	45	277	15	631	15
8	335	20	242	9	331	16
5	600	26	515	12	422	14
12	197	8	77	4	93	3
12	668	65	231	7	368	24
15	430	22	190	12	361	3
32	787	25	448	8	950	11
31	760	62	299	13	416	132
9	160	13	133	5	106	9
4	127	15	85	10	71	8
13	492	42	262	9	389	26
8	433	8	119	4	328	4
10	268	18	164	2	206	13
21	192	32	76	9	130	13
19	347	34	180	5	239	26
15	490	50	259	7	368	99
21	322	23	218	10	447	20
6	105	11	39	4	55	19
15	476	23	171	2	424	47

Endogenous MS4A2	Endogenous MS4A4A	Endogenous MS4A6A	Endogenous MS4A7	Endogenous MT1A	Endogenous MT2A	Endogenous MTOR
21	81	101	103	111	8133	243
9	116	152	74	53	8956	495
7	350	277	170	529	19554	886
7	91	68	60	61	3310	284
16	600	382	206	90	6342	691
9	97	79	54	66	2901	304
9	53	74	29	21	2703	272
15	555	425	201	253	19653	789
10	255	165	181	44	6365	572
9	314	213	145	179	11136	1340
6	101	61	27	561	7281	224
36	418	475	229	56	4138	583
10	307	218	94	76	8169	601
24	740	561	203	682	22203	1274
20	391	296	191	417	12017	1060
8	77	59	43	29	2430	278
6	99	52	42	26	816	166
40	586	445	368	38	4077	372
9	401	284	105	96	5517	360
10	170	126	93	39	3649	375
4	107	58	61	54	3409	174
11	266	229	100	85	5724	432
15	282	201	142	363	25041	304
3	311	204	80	457	15037	530
4	100	34	38	57	3684	132
19	570	433	268	80	7110	413

Endogenous MUC1	Endogenous MX1	Endogenous MX2	Endogenous MYB	Endogenous MYBL1	Endogenous MYC	Endogenous MYD88
987	71	11	19	16	211	416
1279	103	31	31	17	189	422
4160	149	18	36	22	203	710
1303	45	11	12	13	63	322
3826	196	36	20	27	363	593
891	48	17	21	5	100	233
705	48	15	17	12	28	175
4867	292	56	12	18	644	770
1955	136	26	22	20	58	478
6567	282	34	22	21	451	861
1153	35	8	14	7	186	230
2553	127	38	29	28	325	536
2447	85	14	31	17	147	559
4440	265	40	26	21	411	897
5372	144	30	25	34	658	585
711	58	13	22	14	41	203
531	58	14	19	12	76	214
1592	130	42	20	27	367	476
2232	77	15	14	16	272	375
2773	86	17	16	8	185	418
859	38	11	20	15	143	148
2021	93	20	13	12	279	431
2617	144	29	40	25	356	455
1753	104	23	29	21	123	472
540	34	1	62	27	79	92
1396	92	25	16	19	261	498

Endogenous MYL9	Endogenous MYOM2	Endogenous NCAM1	Endogenous NCR1	Endogenous NFAM1	Endogenous NFATC1	Endogenous NFATC2
935	20	80	15	49	56	73
2009	83	85	8	14	89	180
4488	70	58	14	25	90	131
958	35	31	13	21	41	49
4346	41	114	10	43	70	152
1255	69	52	14	9	65	65
1322	57	37	5	16	42	39
3863	36	44	12	32	91	182
3025	58	82	16	25	79	97
5202	64	124	10	28	141	236
937	29	28	5	20	28	76
3923	39	97	17	34	85	151
2646	106	58	14	21	59	82
6692	73	176	16	26	113	181
2805	37	74	24	39	131	233
910	77	39	13	20	42	62
399	29	48	11	29	27	48
1515	27	92	17	50	65	194
2714	39	44	5	14	54	73
1093	57	29	15	25	38	88
515	22	26	23	23	31	51
1783	55	39	18	30	57	128
2642	36	89	15	45	80	149
2750	94	61	15	28	77	120
382	54	60	17	24	48	89
2080	56	77	10	41	57	124

Endogenous NFIL3	Endogenous NFKB1	Endogenous NFKB2	Endogenous NFKBIA	Endogenous NFKBIZ	Endogenous NKG7	Endogenous NLRC5
453	151	352	962	318	67	95
350	125	316	1355	298	50	72
596	241	586	2938	545	63	135
249	86	188	653	283	15	54
409	193	513	3221	529	52	153
150	67	154	2121	148	12	48
190	58	144	1233	119	20	62
583	241	746	3032	1328	57	177
158	154	366	1843	450	44	117
605	308	778	4598	919	45	211
230	61	220	781	411	79	64
344	207	663	1826	1188	122	189
376	150	354	2217	402	22	103
973	279	703	6024	569	67	185
794	161	1003	3329	2280	191	338
156	68	142	1139	110	37	50
101	51	192	599	200	33	73
330	141	452	1632	827	142	153
303	96	250	1550	488	54	79
180	80	335	1064	610	48	85
134	64	179	652	233	18	38
418	166	440	1139	588	44	119
235	87	402	1664	702	42	246
435	125	272	2535	220	31	109
114	48	110	401	177	11	33
515	128	421	2401	565	70	152

Endogenous NLRP3	Endogenous NNMT	Endogenous NOD1	Endogenous NOD2	Endogenous NOS2	Endogenous NOS3	Endogenous NOTCH1
32	486	96	28	56	138	215
29	78	93	22	24	179	374
32	1067	170	36	33	245	561
9	191	63	20	36	130	275
33	459	115	44	37	268	430
21	55	60	20	24	144	219
11	29	56	14	11	107	191
45	676	141	43	74	403	516
27	149	76	26	38	196	383
32	328	199	46	62	436	661
13	375	51	14	62	91	206
51	379	147	54	54	275	376
18	299	91	27	27	176	457
48	430	169	43	81	563	772
69	2432	327	49	169	499	698
11	52	52	18	21	100	230
14	98	34	12	16	108	140
45	1083	131	39	28	260	324
16	451	75	10	26	172	309
35	141	81	22	33	126	361
16	434	41	14	19	74	116
21	714	99	37	26	211	251
47	644	105	43	52	282	291
24	320	114	27	63	213	468
11	232	35	21	17	39	130
40	794	67	29	17	186	394

Endogenous NOTCH2	Endogenous NOX4	Endogenous NPDC1	Endogenous NPHS1	Endogenous NPHS2	Endogenous NPPA	Endogenous NPPB
565	950	269	258	432	39	24
956	3047	374	480	546	10	7
1407	1877	845	379	994	9	15
465	1255	346	195	514	7	6
1287	1207	707	292	653	10	11
590	1096	226	267	277	4	10
483	1225	231	451	717	7	10
1441	1274	915	383	569	11	11
802	628	457	190	480	10	12
2082	1282	972	176	341	7	15
375	198	290	214	228	8	8
1231	787	634	94	152	6	15
857	3151	519	329	659	5	15
2135	2690	1368	854	1354	4	19
1141	732	1535	217	602	13	17
443	1484	242	167	286	6	10
251	173	190	18	17	7	5
829	1084	678	147	319	3	8
685	1379	447	301	472	6	4
618	1136	474	236	332	7	15
209	235	163	130	178	5	14
677	1252	447	163	317	13	14
893	395	587	134	188	12	14
828	2153	723	734	895	13	13
158	93	107	41	99	7	6
759	1216	634	218	440	4	8

Endogenous NR4A1	Endogenous OASL	Endogenous OR2I1P	Endogenous OSMR	Endogenous P2RX4	Endogenous PADI4	Endogenous PALMD
176	15	40	449	191	22	58
1051	16	18	109	161	27	50
128	15	19	619	365	25	55
226	10	13	190	178	16	23
291	15	13	536	264	27	38
1151	7	15	49	141	12	34
361	3	16	59	81	11	15
393	20	30	769	264	19	47
156	24	25	285	250	19	42
411	37	18	833	332	22	87
110	5	11	233	99	27	18
290	13	28	572	163	37	31
365	12	19	395	201	24	31
449	27	23	646	404	22	70
371	23	27	985	272	30	80
339	11	12	69	136	20	31
113	11	14	173	75	24	10
508	26	27	534	212	20	41
203	9	12	326	158	11	28
230	8	19	234	209	12	18
197	10	22	159	71	26	18
366	21	25	533	196	14	39
312	33	23	477	147	38	52
409	12	28	370	199	26	56
42	7	37	140	39	45	12
1126	11	15	526	199	16	28

Endogenous PAX5	Endogenous PDCD1	Endogenous PDCD1LG2	Endogenous PDGFA	Endogenous PDGFRB	Endogenous PDPN	Endogenous PECAM1
46	15	18	145	732	33	483
14	13	23	289	1000	35	1112
22	6	21	372	1483	63	1421
11	11	15	74	396	14	459
26	12	29	216	1245	54	1222
11	10	14	104	412	27	489
13	8	13	122	512	17	481
16	16	26	340	1559	67	1847
28	12	31	100	657	47	1204
23	12	32	450	2194	30	2450
17	7	7	133	334	31	535
28	14	31	312	1381	21	1438
16	15	14	222	859	34	1214
20	18	45	530	1895	122	2635
70	11	42	449	1452	58	2874
13	10	11	106	269	16	421
27	6	11	82	191	12	252
18	17	36	188	977	27	1030
8	5	16	166	828	31	981
13	14	6	134	405	20	657
8	13	9	49	190	15	340
18	15	16	171	683	24	765
58	21	18	250	867	58	1061
34	18	34	210	896	95	1320
54	10	2	46	151	26	218
32	11	22	160	1158	36	1082

Endogenous PF4	Endogenous PHEX	Endogenous PIK3CD	Endogenous PIK3CG	Endogenous PIN1	Endogenous PLA1A	Endogenous PLAAT4
36	6	118	64	206	100	4959
22	16	185	51	342	104	656
32	19	226	59	494	168	8563
15	10	55	24	218	49	2845
20	11	214	86	441	194	4244
16	10	84	19	225	61	375
18	5	94	21	149	66	347
23	21	166	93	560	132	4174
26	16	145	81	353	80	1643
30	49	244	113	694	138	2671
12	9	52	19	166	29	1203
19	17	266	131	338	122	2469
27	12	124	55	403	144	3442
30	25	274	119	772	136	4524
47	11	297	99	394	135	2111
14	14	90	31	206	52	389
25	4	48	25	121	37	1596
22	5	189	91	258	124	2390
9	15	98	34	309	72	2130
14	9	102	42	323	44	1238
39	13	48	27	120	54	810
27	12	91	83	295	109	3676
34	18	118	81	262	93	2434
29	17	125	49	414	93	4611
33	15	39	27	62	38	281
26	7	171	104	236	59	2092

Endogenous PLAT	Endogenous PLAU	Endogenous PLAUR	Endogenous PLK2	Endogenous PNOC	Endogenous POU2AF1	Endogenous PPBP
205	232	49	63	20	50	8
559	498	35	215	12	10	8
830	252	39	113	17	13	11
203	150	30	103	11	7	4
581	349	38	180	10	20	8
217	365	19	82	4	3	9
393	175	9	69	5	4	6
826	982	154	302	15	23	6
569	412	21	131	9	16	11
1319	2450	58	384	11	21	18
170	212	36	69	8	4	7
332	2116	54	259	18	35	5
492	242	33	104	7	9	24
1564	685	58	303	14	22	12
1621	389	100	368	28	48	20
186	248	13	73	11	10	10
86	155	22	53	9	14	11
309	450	59	313	12	20	8
662	301	27	76	5	12	8
187	332	40	157	4	15	10
115	144	30	88	8	15	21
230	794	42	140	12	14	17
629	295	80	194	13	20	9
868	303	36	107	10	8	7
61	65	46	44	10	11	13
310	365	54	147	8	36	4

Endogenous PPM1F	Endogenous PPP3CA	Endogenous PRDM1	Endogenous PRF1	Endogenous PROX1	Endogenous PSEN1	Endogenous PSMB10
199	284	58	32	57	401	304
206	492	53	21	86	549	167
230	714	77	40	237	949	606
117	175	31	23	43	307	159
237	594	81	28	70	753	358
127	214	46	10	72	276	102
135	257	29	19	36	273	95
332	520	41	32	109	838	560
165	448	31	29	131	520	229
355	818	42	21	656	955	507
85	150	21	21	28	233	181
243	460	64	33	315	482	486
189	493	39	24	68	593	324
379	875	88	33	333	1147	433
206	537	124	58	47	755	328
110	226	35	15	50	263	98
55	104	18	20	31	137	174
207	339	66	54	44	388	309
139	379	44	17	42	497	268
118	263	35	36	38	289	189
67	151	34	35	36	190	105
178	289	46	26	109	464	243
143	254	69	39	68	319	317
214	363	43	26	64	516	321
51	117	30	33	43	108	76
168	400	62	41	40	440	299

Endogenous PSMB8	Endogenous PSMB9	Endogenous PSME1	Endogenous PSME2	Endogenous PSTPIP1	Endogenous PTGER4	Endogenous PTGS2
778	347	1252	1372	21	179	63
472	252	1347	998	31	223	69
1292	425	2156	2711	49	377	66
383	132	1019	1122	16	173	36
1375	342	1917	2144	76	303	54
236	115	769	653	25	130	33
240	112	638	521	20	157	32
1455	446	2658	2522	51	347	46
543	205	1412	1145	40	269	64
1360	390	2943	2686	61	605	76
375	88	661	745	26	61	16
1074	441	1871	1558	62	371	94
949	257	1853	1810	37	300	37
1322	323	3047	2725	61	548	147
1135	440	1557	2542	120	271	95
221	119	743	669	26	101	43
321	91	481	643	30	87	34
819	284	1180	1249	82	199	39
675	178	1329	1183	44	170	33
414	113	951	1046	31	120	24
285	61	478	484	28	74	21
836	412	1438	1537	33	283	64
605	319	1152	1294	96	301	44
737	287	1507	1796	56	341	54
144	56	303	275	65	81	66
646	260	1247	1343	58	238	41

Endogenous PTPN2	Endogenous PTPN22	Endogenous PTPN6	Endogenous PTPN7	Endogenous PTPRC	Endogenous PTPRO	Endogenous PTX3
170	8	171	19	184	262	86
217	25	241	25	133	404	24
332	39	415	40	318	422	34
79	12	123	21	89	188	6
251	32	308	47	332	241	22
80	11	92	22	79	190	12
119	7	97	22	65	521	9
322	35	387	41	333	267	89
174	19	214	26	158	166	18
377	20	437	32	221	138	38
97	9	96	14	85	123	102
267	46	322	50	434	105	54
180	17	233	23	119	302	22
392	29	415	56	311	477	38
466	60	503	54	668	368	211
101	15	100	22	71	147	14
57	13	67	11	88	16	15
193	41	254	47	470	157	17
164	21	215	21	156	206	57
165	8	164	24	135	103	18
84	15	94	29	77	84	31
180	23	196	38	276	144	27
166	45	225	63	445	75	67
170	18	178	57	117	455	21
61	11	59	17	51	60	267
156	44	225	45	436	249	31

Endogenous PVR	Endogenous RAB40C	Endogenous RAF1	Endogenous RAG2	Endogenous RAMP3	Endogenous RAPGEF5	Endogenous RARRES1
169	208	706	37	83	255	3135
290	432	1332	6	109	348	56
673	786	2461	16	112	536	1588
230	212	704	14	67	153	615
613	408	1878	15	110	408	546
210	275	844	5	86	234	41
167	229	710	3	74	153	55
862	562	2184	14	156	411	395
318	373	1369	15	96	307	310
790	850	3451	15	163	484	562
327	182	635	9	40	143	917
570	424	1433	19	76	482	687
402	455	1573	11	103	325	372
1108	1120	3527	17	158	716	821
864	664	1925	16	114	1322	756
172	255	847	8	87	181	28
137	92	399	8	52	97	170
356	262	847	9	117	357	705
344	371	921	9	56	281	256
445	490	1621	7	90	153	145
220	107	364	13	44	86	74
350	264	910	19	90	260	1158
416	280	794	8	132	496	1218
408	380	1480	15	164	326	299
141	79	218	7	67	131	140
327	261	1157	8	114	199	1049

Endogenous RASIP1	Endogenous RASSF9	Endogenous REL	Endogenous RELA	Endogenous RELB	Endogenous RGN	Endogenous RGS5
148	56	104	157	172	564	424
145	141	153	236	66	1754	1479
164	167	229	305	195	2121	1464
76	49	62	159	83	866	470
111	126	172	321	205	541	2824
67	70	71	161	41	926	607
69	56	78	127	55	714	464
189	132	245	394	317	872	2740
112	122	135	253	117	528	1465
254	259	271	501	284	1212	3515
54	51	52	150	63	287	304
174	229	166	269	275	576	1660
133	89	121	269	134	1456	2350
319	202	285	520	183	1742	4488
337	187	275	273	327	939	1841
92	45	62	140	31	737	709
48	32	41	120	71	114	363
149	93	151	216	224	497	988
75	85	116	175	93	686	1321
79	84	75	243	103	770	856
63	28	55	83	52	111	281
119	85	107	209	232	540	1590
110	104	157	224	160	211	2284
178	68	151	271	122	1159	1876
59	32	49	94	28	76	166
109	97	180	228	166	905	1027

Endogenous RHOJ	Endogenous RHOU	Endogenous RNF149	Endogenous ROBO4	Endogenous RORA	Endogenous RORC	Endogenous RPL19
81	265	392	135	161	136	8781
74	181	518	240	634	559	19281
110	288	982	362	951	845	24538
38	101	331	140	183	203	8414
121	296	863	307	792	186	20374
49	110	317	154	241	236	9444
47	93	254	147	300	276	9196
171	457	924	499	766	333	29430
140	211	446	211	667	161	12337
312	415	1092	448	1148	418	31385
55	88	230	113	234	106	8525
136	256	628	344	296	139	20641
75	226	753	210	557	281	16089
151	439	1101	632	1348	597	35966
155	306	901	611	636	465	21475
43	104	271	163	402	344	10780
34	109	180	81	94	98	4947
84	269	472	244	247	193	14184
48	219	486	150	356	279	12796
38	145	425	186	418	202	13686
16	97	198	57	130	61	5369
73	243	562	174	199	202	12590
115	269	558	289	295	235	10798
69	232	614	392	526	609	15209
27	37	190	51	99	48	2642
85	267	616	146	361	133	12940

Endogenous RPS6	Endogenous RPS6KB1	Endogenous RTN4	Endogenous RUNX1	Endogenous RXRA	Endogenous S100A12	Endogenous S100A8
9083	210	1846	88	742	146	797
16409	394	4465	73	1742	28	36
21971	615	4792	92	2585	112	919
8751	164	1933	45	682	26	116
19131	439	4653	102	1239	69	494
10203	191	2275	31	600	16	29
8826	228	1876	35	839	8	36
28230	488	5411	210	1359	52	625
11697	336	3637	114	1137	35	233
26880	779	8002	139	1931	52	267
7649	168	968	77	579	60	943
17697	439	3318	278	869	24	154
14096	345	4276	82	1444	37	216
32037	909	7319	121	3640	31	218
17302	958	3754	233	1404	123	2102
10598	226	2223	49	909	11	9
5093	92	712	69	260	14	60
13499	285	1966	232	793	34	158
12608	279	2796	40	1017	32	335
12100	317	2670	90	995	32	151
5141	120	1166	27	300	94	493
12057	236	2715	161	717	50	281
10736	226	2035	161	726	22	155
13766	354	4036	53	1589	28	88
2654	105	565	32	262	32	170
10519	292	2399	192	812	18	258

Endogenous S100A9	Endogenous S100B	Endogenous S1PR1	Endogenous SAMHD1	Endogenous SCGB1A1	Endogenous SDC1	Endogenous SELE
662	74	58	314	27	499	51
85	29	69	406	24	1979	23
1795	42	96	1296	17	4643	21
217	9	70	274	13	1550	13
2221	54	91	990	22	2771	21
52	6	63	256	11	592	5
67	10	45	153	15	699	8
1689	34	133	848	19	3533	71
454	19	85	362	23	907	18
1435	35	169	812	20	4513	56
1748	17	52	180	15	1045	10
578	27	74	828	18	699	51
478	14	82	501	18	4146	16
1777	64	191	3174	20	6169	27
3408	80	165	830	29	2008	57
18	14	53	221	24	1497	11
127	19	46	161	18	178	26
637	28	96	599	22	386	239
797	23	70	538	13	2172	9
429	16	89	210	13	2827	62
763	19	42	136	17	370	20
726	26	85	438	16	648	140
881	39	125	768	27	1072	75
337	41	156	974	29	3472	20
404	16	50	131	39	207	26
1232	31	84	658	16	1113	57

Endogenous SELL	Endogenous SELP	Endogenous SELPLG	Endogenous SEMA7A	Endogenous SERINC5	Endogenous SERPINA3	Endogenous SERPINE1
18	74	48	32	280	21988	428
10	57	44	32	358	100	215
30	52	59	27	484	90325	518
13	56	30	15	184	6574	74
27	76	80	22	416	8908	303
10	47	27	13	170	37	139
13	42	21	9	208	20	63
20	108	65	64	476	7455	2038
15	44	43	25	254	898	141
28	195	51	54	537	763	890
12	20	31	17	134	17732	988
25	162	113	38	368	1786	215
16	75	54	18	338	8149	114
23	129	88	42	603	2383	795
70	127	76	89	678	17526	2337
11	41	30	6	157	42	86
7	39	33	13	84	830	46
28	187	86	26	296	1129	78
15	15	56	17	262	3273	442
12	32	32	23	254	2221	134
12	17	28	11	93	515	114
23	95	54	18	295	5857	90
29	105	72	42	250	3064	1186
10	72	67	22	353	8118	156
12	17	51	16	69	2580	538
36	88	80	17	326	2788	40

Endogenous SERPING1	Endogenous SERTAD1	Endogenous SFTPA2	Endogenous SFTPB	Endogenous SFTPC	Endogenous SFTPD	Endogenous SH2D1A
1419	121	71	19	49	36	8
1171	225	22	26	19	37	17
5143	242	25	20	19	31	15
944	114	19	20	10	24	7
3025	169	30	25	18	28	9
669	115	13	15	16	28	13
495	77	19	11	14	17	7
4354	296	14	31	16	48	16
1389	114	54	24	25	30	18
3571	283	28	27	32	43	22
1426	93	29	11	12	17	2
3055	191	35	19	21	27	16
1939	138	41	15	13	31	11
4079	287	29	34	24	33	11
3790	208	50	31	24	36	17
524	78	16	32	20	27	11
355	68	26	11	16	20	14
2434	129	29	22	19	21	28
2155	75	16	13	16	17	7
1539	138	23	15	10	17	10
634	52	33	23	25	27	14
1840	172	24	14	21	29	21
1790	127	46	39	26	48	30
1649	173	57	29	39	34	12
448	52	58	44	31	58	16
2349	158	24	18	12	18	14

Endogenous SH2D1B	Endogenous SHROOM3	Endogenous SIGIRR	Endogenous SIGLEC5	Endogenous SIRPG	Endogenous SKI	Endogenous SLA
40	215	191	39	12	161	77
12	324	885	13	17	442	61
20	415	775	15	18	622	204
7	148	459	11	15	187	36
23	479	601	36	23	501	194
9	215	555	10	12	255	63
9	197	294	13	13	174	51
16	724	798	27	18	557	172
16	376	387	12	23	352	66
5	583	806	19	25	866	129
10	177	154	17	4	108	77
19	411	405	12	23	518	116
5	247	1015	15	11	348	84
24	678	1220	20	25	960	279
43	959	480	16	25	506	310
11	197	628	14	16	251	38
11	104	168	10	9	109	27
15	312	374	21	28	369	97
6	268	366	11	11	249	163
10	422	390	21	7	289	54
4	161	163	13	24	85	52
12	243	468	20	28	278	95
17	208	239	22	15	452	191
12	352	694	31	26	379	153
9	88	73	17	21	102	52
9	223	425	27	23	285	147

Endogenous SLAMF6	Endogenous SLAMF7	Endogenous SLAMF8	Endogenous SLC11A1	Endogenous SLC12A3	Endogenous SLC19A3	Endogenous SLC22A2
50	38	36	16	3120	358	1198
28	18	32	19	9527	121	4444
45	21	88	211	9763	147	1420
10	6	21	28	2337	83	1625
33	20	80	43	4348	103	1181
8	12	14	10	5810	69	2004
3	11	12	14	4384	116	1917
22	14	63	121	876	80	2264
15	20	46	29	4347	130	2278
18	14	66	41	2904	150	2957
7	4	41	56	314	37	184
38	80	92	30	4237	188	3176
12	9	52	26	4694	136	2832
37	18	158	129	9203	97	6201
93	85	65	216	2002	288	1315
11	13	26	12	8046	54	1590
9	26	24	8	1334	51	516
28	32	85	30	2860	125	2162
24	12	55	65	2879	103	1348
16	12	33	36	2488	63	2370
10	13	37	29	601	76	312
13	30	67	19	2381	303	1702
59	39	91	109	921	64	616
24	12	84	64	8133	170	2391
8	13	28	26	133	78	129
27	68	60	13	2630	141	2331

Endogenous SLC25A15	Endogenous SLC4A1	Endogenous SLPI	Endogenous SMAD2	Endogenous SMAD3	Endogenous SMAD4	Endogenous SMAD5
48	72	109	684	488	381	189
51	143	39	1369	930	721	363
167	114	1123	2450	1182	1276	539
35	146	66	598	377	318	187
117	304	362	1781	920	924	431
43	71	14	677	435	469	205
49	118	23	740	405	365	190
121	86	3231	2109	1440	1092	506
57	94	121	1201	835	782	328
178	113	1601	3102	2219	1550	651
33	14	379	613	622	388	103
56	41	246	1736	1342	845	334
117	298	88	1350	520	707	348
151	310	250	3622	1685	1690	732
59	66	4211	2006	1548	1018	417
46	100	18	742	530	417	176
18	37	21	352	203	188	70
49	32	531	1063	624	593	277
59	88	239	1136	572	557	237
83	70	1404	1030	887	585	201
42	53	178	394	220	223	106
45	126	372	931	511	522	257
56	32	595	891	589	481	227
78	266	48	1311	513	736	370
11	17	348	267	238	167	83
33	152	245	1027	516	581	246

Endogenous SMARCA4	Endogenous SOCS1	Endogenous SOCS3	Endogenous SOD2	Endogenous SOST	Endogenous SOX7	Endogenous SP100
440	326	979	22989	49	48	206
730	85	224	2773	202	70	394
1283	218	350	29851	35	73	611
359	61	368	8773	37	39	169
960	120	337	29259	42	66	511
353	67	98	1663	121	16	235
343	33	47	1464	220	24	188
1088	295	1532	19051	46	92	575
634	111	297	5491	53	57	392
1464	362	655	14658	35	100	852
252	84	411	19203	21	27	122
963	191	844	16879	62	44	427
812	147	327	7161	61	51	368
1550	309	327	13688	89	100	762
936	477	1582	46635	39	106	735
340	35	55	1890	58	31	206
246	58	244	8928	16	16	94
526	110	993	15891	46	43	312
658	146	364	8467	17	38	332
452	116	726	6951	42	43	250
213	80	175	8525	34	21	100
553	289	1311	16588	36	38	313
477	118	646	18016	26	56	303
742	181	228	8648	130	87	296
145	84	171	5202	29	14	71
504	106	587	8873	41	37	306

Endogenous SP140	Endogenous SPIB	Endogenous SPRY4	Endogenous SRC	Endogenous ST5	Endogenous ST8SIA4	Endogenous STAT1
38	38	205	255	181	74	2009
25	13	345	502	140	91	477
36	16	386	892	244	120	959
11	9	147	346	83	37	546
31	15	370	535	227	98	1358
15	4	227	260	95	33	264
13	3	183	212	83	37	279
25	11	455	582	380	109	2331
18	14	186	445	167	67	1125
35	19	766	740	427	100	1826
12	4	156	245	80	38	438
38	33	444	519	313	108	1605
10	13	175	508	154	49	775
24	15	810	983	440	104	1645
92	35	388	793	344	179	1304
10	7	236	275	94	28	325
16	10	86	170	108	25	531
39	14	310	360	174	147	1265
16	5	176	290	166	49	841
26	7	187	381	110	52	656
20	10	84	141	82	32	414
30	16	193	356	193	96	1348
63	40	279	309	228	113	1221
28	9	373	443	177	53	831
13	8	61	90	103	28	162
50	49	238	279	149	95	631

Endogenous STAT3	Endogenous STAT4	Endogenous STAT5A	Endogenous STAT5B	Endogenous STAT6	Endogenous SYK	Endogenous TANK
2116	57	234	290	879	104	289
1794	43	311	450	1367	199	529
5969	68	561	739	2853	364	989
1778	26	172	224	905	125	241
3857	49	445	508	2208	287	760
1097	23	242	272	793	141	252
842	12	203	231	665	94	252
4884	52	453	618	2415	338	775
2380	49	232	323	1644	376	472
5672	52	605	774	3400	666	1240
2274	29	103	131	994	100	280
3811	90	261	357	1798	372	613
3510	29	398	467	1810	237	505
6718	94	928	904	3625	707	1176
6385	89	500	628	3527	413	1249
929	33	279	233	747	115	287
1485	24	93	81	501	91	121
2464	86	265	325	1424	177	495
2430	38	267	363	1601	186	455
2861	35	218	296	1289	207	443
860	30	94	120	487	106	155
2873	41	234	326	1183	194	431
2725	62	318	258	1030	207	448
3362	49	416	522	1594	241	455
672	46	74	83	333	43	155
2647	64	277	288	1504	221	418

Endogenous TAP1	Endogenous TAP2	Endogenous TAPBP	Endogenous TBK1	Endogenous TBX21	Endogenous TCF7	Endogenous TCL1A
165	326	772	184	28	21	22
131	183	911	268	18	33	6
252	490	1135	505	9	31	15
101	172	729	164	5	9	9
301	483	1733	396	12	57	15
77	115	518	137	7	19	8
74	141	394	136	7	12	9
288	483	2313	431	10	75	18
129	216	878	285	12	24	17
255	415	1455	588	10	42	14
94	164	559	153	4	24	6
219	291	1152	329	19	40	20
201	417	1490	328	6	21	11
290	520	2128	668	16	37	17
295	505	1712	574	27	61	14
66	120	477	146	11	15	8
79	144	427	95	10	15	13
185	306	774	246	25	35	18
91	173	1016	200	9	17	8
98	191	1016	316	12	50	11
74	153	250	106	12	20	15
251	385	773	227	15	31	16
200	298	1201	224	26	62	28
189	318	1108	297	19	26	20
42	59	173	72	21	11	17
137	268	908	232	17	56	11

Endogenous TEK	Endogenous TFF3	Endogenous TFRC	Endogenous TGFB1	Endogenous TGFB2	Endogenous TGFBI	Endogenous TGFBR1
138	17	483	239	57	272	390
348	49	561	385	67	502	857
475	29	2483	481	48	485	920
149	16	887	229	24	210	296
306	41	1245	829	67	724	762
196	33	615	132	38	170	303
222	21	483	132	33	121	395
474	64	1839	999	66	1624	1032
246	50	650	522	43	790	440
787	73	2023	1224	105	691	1447
80	29	564	298	20	239	268
270	35	696	647	94	1510	775
283	38	820	372	32	400	1024
914	105	2568	931	101	1500	1273
259	20	1404	1064	77	723	946
191	13	385	162	34	164	407
54	17	277	188	30	260	154
256	36	692	501	61	753	470
252	20	876	345	36	525	811
151	23	1499	408	25	621	549
91	16	514	182	23	181	121
210	24	950	311	49	572	535
243	41	598	777	56	734	696
453	26	1349	470	46	357	738
30	15	219	141	36	161	115
192	28	674	546	69	800	533

Endogenous TGFBR2	Endogenous TGIF1	Endogenous THBD	Endogenous THBS1	Endogenous THEMIS	Endogenous TIGIT	Endogenous TIMP1
1162	220	227	1729	27	34	1390
1369	352	211	2781	17	14	713
2354	524	308	2125	31	53	4913
1004	189	206	1311	3	14	981
2107	444	319	3192	16	22	5004
848	186	132	1380	13	15	355
891	126	98	808	10	17	264
2809	677	421	6820	15	15	9102
1392	394	180	2459	13	23	1161
2383	724	444	5992	12	13	5631
728	214	171	1671	5	20	1789
1662	539	367	4372	24	28	4301
1427	304	323	2073	13	20	1188
2733	595	530	4347	24	29	3702
2157	860	669	3768	35	40	8093
746	169	104	970	8	19	248
487	100	150	729	5	16	474
1507	329	663	2851	37	29	1768
1072	242	236	2805	11	23	2175
1167	247	232	1740	13	20	1523
340	129	137	1186	10	17	655
1353	292	375	3792	15	26	2049
1067	232	467	2390	37	39	5018
1766	196	344	1846	16	25	817
354	94	99	556	15	51	514
1607	410	516	2834	31	26	2041

Endogenous TIPARP	Endogenous TLR2	Endogenous TLR3	Endogenous TLR4	Endogenous TLR5	Endogenous TLR7	Endogenous TLR8
234	103	159	260	62	32	44
249	90	185	283	48	13	33
600	306	264	700	102	16	35
120	63	94	168	30	18	25
510	198	206	626	82	20	39
1153	59	90	147	45	10	23
166	57	71	182	19	12	15
366	356	225	741	101	17	34
161	107	154	422	83	26	38
444	269	330	839	208	27	34
120	88	46	190	28	11	28
208	243	207	475	86	26	58
262	114	175	416	79	18	29
1377	512	293	884	181	20	44
427	392	182	906	98	14	53
138	62	72	196	39	15	29
75	50	47	109	18	15	28
209	198	139	392	79	26	79
263	173	111	311	56	4	29
229	109	108	346	42	9	31
89	78	56	125	26	15	40
191	117	151	303	75	30	44
249	261	86	505	36	26	53
546	168	100	480	56	19	48
86	80	31	89	35	18	48
201	169	106	391	44	30	47

Endogenous TLR9	Endogenous TM4SF1	Endogenous TM4SF18	Endogenous TMEM178A	Endogenous TNC	Endogenous TNF	Endogenous TNFAIP3
35	667	216	236	69	13	154
45	991	314	611	189	24	142
42	988	507	573	255	15	268
39	415	165	160	44	29	84
29	1203	345	208	357	26	355
23	442	233	322	53	12	115
19	506	160	401	79	10	68
32	1751	685	267	1167	54	400
34	1096	236	570	330	27	127
52	2780	856	298	2161	62	270
21	443	66	97	180	10	93
57	1253	385	376	862	39	218
29	796	299	252	105	22	198
60	1596	598	869	541	32	376
34	1649	404	558	402	76	661
26	377	150	251	30	13	93
20	292	91	97	67	16	82
29	1153	512	183	283	53	223
20	843	192	178	189	14	167
19	767	213	188	86	20	139
24	219	107	177	85	20	98
33	1003	368	204	185	31	153
59	1244	172	89	475	44	268
53	807	356	439	105	20	167
63	228	57	36	76	8	69
30	1038	227	314	272	44	227

Endogenous TNFAIP6	Endogenous TNFRSF14	Endogenous TNFRSF17	Endogenous TNFRSF18	Endogenous TNFRSF1A	Endogenous TNFRSF1B	Endogenous TNFRSF4
13	36	56	19	1332	92	20
20	108	23	14	1139	102	29
8	102	30	20	2752	213	31
19	55	20	15	1425	100	28
18	119	23	18	1689	292	43
14	75	9	4	627	51	16
11	59	8	8	481	64	27
16	132	23	21	2742	346	63
25	71	37	16	922	131	44
21	184	32	11	2410	267	34
11	55	21	5	1246	83	21
18	77	50	15	1844	234	55
24	108	25	12	2350	150	38
18	251	15	18	3886	377	30
24	365	40	17	3398	177	46
19	77	23	10	575	68	26
10	57	22	6	573	78	27
27	141	27	8	1570	297	33
18	96	21	7	1351	144	25
14	97	15	11	1436	163	34
12	38	25	12	488	46	23
15	76	11	14	1662	161	27
30	107	45	16	1560	233	56
26	111	32	16	2311	135	44
36	37	46	4	385	45	46
18	75	36	17	1669	224	33

Endogenous TNFRSF9	Endogenous TNFSF10	Endogenous TNFSF14	Endogenous TNFSF18	Endogenous TNFSF4	Endogenous TNFSF8	Endogenous TNFSF9
26	4986	52	12	54	54	8
13	2587	18	24	62	31	20
8	5861	36	13	53	37	15
8	1650	24	9	32	20	15
7	6320	26	4	43	30	13
5	1311	6	15	27	19	13
4	1217	4	6	35	20	14
13	4787	31	8	46	47	7
9	6996	29	6	47	51	16
11	7419	22	14	79	33	15
4	1383	28	4	23	20	10
14	6642	21	9	48	43	29
6	4265	23	8	56	25	14
17	2861	21	4	81	58	14
12	4822	31	15	55	66	24
7	1105	11	21	36	31	15
8	1518	25	6	29	39	18
9	4035	62	11	48	50	19
10	3485	12	8	33	23	8
7	2047	9	9	41	20	15
6	926	16	9	23	25	17
5	3736	34	11	42	21	13
6	1277	33	15	34	60	26
9	1611	24	12	75	50	24
7	337	43	19	85	65	41
11	3176	35	9	32	39	11

Endogenous TOX2	Endogenous TP53	Endogenous TPMT	Endogenous TPSAB1/B2	Endogenous TRAF4	Endogenous TRAF6	Endogenous TRAT1
28	166	696	62	328	201	37
20	248	1855	118	600	349	21
38	341	1702	81	995	498	19
21	114	768	21	366	183	13
38	232	1287	418	651	339	18
15	125	802	32	310	156	9
19	126	684	25	237	200	16
21	385	1529	132	955	414	25
30	198	917	45	287	271	15
44	512	2027	139	967	574	20
18	75	312	17	269	168	11
19	453	1095	446	592	335	31
25	250	1681	79	584	317	14
54	483	2111	575	1558	735	23
48	517	599	376	890	641	38
19	108	844	21	257	182	20
21	85	249	29	181	81	19
31	303	722	426	428	251	36
15	236	1110	127	469	241	13
22	184	874	166	576	269	20
18	82	280	29	196	99	18
24	284	832	63	457	234	21
36	233	386	302	512	206	51
64	202	1264	42	593	326	29
41	61	117	33	132	68	10
13	244	926	367	331	206	21

Endogenous TRDC	Endogenous TRDN	Endogenous TRDV3	Endogenous TREM1	Endogenous TRIB1	Endogenous TRIM22	Endogenous TYK2
11	4	43	61	857	45	131
15	29	38	22	911	123	329
15	15	11	26	1195	80	496
8	7	8	21	472	37	230
14	11	14	35	1086	87	477
5	10	23	23	308	41	196
6	9	21	6	289	36	162
12	11	10	27	1460	128	562
16	10	9	26	777	59	359
13	27	21	19	1616	104	696
7	9	6	39	631	22	194
28	17	16	35	1598	104	359
20	26	27	25	990	51	395
14	20	20	26	1324	203	783
13	11	19	84	1675	208	775
7	9	24	24	316	41	221
7	3	14	28	448	32	123
20	20	13	25	1165	113	270
2	7	3	15	672	52	248
14	13	28	25	900	53	354
11	4	12	30	392	35	142
12	7	13	17	804	67	270
17	14	9	45	575	77	244
12	8	8	46	541	66	362
6	10	10	58	154	28	89
9	20	8	23	912	73	316

Endogenous UMOD	Endogenous VCAM1	Endogenous VCAN	Endogenous VEGFA	Endogenous VEGFC	Endogenous VMP1	Endogenous VSIR
12422	4863	49	1363	89	462	51
24880	1351	67	2921	123	809	148
35415	3036	106	3491	162	1563	194
13030	1795	54	1383	71	522	62
13203	3757	289	1834	154	1205	213
14472	502	24	1378	79	492	104
10620	540	16	1854	66	401	80
12827	2289	218	1861	180	1387	252
16011	2297	103	1251	109	754	138
35534	4193	177	2916	198	1556	227
1383	856	69	770	42	399	78
36233	3556	482	1457	177	856	155
14433	2162	43	2087	144	1127	121
65047	2009	162	5713	224	1623	322
4338	2642	257	4543	159	1019	121
10030	528	32	1438	81	440	105
9400	645	46	377	55	229	38
2300	2955	314	1049	123	667	158
12146	2148	63	1483	107	788	108
4368	697	58	1964	61	714	147
822	807	43	582	39	281	52
23468	2453	135	1195	129	739	86
12096	1569	289	997	126	495	155
14031	1091	72	2683	116	871	150
1897	141	98	749	33	161	37
2201	3533	152	1596	126	690	178

Endogenous VWF	Endogenous WARS	Endogenous WNT9A	Endogenous XAF1	Endogenous XBP1	Endogenous XCL1/2	Endogenous ZAP70
119	1993	10	75	1739	38	42
132	731	24	230	1138	13	55
137	1675	20	205	3864	18	53
85	658	9	86	1381	7	20
259	1049	17	214	1911	10	49
71	375	12	129	584	5	30
68	461	6	121	574	7	17
281	1351	22	341	2066	25	40
138	639	15	164	1143	14	32
496	1734	24	303	2144	14	42
38	501	3	51	785	5	12
361	929	13	312	1761	21	71
408	1000	15	110	2015	12	18
231	1687	25	523	3418	17	72
417	1008	26	323	2149	35	126
73	497	10	93	606	5	27
122	534	6	65	643	8	24
317	687	19	242	887	28	86
343	792	12	85	1515	11	34
121	579	15	112	1116	7	40
63	229	11	25	382	13	11
255	841	10	105	1635	20	39
131	779	13	151	1241	18	92
170	1042	15	155	1738	17	38
61	140	9	45	391	9	16
407	675	21	128	1783	25	90

Endogenous ZEB1
121
190
229
98
248
102
105
332
200
318
132
220
199
431
251
82
79
172
115
145
44
114
235
240
65
151
