# Supplementary material for: Can Gene Expression Analysis in Zero-Time Biopsies Predict Kidney Transplant Rejection?
Source: Front Med (Lausanne). 2022 Mar 30;9:793744. doi: 10.3389/fmed.2022.793744 (PMC9005644; doi:10.3389/fmed.2022.793744)
Supplement: Supplementary file 5 [file Table_5.pdf]

Supplementary data 5: Differentially expressed_sorted DD vs LD adj. P-value

gene_id	baseMean	log2FoldChange	lfcSE	stat	pvalue	padj	Ctrl 1	Ctrl 2	Ctrl 3	Ctrl 4	Ctrl 5	Ctrl 6	Ctrl 7	DGF 1	DGF 2	DGF 3	DGF 4	TCMR 1	TCMR 2	TCMR 3	TCMR 4	TCMR 5	TCMR 6	TCMR 7	TCMR 8	ABMR 1	ABMR 2	ABMR 3	ABMR 4	ABMR 5	ABMR 6	ABMR 7
SERPINA3	7162,314926	7,419959945	0,745549491	9,952337215	2,46E-23	1,56E-20	71,56098841	49,40038388	31,41044834	30761,49529	46307,5338	9048,356089	5569,648982	4090,453089	777,4490144	300,141179	35237,87296	55,00676075	1340,374282	5484,747248	812,4011105	9103,685544	2443,938746	1213,695772	3268,805148	2182,003294	1325,072762	5474,588139	3659,057255	5698,693467	9155,309212	2757,487124
ALDH3A2	1316,044659	-1,659827582	0,1946264	-8,528275626	1,49E-17	4,72E-15	3349,054258	2737,048296	3012,261995	914,9544261	1397,044336	935,9419136	1001,011228	860,3394291	1051,027955	1027,481992	723,3580959	3356,72209	976,3868649	1390,537221	1264,454771	642,0264368	859,795318	932,0409514	1153,51969	1312,542278	905,6808004	793,5675823	681,8935028	1302,879413	674,2281978	961,362082
OSMR	351,8326048	2,236704337	0,275115438	8,130057524	4,29E-16	9,08E-14	78,00147736	65,42213001	92,66082259	628,1567849	317,3469518	261,5131817	335,1293056	421,9394267	246,7405001	327,677067	463,0286713	90,3682498	429,2800052	265,8577939	220,2312704	511,6472818	509,3992809	574,0598248	325,5821809	229,8913871	409,1001343	498,1996718	569,6378298	259,7335037	496,7997247	520,2432666
S100A9	813,1185633	4,131795184	0,522591296	7,90636051	2,65E-15	4,21E-13	60,82684015	69,42756654	105,2250019	926,1465291	920,2548926	298,6755813	1188,660798	926,7304183	393,0532879	564,4857036	3473,708658	23,57432603	433,7829423	321,7215835	605,8064513	1770,247651	373,9520733	684,7867198	795,978522	421,467543	1963,166053	678,5984273	1052,098382	236,5680831	1433,622063	1218,516548
HIF1A	3283,853512	2,058324073	0,273795534	7,517741596	5,57E-14	7,08E-12	937,4489481	889,2069099	1060,102631	5321,84501	2376,769739	2623,390128	4492,358322	4462,462103	2998,979272	3463,227968	4063,921171	737,352531	4636,524252	3075,200659	1545,368961	4094,217132	6631,024165	4253,847803	3022,121716	2481,64805	4687,927325	4285,638828	3568,297349	1790,75721	3222,101071	4658,452064
LTF	1305,865807	4,946991987	0,714335887	6,92530234	4,35E-12	4,60E-10	19,32146687	72,09785756	37,692538	2151,681815	3163,215982	2429,31982	870,3358085	1464,991247	334,1818703	247,8229918	1404,983994	65,48423898	2384,305204	259,1272169	248,5272386	2226,314974	868,6288316	8188,415141	2296,053478	309,4691749	542,8938889	1641,348262	630,5425034	131,9726992	1192,319339	771,4633991
BCL3	633,9832317	2,257978512	0,327832069	6,887607189	5,67E-12	5,15E-10	186,0585699	112,1522229	130,3533606	573,5592824	405,5273649	779,0340046	387,0243287	783,5234087	444,1329002	451,1951931	1120,807599	123,1103693	679,9435047	559,3109538	331,7105667	506,972332	1510,530816	939,5660802	417,4642688	960,828105	571,1964139	1056,220693	820,42178	647,9297943	723,9081702	1261,045941
PLAAT4	2277,932862	2,483413056	0,377438428	6,579650796	4,72E-11	3,74E-09	469,440084	500,6759664	544,9712786	6937,704891	4390,051616	3915,8158	2653,52383	2290,214781	1422,437339	1050,690811	2390,658762	509,4673793	1852,958624	2316,664625	1542,300723	1096,535443	4699,429204	2569,293972	2127,270078	1216,26298	2084,095024	3435,988731	2906,705404	3236,841042	997,1480188	2069,104399
RARRES1	642,3436815	4,107825559	0,627919024	6,541967045	6,07E-11	4,28E-09	40,07415351	54,74096592	86,37873293	4385,905391	814,1307908	846,0761527	341,381718	216,7309149	268,3844036	221,0738435	1822,305972	36,67117383	515,5862999	250,3774667	279,8914443	392,6957818	500,5657673	757,8879709	255,6718967	142,4540647	190,3988046	1082,392533	1454,546911	209,8927503	496,7997247	1037,519366
C1QB	470,7657359	2,257623595	0,348244326	6,482872591	9,00E-11	5,26E-09	115,2131913	85,44931266	102,0839571	414,1078136	478,3274734	345,4726769	663,3809575	741,8232833	593,0429564	358,7532834	369,628038	124,4200541	471,3074183	414,603547	388,6434184	170,3759476	488,7877493	833,1392588	719,0772094	424,4148685	442,5485729	633,7324156	1016,272103	277,2830647	358,4055156	1209,615048
JAK1	1218,352906	0,668765939	0,103187801	6,481056209	9,11E-11	5,26E-09	823,6669766	947,9533123	808,8190447	1326,264214	1409,861256	1156,16354	1488,074155	1513,824289	1244,091574	1360,272866	1514,282607	791,0496069	1394,409527	1029,105233	1397,752645	1165,101374	1224,913878	1252,396434	1482,098026	1145,527168	1350,80233	1088,000784	1366,175424	1107,026312	1085,862255	1203,680714
CD163	591,8920524	2,799470193	0,434821714	6,438202376	1,21E-10	6,40E-09	77,28586748	133,514551	67,53246392	277,004551	1109,945316	407,4100095	930,984209	818,6393037	290,0283071	248,2163617	832,6567093	91,67793458	354,9815427	438,8336244	910,9256262	1222,759088	291,5059469	654,6862046	1169,499184	280,978362	607,2178094	418,7494427	1713,690327	690,7507233	450,6683217	899,0515767
IFITM3	7443,955518	1,924024489	0,309286487	6,220848861	4,94E-10	2,42E-08	1927,853028	2898,600903	2275,686982	11029,81758	11913,07128	6463,504745	10296,47277	9094,468136	4422,282367	6327,353688	22344,61107	2501,497929	8192,343595	5233,696723	4855,997238	10758,61777	9622,640751	9358,035158	7318,608042	6099,981293	4832,012907	5248,388663	11275,72409	4341,059424	8544,955264	6365,562098
C1S	791,6211673	1,903532697	0,307421255	6,191935866	5,94E-10	2,52E-08	314,1527391	283,0508482	212,0205263	1452,175373	884,880192	689,5689687	1244,230071	868,569717	579,1908582	770,6114937	816,7587291	178,11713	1280,33512	658,2504366	853,9927746	453,4701289	806,7942368	1531,901218	871,8811164	506,9399818	566,0505003	997,3340522	1247,948705	571,4137081	464,8625995	1477,649126
FPR1	98,5480353	2,41332899	0,38974856	6,192015148	5,94E-10	2,52E-08	21,46829652	18,69203714	26,69888109	165,0835203	107,6621323	101,8525024	72,52798404	124,5516903	58,00566143	54,67840614	168,9160389	15,71621736	93,81118994	61,92130897	39,5461724	149,598393	108,946667	153,727631	107,8615814	54,03430038	154,3774092	140,2062866	235,2592295	70,19824424	131,2970701	125,6100663
SOD2	13201,66001	2,827645902	0,459990319	6,147185681	7,89E-10	3,13E-08	1984,386209	2220,346984	2299,244818	32161,90719	15303,91577	12075,02707	18293,93349	10453,01433	4753,866969	5766,014943	38161,11405	2475,304234	12667,5126	4819,766234	4666,448343	24224,03146	26288,5363	17083,11737	8456,148239	6828,953127	21934,45689	15504,94588	21514,87451	6070,744161	18459,65834	8775,890693
BCL6	245,0171547	2,143168356	0,353227338	6,067390965	1,30E-09	4,86E-08	56,53318084	109,4819318	45,54515009	317,5759246	263,0032089	213,3397009	271,9799402	311,1048829	160,164886	168,7556563	786,9500164	57,62613031	186,1214008	162,2069072	232,8451357	306,9883691	247,3383792	210,7036061	157,7974987	196,488365	267,5875092	272,0001961	318,8538796	227,4423113	610,3539474	211,6579069
TIMP1	2034,724502	2,549984563	0,421288089	6,052828526	1,42E-09	5,02E-08	510,2298474	473,9766562	414,617918	1944,627909	2518,781219	1350,233849	3128,707174	4994,138702	1005,142879	2215,065503	3555,185806	324,8018254	3227,855424	799,5925549	1262,068364	4203,818732	1395,69514	1900,632528	2172,212403	1496,2589	1685,286717	1915,217875	5992,542201	573,5196554	1823,964703	2018,662561
STAT3	2548,495934	1,082356742	0,179122602	6,042547006	1,52E-09	5,07E-08	1283,804132	1464,654625	1322,379875	2960,311262	3060,167941	2447,212827	2411,555469	2679,781742	2060,499615	2231,193666	4519,000852	1216,69716	2860,115559	2362,432549	2290,268846	3316,617151	4372,589203	2648,845334	2426,885582	2810,766062	2212,742865	2685,417743	3254,220307	2360,064971	2384,638678	2618,030279
S100A8	369,390906	4,079624626	0,680763168	5,992722312	2,06E-09	6,55E-08	25,76195583	38,7192198	56,53880701	1115,013268	471,1499979	159,6606794	308,8691734	342,9286628	201,7211808	105,0297442	1873,974408	11,78716302	115,575386	145,3804645	74,31953088	1091,860494	176,6702708	169,8529069	334,570646	148,3487156	1268,467712	262,6531103	185,1024395	61,77445493	603,2568085	255,1763551
C3	635,4167657	3,77838107	0,63733249	5,928430022	3,06E-09	8,94E-08	85,15757621	85,44931266	47,1156725	2669,316582	2291,15271	323,4505143	1179,830223	239,2270351	152,3730808	75,92037686	900,2231248	41,90991295	1203,0347	367,4895076	92,0471254	300,2356638	556,5113531	1525,451107	966,7593593	191,5761559	596,9259821	588,8664038	388,1180182	251,3097144	440,0226133	961,362082
CD24	13456,69013	1,44600384	0,243991776	5,926444995	3,10E-09	8,94E-08	7259,146664	7378,01409	6085,774365	18226,33985	22313,23325	8122,048871	18710,9694	23225,87247	15882,2964	21696,31288	22219,41448	4555,083664	21072,99522	7334,982882	14699,58501	12628,07824	12888,09626	12291,76037	15341,31252	10410,93602	12957,41054	11859,58243	13747,73731	7537,887466	14471,06627	6958,006427
COL4A1	2478,362284	1,67180927	0,291876101	5,727804579	1,02E-08	2,81E-07	996,8445685	1061,440681	972,153376	3224,724697	3367,774033	1534,669461	2348,406104	4177,694141	2111,579227	2696,550173	4447,459941	681,0360854	5215,151671	1488,803646	2447,43079	3885,922147	2735,444693	2547,793604	2692,544662	2587,751768	1783,059076	2218,998163	3776,089765	1165,290854	2366,895831	1905,910218
ITGB6	702,5130209	3,227246338	0,56451384	5,716859554	1,09E-08	2,87E-07	99,46977389	77,4384396	75,38507601	608,5706045	200,4566368	151,4023684	533,9560205	2355,508399	181,8087896	839,0578438	1677,236904	94,29730414	1308,103233	318,356295	248,5272386	1462,220404	954,0194625	605,2353583	859,8964962	973,5998488	697,2712981	890,7772744	1275,415518	87,04582285	1171,027922	519,254211
SLPI	469,4819978	4,365501658	0,772778015	5,649101774	1,61E-08	4,10E-07	27,90878548	18,69203714	36,12201559	152,4924043	575,7360693	90,84142103	226,3373295	1772,804015	104,756493	629,7850951	753,1668086	23,57432603	184,6204218	59,22907814	85,22881982	2187,357059	61,83459479	570,8347696	238,6936848	1379,348323	457,9863139	347,7115908	710,5545257	33,69515723	1234,902173	242,3186318
ADAMTS1	2104,56687	2,661165169	0,476948638	5,579563409	2,41E-08	5,89E-07	415,0537328	526,0473311	295,2582144	4918,9293	610,5980931	1384,643478	1269,864962	4134,896644	802,5559425	1392,529192	3885,068894	276,3434885	2857,113601	905,2626148	784,4460576	4874,933746	2243,71244	3358,357477	1450,139039	3199,813025	3568,691108	3497,679497	1823,557581	647,9297943	3200,809655	2394,503704
IFITM2	2243,61108	1,680737124	0,305622617	5,499387257	3,81E-08	8,64E-07	817,2264876	1045,418935	802,536955	4741,254663	2672,584265	1848,485279	1992,018596	3077,578991	1866,570239	1534,5357	4731,636336	699,3716724	2060,093731	1305,73195	1270,591246	2762,895322	2243,71244	2953,07554	2195,182925	1221,175189	1862,820737	2756,455595	4238,248759	1698,095528	3243,392488	2693,198507
NNMT	450,6732091	2,85860842	0,519443558	5,503212771	3,73E-08	8,64E-07	55,81757096	73,43300307	45,54515009	679,9202616	547,0261674	262,8895669	286,98573	370,9116416	128,997665	129,0253037	745,2178186	68,10360854	284,4355279	201,2442541	146,5935701	1263,275319	288,5614424	1164,244925	450,4219742	138,5242974	1116,66326	667,3819244	769,0707807	224,6343816	823,2681152	785,3101781
IFITM1	2535,793531	1,529745617	0,279015657	5,482651532	4,19E-08	9,17E-07	815,079658	1209,641832	1056,961587	2963,109288	3423,14313	2155,419172	3129,957656	3105,013284	1642,339399	2362,972559	5816,67348	999,2894869	2735,534299	1745,238632	1639,120663	3839,172649	2470,439287	3810,940222	2483,812527	1916,744001	1788,204989	2340,510278	4598,899964	1719,856984	3282,426752	2880,130023
SERPING1	1680,001518	1,267485735	0,234914558	5,395518038	6,83E-08	1,45E-06	837,9791743	893,2123464	777,4085963	1985,199282	2636,696887	1299,307598	1891,354756	2388,978236	1202,535279	1404,723657	2833,814958	686,2748246	2292,745482	1305,058892	1390,593424	1968,673297	1045,299102	2616,594782	2152,238036	1511,977969	1631,254623	1719,863783	2137,634623	1157,569047	1589,759119	2323,291698
C9	89,03324482	2,04753766	0,382408418	5,354321614	8,59E-08	1,76E-06	20,75268664	22,69747368	29,83992592	214,0489712	118,4283455	206,4577751	84,40756764	60,35544465	68,39473512	55,85851562	133,1455836	30,12274993	34,5225179	120,4773294	56,93285164	138,6901769	164,8922528	63,42608551	67,91284755	55,99918404	128,647841	87,86260629	71,65255721	90,55573506	95,81137547	92,97123015
HAVCR1	483,2155447	2,301288656	0,430864648	5,341094157	9,24E-08	1,83E-06	107,3414826	173,5689163	50,25671734	519,0337799	200,9693136	315,1922033	859,7067074	339,0878617	275,3104528	676,5961047	166,9287914	48,45833685	1009,408404	450,9486631	404,3255212	759,9390591	488,7877493	1038,467773	945,786274	431,2919613	1000,880203	702,9008503	408,4195761	204,2768907	691,9710451	293,7495251
CFB	1310,349105	2,515502017	0,471676869	5,33310447	9,65E-08	1,86E-06	254,7571187	371,1704519	288,9761247	2639,937312	2831,001402	1224,982799	1898,857651	727,5574509	883,9370197	394,5499378	2376,748029	206,9301952	3425,984657	1127,371658	585,6924498	737,0837491	2588,219468	2670,345702	1711,803246	453,8881232	728,1467799	1731,080286	1197,791915	801,6639492	507,445433	1703,153812
FCER1G	212,0628696	1,702414444	0,321553092	5,294349481	1,19E-07	2,17E-06	70,12976864	61,41669347	83,23768809	190,2657522	177,8988567	147,2732129	243,218843	274,8916161	227,693865	136,8927003	329,8830877	62,86486943	324,961962	153,457157	115,911195	141,8068101	341,5625236	404,2069178	226,7090646	149,3311574	285,598207	235,5465615	372,5932975	153,7341549	170,3313342	432,2173146
IFNGR2	701,8728933	0,831554854	0,157079913	5,293833187	1,20E-07	2,17E-06	437,2376392	480,6523837	496,2850837	1036,668547	836,6885709	723,9785979	783,4272759	764,8680894	583,5196389	600,6757278	1041,317698	353,6148905	809,0277021	672,3846485	656,6028279	529,827642	921,6299128	860,0147187	681,1259122	675,9199757	627,8014639	899,1896516	724,8850371	694,2606355	610,3539474	746,7370081
SOST	65,29907887	-2,197292538	0,42766302	-5,13790633	2,78E-07	4,90E-06	144,5531966	161,5526067	345,5149317	68,55163131	17,94368871	50,92625118	26,26013215	25,23954958	45,88507546	13,76794399	41,73219784	75,96171722	46,53035021	41,05652008	30,34145986	20,25811573	47,11207222	49,45084633	16,97821189	41,26255666	87,48053186	33,64950879	31,04944146	91,25771751	102,9085144	40,55128124
PSMB8	626,9822325	1,092033112	0,214095475	5,100682833	3,38E-07	5,81E-06	337,7678653	315,0943404	376,92538	1088,432024	662,378452	527,155519	859,7067074	798,3379269	470,1055844	534,9829665	745,2178186	289,4403363	806,025744	638,7317632	450,6899992	589,5631115	945,1859489	880,4400683	674,1348838	406,7309156	733,2926935	781,4163708	722,4966185	517,36106	510,9940025	638,9299434
IL1R1	406,4109519	1,291918752	0,264881872	4,877339257	1,08E-06	1,80E-05	214,6829652	172,2337708	243,4309746	896,7672586	411,16681	350,9782176	458,9270715	642,5111426	278,7734773	389,0427602	472,9649088	149,3040649	544,1049017	319,7024104	420,3485393	302,8328582	450,5091906	523,53396	462,4065944	196,488365	290,7441206	564,5639808	582,774132	342,5674319	489,7025857	395,622256
LYVE1	555,2579124	1,601920505	0,332327384	4,820308466	1,43E-06	2,33E-05	210,3893059	188,255517	155,4817193	411,3097879	417,3189318	350,9782176	962,2462711	582,1556979	670,9610091	586,514414	488,862889	214,7883039	463,0520336	432,7761051	773,877684	846,6853495	309,1729739	683,7117014	792,982367	297,679873	429,6837888	625,3200384	1085,536242	516,6590776	415,182627	1525,123797
SOCS3	512,2862597	2,406726005	0,502529226	4,789225936	1,67E-06	2,66E-05	160,296614	130,84426	73,81455359	1369,633613	179,4368871	506,5097415	210,7062985	840,5867382	257,1295738	257,6572375	816,7587291	72,03266288	633,4131545	220,0898699	111,4792963	821,752284	718,4591014	1067,49327	363,5334781	713,2527651	450,2674434	1225,402945	771,4591993	160,0519969	606,805378	580,5756606
MUC1	1881,825791	1,016981882	0,21262941	4,78288436	1,73E-06	2,68E-05	915,2650417	1189,61465	1107,218304	1380,825716	2132,735573	1793,429873	2392,172991	2670,454083	1692,553255	2583,259663	2291,296386	931,1858784	1915,999743	1646,972207	1513,66384	2790,425581	1563,531897	1711,42929	2229,139349	2724,311181	2210,169908	1889,046035	3125,245704	1230,575221	1916,227509	1380,721673
MAPK13	338,8392017	0,788828454	0,170150475	4,636063784	3,55E-06	5,37E-05	218,9766245	225,6395912	196,3153021	519,0337799	426,5471146	396,3989281	380,7719162	381,8853589	303,8804054	297,78096	423,2837209	223,9560973	399,2604244	407,1999122	325,2331764	343,8685285	394,5636048	339,7058139	337,5668011	201,4005742	373,0787388	308,4538306	306,9117867	250,6077319	450,6683217	376,8301988
TNFRSF1A	1410,809898	0,938783478	0,203118064	4,621861087	3,80E-06	5,62E-05	815,079658	837,136235	755,4212825	1863,485161	1410,88661	1961,348863	1056,032457	1504,496629	798,2271618	948,0212862	2476,110405	753,0687483	1383,902674	1581,685609	1324,796775	1765,053262	1687,201086	1687,778886	1349,268486	1410,786461	1255,602928	1553,485656	1862,966487	1622,281424	1366,199243	1650,733863
FCGR3A/B	178,6422587	2,189096185	0,475955277	4,599373699	4,24E-06	6,12E-05	50,80830177	30,70834674	37,692538	429,4969554	201,4819904	154,1551387	93,78618626	228,8020038	81,38107723	68,44635013	156,9925538	44,52928251	191,3748275	80,76692474	61,70566555	472,6893669	173,7257663	356,9061083	232,7013747	90,38464792	156,950366	283,216699	123,0035565	105,9993488	248,3998623	488,5934861
EHD3	264,8249564	-1,178697217	0,256823266	-4,589526631	4,44E-06	6,27E-05	384,2825078	527,3824766	862,2168068	146,8963528	292,7384644	287,6644999	228,2130532	246,3599513	283,102258	132,1722623	210,6482367	331,3502493	207,1351074	253,0696975	221,2540162	150,1178319	120,7246851	366,5812739	200,7423876	239,7158053	136,3667114	203,7664699	208,9866252	275,1771174	156,1370563	212,6469626
ABCB1	868,3131278	-0,838579014	0,184883899	-4,535706019	5,74E-06	7,76E-05	1561,460767	1443,292297	1396,194429	818,4225371	646,9981473	762,5173826	937,8618626	859,7907433	778,3147705	697,4447056	707,4601158	1198,361573	1064,194139	856,1294022	766,0366325	477,3643167	768,5156781	852,4895899	1058,641447	870,4434571	753,8763481	653,3612957	501,5679005	750,4192309	461,31403	933,6685241
MME	1727,709227	-1,925092354	0,42415974	-4,538602254	5,66E-06	7,76E-05	3648,179189	4285,817088	5927,151601	1180,766874	1245,804674	3017,036286	907,850283	813,1524451	834,5889197	722,6203746	973,7512829	4526,270599	457,7986069	1821,967211	1369,456677	620,7294434	532,955317	970,7416138	1223,429974	2459,051888	895,3889731	964,619252	474,1010869	2657,705527	482,6054468	1906,899274
ASB15	109,0358882	-2,346192592	0,518304977	-4,526664215	5,99E-06	7,77E-05	264,0600472	218,9638637	321,9570954	83,94077303	51,78035886	145,8968277	23,13392595	30,72640818	52,81112458	29,89610695	33,78320777	461,0090425	19,51272751	158,1685609	42,61440991	23,89418778	50,05657673	26,87545996	40,9474522	247,5753399	82,33461822	49,53955461	46,57416219	65,98634958	173,8799036	89,01500759
GBP2	328,4788627	1,678099009	0,370451377	4,52987656	5,90E-06	7,77E-05	126,6629495	101,4710588	138,2059727	671,5261843	534,2092469	191,3175382	302,616761	327,5654587	225,9623527	265,9180039	683,6131456	113,9425758	330,9658781	160,8607918	111,4792963	465,4172228	391,6191003	490,2083897	232,7013747	232,8387126	339,6303002	499,1343804	502,7621097	160,0519969	592,6111001	347,1585296
LCN2	214,3028376	3,123500001	0,69099488	4,520293986	6,18E-06	7,84E-05	24,33073606	29,37320123	26,69888109	226,6400872	305,555385	41,29155501	135,6773495	253,4928675	27,7041965	68,0529803	707,4601158	26,19369559	661,9317562	40,38346237	29,31871402	566,7078014	276,7834243	491,2834081	264,6603618	64,84116046	355,0680411	305,6497049	303,3291589	30,88722746	241,3027234	67,25578351
IFNAR2	511,4159443	1,338944878	0,297412532	4,501978677	6,73E-06	8,38E-05	221,1234542	260,3533745	245,001497	839,4077303	436,2879742	415,6683205	515,1987832	604,6518182	356,69153	282,0461669	826,6949667	154,542804	677,6920361	310,9526602	366,8248405	864,8657098	803,8497323	896,5653443	393,4950285	478,4491689	514,5913639	607,5605754	739,2155485	287,1108189	574,8682528	623,1050531
KRT8	3776,233042	1,053179371	0,234538361	4,49043545	7,11E-06	8,68E-05	1932,146687	2355,19668	1718,151524	2863,779373	3954,276316	3621,269375	4120,339783	5363,404286	2256,160503	3927,010996	6553,942308	1901,6623	3960,333195	3415,767859	3021,873035	7036,318862	5135,215872	3405,658286	5897,431835	3619,315684	4137,314566	3267,741187	4824,605519	3088,722746	3584,055156	3220,365164
LTBR	720,1139494	0,743521303	0,167427908	4,440844481	8,96E-06	0,000107359	383,5668979	578,118006	381,6369473	566,600218	621,3643063	875,3809663	962,8715123	691,89287	798,2271618	797,7540118	1094,973381	514,7061184	548,6078388	835,2646134	718,990324	680,4649128	959,9084715	763,2630629	907,8349768	588,4826533	800,1895708	690,7496388	667,5629913	740,5914767	713,2624618	840,6972939
HDAC6	451,3220182	-1,064365545	0,240318749	-4,428974227	9,47E-06	0,000111339	589,6625445	842,476817	719,2992669	242,0292289	411,16681	540,9193707	303,2420023	313,8483122	371,4093844	330,037286	413,3474834	977,0248457	249,91301	568,7337617	440,4625408	319,9743407	359,2295507	341,8558507	370,5245065	624,8330008	324,1925592	330,8868364	310,4944146	489,9837448	255,4970013	693,3280036
COL1A1	502,7082268	2,017132499	0,458825712	4,39629351	1,10E-05	0,000127133	185,34296	170,8986253	124,0712709	352,5512467	220,9637096	214,7160861	559,5909114	1460,60176	203,4526931	680,1364331	802,8479965	92,98761936	1133,239175	278,6458904	289,4370721	389,0597097	600,6789208	719,1873085	477,3873696	850,7946206	257,2956819	369,2098881	1190,626659	202,8729258	589,0625307	654,7548336
NR4A1	373,9864693	-1,907967058	0,441445468	-4,322090036	1,55E-05	0,000175258	752,1059882	1536,752482	566,9585925	246,2262676	65,62263301	311,0630478	181,9452014	215,6335431	135,057958	161,6749994	218,5972268	443,9831403	217,6419607	245,6660628	153,0709604	192,7118188	332,7290101	546,1093464	202,7398243	225,9616198	506,8724934	342,1033394	372,5932975	287,1108189	149,0399174	1113,676651
COL3A1	2419,961613	1,711454612	0,397010366	4,31085623	1,63E-05	0,000181168	1268,060715	946,6181668	793,1138205	2968,70534	3136,556787	1102,484519	3935,268376	6852,537711	1440,618218	2563,591171	3217,353729	670,5586072	6266,587488	1247,848987	1654,120935	1783,753062	2087,6537	2511,242979	2336,002212	2275,335267	970,0047209	2397,527501	3617,25993	874,6701232	3168,872529	2832,655353
PSME2	1240,806621	0,714333332	0,166234505	4,297142355	1,73E-05	0,000189421	714,1786643	871,8500182	818,2421792	1919,445677	1389,86686	1544,304157	1340,517222	1383,78574	991,2907811	1056,591359	1480,4994	876,1791176	1169,262671	1218,234448	928,994136	1320,413594	1893,316402	1342,69798	1181,483804	1027,634149	1245,311101	1436,647084	1545,306817	1260,760466	975,856602	1328,301724
MEGF11	115,7089045	2,355304231	0,549854927	4,283501186	1,84E-05	0,000198007	12,88097791	20,02718265	32,98097075	221,0440357	112,2762237	174,8009162	84,40756764	103,1529418	26,83844036	22,02871039	158,9798013	28,81306515	99,8151061	88,17055951	18,40942508	279,9775481	209,0598205	357,9811267	87,88721448	106,1037171	151,8044523	113,0997379	191,0734859	42,82092898	198,7198899	65,27767223
ICAM1	393,4998963	1,651639971	0,389348213	4,242063833	2,21E-05	0,000234393	165,3058832	101,4710588	114,6481364	692,5113775	154,8283998	428,055787	357,6379903	343,4773486	235,4856703	269,0649626	467,0031663	123,1103693	451,7946908	257,7811015	176,5941147	471,6504892	1004,076039	757,8879709	337,5668011	241,680689	391,0894365	679,5331359	924,317988	274,475135	259,0455707	550,9039914
IMPDH1	197,3988601	0,875498281	0,20692663	4,230959936	2,33E-05	0,000242233	119,5068506	132,1794055	136,6354503	233,6351516	213,2735573	169,2953756	207,5800923	211,2440563	187,0033264	171,1158753	317,9596026	83,8198259	237,1546882	134,6115412	213,7538801	278,4192315	256,1718927	246,1792132	159,7949354	192,5585977	236,7120274	140,2062866	257,949206	159,3500144	266,1427096	170,1175701
C1QA	316,2600645	1,472872835	0,355483221	4,143297769	3,42E-05	0,000345064	120,9380704	105,4764953	92,66082259	183,2706878	276,845483	203,7050047	355,1370253	425,7802277	337,6448949	247,0362522	250,393187	129,6587932	404,513851	273,2614287	264,891172	105,4460895	335,6735146	699,8369774	379,5129716	365,468359	272,7334229	401,9246883	631,7367127	242,8859251	209,3655982	906,9640218
MAF	614,2484296	-1,13536863	0,273917542	-4,144928508	3,40E-05	0,000345064	1015,450426	917,2449656	1382,059727	440,6890584	508,062729	802,4325524	611,4859344	402,7354215	532,4400266	415,7919085	554,442057	1270,394236	279,1821013	695,9416682	434,6669811	286,7302533	453,4536951	612,7604871	566,2733024	692,6214868	617,5096367	396,3164369	321,2422982	732,8696698	223,5598761	804,1022352
PSMB10	255,4551647	1,066892875	0,261512453	4,079701983	4,51E-05	0,000447412	119,5068506	136,1848421	149,1996296	425,2999167	310,6821532	218,8452416	223,8363646	307,2640818	198,2581562	199,438503	359,6918004	128,3491084	364,7379065	218,0706968	147,6163159	170,3759476	512,3437854	332,1806851	267,6565168	185,681505	270,160466	227,1341843	378,5643439	225,336364	269,6912791	295,7276363
RELB	130,298715	1,319360644	0,326322095	4,043123846	5,27E-05	0,000515266	47,23025235	54,74096592	86,37873293	240,630216	99,97197998	114,2399689	128,1744546	173,9334178	101,2934685	111,7170312	125,1965935	40,60022817	206,3846179	90,18973263	62,38749611	169,8565088	209,0598205	240,8041212	92,88080621	101,191508	133,7937546	216,85239	191,0734859	85,64185797	99,35994493	164,1832362
FCGR1A	90,49201702	1,463168629	0,366643089	3,990716512	6,59E-05	0,000624328	38,64293374	33,37863776	25,12835867	125,9111595	75,3634926	101,8525024	66,27557163	120,7108893	66,66322284	41,30383197	95,38788078	35,36148905	161,3552467	54,5176742	78,75142951	59,21603058	182,5592799	217,1537165	71,90772094	49,12209126	105,4912296	162,6392925	140,9166958	84,93987553	60,32568085	97,91650835
JAK3	108,9622492	1,414784913	0,354359484	3,992513188	6,54E-05	0,000624328	32,91805467	53,40582041	26,69888109	160,8864816	50,2423284	90,84142103	80,65612019	97,66608315	77,91805267	49,9579682	111,2858609	37,98085861	167,3591629	61,24825126	71,25129337	139,2096158	167,8367573	212,8536429	90,88336952	99,22662434	95,19940232	154,2269153	274,668136	87,04582285	195,1713204	146,3802347
STAT1	893,8442179	1,400470793	0,352918896	3,968251091	7,24E-05	0,000676107	341,3459147	352,4784147	438,1757543	2810,616884	491,6570708	751,5063012	849,0776063	1278,986741	973,9756583	718,2933065	870,4144121	425,6475534	1204,535679	521,6197223	560,8056344	677,3482796	1563,531897	1359,898274	839,9221293	644,4818373	1065,204123	1259,987163	1458,129539	583,3474096	574,8682528	624,0941088
IL4R	330,1771735	0,929104048	0,235020437	3,953290436	7,71E-05	0,000709394	143,8375867	213,6232817	199,4563469	432,2949811	397,3245358	305,5575071	281,3585588	343,4773486	234,6199141	282,8329066	498,7991266	182,0461844	336,2193048	232,2049086	376,029553	484,1170219	491,7322538	450,4327089	264,6603618	199,4356905	319,0466456	331,821545	577,9972948	388,1962906	315,8226821	301,6619702
NFKBIZ	496,7975097	1,442083478	0,375886903	3,836482375	0,000124809	0,001132197	213,2517455	197,6015355	186,8921676	444,8860971	279,4088671	389,5170023	330,7526169	728,6548226	389,5902633	361,5068722	816,7587291	144,0653258	891,5815492	270,5691979	193,9807939	1184,320612	588,9009028	889,0402155	487,374553	599,2895134	599,4989389	549,6086436	838,3349193	154,4361373	628,0967947	558,8164365
TGFB1	417,6997695	1,026377366	0,269089864	3,814255024	0,000136594	0,001221654	275,5098054	176,2392074	207,308959	334,3640792	246,5975506	315,1922033	518,3249894	548,1371746	451,9247055	481,4846699	592,1997598	212,1689343	485,5667191	250,3774667	317,392125	552,6829521	553,5668486	538,5842176	344,5578295	400,8362647	468,2781411	290,6943676	927,9006159	329,9317479	500,3482941	540,0243794
SOCS1	147,4283865	1,408905305	0,378040491	3,726863493	0,000193877	0,001709891	60,82684015	89,45474919	51,82723976	456,0782001	111,7635468	83,95949519	75,02894901	161,8623288	96,09893162	142,3998778	166,9287914	45,83896729	143,3434982	98,93948281	105,3428213	247,7723385	170,7812618	118,2520238	145,8128786	113,9632517	205,8365456	270,1307789	140,9166958	127,0588221	298,0798348	104,8398978
XBP1	1311,733384	0,82434003	0,22169248	3,718394187	0,000200493	0,001744017	814,3640481	779,724978	901,4798673	2432,883405	1980,983234	1900,787916	1194,836013	1133,584988	989,5592689	843,3849119	1559,9893	793,6689765	1321,612044	1356,211278	1165,248425	1116,27412	1893,316402	953,5413194	1513,058295	1096,405077	982,869505	1528,248524	1482,013725	1220,045485	1387,49066	1763,486206
MS4A4A	250,0613389	1,224279574	0,330434823	3,705056147	0,000211344	0,001813559	83,01074655	129,5091145	83,23768809	113,3200436	179,4368871	125,2510502	375,1447451	304,5206525	220,7678159	123,5181261	200,7119991	100,845728	313,7046192	206,6287158	252,2773067	203,1005961	291,5059469	629,9607815	400,4860569	167,0151103	275,3063797	248,6324816	336,7670189	218,3165396	354,8569462	563,7617147
CD82	162,3808913	0,827474538	0,225298439	3,672793045	0,000239914	0,002004543	98,03855412	98,80076776	119,3597037	142,6993142	155,3410766	166,5426052	126,2987308	168,995245	247,6062563	295,0273712	145,0690687	89,05856502	156,1018201	133,2654258	188,1852342	165,181559	250,2828837	144,0524654	150,8064703	204,3478996	187,8258478	107,4914864	248,3955317	138,2905411	145,4913479	149,3474016
DNMT1	194,1684909	0,654700822	0,178213566	3,673686776	0,000239076	0,002004543	121,6536803	121,4982414	136,6354503	160,8864816	174,3101189	154,1551387	221,9606408	239,775721	174,0169843	230,5147194	210,6482367	129,6587932	251,413989	168,9374842	179,3214369	202,0617184	220,8378385	232,2039741	187,7590491	135,5769719	303,6089047	189,7458412	216,1518809	162,1579442	269,6912791	253,1982438
BAX	641,1304761	0,537741054	0,146835086	3,662210909	0,000250048	0,002062083	495,2020398	495,3389843	431,8936646	730,2847254	551,1275819	572,5762295	749,0390076	714,3889903	733,2954512	545,210582	796,886254	436,1250316	584,6313357	545,176742	645,6935389	673,1927687	912,7963993	815,9389644	585,248951	626,7978845	838,7839231	602,8870325	663,9803635	534,2086386	567,7711139	820,9161811
ALOX5	164,6202907	1,265020897	0,347425875	3,64112459	0,00027145	0,002209879	64,40488957	68,09242103	64,39141909	160,8864816	164,0565825	115,616354	156,3103104	178,3229046	118,6085913	123,5181261	170,9032864	72,03266288	198,1292332	79,42080933	115,5702797	304,9106136	161,9477483	426,7823042	181,766739	82,52511331	174,9610637	158,9004582	376,1759253	88,44978774	209,3655982	264,0778559
AHR	255,4884163	0,726971625	0,20313265	3,578802453	0,000345172	0,002774486	152,4249053	178,9094984	114,6481364	246,2262676	305,555385	233,9854784	332,6283406	327,0167728	281,3707457	149,4805348	286,1636423	200,3817713	249,1625205	176,341119	238,9816108	276,8609149	235,5603611	345,0809059	230,703938	229,8913871	337,0573433	230,8730186	348,7091117	247,0978197	351,3083767	336,2789176
CXCL16	214,1890456	0,975162871	0,273083526	3,570932617	0,000355712	0,002823467	108,7727024	125,503678	116,2186588	342,7581566	171,234058	180,3064569	167,5646528	298,4851081	184,406058	191,5711064	190,7757616	104,7747824	397,0089558	146,7265799	133,9797048	202,0617184	312,1174785	350,4559979	216,7218812	177,8219704	213,555416	263,5878189	366,6222511	132,6746816	220,0113066	253,1982438
CDKN1A	508,354303	1,23323719	0,345995912	3,564311451	0,000364813	0,002850022	254,0415088	365,8298698	147,6291072	297,9897443	523,4430336	682,6870429	459,5523127	585,4478131	225,0965966	389,0427602	1142,667322	176,8074453	253,6654576	382,2967771	604,7837054	674,7510853	527,066308	546,1093464	474,3912145	421,467543	517,1643207	420,6188599	1240,783449	573,5196554	851,6566708	478,7029297
VCAM1	1847,759273	1,513914729	0,425017657	3,562004319	0,000368034	0,002850022	966,7889534	670,2430462	848,0821051	6803,399654	1556,486827	2470,611375	2349,031345	1255,941935	1988,641855	1649,39969	1701,083874	691,5135637	2668,740732	1455,150761	684,8987961	1372,357481	1899,205411	3176,679367	2145,247008	684,7619521	2076,376153	2292,840141	1873,714371	765,8628446	500,3482941	3494,333576
STAT6	1320,126673	0,467328688	0,132045202	3,539156915	0,000401407	0,003071007	978,2387115	1058,77039	1044,397407	1229,732325	1462,666969	1245,628576	1380,532662	1325,076353	1423,303095	1337,457416	1975,324031	978,3345304	1349,380156	1218,234448	1235,817887	1832,060876	1475,196761	1530,826199	1598,948073	1266,367513	1253,029971	1105,760247	1230,035565	1118,960013	1181,673631	1487,539682
FCGR2A	224,3708435	1,174682404	0,33264171	3,531374355	0,000413406	0,003088388	99,46977389	110,8170774	109,9365692	384,7285431	138,4227415	323,4505143	150,6831393	253,4928675	226,8281089	100,3093062	151,0308112	86,43919546	234,9032196	212,0131774	99,88817683	239,9807555	217,893334	409,5820098	269,6539535	167,9975521	272,7334229	365,4710538	437,080599	197,9590487	170,3313342	402,5456454
HSD11B1	33,71446221	1,455880722	0,412140885	3,532483124	0,000411677	0,003088388	15,74341745	20,02718265	14,13470175	27,98025768	38,45076153	19,26939234	44,39212817	77,91339218	33,76448949	87,32810189	57,63017797	13,0968478	27,76811222	9,422807886	24,20498483	16,62204367	41,22306319	44,07575434	22,97052197	20,63127833	43,74026593	36,45363452	64,48730149	25,27136792	21,29141677	28,68261356
IL17RA	183,7312941	0,603583696	0,171460948	3,520240054	0,000431156	0,003183538	118,7912408	145,5308606	117,7891813	167,8815461	200,4566368	128,0038205	225,086847	201,3677108	173,1512281	178,1965322	222,5717218	120,4909997	201,8816808	155,4763301	163,2984188	263,3555044	170,7812618	185,9781829	201,741106	147,3662738	234,1390706	201,8970527	278,2507638	174,0916457	216,4627372	182,9752934
AGT	1403,318258	1,617651428	0,46199769	3,501427523	0,000462773	0,00337771	392,1542165	568,7719874	380,0664249	1660,628293	4449,009447	3146,416492	913,4774542	568,4385514	871,8164337	476,7642319	3147,800066	776,6430744	409,7672777	1470,631088	1165,58934	965,1174107	2122,987754	677,261591	751,0361965	1716,325869	1075,495951	2600,359263	1478,431097	1265,674344	1742,347606	1693,263256
CD59	8305,500609	0,660007991	0,189442831	3,483942825	0,000494085	0,003565273	5785,705913	6183,058858	6505,10385	11084,37908	9781,361056	8051,853228	7749,23995	11509,23461	7509,568765	8750,905201	13237,05571	5154,919293	7826,10471	5737,143887	6412,616403	11609,97807	9154,464533	6043,753436	8599,96368	10429,60242	9342,406211	7815,098417	10935,37444	5939,473445	8821,743682	5972,907009
APOL1	459,9702352	0,824157502	0,237485571	3,470347695	0,000519785	0,003708579	253,325899	218,9638637	381,6369473	503,6446382	386,5583226	417,0447056	468,9309313	590,9346717	388,7245072	496,8260932	484,8883939	237,0529451	514,8358104	301,5298524	343,3016862	527,7498866	503,5102719	653,6111862	364,5321964	345,8195225	473,4240548	467,3542888	955,3674295	378,3685364	674,2281978	627,0612757
SIGIRR	435,8916028	-0,790575069	0,228283709	-3,463125217	0,00053394	0,003767242	633,3147474	741,0057582	461,7335905	267,2114608	397,3245358	631,7607917	375,7699863	437,8513166	335,0476265	317,0560816	306,0361175	822,4820416	303,9482554	683,1535718	415,9166407	249,3306551	494,6767583	402,056881	365,5309148	383,1523118	419,3919616	437,4436143	285,4160195	487,175815	259,0455707	420,348647
IFI30	373,7930209	0,611863057	0,17690938	3,458624161	0,000542942	0,003788661	236,1512617	285,7211392	307,8223937	401,5166977	391,6850908	373,0003803	536,4569854	335,7957466	261,4583545	427,9863732	425,2709685	216,0979886	354,2310532	501,4279911	299,3236152	298,1579084	365,1185597	489,1333713	418,4629871	279,9959202	512,0184071	383,2305168	445,440064	346,0773441	411,6340576	415,4033688
IL18BP	180,4254099	1,467908542	0,426766075	3,439609261	0,000582555	0,004020893	75,85464771	78,77358511	86,37873293	677,1222358	294,7891717	308,3102774	154,4345867	97,66608315	96,96468776	110,1435519	280,2018998	78,58108678	212,388534	92,20890575	87,95614205	145,962321	394,5636048	130,0772262	100,870553	101,191508	108,0641864	242,0895216	272,2797174	179,7075052	166,7827647	117,6976211
LDLR	314,4300847	1,031733758	0,300405147	3,434474308	0,000593704	0,004053788	211,8205257	200,2718265	158,6227641	404,3147234	177,3861799	392,2697726	300,7410373	268,3073857	161,0306422	592,0215916	353,7300579	103,4650976	183,1194428	333,1635646	368,5294169	315,8188298	288,5614424	383,7815682	379,5129716	258,3822	200,6906319	313,1273735	636,5135499	274,475135	450,6683217	464,8561508
TNC	248,8884926	1,844096008	0,539707599	3,416842771	0,000633519	0,00427962	135,2502681	70,76271205	124,0712709	96,53188899	130,7325892	60,56094735	223,2111233	640,3163991	285,6995264	850,072199	357,7045529	39,29054339	646,9219658	70,67105915	184,4351661	208,8144236	197,2818024	304,2302068	188,7577675	84,48999697	218,7013296	172,9210868	567,2494112	73,70815645	269,6912791	269,0231341
CD276	231,6487744	0,801304798	0,235496743	3,402615198	0,000667442	0,004461324	148,8468559	144,1957151	153,9111968	242,0292289	180,9749176	147,2732129	313,8711034	340,7339193	261,4583545	239,1688556	292,1253849	130,968478	372,9932912	137,3037721	182,7305897	238,9418778	256,1718927	287,0299124	235,6975297	335,0126624	254,7227251	201,8970527	341,543856	155,8401022	188,0741815	239,3514649
LY96	73,49757696	1,239277367	0,366860857	3,378058314	0,000729996	0,004828619	37,92732386	24,03261919	43,97462767	103,5269534	36,40005425	59,18456219	73,15322529	75,16996288	64,06595441	40,91046214	81,47714816	17,02590214	138,8405611	51,15238567	58,63742804	84,66853495	64,7790993	188,1282197	64,91669251	60,91139316	72,04279094	101,883235	121,8093473	41,4169641	67,42281978	137,4787339
SLC12A3	3454,088477	-1,620879572	0,481804141	-3,364187712	0,000767693	0,00502562	6817,615366	7757,195415	6885,170275	4364,920198	5005,263798	3216,612136	2718,548919	480,6488137	3763,441944	1142,345981	623,9957201	10537,72374	3179,824094	3159,332873	3137,443315	1039,916607	3927,969021	3074,552619	2875,310119	2444,315261	1546,347048	2225,541123	1099,866753	5709,223204	471,9597384	2601,216333
MASP1	62,48602169	-1,120470646	0,33690761	-3,32575048	0,000881808	0,005713758	95,89172447	156,2120247	80,09664326	50,36446382	21,53242646	34,40962918	59,39791797	71,87784772	88,30712635	55,85851562	23,84697019	120,4909997	78,80139955	43,7487509	46,36447798	24,93306551	58,89009028	82,77641668	68,9115659	79,57778784	41,16730911	65,42960043	45,3799529	20,35749083	49,67997247	60,33239403
EGR1	319,3483108	-1,61329928	0,488988379	-3,299258941	0,000969404	0,006217897	462,2839851	1483,346662	342,3738869	187,4677264	48,70429794	247,7493301	146,3064506	262,8205271	98,69620004	192,7512159	369,628038	364,0923688	417,2721729	113,7467523	111,8202116	635,2737316	238,5048656	454,7327825	68,9115659	157,190692	337,0573433	402,8593969	349,903321	131,9726992	266,1427096	411,4471462
FKBP1A	878,0712967	0,644307536	0,197859828	3,256383783	0,001128411	0,007165411	440,8156886	696,9459564	537,1186666	773,6541248	621,3643063	883,6392773	842,1999527	1029,334674	870,0849214	726,1607031	1307,608866	628,6486943	866,0649056	793,5350356	615,3520791	866,4240264	1451,640725	1041,692828	818,949044	1042,370777	1155,257612	843,1071369	1312,436006	758,8430202	1078,765116	827,8395706
NFKB2	352,377475	0,797266712	0,245064146	3,25329806	0,001140738	0,007171965	226,1327234	205,6124086	226,155228	492,4525351	300,4286168	258,7604114	320,748757	409,3196519	316,8667475	306,0417264	437,1944536	185,9752387	497,5745515	238,262428	239,6634413	520,9971813	565,3448666	485,9083161	249,6795866	329,1180114	460,5592707	411,2717741	480,0721333	190,9392243	390,3426408	416,3924244
TLR4	339,032865	0,592808775	0,182759136	3,243661512	0,001180039	0,00734632	202,5175972	196,26639	285,8350799	363,7433498	358,8737743	231,2327081	391,4010173	406,5762226	365,3490914	330,037286	377,5770281	256,6982168	356,4825218	279,9920058	301,3691069	470,6116115	320,950992	421,4072122	310,6014057	339,9248715	321,6196024	283,216699	603,0756898	336,9515723	315,8226821	386,7207552
KITLG	667,6469048	0,908476883	0,282640142	3,214252849	0,001307845	0,008062925	405,0351944	412,5599627	453,8809785	853,3978592	578,2994534	591,8456219	731,5322529	1144,558705	426,8177774	581,7939761	1242,029698	335,2793036	759,4953938	317,0101796	543,4189552	1359,371509	524,1218035	654,6862046	791,9836487	767,2870655	797,616614	504,7426319	832,3638729	387,4943082	738,1024481	624,0941088
CTSS	344,5157581	1,176766878	0,369423871	3,185411041	0,001445485	0,0088258	216,8297949	126,8388235	144,4880623	285,3986283	279,4088671	192,6939234	423,2883207	347,8668355	232,0226457	224,614172	371,6152855	89,05856502	712,9650436	222,7821007	241,708933	400,4873647	371,0075687	932,0409514	313,5975608	262,3119673	301,0359479	404,7288141	566,0552019	157,2440671	319,3712516	817,9490142
SLAMF8	52,92150985	1,045905037	0,32887478	3,180253098	0,001471465	0,008898858	22,89951629	18,69203714	18,846269	50,36446382	45,11556019	28,90408851	50,01929934	34,56720921	39,82478247	25,96240867	81,47714816	34,05180427	69,0450358	34,99900072	53,86461413	33,76352621	70,66810833	91,37656387	54,92950905	32,42058023	95,19940232	62,62547469	108,6730451	58,96652516	99,35994493	59,34333839
MT1A	160,5339643	2,109393396	0,669585303	3,150298232	0,001631039	0,009770845	37,92732386	88,11960368	32,98097075	155,2904301	271,206038	83,95949519	56,27171176	138,8175227	38,09327019	70,41319927	1114,845857	37,98085861	42,02741309	51,15238567	232,5042205	216,6060066	76,55711736	40,85069914	95,87696125	38,31523118	138,9396682	79,45022909	433,4979711	320,8059762	202,2684593	79,12445119
VCAN	124,7449655	1,621877643	0,515979242	3,143300178	0,001670544	0,009913978	47,94586223	32,04349225	25,12835867	68,55163131	54,34374296	74,32479902	180,6947189	119,6135176	89,1728825	69,62645961	137,1200786	41,90991295	361,7359484	28,94148137	55,22827524	133,4957882	135,4472076	337,5557771	62,91925582	56,98162586	110,6371432	126,185658	345,1264839	50,54273585	347,7598073	150,3364573
MYD88	397,6299539	0,547735806	0,175252018	3,12541797	0,001775526	0,010439434	301,9873711	311,0889039	274,8414229	581,9893597	364,0005425	443,1960238	370,7680564	422,4881125	413,8314353	338,6914222	457,0669287	265,8660103	402,2623825	376,2392577	305,8010055	303,8717359	630,123966	511,7087576	374,5193799	410,6606829	380,7976093	402,8593969	543,3652255	331,3357128	326,4683905	492,5497087
APOE	12558,68799	-1,122508461	0,360621748	-3,112703182	0,001853824	0,010619178	15851,47454	19415,68601	23171,48774	7295,852189	12009,96719	16489,0943	10042,62483	5985,065365	9176,149336	5504,030638	17849,45719	32439,58231	5044,040061	17453,73244	11209,97621	11109,239	13636,0004	7446,652446	9095,32798	19989,74382	5009,546927	8468,459712	5366,776535	16560,4678	5081,551469	15823,90118
CXCL1/2	442,6808782	1,583308251	0,507975801	3,116897	0,001827654	0,010619178	207,5268664	126,8388235	124,0712709	867,387988	236,856691	228,4799377	371,3932976	495,463332	163,6279106	169,1490262	552,4548095	110,0135215	439,7868585	133,9384835	142,1616715	1006,67252	444,6201816	1291,097096	269,6539535	323,2233605	638,0932912	955,2721662	720,1081999	102,4894366	613,9025169	775,4196217
TLR2	148,537199	0,892029945	0,28661304	3,112314583	0,001856266	0,010619178	64,40488957	78,77358511	89,51977776	144,098327	156,879107	86,71226553	123,7977659	195,3321663	92,63590706	105,8164838	174,8777814	81,20045634	182,3689532	76,7285785	174,548623	203,620035	147,2252257	212,8536429	172,7782739	107,0861589	200,6906319	109,3609036	311,6886239	117,9330503	283,8855569	167,1504031
EVA1C	129,3320321	1,046297838	0,336999675	3,10474435	0,001904436	0,010701916	50,80830177	64,0869845	84,80821051	330,1670406	178,4115335	81,20672486	118,7958359	142,1096378	88,30712635	139,2529192	164,9415438	82,51014112	88,55776331	90,18973263	144,5480784	140,7679323	164,8922528	183,8281461	114,8526098	46,17476578	120,9289705	189,7458412	229,2881831	125,6548572	120,6513617	77,14633991
LILRB2	116,6219685	1,38109179	0,44467914	3,105816457	0,001897545	0,010701916	31,4868349	26,70291021	75,38507601	159,4874688	50,75500522	89,46503586	105,6657699	136,0740934	88,30712635	48,77785871	164,9415438	43,21959773	149,3474144	53,17155879	43,29624047	108,5627227	232,6158566	277,3547468	82,89362275	130,6647627	133,7937546	142,0757038	337,9612282	45,62885875	124,1999312	150,3364573

IRF7	121,6161479	0,746765712	0,241496828	3,092238178	0,001986534	0,011004266	67,2673291	105,4764953	50,25671734	102,1279405	99,97197998	122,4982799	116,9201122	134,9767217	148,9100562	88,11484154	158,9798013	73,34234766	104,3180432	92,88196345	105,001906	107,0044061	188,4482889	133,3022814	99,87183463	193,5410396	200,6906319	100,9485264	189,8792766	108,8072786	159,6856258	108,7961204
MAPK11	106,4257933	0,780728855	0,25255774	3,091288567	0,001992899	0,011004266	57,96440061	80,10873062	59,67985184	162,2854945	91,25647403	103,2288875	108,7919761	141,0122661	63,20019827	84,18114326	143,0818212	64,1745542	79,55188907	57,88296273	93,06987124	159,4677315	106,0021625	136,5273366	118,8474832	157,190692	128,647841	105,6220693	128,974603	117,2310679	141,9427785	77,14633991
LST1	69,01807535	1,07504188	0,349571629	3,075312155	0,002102823	0,011511145	34,34927444	26,70291021	31,41044834	86,7387988	49,21697476	45,42071051	74,40370777	73,5239053	55,40839301	39,33698283	61,604673	32,74211949	127,5832183	42,40263549	42,27349463	71,68256334	120,7246851	148,352539	46,93976228	71,71825324	74,61574776	80,38493767	99,11937081	27,37731525	120,6513617	109,785176
EMP3	250,5032514	0,849129234	0,276790839	3,067764947	0,002156662	0,011589673	138,8283175	128,173969	160,1932865	172,0785847	180,4622408	161,0370646	293,8633836	308,3614536	244,1432317	315,4826023	335,8448302	147,9943801	270,9267166	156,8224455	205,5719134	241,0196332	318,0064875	379,4814946	242,6885582	170,9448776	385,9435229	196,2888013	472,9068776	146,7143305	383,2455019	356,0600304
IL6R	250,421526	-0,81335737	0,265312578	-3,065657037	0,002171923	0,011589673	183,9117402	650,2158635	361,2201559	249,0242933	308,1187691	276,6534186	205,7043685	205,2085118	116,0113229	141,6131382	220,5844743	358,8536296	151,5988829	252,3966398	233,5269663	364,6460831	159,0032437	199,9534221	280,6398553	253,4699909	226,4202001	208,4400128	260,3376245	260,4354861	230,657015	152,3145685
NPDC1	468,2712283	0,586908476	0,191377653	3,066755541	0,002163958	0,011589673	267,6380966	301,7428853	362,7906783	376,3344658	433,2119132	476,2292678	442,0455579	502,0475623	395,6505563	382,3554731	576,3017797	316,9437167	475,8103554	349,3169495	466,372102	797,3386574	559,4558576	728,8624741	446,4271008	465,6774251	419,3919616	417,8147342	701,0008514	507,5333058	379,6969324	627,0612757
SLC11A1	46,32385981	1,684895891	0,558408506	3,017317738	0,002550223	0,013494933	13,5965878	13,3514551	21,98731384	22,38420614	108,1748091	38,53878468	26,8853734	66,39098911	25,10692808	16,12816296	111,2858609	15,71621736	22,51468559	17,49950036	43,97807103	112,1987948	23,55603611	32,25055195	64,91669251	35,36790571	74,61574776	17,75946297	130,1688123	44,92687631	92,26280601	12,85772332
RORC	245,4962628	-0,898530282	0,298529181	-3,009857461	0,002613703	0,013716542	400,0259252	315,0943404	433,464187	190,2657522	433,2119132	279,4061889	116,294871	182,7123915	139,3867387	164,4285882	210,6482367	450,5315642	104,3180432	189,1292154	203,5264217	241,5390721	288,5614424	207,4785509	278,6424186	198,4532487	156,950366	188,8111327	280,6391824	427,5073074	170,3313342	131,5444001
PVR	367,5969684	0,648032677	0,216457043	2,993816551	0,002755115	0,01434015	207,5268664	280,3805572	262,2772436	236,4331774	345,0315001	316,5685884	383,2728812	472,9672117	275,3104528	310,7621644	649,8299378	225,2657821	427,7790261	270,5691979	377,7341294	448,7951791	403,3971184	382,7065498	343,5591111	437,1866122	566,0505003	327,1480021	496,7910633	286,4088365	500,3482941	323,4211943
TNFRSF1B	147,5017839	0,847783814	0,286478514	2,95932774	0,00308311	0,01591687	72,99220818	68,09242103	100,5134347	128,7091853	109,2001627	137,6385167	182,5704426	189,8453077	113,4140544	105,0297442	164,9415438	89,05856502	175,6145476	100,9586559	128,5250603	91,94067906	229,6713521	319,2804643	143,8154419	160,1380175	118,3560137	150,488081	278,2507638	94,76762972	159,6856258	221,5484633
CD8B	27,26267712	-1,543635493	0,524010488	-2,945810298	0,003221099	0,016379321	44,36781281	34,71378327	109,9365692	74,14768285	11,27889005	9,63469617	13,13006608	7,681602046	15,58361053	10,62098536	19,87247516	49,76802163	23,26517511	9,422807886	5,795559748	9,349899566	58,89009028	31,17553355	24,96795866	8,841976427	30,87548183	36,45363452	26,27260431	14,74163129	14,19427785	13,84677896
MYC	215,5820761	1,283252791	0,436029761	2,943039458	0,00325007	0,016379321	135,2502681	133,514551	43,97462767	295,1917185	104,0733945	86,71226553	226,9625708	353,3536941	50,21385616	177,4097926	369,628038	53,69707597	243,9090939	98,93948281	140,1161798	341,790773	223,782343	394,5317522	271,6513902	181,7517377	367,9328252	260,7836931	425,1385061	86,34384041	280,3369875	258,143522
S100A12	51,22770893	1,865893415	0,633649582	2,944677103	0,003232919	0,016379321	20,03707675	21,36232817	12,56417933	204,255881	57,41980388	35,78601434	43,14164568	28,53166474	30,30146493	20,45523107	119,234851	14,40653258	18,01174847	24,90313513	10,56837366	63,89098036	41,22306319	36,55062555	31,95898708	31,43813841	241,857941	46,73542888	26,27260431	19,65550839	113,5542228	17,80300152
FJX1	134,7368717	1,217932514	0,414607596	2,937554748	0,003308118	0,016540588	57,24879073	44,05980184	50,25671734	190,2657522	53,83106614	77,07756936	101,9143224	154,7294126	87,44137021	161,6749994	149,0435637	75,96171722	328,7144096	76,0555208	60,00108915	93,49899566	250,2828837	285,954894	107,8615814	103,1563916	138,9396682	298,1720362	117,0325101	51,24471829	163,2341952	225,5046859
ACKR1	87,78988973	2,026223997	0,693617464	2,92124132	0,003486397	0,017207257	9,302928493	29,37320123	10,99365692	93,73386322	13,84227415	24,77493301	58,14743548	114,6753448	29,43570878	52,31818717	27,82146523	13,0968478	106,5695118	22,2109043	39,88708767	180,7647249	315,061983	116,101987	19,97436693	25,54348745	48,88617957	162,6392925	538,5883884	18,2515435	95,81137547	114,7304542
TGFBI	506,6409485	1,007890003	0,345118677	2,920415704	0,003495648	0,017207257	359,2361618	226,9747368	190,0332124	380,5315044	248,6482579	289,0408851	452,674659	891,0658373	683,9473512	271,8185514	474,9521564	214,7883039	1133,239175	269,2230825	511,3729189	375,5542992	765,5711736	809,488854	524,3271318	610,0963734	465,7051843	534,6533063	876,5496165	250,6077319	571,3196834	791,2445119
IL1R2	91,43166615	1,347678499	0,462619965	2,913143835	0,003578097	0,017239035	34,34927444	44,05980184	39,26306042	79,74373438	108,6874859	33,03324401	88,78425633	144,8530672	51,0796123	143,9733572	230,5207119	45,83896729	121,5793022	24,23007742	56,59193636	220,7615175	150,1697302	46,22579113	111,8564548	74,66557871	136,3667114	68,23372616	109,8672544	37,90705189	141,9427785	32,63883612
LAIR1	104,9368616	1,059726884	0,363833668	2,912668556	0,003583547	0,017239035	45,79903258	56,07611143	36,12201559	125,9111595	85,61702901	63,31371769	107,5414936	137,1714651	77,91805267	50,35133802	103,3368708	57,62613031	163,6067153	54,5176742	65,11481834	100,7711398	100,1131535	289,1799492	103,866708	78,59534601	79,7616614	142,0757038	247,2013224	68,79427935	127,7485006	160,2270137
TBK1	241,5970612	0,359059498	0,123265361	2,912898603	0,003580909	0,017239035	191,7834489	182,9149349	213,5910487	257,4183706	258,9017943	225,7271674	247,5955317	236,4836058	246,7405001	231,301459	304,04887	191,2139778	246,9110519	220,7629276	227,7314066	298,1579084	279,7279288	264,454526	199,7436693	310,4516168	272,7334229	212,1788471	267,5028802	208,4887854	255,4970013	229,4609085
APOL2	340,16328	0,548434047	0,19089951	2,872893948	0,004067306	0,018983819	197,508328	242,9964829	251,2835867	419,7038652	295,3018485	304,1811219	300,115796	356,6458093	354,9600177	273,7854005	278,2146523	233,1238908	312,2036401	345,2786033	274,4367998	276,8609149	524,1218035	362,2812003	383,507845	290,8027803	367,9328252	410,3370655	511,1215748	327,8258006	635,1939337	314,5196935
ARHGDIB	869,2148093	0,648543155	0,225918618	2,870693711	0,004095722	0,018983819	636,8927968	542,0690772	596,7985184	920,5504776	946,4014105	551,930452	1012,26557	1198,329919	780,9120389	747,009304	989,649263	544,8288684	1338,122813	574,1182234	684,2169655	1141,207186	1074,744148	1212,620753	910,8311319	714,2352069	823,3461822	731,8768162	1419,914842	567,9037959	798,4281289	1140,381153
CMKLR1	156,8766861	0,750975134	0,261200722	2,875088276	0,004039146	0,018983819	82,29513667	86,78445817	95,80186743	130,1081982	102,5353641	126,6274354	168,189894	173,3847319	134,1922018	106,6032235	129,1710886	86,43919546	207,8855969	115,7659255	225,0040843	168,2981922	135,4472076	360,1311635	136,8244134	158,1731339	149,2314955	124,3162408	322,4365074	190,2372419	138,394209	224,5156303
FGD2	94,38853964	1,000728168	0,348337935	2,872865877	0,004067668	0,018983819	47,23025235	70,76271205	32,98097075	79,74373438	96,89591905	46,79709568	120,6715597	130,0385489	46,7508316	66,47950098	135,1328311	40,60022817	94,56167946	71,34411685	117,2748561	218,1643232	53,00108125	136,5273366	109,8590181	77,61290419	77,18870458	83,1890634	217,3460902	72,30419156	120,6513617	90,99311887
KRT19	380,7668503	0,737063396	0,256393168	2,874738836	0,004043618	0,018983819	215,3985751	316,4294859	237,1488849	381,9305173	258,9017943	429,4321721	324,5002045	548,6858604	298,6858686	485,811738	331,8703352	187,2849235	337,7202838	275,9536595	531,8278357	751,1085984	273,8389198	295,6300596	484,378398	435,2217286	414,2460479	290,6943676	675,9224563	334,845625	429,3769049	353,0928634
ABCC2	458,7741206	-0,938422618	0,327387785	-2,866394725	0,004151762	0,019104123	652,6362143	672,9133372	665,9015047	247,6252804	213,7862341	703,3328204	298,8653136	258,4310403	259,7268422	219,1069944	321,9340976	711,1588354	206,3846179	381,6237194	383,1887739	490,8697272	344,5070281	508,4837025	571,2668941	1198,579027	684,406514	310,3232477	414,3906225	405,0438692	486,1540163	317,4868604
TAP2	258,0538836	0,729310316	0,256080437	2,847973566	0,004399858	0,02010007	130,9566088	153,5417337	221,4436608	456,0782001	251,211642	236,7382487	301,9915198	265,0152706	187,0033264	163,2484787	325,9085927	157,1621736	218,3924502	280,6650635	177,2759452	262,3166267	424,00865	328,9556299	172,7782739	187,6463886	393,6623934	359,8628023	355,8743675	223,2304167	209,3655982	265,0669115
HNF1A	163,8566699	-0,908664815	0,32246862	-2,817839497	0,004834797	0,021773732	272,6473658	248,3370649	229,2962729	68,55163131	174,3101189	253,2548707	126,2987308	117,4187741	115,1455667	87,72147171	206,6737417	320,872771	91,55972138	231,5318509	182,0487591	125,1847664	64,7790993	155,8776678	168,7834005	282,9432457	113,2101001	82,25435482	105,0904172	164,965874	81,61709762	189,8986829
TNFSF10	2923,665678	1,015808659	0,360300668	2,819336042	0,004812311	0,021773732	1851,28277	1750,375764	1911,325781	6975,478239	3004,798844	2271,035526	3951,524648	2626,559214	6056,829961	2918,410756	2748,363315	1447,201682	4984,751389	2870,591117	975,358614	2504,734206	4469,757852	4337,699238	3480,533437	2011,058416	2382,558015	3492,071246	1525,005259	1130,893715	1195,867909	3141,240712
CD58	304,6197646	0,46302726	0,164751307	2,810461841	0,004947045	0,022122351	243,3073606	240,3261919	240,2899298	283,9996154	394,7611517	273,9006483	334,5040643	294,0956212	242,4117194	339,084792	321,9340976	196,452717	281,4335698	277,2997749	335,8015501	436,3286464	276,7834243	377,3314578	422,4578605	214,1723179	352,4950843	288,8249505	337,9612282	248,5017846	344,2112378	321,443083
TRIB1	770,1356274	0,770738907	0,27462987	2,806464229	0,005008847	0,022242084	651,9206044	411,2248172	453,8809785	1198,954041	612,6488004	649,6537989	679,0119886	801,0813562	672,6925214	635,6856426	1253,953183	413,8603904	1199,282252	666,3271291	451,3718298	870,0600985	1319,138022	1252,396434	671,1387287	884,1976427	1008,599073	751,5056963	686,6703399	379,7725013	546,4796971	902,0187436
ISG20	58,7088898	1,097137266	0,394409529	2,78172099	0,00540715	0,023844031	23,61512617	26,70291021	29,83992592	139,9012884	38,45076153	45,42071051	65,02508914	53,77121432	39,82478247	39,73035266	73,5281581	35,36148905	53,28475589	28,94148137	28,97779874	83,62965723	94,22414444	76,32630629	39,94873385	49,12209126	54,03209321	45,8007203	126,5861844	30,88722746	131,2970701	72,20106171
ITGB2	106,2123861	0,827153745	0,29994943	2,757643998	0,005821956	0,025496154	67,2673291	49,40038388	45,54515009	81,14274727	103,0480409	93,59419136	125,0482484	91,08185283	79,64956495	61,75906304	83,46439568	60,24549987	143,3434982	69,99800144	67,16031002	94,01843452	117,7801806	153,727631	141,8180052	57,96406769	144,0855819	150,488081	211,3750438	77,9200511	198,7198899	191,8767941
ERRFI1	1204,035585	-1,58143329	0,576866627	-2,741419274	0,006117439	0,02660667	589,6625445	4188,351466	3425,309391	787,6442536	2769,992861	231,2327081	1568,105034	789,5589531	331,5846019	524,3619811	1514,282607	3272,902264	266,4237794	1416,786471	868,6521316	2274,622789	250,2828837	260,1544524	1342,277457	437,1866122	694,6983412	335,5603793	668,7572006	973,6496475	1302,324993	220,5594077
BIRC3	77,08791042	1,302657847	0,475815613	2,737736662	0,006186359	0,026723388	20,03707675	45,39494735	39,26306042	216,846997	43,06485291	89,46503586	63,14936542	47,73566986	58,87141757	38,15687335	135,1328311	34,05180427	54,03524541	39,03734696	32,04603625	95,57675111	191,3927934	93,52660066	29,96155039	56,98162586	115,7830569	114,0344465	183,9082302	38,60903433	46,131403	82,09161811
TIPARP	291,5531356	-1,088519107	0,398077088	-2,734442998	0,006248591	0,026809834	178,1868611	1539,422773	260,7067212	327,3690148	307,6060922	165,1662201	318,8730333	200,8190249	139,3867387	174,6562038	238,4697019	180,7364996	156,1018201	176,341119	469,4403396	221,8003952	220,8378385	224,6788453	262,6629251	224,979178	228,9931569	178,5293383	297,3581124	383,2824135	305,1769737	198,8001836
NOX4	1072,790522	-1,076574786	0,395866562	-2,719539584	0,006537287	0,02786025	2180,463317	1463,319479	1923,889961	1329,06224	962,2943919	1727,363385	754,6661788	699,0257862	543,6948564	504,3001199	393,4750082	1943,572213	590,6352519	2120,804832	917,0621012	380,229249	509,3992809	1165,319944	1377,2326	1116,053913	604,6448526	1170,255139	471,7126683	1511,368198	330,01696	1202,691658
C5AR1	106,0259874	0,776981373	0,286609536	2,710940413	0,006709268	0,028336948	62,25805992	77,4384396	54,96828459	120,315108	114,3269309	167,9189904	73,15322529	97,11739729	90,90439478	53,10492682	141,0945736	60,24549987	81,05286811	74,70940538	83,52424342	174,5314586	88,33513541	136,5273366	105,8641447	92,34953157	136,3667114	78,51552051	179,131393	93,36366483	205,8170288	113,7413986
PSME1	1205,282638	0,375874278	0,138724393	2,709503846	0,006738392	0,028336948	963,9265139	1026,726897	1001,993302	1751,564131	1105,331225	1402,536485	1198,58746	1458,407017	1222,447671	1157,687405	1313,570608	973,0957913	1404,165891	1247,17593	1038,768856	808,7663124	1416,306671	1268,52171	1327,296682	934,3021757	1229,87336	1344,110934	1375,729098	1057,887541	1075,216547	1233,352383
G6PD	218,1459458	0,489337355	0,180803497	2,706459563	0,006800486	0,028409927	150,2780757	164,2228978	196,3153021	205,6548939	203,0200209	211,9633157	249,4712555	274,8916161	204,3184492	236,4152668	331,8703352	158,4718583	216,8914711	188,4561577	174,2077077	231,1502948	114,835676	210,7036061	272,6501086	251,5051072	316,4736888	169,1822525	268,6970895	202,8729258	220,0113066	247,26391
FN1	649,3347725	1,096813123	0,40628192	2,699635574	0,006941547	0,028809687	367,1078705	419,2356902	301,540304	640,7479008	302,9920009	439,0668683	577,0976661	2086,652327	554,0839301	427,5930034	888,2996397	284,2015972	1243,561134	405,1807391	415,9166407	620,7294434	459,3427042	464,4079481	960,7670492	697,5336959	365,3598684	487,9178775	1059,263637	328,527783	1501,044882	584,5318832
DCAF12	198,5652652	0,348081809	0,129611111	2,685586196	0,00724027	0,02966175	149,5624658	186,9203714	153,9111968	230,8371258	196,3552222	183,0592272	195,0752674	226,6072604	236,3514264	185,670559	208,6609892	158,4718583	241,6576253	171,6297151	197,730862	161,5454869	197,2818024	213,9286613	193,7513592	178,8044122	226,4202001	190,6805498	195,850323	177,6015579	259,0455707	245,2857987
MYOM2	55,78542033	-1,068298871	0,397490684	-2,687607317	0,007196597	0,02966175	59,39562038	92,12504021	89,51977776	27,98025768	35,88737743	48,17348085	25,63489091	19,75269098	50,21385616	25,17566901	57,63017797	100,845728	29,26909126	71,34411685	24,88681539	19,219238	85,3906309	29,02549676	38,95001551	55,99918404	56,60505003	51,40897176	42,99153433	65,98634958	191,6227509	55,38711583
LILRB1	46,73743666	1,178800708	0,440457354	2,67631065	0,007443761	0,030299926	21,46829652	17,35689163	28,2694035	92,33485034	29,73525558	34,40962918	40,01543947	65,29361739	51,94536844	18,48838193	47,69394039	19,6452717	74,29846243	13,46115412	20,45491676	67,00761355	70,66810833	98,90169265	39,94873385	33,40302206	43,74026593	45,8007203	101,5077894	19,65550839	60,32568085	59,34333839
TMEM178A	274,2849525	-0,890682764	0,334185984	-2,665230762	0,007693551	0,031117228	437,2376392	429,9168543	629,7794891	330,1670406	293,7638181	220,2216267	130,0501783	146,4991247	493,4810002	117,2242088	192,7630091	328,7308797	282,1840593	169,610542	296,2553777	289,8468865	285,6169378	196,7283669	177,7718657	184,6990631	455,413357	190,6805498	106,2846265	308,1702922	127,7485006	310,5634709
IL10RB	136,1614417	0,615860649	0,231862281	2,656148501	0,00790388	0,031765594	81,57952679	100,1359133	98,94291226	207,0539068	100,4846568	119,7455095	98,7881162	128,9411772	175,7484966	106,2098536	194,7502566	89,05856502	127,5832183	90,18973263	109,7747199	131,9374716	197,2818024	160,1777414	135,8256951	128,6998791	120,9289705	158,9004582	167,1893002	107,4033137	255,4970013	147,3692903
CD14	521,817538	0,971472959	0,366134231	2,653324588	0,007970319	0,031817138	321,308838	172,2337708	265,4182884	453,2801744	291,200434	363,3656841	700,2701908	545,3937453	722,0406214	610,5099735	300,0743749	313,0146623	717,4679807	438,1605667	277,8459526	250,3695328	868,6288316	959,9914298	417,4642688	346,8019643	447,6944866	874,8872286	784,5955014	336,9515723	386,7940713	1401,491842
KIR3DL1	31,29879977	1,67454434	0,631580222	2,651356522	0,008016917	0,031817138	18,60585699	1,33514551	20,41679142	194,4627909	12,30424369	24,77493301	20,00771974	14,81451823	30,30146493	13,76794399	43,71944536	9,167793458	16,51076943	18,17255807	10,90928894	35,84128167	64,7790993	16,12527598	13,98205685	16,70151103	54,03209321	28,9759659	21,49576716	30,88722746	63,87425031	17,80300152
GDF15	879,9206541	1,181089557	0,446100816	2,64758439	0,008106912	0,031974468	598,965473	349,8081237	237,1488849	404,3147234	449,6175715	923,5544471	608,9849695	650,1927446	296,9543563	655,7475038	2156,163555	302,5371841	1000,40253	313,6448911	558,4192274	2016,461673	1716,646132	1312,597464	766,0169716	1115,071472	1054,912296	647,7530442	2175,849321	362,2229403	1185,2222	1018,727309
IFIT1	185,7980379	-0,734304947	0,278250656	-2,639005269	0,008314969	0,032592625	298,4093217	305,7483219	362,7906783	151,0933915	110,2255164	103,2288875	211,956781	210,1466845	284,8337703	252,1500599	85,45164319	210,8592495	213,1390236	179,0333498	144,8889937	92,97955679	229,6713521	255,8543788	171,7795556	147,3662738	126,0748841	164,5087096	182,7140209	114,4231381	95,81137547	125,6100663
EPO	69,20712141	2,769490631	1,053634196	2,628512479	0,008575921	0,033409262	7,871708725	20,02718265	7,852612084	521,8318057	4,101414563	143,1440574	4,376688692	6,584230325	309,0749422	9,440875879	17,88522765	15,71621736	94,56167946	279,3189481	3,409152793	11,94709389	79,50162187	7,525128789	41,94617055	20,63127833	30,87548183	72,90726905	5,971046434	7,721806866	60,32568085	14,8358346
ATF3	193,7590893	-1,177345759	0,448346457	-2,625973153	0,008640163	0,03345429	402,8883647	600,8154796	190,0332124	254,6203449	53,83106614	126,6274354	71,27750156	113,5779731	100,4277123	95,98223811	274,2401572	159,7815431	144,0939878	66,63271291	123,0704158	205,1783516	214,9488295	173,0779621	89,88465117	122,8052281	167,2421933	151,4227896	426,3327154	76,51608622	400,9883492	231,4390197
CASP4	235,9606316	0,702941123	0,269899862	2,604451585	0,009202142	0,035414305	177,4712513	134,8496965	152,3406744	492,4525351	180,9749176	172,0481459	235,0907069	238,6783493	193,9293755	185,2771891	315,9723551	150,6137497	273,9286746	161,5338495	146,2526548	350,1017948	267,9499108	323,5805379	260,6654884	194,5234814	257,2956819	295,3679105	279,4449731	117,9330503	326,4683905	250,2310769
SERPINE1	447,2249742	2,009894338	0,778433754	2,581972234	0,009823749	0,037578798	153,8561251	185,5852259	98,94291226	598,7775143	265,566593	101,8525024	189,4480963	1118,221784	122,0716158	350,0991472	1963,400546	112,6328911	161,3552467	76,7285785	271,027647	1213,928627	135,4472076	83,85143508	441,4335091	131,6472046	293,3170774	84,12377198	1416,332214	109,509261	1909,13037	39,5622256
GATA3	514,4392815	-0,475596504	0,184752383	-2,574237458	0,01004613	0,037784426	655,4986538	679,5890648	813,5306119	465,8712903	661,8657751	521,6499783	413,909702	343,4773486	582,6538827	551,8978691	443,1561961	610,3131073	493,8221039	352,0091803	482,0542049	556,3190242	468,1762177	449,3576905	599,2310078	498,0980054	563,4775434	292,5637848	463,3532033	648,6317767	298,0798348	466,834262
IFNGR1	533,7769488	0,397972658	0,154600601	2,574198641	0,010047257	0,037784426	440,1000787	449,944037	425,611575	541,4179861	659,8150678	481,7348085	580,8491136	679,8217811	425,9520212	460,2426991	606,1104924	379,8085861	527,5941322	537,1000495	527,7368523	686,6981792	412,2306319	634,2608551	607,2207546	407,7133574	494,0077093	563,6292722	803,70285	521,5729547	510,9940025	512,3308215
TAP1	149,7974677	0,660715502	0,256698474	2,573897275	0,010056012	0,037784426	93,74489481	102,8062043	116,2186588	230,8371258	129,1945587	139,0149019	188,1976138	158,0215278	111,6825422	100,3093062	186,8012665	86,43919546	164,3572048	135,2845989	98,86543099	153,2344651	232,6158566	198,8784037	90,88336952	96,27929887	190,3988046	234,611853	238,8418574	132,6746816	149,0399174	135,5006227
SLC22A2	1663,350808	-1,010990556	0,393277463	-2,570680118	0,010149903	0,037912873	3180,170325	2675,631603	3010,691473	1676,017435	728,001085	2236,625897	738,4099065	1242,224788	1972,192489	1163,194582	365,653543	2082,3988	2383,554714	1906,099424	2114,015647	683,0621071	1519,364329	2324,189777	1346,272331	2328,387126	802,7625276	1590,873999	735,6329207	1678,44002	457,7654606	2305,488697
ADORA2A	56,33238108	0,798701993	0,313534769	2,547411235	0,010852546	0,04030039	40,07415351	36,04892878	29,83992592	113,3200436	35,37470061	57,80817702	45,01736941	47,73566986	72,72351582	60,97232339	67,56641555	32,74211949	72,04699388	44,42180861	45,00081686	65,96873582	70,66810833	67,7261591	25,96667701	47,15720761	61,75096367	37,3883431	69,26413864	45,62885875	134,8456396	37,58411432
TNFSF14	31,55732248	1,596583552	0,631007765	2,530212212	0,011399356	0,042084831	12,88097791	8,010873062	6,282089667	72,74866996	18,45636553	33,03324401	16,25627229	17,00926167	25,10692808	8,654136223	55,64293045	14,40653258	15,76027991	15,48032724	7,159220865	16,10260481	73,61261284	66,6511407	11,98462016	8,841976427	41,16730911	31,78009164	39,40890646	16,84757862	152,5884869	34,6169474
NFKB1	121,5585117	0,539872123	0,214367206	2,518445496	0,011787413	0,043265936	89,45123551	89,45474919	91,09030018	211,2509455	123,5551137	118,3691244	120,6715597	132,2332924	133,3264457	121,1579071	121,2220985	89,05856502	155,3513305	100,9586559	95,11536292	83,62965723	150,1697302	151,5775942	95,87696125	78,59534601	164,6692364	155,1616239	103,896208	87,74780529	170,3313342	126,5991219
KLHL13	315,8378743	0,509847638	0,204197484	2,496836046	0,012530688	0,045729809	241,1605309	329,7809411	230,8667953	526,0288443	302,479324	374,3767655	320,748757	472,9672117	288,2967949	347,3455584	250,393187	189,9042931	493,0716143	266,5308516	263,5275109	344,3879673	297,3949559	394,5317522	321,5873075	248,5577818	321,6196024	362,6669281	275,8623453	223,2304167	212,9141677	311,5525266
ALAS1	311,1076221	0,468295745	0,189917875	2,465780242	0,013671518	0,048161335	171,0307623	238,9910463	307,8223937	249,0242933	344,5188233	423,9266315	322,6244808	318,7864849	234,6199141	313,909123	427,258216	231,814206	288,1879755	337,8749685	271,7094776	252,4472883	306,2284694	295,6300596	292,6244755	552,1323057	380,7976093	292,5637848	322,4365074	334,1436426	266,1427096	311,5525266
IL17RB	1765,977236	0,885797133	0,359358677	2,464938764	0,013703667	0,048161335	1197,215336	1073,45699	1012,986959	1677,416448	1996,363539	2254,518904	2102,061055	1727,263089	5055,150106	1220,626577	1208,24649	1079,180258	1190,276378	1636,203284	2288,905185	1067,966306	1610,643969	1183,595257	1558,00062	3075,042913	2421,152367	938,4474118	1583,521514	1113,344154	755,8452954	3887,97772
IL6ST	3311,415906	0,361156865	0,146134763	2,471395978	0,01345867	0,048161335	2588,360951	2770,426934	3048,384011	3679,403885	4829,928325	3136,781796	4015,299255	3767,825804	2695,964622	3395,961728	3424,02747	2602,343657	3766,706899	3643,934421	3873,820318	2826,266863	2994,561091	3237,955416	3304,759008	3166,410003	2717,042401	3007,892202	4315,872363	4063,074376	2388,187248	2835,62252
ISG15	88,18942877	1,004944181	0,406495953	2,47221202	0,013427985	0,048161335	30,77122502	54,74096592	53,39776217	60,15755401	39,47611517	48,17348085	111,292941	195,3321663	121,2058597	149,4805348	166,9287914	61,55518465	78,05091003	41,72957778	52,16003773	49,86613102	156,0587392	173,0779621	59,92310078	59,92895134	92,6264455	75,71139478	149,2761609	43,52291143	74,5199587	93,96028579
LTB	93,1401629	1,336210905	0,542258919	2,464156623	0,013733608	0,048161335	25,76195583	30,70834674	39,26306042	47,56643805	27,17187148	30,28047368	73,15322529	133,3306641	70,1262474	187,6374081	55,64293045	44,52928251	160,6047572	22,2109043	55,56919052	178,1675306	188,4482889	184,9031645	33,95642378	80,56022966	84,90757504	110,2956121	304,5233681	22,46343816	56,77711139	173,084737
MS4A7	117,0138276	0,985177019	0,400237581	2,461480547	0,01383649	0,048161335	52,95513142	72,09785756	45,54515009	144,098327	87,15505947	82,58311003	128,7996958	110,2858579	156,7018615	57,03862511	53,65568294	56,31644553	171,8621	63,26742438	69,20580169	99,21282317	123,6691896	395,6067706	104,8654264	91,36708974	156,950366	93,47085775	169,5777187	56,15859539	134,8456396	265,0669115
RGN	684,3681555	-0,822765128	0,33440771	-2,460365307	0,013879566	0,048161335	1255,179737	1236,344743	1121,353006	789,0432665	1087,387536	1191,949555	338,2555118	478,4540703	457,1192423	476,7642319	570,3400371	965,2376826	432,2819633	979,9720202	593,8744165	487,753094	335,6735146	534,284144	685,1207856	756,4802054	285,598207	504,7426319	251,9781595	813,5976507	269,6912791	895,0953541
RNF149	482,7681917	0,340524621	0,13762819	2,474235987	0,013352147	0,048161335	370,68592	423,2411268	398,9126939	548,4130505	503,4486376	455,5834903	539,5831916	506,985735	386,1272388	429,5598525	457,0669287	354,9245753	471,3074183	506,8124527	375,3477225	468,0144171	530,0108125	507,4086841	485,3771163	417,5377757	509,4454502	525,3062206	666,368782	431,0172196	674,2281978	609,2582742
THBD	287,6934946	0,750281464	0,304445492	2,464419689	0,013723531	0,048161335	150,9936855	176,2392074	153,9111968	317,5759246	157,9044607	283,5353444	199,4519561	230,9967472	155,8361053	174,6562038	339,8193253	136,2072171	275,4296537	217,3976391	180,685098	347,5046005	441,6756771	712,7371982	235,6975297	227,9265034	352,4950843	350,5157166	557,6957369	241,4819602	351,3083767	510,3527102
PTPN6	194,2611864	0,466354337	0,18974175	2,457837225	0,013977651	0,04823809	172,4619821	122,8333869	152,3406744	239,2312031	212,7608805	169,2953756	192,5743025	212,341428	185,2718141	171,902615	190,7757616	130,968478	241,6576253	156,8224455	141,4798409	261,277749	197,2818024	273,0546732	214,7244445	161,1204593	241,857941	183,2028812	268,6970895	124,9528747	209,3655982	222,537519
PLAUR	46,73179484	1,176021124	0,481210381	2,443881452	0,0145302	0,04987393	25,04634594	25,3677647	14,13470175	68,55163131	19,994396	41,29155501	23,75916719	84,4976225	18,18087896	22,81545004	71,54091058	17,02590214	40,52643406	22,2109043	19,7730862	51,94388648	64,7790993	63,42608551	26,96539535	39,29767301	77,18870458	39,25776026	95,53674295	25,27136792	163,2341952	53,40900456
TAPBP	873,9432088	0,517791477	0,212281224	2,439176985	0,014720757	0,050256346	651,9206044	691,6053743	618,7858322	1080,037946	581,8881911	1003,384787	1083,543072	1269,110395	760,1338916	572,3531002	1110,871362	624,7196399	864,5639265	1002,855982	725,4677143	889,2793365	1257,303427	832,0642404	1014,69784	998,1608944	643,2392048	722,5297304	1434,245353	777,7965461	613,9025169	898,062521
CCL19	136,0841294	1,94772227	0,804671262	2,420519237	0,015498359	0,052071207	19,32146687	25,3677647	23,55783625	135,7042497	15,38030461	22,02216267	165,0636878	25,78823544	21,64390352	13,76794399	129,1710886	18,33558692	242,4081148	25,57619283	59,66017387	62,85210264	200,2263069	234,3540109	26,96539535	59,92895134	90,05348868	142,0757038	1536,947352	42,82092898	117,1027922	82,09161811
CCL2	814,2544938	1,263881675	0,521766582	2,422312425	0,015422085	0,052071207	666,948412	184,2500804	224,5847056	684,1173002	182,0002712	421,1738611	564,5928413	1243,32216	591,3114441	1588,033997	673,676908	268,4853798	1367,391905	470,4673366	208,2992356	491,3891661	1981,651538	1695,304014	553,2899639	545,255213	704,9901685	2072,248916	1359,010168	233,7601533	539,3825582	1655,679141
EEF1A1	53507,41852	-0,347788743	0,143517367	-2,423321656	0,015379303	0,052071207	69999,52764	65755,91638	60670,85148	63385,07673	52740,08987	51107,93402	47190,70796	63118,07795	51095,19591	44627,80702	63740,96408	71113,26417	65342,12051	45714,75246	51058,88138	32557,38916	53313,19873	60447,20952	53219,70324	48621,04593	53285,93573	49701,25919	48755,98255	42978,17305	37827,75046	43824,06635
HAVCR2	370,6566527	0,970948081	0,406451934	2,388838629	0,016901724	0,056191597	230,4263827	230,9801733	172,7574659	1152,786616	257,8764407	469,347342	514,573542	165,154444	116,877079	363,4737214	284,1763948	200,3817713	487,0676982	684,4996872	387,2797573	221,8003952	406,3416229	443,9825985	578,2579225	298,6623149	409,1001343	440,24774	189,8792766	456,99057	117,1027922	357,049086
PTX3	41,5859982	1,579413827	0,660674792	2,390607067	0,016820544	0,056191597	17,17463722	16,02174612	14,13470175	120,315108	17,43101189	8,258311003	13,75530732	48,83304158	15,58361053	14,94805348	202,6992466	18,33558692	40,52643406	14,80726954	12,95478061	109,6016005	44,16756771	18,27531277	56,92694574	17,68395285	79,7616614	25,23713159	80,01202222	14,74163129	947,4680463	30,66072484
C3AR1	87,68238705	0,68587338	0,289071866	2,372674276	0,01765983	0,058137116	65,83610934	72,09785756	29,83992592	134,3052369	69,72404757	71,57202869	88,15901509	91,08185283	78,78380881	59,39884407	69,55366307	47,14865207	93,81118994	69,99800144	72,61495449	49,86613102	106,0021625	135,4523182	77,90003102	75,64802054	164,6692364	96,27498349	145,693533	60,37049004	131,2970701	122,6428993
SAMHD1	481,8082038	0,707182607	0,298079741	2,372461153	0,017670021	0,058137116	290,5376129	341,7972506	240,2899298	439,2900455	664,4291592	377,1295358	618,9888293	465,2856096	313,4037229	319,4163006	357,7045529	289,4403363	621,4053222	337,2019108	1082,065096	431,1342577	474,0652267	643,9360207	537,3104703	206,3127833	349,9221274	409,402357	917,1527323	683,7308989	464,8625995	650,7986111
PTPRO	212,0135021	-0,949385765	0,404676272	-2,346037636	0,018974191	0,062106245	289,1063932	253,677647	818,2421792	366,5413756	216,3496182	258,7604114	150,6831393	146,4991247	143,7155194	54,28503631	244,4314445	192,5236626	78,80139955	203,2634273	162,6165882	191,1535022	47,11207222	168,7778886	205,7359793	101,191508	216,1283728	134,5980352	89,56569651	319,4020113	212,9141677	246,2748543
HYAL2	707,2762814	0,438571801	0,187318534	2,341315577	0,019215917	0,062574909	409,3288537	667,5727552	538,689189	663,132107	591,1163739	626,255251	789,0544471	818,0906179	686,5446196	791,4600946	602,1359974	505,538325	653,6763715	603,0597047	880,2432511	965,1174107	677,2360382	752,5128789	884,8644549	430,3095194	774,4600026	655,2307128	1109,420427	957,5040514	571,3196834	785,3101781
LIF	182,7509223	1,306584681	0,559565561	2,334998382	0,019543505	0,063316968	78,00147736	49,40038388	43,97462767	184,6697007	28,70990194	49,54986602	93,16094502	204,1111401	52,81112458	97,94908725	230,5207119	57,62613031	487,0676982	47,78709714	60,68291971	191,1535022	262,0609017	475,1581321	97,87439794	324,2058023	218,7013296	207,5053042	284,2218103	32,99317479	322,919821	568,7069929
HLA-E	2673,48826	0,418339434	0,17938594	2,332063671	0,019697342	0,063491432	1951,468154	2093,50816	2322,802654	4220,821871	2749,485788	2642,659521	3136,210069	2866,334935	2389,486948	1957,408266	3429,989213	2150,502408	2461,605624	2455,314512	1733,21328	2813,280892	3898,523976	3398,133157	2818,383173	2182,985736	2354,25549	2791,039812	3348,56284	2509,587231	2207,210205	2627,920835
ABCF1	291,6331437	0,267061826	0,115257755	2,317083347	0,020499192	0,065742358	227,5639431	263,0236655	255,9951539	223,8420614	342,9807928	308,3102774	316,3720683	278,1837312	317,7325036	316,6627118	323,9213451	256,6982168	277,6811222	294,7992753	293,8689707	223,3587118	382,7855868	294,5550412	291,6257571	317,3287095	275,3063797	296,3026191	279,4449731	325,0178708	312,2741126	286,8261356
MMP14	362,3368124	0,546213499	0,237114688	2,303583572	0,021246031	0,067795124	308,42786	283,0508482	218,3026159	394,5216333	327,0878114	282,1589593	412,6592196	542,1016301	290,0283071	236,021897	391,4877607	209,5495648	501,3269991	289,4148137	268,3003248	394,7735372	373,9520733	528,909052	432,445044	263,2944091	494,0077093	324,3438764	585,1625505	226,0383464	372,5997935	470,7904846
KDR	431,2420969	-0,458064892	0,199061902	-2,301117824	0,021384972	0,067897286	680,5449998	439,2628729	756,9918049	479,8614192	615,2121845	487,2403492	326,3759282	359,9379244	338,510651	333,5776144	339,8193253	417,7894447	436,7849004	412,5843739	364,7793488	460,742273	326,840001	482,6832609	398,4886202	350,7317316	344,7762138	357,0586766	369,0106696	532,8046737	251,9484318	547,9368245
VEGFA	1639,705135	-0,544862376	0,238119061	-2,288193025	0,022126283	0,06990144	2090,296471	1839,830513	2911,748561	1906,854561	1789,75478	1903,540686	1146,692437	1021,104386	1083,060932	1147,066419	1530,180587	1883,326713	1093,46323	1404,671433	1947,64899	2359,810763	1110,078202	1127,6943	1481,099308	1929,515745	1497,460869	1116,97675	1190,626659	1883,418893	2657,878527	1578,532801
LHX6	36,77369691	0,869887721	0,38119649	2,281993	0,022489754	0,070433875	20,75268664	16,02174612	18,846269	58,75854112	12,81692051	27,52770334	21,88344346	37,31063851	25,97268422	27,92925781	23,84697019	15,71621736	42,77790261	24,90313513	34,43244321	46,74949783	67,72360382	62,35106711	19,97436693	20,63127833	66,8968773	32,71480021	64,48730149	29,48326258	85,16566708	50,44183764
TFRC	839,3524701	0,568355821	0,249110875	2,281537574	0,022516656	0,070433875	401,457145	821,1144888	758,5623273	675,7232229	1272,976545	1220,853643	778,425346	1009,033297	562,7414915	795,7871627	1120,807599	504,2286402	522,3407056	551,9073191	875,4704372	729,2921661	815,6277503	743,9127317	874,8772714	1472,680296	1322,499805	887,9731486	714,1371535	946,9743147	777,1367121	666,6235013
MAPK14	512,2693384	0,319580684	0,140774476	2,270160703	0,023197834	0,072208945	431,5127601	451,2791825	452,310456	678,5212487	574,7107157	503,7569712	509,571612	515,2160229	503,0043178	447,6548646	592,1997598	388,9763796	535,0990274	496,7165872	518,8730551	783,313808	356,2850462	513,8587944	597,2335711	482,3789362	512,0184071	496,3302547	561,2783648	425,4013601	503,8968636	487,6044305
LILRB4	54,80352717	0,888959941	0,392193717	2,266634836	0,023412542	0,072521777	30,05561513	21,36232817	28,2694035	76,94570861	36,40005425	27,52770334	52,52026431	57,61201534	50,21385616	19,66849142	47,69394039	30,12274993	120,8288126	33,65288531	23,18223899	49,34669215	61,83459479	89,22652707	55,9282274	55,99918404	38,59435229	73,84197762	111,0614637	32,29119235	78,06852816	122,6428993
BTG2	488,4018814	-0,607231838	0,269670614	-2,251753832	0,024337833	0,07502196	486,6147212	1471,330352	428,7526198	636,5508622	280,9468976	622,1260955	346,3836479	495,463332	399,1135809	314,3024928	506,7481166	440,054086	438,2858794	325,7599298	368,8703322	487,2336551	627,1794614	477,3081689	343,5591111	420,4851012	481,1429252	487,9178775	496,7910633	353,7991509	358,4055156	603,3239403
PTPRC	204,4262807	0,911322253	0,405742626	2,246059923	0,02470017	0,075771053	95,17611458	105,4764953	102,0839571	257,4183706	163,0312289	122,4982799	207,5800923	182,7123915	136,7894702	86,93473206	168,9160389	92,98761936	325,7124515	80,09386703	106,0246519	346,9851617	259,1163972	505,2586473	155,800062	132,6296464	198,1176751	257,9795674	531,4231326	82,13194576	180,9770426	431,228259
IL17RC	243,5375581	-0,391042809	0,176018643	-2,221598811	0,02631043	0,080322708	265,491267	332,4512321	309,3929161	202,8568682	270,1806843	211,9633157	267,6032515	265,0152706	232,0226457	208,0926392	341,8065728	344,4470971	196,6282541	308,2604294	258,0728664	165,181559	276,7834243	248,32925	192,7526408	279,9959202	203,2635887	206,5705956	170,771928	234,4621357	134,8456396	204,7345175
CXCL8	50,75654775	1,592746701	0,719516143	2,213635809	0,026853842	0,081589424	22,18390641	6,675727552	15,70522417	93,73386322	10,25353641	12,3874665	30,0115796	59,80675879	23,3754158	18,48838193	65,57916803	14,40653258	28,51860174	6,057519356	6,477390306	95,57675111	41,22306319	122,5520974	10,98590181	34,38546388	84,90757504	42,99659457	83,59465008	14,03964885	319,3712516	56,37617147
IL2RA	25,56054749	1,09886123	0,500223418	2,196740875	0,028038958	0,084784468	17,8902471	12,01630959	7,852612084	62,95557977	8,715505947	9,63469617	40,64068071	26,88560716	27,7041965	11,01435519	3,974495032	9,167793458	26,26713318	33,65288531	12,61386533	21,81643232	23,55603611	46,22579113	25,96667701	16,70151103	15,43774092	38,32305168	56,12783648	21,76145571	46,131403	41,54033688
F3	114,6704128	-0,699845511	0,320268677	-2,185182508	0,028875468	0,086900104	146,7000262	156,2120247	293,6876919	176,2756234	58,95783435	89,46503586	87,53377385	109,7371721	67,52897898	74,74026738	131,1583361	86,43919546	47,28083973	84,80527098	108,7519741	128,3013996	61,83459479	120,4020606	138,8218501	69,75336959	87,48053186	73,84197762	150,4703701	136,1845938	195,1713204	99,89461963
SYK	202,5931209	0,416969004	0,193070659	2,159670483	0,030798188	0,092249289	142,4063669	188,255517	147,6291072	145,4973399	186,6143626	172,0481459	179,4442364	185,4558208	325,5243089	261,9843057	198,7247516	150,6137497	279,1821013	159,5146764	241,0271024	214,5282511	267,9499108	190,2782565	185,7616124	203,3654578	272,7334229	181,333464	247,2013224	169,1777686	152,5884869	218,5812964
RHOU	198,1931545	0,553481945	0,25663685	2,156673705	0,031031097	0,092510548	129,525389	146,8660061	146,0585848	370,7384142	147,6509243	139,0149019	185,0714076	250,7494382	182,6745457	163,2484787	174,8777814	136,2072171	192,125317	152,1110416	149,6618076	158,9482926	320,950992	289,1799492	218,7193179	142,4540647	249,5768115	227,1341843	321,2422982	162,8599266	131,2970701	264,0778559
CD209	128,0007054	1,032135565	0,480291762	2,1489762	0,031636287	0,093874028	55,10196107	46,73009286	43,97462767	103,5269534	22,5577801	82,58311003	117,5453535	61,45281637	179,2115211	64,90602167	79,48990065	61,55518465	200,3807017	87,4975018	53,52369885	95,05731225	220,8378385	389,1566602	56,92694574	83,50755514	138,9396682	111,2303207	294,9696938	63,17841981	241,3027234	372,8739762
MCAM	145,7909499	0,753647898	0,351215015	2,14583052	0,031886503	0,094176415	92,31367505	96,13047674	59,67985184	172,0785847	39,47611517	92,2178062	140,0540382	230,4480614	165,3594229	160,8882598	172,8905339	106,0844672	201,1311912	121,8234448	99,54726155	148,5595153	164,8922528	165,5528334	156,7987804	79,57778784	146,6585387	164,5087096	391,7006461	129,1647694	212,9141677	80,11350683
CALHM6	45,23772155	0,84432895	0,393867235	2,143689229	0,032057796	0,094243983	30,77122502	21,36232817	17,27574659	82,54176015	44,09020655	24,77493301	41,8911632	46,63829814	25,97268422	27,53588798	37,75770281	27,50338037	75,04895195	22,88396201	38,52342656	28,0496987	108,946667	93,52660066	54,92950905	32,42058023	38,59435229	28,9759659	77,62360364	24,56938548	74,5199587	49,452782
IFNAR1	575,5335517	0,292627709	0,13663538	2,141668643	0,032220156	0,094284788	573,2035172	364,4947243	637,6321012	754,0679444	591,1163739	509,2625118	589,602491	657,8743466	651,914374	643,9464089	580,2762747	428,266923	769,2517575	533,0617033	519,2139703	546,4496857	559,4558576	602,0103031	570,2681758	545,255213	578,9152844	631,8629984	550,5304812	422,5934303	610,3539474	542,9915463
MAP3K1	557,6575205	0,5424108	0,253523199	2,13949178	0,032395861	0,09436409	506,6517979	419,2356902	406,765306	591,7824499	514,2148509	371,6239951	652,1266152	674,3349225	705,5912547	697,0513358	502,7736216	310,3952928	1050,685327	388,3542965	506,9410203	608,2629106	585,9563982	1010,517295	484,378398	423,4324267	650,9580753	480,4402088	530,2289233	296,9385731	422,279766	707,1747825
THBS1	2253,42474	0,591020465	0,276610378	2,136653257	0,032626204	0,094601095	1990,111088	1842,500804	1268,982113	2418,893276	1089,438243	1804,440954	1995,770044	3742,037568	2128,89435	2357,072011	3320,6906	1270,394236	3281,140179	1395,248625	1481,958719	1957,245642	2146,543791	3064,877454	2801,404962	1709,448776	3051,526788	3544,414926	2854,160195	1295,859589	1973,004621	2802,983684
BMP4	169,0573253	-0,576044774	0,27045231	-2,129931056	0,033177306	0,095761769	198,2239379	268,3642476	268,5593333	141,3003013	113,8142541	134,8857464	163,8132053	111,3832297	177,4800089	182,9169702	164,9415438	184,6655539	231,9012615	102,3047713	185,4579119	86,22685155	247,3383792	164,477815	103,866708	95,29685704	154,3774092	114,0344465	210,1808345	150,2242427	315,8226821	123,631955
FOS	1133,84624	-1,142925049	0,539318702	-2,119201588	0,034073434	0,097903306	1124,223128	4388,623292	1212,443306	1101,02314	292,7384644	1408,042026	562,7171176	994,7674649	292,6255756	405,564293	1114,845857	865,7016394	1135,490643	654,8851481	466,7130173	4451,071632	742,0151375	1591,02723	354,545013	808,5496221	1065,204123	1197,361688	803,70285	243,5879075	844,5595319	1357,973394
SDHA	1334,69466	-0,312606852	0,148318925	-2,107666653	0,035059834	0,100283759	1414,760741	2137,567962	1528,118312	1012,885328	1289,89488	1526,41115	1256,109655	1327,271096	1639,742131	1507,393182	1293,698133	1517,92466	1109,22351	1403,998375	1603,324558	1014,983542	1039,410093	1133,069392	1336,285147	1288,963675	1412,553294	1321,677929	1153,606171	1458,719515	841,0109625	1133,457763
CXCL2	291,9641561	1,131864686	0,542368967	2,086890575	0,036898027	0,104134432	225,4171135	132,1794055	89,51977776	640,7479008	103,5607177	118,3691244	144,4307268	399,9919922	77,05229653	116,830839	435,207206	65,48423898	179,3669952	119,131214	118,6385172	1004,594764	315,061983	576,2098616	165,7872455	328,1355696	447,6944866	516,8938434	416,7790411	99,68150681	510,9940025	243,3076874
EGFR	353,5654279	0,254420455	0,121817236	2,088542338	0,03674894	0,104134432	273,3629757	365,8298698	325,0981403	346,9551952	357,3357438	297,2991961	457,0513477	402,1867357	350,631237	413,4316896	387,5132657	288,1306515	336,9697943	365,4703344	385,9160961	356,8545001	282,6724333	383,7815682	411,4719587	310,4516168	442,5485729	323,4091678	335,5728096	355,2031158	347,7598073	289,7933025
SELE	45,59582893	1,389920281	0,664981795	2,090162905	0,036603169	0,104134432	16,45902733	6,675727552	12,56417933	71,34965708	10,76621323	17,89300717	13,13006608	38,95669609	15,58361053	22,02871039	19,87247516	14,40653258	38,2749655	10,7689233	9,20471254	29,60801529	76,55711736	256,9293972	8,988465117	60,91139316	51,45913639	130,8592009	89,56569651	14,03964885	92,26280601	56,37617147
HLA-B	2204,718016	0,602564611	0,289729161	2,079751342	0,037548346	0,105500884	1237,28949	1293,756	2124,91683	2674,912634	1715,929318	1558,068009	4039,058422	2313,259588	1578,273445	1115,596833	2959,011552	1388,265866	3239,863256	2134,939044	1201,726359	1751,028413	3227,176947	3751,81421	2054,363638	1449,101692	2066,084326	2665,788863	3284,075539	1679,843985	2437,86722	2380,656925
HPRT1	433,325509	0,387925626	0,186742644	2,077327479	0,037771345	0,105659929	362,8142112	328,4457955	427,1820974	554,009102	675,1953725	469,347342	456,4261065	399,9919922	350,631237	358,3599136	463,0286713	297,298445	453,2956698	356,7205843	373,6431461	593,1991835	447,5646861	335,4057403	548,2963721	307,5042913	457,9863139	471,0931231	556,5015277	382,5804311	514,542572	325,3993055
IKBKB	254,9198764	0,388797953	0,188014682	2,067912725	0,038648231	0,107638713	165,3058832	217,6287182	210,4500039	221,0440357	293,2511413	184,4356124	308,2439322	266,1126423	251,935037	285,1931255	339,8193253	220,027043	292,6909126	181,7255807	266,9366637	419,7066027	220,8378385	328,9556299	242,6885582	286,873013	213,555416	205,6358871	241,2302759	202,8729258	301,6284043	259,1325777

NOS2	40,3613083	0,924030024	0,448613089	2,059748245	0,039422615	0,108914768	17,17463722	32,04349225	17,27574659	78,3447215	16,91833507	49,54986602	23,13392595	40,60275367	32,89873335	24,38892936	123,209346	27,50338037	40,52643406	18,17255807	27,61413762	87,78516814	47,11207222	30,10051516	25,96667701	32,42058023	48,88617957	24,30242302	62,09888291	44,22489387	60,32568085	16,81394588
TPMT	845,1586973	-0,480225785	0,233179551	-2,059467832	0,039449444	0,108914768	1327,456335	1070,786699	1074,237333	973,7129672	872,5759483	1057,063808	804,6854782	838,9406806	793,8983811	797,360642	620,021225	1105,373954	821,7860239	1131,410004	719,6721545	311,14388	733,1816239	776,1632837	1108,577364	858,6541552	720,4279094	777,6775365	460,9647847	887,3058071	415,182627	915,8655226
PIK3CG	57,54739137	0,697319054	0,339846146	2,051866889	0,0401826	0,110458664	36,49610409	25,3677647	32,98097075	89,53682457	30,2479324	33,03324401	53,77074679	51,02778502	70,1262474	44,4507906	37,75770281	40,60022817	98,31412706	37,01817384	40,56891823	51,42444761	73,61261284	97,82667426	33,95642378	41,26255666	69,46983412	77,58081193	96,73095223	34,39713968	95,81137547	102,8617866
GEMIN7	130,65989	0,384268386	0,187531177	2,049090675	0,040453249	0,110723333	96,60733435	116,1576594	120,9302261	240,630216	132,7832965	134,8857464	126,9239721	130,5872348	170,5539597	133,7457416	143,0818212	104,7747824	123,8307707	104,9970022	122,7295005	112,7182337	85,3906309	129,0022078	135,8256951	79,57778784	123,5019273	143,9451209	119,4209287	130,5687343	209,3655982	124,6210106
IKZF1	64,48389535	0,829727996	0,405469108	2,046340842	0,040722848	0,110757991	30,77122502	46,73009286	21,98731384	60,15755401	49,72965158	30,28047368	70,02701908	53,22252846	32,03297721	29,89610695	67,56641555	26,19369559	80,30237859	28,26842366	33,40969737	105,4460895	88,33513541	134,3772998	54,92950905	47,15720761	79,7616614	67,29901758	191,0734859	36,503087	99,35994493	111,7632873
IL10RA	82,24498972	0,7487434	0,36606085	2,045406935	0,040814756	0,110757991	48,66147212	33,37863776	43,97462767	104,9259663	55,88177342	46,79709568	74,40370777	60,35544465	69,26049126	46,41763974	47,69394039	45,83896729	130,5851764	56,53684732	61,70566555	104,4072118	73,61261284	230,0539373	83,89234109	83,50755514	51,45913639	73,84197762	212,5692531	49,84075341	81,61709762	167,1504031
PPIA	4617,690165	0,260771797	0,12849046	2,029503175	0,042407066	0,113314016	3988,809494	3738,407429	4284,385153	4223,619896	4079,882137	4438,842164	4756,210126	5551,603536	4257,7887	5393,887086	4326,237843	3680,214231	4093,16984	5117,930798	3951,208087	4950,252381	3966,24758	4636,554352	6503,653871	3836,435327	6131,356101	5276,42992	4865,208634	4816,301537	4708,951676	4486,356383
C5	62,20690226	-0,484384099	0,238251349	-2,033080195	0,042044422	0,113314016	78,00147736	65,42213001	106,7955243	76,94570861	56,39445024	55,05540668	58,77267673	65,84230325	38,95902633	58,61210442	27,82146523	72,03266288	98,31412706	68,65188603	69,88763225	45,19118123	29,44504514	80,62637988	54,92950905	54,03430038	77,18870458	37,3883431	62,09888291	56,15859539	67,42281978	55,38711583
CCL21	384,3249844	1,431573492	0,70742018	2,023653738	0,043005792	0,113314016	89,45123551	88,11960368	84,80821051	337,162105	55,3690966	85,33588036	598,9811096	149,7912399	90,90439478	56,25188545	306,0361175	151,9234344	1061,192181	113,0736946	298,6417846	186,9979913	353,3405417	1702,829143	206,7346977	106,1037171	97,77235914	331,821545	2627,260431	207,0848205	127,7485006	477,7138741
CRIP2	395,8986449	0,400031574	0,196836392	2,032304953	0,042122793	0,113314016	269,0693164	329,7809411	318,8160506	264,4134351	304,5300313	378,505921	373,8942626	404,930165	432,0123142	542,8503631	538,5440769	318,2534015	423,276089	329,1252183	432,6214894	413,9927752	391,6191003	409,5820098	589,2438243	295,7149894	373,0787388	360,7975109	669,9514099	395,9180975	422,279766	310,5634709
IRF8	90,53327993	0,73646281	0,363152022	2,027973865	0,042562915	0,113314016	42,22098316	42,72465633	51,82723976	86,7387988	82,54096808	64,69010285	57,52219424	54,86858604	50,21385616	66,08613116	69,55366307	48,45833685	158,3532886	52,49850108	43,29624047	123,1070109	106,0021625	159,102723	77,90003102	54,03430038	159,5233228	109,3609036	182,7140209	38,60903433	223,5598761	148,358346
MS4A6A	184,1195688	0,717109585	0,354214711	2,024505372	0,04291818	0,113314016	108,7727024	105,4764953	116,2186588	141,3003013	142,0114792	93,59419136	238,8421544	233,1914907	142,8497632	83,78777343	121,2220985	77,271402	356,4825218	146,7265799	191,2534717	153,753904	153,1142347	478,3831873	283,6360104	123,78767	149,2314955	214,0482643	240,0360666	143,2044182	120,6513617	428,2610921
RUNX1	103,4736113	0,882991162	0,43563915	2,026886615	0,04267401	0,113314016	52,23952154	41,38951082	54,96828459	123,1131338	47,16626748	61,93733252	63,77460666	115,2240307	98,69620004	54,67840614	153,0180587	64,1745542	208,6360864	55,19073191	41,25074879	121,0292555	203,1708115	249,4042684	39,94873385	88,41976427	69,46983412	150,488081	192,2676952	37,20506944	113,5542228	189,8986829
CFI	2299,514122	0,474571927	0,235665892	2,013748884	0,044035905	0,115548759	1788,3091	1714,326835	1682,029508	2953,316198	2707,958965	3142,287336	2686,661616	2386,234807	1750,558917	1442,88053	1766,663042	1672,467464	2917,152762	3143,852546	1837,19244	2365,52459	2170,099827	3176,679367	3363,683391	2580,874675	1644,119408	2901,335425	1803,256023	2345,32334	1014,890866	2829,688186
AXL	219,2975183	0,514077229	0,256040867	2,007793658	0,044665223	0,116239413	150,2780757	188,255517	160,1932865	167,8815461	207,1214354	118,3691244	190,6985787	367,6195265	181,8087896	237,5953763	329,8830877	142,755641	297,1938497	150,0918685	236,9361191	221,8003952	200,2263069	295,6300596	184,7628941	175,8570867	288,1711638	159,8351668	404,8369482	175,4956106	227,1084456	241,3295761
S100B	29,63259104	0,966339806	0,481235008	2,008041374	0,044638895	0,116239413	20,75268664	8,010873062	15,70522417	103,5269534	21,53242646	12,3874665	33,76302706	18,65531925	16,44936667	13,76794399	33,78320777	18,33558692	20,26321703	9,422807886	21,81857787	41,55510918	55,94558576	30,10051516	22,97052197	15,7190692	48,88617957	24,30242302	46,57416219	28,78128014	56,77711139	30,66072484
MAPK8	209,1291455	-0,245027831	0,124107049	-1,974326467	0,048344633	0,124792041	267,6380966	244,3316284	274,8414229	179,0736491	232,7552765	170,6717607	206,954851	213,9874856	213,8417668	203,3722012	266,2911672	204,3108256	211,6380445	238,262428	184,4351661	159,9871703	223,782343	182,7531277	206,7346977	219,084527	200,6906319	197,2235099	150,4703701	182,515435	188,0741815	213,6360182
MT2A	7529,221575	0,907455891	0,459480047	1,974962563	0,048272397	0,124792041	6409,002122	3873,257125	4245,122093	11378,17178	10024,88255	4555,834903	3965,279955	10783,32321	5510,537836	4380,566408	14469,14917	3182,534015	3105,525632	5498,208402	7569,341946	6242,096838	2402,715683	4382,85001	5509,929117	3584,93022	8771,209797	5350,271898	29904,19475	10555,70999	13072,9299	7032,1856
CCR2	44,49037103	0,818585427	0,420718037	1,945686553	0,051692403	0,132357564	27,90878548	18,69203714	21,98731384	76,94570861	32,29863968	26,15131817	52,52026431	30,72640818	29,43570878	20,45523107	53,65568294	27,50338037	93,06070042	17,49950036	30,00054458	31,16633189	64,7790993	54,82593832	24,96795866	28,49081293	77,18870458	44,86601172	42,99153433	32,29119235	141,9427785	54,39806019
PECAM1	885,6713416	0,380395518	0,195357047	1,947180935	0,051513047	0,132357564	795,7581911	652,8861545	755,4212825	675,7232229	728,5137618	631,7607917	764,0447974	1013,422784	1042,370393	963,7560794	1063,177421	551,3772923	1079,203929	817,0920553	898,3117609	1492,867297	742,0151375	1107,26895	979,7426978	645,4642791	874,8053186	715,0520618	1267,056053	926,6168239	773,5881427	1070,158202
ITGAM	29,11655528	0,742681355	0,385893696	1,924574986	0,054282551	0,138132937	15,74341745	12,01630959	20,41679142	25,18223191	19,48171918	17,89300717	36,88923326	27,43429302	21,64390352	18,0950121	41,73219784	18,33558692	43,52839213	11,441981	14,31844173	27,53025983	50,05657673	60,20103031	26,96539535	31,43813841	36,02139547	28,04125733	31,04944146	18,2515435	39,03426408	64,28861659
TGIF1	292,5385514	0,434677771	0,225950658	1,923772976	0,054383046	0,138132937	251,8946792	248,3370649	197,8858245	307,7828345	268,6426539	260,1367966	277,6071113	371,4603275	341,1079194	284,7997557	425,2709685	221,3367278	404,513851	204,6095427	202,8445912	446,7174237	294,4504514	353,6810531	241,6898398	242,6631308	331,9114297	272,9349046	277,0565545	137,5885587	333,5655294	405,5128124
AQP1	10300,66325	-0,892950634	0,467490042	-1,91009552	0,056120916	0,1414158	19138,27074	17951,03139	17208,21412	5194,534838	4134,738556	7932,107718	12515,45394	13517,97354	20050,04646	16852,35681	2128,34209	17057,33457	11066,71846	8419,278846	12716,13992	1893,354662	5668,171189	11486,57159	13352,86429	15634,57921	4826,866993	7197,256047	4435,293291	8471,524114	1674,924786	7293,296289
VWF	182,660056	0,654272511	0,342349932	1,911122071	0,055988895	0,1414158	94,4605047	94,79533123	106,7955243	166,4825332	70,23672439	116,9927392	161,9374816	154,1807268	119,4743474	195,1114348	75,51540561	95,60698892	270,9267166	274,6075441	78,75142951	216,6060066	359,2295507	340,7808323	342,5603928	118,8754608	162,0962796	238,3506873	156,4414166	119,3370152	216,4627372	402,5456454
CD84	61,79879914	0,706262237	0,3707488	1,904961624	0,05678506	0,142523766	34,34927444	45,39494735	21,98731384	103,5269534	46,65359066	17,89300717	63,14936542	69,13441841	47,61658774	37,76350352	37,75770281	40,60022817	72,7974834	37,69123155	46,70539326	70,64368561	58,89009028	154,8026494	63,91797417	47,15720761	79,7616614	85,99318913	102,7019987	36,503087	56,77711139	126,5991219
ST5	174,6902257	0,521169831	0,273970086	1,902287353	0,057133598	0,142833995	100,1853838	126,8388235	130,3533606	253,221332	125,0931442	114,2399689	141,9297619	208,500627	144,5812755	167,9689167	158,9798013	123,1103693	234,9032196	103,6508867	150,0027229	178,6869695	318,0064875	187,0532013	165,7872455	108,0686008	210,9824592	180,3987555	272,2797174	124,2508923	365,5026546	147,3692903
CXCL14	6379,46562	-0,732666695	0,386508003	-1,895605498	0,05801223	0,144461827	10182,41304	9583,674473	8901,721059	11513,87603	3810,726806	6890,184147	4149,726122	4105,267608	3485,534223	4198,436178	4578,618277	8438,299036	7150,664142	6172,612223	3787,227837	1467,934232	10308,7103	10213,7498	5947,367753	9653,473374	4340,578154	10526,688	2420,662224	3936,015554	2938,215514	7163,73
MIR155HG	31,33888627	0,960915729	0,511356238	1,87915128	0,06022384	0,148802094	16,45902733	9,346018572	20,41679142	82,54176015	12,81692051	15,14023684	13,75530732	21,94743442	21,64390352	19,27512159	51,66843542	17,02590214	39,77594454	15,48032724	13,63661117	66,48817469	67,72360382	21,50036797	23,96924031	25,54348745	43,74026593	17,75946297	46,57416219	17,54956106	92,26280601	20,77016844
VMP1	669,7553831	0,203346902	0,108115865	1,880823899	0,059995878	0,148802094	578,9283962	656,8915911	629,7794891	646,3439524	801,3138703	718,4730572	753,4156963	761,0272884	652,7801301	612,0834529	792,9117589	576,2613031	642,4190287	758,5360349	553,3054983	529,3082032	674,2915337	717,0372717	786,9900569	701,4634632	723,0008662	690,7496388	591,133597	611,4267073	571,3196834	682,4483915
MX1	97,64207174	0,498747988	0,266127728	1,87409253	0,060917673	0,149933032	73,70781806	64,0869845	75,38507601	99,32991476	76,38884624	61,93733252	122,5472834	160,2162712	117,7428351	110,9302916	69,55366307	75,96171722	95,31216898	57,20990502	90,34254901	74,79919652	170,7812618	139,7523918	76,90131267	84,48999697	97,77235914	86,92789771	171,9661373	73,00617401	120,6513617	90,99311887
LEF1	43,35242205	0,578374647	0,309311049	1,869880331	0,061500438	0,150782926	27,90878548	30,70834674	29,83992592	46,16742517	37,42540789	35,78601434	33,13778581	64,19624567	25,97268422	27,92925781	77,50265313	28,81306515	40,52643406	31,63371219	38,86434184	73,76031879	64,7790993	29,02549676	40,9474522	29,47325476	95,19940232	36,45363452	45,3799529	43,52291143	49,67997247	42,52939252
CD47	480,0766038	0,281292607	0,150693122	1,86665856	0,061949284	0,151299213	388,5761671	432,5871453	560,6765028	637,9498751	461,4091384	432,1849425	554,5889814	564,0490645	472,7028528	465,3565069	504,7608691	310,3952928	481,8142715	357,393642	367,1657558	554,7607076	450,5091906	613,8355055	490,3707081	408,6957993	524,8831912	458,9419116	597,1046434	421,1894654	518,0911414	451,9984274
IKBKG	212,435514	0,327924186	0,175943661	1,863802223	0,062349483	0,151478502	171,0307623	192,2609535	179,0395555	190,2657522	240,9581056	216,0924712	195,7005087	207,4032552	212,9760106	225,0075418	331,8703352	170,2590214	210,887555	181,7255807	182,0487591	290,8857643	170,7812618	216,0786981	163,7898088	241,680689	241,857941	170,1169611	333,184391	202,8729258	173,8799036	210,6688513
IL1RL1	504,1494045	1,365727283	0,734534566	1,859309755	0,062983245	0,151478502	127,3785594	160,2174612	565,3880701	1536,116147	1203,252497	465,2181865	238,2169131	378,0445578	99,56195618	112,5037709	1204,271995	140,1362714	33,77202838	350,6630649	330,3469056	2239,820385	58,89009028	118,2520238	945,786274	139,5067392	542,8938889	113,0997379	248,3955317	856,4185797	674,2281978	225,5046859
MRC1	253,2551544	0,495005785	0,266706136	1,855996993	0,063453987	0,151478502	182,4805204	201,6069721	158,6227641	145,4973399	156,3664302	148,649598	327,0011694	346,2207779	286,5652826	166,0020675	184,814019	138,8265866	276,1801432	242,9738319	323,8695153	216,0865677	209,0598205	418,182157	327,5796176	202,383016	334,4843865	223,39535	439,4690175	313,7861517	195,1713204	419,3595913
NFIL3	317,8960776	0,499799968	0,269039413	1,857720257	0,06320875	0,151478502	250,4634594	200,2718265	298,3992592	633,7528364	305,555385	342,7199066	255,7236679	319,8838566	136,7894702	237,9887461	457,0669287	204,3108256	258,1683947	253,0696975	331,7105667	412,4344586	297,3949559	354,7560715	302,6116589	176,8395285	344,7762138	390,7081854	280,6391824	305,3623624	404,5369187	509,3636546
TNFAIP3	178,8216018	0,525895043	0,283053423	1,857935641	0,063178154	0,151478502	101,6166035	153,5417337	106,7955243	215,4479841	137,3973879	115,616354	221,9606408	219,4743442	109,9510299	106,2098536	184,814019	121,8006845	163,6067153	133,2654258	128,184145	343,3490896	241,4493701	239,7291028	166,7859638	136,5594137	252,1497683	143,0104124	320,0480889	117,2310679	244,8512929	224,5156303
WARS	804,63824	0,601019762	0,323803477	1,856125101	0,063435729	0,151478502	523,1108253	500,6795664	724,0108342	2788,232678	858,7336742	905,66144	655,8780626	741,2745974	553,2181739	682,1032823	995,6110056	650,9133355	697,2047637	673,0577062	575,1240761	523,5943757	1572,36541	738,5376397	790,9849303	568,8338168	589,2071116	786,0899137	930,2890344	731,4657049	496,7997247	667,6125569
JUN	304,551752	-0,870977571	0,470857301	-1,849769707	0,064346749	0,153034402	430,0815403	949,2884578	321,9570954	505,0436511	173,7974421	225,7271674	191,32382	211,7927421	161,0306422	212,8130771	194,7502566	183,3558692	244,6595834	133,9384835	183,4124202	1209,773116	194,3372979	385,931605	142,8167235	114,9456935	272,7334229	238,3506873	226,8997645	170,5817335	386,7940713	252,2091882
NLRC5	111,4438902	0,60900313	0,32954178	1,848030104	0,064597988	0,153058666	51,52391165	64,0869845	97,37238984	132,906224	69,21137075	74,32479902	95,66190999	97,11739729	101,2934685	83,00103377	127,183841	65,48423898	141,8425192	69,32494374	63,06932667	175,5703363	214,9488295	164,477815	78,89874936	83,50755514	97,77235914	111,2303207	293,7754846	76,51608622	117,1027922	150,3364573
SLA	103,2086904	0,635441764	0,346024464	1,836407046	0,066297478	0,156501482	43,65220293	84,11416715	80,09664326	107,7239921	104,5860714	49,54986602	121,2968009	94,37396799	57,13990529	50,74470785	153,0180587	49,76802163	87,05678427	56,53684732	95,11536292	161,0260481	79,50162187	104,2767846	162,7910905	53,05185856	133,7937546	88,79731486	228,0939738	107,4033137	184,525612	145,3911791
B2M	11402,02598	0,473558518	0,259156549	1,827306775	0,067653657	0,159111379	9824,608099	7336,624579	10751,79647	16175,38696	12096,60958	10702,77106	17921,91495	11486,73849	8794,350878	7485,041093	9111,529862	6668,914898	14018,39374	12341,85916	8841,296853	17385,6188	8686,288316	17872,18087	16018,44356	5900,545602	11212,94582	14395,4468	10761,01988	9895,144507	6767,121964	14000,08258
PSEN1	417,4300168	0,216985202	0,119101287	1,821854385	0,068477083	0,160453682	392,8698264	368,5001608	428,7526198	561,0041664	486,5303026	422,5502463	470,806655	459,798751	450,1931932	375,668186	463,0286713	344,4470971	361,7359484	399,1232198	391,0298253	392,1763429	403,3971184	417,1071386	496,3630181	283,9256875	488,8617957	433,70478	380,9527625	362,2229403	383,2455019	435,1844816
TLR5	58,62092746	0,47565539	0,26170763	1,817506772	0,069139552	0,161410352	34,34927444	60,08154796	29,83992592	86,7387988	52,29303568	41,29155501	51,26978182	55,4172719	71,85775968	81,82092429	55,64293045	51,07770641	64,54209868	53,17155879	61,70566555	50,90500875	53,00108125	84,92645347	55,9282274	41,26255666	66,8968773	70,10314331	42,99153433	39,31101677	124,1999312	43,51844816
SMARCA4	554,2793021	0,216140042	0,119624881	1,806815099	0,070791114	0,164660649	522,3952154	471,3063651	538,689189	615,5656689	657,7643606	494,122275	600,2315921	596,9702161	548,8893932	575,8934286	500,7863741	445,2928251	722,7214073	546,5228574	528,4186829	486,1947774	724,3481104	565,4596776	657,1566719	444,063705	548,0398025	516,8938434	569,6378298	520,8709722	514,542572	498,4840425
MET	429,5871051	0,413399455	0,229506212	1,801256064	0,071662529	0,16607922	381,4200682	305,7483219	419,3294853	658,9350683	406,0400418	359,2365286	402,6553597	502,5962481	439,8041195	475,1907526	502,7736216	285,511282	519,3387475	312,9718334	287,3915804	667,9983801	294,4504514	521,3839232	463,4053127	392,9767301	512,0184071	346,7768823	409,6137854	246,3958373	706,1653229	349,1366409
NPHS1	248,6510401	-0,765491484	0,425395866	-1,799480308	0,071942736	0,166122318	343,4927444	356,4838513	708,30561	360,945324	194,3045149	268,3951076	182,5704426	210,1466845	164,4936667	69,23308978	425,2709685	218,7173582	70,54601484	221,4359853	291,1416485	112,7182337	53,00108125	158,0277046	300,6142223	231,8562707	334,4843865	152,3574981	160,0240444	515,2551127	145,4913479	215,6141295
BRWD1	346,374698	-0,262652479	0,14662281	-1,791348016	0,073237465	0,168499241	406,4664142	460,6252011	402,0537387	388,9255817	389,1217067	305,5575071	343,2574417	301,2285374	366,2148475	327,677067	296,0998799	366,7117383	333,2173467	282,0111789	353,8700599	493,9863604	238,5048656	339,7058139	366,5296331	261,3295255	357,6409979	313,1273735	417,9732504	310,978222	298,0798348	284,8480243
CD74	10648,38906	0,615809173	0,346745305	1,775969752	0,075737903	0,172998447	5857,982511	7948,121223	6547,507956	13328,39574	13232,70141	11926,37747	13733,42388	7358,97476	7542,467498	4524,539765	7646,928442	7043,484745	18636,15575	10441,1442	7378,429389	10121,78572	19987,29664	23161,27139	9439,88581	5636,268751	6149,366798	14177,6597	14686,38581	9928,839665	4169,569118	16253,15133
CHUK	418,1829311	0,203561112	0,114582356	1,776548496	0,075642556	0,172998447	385,7137275	383,1867615	386,3485145	446,28511	524,9810641	425,3030166	469,5561726	396,6998771	452,7904616	430,739962	538,5440769	382,4279557	388,7535711	440,8527975	472,8494924	407,7595088	409,2861274	366,5812739	363,5334781	529,5361438	439,9756161	373,883431	326,0191353	383,984396	340,6626683	406,501868
ACTA2	1573,976737	0,716110735	0,404136341	1,771953328	0,076402305	0,173890551	896,6591848	1256,371925	920,3261363	770,856099	2101,462287	730,8605237	2073,299958	3230,662346	1709,868378	1294,580105	2386,684267	1113,232063	3314,161718	1203,427179	1519,4594	1620,649258	792,0717142	858,9397003	2603,658729	732,9016016	795,0436572	1297,375506	3885,957019	765,1608622	1554,273424	1495,452128
SEMA7A	25,32116382	0,737723651	0,416750589	1,770180222	0,076697123	0,173938118	22,89951629	17,35689163	14,13470175	44,76841228	13,84227415	20,64577751	13,75530732	35,11589507	21,64390352	21,24197073	33,78320777	7,858108678	28,51860174	12,11503871	14,31844173	46,23005896	38,27855868	27,95047836	16,97821189	22,59616198	28,30252501	16,8247544	50,15679005	15,44361373	56,77711139	16,81394588
GUSB	636,4181941	-0,206838544	0,11811555	-1,751154223	0,079919348	0,18020488	685,554269	636,8644084	794,6843429	540,0189732	602,395264	512,0152822	636,4955841	704,5126448	600,8347617	562,5188545	645,8554428	708,5394658	732,4777711	624,5975513	654,5573362	629,5599041	683,1250472	813,7889276	714,0836176	627,7803263	581,4882412	552,4127693	567,2494112	484,3678852	542,9311277	708,1638382
ADGRL4	206,3879882	0,418958282	0,239498962	1,749311469	0,080237195	0,18020488	145,9844164	125,503678	172,7574659	304,9848087	162,0058752	144,5204425	158,8112754	218,9256583	215,573279	181,7368607	180,839524	165,0202822	208,6360864	127,8809642	167,7303174	387,5013931	341,5625236	258,0044156	165,7872455	167,9975521	249,5768115	188,8111327	248,3955317	204,9788732	294,5312653	178,0300152
CASP8	251,8691976	0,30487163	0,174487467	1,747240851	0,080595568	0,18020488	169,5995425	193,596099	218,3026159	222,4430485	181,4875944	200,9522344	250,7217379	262,2718413	240,6802071	240,3489651	264,3039196	212,1689343	331,7163676	225,4743316	220,5721857	351,6601114	297,3949559	350,4559979	300,6142223	233,8211544	247,0038547	232,7424358	404,8369482	188,1312946	259,0455707	248,2529656
IRF1	139,9741735	0,445147088	0,25454981	1,748762208	0,080332132	0,18020488	115,9288012	62,75183899	114,6481364	149,6943786	104,0733945	114,2399689	103,1648049	138,8175227	120,3401036	103,062895	153,0180587	94,29730414	167,3591629	104,9970022	82,1605823	194,2701354	294,4504514	154,8026494	110,8577364	141,4716228	123,5019273	196,2888013	223,3171366	117,2310679	202,2684593	152,3145685
HLA-DMA	546,757005	0,620484776	0,359274931	1,727047235	0,084159181	0,18751256	330,6117664	369,8353064	457,0220233	858,9939107	658,7897142	699,2036649	638,3713078	464,7369238	327,2558212	204,5523107	363,6662955	286,8209668	827,0394505	421,3341241	340,9152793	511,6472818	909,8518948	1213,695772	374,5193799	376,275219	416,8190047	890,7772744	472,9068776	520,8709722	255,4970013	1023,672587
CD1D	29,6842086	0,561051444	0,327881688	1,711139915	0,087055287	0,193287089	22,89951629	16,02174612	15,70522417	36,37433498	28,70990194	22,02216267	32,51254457	24,14217786	21,64390352	21,24197073	39,74495032	22,26464125	29,26909126	28,94148137	21,13674731	33,24408734	35,33405417	35,47560715	20,97308527	20,63127833	36,02139547	18,69417155	62,09888291	17,54956106	74,5199587	34,6169474
LGALS3	738,9972126	-0,328974603	0,192624533	-1,707854121	0,087663414	0,193959122	850,1445423	775,7195415	1253,276889	689,7133518	570,0966243	834,0894113	898,4716644	713,2916185	544,5606125	724,1938539	574,3145322	703,3007267	594,3876995	916,0315381	682,1714738	678,3871574	441,6756771	876,1399947	981,7401345	564,9040495	650,9580753	816,9352968	733,2445021	786,9223179	496,7997247	862,456518
COL4A4	851,169267	0,265035285	0,155435147	1,705118112	0,088172398	0,194407892	660,507923	789,0709966	739,7160583	814,2254984	1056,626927	673,0523467	1052,906251	857,5959998	785,2408196	913,0113715	924,070095	739,9719005	908,8428082	774,6894198	954,2218667	1393,654474	762,6266691	678,3366094	882,8670182	658,2360229	854,221664	814,131171	995,9705452	906,9613155	734,5538786	805,0912909
BMPR1A	447,4298614	0,222345486	0,130558926	1,703027832	0,08856286	0,194593135	398,5947054	440,5980184	387,919037	520,4327928	449,6175715	381,2586913	516,4492657	435,1078873	469,2398283	534,1962268	580,2762747	388,9763796	536,6000065	321,7215835	478,9859674	420,2260416	544,7333351	440,7575434	358,5398863	496,1331217	437,4026593	392,5776026	470,518459	384,6863784	464,8625995	382,7645326
CD34	152,7149758	-0,336568293	0,198972297	-1,691533436	0,090734967	0,198678291	163,1590536	149,5362972	229,2962729	118,9160951	166,6199666	150,0259832	103,7900461	176,1281612	120,3401036	171,1158753	113,2731084	187,2849235	134,337624	152,7840993	156,1391979	147,5206376	120,7246851	219,3037533	113,8538915	143,4365065	164,6692364	99,07910922	218,5402995	193,7471541	113,5542228	143,4130678
IL33	85,77943619	0,775578117	0,459889313	1,686445182	0,091710085	0,200123382	57,24879073	45,39494735	39,26306042	156,689443	24,09581056	39,91516985	110,0424586	115,7727165	29,43570878	47,99111905	107,3113659	53,69707597	207,8855969	70,67105915	36,81885016	41,55510918	185,5037844	144,0524654	85,88977779	43,22744031	72,04279094	112,1650293	169,5777187	47,73480608	74,5199587	111,7632873
GBP4	214,5287554	-0,377520708	0,225228855	-1,67616493	0,093705891	0,203778223	216,114185	218,9638637	270,1298557	251,8223191	243,0088129	129,3802057	213,2072634	215,6335431	265,7871352	289,9135635	117,2476035	366,7117383	256,6674157	160,1877341	161,5938424	134,534666	279,7279288	251,5543052	138,8218501	214,1723179	169,8151501	214,0482643	224,5113459	177,6015579	180,9770426	215,6141295
TANK	435,7774052	0,270256057	0,161750079	1,670824891	0,09475627	0,205359153	378,5576287	336,4566686	395,771649	404,3147234	507,0373754	331,7088253	475,1833437	425,2315418	408,6368984	487,7785871	556,4293045	375,8795318	460,0500755	339,8941416	400,9163684	648,7791421	356,2850462	532,1341072	454,4168476	435,2217286	398,808307	402,8593969	535,0057605	319,4020113	550,0282666	413,4252575
STAT5A	269,239718	-0,263589141	0,158089382	-1,667342477	0,095446325	0,206151076	222,5546739	323,1052135	318,8160506	327,3690148	287,6116962	236,7382487	278,2323526	248,5546948	200,8554247	237,9887461	204,6864942	365,4020535	195,8777646	267,8769671	316,3693792	259,7194324	273,8389198	284,8798756	266,6577985	214,1723179	241,857941	218,7218071	379,7585532	292,024696	262,5941402	273,9684123
RHOJ	78,01987777	0,496962498	0,299960936	1,656757393	0,097568534	0,210020404	52,95513142	65,42213001	73,81455359	113,3200436	56,39445024	52,30263635	75,65419025	93,82528213	121,2058597	122,7313864	109,2986134	56,31644553	102,0665747	50,47932796	51,47820717	80,51302404	100,1131535	90,30154547	47,93848062	37,33278936	41,16730911	68,23372616	137,334068	48,43678852	95,81137547	84,06972939
IL15	99,12919476	0,289597742	0,176266922	1,64295001	0,100393277	0,215370712	83,72635644	80,10873062	87,94925534	120,315108	95,87056541	67,44287319	106,9162523	102,6042559	128,997665	109,3568123	77,50265313	82,51014112	114,8248965	87,4975018	83,52424342	105,9655284	123,6691896	136,5273366	99,87183463	103,1563916	108,0641864	113,0997379	111,0614637	65,28436714	81,61709762	99,89461963
HK2	47,98672869	0,94106975	0,573679552	1,640410132	0,10091992	0,215505672	30,05561513	14,68660061	23,55783625	125,9111595	30,2479324	28,90408851	29,38633836	35,11589507	32,03297721	22,42208021	25,83421771	24,88401081	35,27300742	14,13421183	29,6596293	50,38556988	73,61261284	22,57538637	34,95514212	26,52592928	69,46983412	44,86601172	83,59465008	42,82092898	283,8855569	12,85772332
TM4SF1	815,1778379	0,397453957	0,242442198	1,63937615	0,101134945	0,215505672	709,1693951	590,1343156	794,6843429	933,1415936	506,5246986	571,1998443	752,1652138	960,7489416	948,8687302	1093,568123	880,3506497	493,7511619	940,363368	535,7539341	544,1007857	856,554688	859,795318	1239,496213	841,919566	753,5328799	563,4775434	937,5127033	1485,596353	566,499831	809,0738373	1026,639754
DEFB1	2646,001444	-0,792692278	0,485632205	-1,632289353	0,102618545	0,217935706	2926,128816	3618,244333	6145,454217	2820,409974	3239,092151	4414,067231	1707,533831	497,1093895	1160,978985	3476,209173	765,0902937	3498,168047	2016,565339	5609,935981	2755,618202	1882,965885	1987,540547	1458,799967	3606,371949	726,0245088	1142,392828	2186,283363	2352,592295	6343,815332	801,9766984	1656,668197
LCP2	112,6955933	0,528325888	0,324212982	1,629564258	0,103193624	0,218426503	72,27659829	61,41669347	87,94925534	93,73386322	84,59167537	82,58311003	103,1648049	113,5779731	77,05229653	71,59330875	101,3496233	60,24549987	175,6145476	50,47932796	91,36529485	138,170738	135,4472076	287,0299124	110,8577364	101,191508	118,3560137	118,7079893	253,1723688	73,00617401	85,16566708	181,9862377
FCER1A	29,56625681	-1,076963844	0,665006151	-1,619479521	0,105344129	0,222237615	57,24879073	36,04892878	29,83992592	23,78321903	7,177475486	11,01108134	37,51447451	7,681602046	9,523317548	9,047506051	5,961742548	18,33558692	84,05482619	10,09586559	13,63661117	6,752705242	97,16864896	84,92645347	4,993591732	11,7893019	15,43774092	20,56358871	22,68997645	16,14559617	28,38855569	98,90556399
ACVRL1	469,9457235	0,452668762	0,281190396	1,609830095	0,107434953	0,225897998	357,804942	341,7972506	394,2011266	464,4722775	466,0232297	410,1627798	398,278671	584,3504413	365,3490914	432,3134413	341,8065728	258,0079016	547,8573493	326,4329875	993,0862085	551,1246355	406,3416229	549,3344016	449,4232559	268,2066183	401,3812638	295,3679105	1037,76787	668,2872851	503,8968636	405,5128124
HEG1	517,8054231	-0,438739575	0,275160888	-1,59448379	0,110827719	0,232262711	696,2884172	520,706749	808,8190447	622,5607333	356,3103902	418,4210908	442,0455579	520,1541957	383,5299703	470,4703147	290,1381374	502,9189554	651,424903	377,5853732	406,0300976	398,4096093	559,4558576	1173,920091	396,4911835	366,4508008	393,6623934	540,2615578	681,8935028	584,049392	259,0455707	641,8971103
RXRA	947,5284017	-0,284566896	0,178680995	-1,592597439	0,111250529	0,232381861	1246,592418	801,0873062	1317,668308	1038,06756	1325,269581	938,694684	774,6738985	745,6640843	984,364732	759,5971385	1150,616312	1190,503465	652,1753925	971,8953277	1240,931617	729,2921661	765,5711736	852,4895899	1015,696558	977,5296161	771,8870458	670,1860501	866,9959422	1115,450101	929,725199	803,1131796
PSMB9	223,0758676	0,484496306	0,306767448	1,579360229	0,114253454	0,237871946	180,3336908	153,5417337	175,8985107	485,4574707	217,8876487	181,6828421	213,8325047	244,7138937	177,4800089	153,414233	174,8777814	155,8524888	330,9658781	172,9758305	110,1156352	228,5531005	267,9499108	305,3052252	177,7718657	111,0159262	156,950366	385,0999339	380,9527625	201,468961	198,7198899	257,1544664
ARG2	794,4544716	0,600188265	0,380954749	1,575484403	0,115144676	0,238944017	407,8976339	502,0147119	1155,904499	892,5702199	1504,706468	711,5911314	643,3732378	583,2530696	1264,003965	1597,474873	1287,73639	471,4865207	281,4335698	664,307956	572,0558386	663,8428692	385,7300913	392,3817154	681,1259122	1530,644364	764,1681754	282,2819904	484,8489704	529,996744	1355,553534	1045,431811
KIT	138,1953377	0,502171821	0,321383076	1,562533496	0,118162361	0,24440749	77,28586748	110,8170774	135,0649278	83,94077303	125,0931442	145,8968277	171,3161002	155,8267844	170,5539597	94,80212862	216,6099793	90,3682498	72,04699388	85,47832868	112,8429574	254,5250437	94,22414444	119,3270422	194,7500775	88,41976427	331,9114297	118,7079893	167,1893002	142,5024358	127,7485006	105,8289535
MBP	360,0690519	0,217950888	0,140276658	1,553721702	0,120250814	0,247117369	278,3722449	368,5001608	273,2709005	254,6203449	319,3976591	382,6350765	350,7603366	358,8405527	333,3161142	401,6305947	369,628038	306,4662384	384,250634	308,2604294	341,2561946	355,8156224	421,0641455	462,2579113	410,4732403	425,3973103	398,808307	379,4916825	435,8863897	261,1374686	440,0226133	340,2351401
TPSAB1/B2	131,8661569	0,920533506	0,591898009	1,555223186	0,119892924	0,247117369	84,44196632	42,72465633	39,26306042	86,7387988	41,52682245	28,90408851	261,3508391	72,42653358	38,95902633	54,67840614	33,78320777	27,50338037	334,7183257	53,17155879	196,0262856	195,3090131	85,3906309	457,9578377	126,83723	163,085343	74,61574776	58,88664038	360,6512046	29,48326258	117,1027922	362,9834198
STK11IP	106,8516055	0,297986519	0,19292625	1,544561813	0,122452289	0,250023162	85,15757621	104,1413498	94,23134501	107,7239921	115,3522846	114,2399689	78,15515522	96,02002557	99,56195618	101,0960459	143,0818212	70,7229781	131,3356659	88,17055951	95,11536292	150,6372708	114,835676	120,4020606	91,88208786	106,1037171	84,90757504	104,6873607	158,8298351	80,72798087	149,0399174	91,98217451
BCL2L11	96,44178468	-0,329124518	0,213007157	-1,545133611	0,122313949	0,250023162	100,1853838	134,8496965	135,0649278	69,95064419	111,25087	70,19564352	85,03280888	98,76345488	101,2934685	107,783333	103,3368708	106,0844672	84,80531571	90,86279033	142,5025867	129,3402773	50,05657673	95,67663746	117,8487649	56,98162586	84,90757504	69,16843474	91,95411508	95,46961216	102,9085144	71,21200607
CRHBP	274,3288933	-0,516873417	0,335522936	-1,540500992	0,123438281	0,251228553	374,9795793	432,5871453	628,2089667	300,78777	455,2570165	367,4948396	148,8074155	265,5639564	399,979337	175,0495736	220,5844743	218,7173582	136,5890926	249,0313513	243,4135094	268,0304542	82,44612639	480,5332241	262,6629251	144,4189483	167,2421933	143,9451209	240,0360666	320,1039937	124,1999312	281,8808574
BMP7	102,6114417	-0,448358024	0,29196552	-1,535654018	0,124623258	0,252829933	100,1853838	148,2011516	221,4436608	131,5072111	117,9156687	88,08865069	72,52798404	88,33842353	118,6085913	112,8971407	95,38788078	95,60698892	90,05874235	93,55502116	185,1169966	59,21603058	38,27855868	67,7261591	120,8449199	55,01674221	115,7830569	91,6014406	77,62360364	143,9064007	60,32568085	78,13539555
CSF3R	38,58152525	0,749813975	0,49090414	1,527414242	0,126658037	0,256139661	20,75268664	21,36232817	23,55783625	34,9753221	20,50707282	33,03324401	31,26206209	30,17772232	22,50965966	13,37457416	83,46439568	19,6452717	33,02153886	14,13421183	15,00027229	41,55510918	61,83459479	51,60088312	35,95386047	36,35034753	82,33461822	17,75946297	51,35099933	16,84757862	156,1370563	34,6169474
CASP3	157,2491673	0,309670362	0,203847476	1,519127772	0,128730337	0,259504014	138,1127076	134,8496965	147,6291072	198,6598295	138,9354183	114,2399689	130,0501783	210,1466845	142,8497632	121,9446468	186,8012665	96,9166737	181,6184637	104,9970022	133,9797048	226,475345	135,4472076	204,2534957	160,7936538	149,3311574	192,9717615	148,6186638	217,3460902	124,9528747	177,4284731	169,1285144
FOSL1	55,33811899	0,682504825	0,452586693	1,50800904	0,131552213	0,26435334	45,0834227	33,37863776	25,12835867	68,55163131	27,17187148	30,28047368	28,76109712	56,51464362	25,10692808	40,91046214	103,3368708	23,57432603	52,53426637	32,9798276	35,45518904	44,1523035	88,33513541	50,52586473	24,96795866	62,87627681	118,3560137	40,19246883	85,98306865	38,60903433	216,4627372	39,5622256
CFH	595,3311389	0,42677067	0,284242209	1,501433135	0,133243569	0,266907465	437,2376392	313,7591949	535,5481441	654,7380297	458,3330774	540,9193707	697,1439846	462,5421803	578,325102	407,924512	572,3272847	436,1250316	753,4914776	497,3896449	597,9653998	477,8837556	627,1794614	795,5136148	771,0105634	223,0142943	439,9756161	662,7083815	1041,350498	524,3808844	571,3196834	1400,502786
HLA-A	7871,842827	0,394881448	0,264230698	1,494457122	0,135056181	0,269687657	7010,114424	6276,519044	6792,509453	15103,74309	6379,237676	8528,082495	13864,72454	6154,609296	6425,642077	6360,396754	8652,475685	6021,930617	9810,398999	5633,493001	6380,911282	9945,176505	8418,338405	10137,4235	11725,9521	4114,466364	6036,156698	7629,09141	8376,183938	4675,905049	5099,294317	9115,136777
CD69	44,54004897	0,791082487	0,530718284	1,490588342	0,136069607	0,270013126	25,76195583	22,69747368	12,56417933	72,74866996	18,96904235	19,26939234	31,88730333	23,04480614	19,91239124	12,58783451	35,77045529	18,33558692	44,27888165	10,09586559	17,72759452	121,0292555	41,22306319	64,50110391	32,95770543	36,35034753	46,31322275	37,3883431	139,7224866	32,99317479	74,5199587	145,3911791
DDX50	288,0796862	0,206001401	0,138051389	1,492208103	0,135644599	0,270013126	254,7571187	229,6450278	276,4119454	428,0979425	316,834275	262,8895669	265,7275278	277,6350454	307,34343	289,9135635	270,2656622	267,1756951	334,7183257	215,378466	248,5272386	283,6136202	315,061983	301,0051516	285,6334471	239,7158053	295,8900342	296,3026191	305,7175774	255,521609	390,3426408	275,9465235
ARRB2	95,8081976	0,273329633	0,184226265	1,483662673	0,137898434	0,271100636	70,84537852	76,10329409	86,37873293	88,13781168	96,38324223	83,95949519	98,7881162	90,53316697	69,26049126	80,24744498	95,38788078	74,65203244	108,0704908	95,57419428	78,75142951	99,21282317	94,22414444	156,9526862	87,88721448	120,8403445	92,6264455	82,25435482	134,9456494	85,64185797	102,9085144	140,4459009
EDA	41,04015469	-0,45773988	0,308427621	-1,484107937	0,137780288	0,271100636	32,91805467	64,0869845	43,97462767	29,37927056	28,19722512	33,03324401	39,39019823	37,31063851	35,49600177	30,28947678	47,69394039	57,62613031	36,02349694	26,24925054	25,22773067	29,60801529	50,05657673	37,62564394	28,96283204	44,20988213	38,59435229	20,56358871	62,09888291	31,58920991	124,1999312	32,63883612
RTN4	2634,85652	-0,186982044	0,125917364	-1,484958368	0,137554853	0,271100636	3195,198132	3037,456036	2946,300054	2582,577784	2456,747323	2660,552528	2909,247498	2968,939191	3148,755084	3147,745366	1923,655596	2911,429265	2490,124226	2877,994752	2495,158929	1949,973498	2096,487214	2113,486171	2792,416496	2623,119673	3000,067651	2537,733788	2430,215899	2833,201137	2004,941746	2372,74448
PDGFRB	723,3114578	0,368592707	0,249295526	1,47853719	0,139264051	0,272940346	715,6098841	550,0799503	804,1074774	1024,077431	760,2997246	545,0485262	778,425346	855,4012564	568,8017845	863,0534033	663,7406704	352,3052057	1036,426026	578,1565696	646,0344542	754,2252316	562,4003621	1050,292975	826,9387908	397,8889392	488,8617957	638,4059585	1035,379452	628,9762684	535,8339887	1145,326431
CD8A	35,43698445	0,778031381	0,528141842	1,473148535	0,140710989	0,274927624	22,89951629	18,69203714	12,56417933	65,75360554	30,76060922	16,51662201	25,00964967	13,71714651	23,3754158	9,834245708	33,78320777	17,02590214	27,76811222	12,78809642	17,72759452	25,97194324	100,1131535	97,82667426	23,96924031	12,77174373	36,02139547	42,99659457	106,2846265	23,86740304	39,03426408	64,28861659
CD72	32,55924983	0,657048634	0,448963931	1,463477549	0,143336753	0,279198889	14,31219768	24,03261919	17,27574659	61,55656689	13,32959733	24,77493301	22,5086847	22,49612028	23,3754158	20,45523107	29,80871274	19,6452717	40,52643406	16,15338495	14,65935701	17,14148254	94,22414444	69,8761959	18,97564858	28,49081293	61,75096367	30,84538306	46,57416219	24,56938548	67,42281978	21,75922408
CGAS	29,38873281	0,503282983	0,346619625	1,451974866	0,146508594	0,284504456	13,5965878	21,36232817	12,56417933	25,18223191	19,48171918	37,16239951	21,88344346	23,04480614	21,64390352	20,06186124	17,88522765	26,19369559	37,52447598	20,86478889	18,0685098	33,76352621	41,22306319	51,60088312	14,9807752	23,5786038	59,17800685	25,23713159	47,76837147	21,05947327	56,77711139	52,41994892
MTOR	422,1534776	0,175165995	0,1208683	1,44923024	0,147273295	0,285117507	354,2268926	405,8842351	427,1820974	339,9601308	454,2316629	390,8933875	432,0416981	432,9131439	495,2125125	527,1155699	445,1434436	364,0923688	437,5353899	404,5076814	434,3260658	550,6051966	488,7877493	399,9068442	359,5386047	368,4156844	447,6944866	403,7941055	363,0396232	372,0506944	468,411169	408,4799793
LAP3	1416,727314	0,428494701	0,296143893	1,44691385	0,147921054	0,285501122	1040,496771	1020,05117	1232,860097	2683,306711	1642,616533	2057,695825	1190,459324	1072,680857	722,0406214	857,1528559	2342,964822	1198,361573	1416,173723	1371,018547	947,0626458	1491,308981	2317,325052	1476,000261	1112,572238	1223,140072	885,0971459	1959,149178	1546,501026	1932,557664	667,1310588	1429,1854
IFI6	501,9110581	0,470445334	0,327473236	1,436591706	0,150834042	0,290241262	324,8868874	345,8026872	486,8619492	477,0633934	274,2820989	291,7936554	647,1246852	767,0628329	656,2431547	597,1353994	4799,202752	362,782684	467,5549707	296,1453907	581,6014664	1046,669312	800,9052278	657,9112598	460,4091577	342,872197	344,7762138	357,0586766	778,624455	329,9317479	198,7198899	670,5797239
HLA-DMB	187,1986054	0,486433038	0,34031147	1,429375973	0,152896201	0,292684676	129,525389	149,5362972	146,0585848	344,1571694	174,8227958	152,7787535	178,8189951	167,3491874	232,0226457	127,4518244	85,45164319	117,8716302	276,9306327	148,0726954	95,4562782	132,4569105	279,7279288	385,931605	126,83723	103,1563916	156,950366	295,3679105	261,5318338	193,7471541	106,4570839	298,6948033
MMP9	27,11269745	0,755816414	0,528940445	1,428925356	0,153025689	0,292684676	10,73414826	14,68660061	15,70522417	60,15755401	6,152121845	13,76385167	27,51061464	24,69086372	17,31512281	10,22761554	15,89798013	17,02590214	48,78181877	14,80726954	8,522881982	32,20520961	44,16756771	45,15077273	7,989746771	17,68395285	82,33461822	31,78009164	59,71046434	16,14559617	39,03426408	22,74827972
NFAM1	32,04863973	0,729643758	0,513684622	1,420411915	0,155487796	0,296500752	10,01853838	12,01630959	25,12835867	68,55163131	12,81692051	28,90408851	26,8853734	17,55794753	21,64390352	11,01435519	39,74495032	26,19369559	25,51664366	14,13421183	8,863797261	20,25811573	85,3906309	53,75091992	13,98205685	24,56104563	59,17800685	28,04125733	53,73941791	19,65550839	85,16566708	40,55128124
TGFBR2	1237,714766	0,224992447	0,15871719	1,417568238	0,156316859	0,297189237	979,6699313	1132,203393	1399,335473	1625,652971	1206,841235	1381,890708	1317,383296	1541,258582	1205,132548	937,4003009	1446,716192	977,0248457	1247,313581	960,4533467	931,7214583	1120,429631	1433,973698	1620,052726	1070,626067	1146,50961	874,8053186	1264,660705	1274,221309	1239,700993	1256,193589	1589,412413
BMP6	103,6982115	0,713300577	0,505322567	1,411574751	0,158075211	0,299635102	46,51464247	80,10873062	102,0839571	85,33978592	72,8001085	38,53878468	41,8911632	177,2255329	177,4800089	403,204074	93,40063326	69,41329332	219,1429397	22,88396201	105,001906	106,4849673	153,1142347	49,45084633	56,92694574	121,8227863	66,8968773	86,92789771	106,2846265	32,29119235	138,394209	42,52939252
CITED4	110,5644413	0,48600157	0,345573045	1,406364231	0,159615996	0,301655231	70,12976864	112,1522229	56,53880701	142,6993142	58,44515753	70,19564352	91,2852213	117,96746	124,6688843	109,7501821	147,0563162	77,271402	114,074407	72,01717456	54,2055294	58,17715285	253,2273882	130,0772262	112,8551731	69,75336959	156,950366	110,2956121	154,052998	73,00617401	244,8512929	92,97123015
MYL9	1892,859891	0,356354802	0,255886642	1,392627606	0,163732431	0,308516598	1437,660257	1675,607615	2076,230635	1308,077046	2300,89357	1318,57699	2717,298437	2119,573479	2618,912326	2046,309847	1862,050923	1191,81315	2944,170385	1780,910691	2281,405049	1457,026016	1174,857301	1628,652874	2710,521592	1073,808915	1325,072762	1666,585394	3155,100936	1930,451716	1355,553534	2057,235731
TRAF4	451,24535	0,232235226	0,167761633	1,384316672	0,166261511	0,312355206	429,3659304	413,8951082	372,2138128	458,8762259	510,1134363	503,7569712	407,0320484	523,9949967	248,4720124	380,388624	534,5695818	336,5889884	444,2897956	393,0657004	531,1460051	462,3005896	532,955317	460,1078745	468,3989044	565,8864913	504,2995366	427,1618199	611,4351548	416,2755883	468,411169	327,3774168
TEK	226,6376515	-0,31595273	0,229873079	-1,374465995	0,16929706	0,317119862	249,0322397	261,68852	348,6559765	193,063778	243,5214897	205,0813899	191,32382	260,0770978	212,9760106	309,5820549	158,9798013	250,1497929	202,6321703	190,4753308	311,5965653	134,534666	159,0032437	275,20471	251,6770233	148,3487156	234,1390706	196,2888013	290,1928567	317,9980464	106,4570839	189,8986829
TNFRSF17	33,27590803	0,820513173	0,59805737	1,371963985	0,17007465	0,317639419	16,45902733	12,01630959	12,56417933	78,3447215	15,38030461	27,52770334	14,38054856	12,61977479	32,03297721	12,58783451	41,73219784	30,12274993	37,52447598	16,82644265	5,113729189	20,77755459	64,7790993	29,02549676	20,97308527	14,73662738	64,32392048	10,28179435	53,73941791	22,46343816	163,2341952	35,60600304
CD81	4015,416001	0,297323981	0,217696549	1,365772592	0,172010351	0,318072117	4353,770535	2838,519355	4320,507169	5686,987373	4456,6996	3149,169262	4688,684072	4932,137199	3917,546537	5204,676198	3044,463195	2658,660103	5001,262158	4041,711526	4381,102254	2494,864867	3141,786316	3752,889229	5825,524114	2424,666425	3594,420677	4309,006542	5746,535088	3187,000288	2966,60407	4281,621865
IL27RA	51,28152723	0,417368772	0,30580521	1,364819036	0,172309934	0,318072117	35,06488432	34,71378327	42,40410525	64,35459266	36,40005425	30,28047368	45,64261065	55,96595776	46,7508316	50,74470785	53,65568294	37,98085861	49,53230829	16,82644265	43,29624047	60,25490831	55,94558576	79,55136148	37,95129716	41,26255666	79,7616614	50,47426319	93,14832437	34,39713968	88,71423655	68,24483915
LRRC32	101,4753007	0,39701635	0,290300517	1,367604696	0,171435846	0,318072117	62,25805992	64,0869845	94,23134501	135,7042497	65,62263301	119,7455095	66,90081287	91,08185283	99,56195618	79,85407515	109,2986134	81,20045634	110,3219594	76,7285785	50,11454605	78,43526858	132,5027031	115,0269686	76,90131267	64,84116046	123,5019273	119,6426979	179,131393	104,5953839	234,2055845	102,8617866

SFTPA2	39,68840047	0,841332998	0,616294257	1,365148204	0,172206474	0,318072117	15,74341745	17,35689163	29,83992592	99,32991476	12,81692051	26,15131817	18,75723725
ABCE1	422,4732388	0,203478732	0,149297016	1,362912245	0,172910169	0,318254949	397,8790955	419,2356902	376,92538	566,600218	482,428888	366,1184544	395,777706
INPP5D	126,0880871	0,405381028	0,298584475	1,357676175	0,17456646	0,31860996	77,28586748	100,1359133	100,5134347	130,1081982	99,45930316	85,33588036	140,6792794
NKG7	53,91852359	0,70608038	0,520115862	1,357544408	0,174608293	0,31860996	35,7804942	16,02174612	31,41044834	93,73386322	32,29863968	20,64577751	32,51254457
STAT4	50,18815621	0,544116457	0,400004495	1,360275857	0,173742646	0,31860996	30,77122502	30,70834674	18,846269	79,74373438	34,86202379	35,78601434	30,63682085
UBB	6833,69006	-0,284275078	0,21369582	-1,330279075	0,183426339	0,333741333	9307,222152	7878,693656	8435,275901	8920,106148	6780,663627	9462,648024	6719,467625
TNF	26,78273216	0,568715823	0,429877191	1,322972781	0,18584443	0,337174894	17,17463722	16,02174612	15,70522417	18,18716749	7,690152306	39,91516985	16,25627229
CD48	57,98578946	0,640732553	0,485222297	1,320492808	0,186670536	0,337708804	29,34000525	40,05436531	25,12835867	69,95064419	30,2479324	22,02216267	41,8911632
P2RX4	174,1026451	0,265344978	0,204303658	1,298777422	0,194020325	0,350008257	115,2131913	188,255517	127,2123158	267,2114608	187,1270394	244,9965597	165,0636878
CR1	180,3894292	-0,504082869	0,388932185	-1,296068797	0,194951784	0,35029379	182,4805204	144,1957151	365,9317231	254,6203449	93,30718131	99,09973203	142,5550031
FAS	215,9825619	0,376179784	0,290461391	1,295111141	0,195281892	0,35029379	205,3800367	122,8333869	147,6291072	230,8371258	142,5241561	184,4356124	219,4596759
CETP	48,07378559	-0,47201439	0,36585496	-1,290168076	0,196992308	0,352366522	68,69854887	34,71378327	81,66716567	75,54669573	35,88737743	48,17348085	53,14550555
IL18	284,8062831	0,315495351	0,247585806	1,274286907	0,202561755	0,361310995	191,0678391	186,9203714	326,6686627	246,2262676	300,4286168	196,8230789	368,8923326
CTNNB1	2024,459516	0,113785081	0,08940812	1,272648175	0,203142916	0,361332637	2034,4789	2014,734575	1916,037349	2316,765336	2419,834592	1829,215887	2110,189191
BASP1	158,0387713	0,529485481	0,419805162	1,261264816	0,207213453	0,364764651	140,2595373	84,11416715	120,9302261	275,6055381	88,18041311	90,84142103	101,2890812
HLA-DRA	3192,498756	0,499384253	0,395855535	1,261531567	0,207117394	0,364764651	2357,934568	2379,229299	2374,629894	5519,105827	4335,70787	3341,863186	3715,183459
HLA-DRB3	2557,676928	0,478747353	0,379007302	1,26316129	0,206531221	0,364764651	1704,582744	2080,156705	1573,663462	3721,374271	2507,502329	3081,726389	3092,443182
SOX7	45,22686739	0,30007526	0,237998211	1,260829895	0,20737014	0,364764651	50,09269189	21,36232817	37,692538	67,15261843	37,42540789	53,67902152	41,26592196
SELPLG	56,39717998	0,466065296	0,37344749	1,248007572	0,212028284	0,371928068	31,4868349	36,04892878	32,98097075	67,15261843	30,2479324	41,29155501	50,01929934
IRF6	221,1745975	-0,160222661	0,128619312	-1,245712314	0,212870031	0,37237595	240,4449211	244,3316284	230,8667953	254,6203449	291,200434	246,3729449	207,5800923
ZEB1	160,1489938	0,288348464	0,232661578	1,239347154	0,215216963	0,375447175	135,965878	136,1848421	164,9048538	169,280559	117,4029919	134,8857464	155,059828
CD55	224,920344	0,272114041	0,223015322	1,220158502	0,222404795	0,38692341	189,6366193	146,8660061	251,2835867	232,2361387	240,9581056	162,4134497	192,5743025
CD83	313,4807334	-0,425122407	0,352591568	-1,205707809	0,227930173	0,395452622	347,7864037	391,1976345	490,002994	286,7976412	401,9386272	225,7271674	264,4770453
GBP1	234,4981761	0,322761643	0,269137507	1,199244384	0,230432934	0,398705485	208,2424763	186,9203714	191,6037349	464,4722775	272,2313916	196,8230789	183,1956838
PTGER4	211,8483219	0,276936493	0,231968057	1,193856158	0,23253424	0,401247941	159,5810042	173,5689163	246,5720194	250,4233062	193,2791613	238,1146339	189,4480963
CX3CL1	186,3549668	-0,237532228	0,199765425	-1,189055754	0,234417728	0,403401781	204,6644268	229,6450278	245,001497	207,0539068	146,6255706	143,1440574	201,9529211
CD22	34,70440681	0,592915851	0,500074882	1,185654135	0,235758914	0,404613272	11,44975815	16,02174612	36,12201559	74,14768285	16,40565825	38,53878468	17,50675477
SPRY4	246,342201	-0,258633992	0,220159733	-1,174756114	0,240092302	0,410797252	246,88541	303,0780308	287,4056023	286,7976412	197,8932527	202,3286196	231,3392595
ST8SIA4	70,21320008	0,40951231	0,349011623	1,17334863	0,240656028	0,410797252	65,12049945	44,05980184	58,10932942	103,5269534	61,52121845	50,92625118	61,27364169
CARD8	161,9145976	0,304026953	0,261287678	1,16357172	0,244597618	0,414185299	110,919532	133,514551	150,770152	263,0144222	130,2199124	165,1662201	183,8209251
PRF1	33,59159571	0,57345313	0,492509063	1,164350412	0,244282035	0,414185299	15,02780757	13,3514551	29,83992592	44,76841228	20,50707282	31,65685884	17,50675477
TREM1	38,89205463	0,736824478	0,631953789	1,165946769	0,243635968	0,414185299	15,74341745	30,70834674	9,423134501	85,33978592	13,32959733	28,90408851	21,88344346
PTPN2	169,0194438	0,231054599	0,200309335	1,153488922	0,248709762	0,420028454	155,2873448	106,8116408	186,8921676	237,8321903	170,2087044	108,7344282	156,9355517
AGR2	81,63847984	0,59588624	0,52467463	1,135725279	0,256071558	0,430439105	57,24879073	53,40582041	25,12835867	117,5170822	32,29863968	34,40962918	29,38633836
PAX5	30,51601021	0,746328354	0,65735696	1,135347154	0,256229892	0,430439105	10,01853838	14,68660061	20,41679142	64,35459266	11,27889005	15,14023684	16,25627229
ITGA4	62,49825605	0,344215534	0,304574003	1,13015402	0,25841133	0,432958298	39,35854362	48,06523837	36,12201559	64,35459266	39,47611517	45,42071051	69,40177784
CCR5	57,23692705	0,579250408	0,518198306	1,117816098	0,263645545	0,440565582	20,75268664	36,04892878	45,54515009	86,7387988	35,88737743	49,54986602	42,51640444
COL13A1	28,28496496	0,351817553	0,316175102	1,112730098	0,265824365	0,443040608	22,18390641	25,3677647	14,13470175	26,58124479	16,91833507	41,29155501	31,26206209
BLNK	205,0214233	0,251964842	0,226924354	1,110347291	0,266849402	0,443584739	163,1590536	201,6069721	183,7511228	272,8075124	246,5975506	183,0592272	176,3180302
FAM30A	28,35411992	0,535302297	0,484163012	1,105624106	0,268889251	0,44580855	20,03707675	22,69747368	6,282089667	75,54669573	10,25353641	22,02216267	15,0057898
IL7R	79,49557769	0,667104119	0,604436372	1,103679643	0,269732129	0,446041412	27,90878548	29,37320123	45,54515009	41,97038652	73,31278532	19,26939234	80,65612019
KLF2	369,209837	-0,347545494	0,319360695	-1,088253812	0,276483075	0,454836146	322,7400577	666,2376097	394,2011266	270,0094866	328,6258419	290,4172703	345,7584067
TYK2	292,9275604	0,168148598	0,154364441	1,089296196	0,27602329	0,454836146	235,4356519	261,68852	254,4246315	183,2706878	254,2877029	316,5685884	298,2400723
NOS3	188,1929967	0,258518785	0,238325611	1,084729347	0,278041546	0,45621804	128,0941693	192,2609535	168,0458986	193,063778	125,605821	178,9300717	167,5646528
RGS5	1134,790335	0,336164562	0,313993685	1,070609306	0,284345137	0,465358665	1058,387019	810,4333248	728,7224014	593,1814628	750,5588651	646,9010285	1765,681267
CCL20	37,34217521	0,577630052	0,54097782	1,067751821	0,285632476	0,46626381	12,88097791	14,68660061	29,83992592	68,55163131	12,81692051	13,76385167	47,51833437
ITGAX	44,75625974	0,40891172	0,387658937	1,054823404	0,291506098	0,474631724	23,61512617	34,71378327	26,69888109	64,35459266	22,5577801	24,77493301	39,39019823
MARCH8.	120,4955829	0,181422508	0,172420899	1,052207186	0,292704503	0,475364091	113,0663617	90,7898947	119,3597037	135,7042497	147,6509243	97,72334686	150,057898
TLR3	122,9864122	0,205100897	0,195476326	1,049236505	0,294069281	0,476362229	132,3878286	120,1630959	111,5070916	222,4430485	135,3466806	129,3802057	128,7996958
REL	120,4057325	0,230233724	0,219843392	1,047262426	0,294978561	0,476619303	109,4883123	94,79533123	122,5007485	145,4973399	117,4029919	85,33588036	107,5414936
NOD1	92,07525581	0,266050995	0,25476886	1,044283805	0,296354103	0,477442657	66,55171922	80,10873062	87,94925534	134,3052369	87,15505947	86,71226553	71,9027428
PDPN	36,2033273	0,413808513	0,396784142	1,042905874	0,296991889	0,477442657	25,04634594	36,04892878	26,69888109	46,16742517	32,29863968	19,26939234	33,76302706
IMPDH2	429,9899902	0,217941031	0,210079637	1,037421018	0,299539687	0,480322478	412,1912932	452,614328	378,4959025	749,8709058	402,9639808	305,5575071	460,1775539
CD3E	32,2058824	0,462764592	0,447106492	1,035020962	0,300659122	0,48090313	18,60585699	17,35689163	20,41679142	55,96051536	17,43101189	20,64577751	22,5086847
GAPDH	8576,193822	0,214360347	0,207804414	1,031548574	0,302283642	0,481763411	6750,348037	8215,150325	7372,032225	6278,769823	7983,403447	10808,75272	8689,602778
HLA-F	139,5902128	0,261188243	0,253425795	1,030630064	0,302714332	0,481763411	114,4975815	134,8496965	111,5070916	146,8963528	116,8903151	128,0038205	203,2034036
PIN1	279,834649	0,124957108	0,123339128	1,013118139	0,311003735	0,493718429	244,7385804	300,4077398	234,0078401	288,1966541	253,2623493	300,0519664	275,7313876
CCR10	32,15166372	0,464181811	0,459723204	1,009698461	0,31263978	0,495077956	20,75268664	21,36232817	23,55783625	79,74373438	16,40565825	19,26939234	24,38440843
NRDE2	170,8045877	-0,146774605	0,145841443	-1,006398469	0,31422393	0,496348745	161,0122239	206,9475541	186,8921676	200,0588424	193,2791613	111,4871985	137,5530732
TCF7	31,16976561	0,414376089	0,414910887	0,998711054	0,317934684	0,50096408	23,61512617	25,3677647	18,846269	29,37927056	15,89298143	12,3874665	35,63875078
IGKC	2369,38394	-0,777897718	0,78288808	-0,993625701	0,320405127	0,503607068	1208,665094	1133,538538	577,9522494	72,74866996	351,6962988	19,26939234	525,8278843
CXCL12	659,9217669	-0,509298081	0,522266845	-0,975168318	0,329476775	0,516587042	682,6918294	1097,489609	665,9015047	320,3739504	338,8793783	412,9155501	805,9359606
LCK	25,34630046	0,467524575	0,480551892	0,972890926	0,330607515	0,517083183	16,45902733	12,01630959	14,13470175	39,17236075	15,89298143	9,63469617	19,38247849
CD68	383,3500115	-0,237185062	0,244363039	-0,970625767	0,33173467	0,517571291	404,3195845	360,4892878	354,9380662	353,9502596	344,0061465	280,7825741	322,6244808
SMAD2	1055,691634	0,113701689	0,117698201	0,966044405	0,334021962	0,518591554	979,6699313	903,8935105	1162,186588	956,9248126	1256,05821	823,0783299	1113,554652
XAF1	142,2732398	-0,277942855	0,287623174	-0,966343746	0,333872203	0,518591554	164,5902733	172,2337708	190,0332124	104,9259663	105,0987482	118,3691244	133,8016257
HLA-C	3663,533556	0,343828748	0,357003213	0,963097067	0,335498814	0,519614016	3029,176639	4165,653992	2302,385863	4801,412218	1898,442266	6514,430996	2922,377564
CD45RB	34,92151469	0,368078742	0,384923896	0,956237701	0,338952157	0,523685205	25,76195583	16,02174612	18,846269	30,77828345	28,19722512	23,39854784	34,3882683
NPHS2	412,382686	-0,390633289	0,409305581	-0,95438056	0,339891043	0,523861194	390,7229967	369,8353064	1126,064573	604,3735658	509,6007595	707,4619759	408,2825309
RAMP3	98,19682672	-0,289220918	0,306517035	-0,943572087	0,345388362	0,530005594	78,00147736	114,8225139	116,2186588	116,1180694	57,41980388	92,2178062	68,77653659
RELA	216,1303095	0,178933853	0,189696893	0,943261909	0,345546954	0,530005594	168,8839326	214,9584272	199,4563469	219,6450228	156,3664302	218,8452416	200,7024386
LRP2	3499,359897	-0,315008848	0,339807804	-0,92702064	0,353915818	0,541533842	4053,214383	5193,716035	3901,177683	4590,161272	2601,322187	6467,6339	3588,259486
TP53	208,2324378	0,191358146	0,208563221	0,917506668	0,3588772	0,547805342	177,4712513	166,8931888	197,8858245	232,2361387	174,8227958	156,907909	145,0559681
BTK	44,78746872	0,452598836	0,496944743	0,910762901	0,362420314	0,548638152	22,18390641	30,70834674	34,55149317	68,55163131	26,14651784	42,66794018	33,13778581
PROX1	85,44512927	0,429900901	0,471953338	0,910897047	0,362349622	0,548638152	61,54245003	96,13047674	56,53880701	79,74373438	121,5044064	59,18456219	43,76688692
SLC4A1	100,9813525	-0,436359573	0,47738717	-0,914058023	0,360686358	0,548638152	102,3322134	94,79533123	185,3216452	100,7289276	58,44515753	200,9522344	190,0733375
SP140	28,3170721	0,435833019	0,478993542	0,90989331	0,362878778	0,548638152	17,8902471	20,02718265	20,41679142	53,16248959	18,45636553	15,14023684	19,38247849
AIM2	28,77030649	0,447505355	0,498985022	0,896831239	0,369808976	0,551478468	18,60585699	16,02174612	21,98731384	75,54669573	12,81692051	24,77493301	18,13199601
BATF3	34,99729459	0,366062095	0,410337994	0,892098953	0,372339896	0,551478468	22,89951629	28,03805572	23,55783625	51,7634767	17,94368871	26,15131817	23,13392595
FABP1	2048,282982	-0,675893985	0,758015143	-0,891662905	0,372573642	0,551478468	2500,340935	2779,772952	3510,117602	565,2012051	5901,935556	3220,741291	677,7615061
ICOSLG	161,1676411	0,173244264	0,193670233	0,894532223	0,371037197	0,551478468	130,9566088	136,1848421	193,1742573	151,0933915	183,0256249	209,2105454	108,7919761
IFI27	562,5128715	-0,218854968	0,245365019	-0,89195668	0,372416152	0,551478468	553,1664404	627,5183899	733,4339687	502,2456253	289,1497267	400,5280836	515,8240245
MAPK3	469,284041	-0,128462098	0,143989081	-0,892165551	0,372304204	0,551478468	526,6888747	538,0636407	540,2597114	436,4920198	431,6738828	337,2143659	533,9560205
NOTCH1	322,3283159	0,132995508	0,14867272	0,894552198	0,371026515	0,551478468	267,6380966	292,3968668	299,9697816	300,78777	287,6116962	378,505921	268,853734
RORA	376,6613814	-0,207025511	0,230076065	-0,899813334	0,368219597	0,551478468	453,6966665	321,770068	471,156725	225,2410743	487,5556562	251,8784856	495,1910635
RPS6	12151,6703	-0,128348784	0,142138901	-0,902981403	0,366535765	0,551478468	11742,44259	13622,48964	13861,43085	12707,23402	11264,02242	12044,7466	11961,4902
CD4	111,033046	0,317218279	0,3580416	0,885981626	0,375627428	0,553418601	102,3322134	70,76271205	87,94925534	123,1131338	77,9268767	64,69010285	124,4230071
IGF1R	520,3061196	-0,158493795	0,178805796	-0,886401884	0,375401004	0,553418601	498,0644793	638,1995539	625,0679219	429,4969554	585,9896057	536,7902152	510,1968533
BMPR1B	134,3304007	-0,215462585	0,244738105	-0,880380212	0,378653373	0,555916663	126,6629495	185,5852259	212,0205263	142,6993142	111,7635468	118,3691244	86,28329136
CD274	25,58342736	0,326060004	0,370689551	0,879603979	0,379073882	0,555916663	15,02780757	20,02718265	21,98731384	54,56150247	22,5577801	24,77493301	25,00964967
PSTPIP1	51,29447698	0,386489583	0,44843238	0,861868143	0,388760081	0,568807953	22,18390641	33,37863776	31,41044834	29,37927056	25,1211642	22,02216267	47,51833437
ATM	251,2710686	0,125454732	0,146595543	0,855788173	0,392114958	0,570593402	236,8668716	221,6341547	262,2772436	232,2361387	291,7131108	251,8784856	290,1119362
IL1B	36,98148896	0,540174152	0,631949428	0,854774335	0,392676089	0,570593402	37,21171397	12,01630959	12,56417933	71,34965708	5,126768204	61,93733252	20,00771974
TNFRSF14	93,67240457	-0,229735338	0,268671295	-0,855079582	0,392507092	0,570593402	77,28586748	100,1359133	92,66082259	50,36446382	52,29303568	75,70118419	74,40370777
ECSCR	158,0438154	0,226763794	0,26759063	0,847428005	0,3967566	0,575206487	125,9473396	122,8333869	164,9048538	186,0687136	118,4283455	134,8857464	160,6869991
MCM6	132,4810581	0,215527947	0,25618377	0,841302115	0,400178706	0,577640404	108,0570925	114,8225139	147,6291072	170,6795718	120,4790528	112,8635837	107,5414936
S1PR1	86,07374244	0,225126748	0,267636428	0,841166316	0,400254768	0,577640404	49,377082	84,11416715	70,67350876	81,14274727	49,21697476	96,3469617	56,896953
FLT3	41,53848383	0,400721099	0,481035097	0,833039214	0,404822614	0,582907845	22,18390641	29,37320123	23,55783625	78,3447215	19,994396	24,77493301	18,75723725
EZH2	39,8724359	0,343994855	0,414525794	0,829851509	0,406622744	0,584175209	25,76195583	20,02718265	37,692538	33,57630921	19,994396	34,40962918	31,26206209
CASP1	77,53673891	0,301192776	0,369789358	0,814498227	0,415359588	0,595379996	64,40488957	62,75183899	54,96828459	159,4874688	55,3690966	55,05540668	60,02315921
HLA-DRB1	2224,856585	0,495121134	0,613849795	0,806583529	0,419906467	0,600541906	1909,247171	2300,455714	791,5432981	6155,656689	916,153478	2985,379427	4512,366042
NOD2	28,63380308	0,287817571	0,361225031	0,796781913	0,425577701	0,607285034	15,74341745	26,70291021	21,98731384	39,17236075	18,45636553	27,52770334	27,51061464
GNG11	573,3323373	-0,146733892	0,185616843	-0,79052035	0,429223942	0,608959269	636,1771869	684,9296468	670,613072	566,600218	442,9527728	483,1111937	360,7641965
HLA-DQA1	216,7582692	-0,779823505	0,984342765	-0,792227598	0,428227979	0,608959269	598,2498631	646,210427	347,0854541	263,0144222	6,664798665	344,0962918	440,7950754
NFKBIA	1641,460509	-0,180160768	0,228101135	-0,789828459	0,429627957	0,608959269	969,6513929	2831,843627	1936,45414	1345,850394	1506,244498	898,7795141	2013,90204
ACVR1	399,9400264	-0,125292854	0,16149261	-0,775842645	0,437841901	0,619219615	486,6147212	461,9603466	402,0537387	402,9157106	405,0146881	418,4210908	373,8942626
AOAH	36,9002533	0,328747664	0,431693417	0,761530407	0,446340324	0,62983579	28,62439536	26,70291021	21,98731384	22,38420614	24,60848738	15,14023684	24,38440843
UMOD	12748,23612	-0,425339252	0,563749974	-0,754482079	0,450559802	0,634380209	17804,37392	19322,22583	16678,94807	17378,53804	18156,44959	17934,29873	8255,060115
TLR8	44,8056682	0,357180074	0,478001874	0,747235718	0,454921287	0,639104021	23,61512617	30,70834674	23,55783625	61,55656689	17,94368871	34,40962918	24,38440843
APOLD1	133,3106093	-0,266774012	0,358168908	-0,744827388	0,456376076	0,639732468	47,94586223	301,7428853	114,6481364	209,8519326	74,85081578	114,2399689	253,2227029
IGHA1	491,1266912	0,543334143	0,732286428	0,741969429	0,458105858	0,640742775	100,9009937	226,9747368	69,10298634	32,17729633	240,4454288	79,83033969	97,53763372
NLRP3	27,54165435	0,285880909	0,391217484	0,730746761	0,464933848	0,648863722	20,75268664	28,03805572	17,27574659	44,76841228	16,40565825	12,3874665	20,63296098
POLR2A	824,9006713	-0,054505708	0,07620158	-0,715283175	0,474434083	0,660670269	772,8586748	893,2123464	855,9347172	805,8314211	757,7363405	686,8161984	817,8155442
CD5	37,23774629	-0,325869063	0,462811494	-0,704107542	0,481365804	0,665941798	29,34000525	36,04892878	31,41044834	25,18223191	27,6845483	27,52770334	25,00964967
HLA-DQB1	286,6764667	0,378392068	0,536754311	0,704963258	0,480833102	0,665941798	95,17611458	109,4819318	362,7906783	449,0831357	528,5698018	110,1108134	70,02701908

SFTPA2	7,681602046	46,7508316	11,01435519	57,63017797	20,95495648	26,26713318	27,59536595	9,886543099	25,97194324	76,55711736	31,17553355	15,97949354	22,59616198	84,90757504	22,43300586	54,93362719	40,01299921	205,8170288	23,73733536
ABCE1	389,5669609	348,8997247	439,7874681	421,2964734	310,3952928	544,1049017	407,1999122	382,5069433	519,4388648	421,0641455	441,8325617	577,2592042	322,2409187	457,9863139	421,5535685	403,6427389	348,1832914	443,5711827	378,8083101
INPP5D	106,4450569	122,937372	97,16234759	97,37512829	82,51014112	195,8777646	61,24825126	97,50176987	219,2032009	147,2252257	233,2789925	88,88593283	93,33197339	126,0748841	107,4914864	235,2592295	76,51608622	177,4284731	179,0190708
NKG7	31,27509404	38,09327019	17,70164227	156,9925538	48,45833685	91,55972138	14,80726954	22,84132371	99,21282317	97,16864896	152,6526126	53,9307907	47,15720761	46,31322275	41,12717741	50,15679005	21,76145571	39,03426408	69,23389479
STAT4	28,53166474	42,4220509	20,45523107	57,63017797	43,21959773	67,54405676	19,51867348	32,04603625	46,23005896	70,66810833	92,45158226	37,95129716	34,38546388	77,18870458	38,32305168	74,04097578	34,39713968	163,2341952	63,29956095
UBB	6750,482141	8301,735634	5870,651318	4242,773447	7875,13458	6035,436716	8069,288839	6569,778347	2614,855245	6893,085067	5629,871353	7054,946399	6929,162193	6128,783144	7818,837251	5102,856283	7254,988542	4130,534854	6898,663088
TNF	29,62903646	23,3754158	24,38892936	19,87247516	17,02590214	29,26909126	14,80726954	10,90928894	39,47735372	47,11207222	56,97597512	13,98205685	19,6488365	51,45913639	28,9759659	52,54520862	14,03964885	28,38855569	43,51844816
CD48	35,11589507	24,24117194	18,88175176	67,56641555	20,95495648	96,81314802	13,46115412	24,88681539	70,64368561	117,7801806	136,5273366	36,95257881	56,98162586	46,31322275	64,49489185	121,8093473	27,37731525	127,7485006	140,4459009
P2RX4	144,8530672	216,4390352	130,598783	196,7375041	178,11713	122,3297917	135,2845989	137,7297728	141,2873712	220,8378385	227,9039005	157,7974987	205,3303415	182,6799342	183,2028812	175,5487652	139,694506	138,394209	196,8220723
CR1	127,8438055	116,877079	61,36569322	127,183841	124,4200541	322,7104934	121,1503871	174,2077077	102,8488952	126,6136941	506,3336657	194,7500775	93,33197339	198,1176751	117,7732808	236,4534388	233,7601533	156,1370563	266,0559671
FAS	272,1481868	191,3321071	178,1965322	290,1381374	191,2139778	239,4061567	117,7850986	121,7067547	339,1935787	182,5592799	380,556513	189,7564858	264,276851	244,4308978	186,0070069	396,4774832	101,7874541	251,9484318	223,5265746
CETP	39,50538195	38,09327019	12,98120433	43,71944536	58,93581509	101,3160851	17,49950036	26,59139178	43,11342577	50,05657673	41,92571754	31,95898708	49,12209126	59,17800685	35,51892595	72,8467665	40,71498166	60,32568085	28,68261356
IL18	328,1141445	230,2911334	212,8130771	262,3166721	201,6914561	286,6869965	252,3966398	283,6415124	447,7563014	167,8367573	288,1049308	366,5296331	197,4708069	607,2178094	242,0895216	330,7959724	274,475135	340,6626683	267,0450228
CTNNB1	2129,99851	2230,187819	2057,324202	1965,387793	1799,506887	2241,712195	2057,537408	1808,896472	1945,817987	2049,375142	2257,538637	2264,094491	1630,85343	1801,069774	2025,513487	1772,206582	1961,338944	1880,741815	2115,590014
BASP1	157,4728419	181,8087896	231,301459	97,37512829	96,9166737	327,96392	106,3431176	63,75115722	81,0324629	359,2295507	256,9293972	105,8641447	96,27929887	100,345316	229,0036015	247,2013224	73,70815645	106,4570839	288,8042469
HLA-DRA	2213,947447	1979,118538	1318,575664	1691,147636	1892,494507	6262,084551	2982,318696	2257,200064	2521,875688	4337,255149	7005,894902	3059,074295	1339,068208	1839,664126	4715,604774	4066,282622	2646,473808	972,3080325	5880,924835
HLA-DRB3	1313,005264	2193,826061	1001,519583	1862,050923	1791,648779	4937,470549	1478,034723	1493,208923	2662,124182	5420,83281	4998,835553	1941,508465	1225,104956	1795,92386	2853,665287	4163,013574	1938,875506	1220,707895	4446,794157
SOX7	50,47909916	49,34810002	39,33698283	53,65568294	40,60022817	33,02153886	34,32594301	34,09152793	55,06051966	47,11207222	46,22579113	37,95129716	42,24499848	54,03209321	35,51892595	66,87572006	61,07247248	49,67997247	36,59505868
SELPLG	35,66458093	37,22751405	20,06186124	61,604673	39,29054339	84,80531571	36,34511613	30,00054458	39,47735372	97,16864896	92,45158226	55,9282274	31,43813841	72,04279094	50,47426319	85,98306865	47,03282364	180,9770426	79,12445119
IRF6	220,02303	199,1239124	198,2583935	222,5717218	273,724119	209,386576	198,5520233	222,6176774	196,3478909	229,6713521	170,9279253	248,6808682	177,8219704	187,8258478	236,4812701	170,771928	241,4819602	227,1084456	203,7454618
ZEB1	182,1637057	173,1512281	125,0916054	262,3166721	107,3941519	165,1076943	133,9384835	146,9344854	130,3791551	232,6158566	184,9031645	114,8526098	142,4540647	113,2101001	106,5567778	280,6391824	168,4757862	230,657015	149,3474016
CD55	237,5809776	269,2501598	180,5567512	377,5770281	185,9752387	255,9169262	162,2069072	175,2304535	281,5358647	185,5037844	289,1799492	261,6642067	191,5761559	239,2849842	156,0963324	256,7549967	154,4361373	361,9540851	209,6797957
CD83	181,6150198	418,160216	170,7225055	306,0361175	336,5889884	179,3669952	290,0878714	309,2101583	189,0757468	895,1293722	274,1296916	204,737261	265,2592928	252,1497683	181,333464	175,5487652	174,0916457	372,5997935	566,7288817
GBP1	187,1018784	192,1978632	171,902615	260,3294246	184,6655539	308,4511925	167,5913688	190,2307258	162,0649258	335,6735146	267,6795812	162,7910905	164,0677848	254,7227251	380,4263911	386,9238089	164,2638915	237,7541539	214,6250739
PTGER4	190,3939936	232,8884019	237,9887461	121,2220985	132,2781627	278,4316118	201,9173119	186,821573	140,7679323	256,1718927	213,9286613	169,7821189	117,893019	190,3988046	264,5225274	359,4569953	239,3760128	287,4341264	235,3952423
CX3CL1	187,6505643	296,9543563	212,4197073	143,0818212	206,9301952	213,8895131	142,6882337	182,7305897	161,5454869	235,5603611	226,8288821	111,8564548	102,1739498	156,950366	186,0070069	183,9082302	179,0055228	149,0399174	187,9205716
CD22	25,23954958	39,82478247	14,16131382	45,70669287	28,81306515	33,77202838	19,51867348	20,79583204	73,24087993	29,44504514	34,40058875	6,991028424	19,6488365	51,45913639	26,17184017	102,7019987	17,54956106	53,22854193	49,452782
SPRY4	249,6520665	161,0306422	301,3212885	310,0106125	309,085608	333,2173467	117,7850986	276,1413762	201,5422795	253,2273882	333,2557035	175,774429	183,7166213	216,1283728	180,3987555	333,184391	261,839451	216,4627372	235,3952423
ST8SIA4	59,80675879	58,00566143	39,33698283	75,51540561	36,67117383	81,05286811	32,9798276	35,45518904	92,97955679	73,61261284	158,0277046	48,93719897	51,08697491	82,33461822	89,73202344	134,9456494	37,20506944	99,35994493	93,96028579
CARD8	165,7031298	130,7291773	113,2905106	133,1455836	134,8975323	189,1233589	139,9960029	187,8443189	316,3382686	147,2252257	220,3787717	119,8462016	140,489181	216,1283728	153,2922067	130,1688123	72,30419156	159,6856258	201,7673505
PRF1	17,55794753	25,10692808	8,260766395	41,73219784	19,6452717	24,76615414	16,15338495	11,25020422	30,12745416	58,89009028	58,05099351	16,97821189	35,36790571	90,05348868	24,30242302	46,57416219	18,2515435	117,1027922	40,55128124
TREM1	14,81451823	22,50965966	7,474026738	77,50265313	31,43243471	26,26713318	16,82644265	8,863797261	43,63286464	82,44612639	26,87545996	14,9807752	24,56104563	77,18870458	15,89004582	53,73941791	32,29119235	205,8170288	22,74827972
PTPN2	176,6768471	150,6415685	148,3004253	192,7630091	132,2781627	200,3807017	121,1503871	133,6387895	242,058511	167,8367573	207,4785509	163,7898088	162,1029012	216,1283728	168,247544	198,2387416	119,3370152	216,4627372	154,2926798
AGR2	81,7541932	21,64390352	35,79665438	67,56641555	26,19369559	45,77986069	37,01817384	39,20525712	297,1190306	53,00108125	180,6030909	99,87183463	119,8579027	203,2635887	72,90726905	109,8672544	23,1654206	159,6856258	98,90556399
PAX5	8,778973767	24,24117194	9,047506051	33,78320777	17,02590214	21,01370655	10,7689233	6,818305585	36,36072053	79,50162187	19,35033117	7,989746771	12,77174373	20,58365456	16,8247544	69,26413864	23,86740304	191,6227509	31,64978048
ITGA4	53,22252846	50,21385616	32,64969575	47,69394039	52,38739119	102,0665747	34,99900072	41,93257935	61,29378604	108,946667	87,07649027	50,93463566	44,20988213	66,8968773	88,79731486	125,3919751	42,11894654	102,9085144	89,01500759
CCR5	29,62903646	33,76448949	23,6021897	63,59192052	34,05180427	75,04895195	18,17255807	21,13674731	24,93306551	129,5581986	74,17626949	30,96026874	21,61372015	77,18870458	56,08251465	96,73095223	35,80110456	269,6912791	55,38711583
COL13A1	27,43429302	19,0466351	23,6021897	21,85972268	22,26464125	19,51272751	25,57619283	35,45518904	22,33587118	35,33405417	37,62564394	16,97821189	28,49081293	61,75096367	28,9759659	56,12783648	24,56938548	31,93712516	18,79205716
BLNK	182,1637057	212,1102545	170,3291357	188,788514	150,6137497	237,9051777	123,1695602	144,5480784	299,716225	209,0598205	190,2782565	199,7436693	208,2776669	313,900732	209,3747214	185,1024395	141,8004534	376,148363	160,2270137
FAM30A	16,46057581	24,24117194	14,94805348	25,83421771	23,57432603	30,01958078	14,80726954	10,90928894	38,43847599	44,16756771	18,27531277	14,9807752	16,70151103	64,32392048	30,84538306	37,02048789	24,56938548	81,61709762	33,62789176
IL7R	23,04480614	21,64390352	13,37457416	79,48990065	36,67117383	56,28671397	14,13421183	43,29624047	189,5951856	79,50162187	102,1267478	73,90515763	55,01674221	77,18870458	46,73542888	519,4810398	42,11894654	92,26280601	182,9752934
KLF2	284,7679616	201,7211808	267,8848531	319,9468501	239,6723147	336,2193048	253,0696975	371,2567391	303,8717359	282,6724333	1000,842129	282,637292	256,4173164	295,8900342	292,5637848	704,5834792	487,8777974	461,31403	338,2570288
TYK2	308,3614536	310,8064545	273,7854005	385,5260181	289,4403363	269,4257375	265,8577939	266,9366637	402,5651202	362,1740552	290,2549676	247,6821499	347,7844061	365,3598684	252,3713159	291,387066	254,1176441	315,8226821	312,5415822
NOS3	221,1204017	169,6882036	171,5092451	180,839524	130,968478	206,3846179	118,4581563	191,9353022	259,1999935	318,0064875	279,5047836	171,7795556	123,78767	190,3988046	197,2235099	336,7670189	149,5222602	138,394209	183,964349
RGS5	1503,399258	1268,332746	1382,694947	604,1232449	928,5665088	1245,812602	1581,685609	1530,027773	956,28695	1068,855139	1062,118178	1319,306936	840,9702024	723,0008662	1486,186638	2727,574011	1316,919062	589,0625307	1015,760142
CCL20	29,62903646	39,82478247	22,02871039	19,87247516	36,67117383	18,01174847	10,7689233	22,15949315	53,50220307	50,05657673	84,92645347	14,9807752	13,75418555	74,61574776	25,23713159	57,32204577	16,14559617	134,8456396	46,48561508
ITGAX	37,31063851	29,43570878	28,71599747	61,604673	39,29054339	72,7974834	21,5378466	18,40942508	54,02164193	61,83459479	67,7261591	19,97436693	39,29767301	92,6264455	48,60484603	68,06992935	27,37731525	99,35994493	39,5622256
MARCH8.	120,1622034	118,6085913	97,94908725	184,814019	117,8716302	124,5812602	100,2855982	93,7517018	93,49899566	135,4472076	113,9519502	111,8564548	148,3487156	159,5233228	96,27498349	109,8672544	106,7013312	131,2970701	113,7413986
TLR3	123,4543186	133,3264457	129,8120433	91,41338574	94,29730414	155,3513305	117,7850986	99,88817683	94,53787339	138,3917121	149,4275574	110,8577364	106,1037171	144,0855819	141,1409952	102,7019987	70,19824424	110,0056533	104,8398978
REL	134,4280358	116,877079	106,6032235	103,3368708	81,20045634	124,5812602	81,43998245	97,16085459	142,8456878	120,7246851	162,3277782	115,8513282	73,68313689	141,5126251	100,0138178	187,490858	105,9993488	173,8799036	178,0300152
NOD1	77,36470632	65,7974667	78,28059583	101,3496233	68,10360854	110,3219594	61,24825126	57,6146822	169,8565088	100,1131535	140,8274102	74,90387598	79,57778784	105,4912296	92,53614918	125,3919751	80,02599843	124,1999312	66,26672787
PDPN	36,76195265	40,69053861	11,80109485	61,604673	20,95495648	15,76027991	22,88396201	41,59166407	30,12745416	35,33405417	29,02549676	30,96026874	19,6488365	38,59435229	22,43300586	69,26413864	66,68833202	92,26280601	35,60600304
IMPDH2	512,4725936	281,3707457	339,084792	524,6333443	302,5371841	529,0951113	359,4128151	386,938842	394,7735372	482,8987403	534,284144	462,4065944	273,1188274	519,7372775	495,3955461	382,1469718	323,6139059	507,445433	406,501868
CD3E	13,71714651	15,58361053	11,40772502	35,77045529	22,26464125	45,77986069	16,15338495	13,63661117	23,89418778	47,11207222	60,20103031	22,97052197	20,63127833	48,88617957	45,8007203	84,78885936	18,95352594	67,42281978	49,452782
GAPDH	8431,655617	5695,80965	7667,171324	13074,10141	7535,926222	5852,317273	8993,39707	6825,123891	8435,687164	8297,61372	7104,796595	9522,779432	15666,01734	9386,146477	7432,802608	8967,317535	8380,968379	11791,89632	7821,452
HLA-F	132,2332924	83,97834565	82,60766395	155,0053063	96,9166737	216,8914711	115,0928678	75,34227672	171,9342642	173,7257663	233,2789925	117,8487649	111,0159262	151,8044523	161,7045839	194,6561138	120,0389976	141,9427785	137,4787339
PIN1	307,2640818	305,6119177	272,9986608	329,8830877	269,7950646	253,6654576	271,2422556	263,1865956	204,6589127	356,2850462	277,3547468	308,603969	317,3287095	308,7548183	275,7390304	312,8828331	290,6207311	220,0113066	233,417131
CCR10	21,94743442	27,7041965	16,91490262	41,73219784	24,88401081	24,01566463	24,90313513	18,40942508	16,62204367	94,22414444	29,02549676	11,98462016	22,59616198	51,45913639	21,49829728	53,73941791	22,46343816	81,61709762	24,726391
NRDE2	157,4728419	179,2115211	171,902615	192,7630091	191,2139778	181,6184637	157,4955032	162,9575035	239,4613167	150,1697302	168,7778886	165,7872455	176,8395285	151,8044523	142,0757038	169,5777187	170,5817335	159,6856258	154,2926798
TCF7	41,15143953	20,77814738	16,52153279	47,69394039	19,6452717	30,01958078	14,13421183	12,61386533	31,68577075	44,16756771	37,62564394	16,97821189	49,12209126	51,45913639	28,9759659	74,04097578	18,2515435	39,03426408	55,38711583
IGKC	732,4956237	331,5846019	533,0161174	457,0669287	244,9110538	7628,725966	254,4158129	697,5126614	8946,815007	5883,120019	2575,744083	1647,885271	915,6357811	2145,845987	1074,914864	6823,711865	188,1312946	7001,327548	9631,423821
CXCL12	356,0971234	933,2851197	643,1596693	135,1328311	666,6295529	1590,287292	424,0263549	642,9662167	122,0681332	1313,249013	799,8136884	441,4335091	313,3989422	265,0145524	603,8217411	1937,007463	433,1231669	149,0399174	1064,223869
LCK	8,778973767	21,64390352	13,37457416	25,83421771	19,6452717	25,51664366	12,78809642	8,181966703	18,69979913	58,89009028	59,12601191	12,9833385	13,75418555	41,16730911	26,17184017	66,87572006	17,54956106	49,67997247	31,64978048
CD68	373,1063851	208,6472299	257,2638677	474,9521564	513,3964336	381,2486759	233,551024	214,4357107	336,0769455	323,8954965	555,784512	260,6654884	515,7819582	401,3812638	488,852586	555,3073184	350,9912212	457,7654606	642,8861659
SMAD2	1157,17848	1039,773125	1220,233207	1218,182727	971,7861065	1302,849806	908,6279033	1234,795142	1041,994363	1036,465589	1142,744558	1134,544041	1011,91508	1013,744987	870,2136857	1064,040475	920,2989819	947,4680463	1015,760142
XAF1	187,1018784	141,9840071	119,191058	101,3496233	121,8006845	234,1527301	74,03634768	178,2986911	167,7787533	191,3927934	260,1544524	84,89105944	110,0334844	64,32392048	98,14440064	180,3256023	108,8072786	159,6856258	126,5991219
HLA-C	4449,293642	4829,187753	1920,824872	7237,555454	3158,959689	2728,779893	1865,042904	1559,346487	2158,787922	6430,797858	3870,066234	2230,138067	2681,083741	4363,734766	6124,2106	5732,204577	2541,176441	2718,204208	3018,597813
CD45RB	35,11589507	19,91239124	15,73479313	29,80871274	34,05180427	47,28083973	15,48032724	17,04576396	44,67174237	55,94558576	72,02623269	22,97052197	30,45569658	41,16730911	35,51892595	52,54520862	16,84757862	63,87425031	80,11350683
NPHS2	312,2022546	415,5629476	134,1391115	453,0924337	374,569847	114,074407	443,5450284	461,5992881	312,7021966	50,05657673	342,9308691	471,3950595	326,170686	457,9863139	296,3026191	224,5113459	628,2742859	351,3083767	435,1844816
RAMP3	85,59499423	83,11258951	64,11928201	79,48990065	113,9425758	57,03720349	69,32494374	53,86461413	59,21603058	153,1142347	125,7771526	55,9282274	88,41976427	113,2101001	84,12377198	157,6356259	115,1251205	237,7541539	112,7523429
RELA	216,182229	219,0363036	197,078284	298,0871274	183,3558692	201,8816808	181,052523	177,2759452	141,8068101	353,3405417	232,2039741	174,7757106	238,7333635	213,555416	195,3540927	267,5028802	190,2372419	333,5655294	225,5046859
LRP2	3440,260345	3407,61617	2109,642389	2605,281494	4188,371925	2631,966745	4472,468457	2318,90573	1695,448455	3374,402173	4467,776464	3448,57445	6180,541522	3257,363333	3865,019968	1221,6761	3562,560895	869,3995182	3470,59624
TP53	211,2440563	171,4197159	201,4053521	149,0435637	141,4459562	339,9717524	168,2644265	164,6620799	268,5498931	250,2828837	325,7305747	235,6975297	180,7692958	210,9824592	265,457236	278,2507638	141,8004534	216,4627372	241,3295761
BTK	24,14217786	37,22751405	12,19446468	39,74495032	27,50338037	43,52839213	14,13421183	18,0685098	35,3218428	106,0021625	41,92571754	27,9641137	37,33278936	64,32392048	27,10654875	72,8467665	23,86740304	205,8170288	47,47467072
PROX1	59,80675879	113,4140544	258,0506074	55,64293045	65,48423898	236,4041987	45,76792402	113,524788	24,41362664	91,27963993	47,30080953	41,94617055	37,33278936	92,6264455	101,883235	81,2062315	44,92687631	152,5884869	39,5622256
SLC4A1	47,186984	81,38107723	44,4507906	27,82146523	130,968478	30,7700703	200,5711964	105,6837366	34,28296507	108,946667	34,40058875	87,88721448	68,77092776	136,3667114	117,7732808	38,21469718	186,7273297	60,32568085	150,3364573
SP140	13,71714651	15,58361053	13,76794399	23,84697019	13,0968478	28,51860174	6,730577062	8,181966703	47,78837556	47,11207222	41,92571754	15,97949354	25,54348745	51,45913639	28,04125733	75,23518507	19,65550839	46,131403	49,452782
AIM2	18,10663339	25,10692808	10,22761554	27,82146523	23,57432603	18,76223799	12,78809642	6,477390306	55,06051966	35,33405417	27,95047836	19,97436693	14,73662738	59,17800685	25,23713159	33,43786003	21,05947327	88,71423655	36,59505868
BATF3	24,69086372	26,83844036	19,27512159	59,61742548	26,19369559	35,27300742	20,86478889	16,02301813	62,33266377	47,11207222	34,40058875	15,97949354	25,54348745	56,60505003	35,51892595	60,90467363	19,65550839	102,9085144	26,70450228
FABP1	968,9792295	1822,416676	707,6723211	590,2125123	2978,223189	60,78965108	6540,774789	2496,181675	1026,930636	288,5614424	222,5288085	829,9349458	4226,464732	676,6876435	1379,62986	137,334068	3147,689272	801,9766984	5196,498332
ICOSLG	154,1807268	134,1922018	209,2727487	206,6737417	134,8975323	105,8190223	154,8032724	151,7072993	176,609214	176,6702708	138,6773734	130,8321034	174,8746449	120,9289705	129,9244923	203,0155788	148,1182953	220,0113066	206,7126287
IFI27	780,7799794	550,6209055	514,9211053	562,3910471	569,7128792	469,8064392	362,7781036	685,5806266	579,6937731	680,1805427	1002,992166	448,4245375	473,5369597	403,9542206	446,7907001	819,2275708	766,5648271	376,148363	509,3636546
MAPK3	468,0290389	492,6152441	502,3332708	461,0414237	429,5766077	568,8710558	384,9890079	468,758509	395,8124149	479,9542357	565,4596776	534,3143153	355,6439407	355,0680411	400,0552712	494,4026447	396,6200799	553,5768361	549,9149358
NOTCH1	283,121904	331,5846019	260,0174565	409,3729883	301,2274993	282,1840593	307,5873717	263,1865956	362,5683276	412,2306319	348,3059611	308,603969	354,6614989	298,462991	234,611853	347,5149025	328,527783	461,31403	389,6879221
RORA	420,2933691	577,4593459	451,5885629	465,0159188	526,4932814	222,1448978	374,8931423	459,5537965	330,363118	276,7834243	265,5295444	355,5437313	410,6606829	334,4843865	186,0070069	352,2917396	369,2427647	351,3083767	357,049086
RPS6	15489,40184	10126,74958	10573,78098	15200,45625	13880,0393	13281,41303	9487,421426	10921,9028	8987,331238	14996,36149	14511,67336	12591,84091	11887,54608	13227,57101	11269,78132	12821,0309	9663,490301	9417,903352	10403,87628
CD4	109,7371721	73,58927196	52,71155699	67,56641555	51,07770641	204,1331493	84,13221327	95,4562782	83,11021836	159,0032437	259,079434	148,8090336	77,61290419	77,18870458	122,4468237	170,771928	77,21806866	63,87425031	258,143522
IGF1R	514,6673371	780,0462828	497,6128328	761,1157987	632,5777486	376,7457388	444,8911438	471,8267465	357,8933778	441,6756771	510,6337392	490,3707081	778,0939255	542,8938889	428,0965285	419,1674597	452,0766929	390,3426408	413,4252575
BMPR1B	98,76345488	251,0692808	153,8076029	141,0945736	115,2522606	114,8248965	105,6700599	132,9569589	137,1318603	123,6691896	93,52660066	136,8244134	127,7174373	226,4202001	85,05848055	148,0819516	124,2508923	110,0056533	83,08067375
CD274	25,78823544	12,98634211	19,66849142	29,80871274	24,88401081	18,76223799	14,13421183	15,68210285	22,85531005	41,22306319	18,27531277	13,98205685	26,52592928	41,16730911	18,69417155	39,40890646	19,65550839	63,87425031	13,84677896
PSTPIP1	27,98297888	34,63024563	23,99555953	51,66843542	34,05180427	46,53035021	24,90313513	20,79583204	62,33266377	88,33513541	88,15150867	43,94360724	30,45569658	72,04279094	30,84538306	114,6440915	39,31101677	230,657015	57,36522711
ATM	179,9689622	264,0556229	237,5953763	218,5972268	203,0011409	299,4453183	197,8789656	242,3907636	282,5747424	259,1163972	333,2557035	264,6603618	228,9089453	231,5661137	265,457236	259,1434152	194,4491365	312,2741126	271,990301
IL1B	23,04480614	16,44936667	10,62098536	39,74495032	30,12274993	24,01566463	14,13421183	7,841051423	24,93306551	50,05657673	41,92571754	9,987183463	19,6488365	177,5340205	44,86601172	50,15679005	14,03964885	70,97138924	71,21200607
TNFRSF14	72,42653358	61,46868599	72,38004841	109,2986134	100,845728	57,787693	72,69023227	85,5697351	189,5951856	167,8367573	151,5775942	95,87696125	95,29685704	97,77235914	71,03785189	127,7803937	77,9200511	131,2970701	74,17917299
ECSCR	189,2966218	130,7291773	133,3523718	188,788514	128,3491084	174,8640581	90,86279033	83,18332814	272,1859651	256,1718927	239,7291028	118,8474832	113,9632517	172,3881069	160,7698753	247,2013224	132,6746816	138,394209	123,631955
MCM6	158,0215278	102,1592246	77,88722601	141,0945736	78,58108678	150,8483934	88,84361721	85,22881982	128,3013996	179,6147753	144,0524654	118,8474832	88,41976427	231,5661137	125,2509494	192,2676952	125,6548572	227,1084456	118,6866768
S1PR1	72,97521944	73,58927196	66,47950098	103,3368708	69,41329332	55,53622445	55,19073191	65,11481834	85,70741268	135,4472076	103,2017662	69,91028424	87,43732244	108,0641864	79,45022909	149,2761609	109,509261	177,4284731	83,08067375
FLT3	37,85932437	18,18087896	10,62098536	53,65568294	35,36148905	46,53035021	62,59436667	42,27349463	59,73546945	55,94558576	18,27531277	16,97821189	27,5083711	56,60505003	15,89004582	68,06992935	91,95969995	124,1999312	20,77016844
EZH2	49,38172744	16,44936667	25,17566901	39,74495032	26,19369559	49,53230829	18,84561577	14,31844173	39,47735372	61,83459479	47,30080953	21,97180362	32,42058023	82,33461822	35,51892595	64,48730149	32,99317479	138,394209	37,58411432
CASP1	63,09887395	58,87141757	53,10492682	69,55366307	60,24549987	102,8170642	47,11403943	31,36420569	83,11021836	114,835676	141,9024286	50,93463566	44,20988213	74,61574776	66,364309	83,59465008	47,73480608	216,4627372	93,96028579
HLA-DRB1	382,4340447	2317,629189	503,5133802	1985,260269	1770,693822	4947,226913	1523,129589	1821,169422	784,8721246	4970,323619	4584,953469	1320,305654	258,3822	1407,40738	2736,826715	1950,143765	523,678902	532,2854193	3955,233504
NOD2	23,593492	22,50965966	18,0950121	27,82146523	23,57432603	40,52643406	18,17255807	14,65935701	25,45250437	35,33405417	41,92571754	9,987183463	21,61372015	36,02139547	34,58421737	51,35099933	18,95352594	74,5199587	28,68261356
GNG11	699,0257862	645,854081	544,4238424	592,1997598	485,8930533	583,1303567	465,7559327	411,8256574	896,5514806	541,7888305	746,0627685	526,3245685	455,8530069	607,2178094	496,3302547	617,4062013	471,0302188	663,5824894	611,2363855
HLA-DQA1	7,681602046	360,1545545	105,423114	143,0818212	19,6452717	14,25930087	169,610542	185,7988272	7,272144107	50,05657673	689,0867934	441,4335091	15,7190692	43,74026593	18,69417155	44,18574361	19,65550839	134,8456396	519,254211
NFKBIA	1663,615529	1595,588567	1808,714471	1552,04031	1491,730964	1370,393863	1492,168935	2053,673642	1729,211981	1763,758204	1754,430026	1548,013437	1045,318102	1677,567846	1064,63307	1987,164253	1779,525491	1422,976354	2374,722591
ACVR1	372,5576992	290,0283071	372,1278576	610,0849875	398,144173	434,5334318	484,6015484	323,8695153	336,5963844	373,9520733	359,0561451	541,3053437	400,8362647	280,4522933	378,5569739	452,6053197	334,845625	351,3083767	352,1038078
AOAH	42,79749711	19,91239124	13,76794399	43,71944536	17,02590214	64,54209868	18,17255807	22,84132371	37,91903713	35,33405417	99,97671105	48,93719897	36,35034753	54,03209321	43,93130314	69,26413864	9,125771751	49,67997247	68,24483915
UMOD	7037,993532	13861,62157	13978,00348	2748,363315	13136,13834	27192,48676	9714,241873	22175,51617	2253,325795	27678,34243	2472,542316	12130,43303	4291,305892	2114,970506	21935,7409	14445,15553	9849,515649	6731,636269	2176,911463
TLR8	18,65531925	32,89873335	13,37457416	55,64293045	37,98085861	43,52839213	19,51867348	15,00027229	27,53025983	82,44612639	84,92645347	28,96283204	30,45569658	102,9182728	41,12717741	63,2930922	33,69515723	170,3313342	46,48561508
APOLD1	132,7819782	63,20019827	75,92037686	135,1328311	163,7105975	84,05482619	101,6317136	109,4338046	196,3478909	126,6136941	123,6271158	128,8346667	82,52511331	149,2314955	103,7526521	219,7345088	99,68150681	127,7485006	125,6100663
IGHA1	226,6072604	158,4333738	131,7788925	79,48990065	49,76802163	1472,460437	14,80726954	136,707027	1558,316594	895,1293722	743,9127317	363,5334781	232,8387126	738,4386072	182,2681726	1101,060962	65,98634958	1192,319339	2578,468053
NLRP3	24,69086372	23,3754158	12,58783451	25,83421771	14,40653258	38,2749655	12,11503871	16,36393341	35,84128167	41,22306319	48,37582793	15,97949354	34,38546388	41,16730911	19,62888013	56,12783648	16,84757862	39,03426408	39,5622256
POLR2A	804,9221572	852,7697986	769,0380143	800,860749	822,4820416	813,5306392	815,0728822	835,2424342	832,6605002	942,2414444	784,7634308	823,9426357	765,3221818	810,4813981	907,6020288	1010,301057	822,7234224	805,5252678	837,730127
CD5	19,75269098	23,3754158	17,70164227	23,84697019	44,52928251	32,27104934	13,46115412	21,81857787	18,69979913	70,66810833	59,12601191	11,98462016	18,66639468	51,45913639	28,9759659	85,98306865	27,37731525	159,6856258	36,59505868
HLA-DQB1	504,2423057	74,4550281	108,9634424	83,46439568	248,8401081	779,0081213	412,5843739	91,02437957	231,1502948	621,2904524	507,4086841	164,7885271	261,3295255	190,3988046	468,2889973	401,2543204	170,5817335	173,8799036	235,3952423

SRC	365,6790909	0,103859461	0,147104961	0,706022832	0,480173939	0,665941798	359,2361618	347,1378327	332,9507524	356,7482854	457,3077238	476,2292678	334,5040643	319,3351708	385,2614826	291,093673	486,8756415	360,1633144	389,5040606	341,9133147	335,1197195	411,9150197	500,5657673	387,0066234	289,6283204	374,3103354	362,7869115	332,7562536	369,0106696	310,978222	319,3712516	275,9465235
CHCHD10	4703,080183	0,146239064	0,211727335	0,69069525	0,489757075	0,676077701	3606,673816	5369,955243	4300,090377	4057,137363	5939,360964	7115,911314	6168,630091	4773,566986	4302,808019	5480,428448	3863,209171	4634,974435	3701,41431	6048,769605	5106,229053	2464,737413	4552,203978	4178,596515	5261,248249	6422,222211	4896,336827	4841,790432	3456,041676	5134,299583	2707,558499	3895,890166
ATXN3	285,8460778	0,093934837	0,136355936	0,688894373	0,490889741	0,676171335	259,050778	339,1269596	273,2709005	286,7976412	328,6258419	268,3951076	323,8749632	283,121904	306,4776738	279,2925781	339,8193253	244,9110538	297,9443393	244,993005	269,3230706	349,582356	341,5625236	275,20471	169,7821189	308,4867331	244,4308978	300,0414534	285,4160195	270,2632403	290,9826959	251,2201325
LAYN	59,17501004	0,205758905	0,301723455	0,681945344	0,495273512	0,680733074	59,39562038	49,40038388	48,68619492	96,53188899	34,86202379	52,30263635	50,64454058	46,08961228	83,11258951	50,74470785	51,66843542	45,83896729	69,79552532	37,01817384	38,52342656	50,38556988	82,44612639	81,70139828	29,96155039	39,29767301	105,4912296	54,2130975	83,59465008	37,90705189	88,71423655	70,22295043
CCL15	262,8945526	0,217454742	0,321257859	0,676885362	0,498478692	0,682334651	201,0863774	256,347938	251,2835867	281,2015897	265,566593	392,2697726	276,3566289	192,588737	165,3594229	184,0970796	250,393187	189,9042931	204,8836388	397,7771043	262,504765	76,35751312	273,8389198	297,7800964	281,6385737	636,6223027	295,8900342	280,4125733	165,9950909	225,336364	166,7827647	362,9834198
IL2RB	37,48249901	0,317008441	0,468453056	0,676713359	0,498587839	0,682334651	17,17463722	29,37320123	14,13470175	32,17729633	22,04510328	28,90408851	29,38633836	12,61977479	18,18087896	15,73479313	35,77045529	34,05180427	53,28475589	14,13421183	11,5911195	28,0496987	44,16756771	77,40132469	26,96539535	20,63127833	54,03209321	35,51892595	87,17727794	27,37731525	138,394209	66,26672787
IGHG2	1628,210662	-0,495940127	0,7395917	-0,670559347	0,502501288	0,685600431	532,4137538	902,558365	315,6750058	67,15261843	130,2199124	89,46503586	448,9232116	493,8172744	291,7598194	471,6504241	236,4824544	251,4594777	4707,070267	400,4693352	508,986512	6298,196235	3701,242174	1723,254493	885,8631732	752,5504381	1088,360735	455,2030773	5211,529328	114,4231381	5205,751401	7048,999546
NFATC1	64,83116367	-0,188206112	0,281086097	-0,669567489	0,503133545	0,685600431	63,68927968	86,78445817	65,96194151	78,3447215	46,14091384	56,43179185	43,76688692	49,9304133	68,39473512	55,46514579	55,64293045	55,00676075	63,79160916	39,71040466	38,52342656	68,04649128	79,50162187	69,8761959	53,9307907	37,33278936	79,7616614	53,27838892	95,53674295	54,05264806	170,3313342	56,37617147
SLC19A3	131,954659	0,332913499	0,498859922	0,667348657	0,504549453	0,686057608	86,58879597	92,12504021	182,1806004	500,8466124	75,3634926	114,2399689	64,3998479	43,89486883	112,5482983	59,00547425	73,5281581	70,7229781	141,0920297	91,53584804	33,06878209	149,598393	150,1697302	134,3772998	102,8679897	61,89383499	195,5447183	283,216699	76,42939436	119,3370152	276,788418	139,4568452
ANXA1	661,5364611	0,212832852	0,321693305	0,661601747	0,508226488	0,68811049	726,3440323	491,3335478	603,0806081	506,442664	497,2965158	444,572409	699,6449495	766,514147	526,3797336	586,1210442	981,700273	442,6734555	831,5423877	380,9506617	459,5537965	1345,34666	494,6767583	1255,621489	555,2874006	350,7317316	813,0543549	451,4642429	975,6689873	363,6269051	589,0625307	1061,256702
LAMP1	2915,83089	-0,114058103	0,172187101	-0,662407941	0,507709816	0,68811049	2990,533706	2889,254884	3263,545582	1853,692071	3157,576537	3047,31676	3058,054913	2876,759966	3019,757419	2914,083688	2692,720384	3191,701808	2380,552756	3908,4461	3190,285183	1706,356671	2856,169378	2985,326092	3413,619308	4057,484738	3084,975226	2658,311194	2319,154435	2824,075366	2217,855914	3253,993055
SMAD4	576,0816713	0,073687397	0,111806639	0,659061013	0,50985659	0,688848797	515,9547264	626,1832443	573,2406821	533,0239088	654,1756228	437,6904831	577,7229074	599,1649596	677,0213021	609,7232339	771,0520363	546,1385531	634,163644	475,8517983	576,146822	528,7887643	553,5668486	637,4859103	556,2861189	574,7284677	573,7693707	487,9178775	574,414667	516,6590776	592,6111001	574,6413268
HLA-G	48,4943161	0,230149193	0,350617521	0,656410989	0,511559722	0,689682428	44,36781281	44,05980184	26,69888109	109,1230049	41,01414563	38,53878468	80,03087895	35,66458093	27,7041965	48,77785871	39,74495032	53,69707597	60,03916156	44,42180861	34,77335849	57,13827512	53,00108125	53,75091992	29,96155039	18,66639468	54,03209321	55,14780607	50,15679005	33,69515723	102,9085144	23,73733536
GNLY	39,73916317	0,391652882	0,598230719	0,654685341	0,512670365	0,689715428	22,89951629	25,3677647	18,846269	58,75854112	11,27889005	30,28047368	21,25820222	10,42503135	36,36175791	7,474026738	51,66843542	32,74211949	41,27692357	15,48032724	13,97752645	51,94388648	91,27963993	56,97597512	17,97693023	24,56104563	56,60505003	14,95533724	50,15679005	18,2515435	230,657015	21,75922408
TOX2	31,23657972	0,309887085	0,478286068	0,647911585	0,517042137	0,694126336	14,31219768	20,02718265	29,83992592	39,17236075	19,48171918	28,90408851	23,75916719	11,52240307	25,97268422	17,30827245	35,77045529	24,88401081	14,25930087	16,82644265	18,40942508	24,93306551	61,83459479	33,32557035	14,9807752	21,61372015	46,31322275	22,43300586	42,99153433	44,92687631	145,4913479	12,85772332
VSIR	119,8320584	-0,154176747	0,242252662	-0,636429527	0,5244965	0,702648265	105,9102628	138,8551331	125,6417933	71,34965708	99,45930316	85,33588036	133,1763845	138,2688368	119,4743474	89,29495103	155,0053063	137,5169019	116,3258755	81,43998245	109,7747199	62,85210264	111,8911715	169,8529069	107,8615814	144,4189483	133,7937546	80,38493767	185,1024395	105,2973664	131,2970701	176,0519039
RPL19	12975,81373	-0,082420908	0,130334862	-0,632378072	0,527139859	0,704702759	13797,67417	12609,1142	14442,52415	12284,73213	12580,06382	11580,9048	12738,66506	16147,82487	10680,83351	12345,91206	16941,28508	14118,40193	15490,85417	10828,82543	12261,35893	11154,94962	14566,46383	15248,06096	12779,59996	13445,69882	13814,20516	11767,98099	12895,07188	10676,45097	9375,320518	12798,37998
CD28	27,21298688	0,317910557	0,507545037	0,626369156	0,531072836	0,708469014	22,18390641	13,3514551	15,70522417	47,56643805	8,715505947	16,51662201	23,13392595	9,876345488	29,43570878	14,16131382	11,9234851	22,26464125	38,2749655	23,55701972	13,63661117	18,69979913	38,27855868	87,07649027	14,9807752	13,75418555	23,15661137	33,64950879	89,56569651	10,52973664	31,93712516	35,60600304
TNFSF8	44,51250547	0,336187833	0,54105588	0,621355106	0,534366004	0,711367741	22,18390641	25,3677647	31,41044834	75,54669573	18,96904235	27,52770334	18,75723725	25,78823544	44,15356318	12,98120433	39,74495032	40,60022817	32,27104934	16,82644265	19,7730862	34,28296507	114,835676	53,75091992	22,97052197	19,6488365	64,32392048	19,62888013	71,65255721	35,09912212	230,657015	38,57316996
CPA3	41,42724165	0,323123329	0,527784671	0,612225679	0,540388454	0,715396053	35,06488432	22,69747368	18,846269	75,54669573	19,994396	9,63469617	20,63296098	12,61977479	23,3754158	14,55468365	25,83421771	18,33558692	116,3258755	18,17255807	34,09152793	56,09939739	35,33405417	162,3277782	44,94232559	23,5786038	48,88617957	24,30242302	74,04097578	16,84757862	56,77711139	68,24483915
IL12RB1	60,26184591	-0,339094801	0,552692802	-0,613532146	0,539524534	0,715396053	30,05561513	60,08154796	76,95559842	50,36446382	23,58313374	48,17348085	25,00964967	24,14217786	46,7508316	21,24197073	49,6811879	49,76802163	35,27300742	28,94148137	15,68210285	24,41362664	138,3917121	59,12601191	29,96155039	38,31523118	123,5019273	42,06188599	90,7599058	54,7546305	344,2112378	35,60600304
LOX	122,472867	-0,222657264	0,365496868	-0,609190621	0,542398096	0,715396053	135,2502681	125,503678	180,6100779	135,7042497	140,4734488	89,46503586	153,8093455	150,3399258	45,88507546	66,47950098	143,0818212	112,6328911	161,3552467	115,0928678	121,3658394	58,17715285	32,38954965	110,726895	159,7949354	72,70069506	66,8968773	78,51552051	170,771928	128,462787	305,1769737	123,631955
MYB	30,0422182	-0,366136713	0,60195604	-0,608244936	0,543025036	0,715396053	22,18390641	28,03805572	26,69888109	26,58124479	18,45636553	16,51662201	12,50482484	6,584230325	19,0466351	8,654136223	27,82146523	28,81306515	21,76419607	20,86478889	8,863797261	12,98597162	55,94558576	21,50036797	13,98205685	15,7190692	51,45913639	12,15121151	47,76837147	20,35749083	220,0113066	15,82489024
RAB40C	321,3632241	-0,093985408	0,154219943	-0,609424478	0,542243117	0,715396053	309,1434699	367,1650153	359,6496335	290,9946798	402,9639808	291,7936554	255,0984266	308,3614536	322,9270405	334,3643541	361,6790479	333,9696188	318,2075563	306,2412563	381,8251128	344,9074062	270,8944153	281,6548204	370,5245065	481,3964943	275,3063797	246,7630645	334,3786003	266,7533281	280,3369875	258,143522
IGHG3	1879,112783	-0,449471604	0,744096496	-0,604050155	0,545810297	0,717576685	697,0040271	954,6290399	358,079111	97,93090187	166,1072898	81,20672486	546,4608453	483,9409289	380,0669458	563,3055941	308,023365	214,7883039	5218,153629	492,0051832	604,4427902	9712,467893	3480,404335	1768,405265	1054,646574	977,5296161	1090,933691	552,4127693	5020,455842	142,5024358	6493,882115	7397,147131
IGHG4	735,959866	-0,438898677	0,731296414	-0,600165226	0,548396126	0,719486654	247,6010199	448,6088915	142,9175399	40,57137363	66,13530983	37,16239951	244,4693255	224,4125169	156,7018615	212,4197073	129,1710886	112,6328911	2147,150515	206,6287158	240,0043566	2854,836001	1843,259826	753,5878973	388,5014367	400,8362647	437,4026593	214,9829728	2369,311225	50,54273585	2373,99297	2791,115016
GZMA	52,11933307	0,289122307	0,491036572	0,588799946	0,555995482	0,725935798	41,50537328	30,70834674	26,69888109	97,93090187	29,22257876	28,90408851	28,13585588	18,65531925	31,16722107	16,52153279	31,79596026	35,36148905	78,05091003	18,84561577	19,43217092	45,19118123	106,0021625	136,5273366	36,95257881	20,63127833	79,7616614	74,7766862	97,92516152	23,86740304	131,2970701	69,23389479
IL21R	35,36500905	0,252898495	0,430327361	0,587688624	0,556741313	0,725935798	19,32146687	24,03261919	28,2694035	53,16248959	16,91833507	33,03324401	28,13585588	17,55794753	25,10692808	13,76794399	19,87247516	27,50338037	36,02349694	24,23007742	15,34118757	18,18036027	76,55711736	76,32630629	15,97949354	26,52592928	79,7616614	39,25776026	63,2930922	25,27136792	74,5199587	41,54033688
NOTCH2	718,7928248	0,091558789	0,154907651	0,591054015	0,55448423	0,725935798	684,1230492	787,7358511	758,5623273	790,4422794	721,3362863	640,0191027	804,6854782	790,6563249	694,3364249	818,9959825	745,2178186	580,1903574	923,8525986	576,8104542	727,8541212	592,6797447	739,070633	891,1902523	684,1220672	607,149048	537,7479753	632,797707	1066,428893	581,2414623	560,673975	750,6932307
MAPK12	58,80708091	0,149788654	0,258749112	0,578895335	0,562659798	0,732149532	72,99220818	76,10329409	37,692538	82,54176015	29,22257876	77,07756936	76,90467274	58,1607012	43,28780704	70,41319927	65,57916803	36,67117383	33,77202838	62,59436667	45,34173214	65,44929696	58,89009028	75,25128789	52,93207236	56,98162586	61,75096367	36,45363452	76,42939436	60,37049004	56,77711139	59,34333839
BST2	548,0750505	0,137885028	0,240573653	0,573150993	0,566542463	0,7356942	560,3225392	461,9603466	656,4783702	688,3143389	623,4150136	602,8567032	614,6121407	520,1541957	612,0895915	519,2481734	560,4037996	437,4347164	631,1616859	325,0868721	523,645869	620,2100045	421,0641455	868,6148659	289,6283204	315,3638259	290,7441206	519,6979691	629,3482942	618,4465317	503,8968636	835,7520157
TIGIT	30,28068782	0,326170359	0,571204057	0,571022483	0,567984403	0,73606142	10,01853838	20,02718265	26,69888109	47,56643805	27,17187148	19,26939234	13,75530732	8,230287906	19,91239124	5,113807768	39,74495032	24,88401081	21,01370655	13,46115412	9,886543099	20,77755459	47,11207222	31,17553355	22,97052197	19,6488365	43,74026593	24,30242302	46,57416219	17,54956106	180,9770426	25,71544664
NFATC2	112,6739733	0,191318431	0,337276446	0,567245159	0,570547631	0,737877282	128,8097791	86,78445817	61,25037426	102,1279405	67,16066347	67,44287319	95,03666875	99,8608266	83,97834565	92,83527948	151,0308112	81,20045634	113,3239175	55,19073191	61,70566555	121,0292555	141,3362167	208,5535693	72,90643928	86,45488062	131,2207978	119,6426979	177,9371837	84,23789308	315,8226821	122,6428993
DNMT3A	241,5393372	-0,079682853	0,142592378	-0,558815656	0,576287537	0,741886498	249,0322397	275,0399751	241,8604522	251,8223191	266,5919466	180,3064569	250,0964967	205,7571977	238,9486948	252,9367996	222,5717218	237,0529451	318,9580458	211,3401197	263,5275109	246,7334608	259,1163972	268,7545996	238,6936848	172,9097612	247,0038547	205,6358871	235,2592295	193,0451716	326,4683905	220,5594077
IGLC1	2192,387982	0,310757779	0,557364443	0,557548626	0,577152646	0,741886498	668,3796317	1343,156383	697,3119531	365,1423627	1003,821214	637,2663324	969,749166	916,3053869	920,2987776	979,8842423	1047,279441	969,166737	7758,560653	895,1667492	1104,22459	6900,745318	2717,777666	2012,434442	1377,2326	1694,712148	1952,874226	672,0554672	5923,278063	451,3747104	4141,180562	8882,708702
SMAD3	672,7044381	0,134340345	0,240938695	0,557570652	0,577137602	0,741886498	665,5171922	580,788297	636,0615788	682,7182873	605,9840017	518,897208	575,2219424	790,107639	722,9063775	872,887649	1236,067955	694,1329332	1007,156935	349,9900072	574,4422456	804,0913626	597,7344163	670,8114806	571,2668941	871,4258989	566,0505003	477,6360831	703,3892699	360,1169929	844,5595319	510,3527102
CCL18	30,14728308	0,344015507	0,619402426	0,55539903	0,578621756	0,742272353	7,156098841	14,68660061	26,69888109	65,75360554	6,152121845	20,64577751	10,62910111	9,876345488	23,3754158	9,440875879	25,83421771	31,43243471	25,51664366	13,46115412	8,181966703	17,6609214	79,50162187	36,55062555	6,991028424	23,5786038	72,04279094	19,62888013	69,26413864	25,27136792	120,6513617	13,84677896
STAT5B	318,1700695	-0,063804758	0,116229344	-0,548955672	0,583035871	0,746426972	322,0244478	363,1595788	362,7906783	405,7137363	378,8681703	308,3102774	317,6225508	339,0878617	279,6392335	304,4682471	260,3294246	305,1565537	267,9247585	314,3179488	308,1874125	326,2076071	238,5048656	349,3809795	362,5347597	290,8027803	308,7548183	304,7149963	308,105996	366,4348349	294,5312653	284,8480243
CD247	27,71453932	0,229832899	0,425641516	0,539968238	0,589218937	0,750125938	19,32146687	16,02174612	31,41044834	79,74373438	17,94368871	16,51662201	20,63296098	15,91188995	25,97268422	12,58783451	21,85972268	24,88401081	24,01566463	10,7689233	12,95478061	34,28296507	38,27855868	46,22579113	18,97564858	25,54348745	33,44843865	27,10654875	53,73941791	18,95352594	31,93712516	41,54033688
IGHG1	1306,03437	-0,479985923	0,886619174	-0,541366505	0,588254989	0,750125938	322,0244478	802,4224517	436,6052319	29,37927056	117,4029919	15,14023684	560,2161526	274,3429302	351,4969931	425,2327844	192,7630091	37,98085861	4313,813758	370,1817384	439,7807103	7607,182174	1522,308834	827,7641668	1543,019845	588,4826533	720,4279094	484,1790432	2709,660872	102,4894366	5897,722446	3264,872667
MEF2C	261,7788827	0,129362215	0,239734746	0,539605614	0,589469044	0,750125938	269,0693164	244,3316284	218,3026159	337,162105	209,1721427	188,5647679	261,3508391	238,6783493	280,5049896	227,3677608	232,5079594	205,6205104	386,5021026	179,0333498	160,2301813	238,9418778	309,1729739	403,1318994	259,6667701	203,3654578	231,5661137	263,5878189	447,8284826	162,8599266	379,6969324	268,0340784
TRIM22	66,92634609	-0,148108192	0,275565184	-0,537470626	0,59094257	0,750497063	88,02001574	54,74096592	56,53880701	62,95557977	41,01414563	50,92625118	54,39598803	70,23179013	51,0796123	40,91046214	43,71944536	53,69707597	78,05091003	34,32594301	69,20580169	108,0432839	94,22414444	121,477079	51,93335401	52,06941673	90,05348868	62,62547469	91,95411508	46,3308412	99,35994493	72,20106171
SKI	305,0275197	-0,101495145	0,189709183	-0,535003863	0,592647186	0,751159607	316,2995688	340,4621051	273,2709005	225,2410743	318,8849823	257,3840262	313,2458621	305,6180243	304,7461615	340,6582713	214,6227317	328,7308797	388,7535711	234,2240817	327,2786681	262,8360656	320,950992	396,681789	248,6808682	283,9256875	218,7013296	259,8489846	539,7825976	266,0513457	361,9540851	281,8808574
RAF1	1089,478358	0,072669071	0,141593935	0,513221634	0,607796294	0,76882599	953,1923656	1126,862811	1115,070916	987,703096	1261,697655	968,9751576	1174,203052	1198,329919	1185,220157	1357,519278	1261,902173	1109,303008	1075,451482	1058,719772	1202,40819	999,9198146	1174,857301	910,5405835	919,819597	1592,538199	936,5562823	850,5848055	948,2021737	1038,934015	773,5881427	1144,337375
CD45R0	120,5449756	0,184841896	0,365126453	0,506240769	0,612687638	0,770409208	88,73562563	84,11416715	83,23768809	127,3101724	92,28182767	64,69010285	104,4152874	83,94893664	70,1262474	42,09057163	107,3113659	94,29730414	198,1292332	57,88296273	60,68291971	82,5907795	209,0598205	225,7538637	115,8513282	65,82360229	177,5340205	118,7079893	235,2592295	80,72798087	251,9484318	211,6579069
IGF2R	553,190522	0,071127493	0,140105763	0,507670001	0,61168479	0,770409208	532,4137538	484,6578202	584,2343391	360,945324	624,9530441	615,2441697	588,3520085	577,766211	604,2977862	564,8790735	528,6078393	526,4932814	487,8181877	467,7751058	543,0780399	511,6472818	580,0673892	612,7604871	679,1284755	771,2168328	545,4668457	474,8319574	558,8899462	440,8449738	542,9311277	573,6522711
SERINC5	265,1579575	0,081402002	0,160637517	0,506743405	0,612334871	0,770409208	256,1883385	226,9747368	326,6686627	391,7236075	248,1355811	253,2548707	260,1003566	261,1744696	219,9020597	211,2395978	266,2911672	205,6205104	276,1801432	227,4935047	205,5719134	352,1795503	247,3383792	318,2054459	261,6642067	249,5402236	239,2849842	275,7390304	298,5523217	247,7998022	244,8512929	322,4321386
AKR1C3	649,756432	-0,171365763	0,34467568	-0,497179734	0,619062312	0,775366109	966,0733435	606,1560617	786,8317308	1392,017819	672,6319884	831,3366409	437,043628	449,9224055	428,5492897	433,4935508	564,3782946	584,1194117	405,2643406	729,5945535	234,2087969	304,3911747	429,897659	577,2848799	934,8003722	917,6006647	777,0329595	627,1894555	499,1794819	463,308412	1302,324993	539,0353238
CSF1	113,8438323	0,134278725	0,271981234	0,493705846	0,621513936	0,775366109	95,89172447	101,4710588	111,5070916	125,9111595	87,15505947	115,616354	106,2910111	126,7464338	94,36741934	118,7976881	59,61742548	92,98761936	129,0841974	96,24725198	115,5702797	119,4709389	79,50162187	173,0779621	80,89618605	53,05185856	100,345316	126,185658	261,5318338	157,9460495	110,0056533	120,6647881
SHROOM3	292,4798602	0,113662732	0,22982721	0,494557335	0,620912625	0,775366109	231,8576024	287,0562847	309,3929161	300,78777	212,7608805	203,7050047	299,4905548	397,2485629	325,5243089	229,3346099	351,7428104	258,0079016	308,4511925	166,2452534	231,1405593	498,1418713	306,2284694	335,4057403	267,6565168	414,5904502	414,2460479	227,1341843	248,3955317	247,0978197	312,2741126	220,5594077
SLC25A15	59,57756632	0,133198915	0,268216217	0,496610222	0,619463943	0,775366109	36,49610409	57,41125694	76,95559842	67,15261843	85,61702901	48,17348085	73,15322529	66,39098911	49,34810002	70,01982944	65,57916803	60,24549987	42,02741309	78,74775162	51,47820717	30,64689302	53,00108125	52,67590152	58,92438243	81,54267149	108,0641864	42,06188599	66,87572006	54,7546305	39,03426408	32,63883612
PLK2	141,6188921	0,163219204	0,332927039	0,490255174	0,623953343	0,776883083	153,8561251	109,4819318	108,3660468	88,13781168	57,9324807	141,7676722	112,5434235	165,7031298	113,4140544	151,0540141	137,1200786	95,60698892	194,3767856	69,99800144	103,2973296	191,1535022	156,0587392	336,4807587	75,90259432	154,2433666	226,4202001	130,8592009	231,6766016	75,11212133	156,1370563	145,3911791
SDC1	1470,866078	0,214755355	0,441602517	0,486309173	0,626747976	0,778835548	1416,191961	790,4061421	1097,795169	698,1074291	2380,358477	2133,397009	1732,543481	1938,507145	785,2408196	1775,278035	2076,673654	1960,598115	524,5921742	2790,49725	2103,106358	1043,03324	524,1218035	414,9571018	2169,216248	2777,36304	951,9940232	605,6911582	1280,192355	2437,28304	734,5538786	1100,818927
TGFBR1	527,7477432	-0,094535314	0,195141329	-0,484445372	0,628069824	0,778953786	613,2776707	404,5490896	620,3563546	545,6150247	471,6626748	407,4100095	476,4338262	566,243808	380,9327019	569,2061416	532,5823343	533,0417053	581,6293776	689,2110911	433,9851505	491,3891661	453,4536951	505,2586473	809,9605789	539,360562	311,3277751	500,069089	831,1696636	518,0630425	408,0854881	527,1666561
HDAC3	438,0558016	-0,055268434	0,116363454	-0,474963851	0,634812739	0,784253092	418,6317822	480,6523837	494,7145613	449,0831357	421,4203464	373,0003803	497,0667872	432,9131439	432,8780704	426,0195241	478,9266514	432,1959773	467,5549707	403,161566	400,9163684	449,8340569	438,7311726	438,6075066	391,4975918	589,4650951	475,9970116	383,2305168	439,4690175	348,1832914	340,6626683	484,6372636
PADI4	28,9065016	0,285600973	0,599845609	0,476124137	0,633985942	0,784253092	19,32146687	16,02174612	17,27574659	30,77828345	12,81692051	22,02216267	16,88151353	10,42503135	16,44936667	8,654136223	53,65568294	26,19369559	27,76811222	16,15338495	7,500136144	15,58316594	70,66810833	21,50036797	10,98590181	11,7893019	66,8968773	13,08592009	45,3799529	18,2515435	159,6856258	15,82489024
CCR6	29,36601813	0,181542552	0,385809863	0,470549277	0,637962637	0,786614125	21,46829652	14,68660061	28,2694035	64,35459266	17,43101189	24,77493301	23,75916719	20,30137684	22,50965966	16,52153279	13,91073261	30,12274993	34,5225179	18,17255807	15,34118757	31,16633189	53,00108125	47,30080953	18,97564858	21,61372015	56,60505003	17,75946297	38,21469718	18,95352594	53,22854193	40,55128124
PIK3CD	124,4056527	-0,110722691	0,243341019	-0,455010386	0,649101767	0,798797717	132,3878286	112,1522229	147,6291072	165,0835203	115,8649614	75,70118419	133,8016257	91,08185283	125,5346404	95,98223811	103,3368708	117,8716302	199,6302122	83,45915556	93,41078652	154,2733428	141,3362167	203,1784773	97,87439794	100,2090662	123,5019273	85,05848055	140,9166958	87,74780529	138,394209	169,1285144
MEOX1	26,05142263	0,280069381	0,623244037	0,449373543	0,653162214	0,802239856	17,8902471	18,69203714	15,70522417	60,15755401	9,228182767	19,26939234	11,87958359	8,778973767	22,50965966	10,22761554	35,77045529	24,88401081	33,02153886	14,13421183	6,477390306	20,77755459	32,38954965	13,97523918	6,991028424	9,824418252	28,30252501	16,8247544	32,24365074	19,65550839	173,8799036	13,84677896
SELP	68,91959299	0,190759581	0,432839997	0,440716159	0,659418497	0,808360512	40,78976339	62,75183899	65,96194151	103,5269534	26,65919466	77,07756936	47,51833437	59,25807293	38,09327019	76,70711652	39,74495032	53,69707597	121,5793022	50,47932796	43,97807103	65,96873582	114,835676	201,0284405	14,9807752	31,43813841	43,74026593	88,79731486	125,3919751	50,54273585	60,32568085	87,03689631
IRF4	55,7227057	0,236833638	0,541919343	0,437027468	0,662091427	0,810073326	26,47756571	29,37320123	43,97462767	81,14274727	15,89298143	39,91516985	11,87958359	23,04480614	26,83844036	20,45523107	73,5281581	48,45833685	75,79944147	17,49950036	13,63661117	77,39639085	85,3906309	59,12601191	27,9641137	37,33278936	84,90757504	38,32305168	106,2846265	35,09912212	259,0455707	90,00406323
OR2I1P	25,47366859	0,242421828	0,561675744	0,431604588	0,666028823	0,813323659	12,88097791	20,02718265	25,12835867	55,96051536	9,740859588	17,89300717	8,128136143	16,46057581	21,64390352	7,08065691	21,85972268	15,71621736	21,01370655	12,78809642	7,841051423	14,02484935	41,22306319	29,02549676	11,98462016	18,66639468	56,60505003	23,36771444	27,4668136	19,65550839	131,2970701	14,8358346
BMPER	26,13354015	-0,237881376	0,55483896	-0,428739497	0,668112814	0,814302566	15,02780757	30,70834674	23,55783625	19,58618037	8,202829126	28,90408851	9,378618626	13,71714651	19,0466351	14,16131382	35,77045529	28,81306515	14,25930087	14,80726954	7,159220865	19,219238	64,7790993	35,47560715	10,98590181	14,73662738	28,30252501	8,412377198	37,02048789	11,93370152	134,8456396	30,66072484
CXCR6	40,328736	0,228418872	0,543766676	0,420067802	0,674435923	0,820434505	32,20244478	25,3677647	26,69888109	75,54669573	21,53242646	23,39854784	25,00964967	12,07108893	26,83844036	8,260766395	35,77045529	26,19369559	30,7700703	11,441981	16,36393341	27,53025983	85,3906309	45,15077273	20,97308527	27,5083711	61,75096367	39,25776026	65,68151077	30,88722746	209,3655982	37,58411432
EPAS1	1103,666558	-0,076366128	0,187592418	-0,407085365	0,683945292	0,830411588	1006,863107	1125,527665	1254,847411	1268,904686	1057,65228	801,0561673	1174,203052	1127,000757	1083,926688	1051,084181	727,3325909	1111,922378	1138,492601	1121,314138	1300,932706	1074,719011	974,6309941	1176,070128	1294,338977	834,0931096	1147,538741	867,4095599	1631,289886	1604,731863	613,9025169	1125,545318
SLAMF7	25,66696252	0,244279264	0,602934444	0,405150621	0,685366799	0,830549461	12,88097791	16,02174612	17,27574659	53,16248959	10,76621323	8,258311003	12,50482484	7,681602046	17,31512281	5,507177596	7,948990065	17,02590214	60,03916156	6,057519356	6,136475027	44,1523035	76,55711736	34,40058875	11,98462016	11,7893019	33,44843865	28,04125733	46,57416219	8,423789308	46,131403	67,25578351
AGER	33,28558798	-0,161924272	0,411929753	-0,3930871	0,694255151	0,838121713	18,60585699	29,37320123	39,26306042	32,17729633	20,50707282	23,39854784	21,88344346	31,27509404	19,91239124	12,19446468	19,87247516	31,43243471	29,26909126	14,13421183	25,56864595	96,09618998	47,11207222	36,55062555	19,97436693	25,54348745	33,44843865	22,43300586	51,35099933	30,88722746	106,4570839	26,70450228
RASIP1	119,2253624	0,098256767	0,249522894	0,393778565	0,693744531	0,838121713	103,7634332	89,45474919	108,3660468	207,0539068	84,07899854	104,6052727	69,40177784	103,7016276	96,96468776	99,91593639	107,3113659	120,4909997	130,5851764	89,51667492	108,7519741	175,0508974	141,3362167	160,1777414	74,90387598	77,61290419	162,0962796	111,2303207	131,3630215	124,9528747	209,3655982	107,8070648
KLRF1	28,8589031	-0,246574662	0,63306798	-0,389491603	0,696912521	0,839733304	17,8902471	29,37320123	18,846269	18,18716749	7,177475486	26,15131817	11,87958359	6,584230325	17,31512281	10,22761554	41,73219784	32,74211949	18,01174847	10,09586559	6,477390306	11,42765502	94,22414444	27,95047836	12,9833385	16,70151103	56,60505003	17,75946297	39,40890646	20,35749083	170,3313342	9,890556399
CCR1	87,86762149	-0,140688229	0,366111524	-0,384276975	0,700773171	0,842785916	63,68927968	105,4764953	83,23768809	93,73386322	40,50146881	70,19564352	60,64840045	75,71864874	53,67688073	39,33698283	83,46439568	68,10360854	99,8151061	65,2865975	32,04603625	57,13827512	147,2252257	188,1282197	71,90772094	55,99918404	87,48053186	147,6839552	144,4993237	49,84075341	163,2341952	136,4896783
PF4	29,64803328	0,206370146	0,547653679	0,376826001	0,706302917	0,847337204	15,74341745	21,36232817	28,2694035	50,36446382	16,40565825	20,64577751	12,50482484	12,61977479	22,50965966	11,80109485	23,84697019	18,33558692	14,25930087	18,17255807	10,22745838	24,41362664	73,61261284	23,65040477	8,988465117	13,75418555	100,345316	25,23713159	40,60311575	20,35749083	117,1027922	25,71544664
PRDM1	49,77161635	-0,134853698	0,35905088	-0,375583811	0,707226328	0,847337204	37,92732386	61,41669347	45,54515009	81,14274727	39,47611517	42,66794018	50,64454058	22,49612028	26,83844036	16,52153279	41,73219784	45,83896729	48,03132925	26,24925054	30,00054458	64,41041923	53,00108125	70,9512143	43,94360724	34,38546388	87,48053186	42,99659457	82,40044079	30,18524502	106,4570839	61,32144967
PLAU	408,6575458	0,174689871	0,470502128	0,371283912	0,710426079	0,84956791	356,3737223	487,3281113	274,8414229	324,5709891	129,1945587	206,4577751	218,2091934	538,8095149	356,69153	963,7560794	421,2964734	324,8018254	1588,035823	162,8799649	233,5269663	202,0617184	456,3981996	483,7582793	300,6142223	326,170686	370,505782	742,1586106	352,2917396	212,70068	230,657015	361,0053086
CD70	39,74477969	0,229318762	0,627950155	0,36518625	0,714972382	0,85140457	24,33073606	26,70291021	25,12835867	60,15755401	11,27889005	35,78601434	21,25820222	14,26583237	32,03297721	8,654136223	29,80871274	30,12274993	21,76419607	14,13421183	9,886543099	24,41362664	91,27963993	53,75091992	11,98462016	16,70151103	59,17800685	22,43300586	63,2930922	31,58920991	269,6912791	23,73733536
HLA-DPA1	860,3337451	-0,14888983	0,406699769	-0,366092733	0,71429588	0,85140457	828,6762458	826,4550709	796,2548653	875,7820653	892,0576675	708,8383611	1037,27522	546,491117	470,1055844	339,084792	357,7045529	789,7399222	1739,634706	417,2957778	553,6464135	859,6713212	1587,087933	2371,490587	560,2809923	497,1155635	450,2674434	1199,231105	1056,875219	595,2811111	408,0854881	1604,248248
TGFB2	50,38009025	-0,126090518	0,346563558	-0,363830862	0,715984316	0,85140457	47,94586223	50,73552939	51,82723976	79,74373438	24,60848738	33,03324401	41,8911632	36,21326679	37,22751405	41,30383197	39,74495032	44,52928251	70,54601484	21,5378466	34,43244321	39,99679259	88,33513541	65,5761223	35,95386047	24,56104563	59,17800685	45,8007203	66,87572006	32,29119235	127,7485006	68,24483915
IL1RN	26,31656527	0,207268641	0,576681169	0,359416349	0,719283646	0,852555687	13,5965878	17,35689163	18,846269	47,56643805	10,25353641	13,76385167	15,0057898	14,26583237	23,3754158	18,48838193	19,87247516	28,81306515	15,76027991	8,74975018	7,500136144	17,14148254	73,61261284	19,35033117	8,988465117	11,7893019	38,59435229	20,56358871	32,24365074	23,86740304	149,0399174	15,82489024
TM4SF18	256,1982034	0,09160153	0,255197818	0,358943234	0,719637556	0,852555687	224,7015036	311,0889039	251,2835867	302,1867829	259,9271479	227,1035526	215,7082284	375,8498144	204,3184492	336,724573	131,1583361	196,452717	288,938465	201,2442541	203,867337	209,8533014	267,9499108	550,40942	191,7539225	209,2601088	275,3063797	343,9727565	205,4039973	249,9057495	202,2684593	224,5156303
COL4A3	284,3080057	0,072097246	0,207366499	0,3476803	0,728080293	0,857756932	276,9410251	234,9856098	276,4119454	254,6203449	296,3272022	250,5021004	335,1293056	270,5021292	284,8337703	186,8506684	439,1817011	299,9178145	237,1546882	208,6478889	295,9144624	443,0813516	235,5603611	316,0554091	365,5309148	259,3646418	329,3384729	230,8730186	333,184391	325,7198533	156,1370563	249,2420213
HLA-DPB1	685,658561	-0,134208742	0,385169681	-0,348440568	0,72750934	0,857756932	676,9669503	543,4042227	667,4720272	563,8021922	478,8401502	408,7863946	835,322299	545,3937453	438,9383634	283,6196462	383,5387706	574,9516183	1494,224633	473,1595674	525,0095301	502,8168211	1089,46667	1836,131424	450,4219742	560,9742822	375,6516956	899,1896516	1462,906376	534,9106211	251,9484318	969,2745271
TNFRSF4	38,76960241	0,161336143	0,460439049	0,350396309	0,726041296	0,857756932	20,75268664	21,36232817	42,40410525	27,98025768	15,89298143	38,53878468	26,8853734	34,56720921	38,09327019	13,37457416	41,73219784	34,05180427	41,27692357	25,57619283	10,22745838	23,89418778	79,50162187	35,47560715	24,96795866	33,40302206	59,17800685	25,23713159	66,87572006	30,88722746	163,2341952	32,63883612
BCL2L1	1090,688121	0,070874857	0,209912652	0,337639755	0,73563468	0,865051892	943,8894371	1113,511356	953,307107	678,5212487	824,3843272	1030,91249	1224,847593	1257,039306	1076,134883	1103,402368	1456,652429	1122,399856	1089,710782	1062,758118	1156,725543	711,1118058	1454,58523	1052,443012	1184,479959	1786,079238	977,7235914	868,3442685	1584,715724	1007,344805	706,1653229	930,7013572
GIMAP5	299,5226665	0,069134771	0,214852239	0,321778222	0,747620714	0,876616799	200,3707675	308,4186129	345,5149317	235,0341645	354,2596829	216,0924712	340,7564768	322,6272859	312,5379668	285,5864954	345,7810678	298,6081298	288,1879755	207,9748312	290,8007332	434,7703298	214,9488295	318,2054459	319,5898708	269,1890601	303,6089047	212,1788471	519,4810398	299,7465029	195,1713204	348,1475852
IL2RG	38,89593251	0,140820422	0,438728995	0,320973594	0,748230402	0,876616799	22,89951629	21,36232817	31,41044834	29,37927056	18,96904235	17,89300717	29,38633836	21,39874856	31,16722107	25,17566901	17,88522765	31,43243471	57,03720349	11,441981	15,34118757	53,50220307	55,94558576	93,52660066	25,96667701	29,47325476	41,16730911	49,53955461	93,14832437	17,54956106	67,42281978	101,8727309
CDH13	48,49447936	-0,122053185	0,388068568	-0,314514483	0,753130318	0,877500462	33,63366455	62,75183899	72,24403117	75,54669573	24,60848738	38,53878468	30,63682085	57,06332948	41,55629476	39,33698283	51,66843542	35,36148905	33,02153886	16,82644265	39,20525712	69,08536901	91,27963993	73,10125109	19,97436693	22,59616198	54,03209321	40,19246883	88,37148722	34,39713968	85,16566708	30,66072484
KLF4	149,1630551	-0,113698621	0,360729758	-0,315190579	0,752616958	0,877500462	159,5810042	214,9584272	109,9365692	165,0835203	74,85081578	83,95949519	112,5434235	137,1714651	71,85775968	141,6131382	139,1073261	73,34234766	114,8248965	102,977829	142,5025867	195,828452	144,2807212	452,5827457	97,87439794	77,61290419	169,8151501	140,2062866	275,8623453	163,5619091	173,8799036	142,4240121
SFTPB	26,80864541	-0,169863295	0,536100565	-0,316849684	0,751357662	0,877500462	18,60585699	20,02718265	17,27574659	26,58124479	10,25353641	27,52770334	15,63103104	17,00926167	20,77814738	10,62098536	21,85972268	41,90991295	14,25930087	10,09586559	11,5911195	16,10260481	32,38954965	23,65040477	12,9833385	14,73662738	59,17800685	13,08592009	46,57416219	20,35749083	156,1370563	17,80300152
CIITA	227,5647894	-0,093105296	0,303658012	-0,30661235	0,759138452	0,878277057	166,0214931	157,5471702	229,2962729	246,2262676	127,1438515	192,6939234	126,2987308	153,0833551	138,5209825	94,01538897	226,5462168	247,5304234	339,2212628	169,610542	143,5253326	355,2961835	479,9542357	437,5324882	141,8180052	208,2776669	133,7937546	261,7184017	305,7175774	222,5284342	365,5026546	247,26391
CXCL9	51,54288147	-0,170044048	0,555043501	-0,306361659	0,759329298	0,878277057	27,90878548	26,70291021	81,66716567	83,94077303	24,09581056	27,52770334	26,8853734	32,37246576	30,30146493	16,52153279	59,61742548	55,00676075	41,27692357	16,82644265	12,61386533	16,10260481	179,6147753	74,17626949	23,96924031	51,08697491	54,03209321	69,16843474	47,76837147	66,68833202	177,4284731	16,81394588
MMRN2	173,4520723	0,073710511	0,237548323	0,310296914	0,756335178	0,878277057	128,0941693	158,8823157	180,6100779	130,1081982	96,89591905	227,1035526	141,9297619	151,9859833	209,5129861	202,5854616	153,0180587	174,1880757	173,363079	127,8809642	152,7300451	155,3122206	250,2828837	281,6548204	118,8474832	161,1204593	195,5447183	168,247544	309,3002053	153,0321724	138,394209	169,1285144
SP100	293,0751122	0,044429325	0,1446498	0,307150962	0,758728469	0,878277057	281,9502943	313,7591949	295,2582144	288,1966541	313,2455373	232,6090932	319,4982745	315,4943697	339,3764072	335,1510937	242,444197	269,7950646	320,4590248	247,6852359	259,7774428	381,7875656	276,7834243	335,4057403	331,574491	245,6104563	257,2956819	292,5637848	361,8454139	207,7868029	251,9484318	302,6510258
OAZ1	2591,801357	0,045303476	0,15535353	0,291615363	0,770580728	0,881415823	2915,394668	2372,553572	2409,181387	3157,572079	2667,457497	2948,217028	3054,303466	3099,526426	2489,048905	2788,205343	1699,096626	2625,917983	2412,823805	2900,878714	2626,752227	1581,171904	2473,383792	2613,369727	2526,757416	2804,871411	2938,316688	2729,349046	1862,966487	2805,12184	2086,558844	2798,038405
TBP	216,6020953	-0,039550982	0,135508971	-0,291869842	0,770386143	0,881415823	231,8576024	214,9584272	246,5720194	244,8272547	261,4651784	205,0813899	215,0829872	198,0755956	187,8690825	213,2064469	186,8012665	197,7624017	244,6595834	228,1665624	219,8903551	293,4829586	194,3372979	217,1537165	207,733416	161,1204593	239,2849842	183,2028812	223,3171366	191,6412068	234,2055845	189,8986829
BMP2	90,41497941	0,104666136	0,356506555	0,293588252	0,769072554	0,881415823	70,12976864	108,1467863	108,3660468	200,0588424	72,28743168	49,54986602	58,14743548	76,81602046	45,01931932	76,70711652	87,43889071	44,52928251	98,31412706	53,17155879	87,95614205	165,181559	61,83459479	176,3030173	98,87311629	38,31523118	105,4912296	73,84197762	107,4788358	77,9200511	110,0056533	98,90556399
CCL13	38,04464943	0,153411292	0,518566389	0,295837322	0,767354319	0,881415823	18,60585699	25,3677647	28,2694035	50,36446382	11,27889005	23,39854784	22,5086847	26,88560716	34,63024563	19,66849142	21,85972268	39,29054339	42,02741309	22,2109043	11,25020422	17,14148254	97,16864896	37,62564394	14,9807752	22,59616198	54,03209321	29,91067448	50,15679005	28,07929769	202,2684593	37,58411432
CD40	135,6893906	0,072291546	0,250785566	0,288260393	0,773147423	0,881415823	115,9288012	128,173969	117,7891813	135,7042497	100,4846568	132,132976	80,03087895	117,96746	126,4003965	95,98223811	166,9287914	117,8716302	135,838603	78,07469392	85,22881982	209,8533014	135,4472076	139,7523918	124,8397933	117,893019	210,9824592	148,6186638	146,8877423	102,4894366	269,6912791	186,9315159
CD46	2130,090617	-0,038937311	0,129493098	-0,300690244	0,763650709	0,881415823	2278,501871	2144,24369	2574,086241	1950,22396	2441,367019	2089,352684	2072,049475	2064,156207	2402,473291	2432,599018	1891,859635	1913,449463	2206,439187	2400,12378	2265,041115	2499,539817	1790,258744	2263,988747	2704,529282	1626,923662	1994,041535	2101,224882	1858,18965	2025,921329	1486,850605	1904,921162
HFE	81,87673767	-0,078022791	0,270527888	-0,288409419	0,773033359	0,881415823	80,14830702	76,10329409	78,52612084	149,6943786	61,00854163	92,2178062	63,77460666	58,70938707	86,57561407	54,67840614	77,50265313	98,22635848	80,30237859	63,94048209	49,43271549	49,86613102	100,1131535	94,60161906	62,91925582	86,45488062	84,90757504	58,88664038	109,8672544	68,79427935	170,3313342	71,21200607
RAPGEF5	294,6413251	0,086215684	0,299076941	0,288272588	0,773138088	0,881415823	249,0322397	312,4240494	240,2899298	356,7482854	274,7947757	210,5869306	255,0984266	225,5098886	265,7871352	190,3909969	284,1763948	237,0529451	361,7359484	218,7437545	244,09534	686,6981792	285,6169378	383,7815682	280,6398553	150,3135993	221,2742865	243,0242302	592,3278063	228,8462762	464,8625995	196,8220723
FADD	271,8355374	0,04405848	0,154805201	0,284605945	0,775946056	0,881441405	208,9580862	304,4131764	241,8604522	214,0489712	269,1553307	257,3840262	300,7410373	280,3784747	264,0556229	243,1025539	353,7300579	294,6790754	263,4218214	283,3572943	260,8001886	183,3619193	344,5070281	266,6045628	248,6808682	336,977546	355,0680411	235,5465615	246,0071131	226,0383464	315,8226821	269,0231341
IGF1	83,46706305	-0,149940308	0,52679586	-0,284626968	0,775929948	0,881441405	72,99220818	122,8333869	108,3660468	106,3249792	19,994396	23,39854784	156,9355517	34,01852335	77,91805267	51,53144751	119,234851	47,14865207	52,53426637	96,24725198	23,52315427	52,46332534	100,1131535	152,6526126	36,95257881	47,15720761	66,8968773	62,62547469	82,40044079	31,58920991	120,6513617	303,6400815
CD96	35,9158419	-0,139440555	0,493551232	-0,282524985	0,777540991	0,881567256	20,03707675	34,71378327	32,98097075	41,97038652	15,89298143	17,89300717	26,26013215	14,26583237	24,24117194	10,22761554	19,87247516	24,88401081	34,5225179	12,11503871	10,22745838	38,95791486	61,83459479	75,25128789	23,96924031	17,68395285	43,74026593	28,9759659	88,37148722	25,27136792	131,2970701	58,35428275
RASSF9	89,42353429	0,068751464	0,244806877	0,280839594	0,778833434	0,881567256	100,9009937	93,46018572	87,94925534	78,3447215	85,61702901	67,44287319	78,78039646	72,42653358	105,6222492	101,8827855	101,3496233	58,93581509	171,8621	59,90213585	68,86488641	97,13506771	94,22414444	99,97671105	84,89105944	82,52511331	72,04279094	79,45022909	124,1977658	47,73480608	113,5542228	95,93839707
COL4A5	126,2785124	0,050346887	0,183413477	0,274499388	0,783700874	0,885498318	100,1853838	137,5199876	150,770152	152,4924043	120,9917296	115,616354	128,1744546	122,3569469	194,7951317	139,6462891	151,0308112	128,3491084	110,3219594	87,4975018	131,9342131	131,9374716	123,6691896	105,351803	142,8167235	111,0159262	172,3881069	88,79731486	113,4498822	149,5222602	81,61709762	90,99311887
CXCR4	151,7672475	0,124502922	0,460710265	0,270241258	0,786974654	0,887617949	100,1853838	129,5091145	130,3533606	90,93583745	105,0987482	81,20672486	148,8074155	88,88710939	113,4140544	50,35133802	117,2476035	64,1745542	188,3728694	42,40263549	128,8659756	486,1947774	156,0587392	188,1282197	110,8577364	83,50755514	110,6371432	100,0138178	512,315784	85,64185797	269,6912791	263,0888002
CDH5	127,798158	-0,065686083	0,247069473	-0,265860782	0,79034643	0,889840395	130,2409989	113,4873684	163,3343313	198,6598295	78,95223034	92,2178062	113,793906	113,5779731	123,8031281	158,5280408	103,3368708	117,8716302	159,1037781	76,0555208	108,4110588	182,8424804	156,0587392	181,6781093	107,8615814	82,52511331	131,2207978	85,05848055	154,052998	107,4033137	173,8799036	108,7961204
CFLAR	621,1370023	-0,041059853	0,163018592	-0,251872211	0,801139839	0,89634723	646,9113352	670,2430462	697,3119531	609,9696174	923,8436304	543,672141	567,7190475	804,9221572	523,7824651	614,4436718	586,2380173	606,384053	575,6254615	555,2726076	810,3556188	691,8925679	432,8421635	594,4851743	757,0285065	493,1857962	586,6341548	472,0278317	729,6618742	599,4930058	496,7997247	558,8164365
PLA1A	87,25845467	0,074627672	0,292942214	0,254752195	0,7989145	0,89634723	74,42342794	81,44387613	103,6544795	139,9012884	86,12970583	67,44287319	121,2968009	72,42653358	69,26049126	54,28503631	57,63017797	68,10360854	91,55972138	96,92030969	46,36447798	70,12424674	108,946667	133,3022814	71,90772094	43,22744031	138,9396682	101,883235	111,0614637	65,28436714	134,8456396	58,35428275
PLAT	403,3955008	0,088342777	0,350931168	0,251738189	0,801243437	0,89634723	400,0259252	289,7265757	617,2153098	286,7976412	425,5217609	279,4061889	363,2651615	453,2145207	492,6152441	518,8548035	337,8320777	243,601369	249,1625205	331,1443914	533,1914968	842,0103998	253,2273882	332,1806851	661,1515453	183,7166213	295,8900342	214,9829728	751,1576414	609,32076	216,4627372	306,6072484
TLR9	40,80394657	0,12692163	0,505554191	0,251054451	0,801772011	0,89634723	32,20244478	30,70834674	29,83992592	48,96545094	21,53242646	53,67902152	18,13199601	17,55794753	29,43570878	20,45523107	41,73219784	34,05180427	42,77790261	19,51867348	20,45491676	17,6609214	58,89009028	31,17553355	19,97436693	18,66639468	61,75096367	30,84538306	70,45834792	37,20506944	223,5598761	29,6716692
CD44	555,8320353	0,104277141	0,418411884	0,249221269	0,803189628	0,896353978	400,0259252	520,706749	544,9712786	272,8075124	231,217246	196,8230789	414,5349433	1011,228041	400,8450932	450,0150836	1077,088154	512,0867489	863,0629475	236,2432549	248,1863233	845,6464718	800,9052278	981,4917978	338,5655194	540,3430038	429,6837888	493,5261289	1102,255172	217,6145571	762,9424343	558,8164365
IKZF2	81,73445983	0,052277511	0,221836745	0,23565758	0,813698389	0,906488556	65,83610934	80,10873062	64,39141909	96,53188899	61,52121845	61,93733252	66,27557163	71,32916185	95,23317548	59,39884407	63,59192052	81,20045634	80,30237859	72,69023227	57,6146822	75,31863539	141,3362167	102,1267478	61,92053747	74,66557871	79,7616614	73,84197762	75,23518507	89,15177018	173,8799036	99,89461963
CD38	44,89250344	-0,106026983	0,465746872	-0,227649372	0,819918834	0,911818669	29,34000525	32,04349225	51,82723976	34,9753221	12,30424369	27,52770334	35,63875078	25,23954958	49,34810002	26,74914833	35,77045529	40,60022817	45,02937117	25,57619283	14,65935701	39,99679259	70,66810833	52,67590152	39,94873385	22,59616198	41,16730911	39,25776026	81,2062315	27,37731525	227,1084456	38,57316996
SMAD5	254,7336571	-0,023296215	0,105904515	-0,219973762	0,825891589	0,916855173	259,7663879	273,7048296	298,3992592	264,4134351	276,3328062	257,3840262	269,4789752	277,6350454	283,9680142	256,0837582	204,6864942	230,5045212	250,6634995	234,2240817	249,5499844	216,6060066	206,115316	297,7800964	236,6962481	197,4708069	272,7334229	240,2201044	271,0855081	259,7335037	294,5312653	243,3076874
MICB	151,060737	0,039469289	0,184264579	0,214199005	0,830391872	0,918639092	146,7000262	157,5471702	128,7828382	209,8519326	131,7579428	140,391287	172,5665827	151,4372975	135,057958	167,9689167	139,1073261	161,0912279	145,5949668	100,9586559	171,1394702	191,1535022	188,4482889	187,0532013	127,8359483	142,4540647	146,6585387	93,47085775	193,4619045	122,8469274	152,5884869	121,6538437

SERTAD1	136,568079	0,04657894	0,21593641	0,215706745	0,829216344	0,918639092	161,0122239	153,5417337	120,9302261	169,280559	124,0677905	156,907909	105,6657699	162,4110147	98,69620004	111,3236614	184,814019	102,1554128	143,3434982	92,88196345	97,84268515	108,0432839	200,2263069	138,6773734	74,90387598	135,5769719	133,7937546	160,7698753	151,6645794	121,4429625	184,525612	156,2707911
AQP2	666,1956843	-0,107557388	0,513096816	-0,209623963	0,833961176	0,918772102	505,936188	1045,418935	708,30561	169,280559	595,7304653	627,6316362	1220,470904	176,6768471	1265,735478	2291,77262	133,1455836	804,1464547	333,2173467	597,6752431	730,2405282	171,9342642	630,123966	459,0328561	913,8272869	422,4499848	439,9756161	285,0861161	976,8631966	953,2921567	330,01696	533,1009899
B3GAT1	33,5226087	0,113148699	0,543910365	0,208028209	0,835206941	0,918772102	20,03707675	38,7192198	20,41679142	26,58124479	17,94368871	34,40962918	13,13006608	14,26583237	32,89873335	15,3414233	49,6811879	24,88401081	30,7700703	15,48032724	8,863797261	16,10260481	70,66810833	32,25055195	17,97693023	21,61372015	61,75096367	32,71480021	38,21469718	21,05947327	180,9770426	14,8358346
FYN	267,5008152	-0,039606711	0,191680352	-0,206628954	0,836299646	0,918772102	249,0322397	267,0291021	348,6559765	307,7828345	317,8596286	267,0187224	247,5955317	255,687611	263,1898668	169,542396	343,7938203	248,8401081	267,9247585	167,5913688	286,0279193	295,0412752	253,2273882	340,7808323	256,670615	267,2241764	200,6906319	186,9417155	385,7295996	256,2235915	202,2684593	302,6510258
JAK2	213,6092051	0,032366627	0,156608492	0,206672233	0,836265844	0,918772102	213,2517455	209,6178451	257,5656764	226,6400872	251,211642	144,5204425	220,7101583	177,7742188	248,4720124	230,9080892	236,4824544	176,8074453	232,6517511	185,7639269	234,2087969	259,7194324	191,3927934	260,1544524	237,6949664	165,0502266	146,6585387	180,3987555	247,2013224	197,9590487	209,3655982	211,6579069
CAV1	96,94813546	0,054862252	0,278323277	0,197117011	0,843735972	0,923745417	104,4790431	90,7898947	73,81455359	78,3447215	88,18041311	52,30263635	94,41142751	125,1003762	83,97834565	90,86843034	93,40063326	86,43919546	120,8288126	78,74775162	102,6154991	112,7182337	97,16864896	97,82667426	110,8577364	32,42058023	113,2101001	77,58081193	152,8587887	101,0854717	202,2684593	58,35428275
SFTPC	25,23315514	0,114920374	0,578021336	0,198816838	0,842406024	0,923745417	13,5965878	21,36232817	21,98731384	68,55163131	9,740859588	13,76385167	11,25434235	8,778973767	21,64390352	12,58783451	23,84697019	26,19369559	15,76027991	8,74975018	8,181966703	12,46653275	47,11207222	20,42534957	15,97949354	9,824418252	64,32392048	19,62888013	31,04944146	27,37731525	110,0056533	11,86866768
IGHM	447,6499096	-0,107738152	0,561844237	-0,191758045	0,847931737	0,925636528	243,3073606	292,3968668	347,0854541	166,4825332	185,0763322	203,7050047	108,7919761	223,3151452	231,1568896	187,2440383	296,0998799	184,6655539	1376,397779	99,61254051	131,2523825	405,1623145	1254,358923	169,8529069	215,7231628	372,3454517	542,8938889	488,852586	1230,035565	121,4429625	1171,027922	1390,61223
PTGS2	54,62252185	-0,080770213	0,422465911	-0,191187527	0,848378676	0,925636528	49,377082	44,05980184	50,25671734	88,13781168	33,83667015	49,54986602	33,76302706	25,23954958	55,40839301	29,89610695	31,79596026	56,31644553	70,54601484	24,90313513	50,11454605	49,34669215	100,1131535	41,92571754	32,95770543	23,5786038	54,03209321	59,82134896	52,54520862	37,90705189	234,2055845	40,55128124
IDO1	42,97865226	-0,090557804	0,482412018	-0,187718798	0,851097094	0,927009699	40,78976339	37,38407429	37,692538	53,16248959	23,58313374	33,03324401	23,13392595	14,81451823	33,76448949	13,76794399	53,65568294	31,43243471	30,7700703	16,82644265	15,34118757	49,86613102	88,33513541	37,62564394	24,96795866	26,52592928	66,8968773	40,19246883	48,96258076	40,01299921	202,2684593	32,63883612
HYAL1	935,1728848	0,043735487	0,244329363	0,179002174	0,857935995	0,92967467	1077,708485	863,8391452	954,8776294	892,5702199	1358,080897	1288,296516	1051,030527	755,5404298	1026,786783	1110,089655	695,5366307	910,2309219	914,0962348	1356,211278	1517,072993	455,5478844	797,9607232	699,8369774	952,7773024	952,9685704	789,8977436	763,6569078	662,7861542	902,7494209	450,6683217	1113,676651
IL21	31,65796784	0,118557589	0,655774483	0,180790183	0,856532269	0,92967467	17,8902471	22,69747368	25,12835867	41,97038652	7,690152306	23,39854784	10,00385987	9,876345488	20,77814738	7,08065691	37,75770281	19,6452717	20,26321703	14,13421183	6,818305585	14,02484935	58,89009028	24,72542316	15,97949354	14,73662738	66,8968773	23,36771444	52,54520862	25,27136792	230,657015	10,87961204
VEGFC	103,9815648	-0,036602239	0,204204774	-0,179242818	0,857747044	0,92967467	88,02001574	105,4764953	103,6544795	124,5121467	83,0536449	97,72334686	96,28715123	98,76345488	94,36741934	77,88722601	83,46439568	106,0844672	132,836645	96,92030969	76,36502256	82,5907795	161,9477483	132,227263	106,8628631	59,92895134	100,345316	120,5774065	150,4703701	81,42996331	117,1027922	124,6210106
CCL3/L1	39,39300532	0,093478164	0,56571879	0,165237863	0,868756764	0,937507352	32,20244478	36,04892878	25,12835867	90,93583745	12,30424369	66,06648802	21,25820222	26,3369213	31,16722107	9,834245708	29,80871274	44,52928251	15,76027991	8,076692474	11,93203477	24,93306551	55,94558576	55,90095672	16,97821189	33,40302206	108,0641864	28,04125733	50,15679005	18,95352594	134,8456396	35,60600304
CD2	31,01631751	0,095589167	0,579605046	0,164921213	0,869005996	0,937507352	15,02780757	17,35689163	28,2694035	32,17729633	12,30424369	24,77493301	14,38054856	10,97371721	17,31512281	11,01435519	31,79596026	26,19369559	30,7700703	14,13421183	5,454644468	18,69979913	58,89009028	43,00073594	9,987183463	10,80686008	59,17800685	28,9759659	74,04097578	14,03964885	163,2341952	33,62789176
PDGFA	177,4175718	0,040418543	0,246774853	0,16378712	0,869898731	0,937507352	206,8112565	138,8551331	191,6037349	202,8568682	190,7157772	101,8525024	135,0521082	186,5531925	86,57561407	177,0164227	264,3039196	138,8265866	234,1527301	149,4188108	180,685098	233,2280503	241,4449701	202,1034589	165,7872455	131,6472046	126,0748841	159,8351668	298,5523217	147,4163129	163,2341952	158,2489024
ZAP70	42,8633844	-0,067528431	0,416072342	-0,162299735	0,871069823	0,937507352	39,35854362	40,05436531	26,69888109	58,75854112	27,17187148	27,52770334	30,63682085	21,94743442	27,7041965	16,52153279	23,84697019	35,36148905	53,28475589	12,11503871	24,54590011	65,44929696	70,66810833	92,45158226	33,95642378	39,29767301	28,30252501	36,45363452	109,8672544	26,67533281	56,77711139	89,01500759
BCL2	250,9241538	-0,033658667	0,211027307	-0,159499108	0,873275661	0,938291108	192,4990588	344,4675417	260,7067212	223,8420614	187,1270394	180,3064569	286,3604887	279,281103	351,4969931	257,6572375	276,2274047	243,601369	320,4590248	148,7457531	191,9353022	274,2637206	220,8378385	349,3809795	242,6885582	297,679873	306,1818615	199,092927	256,7549967	178,3035404	188,0741815	266,0559671
ADAM8	37,04903763	0,063456018	0,412756137	0,15373731	0,877816865	0,941577212	22,89951629	36,04892878	32,98097075	19,58618037	22,04510328	39,91516985	25,63489091	21,39874856	23,3754158	23,99555953	45,70669287	26,19369559	28,51860174	22,2109043	13,63661117	34,80240394	67,72360382	43,00073594	21,97180362	28,49081293	69,46983412	26,17184017	52,54520862	30,88722746	145,4913479	38,57316996
CX3CR1	57,20127451	-0,083482514	0,552803827	-0,151016527	0,879962679	0,941622559	63,68927968	57,41125694	47,1156725	155,2904301	37,42540789	38,53878468	55,02122928	56,51464362	32,89873335	23,99555953	15,89798013	60,24549987	141,0920297	36,34511613	16,02301813	22,85531005	153,1142347	87,07649027	26,96539535	55,01674221	33,44843865	77,58081193	76,42939436	12,63568396	10,64570839	93,96028579
GZMK	31,55851362	-0,070448347	0,46989494	-0,149923613	0,880824882	0,941622559	19,32146687	32,04349225	9,423134501	29,37927056	22,04510328	13,76385167	16,25627229	10,42503135	22,50965966	10,62098536	27,82146523	37,98085861	24,01566463	15,48032724	11,25020422	15,06372708	67,72360382	49,45084633	32,95770543	17,68395285	43,74026593	36,45363452	68,06992935	22,46343816	117,1027922	47,47467072
HMGB1	838,9201348	0,024993713	0,171607737	0,145644442	0,884202083	0,943644239	853,7225917	894,5474919	965,8712863	1068,845843	925,8943376	734,9896792	645,8742027	917,9514445	835,4546758	1000,732843	878,3634021	707,229781	787,263506	838,6299019	669,5576085	829,543867	553,5668486	857,8646819	1044,65939	452,9056814	895,3889731	883,2996058	1040,156289	913,2791575	695,5196145	920,8108008
ABCA1	280,1374721	0,042138327	0,312880977	0,134678455	0,892866108	0,948408086	277,656635	251,0073559	271,7003781	251,8223191	196,867899	79,83033969	304,4924847	334,6983749	425,9520212	252,9367996	361,6790479	280,2725429	452,5451803	187,1100423	222,6176774	192,7118188	300,3394604	291,329986	501,3566099	277,0485947	316,4736888	194,4193841	478,877924	197,2570663	173,8799036	208,69074
PALMD	35,59247251	-0,043077348	0,317324024	-0,135751928	0,892017395	0,948408086	35,7804942	45,39494735	23,55783625	81,14274727	28,19722512	31,65685884	23,75916719	25,78823544	36,36175791	34,22317506	35,77045529	40,60022817	23,26517511	20,86478889	23,86406955	41,55510918	29,44504514	44,07575434	27,9641137	17,68395285	46,31322275	36,45363452	62,09888291	39,31101677	42,58283354	27,69355792
RPS6KB1	297,8827675	-0,022228202	0,165482197	-0,134323827	0,893146512	0,948408086	281,9502943	255,0127925	358,079111	293,7927056	315,2962445	225,7271674	274,4809051	267,7586999	290,8940633	306,4350963	333,8575827	295,9887602	329,4648991	232,2049086	309,8919889	497,6224324	270,8944153	306,3802435	278,6424186	311,4340586	308,7548183	220,5912243	269,8912988	248,5017846	372,5997935	288,8042469
FOXO1	326,9279231	-0,024316238	0,194003599	-0,125339106	0,900255094	0,951798674	300,5561513	304,4131764	414,617918	296,5907314	371,6906948	229,8563229	310,7448972	323,1759718	269,2501598	298,1743299	467,0031663	301,2274993	242,4081148	316,3371219	314,6648028	439,9647184	347,4515326	258,0044156	302,6116589	276,0661529	429,6837888	217,7870986	425,1385061	331,3357128	454,2168911	257,1544664
IL1RAP	47,71067376	-0,04050324	0,321321467	-0,126052082	0,899690698	0,951798674	60,11123026	29,37320123	86,37873293	93,73386322	40,50146881	61,93733252	36,88923326	35,11589507	26,83844036	29,1093673	47,69394039	22,26464125	40,52643406	31,63371219	38,52342656	61,81322491	58,89009028	49,45084633	43,94360724	30,45569658	48,88617957	49,53955461	37,02048789	47,03282364	92,26280601	40,55128124
PPM1F	161,6959511	-0,024578349	0,197250012	-0,124605056	0,900836225	0,951798674	147,4156361	169,5634798	212,0205263	278,4035639	117,9156687	161,0370646	148,1821743	182,1637057	142,8497632	139,6462891	168,9160389	144,0653258	182,3689532	127,2079065	129,2068908	107,0044061	161,9477483	222,5288085	138,8218501	115,9281354	172,3881069	166,3781268	170,771928	150,2242427	180,9770426	166,1613475
TNFSF4	52,46107809	-0,056360096	0,470396291	-0,119814074	0,904630433	0,954219809	44,36781281	36,04892878	54,96828459	75,54669573	27,17187148	44,04432535	26,8853734	25,23954958	40,69053861	31,07621644	45,70669287	47,14865207	36,02349694	37,69123155	27,61413762	28,56913756	85,3906309	51,60088312	32,95770543	40,28011483	59,17800685	39,25776026	40,60311575	52,64868318	301,6284043	31,64978048
IL6	43,22451989	-0,086380578	0,744654296	-0,116000913	0,907651818	0,954572497	137,3970977	13,3514551	17,27574659	156,689443	8,202829126	19,26939234	12,50482484	31,8237799	14,71785439	9,047506051	57,63017797	10,47747824	58,53818252	7,403634768	7,159220865	57,13827512	38,27855868	121,477079	9,987183463	29,47325476	79,7616614	83,1890634	22,68997645	7,721806866	35,48569462	77,14633991
KLRK1	44,75092051	0,048746247	0,421688429	0,115597783	0,90797132	0,954572497	26,47756571	38,7192198	37,692538	78,3447215	23,58313374	22,02216267	31,88730333	19,20400511	36,36175791	15,73479313	57,63017797	47,14865207	32,27104934	20,19173119	17,04576396	42,59398691	76,55711736	45,15077273	32,95770543	38,31523118	97,77235914	43,93130314	65,68151077	33,69515723	124,1999312	58,35428275
CCL5	57,78301088	-0,050335648	0,453733713	-0,110936539	0,91166667	0,95616383	59,39562038	29,37320123	34,55149317	67,15261843	35,37470061	23,39854784	37,51447451	44,99224055	27,7041965	20,45523107	37,75770281	60,24549987	93,81118994	22,2109043	27,27322234	45,7106201	129,5581986	189,2032381	47,93848062	44,20988213	48,88617957	70,10314331	125,3919751	25,97335037	35,48569462	118,6866768
SFTPD	35,89754773	-0,059294029	0,53957553	-0,109890137	0,912496505	0,95616383	26,47756571	37,38407429	26,69888109	50,36446382	15,89298143	33,03324401	17,50675477	26,3369213	25,97268422	16,91490262	33,78320777	35,36148905	20,26321703	20,86478889	11,25020422	18,69979913	58,89009028	22,57538637	16,97821189	16,70151103	69,46983412	27,10654875	57,32204577	23,86740304	205,8170288	17,80300152
HSPA12B	137,1877497	0,025551892	0,246332536	0,103729261	0,917384205	0,959701763	88,02001574	110,8170774	179,0395555	96,53188899	91,76915085	148,649598	112,5434235	135,5254075	102,1592246	153,414233	172,8905339	138,8265866	114,8248965	94,90113657	159,2074354	116,3543057	161,9477483	176,3030173	92,88080621	128,6998791	162,0962796	108,426195	237,6476481	141,8004534	237,7541539	103,8508422
MIF	5819,189245	-0,015303196	0,152287756	-0,100488686	0,919956364	0,96080969	4779,558416	6775,863465	5270,673231	4043,147234	6551,497088	6435,977041	6830,135325	6123,334202	5348,641437	5904,874493	7579,362027	6933,471224	4764,10747	6320,011861	4568,264742	4398,088868	6984,364707	5563,220212	5957,354936	4990,804472	5943,530253	5769,02134	6523,965334	5036,724024	6873,579048	5029,347929
CARD16	27,43807901	-0,037724223	0,419663773	-0,089891541	0,928373403	0,96251583	21,46829652	14,68660061	23,55783625	54,56150247	11,27889005	24,77493301	21,25820222	12,61977479	25,10692808	12,98120433	25,83421771	40,60022817	22,51468559	14,80726954	12,61386533	20,77755459	73,61261284	53,75091992	14,9807752	22,59616198	38,59435229	35,51892595	22,68997645	20,35749083	46,131403	25,71544664
CH25H	39,21038898	0,053344319	0,593886802	0,089822369	0,928428372	0,96251583	20,03707675	41,38951082	23,55783625	51,7634767	10,25353641	33,03324401	10,62910111	20,30137684	46,7508316	12,98120433	43,71944536	40,60022817	30,01958078	22,88396201	5,454644468	8,830460701	79,50162187	30,10051516	7,989746771	37,33278936	92,6264455	46,73542888	56,12783648	34,39713968	173,8799036	38,57316996
FCGR2B	68,71276174	-0,041477621	0,451273401	-0,091912398	0,926767641	0,96251583	45,79903258	62,75183899	56,53880701	121,7141209	33,83667015	30,28047368	49,3940581	20,8500627	45,88507546	38,943613	11,9234851	49,76802163	115,575386	51,15238567	60,34200443	61,29378604	111,8911715	215,0036797	39,94873385	33,40302206	66,8968773	78,51552051	95,53674295	37,20506944	95,81137547	156,2707911
HSP90AA1	4464,895984	-0,023195165	0,254345519	-0,091195493	0,927337256	0,96251583	5495,88391	4116,253608	5358,622486	5004,269086	5181,624624	8938,245275	4689,934554	4320,352465	3745,261065	3518,299744	2829,840463	3999,777317	3706,667737	3957,579312	4610,197322	2481,878896	3707,131183	3810,940222	3420,610336	5394,588062	4415,193902	6225,159126	1970,445323	4802,963871	6039,665224	4345,910482
IL16	68,01059027	-0,028753596	0,323471599	-0,088890636	0,929168825	0,96251583	56,53318084	49,40038388	58,10932942	68,55163131	48,70429794	44,04432535	55,64647052	50,47909916	41,55629476	31,46958626	49,6811879	64,1745542	83,30433667	39,71040466	38,18251128	95,05731225	70,66810833	136,5273366	57,92566409	57,96406769	64,32392048	55,14780607	160,0240444	48,43678852	134,8456396	107,8070648
AIRE	25,30090959	-0,048338495	0,574602825	-0,084125056	0,932957003	0,964865956	17,17463722	26,70291021	20,41679142	48,96545094	7,177475486	20,64577751	12,50482484	13,16846065	17,31512281	10,62098536	39,74495032	27,50338037	23,26517511	10,09586559	9,20471254	14,02484935	58,89009028	10,75018398	8,988465117	12,77174373	30,87548183	13,08592009	34,63206932	23,1654206	131,2970701	14,8358346
IER5	73,58390778	-0,020818623	0,267559409	-0,077809346	0,937979712	0,96749847	67,2673291	74,76814858	80,09664326	55,96051536	51,78035886	56,43179185	66,90081287	69,68310427	51,0796123	53,10492682	109,2986134	55,00676075	77,30042051	40,38346237	66,47847946	119,9903778	79,50162187	119,3270422	51,93335401	65,82360229	95,19940232	57,95193181	131,3630215	66,68833202	81,61709762	68,24483915
MICA	261,5464583	-0,020329872	0,277492159	-0,073262871	0,941596937	0,96749847	195,3614984	245,6667739	191,6037349	254,6203449	317,8596286	254,6312559	233,8402244	270,5021292	207,7814738	249,3964711	427,258216	421,7184991	261,1703528	168,2644265	357,2792127	258,1611158	303,2839649	266,6045628	128,8346667	274,1012692	290,7441206	173,8557954	486,0431797	167,7738037	230,657015	163,1941806
NCAM1	67,65663021	0,031580372	0,419082126	0,075356046	0,93993143	0,96749847	60,82684015	69,42756654	58,10932942	111,9210307	29,73525558	42,66794018	71,27750156	24,14217786	70,99200354	48,77785871	55,64293045	51,07770641	72,7974834	39,03734696	60,00108915	38,43847599	141,3362167	98,90169265	43,94360724	28,49081293	66,8968773	36,45363452	106,2846265	42,82092898	212,9141677	76,15728427
TRAF6	249,5088533	-0,009880687	0,134351845	-0,073543367	0,941373736	0,96749847	249,7478495	208,2826996	314,1044834	281,2015897	255,3130566	251,8784856	211,956781	227,1559462	234,6199141	225,7942815	333,8575827	238,3626299	251,413989	213,3592929	250,5727303	332,9603123	238,5048656	269,829618	240,6911215	264,276851	254,7227251	218,7218071	246,0071131	228,8462762	241,3027234	203,7454618
KLRB1	27,70806244	-0,032805631	0,466324289	-0,070349393	0,94391557	0,968314033	16,45902733	25,3677647	26,69888109	19,58618037	14,35495097	13,76385167	32,51254457	17,55794753	15,58361053	9,047506051	13,91073261	15,71621736	63,04111964	10,09586559	12,27295005	39,99679259	53,00108125	61,27604871	28,96283204	25,54348745	18,01069774	19,62888013	74,04097578	14,74163129	24,83998623	54,39806019
IRS1	255,0177254	0,013538272	0,199427495	0,067885685	0,945876634	0,968760746	201,8019873	280,3805572	259,1361988	246,2262676	252,2369956	214,7160861	247,5955317	249,6520665	282,2365019	377,6350352	288,1508898	288,1306515	280,6830803	191,1483886	267,9594095	209,3338625	306,2284694	316,0554091	170,7808372	174,8746449	313,900732	233,6771444	194,6561138	176,8995755	386,7940713	219,5703521
IFI44	146,3411118	0,016701055	0,261931052	0,063761265	0,949160322	0,968998078	129,525389	162,8877523	138,2059727	124,5121467	124,0677905	105,9816579	153,8093455	171,7386743	128,997665	168,7556563	143,0818212	138,8265866	165,1076943	100,2855982	148,6390618	204,1394738	135,4472076	234,3540109	112,8551731	83,50755514	102,9182728	119,6426979	291,387066	104,5953839	102,9085144	208,69074
IL23R	31,16758725	0,038750225	0,594809008	0,06514734	0,948056688	0,968998078	20,75268664	30,70834674	26,69888109	60,15755401	12,81692051	22,02216267	19,38247849	13,16846065	24,24117194	9,047506051	45,70669287	28,81306515	21,76419607	11,441981	9,54562782	17,14148254	82,44612639	23,65040477	13,98205685	11,7893019	43,74026593	13,08592009	52,54520862	23,1654206	159,6856258	12,85772332
ENG	1509,682137	0,011433536	0,211287262	0,0541137	0,956844577	0,970601128	1481,31246	1400,56764	1817,094436	1821,514775	1331,421703	1193,32594	1327,387156	1538,515153	1678,701157	2094,300966	1033,368708	1330,639736	1826,691491	1398,613913	1205,135512	1703,240038	1687,201086	1983,408945	1154,518408	803,637413	1255,602928	1530,117941	2092,25467	1359,038008	1114,250811	2089,874567
ERG	104,2330564	-0,012815487	0,216358045	-0,059232772	0,952766708	0,970601128	98,754164	100,1359133	130,3533606	106,3249792	102,0226873	107,358043	101,9143224	93,27659627	107,3537615	123,5181261	111,2858609	99,53604326	114,8248965	63,26742438	96,13810876	159,9871703	73,61261284	123,6271158	99,87183463	55,99918404	79,7616614	74,7766862	154,052998	105,9993488	113,5542228	112,7523429
INHBC	36,52249214	0,033302664	0,591616763	0,056290941	0,955110035	0,970601128	21,46829652	28,03805572	32,98097075	43,3693994	15,89298143	33,03324401	14,38054856	20,8500627	21,64390352	11,01435519	43,71944536	31,43243471	21,76419607	14,13421183	8,181966703	11,42765502	100,1131535	35,47560715	9,987183463	28,49081293	51,45913639	25,23713159	53,73941791	35,09912212	212,9141677	23,73733536
TFF3	31,58776353	-0,019071245	0,351104156	-0,054317914	0,956681877	0,970601128	35,06488432	44,05980184	32,98097075	23,78321903	14,86762779	22,02216267	25,63489091	35,11589507	43,28780704	28,71599747	57,63017797	17,02590214	26,26713318	25,57619283	35,79610432	10,3887773	50,05657673	38,70066234	19,97436693	22,59616198	41,16730911	22,43300586	48,96258076	18,2515435	53,22854193	27,69355792
PTPN7	33,04810517	-0,017236753	0,358726211	-0,048049885	0,961676486	0,97394668	17,8902471	29,37320123	34,55149317	26,58124479	20,50707282	28,90408851	29,38633836	22,49612028	22,50965966	12,58783451	27,82146523	28,81306515	37,52447598	15,48032724	19,09125564	28,0496987	32,38954965	50,52586473	20,97308527	23,5786038	74,61574776	35,51892595	75,23518507	40,01299921	60,32568085	44,5075038
MERTK	316,773405	-0,010889179	0,248288368	-0,043856984	0,965018404	0,975774979	281,9502943	284,3859937	383,2074697	212,6499584	471,6626748	220,2216267	336,3797881	289,1574484	231,1568896	367,0140498	280,2018998	301,2274993	333,2173467	249,0313513	581,9423817	359,4516944	161,9477483	279,5047836	408,4758037	300,6271985	352,4950843	235,5465615	450,2169011	379,0705189	251,9484318	233,417131
CD6	26,55093077	-0,018418768	0,559932105	-0,032894645	0,973758603	0,981517041	10,73414826	20,02718265	18,846269	39,17236075	8,202829126	11,01108134	18,75723725	9,327659627	15,58361053	7,474026738	9,936237581	27,50338037	35,27300742	9,422807886	8,522881982	20,77755459	53,00108125	46,22579113	12,9833385	11,7893019	46,31322275	24,30242302	85,98306865	10,52973664	102,9085144	25,71544664
CTSL	1099,815301	0,008626601	0,262549225	0,032857079	0,97378856	0,981517041	1298,83194	1073,45699	1155,904499	1578,086533	1844,6112	1156,16354	878,4639447	847,1709685	510,796123	818,9959825	1494,410132	970,4764218	650,6744134	1314,4817	760,9229033	2051,264077	945,1859489	855,7146451	958,7696125	995,2135689	1196,424921	1185,210476	1012,689475	960,3119811	1110,702242	970,2635828
CSF2RB	35,38227108	-0,010420777	0,469637813	-0,022188964	0,982297221	0,986960024	22,89951629	24,03261919	34,55149317	40,57137363	12,81692051	27,52770334	15,63103104	17,55794753	31,16722107	16,91490262	19,87247516	26,19369559	48,78181877	18,84561577	8,863797261	21,81643232	64,7790993	54,82593832	23,96924031	21,61372015	48,88617957	35,51892595	50,15679005	25,97335037	159,6856258	46,48561508
ROBO4	209,9514758	0,005058214	0,220321004	0,022958381	0,981683471	0,986960024	171,7463722	205,6124086	230,8667953	188,8667393	185,589009	192,6939234	191,9490612	273,7942444	182,6745457	176,2296831	224,5589693	213,4786191	258,1683947	141,3421183	215,4584565	317,3771464	238,5048656	262,3044892	149,807752	182,7341795	146,6585387	162,6392925	345,1264839	275,1771174	180,9770426	144,4021234
ICAM2	136,3344826	0,005255733	0,268208066	0,01959573	0,98436587	0,987476031	110,919532	106,8116408	171,1869434	159,4874688	73,82546214	119,7455095	93,78618626	88,88710939	135,057958	112,1104011	139,1073261	106,0844672	118,5773441	108,3622907	112,5020422	178,6869695	179,6147753	169,8529069	108,8602998	78,59534601	149,2314955	117,7732808	212,5692531	183,2174175	298,0798348	111,7632873
TBC1D10B	199,9282286	-0,002825819	0,185218806	-0,015256653	0,987827425	0,989385512	207,5268664	202,9421176	190,0332124	244,8272547	141,4988024	198,1994641	179,4442364	195,3321663	242,4117194	177,0164227	238,4697019	197,7624017	226,6478349	154,1302147	153,752791	144,4040044	320,950992	181,6781093	179,7693023	155,2258084	200,6906319	174,790504	197,0445323	170,5817335	294,5312653	228,4718528
PPP3CA	330,5970821	0,000964147	0,151031463	0,00638375	0,994906539	0,994906539	352,080063	285,7211392	403,6242611	397,319659	366,0512498	240,8674042	371,3932976	285,3166474	387,858751	321,7765196	298,0871274	295,9887602	345,225179	331,8174491	298,3008694	278,9386704	306,2284694	364,4312371	378,5142533	258,3822	388,5164797	270,1307789	303,3291589	254,8196266	415,182627	395,622256
ADAMDEC1	14,82555204	-0,069735429	0,513378043	-0,135836408	0,891950608	NA	11,44975815	17,35689163	14,13470175	20,98519326	9,740859588	15,14023684	10,00385987	4,389486883	10,38907369	7,474026738	19,87247516	10,47747824	13,50881135	12,78809642	6,477390306	7,791582971	32,38954965	9,675165586	1,997436693	10,80686008	38,59435229	11,21650293	16,71893002	11,93370152	53,22854193	6,923389479
AGR3	22,74932588	0,309946994	0,453408934	0,683592606	0,494232455	NA	12,16536803	9,346018572	18,846269	26,58124479	11,27889005	17,89300717	10,00385987	17,55794753	12,98634211	12,98120433	9,936237581	17,02590214	22,51468559	10,7689233	9,20471254	35,3218428	38,27855868	38,70066234	20,97308527	20,63127833	36,02139547	20,56358871	21,49576716	16,14559617	106,4570839	17,80300152
AICDA	10,43965692	0,89727136	0,614696404	1,459698404	0,144372981	NA	2,146829652	8,010873062	12,56417933	46,16742517	6,152121845	12,3874665	4,376688692	9,327659627	6,926049126	6,687287081	11,9234851	3,929054339	6,754405676	5,384461649	4,77281391	3,636072053	11,77801806	9,675165586	1,997436693	5,894650951	23,15661137	9,347085775	13,13630215	7,019824424	28,38855569	9,890556399
ALOX15	9,764295601	0,601975677	0,5613139	1,072440353	0,283522291	NA	5,724879073	8,010873062	6,282089667	23,78321903	9,228182767	9,63469617	6,252412418	6,584230325	7,791805267	3,146958626	13,91073261	5,238739119	8,255384715	2,692230825	5,795559748	7,272144107	17,66702708	8,600147187	1,997436693	6,877092776	25,72956819	11,21650293	9,553674295	7,721806866	31,93712516	2,96716692
ANKRD1	21,19440292	0,601906062	0,518264601	1,161387564	0,245484324	NA	12,16536803	14,68660061	10,99365692	61,55656689	7,177475486	19,26939234	10,00385987	10,97371721	16,44936667	12,58783451	23,84697019	15,71621736	25,51664366	13,46115412	10,22745838	18,69979913	26,50054062	18,27531277	15,97949354	12,77174373	64,32392048	15,89004582	35,8262786	15,44361373	56,77711139	5,934333839
ANKRD22	11,38589025	0,753906695	0,593857229	1,269508323	0,204259824	NA	4,293659304	10,68116408	4,71156725	50,36446382	3,588737743	6,881925836	5,627171176	9,327659627	6,926049126	4,72043794	23,84697019	9,167793458	6,754405676	4,711403943	8,522881982	18,18036027	11,77801806	7,525128789	6,991028424	6,877092776	20,58365456	8,412377198	7,165255721	9,125771751	28,38855569	10,87961204
AREG	14,33775382	0,379067825	0,475134754	0,797811192	0,424980065	NA	7,156098841	13,3514551	10,99365692	13,99012884	7,690152306	8,258311003	10,00385987	10,97371721	8,657561407	8,260766395	21,85972268	6,548423898	12,00783231	7,403634768	9,20471254	33,24408734	8,833513541	4,300073594	15,97949354	9,824418252	30,87548183	13,08592009	28,66102288	11,23171908	42,58283354	17,80300152
ARG1	25,05213826	-0,071096925	0,567636932	-0,125250704	0,900325077	NA	15,74341745	28,03805572	12,56417933	19,58618037	12,81692051	30,28047368	9,378618626	14,26583237	17,31512281	7,867396566	29,80871274	27,50338037	11,25734279	12,11503871	6,477390306	18,69979913	38,27855868	23,65040477	16,97821189	11,7893019	46,31322275	13,08592009	29,85523217	23,1654206	159,6856258	14,8358346
BATF	15,63136973	0,875751539	0,443991893	1,972449393	0,048558328	NA	6,440488957	9,346018572	9,423134501	30,77828345	15,89298143	9,63469617	16,25627229	12,61977479	7,791805267	11,01435519	17,88522765	9,167793458	20,26321703	8,076692474	13,29569589	15,06372708	35,33405417	25,80044156	4,993591732	8,841976427	18,01069774	20,56358871	33,43786003	10,52973664	14,19427785	21,75922408
BCL2A1	17,2308957	0,848461682	0,561219275	1,511818499	0,130580042	NA	11,44975815	13,3514551	7,852612084	57,35952824	8,715505947	9,63469617	15,0057898	18,10663339	11,25482983	5,507177596	37,75770281	9,167793458	15,76027991	7,403634768	5,454644468	29,60801529	17,66702708	10,75018398	8,988465117	9,824418252	43,74026593	23,36771444	14,33051144	7,019824424	39,03426408	9,890556399
BDNF	15,3183528	1,016839067	0,500324901	2,032357506	0,042117477	NA	7,871708725	5,340582041	12,56417933	46,16742517	6,152121845	12,3874665	25,00964967	12,07108893	12,98634211	17,30827245	11,9234851	7,858108678	14,25930087	8,076692474	8,181966703	12,98597162	23,55603611	12,90022078	6,991028424	10,80686008	41,16730911	13,08592009	19,10734859	12,63568396	31,93712516	4,9452782
BK VP1	14,1313507	0,4404455	0,598890645	0,735435598	0,462074248	NA	6,440488957	12,01630959	9,423134501	19,58618037	6,664798665	17,89300717	5,627171176	9,327659627	15,58361053	2,753588798	17,88522765	9,167793458	9,005874235	6,057519356	3,068237513	6,233266377	38,27855868	12,90022078	3,994873385	9,824418252	43,74026593	14,02062866	25,07839502	13,3376664	42,58283354	6,923389479
BK large T Ag	6,007512845	-0,090293648	0,591990199	-0,15252558	0,878772417	NA	3,57804942	2,670291021	7,852612084	5,596051536	5,126768204	1,376385167	4,376688692	2,194743442	8,657561407	3,540328455	3,974495032	6,548423898	4,502937117	1,346115412	3,750068072	4,674949783	17,66702708	4,300073594	0,998718346	6,877092776	10,29182728	4,673542888	4,776837147	3,509912212	28,38855569	4,9452782
BLK	15,22131477	0,045544809	0,465073813	0,09793028	0,921987656	NA	6,440488957	12,01630959	14,13470175	9,793090187	9,228182767	8,258311003	9,378618626	7,681602046	15,58361053	8,260766395	21,85972268	17,02590214	10,50685327	7,403634768	8,181966703	39,99679259	29,44504514	13,97523918	9,987183463	7,859534601	30,87548183	11,21650293	37,02048789	10,52973664	21,29141677	17,80300152
BTLA	19,27741219	0,38642831	0,504949006	0,76528185	0,444103729	NA	8,587318609	17,35689163	12,56417933	43,3693994	10,25353641	9,63469617	13,13006608	6,584230325	18,18087896	5,900547425	15,89798013	13,0968478	26,26713318	6,730577062	9,20471254	37,39959826	32,38954965	23,65040477	10,98590181	15,7190692	28,30252501	14,02062866	42,99153433	9,125771751	46,131403	23,73733536
CCL22	14,35921257	0,202339235	0,525239961	0,38523199	0,700065544	NA	7,871708725	17,35689163	6,282089667	37,77334786	9,740859588	12,3874665	11,87958359	9,327659627	12,98634211	5,900547425	23,84697019	17,02590214	9,005874235	6,730577062	9,20471254	13,50541048	23,55603611	4,300073594	2,996155039	9,824418252	25,72956819	10,28179435	19,10734859	9,125771751	49,67997247	7,912445119
CCL4	7,979270895	1,141406638	0,708060756	1,612017936	0,106958037	NA	1,431219768	5,340582041	1,570522417	37,77334786	3,076060922	8,258311003	3,126206209	5,486858604	7,791805267	2,753588798	1,987247516	6,548423898	4,502937117	5,384461649	3,409152793	5,194388648	20,6115316	7,525128789	2,996155039	5,894650951	15,43774092	6,542960043	15,52472073	5,615859539	17,74284731	5,934333839
CCR3	19,76391966	0,208476385	0,570789607	0,365242083	0,714930707	NA	12,16536803	16,02174612	15,70522417	55,96051536	7,690152306	13,76385167	10,62910111	9,876345488	11,25482983	8,654136223	15,89798013	17,02590214	8,255384715	12,11503871	5,113729189	16,10260481	47,11207222	10,75018398	7,989746771	13,75418555	66,8968773	23,36771444	27,4668136	12,63568396	56,77711139	10,87961204
CCR4	12,8942416	0,580711996	0,549643065	1,05652565	0,290728129	NA	3,57804942	10,68116408	9,423134501	27,98025768	4,101414563	9,63469617	10,00385987	6,584230325	5,194536844	4,72043794	13,91073261	6,548423898	8,255384715	5,384461649	4,090983351	9,86933843	20,6115316	16,12527598	8,988465117	9,824418252	28,30252501	16,8247544	32,24365074	11,23171908	28,38855569	22,74827972
CCR7	25,15758245	-0,197232334	0,602155071	-0,327544088	0,743256403	NA	14,31219768	14,68660061	31,41044834	34,9753221	6,664798665	16,51662201	11,25434235	6,584230325	19,91239124	7,474026738	31,79596026	28,81306515	14,25930087	8,74975018	7,500136144	13,50541048	26,50054062	35,47560715	11,98462016	15,7190692	46,31322275	13,08592009	38,21469718	23,86740304	159,6856258	14,8358346
CD160	18,62973047	0,539593584	0,601289175	0,897394475	0,36950846	NA	7,871708725	13,3514551	9,423134501	57,35952824	8,715505947	11,01108134	6,877653659	11,52240307	12,12058597	7,08065691	19,87247516	17,02590214	14,25930087	10,09586559	6,136475027	13,50541048	29,44504514	22,57538637	6,991028424	5,894650951	46,31322275	11,21650293	19,10734859	14,03964885	88,71423655	13,84677896
CD19	9,935825874	0,609133291	0,569781101	1,069065453	0,285040177	NA	6,440488957	8,010873062	4,71156725	30,77828345	3,076060922	6,881925836	6,877653659	6,584230325	9,523317548	3,540328455	15,89798013	6,548423898	8,255384715	3,365288531	6,477390306	19,73867686	11,77801806	8,600147187	3,994873385	6,877092776	18,01069774	3,73883431	20,30155788	7,721806866	17,74284731	12,85772332
CD207	15,88167557	-0,178170237	0,389631375	-0,457278979	0,647470537	NA	15,02780757	14,68660061	9,423134501	15,38914172	10,25353641	30,28047368	11,87958359	12,61977479	15,58361053	9,047506051	23,84697019	26,19369559	13,50881135	12,78809642	13,29569589	11,42765502	23,55603611	3,225055195	8,988465117	9,824418252	33,44843865	16,8247544	11,94209287	10,52973664	35,48569462	13,84677896
CD244	20,10335395	0,40148831	0,55713665	0,720628073	0,471138377	NA	12,88097791	13,3514551	14,13470175	39,17236075	7,690152306	11,01108134	13,13006608	8,778973767	18,18087896	6,687287081	7,948990065	7,858108678	20,26321703	11,441981	8,863797261	12,98597162	53,00108125	21,50036797	8,988465117	10,80686008	61,75096367	17,75946297	26,27260431	14,74163129	81,61709762	11,86866768
CD27	17,33409198	0,278889946	0,572714178	0,486961832	0,626285379	NA	10,01853838	10,68116408	14,13470175	34,9753221	11,79156687	9,63469617	7,502894901	5,486858604	8,657561407	4,72043794	13,91073261	10,47747824	22,51468559	5,384461649	6,818305585	28,0496987	44,16756771	20,42534957	2,996155039	5,894650951	20,58365456	15,89004582	38,21469718	14,03964885	46,131403	37,58411432
CD3D	24,12158047	0,348199779	0,511185054	0,681161894	0,495769057	NA	20,03707675	21,36232817	10,99365692	68,55163131	14,35495097	17,89300717	12,50482484	8,230287906	17,31512281	8,260766395	21,85972268	17,02590214	24,01566463	12,78809642	11,25020422	24,93306551	32,38954965	50,52586473	12,9833385	14,73662738	20,58365456	31,78009164	59,71046434	9,125771751	35,48569462	48,46372636
CD3G	17,18707366	0,433750675	0,525788666	0,824952501	0,409398558	NA	5,724879073	10,68116408	12,56417933	41,97038652	4,614091384	9,63469617	7,502894901	8,778973767	10,38907369	5,900547425	21,85972268	15,71621736	16,51076943	6,057519356	7,841051423	14,54428821	35,33405417	31,17553355	8,988465117	12,77174373	23,15661137	19,62888013	39,40890646	13,3376664	39,03426408	23,73733536
CD40LG	16,27785109	0,89803966	0,566567744	1,585052572	0,11295439	NA	8,587318609	5,340582041	4,71156725	23,78321903	11,79156687	20,64577751	7,502894901	7,132916185	12,12058597	4,327068111	17,88522765	11,78716302	27,0176227	4,711403943	4,431898631	12,98597162	47,11207222	29,02549676	14,9807752	3,929767301	28,30252501	16,8247544	28,66102288	8,423789308	35,48569462	25,71544664
CD45RA	23,23495934	0,207486869	0,5159629	0,402135249	0,687584492	NA	13,5965878	20,02718265	6,282089667	5,596051536	16,91833507	15,14023684	15,0057898	13,71714651	21,64390352	9,834245708	19,87247516	22,26464125	21,76419607	6,730577062	7,841051423	36,36072053	35,33405417	29,02549676	8,988465117	13,75418555	36,02139547	20,56358871	68,06992935	14,03964885	85,16566708	40,55128124
CD7	16,5973702	0,717462934	0,523273676	1,37110458	0,170342358	NA	5,724879073	13,3514551	7,852612084	18,18716749	18,45636553	8,258311003	8,753377385	12,61977479	7,791805267	7,474026738	25,83421771	9,167793458	25,51664366	8,74975018	4,431898631	17,14148254	14,72252257	20,42534957	6,991028424	24,56104563	18,01069774	20,56358871	41,79732504	7,019824424	60,32568085	17,80300152
CD79A	21,62188405	0,3808027	0,484869942	0,785370813	0,432236189	NA	14,31219768	10,68116408	12,56417933	40,57137363	9,228182767	17,89300717	10,00385987	14,26583237	14,71785439	9,834245708	21,85972268	19,6452717	23,26517511	7,403634768	11,5911195	31,16633189	29,44504514	27,95047836	7,989746771	14,73662738	59,17800685	17,75946297	25,07839502	16,14559617	78,06852816	16,81394588
CD80	8,662583276	2,447727773	1,063311196	2,301986268	0,021335947	NA	0,715609884	1,33514551	1,570522417	36,37433498	2,050707282	1,376385167	1,875723725	0,54868586	6,926049126	0,393369828	11,9234851	2,619369559	7,504895195	0,673057706	0,340915279	15,06372708	26,50054062	23,65040477	2,996155039	1,96488365	10,29182728	6,542960043	10,74788358	8,423789308	31,93712516	10,87961204
CD86	21,93760749	0,491497113	0,427598854	1,149435057	0,250376631	NA	17,8902471	21,36232817	9,423134501	39,17236075	18,96904235	9,63469617	25,63489091	20,30137684	15,58361053	13,76794399	13,91073261	10,47747824	25,51664366	12,11503871	12,61386533	18,69979913	38,27855868	53,75091992	15,97949354	12,77174373	43,74026593	19,62888013	35,8262786	9,827754193	24,83998623	30,66072484
CEACAM3	14,22146763	0,149204928	0,494999903	0,301424155	0,763091077	NA	7,871708725	6,675727552	12,56417933	33,57630921	9,740859588	16,51662201	12,50482484	7,681602046	8,657561407	8,260766395	15,89798013	20,95495648	12,75832183	7,403634768	5,795559748	7,791582971	17,66702708	17,20029437	2,996155039	15,7190692	23,15661137	12,15121151	10,74788358	13,3376664	53,22854193	8,901500759
CLEC4C	10,89734923	-0,149962792	0,427169502	-0,351061559	0,725542166	NA	8,587318609	10,68116408	10,99365692	11,19210307	8,202829126	16,51662201	10,00385987	5,486858604	10,38907369	3,540328455	3,974495032	10,47747824	13,50881135	6,057519356	5,795559748	10,3887773	23,55603611	13,97523918	4,993591732	9,824418252	25,72956819	12,15121151	11,94209287	9,125771751	21,29141677	4,9452782
CMV UL83	10,2430021	0,193141603	0,490063235	0,394115676	0,693495638	NA	8,587318609	9,346018572	3,141044834	13,99012884	7,177475486	13,76385167	5,627171176	6,035544465	4,328780704	4,72043794	9,936237581	7,858108678	7,504895195	8,74975018	4,77281391	8,311021836	26,50054062	12,90022078	5,992310078	14,73662738	15,43774092	7,47766862	17,9131393	5,615859539	31,93712516	3,95622256
CRP	21,35651741	-0,153376161	0,658472673	-0,232927147	0,815817978	NA	10,73414826	18,69203714	18,846269	15,38914172	21,53242646	17,89300717	6,252412418	7,132916185	15,58361053	4,327068111	23,84697019	24,88401081	7,504895195	9,422807886	2,045491676	9,86933843	35,33405417	17,20029437	13,98205685	9,824418252	33,44843865	8,412377198	37,02048789	19,65550839	152,5884869	13,84677896
CSF2	13,0809978	0,61391502	0,683282386	0,898479212	0,368930123	NA	6,440488957	4,005436531	12,56417933	15,38914172	5,126768204	6,881925836	8,753377385	7,132916185	11,25482983	3,146958626	9,936237581	2,619369559	5,253426637	8,74975018	3,068237513	6,752705242	53,00108125	7,525128789	4,993591732	5,894650951	28,30252501	17,75946297	19,10734859	10,52973664	70,97138924	4,9452782
CSF3	18,89187727	-0,24708245	0,560267415	-0,441008067	0,659207157	NA	14,31219768	20,02718265	15,70522417	32,17729633	8,202829126	9,63469617	11,25434235	12,61977479	18,18087896	6,293917253	7,948990065	20,95495648	16,51076943	8,076692474	5,454644468	10,90821616	32,38954965	20,42534957	6,991028424	10,80686008	43,74026593	26,17184017	25,07839502	9,125771751	92,26280601	5,934333839
CTLA4	14,40245883	0,867072845	0,589330892	1,471283548	0,141214451	NA	7,156098841	12,01630959	6,282089667	47,56643805	6,664798665	11,01108134	11,25434235	8,230287906	7,791805267	4,327068111	11,9234851	5,238739119	8,255384715	4,038346237	4,090983351	15,58316594	38,27855868	27,95047836	8,988465117	10,80686008	20,58365456	17,75946297	23,88418574	7,019824424	31,93712516	15,82489024
CTSW	23,87774467	0,299723105	0,510003064	0,587688832	0,556741173	NA	18,60585699	13,3514551	15,70522417	46,16742517	11,79156687	9,63469617	13,75530732	9,327659627	16,44936667	8,654136223	23,84697019	15,71621736	30,01958078	9,422807886	10,56837366	17,14148254	44,16756771	63,42608551	17,97693023	11,7893019	25,72956819	23,36771444	64,48730149	11,23171908	39,03426408	49,452782
CXCL10	22,83816318	0,693705884	0,49174782	1,410694376	0,15833475	NA	15,74341745	8,010873062	18,846269	74,14768285	9,740859588	13,76385167	40,01543947	29,0803506	40,69053861	12,58783451	11,9234851	19,6452717	11,25734279	10,09586559	11,93203477	23,37474891	50,05657673	15,05025758	14,9807752	21,61372015	33,44843865	21,49829728	15,52472073	14,74163129	46,131403	9,890556399
CXCL11	18,53553534	0,770062339	0,526322915	1,463098637	0,143440391	NA	10,01853838	14,68660061	9,423134501	75,54669573	6,152121845	17,89300717	8,128136143	25,23954958	14,71785439	7,474026738	23,84697019	13,0968478	17,26125895	8,74975018	7,159220865	15,58316594	50,05657673	15,05025758	11,98462016	17,68395285	33,44843865	14,02062866	15,52472073	14,03964885	21,29141677	13,84677896
CXCL13	17,08224736	0,010902643	0,513655627	0,021225588	0,983065703	NA	7,871708725	14,68660061	17,27574659	22,38420614	9,228182767	23,39854784	6,252412418	12,07108893	8,657561407	6,293917253	17,88522765	17,02590214	12,00783231	10,09586559	10,56837366	6,752705242	35,33405417	15,05025758	4,993591732	12,77174373	48,88617957	13,08592009	23,88418574	11,93370152	63,87425031	11,86866768
CXCL5	9,203432941	0,932920698	0,654149251	1,42615878	0,153822515	NA	4,293659304	4,005436531	6,282089667	40,57137363	3,588737743	11,01108134	3,126206209	10,42503135	8,657561407	2,753588798	13,91073261	6,548423898	3,001958078	4,711403943	3,068237513	7,272144107	14,72252257	8,600147187	3,994873385	2,947325476	12,8647841	7,47766862	21,49576716	7,721806866	21,29141677	4,9452782
CXCR3	17,13676661	-0,139215256	0,538969348	-0,258299023	0,79617614	NA	11,44975815	16,02174612	15,70522417	29,37927056	5,639445024	8,258311003	11,87958359	4,389486883	9,523317548	6,293917253	13,91073261	13,0968478	30,01958078	6,057519356	5,795559748	10,90821616	44,16756771	32,25055195	5,992310078	10,80686008	38,59435229	21,49829728	31,04944146	7,019824424	39,03426408	16,81394588
CXCR5	17,65634139	0,440518842	0,649865621	0,677861434	0,497859554	NA	8,587318609	10,68116408	12,56417933	39,17236075	9,228182767	8,258311003	6,252412418	5,486858604	12,98634211	6,293917253	29,80871274	14,40653258	12,00783231	8,076692474	4,77281391	20,77755459	58,89009028	9,675165586	5,992310078	5,894650951	38,59435229	4,673542888	45,3799529	5,615859539	53,22854193	21,75922408
DUSP2	12,88907594	-0,783384049	0,430560577	-1,819451411	0,068842589	NA	8,587318609	37,38407429	12,56417933	6,995064419	6,152121845	6,881925836	6,252412418	9,876345488	8,657561407	8,260766395	15,89798013	9,167793458	10,50685327	8,74975018	3,750068072	15,06372708	11,77801806	13,97523918	10,98590181	13,75418555	12,8647841	12,15121151	28,66102288	21,05947327	21,29141677	13,84677896
EBI3	18,92465948	0,202794757	0,406454651	0,498935751	0,617824647	NA	8,587318609	12,01630959	15,70522417	22,38420614	13,32959733	11,01108134	23,13392595	26,88560716	6,060292985	8,260766395	21,85972268	24,88401081	12,00783231	14,13421183	13,29569589	9,349899566	20,6115316	23,65040477	15,97949354	16,70151103	46,31322275	20,56358871	32,24365074	21,76145571	35,48569462	15,82489024
EBV LMP2	7,152754459	-0,538125414	0,621707245	-0,865560791	0,386731065	NA	3,57804942	13,3514551	4,71156725	5,596051536	4,101414563	6,881925836	6,252412418	2,194743442	1,731512281	2,753588798	13,91073261	10,47747824	7,504895195	6,057519356	1,704576396	1,558316594	8,833513541	4,300073594	1,997436693	8,841976427	12,8647841	7,47766862	10,74788358	5,615859539	31,93712516	0,98905564
EOMES	16,92591322	-0,287502466	0,552760978	-0,520120772	0,602979402	NA	10,73414826	13,3514551	15,70522417	13,99012884	4,101414563	20,64577751	11,87958359	7,132916185	12,12058597	4,327068111	9,936237581	20,95495648	14,25930087	12,78809642	4,77281391	8,311021836	26,50054062	22,57538637	4,993591732	6,877092776	56,60505003	18,69417155	23,88418574	9,125771751	70,97138924	14,8358346
FASLG	9,608299981	0,519543048	0,584995549	0,888114532	0,374479147	NA	7,871708725	4,005436531	10,99365692	34,9753221	5,639445024	6,881925836	3,751447451	6,584230325	4,328780704	3,540328455	5,961742548	2,619369559	6,754405676	6,057519356	3,409152793	6,752705242	26,50054062	13,97523918	4,993591732	8,841976427	12,8647841	8,412377198	11,94209287	7,019824424	21,29141677	13,84677896
FCAR	15,96212633	0,89397039	0,547423007	1,633052279	0,102458002	NA	7,156098841	9,346018572	9,423134501	53,16248959	2,563384102	17,89300717	18,75723725	10,42503135	9,523317548	8,260766395	21,85972268	10,47747824	15,76027991	9,422807886	5,113729189	24,93306551	8,833513541	21,50036797	7,989746771	9,824418252	28,30252501	13,08592009	29,85523217	9,827754193	31,93712516	19,7811128

FCRL2	11,97718718	0,494727759	0,622082423	0,795276865	0,426452468	NA	7,156098841	8,010873062	1,570522417	20,98519326	8,202829126	2,752770334	10,00385987	7,681602046	12,98634211	3,540328455	9,936237581	13,0968478	17,26125895	5,384461649	2,386406955	32,72464848	11,77801806	15,05025758	1,997436693	4,912209126	15,43774092	14,02062866	21,49576716	5,615859539	42,58283354	14,8358346
FGFBP2	10,41769484	-0,212680135	0,463865617	-0,458495148	0,646596748	NA	5,009269189	6,675727552	14,13470175	15,38914172	5,639445024	11,01108134	7,502894901	5,486858604	6,060292985	7,867396566	9,936237581	15,71621736	15,76027991	3,365288531	3,068237513	7,791582971	26,50054062	9,675165586	6,991028424	9,824418252	20,58365456	9,347085775	5,971046434	13,3376664	21,29141677	6,923389479
FOXP3	13,60566052	0,420362404	0,511162305	0,822365812	0,410868731	NA	9,302928493	9,346018572	14,13470175	36,37433498	5,639445024	17,89300717	7,502894901	5,486858604	13,85209825	5,113807768	13,91073261	6,548423898	14,25930087	7,403634768	6,136475027	7,791582971	23,55603611	12,90022078	5,992310078	13,75418555	33,44843865	13,08592009	15,52472073	8,423789308	35,48569462	10,87961204
GBP5	16,91631835	0,369874494	0,450804811	0,820475925	0,411944848	NA	9,302928493	8,010873062	9,423134501	9,793090187	13,32959733	8,258311003	10,00385987	14,26583237	12,98634211	7,867396566	19,87247516	17,02590214	27,76811222	8,076692474	7,500136144	14,02484935	35,33405417	46,22579113	16,97821189	14,73662738	18,01069774	20,56358871	37,02048789	7,019824424	17,74284731	28,68261356
GZMB	14,25213257	0,51523343	0,467360362	1,10243288	0,270273524	NA	9,302928493	10,68116408	9,423134501	36,37433498	11,27889005	8,258311003	11,25434235	10,97371721	11,25482983	4,72043794	17,88522765	9,167793458	16,51076943	5,384461649	6,136475027	21,29699345	26,50054062	20,42534957	12,9833385	16,70151103	25,72956819	10,28179435	8,359465008	8,423789308	28,38855569	12,85772332
GZMH	14,48060399	0,37081985	0,52738374	0,703130989	0,481974122	NA	10,73414826	12,01630959	10,99365692	43,3693994	10,25353641	16,51662201	3,751447451	9,876345488	9,523317548	4,72043794	13,91073261	9,167793458	13,50881135	9,422807886	4,77281391	11,94709389	14,72252257	31,17553355	11,98462016	7,859534601	30,87548183	9,347085775	16,71893002	8,423789308	39,03426408	11,86866768
HDC	19,82117219	0,453642195	0,554261033	0,818463084	0,413092813	NA	12,88097791	17,35689163	12,56417933	43,3693994	6,664798665	8,258311003	13,13006608	10,42503135	15,58361053	7,08065691	27,82146523	9,167793458	30,7700703	8,74975018	9,886543099	35,3218428	23,55603611	69,8761959	5,992310078	15,7190692	25,72956819	11,21650293	40,60311575	8,423789308	28,38855569	16,81394588
ICOS	10,62580771	0,116751283	0,610185288	0,191337427	0,84826124	NA	9,302928493	4,005436531	10,99365692	6,995064419	4,614091384	8,258311003	5,001929934	1,097371721	8,657561407	3,933698283	13,91073261	2,619369559	9,756363754	6,057519356	2,386406955	9,349899566	23,55603611	18,27531277	5,992310078	8,841976427	38,59435229	10,28179435	20,30155788	5,615859539	31,93712516	5,934333839
IFNA1	15,79633584	0,617481577	0,556251399	1,110076447	0,266966086	NA	7,156098841	9,346018572	9,423134501	51,7634767	8,715505947	17,89300717	8,753377385	9,876345488	11,25482983	7,474026738	11,9234851	14,40653258	11,25734279	6,730577062	8,181966703	15,06372708	35,33405417	11,82520238	6,991028424	5,894650951	48,88617957	10,28179435	22,68997645	12,63568396	39,03426408	7,912445119
IFNG	24,97294631	0,101804044	0,631049949	0,161324858	0,871837546	NA	8,587318609	20,02718265	18,846269	20,98519326	9,740859588	31,65685884	11,87958359	7,681602046	22,50965966	7,08065691	25,83421771	27,50338037	18,01174847	13,46115412	5,113729189	9,349899566	38,27855868	19,35033117	7,989746771	20,63127833	64,32392048	8,412377198	28,66102288	21,76145571	166,7827647	14,8358346
IGFBP1	20,04070258	-0,076068955	0,514638577	-0,147810442	0,882492368	NA	24,33073606	17,35689163	14,13470175	22,38420614	16,91833507	26,15131817	8,128136143	12,61977479	31,16722107	6,687287081	31,79596026	23,57432603	2,251468559	9,422807886	15,00027229	28,0496987	14,72252257	10,75018398	5,992310078	16,70151103	66,8968773	26,17184017	27,4668136	14,03964885	35,48569462	12,85772332
IGFL1	7,4397563	-0,310314964	0,685302608	-0,452814509	0,650682315	NA	4,293659304	6,675727552	6,282089667	11,19210307	1,025353641	5,505540668	3,126206209	3,840801023	8,657561407	1,180109485	5,961742548	10,47747824	3,752447598	2,019173119	2,386406955	1,558316594	23,55603611	4,300073594	0,998718346	4,912209126	18,01069774	8,412377198	13,13630215	9,827754193	28,38855569	3,95622256
IL10	12,5392648	0,190725572	0,484875239	0,393349786	0,694061151	NA	7,871708725	14,68660061	7,852612084	12,59111595	6,152121845	11,01108134	5,627171176	11,52240307	9,523317548	6,687287081	7,948990065	5,238739119	10,50685327	12,11503871	4,431898631	20,25811573	14,72252257	22,57538637	5,992310078	11,7893019	36,02139547	9,347085775	32,24365074	6,317841981	14,19427785	18,79205716
IL12A	16,75297505	0,001747737	0,504719851	0,003462786	0,997237102	NA	11,44975815	14,68660061	17,27574659	18,18716749	6,152121845	16,51662201	11,25434235	9,327659627	12,98634211	6,293917253	21,85972268	11,78716302	15,76027991	8,076692474	5,454644468	11,42765502	32,38954965	15,05025758	3,994873385	10,80686008	54,03209321	15,89004582	25,07839502	14,74163129	53,22854193	11,86866768
IL12B	20,28730811	0,709514114	0,631121357	1,124211858	0,26092316	NA	17,17463722	5,340582041	7,852612084	61,55656689	11,27889005	19,26939234	15,63103104	10,97371721	15,58361053	2,753588798	9,936237581	15,71621736	8,255384715	11,441981	5,113729189	12,46653275	55,94558576	22,57538637	6,991028424	13,75418555	64,32392048	14,02062866	27,4668136	13,3376664	63,87425031	14,8358346
IL12RB2	21,62208581	0,848357659	0,414123846	2,048560276	0,040505132	NA	13,5965878	12,01630959	17,27574659	43,3693994	25,1211642	27,52770334	47,51833437	36,21326679	10,38907369	10,22761554	15,89798013	13,0968478	18,01174847	14,80726954	13,97752645	27,01082097	32,38954965	38,70066234	13,98205685	12,77174373	10,29182728	17,75946297	31,04944146	22,46343816	24,83998623	11,86866768
IL13	12,59206379	0,378746881	0,557806972	0,678992735	0,497142465	NA	9,302928493	12,01630959	7,852612084	44,76841228	5,126768204	9,63469617	7,502894901	6,584230325	19,0466351	5,507177596	5,961742548	9,167793458	12,75832183	6,057519356	7,159220865	6,233266377	20,6115316	10,75018398	6,991028424	4,912209126	25,72956819	17,75946297	10,74788358	11,23171908	39,03426408	4,9452782
IL17A	9,157906696	0,285431626	0,481697983	0,592553086	0,553480284	NA	5,009269189	4,005436531	9,423134501	11,19210307	9,740859588	9,63469617	3,126206209	6,584230325	3,463024563	3,933698283	7,948990065	6,548423898	6,754405676	5,384461649	7,159220865	4,674949783	20,6115316	9,675165586	7,989746771	11,7893019	18,01069774	14,95533724	4,776837147	8,423789308	28,38855569	8,901500759
IL17F	12,2370575	0,219728154	0,617711015	0,355713511	0,722055126	NA	8,587318609	10,68116408	23,55783625	58,75854112	5,639445024	16,51662201	4,376688692	2,743429302	8,657561407	4,327068111	25,83421771	6,548423898	8,255384715	2,019173119	3,409152793	10,90821616	8,833513541	8,600147187	4,993591732	8,841976427	20,58365456	11,21650293	13,13630215	11,93370152	21,29141677	7,912445119
IL18RAP	10,69867477	0,425595426	0,485438081	0,876724433	0,380636333	NA	9,302928493	6,675727552	6,282089667	9,793090187	11,27889005	9,63469617	7,502894901	4,389486883	5,194536844	5,507177596	13,91073261	3,929054339	9,756363754	8,74975018	4,090983351	9,349899566	17,66702708	15,05025758	6,991028424	13,75418555	28,30252501	9,347085775	27,4668136	5,615859539	17,74284731	10,87961204
IL1A	13,15400578	0,136700375	0,685849328	0,199315461	0,842015985	NA	10,01853838	14,68660061	12,56417933	58,75854112	4,614091384	6,881925836	7,502894901	3,840801023	8,657561407	3,146958626	17,88522765	10,47747824	6,754405676	6,057519356	2,386406955	9,349899566	41,22306319	6,450110391	1,997436693	8,841976427	20,58365456	13,08592009	9,553674295	4,211894654	42,58283354	9,890556399
IL2	12,21471579	0,018767716	0,592602008	0,031670018	0,974735205	NA	8,587318609	5,340582041	15,70522417	32,17729633	3,076060922	6,881925836	5,627171176	5,486858604	6,060292985	3,933698283	13,91073261	10,47747824	6,003916156	7,403634768	8,863797261	12,98597162	41,22306319	9,675165586	3,994873385	6,877092776	30,87548183	20,56358871	5,971046434	7,019824424	31,93712516	6,923389479
IL22	9,115865483	0,318999579	0,580487881	0,549537018	0,582636967	NA	4,293659304	6,675727552	1,570522417	25,18223191	3,588737743	5,505540668	4,376688692	4,938172744	4,328780704	5,507177596	9,936237581	13,0968478	10,50685327	6,057519356	3,409152793	6,233266377	20,6115316	7,525128789	0,998718346	11,7893019	20,58365456	7,47766862	14,33051144	6,317841981	21,29141677	10,87961204
IL23A	13,74829025	0,44694926	0,476626431	0,937734945	0,348380641	NA	10,73414826	10,68116408	9,423134501	44,76841228	9,740859588	4,129155501	11,25434235	17,00926167	12,12058597	9,047506051	25,83421771	11,78716302	11,25734279	4,711403943	4,77281391	11,94709389	23,55603611	10,75018398	6,991028424	15,7190692	20,58365456	12,15121151	11,94209287	11,23171908	28,38855569	6,923389479
IL27	10,53408877	0,509129486	0,625794465	0,813573008	0,415889606	NA	4,293659304	8,010873062	7,852612084	33,57630921	4,101414563	6,881925836	4,376688692	7,132916185	3,463024563	3,933698283	17,88522765	7,858108678	7,504895195	4,038346237	3,409152793	8,311021836	44,16756771	11,82520238	6,991028424	11,7893019	5,145913639	10,28179435	9,553674295	4,211894654	28,38855569	8,901500759
IL4	9,964247441	0,88116564	0,690329693	1,276441748	0,201799406	NA	5,724879073	6,675727552	3,141044834	23,78321903	2,050707282	13,76385167	4,376688692	3,840801023	6,060292985	3,933698283	23,84697019	5,238739119	6,754405676	4,711403943	1,022745838	8,311021836	26,50054062	5,375091992	2,996155039	8,841976427	20,58365456	10,28179435	9,553674295	7,721806866	39,03426408	4,9452782
IL5	7,638052565	0,995285027	0,640537366	1,553828208	0,1202254	NA	7,156098841	2,670291021	4,71156725	32,17729633	4,101414563	6,881925836	6,877653659	4,389486883	7,791805267	2,36021897	15,89798013	2,619369559	5,253426637	1,346115412	2,386406955	5,713827512	8,833513541	6,450110391	7,989746771	3,929767301	10,29182728	10,28179435	10,74788358	7,019824424	17,74284731	2,96716692
IL7	24,96469661	0,123641155	0,470176082	0,262967768	0,792575423	NA	15,74341745	20,02718265	17,27574659	46,16742517	10,76621323	23,39854784	16,25627229	11,52240307	5,194536844	10,62098536	33,78320777	24,88401081	24,76615414	17,49950036	8,181966703	16,10260481	38,27855868	39,77568074	13,98205685	14,73662738	59,17800685	32,71480021	28,66102288	23,86740304	70,97138924	24,726391
KAAG1	9,160145156	0,858167921	0,647449809	1,325458606	0,18501909	NA	1,431219768	6,675727552	3,141044834	30,77828345	4,101414563	8,258311003	6,877653659	6,584230325	10,38907369	2,753588798	7,948990065	9,167793458	5,253426637	3,365288531	2,727322234	13,50541048	8,833513541	4,300073594	3,994873385	7,859534601	20,58365456	8,412377198	7,165255721	12,63568396	35,48569462	5,934333839
KIR3DL2	18,35090851	0,483793109	0,52770279	0,916790887	0,359252229	NA	12,88097791	14,68660061	15,70522417	53,16248959	8,715505947	26,15131817	16,25627229	10,42503135	12,12058597	7,474026738	19,87247516	10,47747824	14,25930087	6,730577062	6,136475027	6,233266377	23,55603611	19,35033117	6,991028424	12,77174373	41,16730911	14,95533724	27,4668136	18,95352594	56,77711139	13,84677896
KIR_Activating_Su	11,9787626	0,061382735	0,477685898	0,128500203	0,897753138	NA	4,293659304	9,346018572	10,99365692	6,995064419	7,690152306	12,3874665	6,877653659	6,584230325	6,060292985	4,72043794	15,89798013	11,78716302	11,25734279	5,384461649	4,77281391	6,752705242	23,55603611	15,05025758	14,9807752	10,80686008	33,44843865	12,15121151	19,10734859	9,125771751	35,48569462	5,934333839
KIR_Activating_Su	21,96920486	0,704409947	0,623491113	1,12978346	0,258567479	NA	12,88097791	18,69203714	12,56417933	81,14274727	8,715505947	24,77493301	14,38054856	6,584230325	18,18087896	7,474026738	23,84697019	11,78716302	15,00979039	8,076692474	6,136475027	14,02484935	32,38954965	21,50036797	5,992310078	10,80686008	72,04279094	15,89004582	29,85523217	14,03964885	74,5199587	9,890556399
KIR_Inhibiting_Sul	8,732547263	-0,121711397	0,549906409	-0,221331112	0,824834627	NA	6,440488957	4,005436531	10,99365692	6,995064419	5,639445024	5,505540668	5,001929934	5,486858604	7,791805267	3,540328455	25,83421771	11,78716302	4,502937117	2,692230825	3,750068072	9,86933843	14,72252257	7,525128789	4,993591732	8,841976427	25,72956819	6,542960043	9,553674295	7,019824424	21,29141677	0,98905564
KIR_Inhibiting_Sul	11,20352882	0,325186064	0,607832655	0,53499275	0,592654871	NA	8,587318609	6,675727552	3,141044834	6,995064419	6,152121845	11,01108134	5,627171176	8,230287906	5,194536844	7,08065691	17,88522765	10,47747824	11,25734279	5,384461649	3,409152793	2,597194324	17,66702708	9,675165586	8,988465117	4,912209126	28,30252501	11,21650293	19,10734859	8,423789308	60,32568085	2,96716692
KLRC1	16,12960823	0,308959399	0,565125662	0,5467092	0,584578532	NA	7,871708725	6,675727552	26,69888109	34,9753221	15,38030461	19,26939234	9,378618626	4,938172744	16,44936667	5,900547425	21,85972268	10,47747824	9,756363754	4,711403943	6,136475027	12,98597162	41,22306319	10,75018398	8,988465117	10,80686008	25,72956819	13,08592009	15,52472073	7,019824424	63,87425031	8,901500759
KLRD1	19,78289364	0,140811153	0,501244176	0,280923271	0,778769252	NA	6,440488957	22,69747368	14,13470175	30,77828345	11,27889005	24,77493301	10,62910111	9,876345488	19,91239124	6,687287081	21,85972268	20,95495648	14,25930087	10,7689233	8,181966703	15,06372708	50,05657673	21,50036797	6,991028424	16,70151103	46,31322275	13,08592009	15,52472073	12,63568396	67,42281978	15,82489024
KLRG1	17,4785338	-0,055707443	0,523419797	-0,106429759	0,915241383	NA	9,302928493	8,010873062	15,70522417	12,59111595	7,177475486	9,63469617	15,0057898	10,42503135	12,98634211	5,507177596	7,948990065	20,95495648	31,52055982	3,365288531	6,136475027	16,62204367	26,50054062	30,10051516	8,988465117	8,841976427	38,59435229	14,02062866	32,24365074	7,721806866	63,87425031	30,66072484
LAG3	15,54654726	0,084782651	0,408455709	0,207568774	0,835565688	NA	11,44975815	16,02174612	12,56417933	13,99012884	12,30424369	5,505540668	13,13006608	16,46057581	8,657561407	10,22761554	29,80871274	13,0968478	9,005874235	9,422807886	8,522881982	12,98597162	20,6115316	30,10051516	10,98590181	9,824418252	18,01069774	12,15121151	27,4668136	10,52973664	42,58283354	18,79205716
LTA	11,19117287	0,811961953	0,588746203	1,379137476	0,167852371	NA	7,156098841	2,670291021	10,99365692	47,56643805	4,614091384	9,63469617	5,627171176	7,132916185	6,926049126	5,507177596	5,961742548	5,238739119	9,005874235	7,403634768	4,431898631	12,46653275	29,44504514	13,97523918	2,996155039	11,7893019	10,29182728	8,412377198	17,9131393	11,23171908	17,74284731	14,8358346
MALL	14,87202661	0,444224489	0,495906346	0,89578303	0,370368655	NA	8,587318609	13,3514551	10,99365692	61,55656689	7,690152306	13,76385167	12,50482484	12,61977479	15,58361053	7,867396566	5,961742548	13,0968478	15,00979039	10,09586559	7,500136144	12,98597162	35,33405417	15,05025758	7,989746771	6,877092776	23,15661137	12,15121151	17,9131393	9,827754193	21,29141677	7,912445119
MASP2	19,82757762	-0,088301958	0,600469716	-0,147054806	0,883088762	NA	9,302928493	22,69747368	14,13470175	30,77828345	7,690152306	12,3874665	8,753377385	6,584230325	10,38907369	8,260766395	17,88522765	17,02590214	11,25734279	8,74975018	3,068237513	9,349899566	47,11207222	21,50036797	6,991028424	10,80686008	43,74026593	16,8247544	26,27260431	16,14559617	110,0056533	17,80300152
MMP12	14,43659482	-0,024124576	0,465348206	-0,051841989	0,958654598	NA	15,02780757	13,3514551	9,423134501	16,78815461	16,40565825	13,76385167	8,128136143	7,681602046	6,926049126	1,966849142	23,84697019	11,78716302	9,005874235	10,09586559	10,90928894	16,10260481	11,77801806	13,97523918	7,989746771	9,824418252	54,03209321	17,75946297	17,9131393	14,74163129	21,29141677	14,8358346
MPIG6B	8,867502377	0,961652195	0,647475953	1,485232293	0,1374823	NA	2,862439536	6,675727552	3,141044834	34,9753221	5,126768204	9,63469617	5,627171176	8,230287906	7,791805267	4,72043794	7,948990065	6,548423898	5,253426637	8,076692474	2,727322234	6,752705242	29,44504514	9,675165586	3,994873385	1,96488365	23,15661137	4,673542888	8,359465008	7,019824424	14,19427785	1,97811128
MS4A1	23,30591688	0,12568826	0,690351902	0,182064046	0,855532464	NA	9,302928493	20,02718265	10,99365692	27,98025768	5,126768204	8,258311003	14,38054856	8,230287906	13,85209825	5,507177596	5,961742548	11,78716302	18,01174847	2,019173119	3,750068072	68,56593015	23,55603611	27,95047836	3,994873385	12,77174373	33,44843865	24,30242302	118,2267194	14,03964885	67,42281978	46,48561508
MS4A2	12,82330151	0,084695844	0,551696726	0,153518846	0,87798913	NA	6,440488957	12,01630959	14,13470175	29,37927056	3,588737743	9,63469617	10,00385987	8,230287906	8,657561407	3,540328455	11,9234851	10,47747824	27,0176227	6,730577062	8,181966703	10,3887773	17,66702708	43,00073594	8,988465117	9,824418252	10,29182728	10,28179435	17,9131393	2,105947327	14,19427785	18,79205716
MX2	20,82717762	-0,141859158	0,382032643	-0,371327321	0,710393751	NA	22,18390641	22,69747368	23,55783625	15,38914172	9,228182767	15,14023684	22,5086847	30,72640818	22,50965966	13,37457416	15,89798013	17,02590214	28,51860174	9,422807886	13,63661117	15,58316594	41,22306319	45,15077273	14,9807752	16,70151103	28,30252501	18,69417155	34,63206932	16,14559617	3,548569462	24,726391
MYBL1	20,31958061	0,257547031	0,487574089	0,528221323	0,597345731	NA	12,16536803	6,675727552	18,846269	22,38420614	11,27889005	17,89300717	16,88151353	9,876345488	17,31512281	8,260766395	13,91073261	18,33558692	21,01370655	11,441981	7,159220865	17,6609214	35,33405417	29,02549676	15,97949354	7,859534601	38,59435229	11,21650293	29,85523217	14,74163129	95,81137547	18,79205716
NCR1	16,19496331	0,029251924	0,553445675	0,052854192	0,957848083	NA	5,724879073	18,69203714	7,852612084	20,98519326	7,177475486	17,89300717	6,252412418	6,584230325	13,85209825	3,933698283	9,936237581	17,02590214	12,75832183	9,422807886	5,454644468	12,46653275	32,38954965	18,27531277	4,993591732	14,73662738	59,17800685	16,8247544	17,9131393	10,52973664	60,32568085	9,890556399
NPPA	10,37364973	0,547021931	0,658838944	0,830281719	0,406379521	NA	7,156098841	5,340582041	10,99365692	54,56150247	4,614091384	9,63469617	6,252412418	6,035544465	8,657561407	2,753588798	15,89798013	7,858108678	4,502937117	3,365288531	1,363661117	6,752705242	20,6115316	3,225055195	5,992310078	6,877092776	12,8647841	12,15121151	14,33051144	9,125771751	24,83998623	3,95622256
NPPB	12,48690297	-0,016813566	0,476182785	-0,035309058	0,9718333	NA	5,009269189	13,3514551	15,70522417	33,57630921	7,690152306	8,258311003	6,877653659	6,035544465	10,38907369	5,900547425	15,89798013	13,0968478	11,25734279	10,09586559	6,477390306	8,830460701	14,72252257	8,600147187	3,994873385	14,73662738	36,02139547	13,08592009	16,71893002	9,125771751	21,29141677	7,912445119
OASL	15,11761965	0,469287102	0,460015682	1,020154572	0,307655158	NA	11,44975815	9,346018572	4,71156725	20,98519326	7,690152306	13,76385167	9,378618626	10,97371721	20,77814738	14,55468365	9,936237581	14,40653258	9,756363754	8,076692474	9,20471254	11,94709389	32,38954965	27,95047836	8,988465117	7,859534601	25,72956819	19,62888013	39,40890646	8,423789308	24,83998623	10,87961204
PDCD1	13,5196387	-0,121668919	0,472114594	-0,257710565	0,796630292	NA	9,302928493	13,3514551	12,56417933	20,98519326	3,076060922	15,14023684	7,502894901	8,778973767	10,38907369	4,72043794	13,91073261	13,0968478	10,50685327	10,09586559	6,136475027	5,713827512	17,66702708	18,27531277	4,993591732	13,75418555	33,44843865	14,02062866	25,07839502	12,63568396	35,48569462	10,87961204
PDCD1LG2	18,74788031	0,063412904	0,360128597	0,176084056	0,860227907	NA	16,45902733	18,69203714	20,41679142	25,18223191	10,76621323	20,64577751	18,13199601	14,26583237	26,83844036	12,58783451	13,91073261	14,40653258	23,26517511	9,422807886	15,34118757	21,81643232	32,38954965	38,70066234	15,97949354	5,894650951	23,15661137	14,95533724	21,49576716	23,86740304	7,097138924	21,75922408
PHEX	14,09979354	-0,009636643	0,456882052	-0,02109219	0,983172115	NA	11,44975815	13,3514551	7,852612084	8,394077303	9,740859588	13,76385167	6,877653659	11,52240307	13,85209825	19,27512159	17,88522765	18,33558692	12,75832183	8,076692474	8,522881982	5,713827512	11,77801806	5,375091992	14,9807752	8,841976427	33,44843865	11,21650293	21,49576716	11,93370152	53,22854193	6,923389479
PNOC	12,08180307	0,296127387	0,519872814	0,569615065	0,568938808	NA	8,587318609	5,340582041	7,852612084	27,98025768	8,715505947	15,14023684	6,252412418	8,230287906	7,791805267	4,327068111	15,89798013	14,40653258	13,50881135	4,711403943	4,77281391	14,54428821	26,50054062	12,90022078	4,993591732	3,929767301	20,58365456	11,21650293	15,52472073	7,019824424	35,48569462	7,912445119
POU2AF1	18,53846861	1,256005401	0,599114884	2,096434982	0,036043622	NA	7,156098841	4,005436531	6,282089667	69,95064419	6,664798665	9,63469617	12,50482484	12,61977479	13,85209825	8,260766395	7,948990065	13,0968478	26,26713318	6,057519356	7,500136144	24,93306551	41,22306319	21,50036797	11,98462016	14,73662738	38,59435229	13,08592009	23,88418574	5,615859539	39,03426408	35,60600304
PPBP	12,70261509	-0,141512999	0,590086057	-0,239817561	0,810471693	NA	5,724879073	12,01630959	9,423134501	11,19210307	5,639445024	5,505540668	5,001929934	3,292115163	9,523317548	7,08065691	13,91073261	13,0968478	3,752447598	16,15338495	4,090983351	10,3887773	32,38954965	8,600147187	7,989746771	9,824418252	54,03209321	15,89004582	10,74788358	4,913877096	46,131403	3,95622256
PTPN22	23,05989937	0,2449275	0,431335668	0,567835027	0,570146993	NA	17,8902471	14,68660061	10,99365692	11,19210307	19,994396	16,51662201	20,00771974	19,20400511	16,44936667	7,867396566	17,88522765	19,6452717	34,5225179	11,441981	9,886543099	31,16633189	38,27855868	44,07575434	20,97308527	7,859534601	38,59435229	21,49829728	53,73941791	12,63568396	39,03426408	43,51844816
RAG2	13,38986496	1,139330601	0,556046652	2,048983834	0,040463696	NA	4,293659304	6,675727552	4,71156725	51,7634767	8,202829126	19,26939234	9,378618626	7,681602046	12,98634211	5,900547425	17,88522765	10,47747824	14,25930087	7,403634768	5,795559748	8,311021836	23,55603611	9,675165586	8,988465117	6,877092776	33,44843865	17,75946297	9,553674295	10,52973664	24,83998623	7,912445119
SCGB1A1	24,93963477	-0,092082602	0,556791299	-0,16538082	0,868644249	NA	17,17463722	14,68660061	23,55783625	37,77334786	8,715505947	17,89300717	13,75530732	10,42503135	19,91239124	7,867396566	29,80871274	31,43243471	13,50881135	12,11503871	6,818305585	15,06372708	53,00108125	23,65040477	12,9833385	12,77174373	43,74026593	14,95533724	32,24365074	20,35749083	138,394209	15,82489024
SELL	19,72764948	0,40311237	0,433153593	0,930645334	0,352037056	NA	7,156098841	13,3514551	20,41679142	25,18223191	15,38030461	17,89300717	16,88151353	10,97371721	12,98634211	11,01435519	23,84697019	14,40653258	18,76223799	10,7689233	7,841051423	36,36072053	20,6115316	30,10051516	14,9807752	11,7893019	30,87548183	21,49829728	34,63206932	7,019824424	42,58283354	35,60600304
SH2D1A	16,0274247	-0,322789472	0,530872725	-0,608035518	0,543163917	NA	12,16536803	17,35689163	10,99365692	11,19210307	7,690152306	9,63469617	5,627171176	8,778973767	15,58361053	8,654136223	3,974495032	14,40653258	12,00783231	7,403634768	3,750068072	8,830460701	41,22306319	30,10051516	6,991028424	9,824418252	36,02139547	19,62888013	35,8262786	8,423789308	56,77711139	13,84677896
SH2D1B	14,89964769	0,32601762	0,558380971	0,583862339	0,559312933	NA	8,587318609	12,01630959	14,13470175	55,96051536	10,25353641	9,63469617	14,38054856	8,778973767	13,85209825	1,966849142	19,87247516	14,40653258	14,25930087	3,365288531	8,181966703	22,33587118	32,38954965	16,12527598	5,992310078	9,824418252	10,29182728	11,21650293	20,30155788	8,423789308	31,93712516	8,901500759
SIGLEC5	20,49398834	0,402725602	0,509137074	0,790996418	0,428946082	NA	9,302928493	13,3514551	20,41679142	54,56150247	7,690152306	15,14023684	22,5086847	14,81451823	10,38907369	7,474026738	33,78320777	18,33558692	9,005874235	10,09586559	6,818305585	8,311021836	29,44504514	22,57538637	10,98590181	20,63127833	33,44843865	18,69417155	26,27260431	21,76145571	60,32568085	26,70450228
SIRPG	20,00642387	-0,141832559	0,478085156	-0,296667982	0,766720003	NA	12,16536803	16,02174612	20,41679142	16,78815461	9,228182767	20,64577751	14,38054856	9,876345488	19,91239124	9,834245708	7,948990065	20,95495648	17,26125895	7,403634768	8,522881982	12,98597162	26,50054062	30,10051516	10,98590181	6,877092776	61,75096367	26,17184017	17,9131393	18,2515435	74,5199587	22,74827972
SLAMF6	22,97650686	0,823845861	0,527460989	1,561908612	0,118309519	NA	20,03707675	10,68116408	4,71156725	69,95064419	23,07045692	13,76385167	20,63296098	12,07108893	12,98634211	7,08065691	13,91073261	14,40653258	28,51860174	8,076692474	12,61386533	48,30781442	26,50054062	30,10051516	23,96924031	15,7190692	25,72956819	12,15121151	70,45834792	16,84757862	28,38855569	26,70450228
SPIB	16,53972324	1,103578223	0,625849977	1,763327097	0,077845338	NA	9,302928493	5,340582041	4,71156725	53,16248959	8,202829126	12,3874665	9,378618626	6,035544465	12,12058597	7,474026738	7,948990065	9,167793458	24,76615414	8,74975018	5,113729189	18,18036027	29,44504514	15,05025758	4,993591732	6,877092776	25,72956819	14,95533724	47,76837147	6,317841981	28,38855569	48,46372636
TBX21	16,50190829	0,050816006	0,58381619	0,087041105	0,930638839	NA	12,88097791	9,346018572	10,99365692	39,17236075	4,614091384	6,881925836	7,502894901	5,486858604	10,38907369	3,933698283	7,948990065	14,40653258	14,25930087	4,038346237	5,454644468	14,02484935	29,44504514	26,87545996	8,988465117	11,7893019	30,87548183	14,02062866	31,04944146	13,3376664	74,5199587	16,81394588
TCL1A	16,38405784	0,531641039	0,546097512	0,97352767	0,330291115	NA	4,293659304	10,68116408	14,13470175	30,77828345	7,690152306	12,3874665	9,378618626	9,876345488	14,71785439	5,507177596	11,9234851	10,47747824	15,00979039	7,403634768	5,795559748	7,272144107	38,27855868	19,35033117	7,989746771	10,80686008	38,59435229	14,95533724	33,43786003	14,03964885	60,32568085	10,87961204
THEMIS	18,0031808	0,05426055	0,514719026	0,105417806	0,916044288	NA	12,16536803	17,35689163	15,70522417	37,77334786	15,89298143	4,129155501	10,00385987	8,230287906	11,25482983	4,72043794	9,936237581	10,47747824	18,01174847	8,74975018	8,181966703	18,18036027	14,72252257	39,77568074	10,98590181	12,77174373	25,72956819	14,02062866	44,18574361	11,23171908	53,22854193	30,66072484
TLR7	21,19064229	0,383048117	0,535889916	0,714788814	0,474739548	NA	9,302928493	13,3514551	18,846269	44,76841228	8,202829126	24,77493301	12,50482484	9,327659627	22,50965966	10,62098536	21,85972268	19,6452717	19,51272751	12,11503871	6,818305585	7,272144107	44,16756771	27,95047836	3,994873385	8,841976427	38,59435229	28,04125733	31,04944146	13,3376664	63,87425031	29,6716692
TNFAIP6	22,24591914	-0,126819211	0,532975425	-0,237945701	0,811923209	NA	14,31219768	18,69203714	17,27574659	18,18716749	4,101414563	26,15131817	11,25434235	8,778973767	21,64390352	8,260766395	21,85972268	24,88401081	13,50881135	16,15338495	6,136475027	12,46653275	29,44504514	29,02549676	17,97693023	13,75418555	30,87548183	14,02062866	35,8262786	18,2515435	127,7485006	17,80300152
TNFRSF18	12,81029164	0,297576252	0,427458507	0,696152369	0,486333413	NA	10,01853838	5,340582041	12,56417933	26,58124479	10,25353641	20,64577751	11,25434235	11,52240307	13,85209825	4,327068111	9,936237581	13,0968478	11,25734279	8,076692474	6,136475027	8,830460701	17,66702708	8,600147187	6,991028424	10,80686008	30,87548183	13,08592009	19,10734859	11,23171908	14,19427785	16,81394588
TNFRSF9	10,01832007	0,261658604	0,533600614	0,490364136	0,62387625	NA	9,302928493	6,675727552	6,282089667	36,37433498	4,101414563	11,01108134	4,376688692	7,132916185	7,791805267	4,327068111	7,948990065	9,167793458	10,50685327	4,038346237	5,795559748	6,233266377	23,55603611	9,675165586	9,987183463	6,877092776	15,43774092	4,673542888	7,165255721	6,317841981	24,83998623	10,87961204
TNFSF18	13,04721653	-1,092014048	0,564809991	-1,933418432	0,053184672	NA	17,17463722	20,02718265	9,423134501	16,78815461	6,664798665	12,3874665	2,500964967	4,389486883	5,194536844	5,507177596	7,948990065	27,50338037	6,754405676	5,384461649	1,363661117	7,791582971	17,66702708	11,82520238	7,989746771	8,841976427	23,15661137	10,28179435	17,9131393	8,423789308	67,42281978	8,901500759
TNFSF9	21,89086072	-0,396012031	0,613723684	-0,645261119	0,518757985	NA	14,31219768	17,35689163	21,98731384	11,19210307	7,690152306	20,64577751	8,128136143	3,840801023	13,85209825	5,900547425	19,87247516	19,6452717	21,76419607	9,422807886	4,77281391	12,46653275	53,00108125	20,42534957	7,989746771	14,73662738	43,74026593	12,15121151	31,04944146	16,84757862	145,4913479	10,87961204
TRAT1	23,7097973	0,041514871	0,483928069	0,085787277	0,931635521	NA	15,02780757	12,01630959	25,12835867	51,7634767	9,740859588	17,89300717	11,25434235	13,71714651	12,98634211	7,867396566	21,85972268	26,19369559	23,26517511	9,422807886	7,841051423	19,73867686	55,94558576	38,70066234	12,9833385	19,6488365	46,31322275	19,62888013	60,90467363	20,35749083	35,48569462	20,77016844
TRDC	12,33102384	0,239204192	0,471143097	0,507710277	0,61165654	NA	10,73414826	6,675727552	9,423134501	15,38914172	7,690152306	11,01108134	8,753377385	6,584230325	13,85209825	5,113807768	13,91073261	9,167793458	21,01370655	13,46115412	4,77281391	6,752705242	20,6115316	21,50036797	1,997436693	13,75418555	28,30252501	11,21650293	20,30155788	8,423789308	21,29141677	8,901500759
TRDN	12,32103355	-0,642806708	0,414079273	-1,552375958	0,120572293	NA	20,75268664	13,3514551	14,13470175	5,596051536	7,690152306	9,63469617	6,877653659	6,035544465	8,657561407	10,62098536	17,88522765	11,78716302	12,75832183	17,49950036	6,818305585	5,713827512	8,833513541	21,50036797	6,991028424	12,77174373	10,29182728	6,542960043	16,71893002	5,615859539	35,48569462	19,7811128
TRDV3	18,33456692	-1,05154209	0,554392029	-1,896748215	0,057861177	NA	27,1931756	30,70834674	32,98097075	60,15755401	5,639445024	11,01108134	8,753377385	5,486858604	7,791805267	8,260766395	11,9234851	31,43243471	12,00783231	18,17255807	6,818305585	9,86933843	41,22306319	13,97523918	2,996155039	27,5083711	30,87548183	12,15121151	10,74788358	5,615859539	35,48569462	7,912445119
WNT9A	14,09777522	-0,261382869	0,328937194	-0,794628501	0,426829635	NA	17,17463722	16,02174612	9,423134501	13,99012884	10,25353641	12,3874665	10,62910111	12,07108893	12,98634211	9,440875879	5,961742548	13,0968478	9,756363754	10,09586559	8,522881982	13,50541048	17,66702708	20,42534957	11,98462016	14,73662738	28,30252501	9,347085775	15,52472073	10,52973664	31,93712516	20,77016844
XCL1/2	15,94798841	0,881539864	0,538005022	1,63853464	0,101310214	NA	9,302928493	6,675727552	10,99365692	53,16248959	9,228182767	9,63469617	6,252412418	13,71714651	12,12058597	5,507177596	9,936237581	6,548423898	15,76027991	8,076692474	5,795559748	18,18036027	23,55603611	30,10051516	10,98590181	6,877092776	33,44843865	18,69417155	21,49576716	11,93370152	31,93712516	24,726391
