# Supplementary material for: Can Gene Expression Analysis in Zero-Time Biopsies Predict Kidney Transplant Rejection?
Source: Front Med (Lausanne). 2022 Mar 30;9:793744. doi: 10.3389/fmed.2022.793744 (PMC9005644; doi:10.3389/fmed.2022.793744)
Supplement: Supplementary file 6 [file Table_6.pdf]

Pathway enrichment GO LD vs DD

ID	Description	GeneRatio	BgRatio	pvalue	p.adjust	qvalue	geneID	Count
GO:0043687	post-translational protein modification	15/182	21/749	4,97E-06	0,010963518	0,010963518	HIF1A/TIMP1/C3/PSMB8/SOCS3/ASB15/PSME2/PSMB10/APOL1/TNC/VCAN/APOE/PSME1/FN1/KLHL13	15
GO:0002283	neutrophil activation involved in immune response	30/182	69/749	0,000183439	0,134766565	0,134766565	SERPINA3/S100A9/LTF/FPR1/S100A8/C3/SLPI/FCER1G/FCGR3B/MME/LCN2/IMPDH1/ALOX5/FCGR2A/CD59/CTSS/CXCL1	30
GO:0043312	neutrophil degranulation	30/182	69/749	0,000183439	0,134766565	0,134766565	SERPINA3/S100A9/LTF/FPR1/S100A8/C3/SLPI/FCER1G/FCGR3B/MME/LCN2/IMPDH1/ALOX5/FCGR2A/CD59/CTSS/CXCL1	30
GO:0002446	neutrophil mediated immunity	30/182	70/749	0,000252884	0,139339102	0,139339102	SERPINA3/S100A9/LTF/FPR1/S100A8/C3/SLPI/FCER1G/FCGR3B/MME/LCN2/IMPDH1/ALOX5/FCGR2A/CD59/CTSS/CXCL1	30
GO:0045055	regulated exocytosis	37/182	99/749	0,001252391	0,460044793	0,460044793	SERPINA3/S100A9/LTF/FPR1/TIMP1/S100A8/C3/SLPI/SERPING1/FCER1G/FCGR3B/MME/LCN2/IMPDH1/IL4R/TGFB1/ALC	37
GO:0042119	neutrophil activation	30/182	75/749	0,001080622	0,460044793	0,460044793	SERPINA3/S100A9/LTF/FPR1/S100A8/C3/SLPI/FCER1G/FCGR3B/MME/LCN2/IMPDH1/ALOX5/FCGR2A/CD59/CTSS/CXCL1	30
GO:0002275	myeloid cell activation involved in immune response	31/182	81/749	0,002131119	0,521887302	0,521887302	SERPINA3/S100A9/LTF/FPR1/S100A8/C3/SLPI/FCER1G/FCGR3B/MME/LCN2/IMPDH1/IL4R/ALOX5/FCGR2A/CD59/CTSS/	31
GO:0043299	leukocyte degranulation	31/182	81/749	0,002131119	0,521887302	0,521887302	SERPINA3/S100A9/LTF/FPR1/S100A8/C3/SLPI/FCER1G/FCGR3B/MME/LCN2/IMPDH1/IL4R/ALOX5/FCGR2A/CD59/CTSS/	31
GO:0036230	granulocyte activation	30/182	77/749	0,001810774	0,521887302	0,521887302	SERPINA3/S100A9/LTF/FPR1/S100A8/C3/SLPI/FCER1G/FCGR3B/MME/LCN2/IMPDH1/ALOX5/FCGR2A/CD59/CTSS/CXCL1	30
GO:0002444	myeloid leukocyte mediated immunity	31/182	82/749	0,002695502	0,568502765	0,568502765	SERPINA3/S100A9/LTF/FPR1/S100A8/C3/SLPI/FCER1G/FCGR3B/MME/LCN2/IMPDH1/IL4R/ALOX5/FCGR2A/CD59/CTSS/	31
GO:0070741	response to interleukin-6	9/182	15/749	0,003095296	0,568502765	0,568502765	JAK1/STAT3/SOCS3/ICAM1/STAT1/IL6R/ABCC2/NFKB1/IL6ST	9
GO:0071354	cellular response to interleukin-6	9/182	15/749	0,003095296	0,568502765	0,568502765	JAK1/STAT3/SOCS3/ICAM1/STAT1/IL6R/ABCC2/NFKB1/IL6ST	9
GO:0051248	negative regulation of protein metabolic process	41/182	118/749	0,003546836	0,569294933	0,569294933	SERPINA3/LTF/BCL3/RARRES1/TIMP1/STAT3/C3/SLPI/SERPING1/SOCS3/HDAC6/JAK3/TGFB1/SOCS1/DNMT1/BAX/CDKN	41
GO:0006887	exocytosis	37/182	104/749	0,003616211	0,569294933	0,569294933	SERPINA3/S100A9/LTF/FPR1/TIMP1/S100A8/C3/SLPI/SERPING1/FCER1G/FCGR3B/MME/LCN2/IMPDH1/IL4R/TGFB1/ALC	37
GO:0002526	acute inflammatory response	16/182	36/749	0,005294568	0,77794851	0,77794851	SERPINA3/OSMR/CD163/STAT3/S100A8/C3/FCER1G/ICAM1/VCAM1/SIGIRR/IL6R/APOL2/FN1/EPO/GATA3/IL6ST	16
GO:0002263	cell activation involved in immune response	45/182	143/749	0,018756774	0,961873513	0,961873513	SERPINA3/S100A9/LTF/BCL3/FPR1/BCL6/STAT3/S100A8/C3/SLPI/FCER1G/FCGR3B/MME/LCN2/ICAM1/IMPDH1/RELB/JA	45
GO:0002366	leukocyte activation involved in immune response	45/182	143/749	0,018756774	0,961873513	0,961873513	SERPINA3/S100A9/LTF/BCL3/FPR1/BCL6/STAT3/S100A8/C3/SLPI/FCER1G/FCGR3B/MME/LCN2/ICAM1/IMPDH1/RELB/JA	45
GO:0006508	proteolysis	44/182	136/749	0,011713066	0,961873513	0,961873513	SERPINA3/S100A9/HIF1A/LTF/RARRES1/C1S/TIMP1/STAT3/S100A8/C3/SLPI/ADAMTS1/SERPING1/CFB/PSMB8/MME/HD	44
GO:0010243	response to organonitrogen compound	39/182	119/749	0,01432309	0,961873513	0,961873513	TIMP1/STAT3/COL4A1/NNMT/FCER1G/SOCS3/MAPK13/COL1A1/NR4A1/COL3A1/ICAM1/JAK3/STAT1/TGFB1/SOCS1/XBI	39
GO:0032269	negative regulation of cellular protein metabolic process	35/182	102/749	0,009324985	0,961873513	0,961873513	SERPINA3/LTF/RARRES1/TIMP1/STAT3/C3/SLPI/SERPING1/SOCS3/HDAC6/TGFB1/SOCS1/DNMT1/BAX/CDKN1A/AGT/FKE	35
GO:0009636	response to toxic substance	27/182	76/749	0,013801829	0,961873513	0,961873513	S100A9/SOD2/STAT3/S100A8/MAPK13/LCN2/HDAC6/COL1A1/ICAM1/STAT1/BAX/AHR/CDKN1A/VCAM1/STAT6/TNC/EG	27
GO:1901699	cellular response to nitrogen compound	26/182	74/749	0,018314714	0,961873513	0,961873513	STAT3/COL4A1/SOCS3/MAPK13/COL1A1/NR4A1/COL3A1/ICAM1/JAK3/STAT1/TGFB1/SOCS1/XBP1/DNMT1/AHR/VCAM	26
GO:0071417	cellular response to organonitrogen compound	25/182	71/749	0,0200754	0,961873513	0,961873513	STAT3/COL4A1/SOCS3/MAPK13/COL1A1/NR4A1/COL3A1/ICAM1/JAK3/STAT1/TGFB1/SOCS1/XBP1/DNMT1/AHR/VCAM	25
GO:0043434	response to peptide hormone	20/182	50/749	0,007992089	0,961873513	0,961873513	TIMP1/STAT3/SOCS3/COL1A1/NR4A1/ICAM1/JAK3/STAT1/TGFB1/SOCS1/XBP1/STAT6/AGT/EGR1/TLR2/ABCC2/TNFSF10	20
GO:0060759	regulation of response to cytokine stimulus	20/182	51/749	0,010337067	0,961873513	0,961873513	HIF1A/JAK1/CD24/IFNGR2/IL1R1/SOCS3/TNFRSF1A/IFNAR2/STAT1/SOCS1/SIGIRR/TLR4/TLR2/IRF7/IL1R2/TBK1/BIRC3/C	20
GO:0030198	extracellular matrix organization	20/182	53/749	0,016678171	0,961873513	0,961873513	BCL3/TIMP1/COL4A1/ITGB6/TNFRSF1A/COL1A1/COL3A1/ICAM1/TGFB1/VCAM1/AGT/TNC/NFKB2/CTSS/VCAN/TNFRSF1	20
GO:0043062	extracellular structure organization	20/182	53/749	0,016678171	0,961873513	0,961873513	BCL3/TIMP1/COL4A1/ITGB6/TNFRSF1A/COL1A1/COL3A1/ICAM1/TGFB1/VCAM1/AGT/TNC/NFKB2/CTSS/VCAN/TNFRSF1	20
GO:0071214	cellular response to abiotic stimulus	20/182	53/749	0,016678171	0,961873513	0,961873513	MAPK13/TNFRSF1A/MME/LTBR/COL1A1/RELB/TGFB1/BAX/CDKN1A/AGT/EGR1/TLR4/MYD88/MYC/ERRFI1/NOX4/EPO/	20
GO:0104004	cellular response to environmental stimulus	20/182	53/749	0,016678171	0,961873513	0,961873513	MAPK13/TNFRSF1A/MME/LTBR/COL1A1/RELB/TGFB1/BAX/CDKN1A/AGT/EGR1/TLR4/MYD88/MYC/ERRFI1/NOX4/EPO/	20
GO:0032446	protein modification by small protein conjugation	16/182	40/749	0,017489693	0,961873513	0,961873513	HIF1A/PSMB8/SOCS3/ASB15/HDAC6/PSME2/PSMB10/SOCS1/EGR1/FKBP1A/BIRC3/PSME1/DCAF12/KLHL13/ISG15/RNF	16
GO:0097193	intrinsic apoptotic signaling pathway	15/182	37/749	0,018599307	0,961873513	0,961873513	S100A9/HIF1A/BCL3/SOD2/S100A8/CD24/MUC1/TNFRSF1A/XBP1/BAX/CDKN1A/TNFRSF1B/EPO/CASP4/PLAUR	15
GO:0071375	cellular response to peptide hormone stimulus	13/182	30/749	0,0152262	0,961873513	0,961873513	STAT3/SOCS3/NR4A1/JAK3/STAT1/TGFB1/SOCS1/XBP1/STAT6/AGT/ERRFI1/GDF15/NFKB1	13
GO:0010212	response to ionizing radiation	10/182	21/749	0,015599381	0,961873513	0,961873513	ICAM1/TGFB1/BAX/CDKN1A/VCAM1/EGR1/MYC/NOX4/GATA3/THBD	10
GO:0021782	glial cell development	9/182	17/749	0,009433689	0,961873513	0,961873513	S100A8/C1QA/TGFB1/LDLR/TLR4/TLR2/C5AR1/IFNGR1/ADORA2A	9
GO:0043618	regulation of transcription from RNA polymerase II promoter in	9/182	17/749	0,009433689	0,961873513	0,961873513	HIF1A/PSMB8/MUC1/PSME2/PSMB10/EGR1/PSME1/EPO/ATF3	9
GO:0043620	regulation of DNA-templated transcription in response to stress	9/182	18/749	0,014959918	0,961873513	0,961873513	HIF1A/PSMB8/MUC1/PSME2/PSMB10/EGR1/PSME1/EPO/ATF3	9
GO:0045071	negative regulation of viral genome replication	8/182	15/749	0,013672102	0,961873513	0,961873513	LTF/IFITM3/SLPI/IFITM2/IFITM1/ISG20/IFIT1/ISG15	8
GO:0046916	cellular transition metal ion homeostasis	8/182	15/749	0,013672102	0,961873513	0,961873513	S100A9/HIF1A/LTF/S100A8/LCN2/MT1A/SLC11A1/MYC	8
GO:0043487	regulation of RNA stability	7/182	12/749	0,011508766	0,961873513	0,961873513	PSMB8/PSME2/PSMB10/MYD88/SLC11A1/TNFRSF1B/PSME1	7
GO:0097242	amyloid-beta clearance	7/182	12/749	0,011508766	0,961873513	0,961873513	C3/MME/LDLR/APOE/ITGB2/C5AR1/IFNGR1	7
GO:0009308	amine metabolic process	7/182	13/749	0,019877161	0,961873513	0,961873513	PSMB8/PSME2/PSMB10/VCAM1/ABCC2/ITGB2/PSME1	7
GO:0061418	regulation of transcription from RNA polymerase II promoter in	7/182	13/749	0,019877161	0,961873513	0,961873513	HIF1A/PSMB8/PSME2/PSMB10/EGR1/PSME1/EPO	7
GO:0097327	response to antineoplastic agent	7/182	13/749	0,019877161	0,961873513	0,961873513	MAPK13/ICAM1/TGFB1/EGR1/ABCC2/ERRFI1/EPO	7
GO:0044839	cell cycle G2/M phase transition	6/182	10/749	0,016480038	0,961873513	0,961873513	PSMB8/ABCB1/PSME2/PSMB10/CDKN1A/PSME1	6
GO:0070102	interleukin-6-mediated signaling pathway	6/182	10/749	0,016480038	0,961873513	0,961873513	JAK1/STAT3/SOCS3/STAT1/IL6R/IL6ST	6
GO:1901532	regulation of hematopoietic progenitor cell differentiation	6/182	10/749	0,016480038	0,961873513	0,961873513	PSMB8/PSME2/PSMB10/KITLG/PSME1/GATA3	6
GO:0051649	establishment of localization in cell	52/182	174/749	0,032873609	0,96315198	0,96315198	SERPINA3/S100A9/HIF1A/LTF/FPR1/STAT3/S100A8/C3/CD24/SLPI/SERPING1/FCER1G/FCGR3B/EHD3/MME/LCN2	52
GO:1901698	response to nitrogen compound	40/182	126/749	0,023337636	0,96315198	0,96315198	TIMP1/STAT3/COL4A1/NNMT/FCER1G/SOCS3/MAPK13/COL1A1/NR4A1/COL3A1/ICAM1/JAK3/STAT1/TGFB1/SOCS1/XBI	40
GO:0002274	myeloid leukocyte activation	38/182	121/749	0,032458947	0,96315198	0,96315198	SERPINA3/S100A9/LTF/FPR1/S100A8/C3/SLPI/FCER1G/FCGR3B/MME/LCN2/LTBR/IMPDH1/C1QA/RELB/IL4R/TGFB1/ALC	38
GO:0001959	regulation of cytokine-mediated signaling pathway	18/182	48/749	0,024545715	0,96315198	0,96315198	HIF1A/JAK1/CD24/IFNGR2/IL1R1/SOCS3/TNFRSF1A/IFNAR2/STAT1/SOCS1/SIGIRR/IRF7/IL1R2/TBK1/BIRC3/CASP4/IFNGF	18
GO:1901653	cellular response to peptide	17/182	46/749	0,033212137	0,96315198	0,96315198	STAT3/SOCS3/NR4A1/ICAM1/JAK3/STAT1/TGFB1/SOCS1/XBP1/VCAM1/STAT6/AGT/TLR4/ERRFI1/GDF15/CASP4/NFKB1	17
GO:0016567	protein ubiquitination	15/182	38/749	0,024219084	0,96315198	0,96315198	HIF1A/PSMB8/SOCS3/ASB15/HDAC6/PSME2/PSMB10/SOCS1/FKBP1A/BIRC3/PSME1/DCAF12/KLHL13/ISG15/RNF149	15
GO:0032479	regulation of type I interferon production	13/182	32/749	0,027517007	0,96315198	0,96315198	RELB/STAT1/STAT6/NFKB2/TLR4/MYD88/TLR2/IRF7/TBK1/LILRB1/CD14/NFKB1/ISG15	13
GO:0032606	type I interferon production	13/182	32/749	0,027517007	0,96315198	0,96315198	RELB/STAT1/STAT6/NFKB2/TLR4/MYD88/TLR2/IRF7/TBK1/LILRB1/CD14/NFKB1/ISG15	13
GO:0008202	steroid metabolic process	11/182	26/749	0,030556761	0,96315198	0,96315198	HSD11B1/AGT/APOL1/LDLR/EGR1/APOE/RORC/APOL2/TIPARP/G6PD/NFKB1	11
GO:0032481	positive regulation of type I interferon production	10/182	23/749	0,031765275	0,96315198	0,96315198	STAT1/STAT6/NFKB2/TLR4/MYD88/TLR2/IRF7/TBK1/CD14/NFKB1	10
GO:0032868	response to insulin	10/182	23/749	0,031765275	0,96315198	0,96315198	SOCS3/ICAM1/STAT1/SOCS1/XBP1/AGT/EGR1/TLR2/TNFSF10/ERRFI1	10

GO:1901987	regulation of cell cycle phase transition	10/182	23/749	0,031765275	0,96315198	0,96315198	ADAMTS1/PSMB8/MUC1/PSME2/PSMB10/TGFB1/BAX/CDKN1A/PSME1/PTPN6	10
GO:1901990	regulation of mitotic cell cycle phase transition	10/182	23/749	0,031765275	0,96315198	0,96315198	ADAMTS1/PSMB8/MUC1/PSME2/PSMB10/TGFB1/BAX/CDKN1A/PSME1/PTPN6	10
GO:0045444	fat cell differentiation	9/182	19/749	0,022553002	0,96315198	0,96315198	PSMB8/HDAC6/NR4A1/TGFB1/SOCS1/XBP1/RORC/CMKLR1/GATA3	9
GO:0071478	cellular response to radiation	9/182	19/749	0,022553002	0,96315198	0,96315198	MAPK13/MME/TGFB1/BAX/CDKN1A/EGR1/MYC/NOX4/GATA3	9
GO:0048525	negative regulation of viral process	9/182	20/749	0,032560176	0,96315198	0,96315198	LTF/IFITM3/SLPI/IFITM2/IFITM1/STAT1/ISG20/IFIT1/ISG15	9
GO:0006953	acute-phase response	8/182	16/749	0,021741599	0,96315198	0,96315198	SERPINA3/CD163/STAT3/SIGIRR/IL6R/APOL2/FN1/EPO	8
GO:0045471	response to ethanol	8/182	16/749	0,021741599	0,96315198	0,96315198	STAT3/S100A8/ICAM1/VCAM1/TNC/G6PD/CD14/GATA3	8
GO:1903901	negative regulation of viral life cycle	8/182	16/749	0,021741599	0,96315198	0,96315198	LTF/IFITM3/SLPI/IFITM2/IFITM1/ISG20/IFIT1/ISG15	8
GO:0006401	RNA catabolic process	8/182	17/749	0,032681963	0,96315198	0,96315198	PSMB8/PSME2/PSMB10/MYD88/SLC11A1/TNFRSF1B/ISG20/PSME1	8
GO:0071466	cellular response to xenobiotic stimulus	8/182	17/749	0,032681963	0,96315198	0,96315198	ICAM1/TGFB1/AHR/EGR1/RORC/S100A12/ABCC2/ERRFI1	8
GO:0014002	astrocyte development	7/182	14/749	0,031723183	0,96315198	0,96315198	S100A8/C1QA/LDLR/TLR4/C5AR1/IFNGR1/ADORA2A	7
GO:0061515	myeloid cell development	7/182	14/749	0,031723183	0,96315198	0,96315198	LTF/BCL6/TLR2/G6PD/LILRB1/EPO/PTPN6	7
GO:0090184	positive regulation of kidney development	7/182	14/749	0,031723183	0,96315198	0,96315198	CD24/TGFB1/AGT/EGR1/IL6R/MYC/GATA3	7
GO:0043488	regulation of mRNA stability	6/182	11/749	0,029030093	0,96315198	0,96315198	PSMB8/PSME2/PSMB10/MYD88/SLC11A1/PSME1	6
GO:0050832	defense response to fungus	6/182	11/749	0,029030093	0,96315198	0,96315198	S100A9/LTF/S100A8/TGFB1/IL17RA/S100A12	6
GO:0055094	response to lipoprotein particle	6/182	11/749	0,029030093	0,96315198	0,96315198	FCER1G/LDLR/TLR4/MYD88/APOE/ITGB2	6
GO:0060330	regulation of response to interferon-gamma	6/182	11/749	0,029030093	0,96315198	0,96315198	JAK1/IFNGR2/SOCS3/STAT1/SOCS1/IFNGR1	6
GO:0060334	regulation of interferon-gamma-mediated signaling pathway	6/182	11/749	0,029030093	0,96315198	0,96315198	JAK1/IFNGR2/SOCS3/STAT1/SOCS1/IFNGR1	6
GO:0071402	cellular response to lipoprotein particle stimulus	6/182	11/749	0,029030093	0,96315198	0,96315198	FCER1G/LDLR/TLR4/MYD88/APOE/ITGB2	6
GO:0051172	negative regulation of nitrogen compound metabolic process	56/182	190/749	0,035126238	0,981771842	0,981771842	SERPINA3/LTF/BCL3/RARRES1/BCL6/TIMP1/STAT3/C3/SLPI/SERPING1/SOCS3/MUC1/HDAC6/MAF/RELB/JAK3/STAT1/TG	56
GO:0007568	aging	19/182	53/749	0,034404931	0,981771842	0,981771842	SOD2/BCL6/TIMP1/STAT3/SERPING1/MME/ICAM1/C1QA/TGFB1/CDKN1A/VCAM1/AGT/NFKB2/TNFRSF1B/ITGB2/NOX4/	19
GO:0034340	response to type I interferon	16/182	43/749	0,036081452	0,981771842	0,981771842	JAK1/IFITM3/IFITM2/IFITM1/PSMB8/GBP2/IFNAR2/STAT1/EGR1/MYD88/IRF7/TBK1/ISG20/IFIT1/ISG15/PTPN6	16
GO:0060337	type I interferon signaling pathway	16/182	43/749	0,036081452	0,981771842	0,981771842	JAK1/IFITM3/IFITM2/IFITM1/PSMB8/GBP2/IFNAR2/STAT1/EGR1/MYD88/IRF7/TBK1/ISG20/IFIT1/ISG15/PTPN6	16
GO:0071357	cellular response to type I interferon	16/182	43/749	0,036081452	0,981771842	0,981771842	JAK1/IFITM3/IFITM2/IFITM1/PSMB8/GBP2/IFNAR2/STAT1/EGR1/MYD88/IRF7/TBK1/ISG20/IFIT1/ISG15/PTPN6	16
GO:0006950	response to stress	130/182	495/749	0,047547896	0,999954951	0,999954951	SERPINA3/OSMR/S100A9/HIF1A/LTF/BCL3/C1QB/JAK1/CD163/IFITM3/C1S/FPR1/SOD2/BCL6/TIMP1/STAT3/S100A8/C3/	130
GO:0042221	response to chemical	127/182	493/749	0,113736612	0,999954951	0,999954951	OSMR/S100A9/HIF1A/LTF/JAK1/IFITM3/FPR1/SOD2/BCL6/TIMP1/STAT3/S100A8/CD24/COL4A1/SLPI/IFITM2/NNMT/IFIT	127
GO:0044238	primary metabolic process	121/182	500/749	0,573378179	0,999954951	0,999954951	SERPINA3/ALDH3A2/S100A9/HIF1A/LTF/BCL3/PLAAT4/RARRES1/JAK1/C1S/FPR1/SOD2/BCL6/TIMP1/STAT3/S100A8/C3/	121
GO:0048583	regulation of response to stimulus	119/182	490/749	0,542186879	0,999954951	0,999954951	OSMR/S100A9/HIF1A/LTF/BCL3/C1QB/JAK1/C1S/FPR1/SOD2/BCL6/TIMP1/STAT3/S100A8/C3/CD24/IFITM1/SERPING1/(	119
GO:0010033	response to organic substance	117/182	453/749	0,131237423	0,999954951	0,999954951	OSMR/HIF1A/LTF/JAK1/IFITM3/FPR1/SOD2/BCL6/TIMP1/STAT3/S100A8/CD24/COL4A1/SLPI/IFITM2/NNMT/IFITM1/FCE	117
GO:0070887	cellular response to chemical stimulus	116/182	448/749	0,124054102	0,999954951	0,999954951	OSMR/S100A9/HIF1A/LTF/JAK1/IFITM3/FPR1/SOD2/BCL6/TIMP1/STAT3/S100A8/CD24/COL4A1/IFITM2/IFITM1/FCER1G	116
GO:0019222	regulation of metabolic process	111/182	468/749	0,715315845	0,999954951	0,999954951	SERPINA3/S100A9/HIF1A/LTF/BCL3/RARRES1/FPR1/SOD2/BCL6/TIMP1/STAT3/S100A8/C3/CD24/ITGB6/SLPI/SERPING1/	111
GO:0009605	response to external stimulus	110/182	441/749	0,343461212	0,999954951	0,999954951	OSMR/S100A9/LTF/BCL3/C1QB/JAK1/IFITM3/C1S/FPR1/BCL6/S100A8/C3/CD24/SLPI/IFITM2/IFITM1/SERPING1/C9/CFB/	110
GO:0006955	immune response	110/182	460/749	0,655813225	0,999954951	0,999954951	SERPINA3/S100A9/LTF/BCL3/C1QB/JAK1/IFITM3/C1S/FPR1/BCL6/STAT3/S100A8/C3/CD24/SLPI/IFITM2/IFITM1/SERPINC	110
GO:0032502	developmental process	110/182	462/749	0,686708396	0,999954951	0,999954951	ALDH3A2/S100A9/HIF1A/LTF/BCL3/PLAAT4/C1QB/JAK1/SOD2/BCL6/TIMP1/STAT3/S100A8/C3/CD24/COL4A1/ADAMTS1	110
GO:0060255	regulation of macromolecule metabolic process	109/182	456/749	0,657191069	0,999954951	0,999954951	SERPINA3/S100A9/HIF1A/LTF/BCL3/RARRES1/FPR1/SOD2/BCL6/TIMP1/STAT3/S100A8/C3/CD24/ITGB6/SLPI/SERPING1/	109
GO:0007166	cell surface receptor signaling pathway	109/182	465/749	0,785187296	0,999954951	0,999954951	OSMR/HIF1A/LTF/BCL3/JAK1/IFITM3/FPR1/SOD2/BCL6/TIMP1/STAT3/C3/CD24/COL4A1/ITGB6/ADAMTS1/IFITM2/IFITM	109
GO:1901564	organonitrogen compound metabolic process	107/182	418/749	0,199007491	0,999954951	0,999954951	SERPINA3/ALDH3A2/S100A9/HIF1A/LTF/BCL3/PLAAT4/RARRES1/JAK1/C1S/FPR1/BCL6/TIMP1/STAT3/S100A8/C3/CD24/	107
GO:0044260	cellular macromolecule metabolic process	107/182	446/749	0,628440726	0,999954951	0,999954951	SERPINA3/S100A9/HIF1A/LTF/BCL3/RARRES1/JAK1/FPR1/SOD2/BCL6/TIMP1/STAT3/S100A8/C3/CD24/SLPI/SERPING1/F	107
GO:0048856	anatomical structure development	106/182	444/749	0,661386407	0,999954951	0,999954951	ALDH3A2/S100A9/HIF1A/LTF/BCL3/PLAAT4/C1QB/JAK1/SOD2/BCL6/TIMP1/STAT3/S100A8/C3/CD24/COL4A1/ADAMTS1	106
GO:0048519	negative regulation of biological process	105/182	423/749	0,384811379	0,999954951	0,999954951	SERPINA3/HIF1A/LTF/BCL3/PLAAT4/RARRES1/IFITM3/SOD2/BCL6/TIMP1/STAT3/C3/CD24/SLPI/ADAMTS1/IFITM2/IFITM	105
GO:0051179	localization	105/182	448/749	0,776002904	0,999954951	0,999954951	SERPINA3/S100A9/HIF1A/LTF/BCL3/CD163/FPR1/SOD2/BCL6/TIMP1/STAT3/S100A8/C3/CD24/ITGB6/SLPI/ADAMTS1/IFI	105
GO:0071310	cellular response to organic substance	104/182	418/749	0,37090083	0,999954951	0,999954951	OSMR/HIF1A/LTF/JAK1/IFITM3/FPR1/SOD2/BCL6/TIMP1/STAT3/CD24/COL4A1/IFITM2/IFITM1/FCER1G/IFNGR2/SOST/P:	104
GO:0006952	defense response	103/182	386/749	0,068762447	0,999954951	0,999954951	SERPINA3/OSMR/S100A9/HIF1A/LTF/BCL3/C1QB/JAK1/CD163/IFITM3/C1S/FPR1/BCL6/TIMP1/STAT3/S100A8/C3/ITGB6,	103
GO:0007275	multicellular organism development	102/182	425/749	0,620302878	0,999954951	0,999954951	ALDH3A2/S100A9/HIF1A/LTF/BCL3/PLAAT4/C1QB/JAK1/SOD2/BCL6/TIMP1/STAT3/S100A8/C3/CD24/COL4A1/ADAMTS1	102
GO:0048731	system development	100/182	411/749	0,525758677	0,999954951	0,999954951	ALDH3A2/S100A9/HIF1A/LTF/BCL3/PLAAT4/C1QB/JAK1/SOD2/BCL6/TIMP1/STAT3/S100A8/C3/CD24/COL4A1/ADAMTS1	100
GO:0019538	protein metabolic process	99/182	386/749	0,211298299	0,999954951	0,999954951	SERPINA3/S100A9/HIF1A/LTF/BCL3/RARRES1/JAK1/C1S/FPR1/BCL6/TIMP1/STAT3/S100A8/C3/CD24/SLPI/ADAMTS1/SEF	99
GO:0080090	regulation of primary metabolic process	97/182	404/749	0,612608747	0,999954951	0,999954951	SERPINA3/S100A9/HIF1A/LTF/BCL3/RARRES1/FPR1/SOD2/BCL6/TIMP1/STAT3/S100A8/C3/CD24/SLPI/SERPING1/SOST/F	97
GO:0044419	interspecies interaction between organisms	96/182	383/749	0,339230369	0,999954951	0,999954951	S100A9/LTF/BCL3/C1QB/JAK1/IFITM3/C1S/STAT3/S100A8/C3/CD24/ITGB6/SLPI/IFITM2/IFITM1/SERPING1/C9/HAVCR1/(	96
GO:0051171	regulation of nitrogen compound metabolic process	96/182	400/749	0,614298524	0,999954951	0,999954951	SERPINA3/S100A9/HIF1A/LTF/BCL3/RARRES1/FPR1/SOD2/BCL6/TIMP1/STAT3/S100A8/C3/CD24/SLPI/SERPING1/SOST/F	96
GO:0031323	regulation of cellular metabolic process	96/182	412/749	0,785325698	0,999954951	0,999954951	SERPINA3/S100A9/HIF1A/LTF/BCL3/RARRES1/FPR1/SOD2/BCL6/TIMP1/STAT3/S100A8/C3/CD24/SLPI/SERPING1/SOST/F	96
GO:0051239	regulation of multicellular organismal process	95/182	376/749	0,296630357	0,999954951	0,999954951	S100A9/HIF1A/LTF/BCL3/PLAAT4/JAK1/SOD2/BCL6/TIMP1/STAT3/C3/CD24/ITGB6/IFITM1/SERPING1/FCER1G/SOST/PSN	95
GO:0048523	negative regulation of cellular process	94/182	386/749	0,520185877	0,999954951	0,999954951	SERPINA3/HIF1A/LTF/BCL3/PLAAT4/RARRES1/SOD2/BCL6/TIMP1/STAT3/C3/SLPI/ADAMTS1/IFITM1/SERPING1/FCER1G/	94
GO:0010467	gene expression	93/182	393/749	0,695474483	0,999954951	0,999954951	S100A9/HIF1A/LTF/BCL3/SOD2/BCL6/STAT3/S100A8/C3/CD24/ITGB6/FCER1G/SOST/PSMB8/IL1R1/MUC1/MAPK13/TNF	93
GO:0030154	cell differentiation	89/182	355/749	0,351057487	0,999954951	0,999954951	S100A9/HIF1A/LTF/BCL3/PLAAT4/SOD2/BCL6/STAT3/S100A8/C3/CD24/COL4A1/IFITM1/FCER1G/PSMB8/SOCS3/TNFRSF	89
GO:0048869	cellular developmental process	89/182	359/749	0,414306521	0,999954951	0,999954951	S100A9/HIF1A/LTF/BCL3/PLAAT4/SOD2/BCL6/STAT3/S100A8/C3/CD24/COL4A1/IFITM1/FCER1G/PSMB8/SOCS3/TNFRSF	89
GO:0023051	regulation of signaling	89/182	369/749	0,578485207	0,999954951	0,999954951	S100A9/HIF1A/LTF/BCL3/JAK1/FPR1/SOD2/BCL6/TIMP1/STAT3/S100A8/C3/CD24/IFNGR2/SOST/PSMB8/IL1R1/SOCS3/M	89
GO:0009893	positive regulation of metabolic process	89/182	395/749	0,899087023	0,999954951	0,999954951	S100A9/HIF1A/LTF/BCL3/FPR1/BCL6/STAT3/S100A8/C3/CD24/FCER1G/SOST/IL1R1/SOCS3/MUC1/MAPK13/TNFRSF1A/L	89
GO:0010646	regulation of cell communication	88/182	368/749	0,628160744	0,999954951	0,999954951	S100A9/HIF1A/LTF/BCL3/JAK1/FPR1/SOD2/BCL6/TIMP1/STAT3/S100A8/C3/CD24/IFNGR2/SOST/PSMB8/IL1R1/SOCS3/M	88
GO:0044267	cellular protein metabolic process	87/182	347/749	0,354321879	0,999954951	0,999954951	SERPINA3/S100A9/HIF1A/LTF/BCL3/RARRES1/JAK1/FPR1/BCL6/TIMP1/STAT3/S100A8/C3/CD24/SLPI/SERPING1/PSMB8/	87

GO:0010468	regulation of gene expression	87/182	371/749	0,732924146	0,999954951	0,999954951	S100A9/HIF1A/LTF/BCL3/SOD2/BCL6/STAT3/S100A8/C3/CD24/ITGB6/FCER1G/SOST/PSMB8/IL1R1/MUC1/MAPK13/TNF	87
GO:0010604	positive regulation of macromolecule metabolic process	87/182	381/749	0,849905978	0,999954951	0,999954951	S100A9/HIF1A/LTF/BCL3/FPR1/BCL6/STAT3/S100A8/C3/CD24/FCER1G/SOST/IL1R1/SOCS3/MUC1/MAPK13/TNFRSF1A/L	87
GO:0034097	response to cytokine	86/182	328/749	0,159667169	0,999954951	0,999954951	OSMR/HIF1A/JAK1/IFITM3/FPR1/SOD2/BCL6/TIMP1/STAT3/CD24/IFITM2/IFITM1/FCER1G/IFNGR2/PSMB8/IL1R1/SOCS3	86
GO:0006810	transport	86/182	349/749	0,452295197	0,999954951	0,999954951	SERPINA3/S100A9/HIF1A/LTF/CD163/FPR1/TIMP1/STAT3/S100A8/C3/CD24/ITGB6/SLPI/SERPING1/FCER1G/PSMB8/TNF	86
GO:0051234	establishment of localization	86/182	351/749	0,485318297	0,999954951	0,999954951	SERPINA3/S100A9/HIF1A/LTF/CD163/FPR1/TIMP1/STAT3/S100A8/C3/CD24/ITGB6/SLPI/SERPING1/FCER1G/PSMB8/TNF	86
GO:0048584	positive regulation of response to stimulus	86/182	387/749	0,927218995	0,999954951	0,999954951	OSMR/S100A9/HIF1A/LTF/C1QB/C1S/FPR1/STAT3/S100A8/C3/CD24/SERPING1/C9/CFB/FCER1G/PSMB8/IL1R1/SOCS3/N	86
GO:0043207	response to external biotic stimulus	84/182	337/749	0,3907474	0,999954951	0,999954951	S100A9/LTF/BCL3/C1QB/JAK1/IFITM3/C1S/S100A8/C3/CD24/SLPI/IFITM2/IFITM1/SERPING1/C9/CFB/FCER1G/IFNGR2/P	84
GO:0051707	response to other organism	84/182	337/749	0,3907474	0,999954951	0,999954951	S100A9/LTF/BCL3/C1QB/JAK1/IFITM3/C1S/S100A8/C3/CD24/SLPI/IFITM2/IFITM1/SERPING1/C9/CFB/FCER1G/IFNGR2/P	84
GO:0009607	response to biotic stimulus	84/182	338/749	0,406832719	0,999954951	0,999954951	S100A9/LTF/BCL3/C1QB/JAK1/IFITM3/C1S/S100A8/C3/CD24/SLPI/IFITM2/IFITM1/SERPING1/C9/CFB/FCER1G/IFNGR2/P	84
GO:0002682	regulation of immune system process	83/182	362/749	0,824122632	0,999954951	0,999954951	HIF1A/LTF/C1QB/JAK1/C1S/FPR1/BCL6/STAT3/C3/CD24/IFITM1/SERPING1/C9/CFB/FCER1G/IFNGR2/PSMB8/IL1R1/SOCS	83
GO:0071840	cellular component organization or biogenesis	81/182	331/749	0,494521364	0,999954951	0,999954951	S100A9/HIF1A/BCL3/C1QB/SOD2/BCL6/TIMP1/STAT3/S100A8/C3/CD24/COL4A1/ITGB6/C9/FCER1G/SOST/MUC1/TNFRS	81
GO:0071345	cellular response to cytokine stimulus	80/182	314/749	0,289713231	0,999954951	0,999954951	OSMR/HIF1A/JAK1/IFITM3/FPR1/SOD2/BCL6/TIMP1/STAT3/CD24/IFITM2/IFITM1/FCER1G/IFNGR2/PSMB8/IL1R1/SOCS3	80
GO:0016043	cellular component organization	80/182	329/749	0,529654509	0,999954951	0,999954951	S100A9/HIF1A/BCL3/C1QB/SOD2/BCL6/TIMP1/STAT3/S100A8/C3/CD24/COL4A1/ITGB6/C9/FCER1G/SOST/MUC1/TNFRS	80
GO:0009966	regulation of signal transduction	80/182	334/749	0,611329223	0,999954951	0,999954951	S100A9/HIF1A/LTF/BCL3/JAK1/FPR1/SOD2/BCL6/TIMP1/STAT3/S100A8/C3/CD24/IFNGR2/SOST/PSMB8/IL1R1/SOCS3/N	80
GO:0048513	animal organ development	79/182	341/749	0,771918264	0,999954951	0,999954951	HIF1A/LTF/BCL3/PLAAT4/C1QB/BCL6/TIMP1/STAT3/CD24/COL4A1/ADAMTS1/NNMT/FCER1G/PSMB8/SOCS3/TNFRSF1A	79
GO:0065008	regulation of biological quality	78/182	304/749	0,263854654	0,999954951	0,999954951	SERPINA3/S100A9/HIF1A/LTF/BCL3/FPR1/SOD2/BCL6/STAT3/S100A8/C3/CD24/SERPING1/FCER1G/PSMB8/EHD3/ABCB1	78
GO:0051246	regulation of protein metabolic process	77/182	293/749	0,177135497	0,999954951	0,999954951	SERPINA3/S100A9/LTF/BCL3/RARRES1/FPR1/BCL6/TIMP1/STAT3/S100A8/C3/CD24/SLPI/SERPING1/PSMB8/SOCS3/MUC	77
GO:0051173	positive regulation of nitrogen compound metabolic process	76/182	329/749	0,776972396	0,999954951	0,999954951	S100A9/HIF1A/LTF/BCL3/FPR1/BCL6/STAT3/S100A8/C3/CD24/SOST/SOCS3/MUC1/TNFRSF1A/LTBR/HDAC6/COL1A1/NR	76
GO:0001775	cell activation	76/182	333/749	0,823365352	0,999954951	0,999954951	SERPINA3/S100A9/LTF/BCL3/FPR1/BCL6/TIMP1/STAT3/S100A8/C3/CD24/SLPI/FCER1G/FCGR3B/MME/LCN2/LTBR/COL1	76
GO:0009058	biosynthetic process	75/182	327/749	0,802636866	0,999954951	0,999954951	ALDH3A2/S100A9/HIF1A/LTF/BCL3/SOD2/BCL6/STAT3/S100A8/C3/NNMT/SOST/PSMB8/MUC1/TNFRSF1A/HDAC6/COL1	75
GO:0031325	positive regulation of cellular metabolic process	75/182	338/749	0,904514053	0,999954951	0,999954951	S100A9/HIF1A/LTF/BCL3/FPR1/BCL6/STAT3/S100A8/C3/CD24/SOST/SOCS3/MUC1/TNFRSF1A/LTBR/HDAC6/COL1A1/NR	75
GO:1901576	organic substance biosynthetic process	74/182	319/749	0,755007542	0,999954951	0,999954951	ALDH3A2/S100A9/HIF1A/LTF/BCL3/SOD2/BCL6/STAT3/S100A8/C3/NNMT/SOST/PSMB8/MUC1/TNFRSF1A/HDAC6/COL1	74
GO:0044249	cellular biosynthetic process	73/182	321/749	0,82808039	0,999954951	0,999954951	ALDH3A2/S100A9/HIF1A/LTF/BCL3/SOD2/BCL6/STAT3/S100A8/C3/NNMT/SOST/PSMB8/MUC1/TNFRSF1A/HDAC6/COL1	73
GO:0006464	cellular protein modification process	72/182	292/749	0,460667862	0,999954951	0,999954951	S100A9/HIF1A/LTF/JAK1/FPR1/BCL6/TIMP1/STAT3/S100A8/C3/CD24/PSMB8/SOCS3/MUC1/MAPK13/TNFRSF1A/ASB15/	72
GO:0036211	protein modification process	72/182	292/749	0,460667862	0,999954951	0,999954951	S100A9/HIF1A/LTF/JAK1/FPR1/BCL6/TIMP1/STAT3/S100A8/C3/CD24/PSMB8/SOCS3/MUC1/MAPK13/TNFRSF1A/ASB15/	72
GO:0043412	macromolecule modification	72/182	295/749	0,511403764	0,999954951	0,999954951	S100A9/HIF1A/LTF/JAK1/FPR1/BCL6/TIMP1/STAT3/S100A8/C3/CD24/PSMB8/SOCS3/MUC1/MAPK13/TNFRSF1A/ASB15/	72
GO:0050793	regulation of developmental process	72/182	297/749	0,545098555	0,999954951	0,999954951	S100A9/HIF1A/LTF/PLAAT4/JAK1/SOD2/BCL6/TIMP1/STAT3/C3/CD24/IFITM1/PSMB8/SOCS3/TNFRSF1A/MME/HDAC6/C	72
GO:1901360	organic cyclic compound metabolic process	72/182	297/749	0,545098555	0,999954951	0,999954951	S100A9/HIF1A/LTF/BCL3/SOD2/BCL6/STAT3/S100A8/NNMT/SOST/PSMB8/MUC1/TNFRSF1A/MME/HDAC6/COL1A1/NR4	72
GO:0035556	intracellular signal transduction	72/182	307/749	0,703555228	0,999954951	0,999954951	S100A9/HIF1A/LTF/BCL3/JAK1/FPR1/SOD2/BCL6/S100A8/CD24/PSMB8/SOCS3/MUC1/MAPK13/TNFRSF1A/ASB15/LTBR/	72
GO:0098542	defense response to other organism	71/182	268/749	0,169463214	0,999954951	0,999954951	S100A9/LTF/BCL3/C1QB/JAK1/IFITM3/C1S/S100A8/C3/SLPI/IFITM2/IFITM1/SERPING1/C9/CFB/FCER1G/IFNGR2/PSMB8/	71
GO:0019221	cytokine-mediated signaling pathway	71/182	275/749	0,257135263	0,999954951	0,999954951	OSMR/HIF1A/JAK1/IFITM3/FPR1/SOD2/BCL6/TIMP1/STAT3/CD24/IFITM2/IFITM1/FCER1G/IFNGR2/PSMB8/IL1R1/SOCS3	71
GO:0065009	regulation of molecular function	71/182	275/749	0,257135263	0,999954951	0,999954951	SERPINA3/S100A9/HIF1A/LTF/BCL3/PLAAT4/RARRES1/FPR1/BCL6/TIMP1/STAT3/S100A8/C3/CD24/SLPI/SERPING1/PSMI	71
GO:0002252	immune effector process	69/182	261/749	0,181677001	0,999954951	0,999954951	SERPINA3/S100A9/LTF/BCL3/C1QB/IFITM3/C1S/FPR1/BCL6/STAT3/S100A8/C3/SLPI/IFITM2/IFITM1/SERPING1/C9/CFB/F	69
GO:0032268	regulation of cellular protein metabolic process	69/182	268/749	0,273215707	0,999954951	0,999954951	SERPINA3/S100A9/LTF/BCL3/RARRES1/FPR1/BCL6/TIMP1/STAT3/S100A8/C3/CD24/SLPI/SERPING1/SOCS3/MUC1/TNFR	69
GO:0034641	cellular nitrogen compound metabolic process	69/182	305/749	0,83472259	0,999954951	0,999954951	S100A9/HIF1A/LTF/BCL3/PLAAT4/SOD2/BCL6/STAT3/S100A8/NNMT/SOST/PSMB8/MUC1/TNFRSF1A/MME/HDAC6/COL	69
GO:0045321	leukocyte activation	69/182	306/749	0,844915592	0,999954951	0,999954951	SERPINA3/S100A9/LTF/BCL3/FPR1/BCL6/STAT3/S100A8/C3/CD24/SLPI/FCER1G/FCGR3B/MME/LCN2/LTBR/ICAM1/IMPD	69
GO:0009892	negative regulation of metabolic process	67/182	259/749	0,260650843	0,999954951	0,999954951	SERPINA3/HIF1A/LTF/BCL3/RARRES1/BCL6/TIMP1/STAT3/C3/CD24/SLPI/SERPING1/PSMB8/SOCS3/MUC1/HDAC6/PSME	67
GO:0051240	positive regulation of multicellular organismal process	67/182	286/749	0,699356161	0,999954951	0,999954951	S100A9/HIF1A/LTF/BCL3/PLAAT4/JAK1/SOD2/BCL6/STAT3/C3/CD24/IFITM1/FCER1G/IL1R1/MAPK13/MME/LCN2/HDAC	67
GO:0050776	regulation of immune response	66/182	269/749	0,488689741	0,999954951	0,999954951	C1QB/JAK1/C1S/FPR1/BCL6/C3/CD24/IFITM1/SERPING1/C9/CFB/FCER1G/IFNGR2/PSMB8/IL1R1/SOCS3/MUC1/IFNAR2/	66
GO:0046483	heterocycle metabolic process	66/182	284/749	0,73033796	0,999954951	0,999954951	S100A9/HIF1A/LTF/BCL3/SOD2/BCL6/STAT3/S100A8/NNMT/SOST/PSMB8/MUC1/TNFRSF1A/MME/HDAC6/COL1A1/NR4	66
GO:0010605	negative regulation of macromolecule metabolic process	65/182	248/749	0,220846053	0,999954951	0,999954951	SERPINA3/HIF1A/LTF/BCL3/RARRES1/BCL6/TIMP1/STAT3/C3/CD24/SLPI/SERPING1/PSMB8/SOCS3/MUC1/HDAC6/PSME	65
GO:1901362	organic cyclic compound biosynthetic process	65/182	262/749	0,438850418	0,999954951	0,999954951	S100A9/HIF1A/LTF/BCL3/SOD2/BCL6/STAT3/S100A8/NNMT/SOST/PSMB8/MUC1/TNFRSF1A/HDAC6/COL1A1/NR4A1/PS	65
GO:0008219	cell death	65/182	266/749	0,507853042	0,999954951	0,999954951	S100A9/HIF1A/LTF/BCL3/SOD2/BCL6/TIMP1/STAT3/S100A8/CD24/FCER1G/SOCS3/MUC1/TNFRSF1A/LCN2/KRT8/LTBR/I	65
GO:0009059	macromolecule biosynthetic process	65/182	288/749	0,831377591	0,999954951	0,999954951	S100A9/HIF1A/LTF/BCL3/SOD2/BCL6/STAT3/S100A8/SOST/PSMB8/MUC1/TNFRSF1A/HDAC6/COL1A1/NR4A1/PSME2/IC	65
GO:0006725	cellular aromatic compound metabolic process	64/182	285/749	0,8436254	0,999954951	0,999954951	S100A9/HIF1A/LTF/BCL3/SOD2/BCL6/STAT3/S100A8/NNMT/SOST/PSMB8/MUC1/TNFRSF1A/HDAC6/COL1A1/NR4A1/PS	64
GO:0045087	innate immune response	63/182	229/749	0,103062112	0,999954951	0,999954951	S100A9/LTF/C1QB/JAK1/IFITM3/C1S/S100A8/C3/SLPI/IFITM2/IFITM1/SERPING1/C9/CFB/FCER1G/IFNGR2/PSMB8/SOCS	63
GO:0080134	regulation of response to stress	63/182	231/749	0,120424069	0,999954951	0,999954951	OSMR/S100A9/HIF1A/JAK1/SOD2/BCL6/S100A8/C3/SERPING1/FCER1G/IFNGR2/PSMB8/IL1R1/SOCS3/MUC1/MAPK13/T	63
GO:0018130	heterocycle biosynthetic process	63/182	258/749	0,511786452	0,999954951	0,999954951	S100A9/HIF1A/LTF/BCL3/SOD2/BCL6/STAT3/S100A8/NNMT/SOST/PSMB8/MUC1/TNFRSF1A/HDAC6/COL1A1/NR4A1/PS	63
GO:0019438	aromatic compound biosynthetic process	63/182	258/749	0,511786452	0,999954951	0,999954951	S100A9/HIF1A/LTF/BCL3/SOD2/BCL6/STAT3/S100A8/NNMT/SOST/PSMB8/MUC1/TNFRSF1A/HDAC6/COL1A1/NR4A1/PS	63
GO:0044271	cellular nitrogen compound biosynthetic process	63/182	278/749	0,813247415	0,999954951	0,999954951	S100A9/HIF1A/LTF/BCL3/SOD2/BCL6/STAT3/S100A8/NNMT/SOST/PSMB8/MUC1/TNFRSF1A/HDAC6/COL1A1/NR4A1/PS	63
GO:0006139	nucleobase-containing compound metabolic process	63/182	279/749	0,82444055	0,999954951	0,999954951	S100A9/HIF1A/LTF/BCL3/SOD2/BCL6/STAT3/S100A8/NNMT/SOST/PSMB8/MUC1/TNFRSF1A/HDAC6/COL1A1/NR4A1/PS	63
GO:0031326	regulation of cellular biosynthetic process	63/182	281/749	0,845489543	0,999954951	0,999954951	S100A9/HIF1A/LTF/BCL3/SOD2/BCL6/STAT3/S100A8/C3/SOST/PSMB8/MUC1/TNFRSF1A/HDAC6/COL1A1/NR4A1/PSME	63
GO:0009889	regulation of biosynthetic process	63/182	284/749	0,873739607	0,999954951	0,999954951	S100A9/HIF1A/LTF/BCL3/SOD2/BCL6/STAT3/S100A8/C3/SOST/PSMB8/MUC1/TNFRSF1A/HDAC6/COL1A1/NR4A1/PSME	63
GO:0012501	programmed cell death	62/182	251/749	0,461374697	0,999954951	0,999954951	S100A9/HIF1A/LTF/BCL3/SOD2/BCL6/TIMP1/STAT3/S100A8/CD24/FCER1G/SOCS3/MUC1/TNFRSF1A/LCN2/KRT8/LTBR/I	62
GO:0034654	nucleobase-containing compound biosynthetic process	62/182	256/749	0,548506669	0,999954951	0,999954951	S100A9/HIF1A/LTF/BCL3/SOD2/BCL6/STAT3/S100A8/NNMT/SOST/PSMB8/MUC1/TNFRSF1A/HDAC6/COL1A1/NR4A1/PS	62
GO:0010647	positive regulation of cell communication	62/182	278/749	0,857180471	0,999954951	0,999954951	S100A9/HIF1A/LTF/FPR1/STAT3/S100A8/C3/CD24/PSMB8/IL1R1/SOCS3/TNFRSF1A/MME/LTBR/HDAC6/COL1A1/COL3A1	62
GO:0023056	positive regulation of signaling	62/182	278/749	0,857180471	0,999954951	0,999954951	S100A9/HIF1A/LTF/FPR1/STAT3/S100A8/C3/CD24/PSMB8/IL1R1/SOCS3/TNFRSF1A/MME/LTBR/HDAC6/COL1A1/COL3A1	62
GO:0006954	inflammatory response	61/182	217/749	0,073161474	0,999954951	0,999954951	SERPINA3/OSMR/S100A9/HIF1A/CD163/FPR1/BCL6/TIMP1/STAT3/S100A8/C3/ITGB6/FCER1G/IL1R1/SOCS3/MAPK13/TN	61
GO:0090304	nucleic acid metabolic process	61/182	266/749	0,768550762	0,999954951	0,999954951	S100A9/HIF1A/LTF/BCL3/SOD2/BCL6/STAT3/S100A8/SOST/PSMB8/MUC1/TNFRSF1A/HDAC6/COL1A1/NR4A1/PSME2/IC	61

GO:0010556	regulation of macromolecule biosynthetic process	61/182	268/749	0,79385475	0,999954951	0,999954951	S100A9/HIF1A/LTF/BCL3/SOD2/BCL6/STAT3/S100A8/SOST/PSMB8/MUC1/TNFRSF1A/HDAC6/COL1A1/NR4A1/PSME2/IC	61
GO:0034645	cellular macromolecule biosynthetic process	61/182	268/749	0,79385475	0,999954951	0,999954951	S100A9/HIF1A/LTF/BCL3/SOD2/BCL6/STAT3/S100A8/SOST/PSMB8/MUC1/TNFRSF1A/HDAC6/COL1A1/NR4A1/PSME2/IC	61
GO:0050790	regulation of catalytic activity	60/182	221/749	0,139558814	0,999954951	0,999954951	SERPINA3/S100A9/HIF1A/LTF/PLAAT4/RARRES1/FPR1/BCL6/TIMP1/STAT3/S100A8/C3/CD24/SLPI/SERPING1/PSMB8/SO	60
GO:0032101	regulation of response to external stimulus	60/182	226/749	0,196957968	0,999954951	0,999954951	OSMR/S100A9/LTF/JAK1/BCL6/S100A8/C3/SERPING1/FCER1G/IFNGR2/PSMB8/IL1R1/SOCS3/MUC1/MAPK13/TNFRSF1A	60
GO:0006915	apoptotic process	60/182	247/749	0,535341402	0,999954951	0,999954951	S100A9/HIF1A/LTF/BCL3/SOD2/BCL6/TIMP1/STAT3/S100A8/CD24/FCER1G/SOCS3/MUC1/TNFRSF1A/LCN2/KRT8/LTBR/I	60
GO:2000026	regulation of multicellular organismal development	60/182	258/749	0,715269694	0,999954951	0,999954951	S100A9/HIF1A/LTF/PLAAT4/JAK1/SOD2/BCL6/TIMP1/STAT3/C3/CD24/PSMB8/TNFRSF1A/MME/COL1A1/COL3A1/PSME2	60
GO:0006796	phosphate-containing compound metabolic process	60/182	272/749	0,878948422	0,999954951	0,999954951	HIF1A/LTF/PLAAT4/JAK1/FPR1/STAT3/C3/CD24/NNMT/PSMB8/SOCS3/MAPK13/TNFRSF1A/LTBR/HDAC6/PSME2/ICAM1	60
GO:0006793	phosphorus metabolic process	60/182	273/749	0,887298814	0,999954951	0,999954951	HIF1A/LTF/PLAAT4/JAK1/FPR1/STAT3/C3/CD24/NNMT/PSMB8/SOCS3/MAPK13/TNFRSF1A/LTBR/HDAC6/PSME2/ICAM1	60
GO:0032879	regulation of localization	60/182	301/749	0,991516148	0,999954951	0,999954951	HIF1A/SOD2/BCL6/TIMP1/STAT3/S100A8/C3/ADAMTS1/IFITM1/FCER1G/IL1R1/TNFRSF1A/EHD3/ABCB1/HDAC6/COL1A1	60
GO:1901700	response to oxygen-containing compound	59/182	225/749	0,23752965	0,999954951	0,999954951	HIF1A/LTF/SOD2/TIMP1/STAT3/S100A8/COL4A1/SLPI/SOCS3/MAPK13/LCN2/KRT8/HDAC6/COL1A1/NR4A1/COL3A1/ICA	59
GO:0001816	cytokine production	59/182	229/749	0,297264634	0,999954951	0,999954951	S100A9/HIF1A/LTF/BCL3/BCL6/STAT3/S100A8/C3/CD24/ITGB6/FCER1G/IL1R1/MAPK13/MAF/RELB/JAK3/STAT1/IL4R/TG	59
GO:0016070	RNA metabolic process	59/182	249/749	0,639790384	0,999954951	0,999954951	S100A9/HIF1A/LTF/BCL3/SOD2/BCL6/STAT3/S100A8/SOST/PSMB8/MUC1/TNFRSF1A/HDAC6/COL1A1/NR4A1/PSME2/IC	59
GO:0008283	cell population proliferation	59/182	282/749	0,961775555	0,999954951	0,999954951	OSMR/HIF1A/LTF/PLAAT4/RARRES1/SOD2/BCL6/TIMP1/STAT3/CD24/ADAMTS1/IFITM1/ABCB1/NR4A1/IMPDH1/PSMB1	59
GO:0045595	regulation of cell differentiation	58/182	226/749	0,314064166	0,999954951	0,999954951	S100A9/HIF1A/LTF/PLAAT4/SOD2/BCL6/STAT3/CD24/IFITM1/PSMB8/SOCS3/TNFRSF1A/MME/HDAC6/COL1A1/COL3A1/	58
GO:0032774	RNA biosynthetic process	58/182	240/749	0,55714386	0,999954951	0,999954951	S100A9/HIF1A/LTF/BCL3/SOD2/BCL6/STAT3/S100A8/SOST/PSMB8/MUC1/TNFRSF1A/HDAC6/COL1A1/NR4A1/PSME2/IC	58
GO:0019219	regulation of nucleobase-containing compound metabolic proce	58/182	251/749	0,734602744	0,999954951	0,999954951	S100A9/HIF1A/LTF/BCL3/SOD2/BCL6/STAT3/S100A8/SOST/PSMB8/MUC1/TNFRSF1A/HDAC6/COL1A1/NR4A1/PSME2/IC	58
GO:0002684	positive regulation of immune system process	58/182	273/749	0,941908344	0,999954951	0,999954951	HIF1A/C1QB/C1S/FPR1/BCL6/STAT3/C3/CD24/SERPING1/C9/CFB/FCER1G/PSMB8/IL1R1/MUC1/PSME2/ICAM1/C1QA/PS	58
GO:0010628	positive regulation of gene expression	58/182	288/749	0,986160785	0,999954951	0,999954951	HIF1A/BCL3/STAT3/C3/FCER1G/SOST/IL1R1/MUC1/MAPK13/TNFRSF1A/LCN2/COL1A1/NR4A1/MAF/RELB/STAT1/IL4R/T	58
GO:2000112	regulation of cellular macromolecule biosynthetic process	57/182	250/749	0,777916461	0,999954951	0,999954951	S100A9/HIF1A/LTF/BCL3/SOD2/BCL6/STAT3/S100A8/SOST/PSMB8/MUC1/TNFRSF1A/HDAC6/COL1A1/NR4A1/PSME2/IC	57
GO:0051641	cellular localization	56/182	203/749	0,118902945	0,999954951	0,999954951	SERPINA3/S100A9/HIF1A/LTF/BCL3/FPR1/TIMP1/STAT3/S100A8/C3/CD24/SLPI/SERPING1/FCER1G/TNFRSF1A/FCGR3B/I	56
GO:0046903	secretion	56/182	214/749	0,253393076	0,999954951	0,999954951	SERPINA3/S100A9/HIF1A/LTF/FPR1/TIMP1/STAT3/S100A8/C3/ITGB6/SLPI/SERPING1/FCER1G/TNFRSF1A/FCGR3B/MME/LCN2/I	56
GO:0051247	positive regulation of protein metabolic process	56/182	236/749	0,630517902	0,999954951	0,999954951	S100A9/LTF/BCL3/FPR1/BCL6/STAT3/S100A8/C3/CD24/SOCS3/MUC1/TNFRSF1A/LTBR/HDAC6/PSME2/ICAM1/FCGR1A/	56
GO:2001141	regulation of RNA biosynthetic process	56/182	237/749	0,647124752	0,999954951	0,999954951	S100A9/HIF1A/LTF/BCL3/SOD2/BCL6/STAT3/S100A8/SOST/PSMB8/MUC1/TNFRSF1A/HDAC6/COL1A1/NR4A1/PSME2/IC	56
GO:0006351	transcription, DNA-templated	56/182	238/749	0,663428279	0,999954951	0,999954951	S100A9/HIF1A/LTF/BCL3/SOD2/BCL6/STAT3/S100A8/SOST/PSMB8/MUC1/TNFRSF1A/HDAC6/COL1A1/NR4A1/PSME2/IC	56
GO:0009653	anatomical structure morphogenesis	56/182	238/749	0,663428279	0,999954951	0,999954951	HIF1A/LTF/BCL3/JAK1/BCL6/STAT3/C3/COL4A1/ADAMTS1/PSMB8/LYVE1/SOCS3/TNFRSF1A/KRT8/HDAC6/COL1A1/NR4	56
GO:0097659	nucleic acid-templated transcription	56/182	238/749	0,663428279	0,999954951	0,999954951	S100A9/HIF1A/LTF/BCL3/SOD2/BCL6/STAT3/S100A8/SOST/PSMB8/MUC1/TNFRSF1A/HDAC6/COL1A1/NR4A1/PSME2/IC	56
GO:0051252	regulation of RNA metabolic process	56/182	239/749	0,679403514	0,999954951	0,999954951	S100A9/HIF1A/LTF/BCL3/SOD2/BCL6/STAT3/S100A8/SOST/PSMB8/MUC1/TNFRSF1A/HDAC6/COL1A1/NR4A1/PSME2/IC	56
GO:0031324	negative regulation of cellular metabolic process	55/182	196/749	0,092281125	0,999954951	0,999954951	SERPINA3/HIF1A/LTF/BCL3/RARRES1/BCL6/TIMP1/STAT3/C3/SLPI/SERPING1/SOCS3/MUC1/HDAC6/MAF/RELB/JAK3/ST	55
GO:0032940	secretion by cell	55/182	204/749	0,172504363	0,999954951	0,999954951	SERPINA3/S100A9/HIF1A/LTF/FPR1/TIMP1/S100A8/C3/ITGB6/SLPI/SERPING1/FCER1G/TNFRSF1A/FCGR3B/MME/LCN2/I	55
GO:0140352	export from cell	55/182	204/749	0,172504363	0,999954951	0,999954951	SERPINA3/S100A9/HIF1A/LTF/FPR1/TIMP1/S100A8/C3/ITGB6/SLPI/SERPING1/FCER1G/TNFRSF1A/FCGR3B/MME/LCN2/I	55
GO:0001817	regulation of cytokine production	55/182	211/749	0,268994344	0,999954951	0,999954951	HIF1A/LTF/BCL3/BCL6/STAT3/C3/CD24/ITGB6/FCER1G/IL1R1/MAPK13/RELB/JAK3/STAT1/IL4R/TGFB1/SOCS1/XBP1/STA	55
GO:0009967	positive regulation of signal transduction	55/182	246/749	0,830607248	0,999954951	0,999954951	S100A9/HIF1A/LTF/FPR1/STAT3/S100A8/C3/CD24/PSMB8/IL1R1/SOCS3/TNFRSF1A/LTBR/HDAC6/COL1A1/COL3A1/PSM	55
GO:0010941	regulation of cell death	54/182	233/749	0,715469153	0,999954951	0,999954951	S100A9/HIF1A/LTF/BCL3/SOD2/BCL6/TIMP1/STAT3/S100A8/FCER1G/SOCS3/MUC1/TNFRSF1A/LTBR/HDAC6/ICAM1/C1	54
GO:0006355	regulation of transcription, DNA-templated	54/182	235/749	0,744743385	0,999954951	0,999954951	S100A9/HIF1A/LTF/BCL3/SOD2/BCL6/STAT3/S100A8/SOST/PSMB8/MUC1/TNFRSF1A/HDAC6/COL1A1/NR4A1/PSME2/IC	54
GO:1903506	regulation of nucleic acid-templated transcription	54/182	235/749	0,744743385	0,999954951	0,999954951	S100A9/HIF1A/LTF/BCL3/SOD2/BCL6/STAT3/S100A8/SOST/PSMB8/MUC1/TNFRSF1A/HDAC6/COL1A1/NR4A1/PSME2/IC	54
GO:0048585	negative regulation of response to stimulus	53/182	214/749	0,459718613	0,999954951	0,999954951	HIF1A/LTF/BCL3/SOD2/BCL6/SERPING1/SOST/PSMB8/SOCS3/MUC1/TNFRSF1A/COL3A1/PSME2/ICAM1/PSMB10/JAK3/S	53
GO:0016310	phosphorylation	53/182	246/749	0,907289543	0,999954951	0,999954951	HIF1A/LTF/JAK1/FPR1/STAT3/C3/CD24/PSMB8/SOCS3/MAPK13/TNFRSF1A/LTBR/HDAC6/PSME2/ICAM1/PSMB10/FCGR	53
GO:0032270	positive regulation of cellular protein metabolic process	52/182	217/749	0,588732421	0,999954951	0,999954951	S100A9/LTF/BCL3/FPR1/BCL6/STAT3/S100A8/C3/CD24/SOCS3/MUC1/TNFRSF1A/LTBR/HDAC6/PSME2/ICAM1/FCGR1A/	52
GO:0042127	regulation of cell population proliferation	52/182	254/749	0,968046152	0,999954951	0,999954951	OSMR/HIF1A/LTF/PLAAT4/RARRES1/SOD2/BCL6/TIMP1/STAT3/CD24/ADAMTS1/IFITM1/NR4A1/JAK3/STAT1/IL4R/TGFB	52
GO:0031399	regulation of protein modification process	51/182	211/749	0,555277054	0,999954951	0,999954951	LTF/FPR1/BCL6/STAT3/C3/CD24/SOCS3/MUC1/TNFRSF1A/LTBR/HDAC6/ICAM1/FCGR1A/TGFB1/SOCS1/DNMT1/BAX/CD	51
GO:0006468	protein phosphorylation	51/182	235/749	0,887883812	0,999954951	0,999954951	LTF/JAK1/FPR1/STAT3/C3/CD24/PSMB8/SOCS3/MAPK13/TNFRSF1A/LTBR/HDAC6/PSME2/ICAM1/PSMB10/FCGR1A/JAK	51
GO:0016192	vesicle-mediated transport	50/182	182/749	0,147479822	0,999954951	0,999954951	SERPINA3/S100A9/LTF/CD163/FPR1/TIMP1/S100A8/C3/SLPI/SERPING1/FCER1G/FCGR3B/EHD3/MME/LCN2/IMPDH1/FC	50
GO:0043067	regulation of programmed cell death	50/182	215/749	0,695448145	0,999954951	0,999954951	S100A9/HIF1A/LTF/BCL3/SOD2/BCL6/TIMP1/STAT3/S100A8/FCER1G/SOCS3/MUC1/TNFRSF1A/LTBR/HDAC6/ICAM1/JAK	50
GO:0002443	leukocyte mediated immunity	49/182	182/749	0,197178568	0,999954951	0,999954951	SERPINA3/S100A9/LTF/BCL3/C1QB/C1S/FPR1/BCL6/S100A8/C3/SLPI/SERPING1/C9/FCER1G/IL1R1/FCGR3B/MME/LCN2/	49
GO:0051241	negative regulation of multicellular organismal process	49/182	186/749	0,255836409	0,999954951	0,999954951	HIF1A/LTF/BCL3/BCL6/TIMP1/STAT3/CD24/SERPING1/SOST/TNFRSF1A/COL3A1/RELB/JAK3/STAT1/IL4R/TGFB1/SOCS1/D	49
GO:0044093	positive regulation of molecular function	49/182	197/749	0,44836764	0,999954951	0,999954951	S100A9/HIF1A/LTF/PLAAT4/FPR1/STAT3/S100A8/CD24/EHD3/ABCB1/PSME2/ICAM1/FCGR1A/TGFB1/SOCS1/BAX/CDKN	49
GO:0042981	regulation of apoptotic process	49/182	211/749	0,698292343	0,999954951	0,999954951	S100A9/HIF1A/LTF/BCL3/SOD2/BCL6/TIMP1/STAT3/S100A8/FCER1G/SOCS3/MUC1/TNFRSF1A/LTBR/ICAM1/JAK3/STAT1	49
GO:0033036	macromolecule localization	49/182	212/749	0,713992564	0,999954951	0,999954951	HIF1A/BCL3/BCL6/STAT3/C3/CD24/ITGB6/FCER1G/TNFRSF1A/EHD3/ABCB1/GBP2/HDAC6/COL1A1/IL4R/TGFB1/SOCS1/X	49
GO:1902531	regulation of intracellular signal transduction	49/182	216/749	0,772315166	0,999954951	0,999954951	S100A9/HIF1A/LTF/BCL3/FPR1/SOD2/BCL6/S100A8/CD24/MUC1/TNFRSF1A/LTBR/COL3A1/ICAM1/STAT1/TGFB1/XBP1/I	49
GO:0031347	regulation of defense response	48/182	169/749	0,095885043	0,999954951	0,999954951	OSMR/S100A9/JAK1/BCL6/S100A8/C3/SERPING1/FCER1G/IFNGR2/PSMB8/IL1R1/SOCS3/MUC1/MAPK13/TNFRSF1A/IFN	48
GO:0007267	cell-cell signaling	48/182	210/749	0,746987795	0,999954951	0,999954951	S100A9/HIF1A/STAT3/S100A8/C3/CD24/ITGB6/FCER1G/SOST/PSMB8/MME/COL1A1/PSME2/C1QA/PSMB10/IL4R/SOCS	48
GO:0042592	homeostatic process	47/182	197/749	0,601598616	0,999954951	0,999954951	SERPINA3/S100A9/HIF1A/LTF/FPR1/SOD2/BCL6/STAT3/S100A8/CD24/FCER1G/LCN2/ICAM1/JAK3/STAT1/IL4R/TGFB1/X	47
GO:0071702	organic substance transport	47/182	199/749	0,637166073	0,999954951	0,999954951	HIF1A/STAT3/S100A8/C3/CD24/ITGB6/FCER1G/EHD3/ABCB1/HDAC6/COL1A1/IL4R/TGFB1/SOCS1/XBP1/BAX/CDKN1A/I	47
GO:0019220	regulation of phosphate metabolic process	47/182	209/749	0,791426904	0,999954951	0,999954951	HIF1A/LTF/FPR1/STAT3/C3/CD24/SOCS3/TNFRSF1A/LTBR/HDAC6/ICAM1/FCGR1A/TGFB1/SOCS1/BAX/CDKN1A/AGT/LD	47
GO:0051174	regulation of phosphorus metabolic process	47/182	209/749	0,791426904	0,999954951	0,999954951	HIF1A/LTF/FPR1/STAT3/C3/CD24/SOCS3/TNFRSF1A/LTBR/HDAC6/ICAM1/FCGR1A/TGFB1/SOCS1/BAX/CDKN1A/AGT/LD	47
GO:0040011	locomotion	47/182	244/749	0,990704061	0,999954951	0,999954951	S100A9/HIF1A/FPR1/SOD2/TIMP1/STAT3/S100A8/CD24/ITGB6/ADAMTS1/IFITM1/FCER1G/IL1R1/HDAC6/COL1A1/NR4A	47
GO:0009888	tissue development	46/182	179/749	0,341693139	0,999954951	0,999954951	ALDH3A2/HIF1A/LTF/PLAAT4/TIMP1/CD24/COL4A1/PSMB8/SOCS3/TNFRSF1A/KRT8/COL1A1/COL3A1/PSME2/ICAM1/M	46
GO:0010629	negative regulation of gene expression	46/182	195/749	0,640023004	0,999954951	0,999954951	HIF1A/LTF/BCL3/BCL6/STAT3/CD24/PSMB8/MUC1/HDAC6/PSME2/MAF/PSMB10/RELB/JAK3/STAT1/TGFB1/DNMT1/AH	46
GO:0051094	positive regulation of developmental process	46/182	202/749	0,752971372	0,999954951	0,999954951	S100A9/HIF1A/LTF/PLAAT4/JAK1/SOD2/BCL6/STAT3/C3/CD24/IFITM1/SOCS3/TNFRSF1A/MME/COL1A1/STAT1/IL4R/NF	46

GO:1901701	cellular response to oxygen-containing compound	45/182	165/749	0,18203504	0,999954951	0,999954951	HIF1A/LTF/SOD2/STAT3/COL4A1/SOCS3/MAPK13/LCN2/HDAC6/COL1A1/NR4A1/COL3A1/ICAM1/JAK3/STAT1/TGFB1/SC	45
GO:0006928	movement of cell or subcellular component	45/182	241/749	0,995367795	0,999954951	0,999954951	S100A9/HIF1A/SOD2/BCL6/TIMP1/STAT3/S100A8/CD24/ITGB6/ADAMTS1/IFITM1/FCER1G/IL1R1/HDAC6/COL1A1/NR4A	45
GO:0042325	regulation of phosphorylation	44/182	200/749	0,836832357	0,999954951	0,999954951	HIF1A/LTF/FPR1/STAT3/C3/CD24/SOCS3/TNFRSF1A/LTBR/HDAC6/ICAM1/FCGR1A/TGFB1/SOCS1/BAX/CDKN1A/AGT/EG	44
GO:0048870	cell motility	44/182	234/749	0,993642161	0,999954951	0,999954951	S100A9/HIF1A/SOD2/TIMP1/STAT3/S100A8/CD24/ITGB6/ADAMTS1/IFITM1/FCER1G/IL1R1/HDAC6/COL1A1/NR4A1/COL	44
GO:0051674	localization of cell	44/182	234/749	0,993642161	0,999954951	0,999954951	S100A9/HIF1A/SOD2/TIMP1/STAT3/S100A8/CD24/ITGB6/ADAMTS1/IFITM1/FCER1G/IL1R1/HDAC6/COL1A1/NR4A1/COL	44
GO:0048468	cell development	43/182	169/749	0,38168859	0,999954951	0,999954951	S100A9/HIF1A/LTF/BCL6/STAT3/S100A8/C3/TNFRSF1A/MME/KRT8/HDAC6/COL3A1/ICAM1/C1QA/MAF/TGFB1/XBP1/B	43
GO:0048534	hematopoietic or lymphoid organ development	43/182	205/749	0,920034809	0,999954951	0,999954951	HIF1A/LTF/BCL3/BCL6/STAT3/FCER1G/PSMB8/LTBR/PSME2/PSMB10/RELB/JAK3/STAT1/IL4R/NFKBIZ/TGFB1/SOCS1/XBP	43
GO:0002520	immune system development	43/182	217/749	0,973940825	0,999954951	0,999954951	HIF1A/LTF/BCL3/BCL6/STAT3/FCER1G/PSMB8/LTBR/PSME2/PSMB10/RELB/JAK3/STAT1/IL4R/NFKBIZ/TGFB1/SOCS1/XBP	43
GO:0016477	cell migration	43/182	229/749	0,993188953	0,999954951	0,999954951	S100A9/HIF1A/SOD2/TIMP1/STAT3/S100A8/CD24/ITGB6/ADAMTS1/IFITM1/FCER1G/IL1R1/HDAC6/COL1A1/NR4A1/COL	43
GO:0009719	response to endogenous stimulus	42/182	177/749	0,615726476	0,999954951	0,999954951	TIMP1/STAT3/COL4A1/FCER1G/SOST/SOCS3/MAPK13/HDAC6/COL1A1/NR4A1/COL3A1/ICAM1/JAK3/STAT1/TGFB1/SOC	42
GO:0001932	regulation of protein phosphorylation	42/182	193/749	0,853679636	0,999954951	0,999954951	LTF/FPR1/STAT3/C3/CD24/SOCS3/TNFRSF1A/LTBR/HDAC6/ICAM1/FCGR1A/TGFB1/SOCS1/BAX/CDKN1A/AGT/EGR1/TLR	42
GO:0008104	protein localization	42/182	195/749	0,873875398	0,999954951	0,999954951	HIF1A/BCL3/BCL6/STAT3/CD24/ITGB6/FCER1G/TNFRSF1A/EHD3/GBP2/HDAC6/COL1A1/IL4R/TGFB1/SOCS1/XBP1/BAX/	42
GO:0046649	lymphocyte activation	42/182	235/749	0,998234915	0,999954951	0,999954951	BCL3/BCL6/STAT3/CD24/FCER1G/ICAM1/IMPDH1/PSMB10/RELB/JAK3/IL4R/NFKBIZ/TGFB1/SOCS1/XBP1/BAX/AHR/CDK	42
GO:0010648	negative regulation of cell communication	41/182	169/749	0,542082055	0,999954951	0,999954951	HIF1A/LTF/BCL3/SOD2/BCL6/SOST/PSMB8/SOCS3/MUC1/PSME2/ICAM1/PSMB10/STAT1/TGFB1/SOCS1/XBP1/BAX/AGT	41
GO:0023057	negative regulation of signaling	41/182	170/749	0,561602666	0,999954951	0,999954951	HIF1A/LTF/BCL3/SOD2/BCL6/SOST/PSMB8/SOCS3/MUC1/PSME2/ICAM1/PSMB10/STAT1/TGFB1/SOCS1/XBP1/BAX/AGT	41
GO:0071705	nitrogen compound transport	41/182	180/749	0,739013212	0,999954951	0,999954951	HIF1A/STAT3/S100A8/CD24/ITGB6/FCER1G/EHD3/ABCB1/HDAC6/COL1A1/IL4R/TGFB1/SOCS1/XBP1/BAX/CDKN1A/IL17	41
GO:0050778	positive regulation of immune response	41/182	191/749	0,876669286	0,999954951	0,999954951	C1QB/C1S/FPR1/C3/SERPING1/C9/CFB/FCER1G/PSMB8/IL1R1/MUC1/PSME2/C1QA/PSMB10/RELB/FCGR1A/IL4R/NFKBI	41
GO:0030097	hemopoiesis	41/182	196/749	0,917624422	0,999954951	0,999954951	HIF1A/LTF/BCL3/BCL6/STAT3/FCER1G/PSMB8/LTBR/PSME2/PSMB10/RELB/JAK3/STAT1/IL4R/NFKBIZ/TGFB1/SOCS1/XBP	41
GO:0009891	positive regulation of biosynthetic process	41/182	208/749	0,973348927	0,999954951	0,999954951	HIF1A/BCL3/STAT3/SOST/MUC1/TNFRSF1A/HDAC6/COL1A1/NR4A1/ICAM1/MAF/RELB/STAT1/TGFB1/XBP1/AHR/STAT6	41
GO:0009628	response to abiotic stimulus	40/182	141/749	0,127428679	0,999954951	0,999954951	HIF1A/BCL3/CD24/SOST/PSMB8/MAPK13/TNFRSF1A/ABCB1/MME/KRT8/LTBR/COL1A1/COL3A1/PSME2/ICAM1/PSMB1	40
GO:0051128	regulation of cellular component organization	40/182	168/749	0,602942632	0,999954951	0,999954951	S100A9/HIF1A/BCL6/S100A8/C3/SOST/MUC1/TNFRSF1A/EHD3/LCN2/HDAC6/ICAM1/IL4R/TGFB1/DNMT1/BAX/CXCL16/	40
GO:0006366	transcription by RNA polymerase II	40/182	182/749	0,825722563	0,999954951	0,999954951	HIF1A/BCL3/SOD2/BCL6/STAT3/PSMB8/MUC1/TNFRSF1A/NR4A1/PSME2/MAF/PSMB10/RELB/STAT1/TGFB1/XBP1/DN	40
GO:0031328	positive regulation of cellular biosynthetic process	40/182	205/749	0,977005522	0,999954951	0,999954951	HIF1A/BCL3/STAT3/SOST/MUC1/TNFRSF1A/COL1A1/NR4A1/ICAM1/MAF/RELB/STAT1/TGFB1/XBP1/AHR/STAT6/AGT/L	40
GO:0051049	regulation of transport	40/182	208/749	0,983393034	0,999954951	0,999954951	HIF1A/S100A8/C3/FCER1G/TNFRSF1A/EHD3/ABCB1/ICAM1/IL4R/TGFB1/SOCS1/XBP1/BAX/IL17RA/AGT/FKBP1A/TLR4/C	40
GO:0043085	positive regulation of catalytic activity	39/182	157/749	0,466640454	0,999954951	0,999954951	S100A9/HIF1A/LTF/PLAAT4/FPR1/STAT3/S100A8/CD24/PSME2/ICAM1/FCGR1A/TGFB1/SOCS1/BAX/CDKN1A/AGT/EGR1	39
GO:0001819	positive regulation of cytokine production	39/182	159/749	0,507142806	0,999954951	0,999954951	HIF1A/BCL3/STAT3/C3/FCER1G/IL1R1/MAPK13/STAT1/IL4R/TGFB1/XBP1/STAT6/IL17RA/AGT/CD276/LY96/EGR1/NFKB2	39
GO:0072359	circulatory system development	39/182	159/749	0,507142806	0,999954951	0,999954951	HIF1A/JAK1/SOD2/STAT3/C3/COL4A1/ADAMTS1/SOCS3/TNFRSF1A/COL1A1/NR4A1/COL3A1/STAT1/TGFB1/XBP1/DNMT	39
GO:0033554	cellular response to stress	39/182	164/749	0,605984104	0,999954951	0,999954951	HIF1A/BCL3/SOD2/BCL6/PSMB8/MUC1/MAPK13/TNFRSF1A/LCN2/LTBR/HDAC6/PSME2/ICAM1/PSMB10/RELB/XBP1/BA	39
GO:0009617	response to bacterium	39/182	170/749	0,713812222	0,999954951	0,999954951	S100A9/LTF/BCL3/S100A8/C3/CD24/SLPI/FCER1G/TNFRSF1A/GBP2/LCN2/ICAM1/TGFB1/XBP1/VCAM1/SIGIRR/LY96/NF	39
GO:0022610	biological adhesion	39/182	235/749	0,999764662	0,999954951	0,999954951	S100A9/LTF/BCL6/S100A8/CD24/ITGB6/LYVE1/MUC1/COL1A1/COL3A1/MEGF11/ICAM1/JAK3/IL4R/NFKBIZ/TGFB1/SOC	39
GO:0009968	negative regulation of signal transduction	38/182	153/749	0,468595999	0,999954951	0,999954951	HIF1A/LTF/BCL3/SOD2/BCL6/SOST/PSMB8/SOCS3/MUC1/PSME2/ICAM1/PSMB10/STAT1/TGFB1/SOCS1/XBP1/BAX/AGT	38
GO:0042886	amide transport	38/182	166/749	0,717386457	0,999954951	0,999954951	HIF1A/STAT3/S100A8/CD24/ITGB6/EHD3/ABCB1/HDAC6/COL1A1/IL4R/TGFB1/SOCS1/XBP1/BAX/CDKN1A/IL17RA/AGT/	38
GO:0031401	positive regulation of protein modification process	38/182	173/749	0,820089706	0,999954951	0,999954951	LTF/FPR1/BCL6/STAT3/C3/CD24/SOCS3/MUC1/TNFRSF1A/LTBR/HDAC6/ICAM1/FCGR1A/TGFB1/SOCS1/DNMT1/CDKN1	38
GO:0006357	regulation of transcription by RNA polymerase II	38/182	176/749	0,855312338	0,999954951	0,999954951	HIF1A/BCL3/SOD2/BCL6/STAT3/PSMB8/MUC1/TNFRSF1A/NR4A1/PSME2/MAF/PSMB10/RELB/STAT1/TGFB1/XBP1/DN	38
GO:0007155	cell adhesion	38/182	234/749	0,99986858	0,999954951	0,999954951	S100A9/BCL6/S100A8/CD24/ITGB6/LYVE1/MUC1/COL1A1/COL3A1/MEGF11/ICAM1/JAK3/IL4R/NFKBIZ/TGFB1/SOCS1/X	38
GO:0045597	positive regulation of cell differentiation	37/182	154/749	0,572857965	0,999954951	0,999954951	S100A9/HIF1A/LTF/PLAAT4/SOD2/BCL6/STAT3/CD24/IFITM1/SOCS3/MME/COL1A1/STAT1/IL4R/NFKBIZ/TGFB1/SOCS1/	37
GO:0015833	peptide transport	37/182	164/749	0,753275145	0,999954951	0,999954951	HIF1A/STAT3/S100A8/CD24/ITGB6/EHD3/HDAC6/COL1A1/IL4R/TGFB1/SOCS1/XBP1/BAX/CDKN1A/IL17RA/AGT/TLR4/A	37
GO:0033993	response to lipid	37/182	172/749	0,858597787	0,999954951	0,999954951	LTF/STAT3/S100A8/SLPI/LCN2/HDAC6/COL1A1/NR4A1/ICAM1/TGFB1/XBP1/AHR/CDKN1A/VCAM1/AGT/SIGIRR/LDLR/T	37
GO:0002250	adaptive immune response	37/182	185/749	0,954238944	0,999954951	0,999954951	BCL3/C1QB/C1S/BCL6/STAT3/C3/SERPING1/C9/FCER1G/IL1R1/ICAM1/C1QA/RELB/JAK3/IL4R/NFKBIZ/TGFB1/STAT6/IL1	37
GO:0010557	positive regulation of macromolecule biosynthetic process	37/182	195/749	0,984039995	0,999954951	0,999954951	HIF1A/BCL3/STAT3/SOST/MUC1/TNFRSF1A/HDAC6/COL1A1/NR4A1/MAF/RELB/STAT1/TGFB1/XBP1/AHR/STAT6/AGT/C	37
GO:0015031	protein transport	36/182	160/749	0,757061896	0,999954951	0,999954951	HIF1A/STAT3/CD24/ITGB6/EHD3/HDAC6/COL1A1/IL4R/TGFB1/SOCS1/XBP1/BAX/CDKN1A/IL17RA/AGT/TLR4/APOE/TLR	36
GO:0045184	establishment of protein localization	36/182	164/749	0,814408844	0,999954951	0,999954951	HIF1A/STAT3/CD24/ITGB6/EHD3/HDAC6/COL1A1/IL4R/TGFB1/SOCS1/XBP1/BAX/CDKN1A/IL17RA/AGT/TLR4/APOE/TLR	36
GO:0045935	positive regulation of nucleobase-containing compound metabo	36/182	179/749	0,94662968	0,999954951	0,999954951	HIF1A/BCL3/STAT3/SOST/MUC1/TNFRSF1A/COL1A1/NR4A1/MAF/RELB/STAT1/TGFB1/XBP1/BAX/AHR/STAT6/AGT/EGR	36
GO:0032103	positive regulation of response to external stimulus	35/182	149/749	0,638403891	0,999954951	0,999954951	OSMR/S100A9/S100A8/C3/FCER1G/PSMB8/MUC1/MAPK13/TNFRSF1A/PSME2/PSMB10/RELB/NFKBIZ/TGFB1/IL17RA/A	35
GO:0060548	negative regulation of cell death	35/182	152/749	0,694106099	0,999954951	0,999954951	HIF1A/LTF/BCL3/SOD2/BCL6/TIMP1/STAT3/FCER1G/SOCS3/MUC1/ICAM1/JAK3/XBP1/DNMT1/BAX/CDKN1A/KITLG/MY	35
GO:0048878	chemical homeostasis	34/182	125/749	0,235599199	0,999954951	0,999954951	S100A9/HIF1A/LTF/FPR1/SOD2/STAT3/S100A8/CD24/LCN2/ICAM1/STAT1/TGFB1/XBP1/BAX/AGT/LDLR/SLC12A3/FKBP1	34
GO:0043066	negative regulation of apoptotic process	34/182	139/749	0,519361108	0,999954951	0,999954951	HIF1A/LTF/BCL3/SOD2/BCL6/TIMP1/STAT3/FCER1G/SOCS3/MUC1/ICAM1/JAK3/XBP1/DNMT1/BAX/CDKN1A/KITLG/MY	34
GO:0043069	negative regulation of programmed cell death	34/182	141/749	0,56139455	0,999954951	0,999954951	HIF1A/LTF/BCL3/SOD2/BCL6/TIMP1/STAT3/FCER1G/SOCS3/MUC1/ICAM1/JAK3/XBP1/DNMT1/BAX/CDKN1A/KITLG/MY	34
GO:0007399	nervous system development	34/182	151/749	0,749089508	0,999954951	0,999954951	ALDH3A2/S100A9/HIF1A/BCL6/STAT3/S100A8/C3/COL4A1/MME/HDAC6/COL3A1/C1QA/TGFB1/XBP1/BAX/VCAM1/AGT	34
GO:0051254	positive regulation of RNA metabolic process	34/182	168/749	0,934366835	0,999954951	0,999954951	HIF1A/BCL3/STAT3/SOST/MUC1/TNFRSF1A/COL1A1/NR4A1/MAF/RELB/STAT1/TGFB1/XBP1/AHR/STAT6/AGT/EGR1/NF	34
GO:0010562	positive regulation of phosphorus metabolic process	34/182	173/749	0,959681182	0,999954951	0,999954951	HIF1A/LTF/FPR1/STAT3/C3/CD24/SOCS3/TNFRSF1A/LTBR/HDAC6/ICAM1/FCGR1A/TGFB1/SOCS1/CDKN1A/AGT/EGR1/T	34
GO:0045937	positive regulation of phosphate metabolic process	34/182	173/749	0,959681182	0,999954951	0,999954951	HIF1A/LTF/FPR1/STAT3/C3/CD24/SOCS3/TNFRSF1A/LTBR/HDAC6/ICAM1/FCGR1A/TGFB1/SOCS1/CDKN1A/AGT/EGR1/T	34
GO:0051336	regulation of hydrolase activity	33/182	129/749	0,39276261	0,999954951	0,999954951	SERPINA3/S100A9/LTF/RARRES1/BCL6/TIMP1/STAT3/S100A8/C3/SLPI/SERPING1/PSMB8/PSME2/ICAM1/BAX/AGT/FKBP	33
GO:0045893	positive regulation of transcription, DNA-templated	33/182	164/749	0,936855485	0,999954951	0,999954951	HIF1A/BCL3/STAT3/SOST/MUC1/TNFRSF1A/COL1A1/NR4A1/MAF/RELB/STAT1/TGFB1/XBP1/AHR/STAT6/AGT/EGR1/NF	33
GO:1902680	positive regulation of RNA biosynthetic process	33/182	165/749	0,942613213	0,999954951	0,999954951	HIF1A/BCL3/STAT3/SOST/MUC1/TNFRSF1A/COL1A1/NR4A1/MAF/RELB/STAT1/TGFB1/XBP1/AHR/STAT6/AGT/EGR1/NF	33
GO:1903508	positive regulation of nucleic acid-templated transcription	33/182	165/749	0,942613213	0,999954951	0,999954951	HIF1A/BCL3/STAT3/SOST/MUC1/TNFRSF1A/COL1A1/NR4A1/MAF/RELB/STAT1/TGFB1/XBP1/AHR/STAT6/AGT/EGR1/NF	33
GO:0042110	T cell activation	33/182	179/749	0,987362996	0,999954951	0,999954951	BCL3/BCL6/STAT3/CD24/FCER1G/ICAM1/PSMB10/RELB/JAK3/IL4R/NFKBIZ/TGFB1/SOCS1/XBP1/BAX/VCAM1/STAT6/CD	33
GO:0042493	response to drug	32/182	119/749	0,270691855	0,999954951	0,999954951	STAT3/S100A8/NNMT/MAPK13/ABCB1/LCN2/HDAC6/COL1A1/ICAM1/STAT1/TGFB1/AHR/CDKN1A/VCAM1/STAT6/AGT/	32
GO:0044403	symbiotic process	32/182	130/749	0,502916571	0,999954951	0,999954951	LTF/IFITM3/STAT3/ITGB6/SLPI/IFITM2/IFITM1/HAVCR1/PSMB8/TNFRSF1A/KRT8/LTBR/ICAM1/C1QA/PSMB10/STAT1/TG	32

GO:0071495	cellular response to endogenous stimulus	32/182	138/749	0,668896973	0,999954951	0,999954951	STAT3/COL4A1/SOST/SOCS3/MAPK13/HDAC6/COL1A1/NR4A1/COL3A1/ICAM1/JAK3/STAT1/TGFB1/SOCS1/XBP1/DNMT	32
GO:0048646	anatomical structure formation involved in morphogenesis	32/182	150/749	0,854267337	0,999954951	0,999954951	HIF1A/BCL3/JAK1/BCL6/STAT3/C3/COL4A1/ADAMTS1/KRT8/COL1A1/NR4A1/MEGF11/STAT1/IL4R/TGFB1/XBP1/AGT/FK	32
GO:0042327	positive regulation of phosphorylation	32/182	165/749	0,963328047	0,999954951	0,999954951	HIF1A/LTF/FPR1/STAT3/C3/CD24/SOCS3/TNFRSF1A/LTBR/HDAC6/ICAM1/FCGR1A/TGFB1/SOCS1/CDKN1A/AGT/EGR1/TI	32
GO:1902533	positive regulation of intracellular signal transduction	32/182	169/749	0,976205457	0,999954951	0,999954951	S100A9/LTF/FPR1/S100A8/CD24/TNFRSF1A/LTBR/COL3A1/ICAM1/TGFB1/BAX/AGT/FKBP1A/TLR4/KITLG/MYD88/APOE/	32
GO:0009056	catabolic process	31/182	123/749	0,43892246	0,999954951	0,999954951	ALDH3A2/S100A9/HIF1A/PLAAT4/TIMP1/STAT3/S100A8/PSMB8/LYVE1/MME/HDAC6/PSME2/PSMB10/TGFB1/XBP1/BA	31
GO:0016032	viral process	31/182	123/749	0,43892246	0,999954951	0,999954951	LTF/IFITM3/STAT3/ITGB6/SLPI/IFITM2/IFITM1/HAVCR1/PSMB8/TNFRSF1A/KRT8/LTBR/ICAM1/C1QA/PSMB10/STAT1/TG	31
GO:0006996	organelle organization	31/182	147/749	0,869260087	0,999954951	0,999954951	S100A9/HIF1A/SOD2/BCL6/STAT3/S100A8/MUC1/EHD3/KRT8/HDAC6/ICAM1/TGFB1/DNMT1/BAX/CD59/SLAMF8/APOE	31
GO:0000165	MAPK cascade	31/182	149/749	0,889467458	0,999954951	0,999954951	JAK1/FPR1/CD24/PSMB8/MAPK13/LTBR/PSME2/ICAM1/PSMB10/JAK3/TGFB1/AGT/TLR4/KITLG/APOE/MAPK11/MYC/S1	31
GO:0023014	signal transduction by protein phosphorylation	31/182	149/749	0,889467458	0,999954951	0,999954951	JAK1/FPR1/CD24/PSMB8/MAPK13/LTBR/PSME2/ICAM1/PSMB10/JAK3/TGFB1/AGT/TLR4/KITLG/APOE/MAPK11/MYC/S1	31
GO:0002521	leukocyte differentiation	31/182	160/749	0,961267531	0,999954951	0,999954951	LTF/BCL3/BCL6/STAT3/FCER1G/LTBR/RELB/JAK3/IL4R/NFKBIZ/TGFB1/SOCS1/XBP1/BAX/VCAM1/STAT6/EGR1/TLR4/KITL	31
GO:0001934	positive regulation of protein phosphorylation	31/182	162/749	0,968698974	0,999954951	0,999954951	LTF/FPR1/STAT3/C3/CD24/SOCS3/TNFRSF1A/LTBR/HDAC6/ICAM1/FCGR1A/TGFB1/SOCS1/CDKN1A/AGT/EGR1/TLR4/KIT	31
GO:0008284	positive regulation of cell population proliferation	31/182	173/749	0,991391714	0,999954951	0,999954951	OSMR/HIF1A/LTF/BCL6/TIMP1/STAT3/CD24/ADAMTS1/NR4A1/JAK3/STAT1/TGFB1/XBP1/DNMT1/CDKN1A/VCAM1/AGT	31
GO:0050865	regulation of cell activation	31/182	192/749	0,999413202	0,999954951	0,999954951	BCL6/CD24/FCER1G/JAK3/IL4R/NFKBIZ/TGFB1/SOCS1/XBP1/AHR/CDKN1A/VCAM1/STAT6/LDLR/CD276/TLR4/SLAMF8/A	31
GO:0030162	regulation of proteolysis	30/182	95/749	0,052729636	0,999954951	0,999954951	SERPINA3/S100A9/LTF/RARRES1/TIMP1/STAT3/S100A8/C3/SLPI/SERPING1/PSMB8/HDAC6/PSME2/BAX/AGT/APOE/TNF	30
GO:0044281	small molecule metabolic process	30/182	97/749	0,068469899	0,999954951	0,999954951	ALDH3A2/HIF1A/STAT3/C3/NNMT/PSMB8/LYVE1/TNFRSF1A/MME/PSME2/IMPDH1/PSMB10/TGFB1/XBP1/ALOX5/APOI	30
GO:0097190	apoptotic signaling pathway	30/182	107/749	0,195929669	0,999954951	0,999954951	S100A9/HIF1A/BCL3/SOD2/S100A8/CD24/MUC1/TNFRSF1A/LCN2/KRT8/LTBR/ICAM1/TGFB1/XBP1/BAX/CDKN1A/AGT/L	30
GO:0051338	regulation of transferase activity	30/182	119/749	0,4405941	0,999954951	0,999954951	LTF/PLAAT4/FPR1/CD24/SOCS3/FCGR1A/TGFB1/SOCS1/CDKN1A/AGT/EGR1/TLR4/KITLG/SLAMF8/APOE/MAPK11/IL6R/S	30
GO:0044092	negative regulation of molecular function	30/182	120/749	0,463079257	0,999954951	0,999954951	SERPINA3/LTF/RARRES1/TIMP1/C3/SLPI/SERPING1/SOCS3/HDAC6/SOCS1/BAX/CDKN1A/AGT/SIGIRR/IL18BP/FKBP1A/AF	30
GO:0051093	negative regulation of developmental process	30/182	121/749	0,485564808	0,999954951	0,999954951	HIF1A/LTF/BCL6/TIMP1/STAT3/COL3A1/JAK3/STAT1/IL4R/TGFB1/SOCS1/XBP1/DNMT1/CDKN1A/AGT/LDLR/TLR4/APOE/	30
GO:0002460	adaptive immune response based on somatic recombination of i	30/182	124/749	0,552328567	0,999954951	0,999954951	BCL3/C1QB/C1S/BCL6/STAT3/C3/SERPING1/C9/FCER1G/IL1R1/ICAM1/C1QA/RELB/JAK3/IL4R/NFKBIZ/TGFB1/STAT6/IL1	30
GO:0018193	peptidyl-amino acid modification	30/182	133/749	0,732507571	0,999954951	0,999954951	S100A9/JAK1/STAT3/S100A8/CD24/SOCS3/MUC1/MAPK13/TNFRSF1A/HDAC6/ICAM1/FCGR1A/JAK3/TGFB1/SOCS1/DNN	30
GO:0002697	regulation of immune effector process	30/182	141/749	0,850572767	0,999954951	0,999954951	C1QB/C1S/BCL6/C3/SERPING1/C9/CFB/FCER1G/IL1R1/ICAM1/C1QA/JAK3/STAT1/IL4R/NFKBIZ/TGFB1/XBP1/STAT6/CD5	30
GO:0051270	regulation of cellular component movement	30/182	156/749	0,963224699	0,999954951	0,999954951	HIF1A/SOD2/BCL6/TIMP1/STAT3/ADAMTS1/IFITM1/IL1R1/HDAC6/COL1A1/COL3A1/ICAM1/TGFB1/XBP1/CXCL16/AGT/K	30
GO:0040012	regulation of locomotion	30/182	158/749	0,970387871	0,999954951	0,999954951	HIF1A/SOD2/TIMP1/STAT3/ADAMTS1/IFITM1/IL1R1/HDAC6/COL1A1/COL3A1/ICAM1/TGFB1/XBP1/CXCL16/AGT/KITLG/	30
GO:0050727	regulation of inflammatory response	29/182	99/749	0,132489465	0,999954951	0,999954951	OSMR/S100A9/BCL6/S100A8/C3/FCER1G/IL1R1/SOCS3/MAPK13/TNFRSF1A/NFKBIZ/IL17RA/AGT/LDLR/TLR4/SLAMF8/M	29
GO:0002831	regulation of response to biotic stimulus	29/182	101/749	0,161581282	0,999954951	0,999954951	LTF/JAK1/SERPING1/FCER1G/IFNGR2/PSMB8/SOCS3/MUC1/IFNAR2/PSME2/PSMB10/RELB/STAT1/SOCS1/SIGIRR/LY96/	29
GO:0044248	cellular catabolic process	29/182	108/749	0,288588654	0,999954951	0,999954951	ALDH3A2/S100A9/HIF1A/TIMP1/STAT3/S100A8/PSMB8/LYVE1/MME/HDAC6/PSME2/PSMB10/TGFB1/XBP1/BAX/LDLR/(	29
GO:0022008	neurogenesis	29/182	119/749	0,533180043	0,999954951	0,999954951	S100A9/HIF1A/BCL6/STAT3/S100A8/C3/MME/HDAC6/COL3A1/C1QA/TGFB1/XBP1/BAX/VCAM1/AGT/LDLR/TNC/TLR4/A	29
GO:0003008	system process	29/182	129/749	0,737073243	0,999954951	0,999954951	SERPINA3/HIF1A/SOD2/SERPING1/TNFRSF1A/EHD3/MME/COL1A1/ICAM1/STAT1/TGFB1/AGT/LDLR/TLR4/APOE/TLR2/L	29
GO:0022603	regulation of anatomical structure morphogenesis	29/182	129/749	0,737073243	0,999954951	0,999954951	HIF1A/JAK1/BCL6/STAT3/C3/PSMB8/TNFRSF1A/PSME2/ICAM1/PSMB10/STAT1/IL4R/TGFB1/XBP1/BAX/AGT/APOE/LST1	29
GO:2000145	regulation of cell motility	29/182	153/749	0,968786799	0,999954951	0,999954951	HIF1A/SOD2/TIMP1/STAT3/ADAMTS1/IFITM1/IL1R1/HDAC6/COL1A1/COL3A1/ICAM1/TGFB1/XBP1/CXCL16/AGT/KITLG/	29
GO:0044085	cellular component biogenesis	29/182	163/749	0,99047802	0,999954951	0,999954951	SOD2/C9/FCER1G/SOST/TNFRSF1A/EHD3/LCN2/KRT8/HDAC6/COL1A1/ICAM1/TGFB1/DNMT1/BAX/AGT/CD59/FKBP1A/	29
GO:0002694	regulation of leukocyte activation	29/182	184/749	0,999535937	0,999954951	0,999954951	BCL6/CD24/FCER1G/JAK3/IL4R/NFKBIZ/TGFB1/SOCS1/XBP1/AHR/CDKN1A/VCAM1/STAT6/LDLR/CD276/TLR4/SLAMF8/L	29
GO:0031349	positive regulation of defense response	28/182	110/749	0,42091196	0,999954951	0,999954951	OSMR/S100A9/S100A8/C3/FCER1G/PSMB8/MUC1/MAPK13/TNFRSF1A/PSME2/PSMB10/RELB/NFKBIZ/IL17RA/AGT/LDLI	28
GO:0043549	regulation of kinase activity	28/182	113/749	0,49029611	0,999954951	0,999954951	LTF/FPR1/CD24/SOCS3/FCGR1A/TGFB1/SOCS1/CDKN1A/AGT/EGR1/TLR4/KITLG/SLAMF8/APOE/MAPK11/IL6R/SLC11A1/	28
GO:0001944	vasculature development	28/182	127/749	0,775536447	0,999954951	0,999954951	HIF1A/JAK1/SOD2/STAT3/C3/COL4A1/SOCS3/COL1A1/NR4A1/COL3A1/STAT1/TGFB1/XBP1/DNMT1/BAX/AHR/AGT/LDLF	28
GO:0072358	cardiovascular system development	28/182	127/749	0,775536447	0,999954951	0,999954951	HIF1A/JAK1/SOD2/STAT3/C3/COL4A1/SOCS3/COL1A1/NR4A1/COL3A1/STAT1/TGFB1/XBP1/DNMT1/BAX/AHR/AGT/LDLF	28
GO:0030334	regulation of cell migration	28/182	149/749	0,970593592	0,999954951	0,999954951	HIF1A/SOD2/TIMP1/STAT3/ADAMTS1/IFITM1/IL1R1/HDAC6/COL1A1/COL3A1/ICAM1/TGFB1/XBP1/CXCL16/AGT/KITLG/	28
GO:0051050	positive regulation of transport	28/182	153/749	0,981487484	0,999954951	0,999954951	HIF1A/S100A8/C3/FCER1G/EHD3/ABCB1/IL4R/TGFB1/XBP1/BAX/IL17RA/AGT/TLR4/CTSS/APOE/TLR2/MAPK11/SLC11A1	28
GO:0035295	tube development	28/182	154/749	0,983580587	0,999954951	0,999954951	HIF1A/JAK1/STAT3/C3/CD24/COL4A1/MME/NR4A1/COL3A1/STAT1/TGFB1/XBP1/BAX/CDKN1A/HSD11B1/AGT/LDLR/TN	28
GO:0022607	cellular component assembly	28/182	160/749	0,992284935	0,999954951	0,999954951	SOD2/C9/FCER1G/SOST/TNFRSF1A/EHD3/LCN2/KRT8/HDAC6/COL1A1/ICAM1/TGFB1/DNMT1/BAX/AGT/CD59/FKBP1A/	28
GO:0030155	regulation of cell adhesion	28/182	179/749	0,999519375	0,999954951	0,999954951	BCL6/CD24/MUC1/COL1A1/ICAM1/JAK3/IL4R/NFKBIZ/TGFB1/SOCS1/XBP1/VCAM1/TNC/CD276/LILRB2/IL6R/TGFBI/ARH	28
GO:1901575	organic substance catabolic process	27/182	98/749	0,24596301	0,999954951	0,999954951	ALDH3A2/HIF1A/PLAAT4/TIMP1/STAT3/PSMB8/LYVE1/HDAC6/PSME2/PSMB10/TGFB1/XBP1/BAX/LDLR/CTSS/VCAN/MY	27
GO:0006811	ion transport	27/182	100/749	0,28705723	0,999954951	0,999954951	LTF/FCER1G/EHD3/ABCB1/LCN2/ICAM1/TGFB1/BAX/AGT/APOL1/LDLR/SLC12A3/FKBP1A/CTSS/APOE/LILRB2/SLC11A1/A	27
GO:0019725	cellular homeostasis	27/182	101/749	0,30853291	0,999954951	0,999954951	S100A9/HIF1A/LTF/FPR1/S100A8/CD24/LCN2/ICAM1/TGFB1/XBP1/BAX/AGT/SLC12A3/FKBP1A/SLAMF8/MT1A/APOE/SL	27
GO:0045859	regulation of protein kinase activity	27/182	105/749	0,398948092	0,999954951	0,999954951	LTF/FPR1/CD24/SOCS3/FCGR1A/TGFB1/SOCS1/CDKN1A/AGT/EGR1/TLR4/KITLG/APOE/MAPK11/IL6R/SLC11A1/S100A12	27
GO:0000003	reproduction	27/182	106/749	0,422287085	0,999954951	0,999954951	HIF1A/BCL6/TIMP1/STAT3/ADAMTS1/SOCS3/MME/KRT8/ICAM1/IL4R/TGFB1/BAX/AGT/TNC/EGR1/KITLG/APOL2/ARHGI	27
GO:0022414	reproductive process	27/182	106/749	0,422287085	0,999954951	0,999954951	HIF1A/BCL6/TIMP1/STAT3/ADAMTS1/SOCS3/MME/KRT8/ICAM1/IL4R/TGFB1/BAX/AGT/TNC/EGR1/KITLG/APOL2/ARHGI	27
GO:0009725	response to hormone	27/182	107/749	0,445756748	0,999954951	0,999954951	TIMP1/STAT3/SOST/SOCS3/HDAC6/COL1A1/NR4A1/ICAM1/JAK3/STAT1/TGFB1/SOCS1/XBP1/CDKN1A/STAT6/AGT/TNC/	27
GO:0002253	activation of immune response	27/182	114/749	0,607002128	0,999954951	0,999954951	C1QB/C1S/FPR1/C3/SERPING1/C9/CFB/FCER1G/PSMB8/MUC1/PSME2/C1QA/PSMB10/RELB/FCGR1A/NFKBIZ/BAX/FCGF	27
GO:0002237	response to molecule of bacterial origin	27/182	115/749	0,628726653	0,999954951	0,999954951	LTF/S100A8/CD24/SLPI/LCN2/ICAM1/TGFB1/XBP1/VCAM1/SIGIRR/LY96/NFKB2/TLR4/CXCL1/TLR2/LILRB2/SLC11A1/TNF	27
GO:1903706	regulation of hemopoiesis	27/182	120/749	0,728684559	0,999954951	0,999954951	HIF1A/LTF/BCL6/STAT3/PSMB8/PSME2/PSMB10/JAK3/STAT1/IL4R/NFKBIZ/TGFB1/SOCS1/XBP1/TLR4/KITLG/SLAMF8/LII	27
GO:0045944	positive regulation of transcription by RNA polymerase II	27/182	141/749	0,956991089	0,999954951	0,999954951	HIF1A/BCL3/STAT3/MUC1/TNFRSF1A/NR4A1/MAF/RELB/STAT1/TGFB1/XBP1/AHR/STAT6/EGR1/NFKB2/TLR4/TLR2/IRF7	27
GO:0055082	cellular chemical homeostasis	26/182	94/749	0,24434924	0,999954951	0,999954951	S100A9/HIF1A/LTF/FPR1/S100A8/CD24/LCN2/ICAM1/TGFB1/XBP1/BAX/AGT/FKBP1A/SLAMF8/MT1A/APOE/SLC11A1/M	26
GO:0009611	response to wounding	26/182	97/749	0,307943502	0,999954951	0,999954951	HIF1A/TIMP1/S100A8/SERPING1/FCER1G/LYVE1/EHD3/COL1A1/COL3A1/TGFB1/XBP1/BAX/CDKN1A/CD59/TNC/TLR4/AI	26
GO:0050801	ion homeostasis	26/182	97/749	0,307943502	0,999954951	0,999954951	S100A9/HIF1A/LTF/FPR1/S100A8/CD24/LCN2/TGFB1/XBP1/BAX/AGT/SLC12A3/FKBP1A/SLAMF8/MT1A/APOE/SLC11A1/	26
GO:0009887	animal organ morphogenesis	26/182	106/749	0,51902422	0,999954951	0,999954951	HIF1A/LTF/STAT3/ADAMTS1/PSMB8/COL1A1/COL3A1/PSME2/MEGF11/PSMB10/STAT1/TGFB1/XBP1/BAX/STAT6/AGT/T	26
GO:0032496	response to lipopolysaccharide	26/182	111/749	0,633044715	0,999954951	0,999954951	LTF/S100A8/SLPI/LCN2/ICAM1/TGFB1/XBP1/VCAM1/SIGIRR/LY96/NFKB2/TLR4/CXCL1/TLR2/LILRB2/SLC11A1/TNFRSF1B	26
GO:0009890	negative regulation of biosynthetic process	26/182	113/749	0,675263848	0,999954951	0,999954951	BCL3/BCL6/STAT3/MUC1/HDAC6/MAF/RELB/JAK3/STAT1/TGFB1/DNMT1/AHR/STAT6/SIGIRR/EGR1/APOE/IRF7/RORC/M	26

GO:0071396	cellular response to lipid	26/182	116/749	0,733692273	0,999954951	0,999954951	LTF/LCN2/HDAC6/COL1A1/NR4A1/ICAM1/TGFB1/XBP1/AHR/SIGIRR/LDLR/TNC/LY96/TLR4/CXCL1/TLR2/LILRB2/RORC/TN	26
GO:0002790	peptide secretion	26/182	117/749	0,751725347	0,999954951	0,999954951	HIF1A/S100A8/ITGB6/IL4R/TGFB1/SOCS1/XBP1/IL17RA/AGT/TLR4/APOE/TLR2/MAPK11/TNFRSF1B/S100A12/IL1R2/HNF	26
GO:0060429	epithelium development	26/182	120/749	0,801209233	0,999954951	0,999954951	HIF1A/PLAAT4/CD24/COL4A1/PSMB8/SOCS3/TNFRSF1A/KRT8/PSME2/ICAM1/MAF/PSMB10/STAT1/TGFB1/XBP1/BAX/C	26
GO:0051249	regulation of lymphocyte activation	26/182	172/749	0,999689149	0,999954951	0,999954951	BCL6/CD24/JAK3/IL4R/NFKBIZ/TGFB1/SOCS1/XBP1/AHR/CDKN1A/VCAM1/STAT6/CD276/TLR4/SLAMF8/LILRB2/LST1/IL6	26
GO:0098609	cell-cell adhesion	26/182	176/749	0,999842649	0,999954951	0,999954951	S100A9/BCL6/S100A8/CD24/MEGF11/ICAM1/JAK3/IL4R/NFKBIZ/TGFB1/SOCS1/XBP1/VCAM1/CD276/LILRB2/IL6R/PVR/C	26
GO:0043086	negative regulation of catalytic activity	25/182	85/749	0,150941964	0,999954951	0,999954951	SERPINA3/LTF/RARRES1/TIMP1/C3/SLPI/SERPING1/SOCS3/HDAC6/SOCS1/CDKN1A/AGT/FKBP1A/APOE/TRIB1/ERRFI1/BI	25
GO:0098771	inorganic ion homeostasis	25/182	90/749	0,242556272	0,999954951	0,999954951	S100A9/HIF1A/LTF/FPR1/S100A8/CD24/LCN2/TGFB1/BAX/AGT/SLC12A3/FKBP1A/SLAMF8/MT1A/APOE/SLC11A1/MYC/C	25
GO:0008285	negative regulation of cell population proliferation	25/182	106/749	0,615342975	0,999954951	0,999954951	PLAAT4/RARRES1/SOD2/BCL6/STAT3/ADAMTS1/IFITM1/STAT1/TGFB1/BAX/CDKN1A/APOE/CXCL1/TLR2/LILRB2/MAPK1	25
GO:0010558	negative regulation of macromolecule biosynthetic process	25/182	107/749	0,637538342	0,999954951	0,999954951	BCL3/BCL6/STAT3/MUC1/HDAC6/MAF/RELB/JAK3/STAT1/TGFB1/DNMT1/AHR/STAT6/SIGIRR/EGR1/IRF7/RORC/MYC/ER	25
GO:0023061	signal release	25/182	109/749	0,680139192	0,999954951	0,999954951	HIF1A/ITGB6/FCER1G/IL4R/SOCS1/XBP1/IL17RA/AGT/TLR4/TLR2/MAPK11/TNFRSF1B/S100A12/IL1R2/HNF1A/CD58/FN1	25
GO:0031327	negative regulation of cellular biosynthetic process	25/182	110/749	0,70044785	0,999954951	0,999954951	BCL3/BCL6/STAT3/MUC1/HDAC6/MAF/RELB/JAK3/STAT1/TGFB1/DNMT1/AHR/STAT6/SIGIRR/EGR1/IRF7/RORC/MYC/ER	25
GO:0051130	positive regulation of cellular component organization	25/182	110/749	0,70044785	0,999954951	0,999954951	S100A9/HIF1A/BCL6/C3/MUC1/LCN2/HDAC6/ICAM1/IL4R/TGFB1/DNMT1/BAX/AGT/TLR4/APOE/TNFSF10/NOX4/FN1/CI	25
GO:0048699	generation of neurons	25/182	111/749	0,720041785	0,999954951	0,999954951	S100A9/HIF1A/BCL6/STAT3/C3/MME/HDAC6/COL3A1/C1QA/TGFB1/XBP1/BAX/VCAM1/AGT/LDLR/TNC/APOE/TLR2/LST	25
GO:0009306	protein secretion	25/182	113/749	0,756964471	0,999954951	0,999954951	HIF1A/ITGB6/IL4R/TGFB1/SOCS1/XBP1/IL17RA/AGT/TLR4/APOE/TLR2/MAPK11/TNFRSF1B/S100A12/IL1R2/HNF1A/CD58	25
GO:0035592	establishment of protein localization to extracellular region	25/182	113/749	0,756964471	0,999954951	0,999954951	HIF1A/ITGB6/IL4R/TGFB1/SOCS1/XBP1/IL17RA/AGT/TLR4/APOE/TLR2/MAPK11/TNFRSF1B/S100A12/IL1R2/HNF1A/CD58	25
GO:0071692	protein localization to extracellular region	25/182	113/749	0,756964471	0,999954951	0,999954951	HIF1A/ITGB6/IL4R/TGFB1/SOCS1/XBP1/IL17RA/AGT/TLR4/APOE/TLR2/MAPK11/TNFRSF1B/S100A12/IL1R2/HNF1A/CD58	25
GO:0010942	positive regulation of cell death	25/182	117/749	0,821255808	0,999954951	0,999954951	S100A9/SOD2/BCL6/S100A8/TNFRSF1A/LTBR/HDAC6/C1QA/TGFB1/BAX/CDKN1A/AGT/EGR1/TLR4/TNFRSF1B/MYC/FGD	25
GO:0001568	blood vessel development	25/182	119/749	0,848550446	0,999954951	0,999954951	HIF1A/JAK1/STAT3/C3/COL4A1/SOCS3/COL1A1/NR4A1/COL3A1/STAT1/TGFB1/XBP1/BAX/AHR/AGT/LDLR/EGR1/APOE/I	25
GO:0051046	regulation of secretion	25/182	126/749	0,920310847	0,999954951	0,999954951	HIF1A/S100A8/FCER1G/TNFRSF1A/IL4R/TGFB1/SOCS1/XBP1/IL17RA/AGT/TLR4/APOE/TLR2/MAPK11/TNFRSF1B/IL1R2/C	25
GO:0050900	leukocyte migration	25/182	139/749	0,981096483	0,999954951	0,999954951	S100A9/S100A8/FCER1G/IL1R1/COL1A1/ICAM1/TGFB1/CXCL16/VCAM1/IL17RA/KITLG/SLAMF8/CXCL1/IL6R/S100A12/CI	25
GO:0052547	regulation of peptidase activity	24/182	75/749	0,069813347	0,999954951	0,999954951	SERPINA3/S100A9/LTF/RARRES1/TIMP1/STAT3/S100A8/C3/SLPI/SERPING1/PSMB8/PSME2/BAX/AGT/MYC/TNFSF10/BIR	24
GO:0006629	lipid metabolic process	24/182	76/749	0,080214955	0,999954951	0,999954951	SERPINA3/ALDH3A2/PLAAT4/C3/SOCS3/TNFRSF1A/TGFB1/SOCS1/XBP1/BAX/ALOX5/HSD11B1/AGT/APOL1/LDLR/EGR1/	24
GO:0045088	regulation of innate immune response	24/182	79/749	0,117574708	0,999954951	0,999954951	JAK1/SERPING1/FCER1G/IFNGR2/PSMB8/SOCS3/MUC1/IFNAR2/PSME2/PSMB10/RELB/STAT1/SOCS1/SLAMF8/APOE/IRI	24
GO:0055085	transmembrane transport	24/182	80/749	0,13212535	0,999954951	0,999954951	C3/PSMB8/EHD3/ABCB1/PSME2/PSMB10/TGFB1/BAX/AGT/APOL1/SLC12A3/FKBP1A/CTSS/SLC11A1/ABCC2/TAP2/HNF1	24
GO:0034612	response to tumor necrosis factor	24/182	84/749	0,200608889	0,999954951	0,999954951	PSMB8/TNFRSF1A/GBP2/LCN2/KRT8/LTBR/COL1A1/PSME2/ICAM1/PSMB10/STAT1/CXCL16/VCAM1/TNFRSF1B/ABCC2/C	24
GO:0055080	cation homeostasis	24/182	89/749	0,306394427	0,999954951	0,999954951	S100A9/HIF1A/LTF/FPR1/S100A8/CD24/LCN2/TGFB1/BAX/AGT/SLC12A3/FKBP1A/SLAMF8/MT1A/APOE/SLC11A1/MYC/C	24
GO:0014070	response to organic cyclic compound	24/182	111/749	0,796146816	0,999954951	0,999954951	STAT3/HDAC6/COL1A1/NR4A1/ICAM1/STAT1/TGFB1/AHR/CDKN1A/AGT/TNC/EGR1/TLR2/RORC/ABCC2/ERRFI1/TIPARP/	24
GO:1903530	regulation of secretion by cell	24/182	121/749	0,91614891	0,999954951	0,999954951	HIF1A/FCER1G/TNFRSF1A/IL4R/TGFB1/SOCS1/XBP1/IL17RA/AGT/TLR4/APOE/TLR2/MAPK11/TNFRSF1B/IL1R2/CD58/ITG	24
GO:0070661	leukocyte proliferation	24/182	134/749	0,980246593	0,999954951	0,999954951	BCL6/CD24/IMPDH1/PSMB10/JAK3/TGFB1/BAX/AHR/CDKN1A/VCAM1/CD276/TLR4/KITLG/LILRB2/LST1/IL6R/SLC11A1/T	24
GO:0032880	regulation of protein localization	24/182	138/749	0,988105389	0,999954951	0,999954951	HIF1A/FCER1G/HDAC6/IL4R/TGFB1/SOCS1/XBP1/IL17RA/FKBP1A/TLR4/APOE/TLR2/MAPK11/TNFRSF1B/IL1R2/CD58/ITG	24
GO:0052548	regulation of endopeptidase activity	23/182	69/749	0,048685315	0,999954951	0,999954951	SERPINA3/S100A9/LTF/RARRES1/TIMP1/STAT3/S100A8/C3/SLPI/SERPING1/PSMB8/PSME2/BAX/AGT/MYC/TNFSF10/BIR	23
GO:1901652	response to peptide	23/182	72/749	0,076600226	0,999954951	0,999954951	TIMP1/STAT3/SOCS3/COL1A1/NR4A1/ICAM1/JAK3/STAT1/TGFB1/SOCS1/XBP1/VCAM1/STAT6/AGT/EGR1/TLR4/TLR2/AI	23
GO:0009057	macromolecule catabolic process	23/182	75/749	0,113905697	0,999954951	0,999954951	TIMP1/PSMB8/LYVE1/HDAC6/PSME2/PSMB10/TGFB1/XBP1/BAX/LDLR/CTSS/VCAN/MYD88/APOE/SLC11A1/TNFRSF1B/	23
GO:0006873	cellular ion homeostasis	23/182	83/749	0,259959304	0,999954951	0,999954951	S100A9/HIF1A/LTF/FPR1/S100A8/CD24/LCN2/TGFB1/BAX/AGT/FKBP1A/SLAMF8/MT1A/APOE/SLC11A1/MYC/CMKLR1/	23
GO:0042060	wound healing	23/182	86/749	0,329079101	0,999954951	0,999954951	HIF1A/TIMP1/S100A8/SERPING1/FCER1G/EHD3/COL1A1/COL3A1/TGFB1/XBP1/CDKN1A/CD59/TNC/TLR4/APOE/SLC11A	23
GO:0055065	metal ion homeostasis	23/182	86/749	0,329079101	0,999954951	0,999954951	S100A9/HIF1A/LTF/FPR1/S100A8/CD24/LCN2/TGFB1/BAX/AGT/SLC12A3/FKBP1A/MT1A/APOE/SLC11A1/MYC/CMKLR1/	23
GO:0030099	myeloid cell differentiation	23/182	90/749	0,427863591	0,999954951	0,999954951	HIF1A/LTF/BCL6/STAT3/FCER1G/LTBR/RELB/JAK3/STAT1/TGFB1/TLR4/KITLG/TLR2/IRF7/MYC/TRIB1/G6PD/LILRB1/TMEN	23
GO:0051253	negative regulation of RNA metabolic process	23/182	100/749	0,669171384	0,999954951	0,999954951	BCL3/BCL6/STAT3/MUC1/HDAC6/MAF/RELB/STAT1/TGFB1/DNMT1/AHR/STAT6/EGR1/MYD88/IRF7/SLC11A1/RORC/MY	23
GO:0045934	negative regulation of nucleobase-containing compound metab	23/182	104/749	0,749908017	0,999954951	0,999954951	BCL3/BCL6/STAT3/MUC1/HDAC6/MAF/RELB/STAT1/TGFB1/DNMT1/AHR/STAT6/EGR1/MYD88/IRF7/SLC11A1/RORC/MY	23
GO:0002683	negative regulation of immune system process	23/182	112/749	0,870963535	0,999954951	0,999954951	LTF/BCL6/SERPING1/FCER1G/COL3A1/JAK3/IL4R/TGFB1/SOCS1/STAT6/CD59/LDLR/TLR4/KITLG/SLAMF8/LILRB2/LST1/M	23
GO:0046651	lymphocyte proliferation	23/182	127/749	0,973683455	0,999954951	0,999954951	BCL6/CD24/IMPDH1/PSMB10/JAK3/TGFB1/BAX/AHR/CDKN1A/VCAM1/CD276/TLR4/LILRB2/LST1/IL6R/SLC11A1/TNFRSF	23
GO:0032943	mononuclear cell proliferation	23/182	128/749	0,976690442	0,999954951	0,999954951	BCL6/CD24/IMPDH1/PSMB10/JAK3/TGFB1/BAX/AHR/CDKN1A/VCAM1/CD276/TLR4/LILRB2/LST1/IL6R/SLC11A1/TNFRSF	23
GO:0035239	tube morphogenesis	23/182	133/749	0,987635459	0,999954951	0,999954951	HIF1A/JAK1/STAT3/C3/COL4A1/NR4A1/COL3A1/STAT1/TGFB1/XBP1/BAX/AGT/LDLR/TNC/APOE/MYC/TGFBI/ITGB2/TIPA	23
GO:0007159	leukocyte cell-cell adhesion	23/182	139/749	0,994550782	0,999954951	0,999954951	S100A9/BCL6/S100A8/CD24/ICAM1/JAK3/IL4R/NFKBIZ/TGFB1/SOCS1/XBP1/VCAM1/CD276/LILRB2/IL6R/ITGB2/LILRB1/I	23
GO:0002696	positive regulation of leukocyte activation	23/182	141/749	0,995909849	0,999954951	0,999954951	BCL6/CD24/FCER1G/JAK3/IL4R/NFKBIZ/TGFB1/SOCS1/XBP1/CDKN1A/VCAM1/STAT6/CD276/TLR4/LILRB2/IL6R/ITGB2/LI	23
GO:0050867	positive regulation of cell activation	23/182	145/749	0,997741527	0,999954951	0,999954951	BCL6/CD24/FCER1G/JAK3/IL4R/NFKBIZ/TGFB1/SOCS1/XBP1/CDKN1A/VCAM1/STAT6/CD276/TLR4/LILRB2/IL6R/ITGB2/LI	23
GO:0071356	cellular response to tumor necrosis factor	22/182	79/749	0,257904496	0,999954951	0,999954951	PSMB8/TNFRSF1A/GBP2/LCN2/KRT8/TLBR/COL1A1/PSME2/ICAM1/PSMB10/STAT1/VCAM1/TNFRSF1B/ABCC2/CD58/BIF	22
GO:0060284	regulation of cell development	22/182	80/749	0,28071862	0,999954951	0,999954951	S100A9/HIF1A/LTF/BCL6/STAT3/TNFRSF1A/MME/COL3A1/TGFB1/AGT/LDLR/SLAMF8/APOE/TLR2/TNFRSF1B/TRIB1/G6P	22
GO:0030003	cellular cation homeostasis	22/182	82/749	0,328417862	0,999954951	0,999954951	S100A9/HIF1A/LTF/FPR1/S100A8/CD24/LCN2/TGFB1/BAX/AGT/FKBP1A/SLAMF8/MT1A/APOE/SLC11A1/MYC/CMKLR1/C	22
GO:0006959	humoral immune response	22/182	85/749	0,403646751	0,999954951	0,999954951	S100A9/LTF/BCL3/C1QB/C1S/S100A8/C3/SLPI/SERPING1/C9/CFB/LCN2/C1QA/PSMB10/CD59/MASP1/CXCL1/SLC11A1/S	22
GO:0051347	positive regulation of transferase activity	22/182	92/749	0,581460912	0,999954951	0,999954951	LTF/PLAAT4/FPR1/CD24/FCGR1A/TGFB1/SOCS1/CDKN1A/AGT/EGR1/TLR4/KITLG/MAPK11/IL6R/SLC11A1/MYC/S100A12	22
GO:2000113	negative regulation of cellular macromolecule biosynthetic proc	22/182	96/749	0,674525257	0,999954951	0,999954951	BCL3/BCL6/STAT3/MUC1/HDAC6/MAF/RELB/JAK3/STAT1/TGFB1/DNMT1/AHR/STAT6/EGR1/IRF7/RORC/MYC/EPO/ATF3	22
GO:0002449	lymphocyte mediated immunity	22/182	113/749	0,924344271	0,999954951	0,999954951	BCL3/C1QB/C1S/BCL6/C3/SERPING1/C9/FCER1G/IL1R1/ICAM1/C1QA/IL4R/TGFB1/STAT6/IRF7/SLC11A1/PVR/TNFRSF1B/	22
GO:0043408	regulation of MAPK cascade	22/182	117/749	0,950827553	0,999954951	0,999954951	FPR1/CD24/LTBR/ICAM1/TGFB1/TLR4/KITLG/APOE/MAPK11/MYC/S100A12/FGD2/TRIB1/ERRFI1/NOX4/C5AR1/FN1/GDI	22
GO:0051272	positive regulation of cellular component movement	22/182	120/749	0,965133475	0,999954951	0,999954951	HIF1A/SOD2/BCL6/STAT3/ADAMTS1/IL1R1/HDAC6/COL1A1/ICAM1/TGFB1/XBP1/CXCL16/AGT/KITLG/IL6R/CMKLR1/NOX	22
GO:0007167	enzyme linked receptor protein signaling pathway	22/182	121/749	0,969028437	0,999954951	0,999954951	HIF1A/STAT3/COL4A1/SOST/SOCS3/COL1A1/COL3A1/JAK3/TGFB1/SOCS1/STAT6/AGT/EGR1/FKBP1A/MAPK11/CD8B/ER	22
GO:0070201	regulation of establishment of protein localization	22/182	121/749	0,969028437	0,999954951	0,999954951	HIF1A/HDAC6/IL4R/TGFB1/SOCS1/XBP1/IL17RA/TLR4/APOE/TLR2/MAPK11/TNFRSF1B/IL1R2/CD58/ITGB2/FN1/LILRB1/C	22
GO:0090087	regulation of peptide transport	22/182	124/749	0,978536902	0,999954951	0,999954951	HIF1A/S100A8/IL4R/TGFB1/SOCS1/XBP1/IL17RA/TLR4/APOE/TLR2/MAPK11/TNFRSF1B/IL1R2/CD58/ITGB2/FN1/LILRB1/	22
GO:0006812	cation transport	21/182	79/749	0,352845259	0,999954951	0,999954951	LTF/FCER1G/EHD3/LCN2/ICAM1/TGFB1/BAX/AGT/SLC12A3/FKBP1A/CTSS/LILRB2/SLC11A1/ABCC2/G6PD/LILRB1/EPO/SI	21

GO:0006875	cellular metal ion homeostasis	21/182	79/749	0,352845259	0,999954951	0,999954951	S100A9/HIF1A/LTF/FPR1/S100A8/CD24/LCN2/TGFB1/BAX/AGT/FKBP1A/MT1A/APOE/SLC11A1/MYC/CMKLR1/C5AR1/TN	21
GO:0040007	growth	21/182	88/749	0,585845067	0,999954951	0,999954951	S100A9/HIF1A/BCL6/STAT3/S100A8/SOCS3/HDAC6/TGFB1/SOCS1/CXCL16/CDKN1A/AGT/TNC/MT1A/APOE/MAPK11/G6	21
GO:1902679	negative regulation of RNA biosynthetic process	21/182	94/749	0,72275454	0,999954951	0,999954951	BCL3/BCL6/STAT3/MUC1/HDAC6/MAF/RELB/STAT1/TGFB1/DNMT1/AHR/STAT6/EGR1/IRF7/RORC/MYC/EPO/ATF3/GAT	21
GO:0002791	regulation of peptide secretion	21/182	105/749	0,89241512	0,999954951	0,999954951	HIF1A/S100A8/IL4R/TGFB1/SOCS1/XBP1/IL17RA/TLR4/APOE/TLR2/MAPK11/TNFRSF1B/IL1R2/CD58/FN1/LILRB1/CD14/C	21
GO:0043068	positive regulation of programmed cell death	21/182	107/749	0,911944916	0,999954951	0,999954951	S100A9/SOD2/BCL6/S100A8/TNFRSF1A/LTBR/HDAC6/TGFB1/BAX/CDKN1A/AGT/TNFRSF1B/MYC/FGD2/TNFSF10/NOX4/	21
GO:0002764	immune response-regulating signaling pathway	21/182	110/749	0,935809618	0,999954951	0,999954951	FPR1/CD24/FCER1G/PSMB8/MUC1/PSME2/PSMB10/RELB/FCGR1A/NFKBIZ/BAX/FCGR2A/CD276/LILRB2/CMKLR1/C5AR	21
GO:0002768	immune response-regulating cell surface receptor signaling path	21/182	110/749	0,935809618	0,999954951	0,999954951	FPR1/CD24/FCER1G/PSMB8/MUC1/PSME2/PSMB10/RELB/FCGR1A/NFKBIZ/BAX/FCGR2A/CD276/LILRB2/CMKLR1/C5AR	21
GO:0043933	protein-containing complex subunit organization	21/182	117/749	0,971393384	0,999954951	0,999954951	SOD2/C9/FCER1G/SOST/TNFRSF1A/EHD3/LCN2/HDAC6/COL1A1/ICAM1/TGFB1/BAX/AGT/CD59/FKBP1A/TLR4/APOE/M	21
GO:0030335	positive regulation of cell migration	21/182	118/749	0,974711546	0,999954951	0,999954951	HIF1A/SOD2/STAT3/ADAMTS1/IL1R1/HDAC6/COL1A1/ICAM1/TGFB1/XBP1/CXCL16/AGT/KITLG/IL6R/CMKLR1/NOX4/C5	21
GO:2000147	positive regulation of cell motility	21/182	119/749	0,977687472	0,999954951	0,999954951	HIF1A/SOD2/STAT3/ADAMTS1/IL1R1/HDAC6/COL1A1/ICAM1/TGFB1/XBP1/CXCL16/AGT/KITLG/IL6R/CMKLR1/NOX4/C5	21
GO:0051223	regulation of protein transport	21/182	120/749	0,980350441	0,999954951	0,999954951	HIF1A/IL4R/TGFB1/SOCS1/XBP1/IL17RA/TLR4/APOE/TLR2/MAPK11/TNFRSF1B/IL1R2/CD58/ITGB2/FN1/LILRB1/CD14/G	21
GO:0040017	positive regulation of locomotion	21/182	121/749	0,982728062	0,999954951	0,999954951	HIF1A/SOD2/STAT3/ADAMTS1/IL1R1/HDAC6/COL1A1/ICAM1/TGFB1/XBP1/CXCL16/AGT/KITLG/IL6R/CMKLR1/NOX4/C5	21
GO:0030098	lymphocyte differentiation	21/182	125/749	0,989877964	0,999954951	0,999954951	BCL3/BCL6/STAT3/FCER1G/RELB/JAK3/IL4R/NFKBIZ/TGFB1/SOCS1/XBP1/BAX/VCAM1/STAT6/EGR1/SLAMF8/LILRB2/RO	21
GO:1903037	regulation of leukocyte cell-cell adhesion	21/182	129/749	0,994236893	0,999954951	0,999954951	BCL6/CD24/ICAM1/JAK3/IL4R/NFKBIZ/TGFB1/SOCS1/XBP1/VCAM1/CD276/LILRB2/IL6R/ITGB2/LILRB1/EPO/GATA3/ADO	21
GO:0051251	positive regulation of lymphocyte activation	21/182	131/749	0,995697008	0,999954951	0,999954951	BCL6/CD24/JAK3/IL4R/NFKBIZ/TGFB1/SOCS1/XBP1/CDKN1A/VCAM1/STAT6/CD276/TLR4/LILRB2/IL6R/LILRB1/EPO/GAT	21
GO:0045785	positive regulation of cell adhesion	21/182	132/749	0,996291463	0,999954951	0,999954951	BCL6/CD24/ICAM1/JAK3/IL4R/NFKBIZ/TGFB1/SOCS1/XBP1/VCAM1/CD276/LILRB2/IL6R/ITGB2/FN1/LILRB1/EPO/GATA3/	21
GO:0022407	regulation of cell-cell adhesion	21/182	143/749	0,999352706	0,999954951	0,999954951	BCL6/CD24/ICAM1/JAK3/IL4R/NFKBIZ/TGFB1/SOCS1/XBP1/VCAM1/CD276/LILRB2/IL6R/ITGB2/LILRB1/EPO/GATA3/ADO	21
GO:0032870	cellular response to hormone stimulus	20/182	63/749	0,101162959	0,999954951	0,999954951	STAT3/SOST/SOCS3/HDAC6/NR4A1/ICAM1/JAK3/STAT1/TGFB1/SOCS1/XBP1/STAT6/AGT/TNC/CTSS/RORC/ABCC2/ERRF	20
GO:0042742	defense response to bacterium	20/182	67/749	0,167715127	0,999954951	0,999954951	S100A9/LTF/BCL3/S100A8/SLPI/FCER1G/TNFRSF1A/GBP2/LCN2/TLR4/SLAMF8/MYD88/TLR2/IL6R/SLC11A1/S100A12/TB	20
GO:0007417	central nervous system development	20/182	77/749	0,405337331	0,999954951	0,999954951	ALDH3A2/HIF1A/STAT3/S100A8/C3/COL4A1/COL3A1/C1QA/TGFB1/BAX/LDLR/TLR4/VCAN/TLR2/TNFRSF1B/C5AR1/G6P	20
GO:0034341	response to interferon-gamma	20/182	77/749	0,405337331	0,999954951	0,999954951	JAK1/IFITM3/IFITM2/IFITM1/IFNGR2/SOCS3/GBP2/ICAM1/FCGR1A/STAT1/SOCS1/CXCL16/VCAM1/IFI30/TLR4/TLR2/IRF	20
GO:0050663	cytokine secretion	20/182	81/749	0,512525268	0,999954951	0,999954951	ITGB6/IL4R/SOCS1/XBP1/IL17RA/AGT/TLR4/TLR2/MAPK11/TNFRSF1B/S100A12/IL1R2/CD58/FN1/LILRB1/CD14/GATA3/I	20
GO:0045860	positive regulation of protein kinase activity	20/182	82/749	0,538918939	0,999954951	0,999954951	LTF/FPR1/CD24/FCGR1A/TGFB1/SOCS1/CDKN1A/AGT/EGR1/TLR4/KITLG/MAPK11/IL6R/SLC11A1/S100A12/FGD2/NOX4/	20
GO:0033674	positive regulation of kinase activity	20/182	86/749	0,639702389	0,999954951	0,999954951	LTF/FPR1/CD24/FCGR1A/TGFB1/SOCS1/CDKN1A/AGT/EGR1/TLR4/KITLG/MAPK11/IL6R/SLC11A1/S100A12/FGD2/NOX4/	20
GO:0001818	negative regulation of cytokine production	20/182	87/749	0,663267193	0,999954951	0,999954951	LTF/BCL3/BCL6/CD24/RELB/JAK3/TGFB1/SIGIRR/TLR4/SLC11A1/IL1R2/TBK1/CMKLR1/ERRFI1/FN1/LILRB1/GATA3/NFKB1	20
GO:0007049	cell cycle	20/182	92/749	0,768258798	0,999954951	0,999954951	BCL6/STAT3/ADAMTS1/PSMB8/MUC1/MAPK13/ABCB1/NR4A1/PSME2/PSMB10/TGFB1/BAX/AHR/CDKN1A/MYC/PSME1	20
GO:0045596	negative regulation of cell differentiation	20/182	92/749	0,768258798	0,999954951	0,999954951	LTF/BCL6/STAT3/COL3A1/JAK3/STAT1/IL4R/TGFB1/SOCS1/XBP1/DNMT1/LDLR/TLR4/APOE/MYC/TRIB1/G6PD/LILRB1/TN	20
GO:0045892	negative regulation of transcription, DNA-templated	20/182	92/749	0,768258798	0,999954951	0,999954951	BCL3/BCL6/STAT3/MUC1/HDAC6/MAF/RELB/STAT1/TGFB1/DNMT1/AHR/STAT6/EGR1/IRF7/RORC/MYC/EPO/ATF3/GAT	20
GO:1903507	negative regulation of nucleic acid-templated transcription	20/182	93/749	0,7864483	0,999954951	0,999954951	BCL3/BCL6/STAT3/MUC1/HDAC6/MAF/RELB/STAT1/TGFB1/DNMT1/AHR/STAT6/EGR1/IRF7/RORC/MYC/EPO/ATF3/GAT	20
GO:0050708	regulation of protein secretion	20/182	101/749	0,897648699	0,999954951	0,999954951	HIF1A/IL4R/TGFB1/SOCS1/XBP1/IL17RA/TLR4/APOE/TLR2/MAPK11/TNFRSF1B/IL1R2/CD58/FN1/LILRB1/CD14/GATA3/IF	20
GO:0048514	blood vessel morphogenesis	20/182	107/749	0,94619364	0,999954951	0,999954951	HIF1A/JAK1/STAT3/C3/COL4A1/NR4A1/COL3A1/STAT1/TGFB1/XBP1/BAX/AGT/LDLR/APOE/TGFBI/ITGB2/TIPARP/C5AR1	20
GO:1903039	positive regulation of leukocyte cell-cell adhesion	20/182	108/749	0,952013304	0,999954951	0,999954951	BCL6/CD24/ICAM1/JAK3/IL4R/NFKBIZ/TGFB1/SOCS1/XBP1/VCAM1/CD276/LILRB2/IL6R/ITGB2/LILRB1/EPO/GATA3/TNFS	20
GO:0065003	protein-containing complex assembly	20/182	113/749	0,973730717	0,999954951	0,999954951	SOD2/C9/FCER1G/SOST/TNFRSF1A/EHD3/LCN2/HDAC6/COL1A1/ICAM1/TGFB1/BAX/CD59/FKBP1A/TLR4/APOE/MYC/BI	20
GO:0022409	positive regulation of cell-cell adhesion	20/182	114/749	0,97685027	0,999954951	0,999954951	BCL6/CD24/ICAM1/JAK3/IL4R/NFKBIZ/TGFB1/SOCS1/XBP1/VCAM1/CD276/LILRB2/IL6R/ITGB2/LILRB1/EPO/GATA3/TNFS	20
GO:0006935	chemotaxis	20/182	119/749	0,988043463	0,999954951	0,999954951	S100A9/FPR1/S100A8/FCER1G/NR4A1/TGFB1/CXCL16/VCAM1/IL17RA/SLAMF8/CXCL1/IL6R/S100A12/CMKLR1/ITGB2/C	20
GO:0042330	taxis	20/182	119/749	0,988043463	0,999954951	0,999954951	S100A9/FPR1/S100A8/FCER1G/NR4A1/TGFB1/CXCL16/VCAM1/IL17RA/SLAMF8/CXCL1/IL6R/S100A12/CMKLR1/ITGB2/C	20
GO:0050863	regulation of T cell activation	20/182	134/749	0,998742263	0,999954951	0,999954951	BCL6/CD24/JAK3/IL4R/NFKBIZ/TGFB1/SOCS1/XBP1/VCAM1/CD276/LILRB2/IL6R/TNFRSF1B/LILRB1/EPO/GATA3/ADORA2	20
GO:0031400	negative regulation of protein modification process	19/182	59/749	0,096251939	0,999954951	0,999954951	SOCS3/TGFB1/SOCS1/DNMT1/BAX/CDKN1A/FKBP1A/TLR4/APOE/MYC/TRIB1/ERRFI1/G6PD/ATF3/ADORA2A/ISG15/RGN	19
GO:0019752	carboxylic acid metabolic process	19/182	61/749	0,127103701	0,999954951	0,999954951	ALDH3A2/HIF1A/STAT3/C3/PSMB8/LYVE1/TNFRSF1A/PSME2/PSMB10/TGFB1/XBP1/ALOX5/VCAN/TLR2/ABCC2/NOX4/P	19
GO:0043436	oxoacid metabolic process	19/182	61/749	0,127103701	0,999954951	0,999954951	ALDH3A2/HIF1A/STAT3/C3/PSMB8/LYVE1/TNFRSF1A/PSME2/PSMB10/TGFB1/XBP1/ALOX5/VCAN/TLR2/ABCC2/NOX4/P	19
GO:0006082	organic acid metabolic process	19/182	63/749	0,163251105	0,999954951	0,999954951	ALDH3A2/HIF1A/STAT3/C3/PSMB8/LYVE1/TNFRSF1A/PSME2/PSMB10/TGFB1/XBP1/ALOX5/VCAN/TLR2/ABCC2/NOX4/P	19
GO:0032102	negative regulation of response to external stimulus	19/182	70/749	0,325458046	0,999954951	0,999954951	LTF/SERPING1/SOCS3/TNFRSF1A/CDKN1A/SIGIRR/LDLR/SLAMF8/APOE/TNFRSF1B/IL1R2/TRIB1/LILRB1/SERPINE1/GATA	19
GO:1901565	organonitrogen compound catabolic process	19/182	71/749	0,351926606	0,999954951	0,999954951	TIMP1/PSMB8/LYVE1/HDAC6/PSME2/PSMB10/TGFB1/XBP1/LDLR/CTSS/VCAN/APOE/TNFRSF1B/TRIB1/TIPARP/PSME1/I	19
GO:0009894	regulation of catabolic process	19/182	75/749	0,46111304	0,999954951	0,999954951	HIF1A/TIMP1/STAT3/PSMB8/HDAC6/PSME2/PSMB10/XBP1/BAX/LDLR/MYD88/APOE/SLC11A1/TNFRSF1B/TBK1/TRIB1/T	19
GO:0018108	peptidyl-tyrosine phosphorylation	19/182	87/749	0,755749053	0,999954951	0,999954951	JAK1/STAT3/CD24/SOCS3/TNFRSF1A/ICAM1/FCGR1A/JAK3/TGFB1/SOCS1/AGT/KITLG/IL6R/ITGB2/ERRFI1/NOX4/EPO/IL	19
GO:0018212	peptidyl-tyrosine modification	19/182	87/749	0,755749053	0,999954951	0,999954951	JAK1/STAT3/CD24/SOCS3/TNFRSF1A/ICAM1/FCGR1A/JAK3/TGFB1/SOCS1/AGT/KITLG/IL6R/ITGB2/ERRFI1/NOX4/EPO/IL	19
GO:0009615	response to virus	19/182	89/749	0,79312567	0,999954951	0,999954951	BCL3/IFITM3/IFITM2/IFITM1/IFNGR2/LCN2/IFNAR2/STAT1/IRF7/MAPK11/TBK1/ISG20/BIRC3/LILRB1/IL10RB/IFIT1/GATA	19
GO:1901566	organonitrogen compound biosynthetic process	19/182	96/749	0,89252379	0,999954951	0,999954951	ALDH3A2/HIF1A/BCL3/STAT3/NNMT/MUC1/TNFRSF1A/IMPDH1/JAK3/TGFB1/EGR1/TLR4/VCAN/APOE/TBK1/EPO/GATA	19
GO:1902105	regulation of leukocyte differentiation	19/182	98/749	0,912660228	0,999954951	0,999954951	LTF/BCL6/JAK3/IL4R/NFKBIZ/TGFB1/SOCS1/XBP1/TLR4/KITLG/SLAMF8/LILRB2/IRF7/MYC/TRIB1/LILRB1/TMEM178A/GA	19
GO:0043065	positive regulation of apoptotic process	19/182	105/749	0,960518576	0,999954951	0,999954951	S100A9/SOD2/BCL6/S100A8/TNFRSF1A/LTBR/TGFB1/BAX/AGT/TNFRSF1B/MYC/FGD2/TNFSF10/NOX4/LILRB1/ATF3/CAS	19
GO:0044265	cellular macromolecule catabolic process	18/182	50/749	0,037562459	0,999954951	0,999954951	PSMB8/HDAC6/PSME2/PSMB10/XBP1/BAX/LDLR/CTSS/MYD88/APOE/SLC11A1/TNFRSF1B/TRIB1/ISG20/PSME1/ISG15/	18
GO:0070647	protein modification by small protein conjugation or removal	18/182	52/749	0,05505828	0,999954951	0,999954951	HIF1A/PSMB8/SOCS3/ASB15/HDAC6/PSME2/PSMB10/SOCS1/EGR1/FKBP1A/MYC/BIRC3/PSME1/DCAF12/GATA3/KLHL1	18
GO:0050729	positive regulation of inflammatory response	18/182	60/749	0,178625544	0,999954951	0,999954951	OSMR/S100A9/S100A8/C3/FCER1G/MAPK13/TNFRSF1A/NFKBIZ/IL17RA/AGT/LDLR/TLR4/MYD88/TLR2/S100A12/SERPIN	18
GO:0030001	metal ion transport	18/182	66/749	0,324065769	0,999954951	0,999954951	LTF/EHD3/LCN2/ICAM1/TGFB1/BAX/AGT/SLC12A3/FKBP1A/LILRB2/SLC11A1/ABCC2/G6PD/LILRB1/EPO/ADORA2A/RGN/	18
GO:0031329	regulation of cellular catabolic process	18/182	66/749	0,324065769	0,999954951	0,999954951	HIF1A/TIMP1/STAT3/PSMB8/HDAC6/PSME2/PSMB10/XBP1/BAX/LDLR/MYD88/APOE/SLC11A1/TNFRSF1B/TBK1/TRIB1/	18
GO:0040008	regulation of growth	18/182	66/749	0,324065769	0,999954951	0,999954951	S100A9/HIF1A/BCL6/STAT3/S100A8/SOCS3/TGFB1/SOCS1/CXCL16/CDKN1A/AGT/MT1A/APOE/MAPK11/G6PD/FN1/GDF	18
GO:0002819	regulation of adaptive immune response	18/182	77/749	0,626283899	0,999954951	0,999954951	BCL6/C3/FCER1G/IL1R1/JAK3/IL4R/NFKBIZ/TGFB1/STAT6/IRF7/IL6R/SLC11A1/PVR/TNFRSF1B/LILRB1/GATA3/IL6ST/PTPN	18
GO:0010035	response to inorganic substance	18/182	78/749	0,651331187	0,999954951	0,999954951	HIF1A/SOD2/S100A8/MAPK13/LCN2/KRT8/HDAC6/COL1A1/ICAM1/C1QA/STAT1/VCAM1/STAT6/MT1A/ABCC2/G6PD/C	18
GO:0061061	muscle structure development	18/182	79/749	0,675530939	0,999954951	0,999954951	SOD2/KRT8/COL3A1/IL4R/TGFB1/XBP1/DNMT1/AGT/EGR1/FKBP1A/MAPK11/KRT19/NOX4/G6PD/MYOM2/GDF15/ATF3	18

GO:0071222	cellular response to lipopolysaccharide	18/182	80/749	0,698818394	0,999954951	0,999954951	LTF/LCN2/ICAM1/TGFB1/XBP1/SIGIRR/LY96/TLR4/CXCL1/TLR2/LILRB2/TNFRSF1B/ABCC2/TRIB1/LILRB1/CD14/SERPINE1/	18
GO:0033043	regulation of organelle organization	18/182	81/749	0,72114058	0,999954951	0,999954951	S100A9/HIF1A/BCL6/S100A8/MUC1/EHD3/HDAC6/ICAM1/TGFB1/DNMT1/BAX/LILRB2/MYC/ARHGDIB/TNFSF10/NOX4/(	18
GO:0071219	cellular response to molecule of bacterial origin	18/182	81/749	0,72114058	0,999954951	0,999954951	LTF/LCN2/ICAM1/TGFB1/XBP1/SIGIRR/LY96/TLR4/CXCL1/TLR2/LILRB2/TNFRSF1B/ABCC2/TRIB1/LILRB1/CD14/SERPINE1/	18
GO:0051090	regulation of DNA-binding transcription factor activity	18/182	83/749	0,76273472	0,999954951	0,999954951	S100A9/LTF/STAT3/S100A8/ICAM1/TGFB1/AGT/SIGIRR/NFKB2/TLR4/MYD88/TLR2/MAPK11/S100A12/CMKLR1/TRIB1/IT	18
GO:0071216	cellular response to biotic stimulus	18/182	85/749	0,800112984	0,999954951	0,999954951	LTF/LCN2/ICAM1/TGFB1/XBP1/SIGIRR/LY96/TLR4/CXCL1/TLR2/LILRB2/TNFRSF1B/ABCC2/TRIB1/LILRB1/CD14/SERPINE1/	18
GO:0051047	positive regulation of secretion	18/182	89/749	0,862191039	0,999954951	0,999954951	HIF1A/S100A8/FCER1G/IL4R/TGFB1/XBP1/IL17RA/AGT/TLR4/TLR2/MAPK11/CD58/ITGB2/CD14/GATA3/IFNGR1/ADORA:	18
GO:0002429	immune response-activating cell surface receptor signaling path	18/182	94/749	0,917860992	0,999954951	0,999954951	FPR1/FCER1G/PSMB8/MUC1/PSME2/PSMB10/RELB/FCGR1A/NFKBIZ/BAX/FCGR2A/CD276/CMKLR1/C5AR1/PSME1/GAT	18
GO:0002757	immune response-activating signal transduction	18/182	94/749	0,917860992	0,999954951	0,999954951	FPR1/FCER1G/PSMB8/MUC1/PSME2/PSMB10/RELB/FCGR1A/NFKBIZ/BAX/FCGR2A/CD276/CMKLR1/C5AR1/PSME1/GAT	18
GO:0050870	positive regulation of T cell activation	18/182	102/749	0,968003089	0,999954951	0,999954951	BCL6/CD24/JAK3/IL4R/NFKBIZ/TGFB1/SOCS1/XBP1/VCAM1/CD276/LILRB2/IL6R/LILRB1/EPO/GATA3/TNFSF14/IL6ST/PTF	18
GO:0070663	regulation of leukocyte proliferation	18/182	111/749	0,990527888	0,999954951	0,999954951	BCL6/CD24/JAK3/TGFB1/AHR/CDKN1A/VCAM1/CD276/TLR4/KITLG/LILRB2/LST1/IL6R/TNFRSF1B/LILRB1/EPO/IL6ST/PTP	18
GO:0010563	negative regulation of phosphorus metabolic process	17/182	53/749	0,116099207	0,999954951	0,999954951	STAT3/SOCS3/TGFB1/SOCS1/BAX/CDKN1A/FKBP1A/TLR4/APOE/MYC/TRIB1/ERRFI1/ATF3/ADORA2A/RGN/RNF149/PTPN	17
GO:0045936	negative regulation of phosphate metabolic process	17/182	53/749	0,116099207	0,999954951	0,999954951	STAT3/SOCS3/TGFB1/SOCS1/BAX/CDKN1A/FKBP1A/TLR4/APOE/MYC/TRIB1/ERRFI1/ATF3/ADORA2A/RGN/RNF149/PTPN	17
GO:0034220	ion transmembrane transport	17/182	58/749	0,218726482	0,999954951	0,999954951	EHD3/ABCB1/TGFB1/BAX/AGT/APOL1/SLC12A3/FKBP1A/CTSS/SLC11A1/ABCC2/TAP2/G6PD/EPO/TAP1/RGN/PTPN6	17
GO:0046677	response to antibiotic	17/182	60/749	0,268685292	0,999954951	0,999954951	JAK1/STAT3/S100A8/MAPK13/LCN2/HDAC6/COL1A1/ICAM1/STAT1/AHR/VCAM1/STAT6/TNC/EGR1/G6PD/CD14/GATA3	17
GO:0070555	response to interleukin-1	17/182	60/749	0,268685292	0,999954951	0,999954951	HIF1A/PSMB8/IL1R1/MAPK13/GBP2/LCN2/PSME2/ICAM1/PSMB10/EGR1/MYD88/MAPK11/IL1R2/ABCC2/PSME1/EPO/N	17
GO:0050878	regulation of body fluid levels	17/182	61/749	0,295148289	0,999954951	0,999954951	HIF1A/SERPING1/FCER1G/EHD3/COL1A1/COL3A1/XBP1/CD59/TLR4/APOE/SERPINE1/GATA3/SLC22A2/ADORA2A/THBD/	17
GO:0071407	cellular response to organic cyclic compound	17/182	66/749	0,436512953	0,999954951	0,999954951	STAT3/HDAC6/COL1A1/NR4A1/ICAM1/STAT1/TGFB1/AHR/TNC/EGR1/RORC/ABCC2/ERRFI1/TIPARP/NOX4/IFIT1/NFKB1	17
GO:0046907	intracellular transport	17/182	69/749	0,522998018	0,999954951	0,999954951	HIF1A/STAT3/CD24/FCER1G/EHD3/HDAC6/IL4R/TGFB1/BAX/CDKN1A/AGT/CD59/LDLR/APOE/TAP2/ITGB2/TAP1	17
GO:0002285	lymphocyte activation involved in immune response	17/182	70/749	0,551209574	0,999954951	0,999954951	BCL3/BCL6/STAT3/FCER1G/ICAM1/RELB/JAK3/IL4R/NFKBIZ/TGFB1/XBP1/STAT6/TLR4/SLC11A1/RORC/LILRB1/GATA3	17
GO:0050730	regulation of peptidyl-tyrosine phosphorylation	17/182	70/749	0,551209574	0,999954951	0,999954951	STAT3/CD24/SOCS3/TNFRSF1A/ICAM1/FCGR1A/TGFB1/SOCS1/AGT/KITLG/IL6R/ITGB2/ERRFI1/NOX4/EPO/IL6ST/PTPN6	17
GO:0072503	cellular divalent inorganic cation homeostasis	17/182	70/749	0,551209574	0,999954951	0,999954951	S100A9/FPR1/S100A8/CD24/TGFB1/BAX/AGT/FKBP1A/MT1A/APOE/SLC11A1/CMKLR1/C5AR1/TMEM178A/EPO/RGN/PT	17
GO:0072507	divalent inorganic cation homeostasis	17/182	71/749	0,578893309	0,999954951	0,999954951	S100A9/FPR1/S100A8/CD24/TGFB1/BAX/AGT/FKBP1A/MT1A/APOE/SLC11A1/CMKLR1/C5AR1/TMEM178A/EPO/RGN/PT	17
GO:0051726	regulation of cell cycle	17/182	77/749	0,728294671	0,999954951	0,999954951	BCL6/STAT3/ADAMTS1/PSMB8/MUC1/NR4A1/PSME2/PSMB10/TGFB1/BAX/CDKN1A/MYC/PSME1/LILRB1/GATA3/KLHL:	17
GO:0003006	developmental process involved in reproduction	17/182	80/749	0,789324712	0,999954951	0,999954951	HIF1A/BCL6/ADAMTS1/SOCS3/MME/KRT8/ICAM1/TGFB1/BAX/TNC/KITLG/KRT19/TNFSF10/BIRC3/TIPARP/GATA3/RGN	17
GO:0007169	transmembrane receptor protein tyrosine kinase signaling pathw	17/182	80/749	0,789324712	0,999954951	0,999954951	HIF1A/STAT3/COL4A1/SOCS3/COL1A1/JAK3/TGFB1/SOCS1/STAT6/AGT/MAPK11/CD8B/ERRFI1/TIPARP/GDF15/GATA3/P	17
GO:0030182	neuron differentiation	17/182	85/749	0,868886111	0,999954951	0,999954951	S100A9/HIF1A/BCL6/STAT3/C3/HDAC6/C1QA/XBP1/VCAM1/AGT/TNC/APOE/LST1/FN1/EPO/GATA3/ADORA2A	17
GO:1903532	positive regulation of secretion by cell	17/182	86/749	0,881619707	0,999954951	0,999954951	HIF1A/FCER1G/IL4R/TGFB1/XBP1/IL17RA/AGT/TLR4/TLR2/MAPK11/CD58/ITGB2/CD14/GATA3/IFNGR1/ADORA2A/IL17R	17
GO:0070848	response to growth factor	17/182	93/749	0,945749452	0,999954951	0,999954951	HIF1A/SOST/HDAC6/COL1A1/NR4A1/COL3A1/TGFB1/VCAM1/AGT/TNC/EGR1/TNFRSF1B/MYC/ERRFI1/NOX4/GDF15/GA	17
GO:0050670	regulation of lymphocyte proliferation	17/182	107/749	0,991688356	0,999954951	0,999954951	BCL6/CD24/JAK3/TGFB1/AHR/CDKN1A/VCAM1/CD276/TLR4/LILRB2/LST1/IL6R/TNFRSF1B/LILRB1/EPO/IL6ST/PTPN6	17
GO:0032944	regulation of mononuclear cell proliferation	17/182	108/749	0,992837741	0,999954951	0,999954951	BCL6/CD24/JAK3/TGFB1/AHR/CDKN1A/VCAM1/CD276/TLR4/LILRB2/LST1/IL6R/TNFRSF1B/LILRB1/EPO/IL6ST/PTPN6	17
GO:0042326	negative regulation of phosphorylation	16/182	47/749	0,079110846	0,999954951	0,999954951	STAT3/SOCS3/TGFB1/SOCS1/BAX/CDKN1A/TLR4/APOE/MYC/TRIB1/ERRFI1/ATF3/ADORA2A/RGN/RNF149/PTPN6	16
GO:0051346	negative regulation of hydrolase activity	16/182	50/749	0,127587049	0,999954951	0,999954951	SERPINA3/LTF/RARRES1/TIMP1/C3/SLPI/SERPING1/AGT/FKBP1A/BIRC3/IFIT1/SERPINE1/ADORA2A/TNFSF14/RGN/PLAU	16
GO:0030163	protein catabolic process	16/182	54/749	0,213960399	0,999954951	0,999954951	TIMP1/PSMB8/HDAC6/PSME2/PSMB10/XBP1/LDLR/CTSS/APOE/TNFRSF1B/TRIB1/TIPARP/PSME1/ISG15/RGN/RNF149	16
GO:0071347	cellular response to interleukin-1	16/182	55/749	0,239000303	0,999954951	0,999954951	HIF1A/PSMB8/IL1R1/MAPK13/GBP2/LCN2/PSME2/ICAM1/PSMB10/EGR1/MYD88/MAPK11/IL1R2/ABCC2/PSME1/NFKB1	16
GO:0071496	cellular response to external stimulus	16/182	57/749	0,292402455	0,999954951	0,999954951	TNFRSF1A/LCN2/LTBR/COL1A1/ICAM1/TGFB1/XBP1/CDKN1A/VCAM1/AGT/TNC/TLR4/MYD88/ATF3/TNFSF14/NFKB1	16
GO:0007249	I-kappaB kinase/NF-kappaB signaling	16/182	59/749	0,349232704	0,999954951	0,999954951	LTF/BCL3/TNFRSF1A/LTBR/RELB/STAT1/LY96/FKBP1A/TLR4/MYD88/TLR2/S100A12/TBK1/TNFSF10/BIRC3/CD14	16
GO:1901214	regulation of neuron death	16/182	59/749	0,349232704	0,999954951	0,999954951	HIF1A/SOD2/STAT3/C1QA/BAX/EGR1/TLR4/APOE/TNFRSF1B/TBK1/ITGB2/C5AR1/G6PD/EPO/GATA3/ADORA2A	16
GO:0070997	neuron death	16/182	61/749	0,408166897	0,999954951	0,999954951	HIF1A/SOD2/STAT3/C1QA/BAX/EGR1/TLR4/APOE/TNFRSF1B/TBK1/ITGB2/C5AR1/G6PD/EPO/GATA3/ADORA2A	16
GO:0043269	regulation of ion transport	16/182	63/749	0,46783079	0,999954951	0,999954951	EHD3/ABCB1/ICAM1/TGFB1/BAX/AGT/FKBP1A/CTSS/APOE/LILRB2/G6PD/LILRB1/EPO/ADORA2A/RGN/PTPN6	16
GO:0050767	regulation of neurogenesis	16/182	65/749	0,526893558	0,999954951	0,999954951	S100A9/HIF1A/BCL6/STAT3/MME/COL3A1/TGFB1/AGT/LDLR/APOE/TLR2/TNFRSF1B/FN1/EPO/GATA3/IL6ST	16
GO:0051960	regulation of nervous system development	16/182	68/749	0,611765653	0,999954951	0,999954951	S100A9/HIF1A/BCL6/STAT3/MME/COL3A1/TGFB1/AGT/LDLR/APOE/TLR2/TNFRSF1B/FN1/EPO/GATA3/IL6ST	16
GO:0070482	response to oxygen levels	16/182	68/749	0,611765653	0,999954951	0,999954951	HIF1A/CD24/PSMB8/COL1A1/PSME2/ICAM1/PSMB10/TGFB1/CDKN1A/VCAM1/EGR1/TLR2/MYC/NOX4/PSME1/EPO	16
GO:0002822	regulation of adaptive immune response based on somatic recoı	16/182	70/749	0,664446054	0,999954951	0,999954951	BCL6/C3/FCER1G/IL1R1/JAK3/IL4R/NFKBIZ/TGFB1/STAT6/IL6R/SLC11A1/PVR/TNFRSF1B/LILRB1/GATA3/PTPN6	16
GO:2001233	regulation of apoptotic signaling pathway	16/182	70/749	0,664446054	0,999954951	0,999954951	S100A9/HIF1A/SOD2/S100A8/MUC1/LTBR/ICAM1/XBP1/BAX/AGT/TNFSF10/EPO/ATF3/SERPINE1/ADORA2A/PLAUR	16
GO:0071900	regulation of protein serine/threonine kinase activity	16/182	71/749	0,689334125	0,999954951	0,999954951	LTF/FPR1/CD24/TGFB1/CDKN1A/TLR4/KITLG/APOE/MAPK11/S100A12/FGD2/TRIB1/NOX4/C5AR1/GDF15/PTPN6	16
GO:0030855	epithelial cell differentiation	16/182	72/749	0,713162907	0,999954951	0,999954951	HIF1A/PLAAT4/CD24/COL4A1/TNFRSF1A/KRT8/ICAM1/MAF/STAT1/TGFB1/XBP1/CDKN1A/KRT19/ERRFI1/SERPINE1/GAT	16
GO:0048729	tissue morphogenesis	16/182	72/749	0,713162907	0,999954951	0,999954951	HIF1A/COL4A1/PSMB8/SOCS3/COL3A1/PSME2/PSMB10/STAT1/TGFB1/AGT/TNC/FKBP1A/MYC/PSME1/MYOM2/GATA3	16
GO:0050707	regulation of cytokine secretion	16/182	72/749	0,713162907	0,999954951	0,999954951	IL4R/SOCS1/XBP1/IL17RA/TLR4/TLR2/MAPK11/TNFRSF1B/IL1R2/CD58/FN1/LILRB1/CD14/GATA3/IFNGR1/IL17RB	16
GO:0000122	negative regulation of transcription by RNA polymerase II	16/182	74/749	0,757455013	0,999954951	0,999954951	BCL6/STAT3/MAF/RELB/STAT1/TGFB1/DNMT1/STAT6/EGR1/IRF7/RORC/MYC/EPO/ATF3/GATA3/NFKB1	16
GO:0051704	multi-organism process	16/182	76/749	0,797081312	0,999954951	0,999954951	BCL6/TIMP1/STAT3/ADAMTS1/IL4R/TGFB1/BAX/AGT/APOL2/ARHGDIB/ABCC2/BIRC3/IFIT1/EPO/RGN/THBD	16
GO:0051345	positive regulation of hydrolase activity	16/182	82/749	0,888283853	0,999954951	0,999954951	S100A9/STAT3/S100A8/PSME2/ICAM1/BAX/AGT/MYC/ARHGDIB/TNFSF10/ERRFI1/C5AR1/PSME1/FN1/CASP4/RGN	16
GO:0002703	regulation of leukocyte mediated immunity	16/182	83/749	0,899737349	0,999954951	0,999954951	BCL6/C3/FCER1G/IL1R1/ICAM1/JAK3/IL4R/TGFB1/STAT6/TLR4/PVR/TNFRSF1B/ITGB2/LILRB1/GATA3/PTPN6	16
GO:0042098	T cell proliferation	16/182	93/749	0,970081489	0,999954951	0,999954951	CD24/PSMB10/JAK3/TGFB1/BAX/VCAM1/CD276/LILRB2/IL6R/SLC11A1/TNFRSF1B/LILRB1/EPO/TNFSF14/IL6ST/PTPN6	16
GO:0060326	cell chemotaxis	16/182	94/749	0,973809663	0,999954951	0,999954951	S100A9/S100A8/FCER1G/NR4A1/CXCL16/VCAM1/IL17RA/SLAMF8/CXCL1/IL6R/S100A12/CMKLR1/ITGB2/C5AR1/SERPIN	16
GO:0030217	T cell differentiation	16/182	99/749	0,986948816	0,999954951	0,999954951	BCL3/BCL6/STAT3/FCER1G/RELB/JAK3/IL4R/NFKBIZ/TGFB1/SOCS1/XBP1/STAT6/EGR1/LILRB2/RORC/GATA3	16
GO:0009314	response to radiation	15/182	42/749	0,059786618	0,999954951	0,999954951	HIF1A/BCL3/MAPK13/MME/COL3A1/ICAM1/TGFB1/BAX/CDKN1A/VCAM1/EGR1/MYC/NOX4/GATA3/THBD	15
GO:0045861	negative regulation of proteolysis	15/182	43/749	0,072485814	0,999954951	0,999954951	SERPINA3/LTF/RARRES1/TIMP1/C3/SLPI/SERPING1/HDAC6/AGT/IL1R2/BIRC3/SERPINE1/ADORA2A/TNFSF14/PLAUR	15
GO:0001933	negative regulation of protein phosphorylation	15/182	46/749	0,120767445	0,999954951	0,999954951	SOCS3/TGFB1/SOCS1/BAX/CDKN1A/TLR4/APOE/MYC/TRIB1/ERRFI1/ATF3/ADORA2A/RGN/RNF149/PTPN6	15
GO:0031667	response to nutrient levels	15/182	47/749	0,140293976	0,999954951	0,999954951	LCN2/COL1A1/ICAM1/STAT1/TGFB1/XBP1/CDKN1A/VCAM1/LDLR/TNC/APOE/G6PD/GDF15/EPO/ATF3	15

GO:0009991	response to extracellular stimulus	15/182	53/749	0,289221721	0,999954951	0,999954951	LCN2/COL1A1/ICAM1/STAT1/TGFB1/XBP1/CDKN1A/VCAM1/LDLR/TNC/APOE/G6PD/GDF15/EPO/ATF3	15
GO:0001655	urogenital system development	15/182	56/749	0,378098472	0,999954951	0,999954951	CD24/COL4A1/ADAMTS1/MME/STAT1/TGFB1/BAX/AGT/TNC/EGR1/IL6R/MYC/TIPARP/GATA3/RGN	15
GO:0072511	divalent inorganic cation transport	15/182	57/749	0,408704192	0,999954951	0,999954951	EHD3/ICAM1/TGFB1/BAX/AGT/FKBP1A/LILRB2/SLC11A1/ABCC2/G6PD/LILRB1/EPO/ADORA2A/RGN/PTPN6	15
GO:1902532	negative regulation of intracellular signal transduction	15/182	61/749	0,531042635	0,999954951	0,999954951	HIF1A/SOD2/BCL6/MUC1/STAT1/XBP1/TLR4/APOE/MYC/ERRFI1/EPO/ATF3/RNF149/PTPN6/PLAUR	15
GO:0007507	heart development	15/182	62/749	0,560739378	0,999954951	0,999954951	HIF1A/ADAMTS1/TNFRSF1A/COL3A1/TGFB1/CDKN1A/VCAM1/AGT/FKBP1A/MAPK11/TNFRSF1B/NOX4/G6PD/MYOM2/[illegible]	15
GO:0044703	multi-organism reproductive process	15/182	67/749	0,696937559	0,999954951	0,999954951	BCL6/TIMP1/STAT3/ADAMTS1/IL4R/TGFB1/BAX/AGT/APOL2/ARHGDIB/ABCC2/BIRC3/EPO/RGN/THBD	15
GO:0071346	cellular response to interferon-gamma	15/182	70/749	0,765637674	0,999954951	0,999954951	JAK1/IFNGR2/SOCS3/GBP2/ICAM1/FCGR1A/STAT1/SOCS1/VCAM1/IFI30/TLR4/TLR2/IRF7/CD58/IFNGR1	15
GO:0007162	negative regulation of cell adhesion	15/182	73/749	0,823207375	0,999954951	0,999954951	BCL6/MUC1/COL1A1/JAK3/IL4R/TGFB1/SOCS1/TNC/LILRB2/TGFBI/ARHGDIB/LILRB1/SERPINE1/ADORA2A/PTPN6	15
GO:0002793	positive regulation of peptide secretion	15/182	75/749	0,85544048	0,999954951	0,999954951	HIF1A/S100A8/IL4R/TGFB1/XBP1/IL17RA/TLR4/TLR2/MAPK11/CD58/CD14/GATA3/IFNGR1/ADORA2A/IL17RB	15
GO:0030030	cell projection organization	15/182	78/749	0,895164945	0,999954951	0,999954951	S100A9/EHD3/LCN2/HDAC6/ICAM1/AGT/TNC/APOE/LST1/EMP3/FGD2/FN1/EPO/GATA3/ADORA2A	15
GO:0034613	cellular protein localization	15/182	85/749	0,95453779	0,999954951	0,999954951	BCL3/STAT3/CD24/FCER1G/TNFRSF1A/EHD3/GBP2/HDAC6/COL1A1/TGFB1/BAX/CDKN1A/AGT/APOE/ITGB2	15
GO:0070727	cellular macromolecule localization	15/182	85/749	0,95453779	0,999954951	0,999954951	BCL3/STAT3/CD24/FCER1G/TNFRSF1A/EHD3/GBP2/HDAC6/COL1A1/TGFB1/BAX/CDKN1A/AGT/APOE/ITGB2	15
GO:0051222	positive regulation of protein transport	15/182	88/749	0,969305071	0,999954951	0,999954951	HIF1A/IL4R/TGFB1/XBP1/IL17RA/TLR4/TLR2/MAPK11/CD58/ITGB2/CD14/GATA3/IFNGR1/ADORA2A/IL17RB	15
GO:1904951	positive regulation of establishment of protein localization	15/182	88/749	0,969305071	0,999954951	0,999954951	HIF1A/IL4R/TGFB1/XBP1/IL17RA/TLR4/TLR2/MAPK11/CD58/ITGB2/CD14/GATA3/IFNGR1/ADORA2A/IL17RB	15
GO:0043410	positive regulation of MAPK cascade	15/182	95/749	0,988613618	0,999954951	0,999954951	FPR1/CD24/LTBR/ICAM1/TGFB1/TLR4/KITLG/APOE/MAPK11/S100A12/FGD2/NOX4/C5AR1/GDF15/EPO	15
GO:0051092	positive regulation of NF-kappaB transcription factor activity	14/182	44/749	0,154381996	0,999954951	0,999954951	S100A9/LTF/STAT3/S100A8/ICAM1/TGFB1/AGT/NFKB2/TLR4/MYD88/TLR2/S100A12/ITGB2/NFKB1	14
GO:0007596	blood coagulation	14/182	47/749	0,22885722	0,999954951	0,999954951	SERPING1/FCER1G/EHD3/COL1A1/COL3A1/CD59/TLR4/APOE/SERPINE1/GATA3/ADORA2A/THBD/PTPN6/PLAUR	14
GO:0007599	hemostasis	14/182	47/749	0,22885722	0,999954951	0,999954951	SERPING1/FCER1G/EHD3/COL1A1/COL3A1/CD59/TLR4/APOE/SERPINE1/GATA3/ADORA2A/THBD/PTPN6/PLAUR	14
GO:0019216	regulation of lipid metabolic process	14/182	47/749	0,22885722	0,999954951	0,999954951	SERPINA3/C3/SOCS3/TNFRSF1A/TGFB1/SOCS1/AGT/LDLR/EGR1/APOE/RORC/NFKB1/ALAS1/RGN	14
GO:0050817	coagulation	14/182	48/749	0,25659724	0,999954951	0,999954951	SERPING1/FCER1G/EHD3/COL1A1/COL3A1/CD59/TLR4/APOE/SERPINE1/GATA3/ADORA2A/THBD/PTPN6/PLAUR	14
GO:1905114	cell surface receptor signaling pathway involved in cell-cell signa	14/182	51/749	0,346162909	0,999954951	0,999954951	C3/CD24/SOST/PSMB8/COL1A1/PSME2/PSMB10/EGR1/APOE/TLR2/PSME1/GATA3/ADORA2A/NFKB1	14
GO:0002286	T cell activation involved in immune response	14/182	52/749	0,377466127	0,999954951	0,999954951	BCL3/BCL6/STAT3/FCER1G/ICAM1/RELB/JAK3/IL4R/NFKBIZ/STAT6/SLC11A1/RORC/LILRB1/GATA3	14
GO:0019882	antigen processing and presentation	14/182	52/749	0,377466127	0,999954951	0,999954951	FCER1G/PSMB8/PSME2/ICAM1/PSMB10/RELB/FCGR1A/IFI30/CTSS/LILRB2/SLC11A1/TAP2/PSME1/TAP1	14
GO:0007259	receptor signaling pathway via JAK-STAT	14/182	53/749	0,409143606	0,999954951	0,999954951	BCL3/STAT3/SOCS3/TNFRSF1A/IFNAR2/JAK3/STAT1/SOCS1/STAT6/AGT/IL6R/IL10RB/EPO/IL6ST	14
GO:0072001	renal system development	14/182	53/749	0,409143606	0,999954951	0,999954951	CD24/COL4A1/ADAMTS1/MME/STAT1/TGFB1/BAX/AGT/EGR1/IL6R/MYC/TIPARP/GATA3/RGN	14
GO:0019058	viral life cycle	14/182	54/749	0,440984935	0,999954951	0,999954951	LTF/IFITM3/ITGB6/SLPI/IFITM2/IFITM1/HAVCR1/ICAM1/LDLR/APOE/PVR/ISG20/IFIT1/ISG15	14
GO:0034330	cell junction organization	14/182	54/749	0,440984935	0,999954951	0,999954951	C1QB/C3/COL4A1/HDAC6/C1QA/TGFB1/AGT/TNC/APOE/TLR2/LILRB2/PVR/C5AR1/FN1	14
GO:0042692	muscle cell differentiation	14/182	54/749	0,440984935	0,999954951	0,999954951	SOD2/KRT8/IL4R/TGFB1/XBP1/DNMT1/AGT/MAPK11/KRT19/NOX4/G6PD/MYOM2/GDF15/TNFSF14	14
GO:0044255	cellular lipid metabolic process	14/182	54/749	0,440984935	0,999954951	0,999954951	ALDH3A2/PLAAT4/C3/SOCS3/TNFRSF1A/TGFB1/SOCS1/XBP1/BAX/ALOX5/AGT/LDLR/APOE/RGN	14
GO:0072593	reactive oxygen species metabolic process	14/182	54/749	0,440984935	0,999954951	0,999954951	HIF1A/SOD2/STAT3/HDAC6/ICAM1/TGFB1/CDKN1A/AGT/TLR4/TLR2/ITGB2/NOX4/G6PD/RGN	14
GO:0097696	receptor signaling pathway via STAT	14/182	54/749	0,440984935	0,999954951	0,999954951	BCL3/STAT3/SOCS3/TNFRSF1A/IFNAR2/JAK3/STAT1/SOCS1/STAT6/AGT/IL6R/IL10RB/EPO/IL6ST	14
GO:0045862	positive regulation of proteolysis	14/182	55/749	0,472784918	0,999954951	0,999954951	S100A9/STAT3/S100A8/PSME2/BAX/APOE/TNFRSF1B/MYC/TNFSF10/TRIB1/PSME1/FN1/CASP4/RGN	14
GO:0001501	skeletal system development	14/182	56/749	0,504347067	0,999954951	0,999954951	HIF1A/LTF/TIMP1/COL1A1/COL3A1/MAF/TGFB1/VCAN/TGFBI/CMKLR1/TIPARP/LILRB1/RGN/PTPN6	14
GO:0070838	divalent metal ion transport	14/182	56/749	0,504347067	0,999954951	0,999954951	EHD3/ICAM1/TGFB1/BAX/AGT/FKBP1A/LILRB2/SLC11A1/G6PD/LILRB1/EPO/ADORA2A/RGN/PTPN6	14
GO:0050877	nervous system process	14/182	57/749	0,535486607	0,999954951	0,999954951	HIF1A/SOD2/MME/COL1A1/ICAM1/AGT/LDLR/APOE/TLR2/LILRB2/TNFRSF1B/TGFBI/C5AR1/ADORA2A	14
GO:0051091	positive regulation of DNA-binding transcription factor activity	14/182	57/749	0,535486607	0,999954951	0,999954951	S100A9/LTF/STAT3/S100A8/ICAM1/TGFB1/AGT/NFKB2/TLR4/MYD88/TLR2/S100A12/ITGB2/NFKB1	14
GO:0016064	immunoglobulin mediated immune response	14/182	58/749	0,566032975	0,999954951	0,999954951	BCL3/C1QB/C1S/BCL6/C3/SERPING1/C9/FCER1G/C1QA/IL4R/TGFB1/STAT6/IRF7/PTPN6	14
GO:0048872	homeostasis of number of cells	14/182	58/749	0,566032975	0,999954951	0,999954951	HIF1A/BCL6/STAT3/FCER1G/JAK3/STAT1/TGFB1/BAX/KITLG/G6PD/EPO/GATA3/TNFSF14/ISG15	14
GO:0035690	cellular response to drug	14/182	59/749	0,59583177	0,999954951	0,999954951	MAPK13/LCN2/HDAC6/ICAM1/TGFB1/AHR/STAT6/EGR1/SLAMF8/MYC/ABCC2/ERRFI1/NFKB1/RNF149	14
GO:0019724	B cell mediated immunity	14/182	60/749	0,624746176	0,999954951	0,999954951	BCL3/C1QB/C1S/BCL6/C3/SERPING1/C9/FCER1G/C1QA/IL4R/TGFB1/STAT6/IRF7/PTPN6	14
GO:0097191	extrinsic apoptotic signaling pathway	14/182	60/749	0,624746176	0,999954951	0,999954951	TNFRSF1A/LCN2/KRT8/LTBR/ICAM1/TGFB1/BAX/AGT/KITLG/IL6R/TNFRSF1B/TNFSF10/ATF3/SERPINE1	14
GO:0043405	regulation of MAP kinase activity	14/182	61/749	0,65265787	0,999954951	0,999954951	FPR1/CD24/TGFB1/TLR4/KITLG/APOE/MAPK11/S100A12/FGD2/TRIB1/NOX4/C5AR1/GDF15/PTPN6	14
GO:0001666	response to hypoxia	14/182	63/749	0,70509432	0,999954951	0,999954951	HIF1A/CD24/PSMB8/PSME2/ICAM1/PSMB10/TGFB1/VCAM1/EGR1/TLR2/MYC/NOX4/PSME1/EPO	14
GO:0002573	myeloid leukocyte differentiation	14/182	63/749	0,70509432	0,999954951	0,999954951	LTF/FCER1G/LTBR/RELB/TGFB1/TLR4/KITLG/TLR2/IRF7/MYC/TRIB1/LILRB1/TMEM178A/GATA3	14
GO:0036293	response to decreased oxygen levels	14/182	63/749	0,70509432	0,999954951	0,999954951	HIF1A/CD24/PSMB8/PSME2/ICAM1/PSMB10/TGFB1/VCAM1/EGR1/TLR2/MYC/NOX4/PSME1/EPO	14
GO:0050673	epithelial cell proliferation	14/182	63/749	0,70509432	0,999954951	0,999954951	HIF1A/STAT3/NR4A1/STAT1/TGFB1/XBP1/BAX/STAT6/APOE/MYC/ERRFI1/C5AR1/GATA3/RGN	14
GO:0006979	response to oxidative stress	14/182	65/749	0,752569557	0,999954951	0,999954951	HIF1A/SOD2/MAPK13/LCN2/HDAC6/COL1A1/STAT1/STAT6/TLR4/APOE/ABCC2/NOX4/G6PD/RGN	14
GO:0007010	cytoskeleton organization	14/182	66/749	0,774345927	0,999954951	0,999954951	S100A9/BCL6/S100A8/KRT8/HDAC6/ICAM1/TGFB1/APOE/CXCL1/ARHGDIB/FGD2/KRT19/NOX4/MYOM2	14
GO:0022402	cell cycle process	14/182	67/749	0,79479342	0,999954951	0,999954951	BCL6/ADAMTS1/PSMB8/MUC1/ABCB1/PSME2/PSMB10/TGFB1/BAX/CDKN1A/MYC/PSME1/KLHL13/PTPN6	14
GO:0046631	alpha-beta T cell activation	14/182	69/749	0,831721996	0,999954951	0,999954951	BCL3/BCL6/STAT3/RELB/JAK3/IL4R/NFKBIZ/SOCS1/STAT6/IL6R/RORC/LILRB1/GATA3/ADORA2A	14
GO:1903708	positive regulation of hemopoiesis	14/182	72/749	0,877572098	0,999954951	0,999954951	HIF1A/BCL6/STAT3/STAT1/IL4R/NFKBIZ/TGFB1/SOCS1/XBP1/KITLG/LILRB2/TRIB1/GATA3/ISG15	14
GO:0050714	positive regulation of protein secretion	14/182	73/749	0,890472201	0,999954951	0,999954951	HIF1A/IL4R/TGFB1/XBP1/IL17RA/TLR4/TLR2/MAPK11/CD58/CD14/GATA3/IFNGR1/ADORA2A/IL17RB	14
GO:0120036	plasma membrane bounded cell projection organization	14/182	74/749	0,902266764	0,999954951	0,999954951	S100A9/EHD3/HDAC6/ICAM1/AGT/TNC/APOE/LST1/EMP3/FGD2/FN1/EPO/GATA3/ADORA2A	14
GO:1901342	regulation of vasculature development	14/182	74/749	0,902266764	0,999954951	0,999954951	HIF1A/JAK1/SOD2/STAT3/C3/STAT1/XBP1/DNMT1/AGT/EGR1/IL6R/ITGB2/C5AR1/SERPINE1	14
GO:0002440	production of molecular mediator of immune response	14/182	77/749	0,931608556	0,999954951	0,999954951	BCL6/FCER1G/IL1R1/JAK3/IL4R/TGFB1/XBP1/STAT6/TLR4/TLR2/SLC11A1/TNFRSF1B/LILRB1/GATA3	14
GO:0030595	leukocyte chemotaxis	14/182	79/749	0,946751867	0,999954951	0,999954951	S100A9/S100A8/FCER1G/CXCL16/IL17RA/SLAMF8/CXCL1/IL6R/S100A12/CMKLR1/ITGB2/C5AR1/SERPINE1/TNFSF14	14
GO:0071363	cellular response to growth factor stimulus	14/182	88/749	0,984591759	0,999954951	0,999954951	HIF1A/SOST/COL1A1/NR4A1/COL3A1/TGFB1/VCAM1/AGT/EGR1/TNFRSF1B/ERRFI1/NOX4/GDF15/GATA3	14
GO:0001525	angiogenesis	14/182	95/749	0,994782796	0,999954951	0,999954951	HIF1A/JAK1/STAT3/C3/COL4A1/NR4A1/STAT1/XBP1/AGT/TGFBI/ITGB2/C5AR1/FN1/SERPINE1	14
GO:0044257	cellular protein catabolic process	13/182	34/749	0,045875397	0,999954951	0,999954951	PSMB8/HDAC6/PSME2/PSMB10/XBP1/LDLR/CTSS/APOE/TRIB1/PSME1/ISG15/RGN/RNF149	13

GO:0010466	negative regulation of peptidase activity	13/182	36/749	0,071425161	0,999954951	0,999954951	SERPINA3/LTF/RARRES1/TIMP1/C3/SLPI/SERPING1/AGT/BIRC3/SERPINE1/ADORA2A/TNFSF14/PLAUR	13
GO:0010951	negative regulation of endopeptidase activity	13/182	36/749	0,071425161	0,999954951	0,999954951	SERPINA3/LTF/RARRES1/TIMP1/C3/SLPI/SERPING1/AGT/BIRC3/SERPINE1/ADORA2A/TNFSF14/PLAUR	13
GO:0006898	receptor-mediated endocytosis	13/182	40/749	0,146381538	0,999954951	0,999954951	CD163/C3/FCER1G/FCGR1A/CXCL16/APOL1/LDLR/MASP1/APOE/ITGB2/LILRB1/CD14/SERPINE1	13
GO:0002224	toll-like receptor signaling pathway	13/182	42/749	0,195513648	0,999954951	0,999954951	S100A9/LTF/S100A8/LY96/TLR4/CTSS/MYD88/TLR2/IRF7/TBK1/ITGB2/BIRC3/CD14	13
GO:0062012	regulation of small molecule metabolic process	13/182	42/749	0,195513648	0,999954951	0,999954951	HIF1A/STAT3/PSMB8/PSME2/PSMB10/TGFB1/LDLR/EGR1/APOE/RORC/PSME1/NFKB1/RGN	13
GO:1903047	mitotic cell cycle process	13/182	42/749	0,195513648	0,999954951	0,999954951	BCL6/ADAMTS1/PSMB8/MUC1/ABCB1/PSME2/PSMB10/TGFB1/BAX/CDKN1A/MYC/PSME1/PTPN6	13
GO:0033209	tumor necrosis factor-mediated signaling pathway	13/182	44/749	0,251274257	0,999954951	0,999954951	PSMB8/TNFRSF1A/KRT8/LTBR/PSME2/PSMB10/STAT1/TNFRSF1B/BIRC3/PSME1/CASP4/TNFSF14/LTB	13
GO:0040013	negative regulation of locomotion	13/182	44/749	0,251274257	0,999954951	0,999954951	TIMP1/STAT3/IFITM1/COL3A1/TGFB1/SLAMF8/APOE/ARHGDIB/TRIB1/SERPINE1/GATA3/ADORA2A/RGN	13
GO:0002221	pattern recognition receptor signaling pathway	13/182	45/749	0,281200909	0,999954951	0,999954951	S100A9/LTF/S100A8/LY96/TLR4/CTSS/MYD88/TLR2/IRF7/TBK1/ITGB2/BIRC3/CD14	13
GO:0051924	regulation of calcium ion transport	13/182	45/749	0,281200909	0,999954951	0,999954951	EHD3/ICAM1/TGFB1/BAX/AGT/FKBP1A/LILRB2/G6PD/LILRB1/EPO/ADORA2A/RGN/PTPN6	13
GO:0010959	regulation of metal ion transport	13/182	48/749	0,376580449	0,999954951	0,999954951	EHD3/ICAM1/TGFB1/BAX/AGT/FKBP1A/LILRB2/G6PD/LILRB1/EPO/ADORA2A/RGN/PTPN6	13
GO:0017144	drug metabolic process	13/182	48/749	0,376580449	0,999954951	0,999954951	ALDH3A2/STAT3/HDAC6/ICAM1/TGFB1/AGT/EGR1/TLR4/VCAN/TLR2/ABCC2/ITGB2/RGN	13
GO:0000278	mitotic cell cycle	13/182	50/749	0,44249503	0,999954951	0,999954951	BCL6/ADAMTS1/PSMB8/MUC1/ABCB1/PSME2/PSMB10/TGFB1/BAX/CDKN1A/MYC/PSME1/PTPN6	13
GO:0001101	response to acid chemical	13/182	51/749	0,475450756	0,999954951	0,999954951	COL4A1/KRT8/COL1A1/COL3A1/ICAM1/SOCS1/XBP1/DNMT1/LDLR/TNC/EGR1/TLR2/ABCC2	13
GO:0001822	kidney development	13/182	52/749	0,508112299	0,999954951	0,999954951	CD24/ADAMTS1/MME/STAT1/TGFB1/BAX/AGT/EGR1/IL6R/MYC/TIPARP/GATA3/RGN	13
GO:0009612	response to mechanical stimulus	13/182	53/749	0,540277124	0,999954951	0,999954951	SOST/TNFRSF1A/LTBR/COL1A1/COL3A1/STAT1/TGFB1/AGT/TNC/TLR4/MYD88/TNFSF14/NFKB1	13
GO:0045637	regulation of myeloid cell differentiation	13/182	54/749	0,571760013	0,999954951	0,999954951	HIF1A/LTF/STAT3/STAT1/TGFB1/TLR4/KITLG/IRF7/MYC/TRIB1/LILRB1/TMEM178A/ISG15	13
GO:0045786	negative regulation of cell cycle	13/182	54/749	0,571760013	0,999954951	0,999954951	BCL6/PSMB8/MUC1/NR4A1/PSME2/PSMB10/TGFB1/BAX/CDKN1A/MYC/PSME1/LILRB1/GATA3	13
GO:0062197	cellular response to chemical stress	13/182	54/749	0,571760013	0,999954951	0,999954951	HIF1A/SOD2/MAPK13/LCN2/HDAC6/RELB/STAT6/TLR4/ERRFI1/NOX4/G6PD/EPO/RGN	13
GO:0006816	calcium ion transport	13/182	55/749	0,602395162	0,999954951	0,999954951	EHD3/ICAM1/TGFB1/BAX/AGT/FKBP1A/LILRB2/G6PD/LILRB1/EPO/ADORA2A/RGN/PTPN6	13
GO:0006909	phagocytosis	13/182	55/749	0,602395162	0,999954951	0,999954951	C3/FCER1G/FCGR1A/TGFB1/FCGR2A/LDLR/TLR4/MYD88/TLR2/SLC11A1/ITGB2/CD14/ADORA2A	13
GO:0042089	cytokine biosynthetic process	13/182	55/749	0,602395162	0,999954951	0,999954951	BCL3/STAT3/SIGIRR/CD276/EGR1/TLR4/IRF7/TBK1/ERRFI1/LILRB1/GATA3/NFKB1/LTB	13
GO:0042107	cytokine metabolic process	13/182	55/749	0,602395162	0,999954951	0,999954951	BCL3/STAT3/SIGIRR/CD276/EGR1/TLR4/IRF7/TBK1/ERRFI1/LILRB1/GATA3/NFKB1/LTB	13
GO:0010720	positive regulation of cell development	13/182	56/749	0,632037609	0,999954951	0,999954951	S100A9/HIF1A/BCL6/MME/TGFB1/AGT/APOE/TLR2/TNFRSF1B/TRIB1/FN1/EPO/IL6ST	13
GO:0050678	regulation of epithelial cell proliferation	13/182	56/749	0,632037609	0,999954951	0,999954951	HIF1A/STAT3/NR4A1/STAT1/TGFB1/XBP1/BAX/APOE/MYC/ERRFI1/C5AR1/GATA3/RGN	13
GO:0050731	positive regulation of peptidyl-tyrosine phosphorylation	13/182	59/749	0,713884673	0,999954951	0,999954951	STAT3/CD24/SOCS3/TNFRSF1A/ICAM1/FCGR1A/TGFB1/AGT/KITLG/IL6R/NOX4/EPO/IL6ST	13
GO:0002009	morphogenesis of an epithelium	13/182	60/749	0,738540002	0,999954951	0,999954951	HIF1A/COL4A1/PSMB8/SOCS3/PSME2/PSMB10/STAT1/TGFB1/AGT/TNC/MYC/PSME1/GATA3	13
GO:0048608	reproductive structure development	13/182	60/749	0,738540002	0,999954951	0,999954951	HIF1A/ADAMTS1/SOCS3/MME/KRT8/ICAM1/BAX/TNC/KITLG/KRT19/TNFSF10/TIPARP/GATA3	13
GO:0051607	defense response to virus	13/182	60/749	0,738540002	0,999954951	0,999954951	IFITM3/IFITM2/IFITM1/IFNAR2/STAT1/IRF7/TBK1/ISG20/BIRC3/LILRB1/IL10RB/IFIT1/ISG15	13
GO:0061458	reproductive system development	13/182	60/749	0,738540002	0,999954951	0,999954951	HIF1A/ADAMTS1/SOCS3/MME/KRT8/ICAM1/BAX/TNC/KITLG/KRT19/TNFSF10/TIPARP/GATA3	13
GO:0050866	negative regulation of cell activation	13/182	63/749	0,804065675	0,999954951	0,999954951	BCL6/JAK3/IL4R/TGFB1/SOCS1/LDLR/APOE/LILRB2/LST1/LILRB1/ADORA2A/THBD/PTPN6	13
GO:0061024	membrane organization	13/182	64/749	0,823078608	0,999954951	0,999954951	STAT3/C3/CD24/FCER1G/ABCB1/FCGR1A/BAX/CD59/LDLR/APOE/TAP2/ITGB2/TAP1	13
GO:0006874	cellular calcium ion homeostasis	13/182	65/749	0,840705871	0,999954951	0,999954951	FPR1/CD24/TGFB1/BAX/AGT/FKBP1A/APOE/CMKLR1/C5AR1/TMEM178A/EPO/RGN/PTPN6	13
GO:0055074	calcium ion homeostasis	13/182	65/749	0,840705871	0,999954951	0,999954951	FPR1/CD24/TGFB1/BAX/AGT/FKBP1A/APOE/CMKLR1/C5AR1/TMEM178A/EPO/RGN/PTPN6	13
GO:0006897	endocytosis	13/182	67/749	0,871954365	0,999954951	0,999954951	CD163/C3/FCER1G/FCGR1A/CXCL16/APOL1/LDLR/MASP1/APOE/ITGB2/LILRB1/CD14/SERPINE1	13
GO:0048666	neuron development	13/182	68/749	0,885673009	0,999954951	0,999954951	S100A9/C3/HDAC6/C1QA/XBP1/AGT/TNC/APOE/LST1/FN1/EPO/GATA3/ADORA2A	13
GO:0070371	ERK1 and ERK2 cascade	13/182	71/749	0,919918321	0,999954951	0,999954951	ICAM1/TGFB1/AGT/TLR4/APOE/MYC/ERRFI1/NOX4/C5AR1/FN1/EPO/ATF3/PTPN6	13
GO:0080135	regulation of cellular response to stress	13/182	73/749	0,93765045	0,999954951	0,999954951	HIF1A/SOD2/BCL6/MUC1/LTBR/HDAC6/XBP1/BAX/TLR4/MYC/FGD2/EPO/RGN	13
GO:0000902	cell morphogenesis	13/182	74/749	0,945191236	0,999954951	0,999954951	BCL6/HDAC6/ICAM1/PSMB10/APOE/LST1/FGD2/ITGB2/NOX4/FN1/GATA3/ADORA2A/PTPN6	13
GO:0097529	myeloid leukocyte migration	13/182	76/749	0,957956169	0,999954951	0,999954951	S100A9/S100A8/FCER1G/IL1R1/IL17RA/SLAMF8/CXCL1/IL6R/S100A12/CMKLR1/ITGB2/C5AR1/SERPINE1	13
GO:0002699	positive regulation of immune effector process	13/182	85/749	0,98861883	0,999954951	0,999954951	C3/FCER1G/IL1R1/IL4R/NFKBIZ/TGFB1/XBP1/STAT6/TLR4/PVR/ITGB2/LILRB1/GATA3	13
GO:0042113	B cell activation	13/182	85/749	0,98861883	0,999954951	0,999954951	BCL3/BCL6/JAK3/TGFB1/XBP1/BAX/AHR/CDKN1A/VCAM1/STAT6/TLR4/SLAMF8/PTPN6	13
GO:0010001	glial cell differentiation	12/182	30/749	0,038354947	0,999954951	0,999954951	STAT3/S100A8/C1QA/TGFB1/LDLR/TLR4/TLR2/TNFRSF1B/C5AR1/IFNGR1/ADORA2A/IL6ST	12
GO:0051603	proteolysis involved in cellular protein catabolic process	12/182	30/749	0,038354947	0,999954951	0,999954951	PSMB8/HDAC6/PSME2/PSMB10/XBP1/CTSS/APOE/TRIB1/PSME1/ISG15/RGN/RNF149	12
GO:0044772	mitotic cell cycle phase transition	12/182	31/749	0,049508756	0,999954951	0,999954951	ADAMTS1/PSMB8/MUC1/ABCB1/PSME2/PSMB10/TGFB1/BAX/CDKN1A/MYC/PSME1/PTPN6	12
GO:0044770	cell cycle phase transition	12/182	32/749	0,062703805	0,999954951	0,999954951	ADAMTS1/PSMB8/MUC1/ABCB1/PSME2/PSMB10/TGFB1/BAX/CDKN1A/MYC/PSME1/PTPN6	12
GO:0008610	lipid biosynthetic process	12/182	33/749	0,078043258	0,999954951	0,999954951	ALDH3A2/C3/TNFRSF1A/XBP1/ALOX5/HSD11B1/LDLR/EGR1/APOE/G6PD/NFKB1/RGN	12
GO:0055114	oxidation-reduction process	12/182	33/749	0,078043258	0,999954951	0,999954951	ALDH3A2/HIF1A/SOD2/IMPDH1/ALOX5/VCAM1/HSD11B1/IFI30/MYC/NOX4/G6PD/IL6ST	12
GO:0002262	myeloid cell homeostasis	12/182	38/749	0,187515986	0,999954951	0,999954951	HIF1A/BCL6/STAT3/FCER1G/JAK3/STAT1/BAX/KITLG/G6PD/EPO/GATA3/ISG15	12
GO:0038061	NIK/NF-kappaB signaling	12/182	39/749	0,215463793	0,999954951	0,999954951	BCL3/PSMB8/PSME2/PSMB10/RELB/NFKB2/TLR4/TLR2/BIRC3/PSME1/CD14/TNFSF14	12
GO:0010564	regulation of cell cycle process	12/182	40/749	0,245070474	0,999954951	0,999954951	BCL6/ADAMTS1/PSMB8/MUC1/PSME2/PSMB10/TGFB1/BAX/CDKN1A/PSME1/KLHL13/PTPN6	12
GO:2000146	negative regulation of cell motility	12/182	40/749	0,245070474	0,999954951	0,999954951	TIMP1/STAT3/IFITM1/COL3A1/TGFB1/SLAMF8/APOE/ARHGDIB/TRIB1/SERPINE1/GATA3/RGN	12
GO:0048880	sensory system development	12/182	41/749	0,276115658	0,999954951	0,999954951	HIF1A/STAT3/C3/COL4A1/MEGF11/C1QA/MAF/TGFB1/BAX/FJX1/MYOM2/GATA3	12
GO:0150063	visual system development	12/182	41/749	0,276115658	0,999954951	0,999954951	HIF1A/STAT3/C3/COL4A1/MEGF11/C1QA/MAF/TGFB1/BAX/FJX1/MYOM2/GATA3	12
GO:0051271	negative regulation of cellular component movement	12/182	42/749	0,308356463	0,999954951	0,999954951	TIMP1/STAT3/IFITM1/COL3A1/TGFB1/SLAMF8/APOE/ARHGDIB/TRIB1/SERPINE1/GATA3/RGN	12
GO:0016049	cell growth	12/182	44/749	0,375381843	0,999954951	0,999954951	S100A9/BCL6/S100A8/HDAC6/TGFB1/CXCL16/CDKN1A/AGT/APOE/G6PD/FN1/IL17RB	12
GO:0034762	regulation of transmembrane transport	12/182	44/749	0,375381843	0,999954951	0,999954951	C3/EHD3/ABCB1/TGFB1/BAX/AGT/FKBP1A/CTSS/G6PD/EPO/RGN/PTPN6	12
GO:0060333	interferon-gamma-mediated signaling pathway	12/182	44/749	0,375381843	0,999954951	0,999954951	JAK1/IFNGR2/SOCS3/GBP2/ICAM1/FCGR1A/STAT1/SOCS1/VCAM1/IFI30/IRF7/IFNGR1	12
GO:0016055	Wnt signaling pathway	12/182	45/749	0,409629456	0,999954951	0,999954951	CD24/SOST/PSMB8/COL1A1/PSME2/PSMB10/EGR1/APOE/TLR2/PSME1/GATA3/NFKB1	12

GO:0198738	cell-cell signaling by wnt	12/182	45/749	0,409629456	0,999954951	0,999954951	CD24/SOST/PSMB8/COL1A1/PSME2/PSMB10/EGR1/APOE/TLR2/PSME1/GATA3/NFKB1	12
GO:0098655	cation transmembrane transport	12/182	46/749	0,444011564	0,999954951	0,999954951	EHD3/TGFB1/BAX/AGT/SLC12A3/FKBP1A/CTSS/SLC11A1/G6PD/EPO/RGN/PTPN6	12
GO:0031348	negative regulation of defense response	12/182	47/749	0,478272101	0,999954951	0,999954951	SERPING1/SOCS3/TNFRSF1A/LDLR/SLAMF8/APOE/TNFRSF1B/IL1R2/LILRB1/GATA3/ADORA2A/NFKB1	12
GO:0042063	gliogenesis	12/182	47/749	0,478272101	0,999954951	0,999954951	STAT3/S100A8/C1QA/TGFB1/LDLR/TLR4/TLR2/TNFRSF1B/C5AR1/IFNGR1/ADORA2A/IL6ST	12
GO:2000377	regulation of reactive oxygen species metabolic process	12/182	47/749	0,478272101	0,999954951	0,999954951	HIF1A/STAT3/HDAC6/ICAM1/TGFB1/CDKN1A/AGT/TLR4/ITGB2/NOX4/G6PD/RGN	12
GO:0042035	regulation of cytokine biosynthetic process	12/182	51/749	0,609555209	0,999954951	0,999954951	BCL3/STAT3/SIGIRR/CD276/EGR1/TLR4/TBK1/ERRFI1/LILRB1/GATA3/NFKB1/LTB	12
GO:0050769	positive regulation of neurogenesis	12/182	51/749	0,609555209	0,999954951	0,999954951	S100A9/HIF1A/BCL6/MME/TGFB1/AGT/APOE/TLR2/TNFRSF1B/FN1/EPO/IL6ST	12
GO:0003013	circulatory system process	12/182	52/749	0,639988082	0,999954951	0,999954951	SOD2/SERPING1/EHD3/MME/ICAM1/STAT1/TGFB1/AGT/APOE/NOX4/EPO/ADORA2A	12
GO:0051962	positive regulation of nervous system development	12/182	54/749	0,697003709	0,999954951	0,999954951	S100A9/HIF1A/BCL6/MME/TGFB1/AGT/APOE/TLR2/TNFRSF1B/FN1/EPO/IL6ST	12
GO:0071902	positive regulation of protein serine/threonine kinase activity	12/182	55/749	0,723406094	0,999954951	0,999954951	LTF/FPR1/CD24/TGFB1/TLR4/KITLG/MAPK11/S100A12/FGD2/NOX4/C5AR1/GDF15	12
GO:0010638	positive regulation of organelle organization	12/182	56/749	0,748324772	0,999954951	0,999954951	HIF1A/BCL6/MUC1/HDAC6/ICAM1/TGFB1/DNMT1/BAX/TNFSF10/NOX4/GATA3/PLAUR	12
GO:0060627	regulation of vesicle-mediated transport	12/182	56/749	0,748324772	0,999954951	0,999954951	C3/FCER1G/IL4R/TGFB1/APOE/TLR2/SLC11A1/ITGB2/LILRB1/CD14/SERPINE1/ADORA2A	12
GO:0001667	ameboidal-type cell migration	12/182	58/749	0,793602751	0,999954951	0,999954951	HIF1A/TIMP1/HDAC6/NR4A1/TGFB1/AGT/KITLG/APOE/ARHGDIB/ITGB2/FN1/GATA3	12
GO:0002833	positive regulation of response to biotic stimulus	12/182	59/749	0,813956303	0,999954951	0,999954951	FCER1G/PSMB8/MUC1/PSME2/PSMB10/RELB/LY96/IRF7/PVR/TBK1/PSME1/NFKB1	12
GO:0006259	DNA metabolic process	12/182	60/749	0,832810378	0,999954951	0,999954951	BCL6/TGFB1/DNMT1/BAX/CDKN1A/STAT6/MYC/ISG20/NOX4/GATA3/ISG15/RGN	12
GO:0051051	negative regulation of transport	12/182	60/749	0,832810378	0,999954951	0,999954951	TNFRSF1A/ICAM1/TGFB1/APOE/TLR2/LILRB2/TNFRSF1B/IL1R2/FN1/LILRB1/EPO/NFKB1	12
GO:0002700	regulation of production of molecular mediator of immune resp	12/182	61/749	0,850200736	0,999954951	0,999954951	BCL6/FCER1G/IL1R1/JAK3/IL4R/TGFB1/XBP1/STAT6/TLR4/TNFRSF1B/LILRB1/GATA3	12
GO:0048589	developmental growth	12/182	62/749	0,86617459	0,999954951	0,999954951	STAT3/HDAC6/TGFB1/CDKN1A/AGT/TNC/APOE/MAPK11/G6PD/FN1/GDF15/GATA3	12
GO:0048871	multicellular organismal homeostasis	12/182	66/749	0,917133148	0,999954951	0,999954951	SERPINA3/LTF/STAT3/LCN2/IL4R/BAX/STAT6/EGR1/TLR4/SLC11A1/CMKLR1/NOX4	12
GO:0044087	regulation of cellular component biogenesis	12/182	69/749	0,943769671	0,999954951	0,999954951	SOST/TNFRSF1A/HDAC6/ICAM1/TGFB1/DNMT1/BAX/AGT/TLR4/APOE/TLR2/NOX4	12
GO:0007186	G protein-coupled receptor signaling pathway	12/182	76/749	0,979143234	0,999954951	0,999954951	FPR1/C3/CXCL16/AGT/APOE/CXCL1/ACKR1/CMKLR1/FGD2/C5AR1/ADORA2A/PTPN6	12
GO:0070665	positive regulation of leukocyte proliferation	12/182	79/749	0,986829165	0,999954951	0,999954951	BCL6/CD24/JAK3/CDKN1A/VCAM1/CD276/TLR4/KITLG/LILRB2/IL6R/EPO/IL6ST	12
GO:0009790	embryo development	12/182	81/749	0,990409744	0,999954951	0,999954951	HIF1A/SOCS3/KRT8/COL1A1/TGFB1/DNMT1/BAX/KITLG/KRT19/ITGB2/FN1/GATA3	12
GO:0042129	regulation of T cell proliferation	12/182	84/749	0,994133215	0,999954951	0,999954951	CD24/JAK3/TGFB1/VCAM1/CD276/LILRB2/IL6R/TNFRSF1B/LILRB1/EPO/IL6ST/PTPN6	12
GO:0009410	response to xenobiotic stimulus	11/182	28/749	0,053281593	0,999954951	0,999954951	ICAM1/TGFB1/AHR/EGR1/RORC/S100A12/ABCC2/ERRFI1/TIPARP/EPO/ADORA2A	11
GO:1901361	organic cyclic compound catabolic process	11/182	28/749	0,053281593	0,999954951	0,999954951	PSMB8/PSME2/PSMB10/BAX/MYD88/APOE/SLC11A1/TNFRSF1B/ISG20/PSME1/RGN	11
GO:0034765	regulation of ion transmembrane transport	11/182	35/749	0,207083841	0,999954951	0,999954951	EHD3/TGFB1/BAX/AGT/FKBP1A/CTSS/G6PD/EPO/RGN/PTPN6	11
GO:0044283	small molecule biosynthetic process	11/182	35/749	0,207083841	0,999954951	0,999954951	IMPDH1/TGFB1/XBP1/ALOX5/EGR1/VCAN/APOE/G6PD/ATF3/NFKB1/RGN	11
GO:2000027	regulation of animal organ morphogenesis	11/182	36/749	0,237776617	0,999954951	0,999954951	PSMB8/PSME2/PSMB10/STAT1/TGFB1/XBP1/BAX/AGT/MYC/PSME1/GATA3	11
GO:0097305	response to alcohol	11/182	37/749	0,270082014	0,999954951	0,999954951	STAT3/S100A8/ICAM1/TGFB1/AHR/CDKN1A/VCAM1/TNC/G6PD/CD14/GATA3	11
GO:0001558	regulation of cell growth	11/182	39/749	0,338392117	0,999954951	0,999954951	S100A9/BCL6/S100A8/TGFB1/CXCL16/CDKN1A/AGT/APOE/G6PD/FN1/IL17RB	11
GO:0002292	T cell differentiation involved in immune response	11/182	39/749	0,338392117	0,999954951	0,999954951	BCL3/BCL6/STAT3/FCER1G/RELB/JAK3/IL4R/NFKBIZ/STAT6/RORC/GATA3	11
GO:0043367	CD4-positive, alpha-beta T cell differentiation	11/182	39/749	0,338392117	0,999954951	0,999954951	BCL3/BCL6/STAT3/RELB/JAK3/IL4R/NFKBIZ/SOCS1/STAT6/RORC/GATA3	11
GO:0051235	maintenance of location	11/182	39/749	0,338392117	0,999954951	0,999954951	S100A9/BCL3/S100A8/C3/LCN2/TGFB1/BAX/FKBP1A/APOE/NFKB1/PTPN6	11
GO:0007346	regulation of mitotic cell cycle	11/182	40/749	0,373789982	0,999954951	0,999954951	BCL6/ADAMTS1/PSMB8/MUC1/PSME2/PSMB10/TGFB1/BAX/CDKN1A/PSME1/PTPN6	11
GO:0048002	antigen processing and presentation of peptide antigen	11/182	40/749	0,373789982	0,999954951	0,999954951	FCER1G/PSMB8/PSME2/PSMB10/FCGR1A/IFI30/CTSS/SLC11A1/TAP2/PSME1/TAP1	11
GO:0007610	behavior	11/182	42/749	0,44553041	0,999954951	0,999954951	HIF1A/STAT3/MME/AGT/LDLR/EGR1/APOE/TLR2/LILRB2/GDF15/ADORA2A	11
GO:0046425	regulation of receptor signaling pathway via JAK-STAT	11/182	44/749	0,516578141	0,999954951	0,999954951	BCL3/STAT3/SOCS3/TNFRSF1A/JAK3/SOCS1/AGT/IL6R/IL10RB/EPO/IL6ST	11
GO:0051146	striated muscle cell differentiation	11/182	44/749	0,516578141	0,999954951	0,999954951	KRT8/IL4R/TGFB1/XBP1/AGT/KRT19/NOX4/G6PD/MYOM2/GDF15/TNFSF14	11
GO:0097435	supramolecular fiber organization	11/182	45/749	0,551179186	0,999954951	0,999954951	KRT8/HDAC6/COL1A1/COL3A1/ICAM1/LDLR/FKBP1A/APOE/KRT19/NOX4/MYOM2	11
GO:1904892	regulation of receptor signaling pathway via STAT	11/182	45/749	0,551179186	0,999954951	0,999954951	BCL3/STAT3/SOCS3/TNFRSF1A/JAK3/SOCS1/AGT/IL6R/IL10RB/EPO/IL6ST	11
GO:0007423	sensory organ development	11/182	47/749	0,617429097	0,999954951	0,999954951	HIF1A/C1QB/STAT3/COL4A1/MEGF11/MAF/TGFB1/BAX/FJX1/MYOM2/GATA3	11
GO:1901135	carbohydrate derivative metabolic process	11/182	47/749	0,617429097	0,999954951	0,999954951	HIF1A/STAT3/LYVE1/MUC1/IMPDH1/JAK3/TGFB1/BAX/VCAN/G6PD/NFKB1	11
GO:0001503	ossification	11/182	48/749	0,648722545	0,999954951	0,999954951	HIF1A/LTF/IFITM1/SOST/COL1A1/TGFB1/TNC/VCAN/IL6R/IL6ST/ISG15	11
GO:0008015	blood circulation	11/182	48/749	0,648722545	0,999954951	0,999954951	SOD2/SERPING1/EHD3/MME/ICAM1/STAT1/TGFB1/AGT/APOE/EPO/ADORA2A	11
GO:0046632	alpha-beta T cell differentiation	11/182	48/749	0,648722545	0,999954951	0,999954951	BCL3/BCL6/STAT3/RELB/JAK3/IL4R/NFKBIZ/SOCS1/STAT6/RORC/GATA3	11
GO:1904018	positive regulation of vasculature development	11/182	48/749	0,648722545	0,999954951	0,999954951	HIF1A/JAK1/SOD2/STAT3/C3/XBP1/EGR1/IL6R/ITGB2/C5AR1/SERPINE1	11
GO:0002367	cytokine production involved in immune response	11/182	49/749	0,678608622	0,999954951	0,999954951	BCL6/FCER1G/IL1R1/JAK3/TGFB1/TLR4/TLR2/SLC11A1/TNFRSF1B/LILRB1/GATA3	11
GO:0032640	tumor necrosis factor production	11/182	49/749	0,678608622	0,999954951	0,999954951	LTF/BCL3/FCER1G/LY96/TLR4/TLR2/ERRFI1/LILRB1/CD14/IFNGR1/PTPN6	11
GO:0032680	regulation of tumor necrosis factor production	11/182	49/749	0,678608622	0,999954951	0,999954951	LTF/BCL3/FCER1G/LY96/TLR4/TLR2/ERRFI1/LILRB1/CD14/IFNGR1/PTPN6	11
GO:0035710	CD4-positive, alpha-beta T cell activation	11/182	49/749	0,678608622	0,999954951	0,999954951	BCL3/BCL6/STAT3/RELB/JAK3/IL4R/NFKBIZ/SOCS1/STAT6/RORC/GATA3	11
GO:0043122	regulation of I-kappaB kinase/NF-kappaB signaling	11/182	49/749	0,678608622	0,999954951	0,999954951	LTF/TNFRSF1A/LTBR/STAT1/FKBP1A/TLR4/MYD88/S100A12/TBK1/TNFSF10/BIRC3	11
GO:2000116	regulation of cysteine-type endopeptidase activity	11/182	50/749	0,706985069	0,999954951	0,999954951	S100A9/LTF/S100A8/BAX/MYC/TNFSF10/BIRC3/CASP4/ADORA2A/TNFSF14/PLAUR	11
GO:0002821	positive regulation of adaptive immune response	11/182	51/749	0,733778281	0,999954951	0,999954951	C3/FCER1G/IL1R1/NFKBIZ/TGFB1/STAT6/IL6R/SLC11A1/PVR/GATA3/IL6ST	11
GO:0043406	positive regulation of MAP kinase activity	11/182	51/749	0,733778281	0,999954951	0,999954951	FPR1/CD24/TGFB1/TLR4/KITLG/MAPK11/S100A12/FGD2/NOX4/C5AR1/GDF15	11
GO:0045089	positive regulation of innate immune response	11/182	51/749	0,733778281	0,999954951	0,999954951	FCER1G/PSMB8/MUC1/PSME2/PSMB10/RELB/IRF7/PVR/TBK1/PSME1/NFKB1	11
GO:0050715	positive regulation of cytokine secretion	11/182	52/749	0,758941505	0,999954951	0,999954951	IL4R/XBP1/IL17RA/TLR4/TLR2/MAPK11/CD58/CD14/GATA3/IFNGR1/IL17RB	11
GO:0060537	muscle tissue development	11/182	52/749	0,758941505	0,999954951	0,999954951	COL3A1/TGFB1/AGT/EGR1/FKBP1A/MAPK11/TIPARP/NOX4/G6PD/MYOM2/ATF3	11
GO:1903555	regulation of tumor necrosis factor superfamily cytokine produc	11/182	52/749	0,758941505	0,999954951	0,999954951	LTF/BCL3/FCER1G/LY96/TLR4/TLR2/ERRFI1/LILRB1/CD14/IFNGR1/PTPN6	11

GO:0051129	negative regulation of cellular component organization	11/182	53/749	0,782452624	0,999954951	0,999954951	SOST/TNFRSF1A/HDAC6/TGFB1/DNMT1/LDLR/APOE/TLR2/LILRB2/TNFRSF1B/LILRB1	11
GO:0071706	tumor necrosis factor superfamily cytokine production	11/182	53/749	0,782452624	0,999954951	0,999954951	LTF/BCL3/FCER1G/LY96/TLR4/TLR2/ERRFI1/LILRB1/CD14/IFNGR1/PTPN6	11
GO:0048545	response to steroid hormone	11/182	55/749	0,824538038	0,999954951	0,999954951	HDAC6/COL1A1/NR4A1/ICAM1/TGFB1/CDKN1A/TLR2/RORC/ABCC2/ERRFI1/EPO	11
GO:0050777	negative regulation of immune response	11/182	55/749	0,824538038	0,999954951	0,999954951	BCL6/SERPING1/COL3A1/JAK3/IL4R/TGFB1/STAT6/CD59/SLAMF8/LILRB1/PTPN6	11
GO:0051480	regulation of cytosolic calcium ion concentration	11/182	56/749	0,843167947	0,999954951	0,999954951	FPR1/CD24/TGFB1/BAX/AGT/FKBP1A/CMKLR1/C5AR1/TMEM178A/EPO/PTPN6	11
GO:0097530	granulocyte migration	11/182	56/749	0,843167947	0,999954951	0,999954951	S100A9/S100A8/FCER1G/IL1R1/IL17RA/SLAMF8/CXCL1/S100A12/CMKLR1/ITGB2/C5AR1	11
GO:0002695	negative regulation of leukocyte activation	11/182	59/749	0,890034149	0,999954951	0,999954951	BCL6/JAK3/IL4R/TGFB1/SOCS1/LDLR/LILRB2/LST1/LILRB1/ADORA2A/PTPN6	11
GO:0001906	cell killing	11/182	62/749	0,924886422	0,999954951	0,999954951	LTF/C3/C9/ICAM1/CD59/APOL1/PVR/S100A12/LILRB1/KIR3DL1/PTPN6	11
GO:0070372	regulation of ERK1 and ERK2 cascade	11/182	67/749	0,962301637	0,999954951	0,999954951	ICAM1/TGFB1/TLR4/APOE/ERRFI1/NOX4/C5AR1/FN1/EPO/ATF3/PTPN6	11
GO:0002706	regulation of lymphocyte mediated immunity	11/182	69/749	0,971890522	0,999954951	0,999954951	BCL6/C3/FCER1G/IL1R1/TGFB1/STAT6/PVR/TNFRSF1B/LILRB1/GATA3/PTPN6	11
GO:0045619	regulation of lymphocyte differentiation	11/182	69/749	0,971890522	0,999954951	0,999954951	BCL6/JAK3/IL4R/NFKBIZ/TGFB1/SOCS1/XBP1/SLAMF8/LILRB2/GATA3/PTPN6	11
GO:0050671	positive regulation of lymphocyte proliferation	11/182	76/749	0,990648868	0,999954951	0,999954951	BCL6/CD24/JAK3/CDKN1A/VCAM1/CD276/TLR4/LILRB2/IL6R/EPO/IL6ST	11
GO:0032946	positive regulation of mononuclear cell proliferation	11/182	77/749	0,992079237	0,999954951	0,999954951	BCL6/CD24/JAK3/CDKN1A/VCAM1/CD276/TLR4/LILRB2/IL6R/EPO/IL6ST	11
GO:0034655	nucleobase-containing compound catabolic process	10/182	25/749	0,057130794	0,999954951	0,999954951	PSMB8/PSME2/PSMB10/BAX/MYD88/SLC11A1/TNFRSF1B/ISG20/PSME1/RGN	10
GO:0019439	aromatic compound catabolic process	10/182	27/749	0,09309822	0,999954951	0,999954951	PSMB8/PSME2/PSMB10/BAX/MYD88/SLC11A1/TNFRSF1B/ISG20/PSME1/RGN	10
GO:0044270	cellular nitrogen compound catabolic process	10/182	27/749	0,09309822	0,999954951	0,999954951	PSMB8/PSME2/PSMB10/BAX/MYD88/SLC11A1/TNFRSF1B/ISG20/PSME1/RGN	10
GO:0046700	heterocycle catabolic process	10/182	27/749	0,09309822	0,999954951	0,999954951	PSMB8/PSME2/PSMB10/BAX/MYD88/SLC11A1/TNFRSF1B/ISG20/PSME1/RGN	10
GO:0050808	synapse organization	10/182	29/749	0,140040378	0,999954951	0,999954951	C1QB/C3/COL4A1/HDAC6/C1QA/TNC/APOE/TLR2/LILRB2/C5AR1	10
GO:0006956	complement activation	10/182	31/749	0,197206136	0,999954951	0,999954951	C1QB/C1S/C3/SERPING1/C9/CFB/C1QA/CD59/MASP1/C5AR1	10
GO:0050728	negative regulation of inflammatory response	10/182	31/749	0,197206136	0,999954951	0,999954951	SOCS3/TNFRSF1A/LDLR/SLAMF8/APOE/TNFRSF1B/IL1R2/GATA3/ADORA2A/NFKB1	10
GO:0060070	canonical Wnt signaling pathway	10/182	31/749	0,197206136	0,999954951	0,999954951	SOST/PSMB8/COL1A1/PSME2/PSMB10/EGR1/APOE/PSME1/GATA3/NFKB1	10
GO:1902106	negative regulation of leukocyte differentiation	10/182	31/749	0,197206136	0,999954951	0,999954951	LTF/BCL6/JAK3/IL4R/SOCS1/TLR4/MYC/TRIB1/LILRB1/TMEM178A	10
GO:1904062	regulation of cation transmembrane transport	10/182	31/749	0,197206136	0,999954951	0,999954951	EHD3/TGFB1/BAX/AGT/FKBP1A/CTSS/G6PD/EPO/RGN/PTPN6	10
GO:0002920	regulation of humoral immune response	10/182	32/749	0,229108078	0,999954951	0,999954951	C1QB/C1S/C3/SERPING1/C9/CFB/C1QA/CD59/C5AR1/PTPN6	10
GO:0030111	regulation of Wnt signaling pathway	10/182	33/749	0,262845868	0,999954951	0,999954951	SOST/PSMB8/COL1A1/PSME2/PSMB10/EGR1/APOE/TLR2/PSME1/NFKB1	10
GO:0032602	chemokine production	10/182	33/749	0,262845868	0,999954951	0,999954951	S100A9/HIF1A/S100A8/IL4R/SIGIRR/EGR1/TLR4/TLR2/IL6R/ACKR1	10
GO:0007265	Ras protein signal transduction	10/182	34/749	0,298091491	0,999954951	0,999954951	BCL6/COL3A1/DNMT1/CDKN1A/KITLG/APOE/MAPK11/ARHGDIB/FGD2/EPO	10
GO:0022411	cellular component disassembly	10/182	34/749	0,298091491	0,999954951	0,999954951	HIF1A/C1QB/TIMP1/C3/HDAC6/C1QA/TGFB1/BAX/CTSS/MYC	10
GO:0042088	T-helper 1 type immune response	10/182	34/749	0,298091491	0,999954951	0,999954951	BCL3/IL1R1/RELB/JAK3/IL4R/STAT6/IL18BP/TLR4/IL6R/SLC11A1	10
GO:0050792	regulation of viral process	10/182	34/749	0,298091491	0,999954951	0,999954951	LTF/IFITM3/SLPI/IFITM2/IFITM1/STAT1/APOE/ISG20/IFIT1/ISG15	10
GO:0097237	cellular response to toxic substance	10/182	34/749	0,298091491	0,999954951	0,999954951	S100A9/SOD2/MAPK13/LCN2/HDAC6/STAT6/EGR1/APOE/NFKB1/RGN	10
GO:1903707	negative regulation of hemopoiesis	10/182	34/749	0,298091491	0,999954951	0,999954951	LTF/BCL6/JAK3/IL4R/SOCS1/TLR4/MYC/TRIB1/LILRB1/TMEM178A	10
GO:0032787	monocarboxylic acid metabolic process	10/182	35/749	0,334494552	0,999954951	0,999954951	ALDH3A2/HIF1A/STAT3/C3/TNFRSF1A/XBP1/ALOX5/VCAN/ABCC2/RGN	10
GO:0042093	T-helper cell differentiation	10/182	35/749	0,334494552	0,999954951	0,999954951	BCL3/BCL6/STAT3/RELB/JAK3/IL4R/NFKBIZ/STAT6/RORC/GATA3	10
GO:1901615	organic hydroxy compound metabolic process	10/182	35/749	0,334494552	0,999954951	0,999954951	ALDH3A2/HIF1A/APOL1/LDLR/APOE/APOL2/ITGB2/G6PD/GATA3/NFKB1	10
GO:0002294	CD4-positive, alpha-beta T cell differentiation involved in immur	10/182	36/749	0,371693757	0,999954951	0,999954951	BCL3/BCL6/STAT3/RELB/JAK3/IL4R/NFKBIZ/STAT6/RORC/GATA3	10
GO:0007264	small GTPase mediated signal transduction	10/182	36/749	0,371693757	0,999954951	0,999954951	BCL6/COL3A1/DNMT1/CDKN1A/KITLG/APOE/MAPK11/ARHGDIB/FGD2/EPO	10
GO:0010608	posttranscriptional regulation of gene expression	10/182	36/749	0,371693757	0,999954951	0,999954951	BCL3/STAT3/PSMB8/PSME2/PSMB10/TGFB1/MYD88/SLC11A1/TNFRSF1B/PSME1	10
GO:0002287	alpha-beta T cell activation involved in immune response	10/182	37/749	0,409327736	0,999954951	0,999954951	BCL3/BCL6/STAT3/RELB/JAK3/IL4R/NFKBIZ/STAT6/RORC/GATA3	10
GO:0002293	alpha-beta T cell differentiation involved in immune response	10/182	37/749	0,409327736	0,999954951	0,999954951	BCL3/BCL6/STAT3/RELB/JAK3/IL4R/NFKBIZ/STAT6/RORC/GATA3	10
GO:0002478	antigen processing and presentation of exogenous peptide antig	10/182	37/749	0,409327736	0,999954951	0,999954951	FCER1G/PSMB8/PSME2/PSMB10/FCGR1A/IFI30/CTSS/TAP2/PSME1/TAP1	10
GO:0010817	regulation of hormone levels	10/182	37/749	0,409327736	0,999954951	0,999954951	HIF1A/MME/HSD11B1/AGT/EGR1/ABCC2/HNF1A/TIPARP/GATA3/NFKB1	10
GO:0030336	negative regulation of cell migration	10/182	37/749	0,409327736	0,999954951	0,999954951	TIMP1/STAT3/IFITM1/COL3A1/TGFB1/SLAMF8/APOE/ARHGDIB/TRIB1/SERPINE1	10
GO:0001654	eye development	10/182	38/749	0,447044776	0,999954951	0,999954951	HIF1A/STAT3/COL4A1/MEGF11/MAF/TGFB1/BAX/FJX1/MYOM2/GATA3	10
GO:0019884	antigen processing and presentation of exogenous antigen	10/182	38/749	0,447044776	0,999954951	0,999954951	FCER1G/PSMB8/PSME2/PSMB10/FCGR1A/IFI30/CTSS/TAP2/PSME1/TAP1	10
GO:0043903	regulation of interspecies interactions between organisms	10/182	39/749	0,48451121	0,999954951	0,999954951	LTF/IFITM3/SLPI/IFITM2/IFITM1/STAT1/APOE/ISG20/IFIT1/ISG15	10
GO:0061448	connective tissue development	10/182	40/749	0,521418285	0,999954951	0,999954951	HIF1A/TIMP1/COL1A1/MAF/TGFB1/XBP1/EGR1/IL6R/RORC/TGFBI	10
GO:0098660	inorganic ion transmembrane transport	10/182	40/749	0,521418285	0,999954951	0,999954951	EHD3/TGFB1/BAX/APOL1/SLC12A3/FKBP1A/SLC11A1/G6PD/RGN/PTPN6	10
GO:0010952	positive regulation of peptidase activity	10/182	41/749	0,557487442	0,999954951	0,999954951	S100A9/STAT3/S100A8/PSME2/BAX/MYC/TNFSF10/PSME1/FN1/CASP4	10
GO:0009896	positive regulation of catabolic process	10/182	42/749	0,592474014	0,999954951	0,999954951	HIF1A/HDAC6/BAX/LDLR/APOE/TNFRSF1B/TBK1/TRIB1/TIPARP/RGN	10
GO:0032675	regulation of interleukin-6 production	10/182	44/749	0,658402192	0,999954951	0,999954951	STAT3/FCER1G/MAPK13/XBP1/TLR4/MYD88/TLR2/LILRB2/IL6R/PTPN6	10
GO:0043123	positive regulation of I-kappaB kinase/NF-kappaB signaling	10/182	44/749	0,658402192	0,999954951	0,999954951	LTF/TNFRSF1A/LTBR/FKBP1A/TLR4/MYD88/S100A12/TBK1/TNFSF10/BIRC3	10
GO:0051701	interaction with host	10/182	45/749	0,689037337	0,999954951	0,999954951	IFITM3/ITGB6/IFITM2/IFITM1/HAVCR1/ICAM1/TGFB1/LDLR/PVR/IFIT1	10
GO:0006974	cellular response to DNA damage stimulus	10/182	46/749	0,717975364	0,999954951	0,999954951	BCL3/BCL6/MUC1/TNFRSF1A/BAX/CDKN1A/IRF7/TNFRSF1B/MYC/ISG15	10
GO:0032989	cellular component morphogenesis	10/182	46/749	0,717975364	0,999954951	0,999954951	KRT8/HDAC6/BAX/APOE/TLR2/KRT19/FN1/MYOM2/GATA3/ADORA2A	10
GO:0034599	cellular response to oxidative stress	10/182	46/749	0,717975364	0,999954951	0,999954951	HIF1A/SOD2/MAPK13/LCN2/HDAC6/STAT6/TLR4/NOX4/G6PD/RGN	10
GO:0043281	regulation of cysteine-type endopeptidase activity involved in ap	10/182	46/749	0,717975364	0,999954951	0,999954951	S100A9/S100A8/BAX/MYC/TNFSF10/BIRC3/CASP4/ADORA2A/TNFSF14/PLAUR	10
GO:0032635	interleukin-6 production	10/182	47/749	0,74515003	0,999954951	0,999954951	STAT3/FCER1G/MAPK13/XBP1/TLR4/MYD88/TLR2/LILRB2/IL6R/PTPN6	10
GO:0033002	muscle cell proliferation	10/182	47/749	0,74515003	0,999954951	0,999954951	SOD2/STAT3/ADAMTS1/STAT1/DNMT1/CDKN1A/AGT/MAPK11/IL6R/TRIB1	10

GO:0044057	regulation of system process	10/182	47/749	0,74515003	0,999954951	0,999954951	TNFRSF1A/EHD3/ICAM1/AGT/APOE/TNFRSF1B/ERRFI1/G6PD/ADORA2A/IL6ST	10
GO:0051259	protein complex oligomerization	10/182	48/749	0,770525684	0,999954951	0,999954951	SOD2/C9/FCER1G/EHD3/LCN2/HDAC6/COL1A1/BAX/APOE/BIRC3	10
GO:0030036	actin cytoskeleton organization	10/182	49/749	0,794094162	0,999954951	0,999954951	BCL6/KRT8/ICAM1/TGFB1/CXCL1/ARHGDIB/FGD2/KRT19/NOX4/MYOM2	10
GO:2001234	negative regulation of apoptotic signaling pathway	10/182	49/749	0,794094162	0,999954951	0,999954951	HIF1A/SOD2/MUC1/ICAM1/XBP1/BAX/TNFSF10/EPO/SERPINE1/PLAUR	10
GO:0002824	positive regulation of adaptive immune response based on soma	10/182	50/749	0,815871435	0,999954951	0,999954951	C3/FCER1G/IL1R1/NFKBIZ/TGFB1/STAT6/IL6R/SLC11A1/PVR/GATA3	10
GO:0019637	organophosphate metabolic process	10/182	51/749	0,835894156	0,999954951	0,999954951	HIF1A/PLAAT4/STAT3/NNMT/SOCS3/IMPDH1/TGFB1/SOCS1/LDLR/G6PD	10
GO:0032147	activation of protein kinase activity	10/182	51/749	0,835894156	0,999954951	0,999954951	FPR1/SOCS1/AGT/TLR4/MAPK11/IL6R/SLC11A1/C5AR1/GDF15/EPO	10
GO:0030029	actin filament-based process	10/182	52/749	0,854216215	0,999954951	0,999954951	BCL6/KRT8/ICAM1/TGFB1/CXCL1/ARHGDIB/FGD2/KRT19/NOX4/MYOM2	10
GO:0007204	positive regulation of cytosolic calcium ion concentration	10/182	54/749	0,886040371	0,999954951	0,999954951	FPR1/CD24/TGFB1/BAX/AGT/FKBP1A/CMKLR1/C5AR1/EPO/PTPN6	10
GO:0051250	negative regulation of lymphocyte activation	10/182	54/749	0,886040371	0,999954951	0,999954951	BCL6/JAK3/IL4R/TGFB1/SOCS1/LILRB2/LST1/LILRB1/ADORA2A/PTPN6	10
GO:0032649	regulation of interferon-gamma production	10/182	57/749	0,923008834	0,999954951	0,999954951	BCL3/IL1R1/CD276/TLR4/IL6R/SLC11A1/LILRB1/CD14/GATA3/ISG15	10
GO:0031175	neuron projection development	10/182	58/749	0,932833561	0,999954951	0,999954951	S100A9/HDAC6/AGT/TNC/APOE/LST1/FN1/EPO/GATA3/ADORA2A	10
GO:0032609	interferon-gamma production	10/182	59/749	0,941568135	0,999954951	0,999954951	BCL3/IL1R1/CD276/TLR4/IL6R/SLC11A1/LILRB1/CD14/GATA3/ISG15	10
GO:0002685	regulation of leukocyte migration	10/182	62/749	0,962145854	0,999954951	0,999954951	IL1R1/ICAM1/TGFB1/KITLG/SLAMF8/IL6R/CMKLR1/C5AR1/SERPINE1/TNFSF14	10
GO:0045765	regulation of angiogenesis	10/182	64/749	0,972022629	0,999954951	0,999954951	HIF1A/JAK1/STAT3/C3/STAT1/XBP1/AGT/ITGB2/C5AR1/SERPINE1	10
GO:1902107	positive regulation of leukocyte differentiation	10/182	65/749	0,976037214	0,999954951	0,999954951	BCL6/IL4R/NFKBIZ/TGFB1/SOCS1/XBP1/KITLG/LILRB2/TRIB1/GATA3	10
GO:0050851	antigen receptor-mediated signaling pathway	10/182	74/749	0,994639108	0,999954951	0,999954951	PSMB8/PSME2/PSMB10/NFKBIZ/BAX/CD276/PSME1/GATA3/NFKB1/PTPN6	10
GO:0060341	regulation of cellular localization	10/182	79/749	0,997831808	0,999954951	0,999954951	HIF1A/FCER1G/IL4R/TGFB1/BAX/FKBP1A/ITGB2/EPO/ADORA2A/PTPN6	10
GO:0048708	astrocyte differentiation	9/182	21/749	0,045280773	0,999954951	0,999954951	STAT3/S100A8/C1QA/LDLR/TLR4/C5AR1/IFNGR1/ADORA2A/IL6ST	9
GO:0002532	production of molecular mediator involved in inflammatory resp	9/182	22/749	0,0609463	0,999954951	0,999954951	FCER1G/IL4R/ALOX5/IL17RA/TLR4/SLAMF8/MYD88/IL1R2/SERPINE1	9
GO:0019941	modification-dependent protein catabolic process	9/182	22/749	0,0609463	0,999954951	0,999954951	PSMB8/HDAC6/PSME2/PSMB10/XBP1/TRIB1/PSME1/ISG15/RNF149	9
GO:0042590	antigen processing and presentation of exogenous peptide antig	9/182	22/749	0,0609463	0,999954951	0,999954951	FCER1G/PSMB8/PSME2/PSMB10/FCGR1A/IFI30/TAP2/PSME1/TAP1	9
GO:0043632	modification-dependent macromolecule catabolic process	9/182	22/749	0,0609463	0,999954951	0,999954951	PSMB8/HDAC6/PSME2/PSMB10/XBP1/TRIB1/PSME1/ISG15/RNF149	9
GO:0002474	antigen processing and presentation of peptide antigen via MHC	9/182	23/749	0,079705531	0,999954951	0,999954951	FCER1G/PSMB8/PSME2/PSMB10/FCGR1A/IFI30/TAP2/PSME1/TAP1	9
GO:0016071	mRNA metabolic process	9/182	23/749	0,079705531	0,999954951	0,999954951	HIF1A/STAT3/PSMB8/PSME2/PSMB10/MYD88/SLC11A1/C5AR1/PSME1	9
GO:0030449	regulation of complement activation	9/182	25/749	0,126638016	0,999954951	0,999954951	C1QB/C1S/C3/SERPING1/C9/CFB/C1QA/CD59/C5AR1	9
GO:0060828	regulation of canonical Wnt signaling pathway	9/182	25/749	0,126638016	0,999954951	0,999954951	SOST/PSMB8/COL1A1/PSME2/PSMB10/EGR1/APOE/PSME1/NFKB1	9
GO:0070498	interleukin-1-mediated signaling pathway	9/182	25/749	0,126638016	0,999954951	0,999954951	PSMB8/IL1R1/PSME2/PSMB10/EGR1/MYD88/IL1R2/PSME1/NFKB1	9
GO:2001242	regulation of intrinsic apoptotic signaling pathway	9/182	25/749	0,126638016	0,999954951	0,999954951	S100A9/HIF1A/SOD2/S100A8/MUC1/XBP1/BAX/EPO/PLAUR	9
GO:0019730	antimicrobial humoral response	9/182	26/749	0,154643079	0,999954951	0,999954951	S100A9/LTF/BCL3/S100A8/SLPI/LCN2/CXCL1/SLC11A1/S100A12	9
GO:0030218	erythrocyte differentiation	9/182	26/749	0,154643079	0,999954951	0,999954951	HIF1A/BCL6/STAT3/JAK3/STAT1/G6PD/EPO/GATA3/ISG15	9
GO:0034101	erythrocyte homeostasis	9/182	27/749	0,185415509	0,999954951	0,999954951	HIF1A/BCL6/STAT3/JAK3/STAT1/G6PD/EPO/GATA3/ISG15	9
GO:0061041	regulation of wound healing	9/182	27/749	0,185415509	0,999954951	0,999954951	SERPING1/FCER1G/XBP1/CDKN1A/TLR4/APOE/SERPINE1/THBD/PLAUR	9
GO:0006469	negative regulation of protein kinase activity	9/182	28/749	0,218666692	0,999954951	0,999954951	SOCS3/SOCS1/CDKN1A/APOE/TRIB1/ERRFI1/ADORA2A/RGN/PTPN6	9
GO:0033673	negative regulation of kinase activity	9/182	28/749	0,218666692	0,999954951	0,999954951	SOCS3/SOCS1/CDKN1A/APOE/TRIB1/ERRFI1/ADORA2A/RGN/PTPN6	9
GO:0045926	negative regulation of growth	9/182	28/749	0,218666692	0,999954951	0,999954951	HIF1A/BCL6/TGFB1/CDKN1A/AGT/MT1A/MAPK11/G6PD/GDF15	9
GO:0045930	negative regulation of mitotic cell cycle	9/182	28/749	0,218666692	0,999954951	0,999954951	BCL6/PSMB8/MUC1/PSME2/PSMB10/TGFB1/BAX/CDKN1A/PSME1	9
GO:0006820	anion transport	9/182	29/749	0,254048534	0,999954951	0,999954951	ABCB1/AGT/APOL1/LDLR/SLC12A3/APOE/SLC11A1/ABCC2/ADORA2A	9
GO:0048863	stem cell differentiation	9/182	29/749	0,254048534	0,999954951	0,999954951	HIF1A/STAT3/PSMB8/PSME2/PSMB10/KITLG/PSME1/FN1/GATA3	9
GO:0051651	maintenance of location in cell	9/182	29/749	0,254048534	0,999954951	0,999954951	S100A9/BCL3/S100A8/LCN2/TGFB1/BAX/FKBP1A/APOE/PTPN6	9
GO:1903034	regulation of response to wounding	9/182	29/749	0,254048534	0,999954951	0,999954951	SERPING1/FCER1G/XBP1/CDKN1A/TLR4/APOE/SERPINE1/THBD/PLAUR	9
GO:0051348	negative regulation of transferase activity	9/182	30/749	0,291169046	0,999954951	0,999954951	SOCS3/SOCS1/CDKN1A/APOE/TRIB1/ERRFI1/ADORA2A/RGN/PTPN6	9
GO:0010876	lipid localization	9/182	31/749	0,329608419	0,999954951	0,999954951	C3/ABCB1/AGT/APOL1/LDLR/APOE/APOL2/ABCC2/NFKB1	9
GO:0036294	cellular response to decreased oxygen levels	9/182	31/749	0,329608419	0,999954951	0,999954951	HIF1A/PSMB8/PSME2/ICAM1/PSMB10/EGR1/MYC/PSME1/EPO	9
GO:0071229	cellular response to acid chemical	9/182	31/749	0,329608419	0,999954951	0,999954951	COL4A1/COL1A1/COL3A1/SOCS1/XBP1/DNMT1/LDLR/TNC/EGR1	9
GO:0071456	cellular response to hypoxia	9/182	31/749	0,329608419	0,999954951	0,999954951	HIF1A/PSMB8/PSME2/ICAM1/PSMB10/EGR1/MYC/PSME1/EPO	9
GO:1901654	response to ketone	9/182	31/749	0,329608419	0,999954951	0,999954951	ICAM1/TGFB1/AHR/CDKN1A/TNC/TLR2/ABCC2/ERRFI1/EPO	9
GO:2001235	positive regulation of apoptotic signaling pathway	9/182	31/749	0,329608419	0,999954951	0,999954951	S100A9/S100A8/LTBR/BAX/AGT/TNFSF10/ATF3/ADORA2A/PLAUR	9
GO:0005975	carbohydrate metabolic process	9/182	32/749	0,368934639	0,999954951	0,999954951	HIF1A/STAT3/TGFB1/RORC/G6PD/ATF3/NFKB1/IL6ST/RGN	9
GO:0021700	developmental maturation	9/182	32/749	0,368934639	0,999954951	0,999954951	HIF1A/LTF/C3/C1QA/XBP1/CDKN1A/G6PD/EPO/GATA3	9
GO:0032755	positive regulation of interleukin-6 production	9/182	32/749	0,368934639	0,999954951	0,999954951	STAT3/FCER1G/MAPK13/XBP1/TLR4/MYD88/TLR2/LILRB2/IL6R	9
GO:0071260	cellular response to mechanical stimulus	9/182	32/749	0,368934639	0,999954951	0,999954951	TNFRSF1A/LTBR/COL1A1/TGFB1/AGT/TLR4/MYD88/TNFSF14/NFKB1	9
GO:0071695	anatomical structure maturation	9/182	32/749	0,368934639	0,999954951	0,999954951	HIF1A/LTF/C3/C1QA/XBP1/CDKN1A/G6PD/EPO/GATA3	9
GO:0043010	camera-type eye development	9/182	33/749	0,408717928	0,999954951	0,999954951	HIF1A/COL4A1/MEGF11/MAF/TGFB1/BAX/FJX1/MYOM2/GATA3	9
GO:0010950	positive regulation of endopeptidase activity	9/182	34/749	0,448543445	0,999954951	0,999954951	S100A9/STAT3/S100A8/PSME2/BAX/MYC/TNFSF10/PSME1/CASP4	9
GO:0051147	regulation of muscle cell differentiation	9/182	34/749	0,448543445	0,999954951	0,999954951	SOD2/IL4R/TGFB1/XBP1/DNMT1/MAPK11/G6PD/GDF15/TNFSF14	9
GO:0071453	cellular response to oxygen levels	9/182	34/749	0,448543445	0,999954951	0,999954951	HIF1A/PSMB8/PSME2/ICAM1/PSMB10/EGR1/MYC/PSME1/EPO	9
GO:0042116	macrophage activation	9/182	35/749	0,488021909	0,999954951	0,999954951	C1QA/IL4R/LDLR/TLR4/TLR2/SLC11A1/ITGB2/C5AR1/IFNGR1	9
GO:0042176	regulation of protein catabolic process	9/182	35/749	0,488021909	0,999954951	0,999954951	TIMP1/PSME2/LDLR/APOE/TNFRSF1B/TRIB1/TIPARP/PSME1/RGN	9

GO:1901215	negative regulation of neuron death	9/182	35/749	0,488021909	0,999954951	0,999954951	HIF1A/SOD2/STAT3/BAX/APOE/TNFRSF1B/C5AR1/EPO/ADORA2A	9
GO:0043270	positive regulation of ion transport	9/182	37/749	0,564556245	0,999954951	0,999954951	EHD3/ABCB1/BAX/AGT/CTSS/APOE/G6PD/ADORA2A/RGN	9
GO:0051052	regulation of DNA metabolic process	9/182	37/749	0,564556245	0,999954951	0,999954951	BCL6/TGFB1/BAX/CDKN1A/STAT6/MYC/NOX4/GATA3/RGN	9
GO:0098662	inorganic cation transmembrane transport	9/182	37/749	0,564556245	0,999954951	0,999954951	EHD3/TGFB1/BAX/SLC12A3/FKBP1A/SLC11A1/G6PD/RGN/PTPN6	9
GO:0002761	regulation of myeloid leukocyte differentiation	9/182	38/749	0,601025322	0,999954951	0,999954951	LTF/TGFB1/TLR4/KITLG/IRF7/MYC/TRIB1/LILRB1/TMEM178A	9
GO:0046427	positive regulation of receptor signaling pathway via JAK-STAT	9/182	38/749	0,601025322	0,999954951	0,999954951	STAT3/SOCS3/TNFRSF1A/SOCS1/AGT/IL6R/IL10RB/EPO/IL6ST	9
GO:0010038	response to metal ion	9/182	39/749	0,635979556	0,999954951	0,999954951	HIF1A/S100A8/ICAM1/C1QA/VCAM1/MT1A/ABCC2/G6PD/CD14	9
GO:0031331	positive regulation of cellular catabolic process	9/182	39/749	0,635979556	0,999954951	0,999954951	HIF1A/HDAC6/BAX/LDLR/APOE/TNFRSF1B/TBK1/TRIB1/RGN	9
GO:0043603	cellular amide metabolic process	9/182	39/749	0,635979556	0,999954951	0,999954951	BCL3/PLAAT4/STAT3/TNFRSF1A/MME/APOE/ABCC2/G6PD/IFNGR1	9
GO:1904894	positive regulation of receptor signaling pathway via STAT	9/182	39/749	0,635979556	0,999954951	0,999954951	STAT3/SOCS3/TNFRSF1A/SOCS1/AGT/IL6R/IL10RB/EPO/IL6ST	9
GO:0002698	negative regulation of immune effector process	9/182	41/749	0,700669484	0,999954951	0,999954951	BCL6/SERPING1/JAK3/IL4R/TGFB1/CD59/SLAMF8/LILRB1/PTPN6	9
GO:0002718	regulation of cytokine production involved in immune response	9/182	41/749	0,700669484	0,999954951	0,999954951	BCL6/FCER1G/IL1R1/JAK3/TGFB1/TLR4/TNFRSF1B/LILRB1/GATA3	9
GO:0002702	positive regulation of production of molecular mediator of immu	9/182	42/749	0,730177339	0,999954951	0,999954951	FCER1G/IL1R1/IL4R/TGFB1/XBP1/STAT6/TLR4/LILRB1/GATA3	9
GO:0050868	negative regulation of T cell activation	9/182	45/749	0,806796707	0,999954951	0,999954951	BCL6/JAK3/IL4R/TGFB1/SOCS1/LILRB2/LILRB1/ADORA2A/PTPN6	9
GO:0051403	stress-activated MAPK cascade	9/182	45/749	0,806796707	0,999954951	0,999954951	MAPK13/LTBR/AGT/TLR4/MAPK11/MYC/FGD2/TRIB1/NFKB1	9
GO:0031098	stress-activated protein kinase signaling cascade	9/182	46/749	0,828401899	0,999954951	0,999954951	MAPK13/LTBR/AGT/TLR4/MAPK11/MYC/FGD2/TRIB1/NFKB1	9
GO:1990266	neutrophil migration	9/182	46/749	0,828401899	0,999954951	0,999954951	S100A9/S100A8/FCER1G/IL1R1/SLAMF8/CXCL1/S100A12/ITGB2/C5AR1	9
GO:0031589	cell-substrate adhesion	9/182	48/749	0,8660257	0,999954951	0,999954951	BCL6/ITGB6/LYVE1/COL1A1/COL3A1/VCAM1/ITGB2/FN1/SERPINE1	9
GO:1903038	negative regulation of leukocyte cell-cell adhesion	9/182	48/749	0,8660257	0,999954951	0,999954951	BCL6/JAK3/IL4R/TGFB1/SOCS1/LILRB2/LILRB1/ADORA2A/PTPN6	9
GO:0002687	positive regulation of leukocyte migration	9/182	49/749	0,882210296	0,999954951	0,999954951	IL1R1/ICAM1/TGFB1/KITLG/IL6R/CMKLR1/C5AR1/SERPINE1/TNFSF14	9
GO:0007517	muscle organ development	9/182	49/749	0,882210296	0,999954951	0,999954951	COL3A1/TGFB1/XBP1/EGR1/FKBP1A/MAPK11/G6PD/MYOM2/ATF3	9
GO:0071621	granulocyte chemotaxis	9/182	49/749	0,882210296	0,999954951	0,999954951	S100A9/S100A8/FCER1G/IL17RA/CXCL1/S100A12/CMKLR1/ITGB2/C5AR1	9
GO:0014706	striated muscle tissue development	9/182	50/749	0,896772485	0,999954951	0,999954951	TGFB1/AGT/EGR1/FKBP1A/MAPK11/NOX4/G6PD/MYOM2/ATF3	9
GO:0050852	T cell receptor signaling pathway	9/182	50/749	0,896772485	0,999954951	0,999954951	PSMB8/PSME2/PSMB10/NFKBIZ/CD276/PSME1/GATA3/NFKB1/PTPN6	9
GO:0000302	response to reactive oxygen species	9/182	51/749	0,909817841	0,999954951	0,999954951	SOD2/MAPK13/LCN2/HDAC6/COL1A1/STAT1/STAT6/APOE/RGN	9
GO:0032504	multicellular organism reproduction	9/182	51/749	0,909817841	0,999954951	0,999954951	BCL6/ADAMTS1/IL4R/TGFB1/BAX/EGR1/APOL2/BIRC3/RGN	9
GO:0048609	multicellular organismal reproductive process	9/182	51/749	0,909817841	0,999954951	0,999954951	BCL6/ADAMTS1/IL4R/TGFB1/BAX/EGR1/APOL2/BIRC3/RGN	9
GO:0046634	regulation of alpha-beta T cell activation	9/182	52/749	0,921455391	0,999954951	0,999954951	BCL6/JAK3/IL4R/NFKBIZ/SOCS1/IL6R/LILRB1/GATA3/ADORA2A	9
GO:0051098	regulation of binding	9/182	54/749	0,940946115	0,999954951	0,999954951	BCL3/SLPI/TGFB1/BAX/FKBP1A/APOE/IFIT1/GATA3/PLAUR	9
GO:0022408	negative regulation of cell-cell adhesion	9/182	55/749	0,949014528	0,999954951	0,999954951	BCL6/JAK3/IL4R/TGFB1/SOCS1/LILRB2/LILRB1/ADORA2A/PTPN6	9
GO:0048732	gland development	9/182	57/749	0,962307742	0,999954951	0,999954951	HIF1A/TGFB1/XBP1/BAX/STAT6/TNC/HNF1A/GATA3/RGN	9
GO:0050864	regulation of B cell activation	9/182	57/749	0,962307742	0,999954951	0,999954951	BCL6/TGFB1/XBP1/AHR/CDKN1A/STAT6/TLR4/SLAMF8/PTPN6	9
GO:0060322	head development	9/182	57/749	0,962307742	0,999954951	0,999954951	HIF1A/COL4A1/COL1A1/COL3A1/TGFB1/BAX/TIPARP/C5AR1/G6PD	9
GO:0002705	positive regulation of leukocyte mediated immunity	9/182	60/749	0,976509522	0,999954951	0,999954951	C3/FCER1G/IL1R1/IL4R/TGFB1/STAT6/PVR/ITGB2/GATA3	9
GO:0045580	regulation of T cell differentiation	9/182	61/749	0,980035662	0,999954951	0,999954951	BCL6/JAK3/IL4R/NFKBIZ/TGFB1/SOCS1/XBP1/LILRB2/GATA3	9
GO:0019932	second-messenger-mediated signaling	9/182	66/749	0,991454784	0,999954951	0,999954951	FPR1/AHR/VCAM1/AGT/FKBP1A/APOE/CMKLR1/ADORA2A/RGN	9
GO:0055076	transition metal ion homeostasis	8/182	18/749	0,046859599	0,999954951	0,999954951	S100A9/HIF1A/LTF/S100A8/LCN2/MT1A/SLC11A1/MYC	8
GO:0090183	regulation of kidney development	8/182	18/749	0,046859599	0,999954951	0,999954951	CD24/STAT1/TGFB1/AGT/EGR1/IL6R/MYC/GATA3	8
GO:0015850	organic hydroxy compound transport	8/182	19/749	0,064537124	0,999954951	0,999954951	FCER1G/AGT/LDLR/APOE/ABCC2/LILRB1/ADORA2A/NFKB1	8
GO:0006511	ubiquitin-dependent protein catabolic process	8/182	21/749	0,110823899	0,999954951	0,999954951	PSMB8/HDAC6/PSME2/PSMB10/XBP1/TRIB1/PSME1/RNF149	8
GO:0045069	regulation of viral genome replication	8/182	21/749	0,110823899	0,999954951	0,999954951	LTF/IFITM3/SLPI/IFITM2/IFITM1/ISG20/IFIT1/ISG15	8
GO:1905330	regulation of morphogenesis of an epithelium	8/182	21/749	0,110823899	0,999954951	0,999954951	PSMB8/PSME2/PSMB10/STAT1/TGFB1/AGT/PSME1/GATA3	8
GO:0002220	innate immune response activating cell surface receptor signalin	8/182	22/749	0,139325384	0,999954951	0,999954951	FCER1G/PSMB8/MUC1/PSME2/PSMB10/RELB/PSME1/NFKB1	8
GO:0002223	stimulatory C-type lectin receptor signaling pathway	8/182	22/749	0,139325384	0,999954951	0,999954951	FCER1G/PSMB8/MUC1/PSME2/PSMB10/RELB/PSME1/NFKB1	8
GO:0002758	innate immune response-activating signal transduction	8/182	22/749	0,139325384	0,999954951	0,999954951	FCER1G/PSMB8/MUC1/PSME2/PSMB10/RELB/PSME1/NFKB1	8
GO:0010948	negative regulation of cell cycle process	8/182	22/749	0,139325384	0,999954951	0,999954951	BCL6/PSMB8/MUC1/PSME2/PSMB10/BAX/CDKN1A/PSME1	8
GO:0002244	hematopoietic progenitor cell differentiation	8/182	23/749	0,171122639	0,999954951	0,999954951	PSMB8/PSME2/PSMB10/TGFB1/KITLG/PSME1/GATA3/PTPN6	8
GO:0043112	receptor metabolic process	8/182	23/749	0,171122639	0,999954951	0,999954951	HIF1A/FCER1G/EHD3/HDAC6/TGFB1/APOE/ITGB2/LILRB1	8
GO:0016579	protein deubiquitination	8/182	24/749	0,20587625	0,999954951	0,999954951	HIF1A/PSMB8/PSME2/PSMB10/MYC/BIRC3/PSME1/GATA3	8
GO:0019079	viral genome replication	8/182	24/749	0,20587625	0,999954951	0,999954951	LTF/IFITM3/SLPI/IFITM2/IFITM1/ISG20/IFIT1/ISG15	8
GO:0048469	cell maturation	8/182	24/749	0,20587625	0,999954951	0,999954951	HIF1A/C3/C1QA/XBP1/CDKN1A/G6PD/EPO/GATA3	8
GO:0070646	protein modification by small protein removal	8/182	24/749	0,20587625	0,999954951	0,999954951	HIF1A/PSMB8/PSME2/PSMB10/MYC/BIRC3/PSME1/GATA3	8
GO:1903900	regulation of viral life cycle	8/182	24/749	0,20587625	0,999954951	0,999954951	LTF/IFITM3/SLPI/IFITM2/IFITM1/ISG20/IFIT1/ISG15	8
GO:0000082	G1/S transition of mitotic cell cycle	8/182	25/749	0,243164831	0,999954951	0,999954951	ADAMTS1/MUC1/PSME2/BAX/CDKN1A/MYC/PSME1/PTPN6	8
GO:0002218	activation of innate immune response	8/182	25/749	0,243164831	0,999954951	0,999954951	FCER1G/PSMB8/MUC1/PSME2/PSMB10/RELB/PSME1/NFKB1	8
GO:0044843	cell cycle G1/S phase transition	8/182	25/749	0,243164831	0,999954951	0,999954951	ADAMTS1/MUC1/PSME2/BAX/CDKN1A/MYC/PSME1/PTPN6	8
GO:0006869	lipid transport	8/182	26/749	0,282507831	0,999954951	0,999954951	ABCB1/AGT/APOL1/LDLR/APOE/APOL2/ABCC2/NFKB1	8
GO:0034764	positive regulation of transmembrane transport	8/182	26/749	0,282507831	0,999954951	0,999954951	C3/EHD3/ABCB1/BAX/AGT/CTSS/G6PD/RGN	8
GO:0031663	lipopolysaccharide-mediated signaling pathway	8/182	28/749	0,365277656	0,999954951	0,999954951	LTF/TGFB1/SIGIRR/LY96/TLR4/TLR2/TRIB1/CD14	8

GO:0032642	regulation of chemokine production	8/182	28/749	0,365277656	0,999954951	0,999954951	HIF1A/IL4R/SIGIRR/EGR1/TLR4/TLR2/IL6R/ACKR1	8
GO:1901137	carbohydrate derivative biosynthetic process	8/182	28/749	0,365277656	0,999954951	0,999954951	STAT3/MUC1/IMPDH1/JAK3/TGFB1/VCAN/G6PD/NFKB1	8
GO:0007005	mitochondrion organization	8/182	29/749	0,407650655	0,999954951	0,999954951	HIF1A/SOD2/STAT3/HDAC6/BAX/TNFSF10/ALAS1/PLAUR	8
GO:0030522	intracellular receptor signaling pathway	8/182	29/749	0,407650655	0,999954951	0,999954951	STAT3/HDAC6/NR4A1/AHR/TLR4/IRF7/RORC/BIRC3	8
GO:0048511	rhythmic process	8/182	29/749	0,407650655	0,999954951	0,999954951	ADAMTS1/RELB/AHR/EGR1/NFKB2/RORC/SERPINE1/ADORA2A	8
GO:0050890	cognition	8/182	29/749	0,407650655	0,999954951	0,999954951	HIF1A/MME/AGT/LDLR/APOE/TLR2/LILRB2/C5AR1	8
GO:0002455	humoral immune response mediated by circulating immunoglob	8/182	30/749	0,450007603	0,999954951	0,999954951	BCL3/C1QB/C1S/C3/SERPING1/C9/C1QA/PTPN6	8
GO:0007565	female pregnancy	8/182	30/749	0,450007603	0,999954951	0,999954951	TIMP1/TGFB1/AGT/APOL2/ARHGDIB/ABCC2/EPO/THBD	8
GO:0031668	cellular response to extracellular stimulus	8/182	30/749	0,450007603	0,999954951	0,999954951	LCN2/COL1A1/ICAM1/XBP1/CDKN1A/VCAM1/TNC/ATF3	8
GO:0044706	multi-multicellular organism process	8/182	30/749	0,450007603	0,999954951	0,999954951	TIMP1/TGFB1/AGT/APOL2/ARHGDIB/ABCC2/EPO/THBD	8
GO:0010721	negative regulation of cell development	8/182	31/749	0,49188558	0,999954951	0,999954951	LTF/STAT3/COL3A1/TGFB1/LDLR/APOE/G6PD/LILRB1	8
GO:0072006	nephron development	8/182	31/749	0,49188558	0,999954951	0,999954951	CD24/STAT1/TGFB1/AGT/EGR1/IL6R/MYC/GATA3	8
GO:2000379	positive regulation of reactive oxygen species metabolic process	8/182	31/749	0,49188558	0,999954951	0,999954951	ICAM1/TGFB1/CDKN1A/AGT/TLR4/ITGB2/NOX4/RGN	8
GO:0150076	neuroinflammatory response	8/182	32/749	0,532869227	0,999954951	0,999954951	C1QA/LDLR/TLR2/TNFRSF1B/ITGB2/C5AR1/IFNGR1/ADORA2A	8
GO:0001505	regulation of neurotransmitter levels	8/182	33/749	0,572597643	0,999954951	0,999954951	ICAM1/AGT/TLR4/TLR2/ITGB2/SLC22A2/ADORA2A/RGN	8
GO:0046718	viral entry into host cell	8/182	33/749	0,572597643	0,999954951	0,999954951	IFITM3/ITGB6/IFITM2/IFITM1/HAVCR1/ICAM1/LDLR/PVR	8
GO:0030278	regulation of ossification	8/182	34/749	0,610768165	0,999954951	0,999954951	HIF1A/LTF/IFITM1/SOST/TGFB1/IL6R/IL6ST/ISG15	8
GO:0072657	protein localization to membrane	8/182	34/749	0,610768165	0,999954951	0,999954951	CD24/FCER1G/TNFRSF1A/EHD3/TGFB1/BAX/APOE/ITGB2	8
GO:0007160	cell-matrix adhesion	8/182	35/749	0,647137426	0,999954951	0,999954951	BCL6/ITGB6/LYVE1/COL3A1/VCAM1/ITGB2/FN1/SERPINE1	8
GO:0007260	tyrosine phosphorylation of STAT protein	8/182	35/749	0,647137426	0,999954951	0,999954951	STAT3/SOCS3/TNFRSF1A/JAK3/SOCS1/IL6R/EPO/IL6ST	8
GO:0032615	interleukin-12 production	8/182	35/749	0,647137426	0,999954951	0,999954951	JAK3/TLR4/TLR2/MAPK11/CMKLR1/LILRB1/NFKB1/LTB	8
GO:0032655	regulation of interleukin-12 production	8/182	35/749	0,647137426	0,999954951	0,999954951	JAK3/TLR4/TLR2/MAPK11/CMKLR1/LILRB1/NFKB1/LTB	8
GO:0043523	regulation of neuron apoptotic process	8/182	35/749	0,647137426	0,999954951	0,999954951	HIF1A/SOD2/BAX/APOE/C5AR1/G6PD/GATA3/ADORA2A	8
GO:0030183	B cell differentiation	8/182	36/749	0,681520131	0,999954951	0,999954951	BCL3/BCL6/JAK3/XBP1/BAX/VCAM1/SLAMF8/PTPN6	8
GO:0003012	muscle system process	8/182	37/749	0,713786031	0,999954951	0,999954951	TNFRSF1A/EHD3/AGT/TNFRSF1B/ERRFI1/G6PD/MYOM2/IL6ST	8
GO:0044409	entry into host	8/182	37/749	0,713786031	0,999954951	0,999954951	IFITM3/ITGB6/IFITM2/IFITM1/HAVCR1/ICAM1/LDLR/PVR	8
GO:0051402	neuron apoptotic process	8/182	37/749	0,713786031	0,999954951	0,999954951	HIF1A/SOD2/BAX/APOE/C5AR1/G6PD/GATA3/ADORA2A	8
GO:0052126	movement in host environment	8/182	37/749	0,713786031	0,999954951	0,999954951	IFITM3/ITGB6/IFITM2/IFITM1/HAVCR1/ICAM1/LDLR/PVR	8
GO:2000106	regulation of leukocyte apoptotic process	8/182	37/749	0,713786031	0,999954951	0,999954951	HIF1A/BCL6/FCER1G/JAK3/BAX/KITLG/IRF7/LILRB1	8
GO:0048659	smooth muscle cell proliferation	8/182	39/749	0,77169452	0,999954951	0,999954951	SOD2/ADAMTS1/STAT1/DNMT1/CDKN1A/AGT/IL6R/TRIB1	8
GO:0048660	regulation of smooth muscle cell proliferation	8/182	39/749	0,77169452	0,999954951	0,999954951	SOD2/ADAMTS1/STAT1/DNMT1/CDKN1A/AGT/IL6R/TRIB1	8
GO:0031344	regulation of cell projection organization	8/182	40/749	0,797308331	0,999954951	0,999954951	S100A9/LCN2/ICAM1/AGT/APOE/FN1/EPO/GATA3	8
GO:0038093	Fc receptor signaling pathway	8/182	42/749	0,842043833	0,999954951	0,999954951	FCER1G/PSMB8/PSME2/PSMB10/FCGR1A/FCGR2A/PSME1/NFKB1	8
GO:0045582	positive regulation of T cell differentiation	8/182	42/749	0,842043833	0,999954951	0,999954951	BCL6/IL4R/NFKBIZ/TGFB1/SOCS1/XBP1/LILRB2/GATA3	8
GO:0045766	positive regulation of angiogenesis	8/182	42/749	0,842043833	0,999954951	0,999954951	HIF1A/JAK1/STAT3/C3/XBP1/ITGB2/C5AR1/SERPINE1	8
GO:0019953	sexual reproduction	8/182	43/749	0,861320404	0,999954951	0,999954951	BCL6/STAT3/ADAMTS1/IL4R/TGFB1/BAX/BIRC3/RGN	8
GO:0071887	leukocyte apoptotic process	8/182	44/749	0,87867053	0,999954951	0,999954951	HIF1A/BCL6/FCER1G/JAK3/BAX/KITLG/IRF7/LILRB1	8
GO:0006886	intracellular protein transport	8/182	45/749	0,894210816	0,999954951	0,999954951	STAT3/CD24/HDAC6/TGFB1/BAX/CDKN1A/AGT/ITGB2	8
GO:0045621	positive regulation of lymphocyte differentiation	8/182	47/749	0,920361916	0,999954951	0,999954951	BCL6/IL4R/NFKBIZ/TGFB1/SOCS1/XBP1/LILRB2/GATA3	8
GO:0060249	anatomical structure homeostasis	8/182	47/749	0,920361916	0,999954951	0,999954951	SERPINA3/HIF1A/LTF/TGFB1/BAX/TLR4/MYC/NOX4	8
GO:0010631	epithelial cell migration	8/182	49/749	0,940793815	0,999954951	0,999954951	HIF1A/HDAC6/NR4A1/TGFB1/AGT/APOE/ITGB2/GATA3	8
GO:0090132	epithelium migration	8/182	49/749	0,940793815	0,999954951	0,999954951	HIF1A/HDAC6/NR4A1/TGFB1/AGT/APOE/ITGB2/GATA3	8
GO:0044089	positive regulation of cellular component biogenesis	8/182	50/749	0,94917883	0,999954951	0,999954951	HDAC6/ICAM1/TGFB1/DNMT1/BAX/AGT/TLR4/NOX4	8
GO:0090130	tissue migration	8/182	50/749	0,94917883	0,999954951	0,999954951	HIF1A/HDAC6/NR4A1/TGFB1/AGT/APOE/ITGB2/GATA3	8
GO:0033365	protein localization to organelle	8/182	51/749	0,956501805	0,999954951	0,999954951	BCL3/STAT3/GBP2/COL1A1/TGFB1/BAX/CDKN1A/AGT	8
GO:0042102	positive regulation of T cell proliferation	8/182	61/749	0,992047556	0,999954951	0,999954951	CD24/JAK3/VCAM1/CD276/LILRB2/IL6R/EPO/IL6ST	8
GO:0007229	integrin-mediated signaling pathway	7/182	15/749	0,047517878	0,999954951	0,999954951	TIMP1/ITGB6/ADAMTS1/FCER1G/COL3A1/ITGB2/ISG15	7
GO:0009620	response to fungus	7/182	15/749	0,047517878	0,999954951	0,999954951	S100A9/LTF/S100A8/TGFB1/IL17RA/TLR4/S100A12	7
GO:0042445	hormone metabolic process	7/182	15/749	0,047517878	0,999954951	0,999954951	HIF1A/MME/HSD11B1/EGR1/TIPARP/GATA3/NFKB1	7
GO:0090090	negative regulation of canonical Wnt signaling pathway	7/182	15/749	0,047517878	0,999954951	0,999954951	SOST/PSMB8/PSME2/PSMB10/EGR1/APOE/PSME1	7
GO:0030178	negative regulation of Wnt signaling pathway	7/182	16/749	0,067566096	0,999954951	0,999954951	SOST/PSMB8/PSME2/PSMB10/EGR1/APOE/PSME1	7
GO:0060760	positive regulation of response to cytokine stimulus	7/182	16/749	0,067566096	0,999954951	0,999954951	HIF1A/IL1R1/TLR4/TLR2/IRF7/TBK1/CASP4	7
GO:1901988	negative regulation of cell cycle phase transition	7/182	16/749	0,067566096	0,999954951	0,999954951	PSMB8/MUC1/PSME2/PSMB10/BAX/CDKN1A/PSME1	7
GO:1901991	negative regulation of mitotic cell cycle phase transition	7/182	16/749	0,067566096	0,999954951	0,999954951	PSMB8/MUC1/PSME2/PSMB10/BAX/CDKN1A/PSME1	7
GO:0061326	renal tubule development	7/182	17/749	0,091984989	0,999954951	0,999954951	CD24/COL4A1/STAT1/TGFB1/AGT/MYC/GATA3	7
GO:0030177	positive regulation of Wnt signaling pathway	7/182	18/749	0,120701146	0,999954951	0,999954951	PSMB8/COL1A1/PSME2/PSMB10/TLR2/PSME1/NFKB1	7
GO:0030193	regulation of blood coagulation	7/182	18/749	0,120701146	0,999954951	0,999954951	SERPING1/FCER1G/TLR4/APOE/SERPINE1/THBD/PLAUR	7
GO:0034121	regulation of toll-like receptor signaling pathway	7/182	18/749	0,120701146	0,999954951	0,999954951	LTF/LY96/TLR4/TLR2/IRF7/BIRC3/CD14	7
GO:0043200	response to amino acid	7/182	18/749	0,120701146	0,999954951	0,999954951	COL4A1/COL1A1/COL3A1/ICAM1/SOCS1/XBP1/DNMT1	7

GO:0062207	regulation of pattern recognition receptor signaling pathway	7/182	18/749	0,120701146	0,999954951	0,999954951	LTF/LY96/TLR4/TLR2/IRF7/BIRC3/CD14	7
GO:1900046	regulation of hemostasis	7/182	18/749	0,120701146	0,999954951	0,999954951	SERPING1/FCER1G/TLR4/APOE/SERPINE1/THBD/PLAUR	7
GO:1902806	regulation of cell cycle G1/S phase transition	7/182	18/749	0,120701146	0,999954951	0,999954951	ADAMTS1/MUC1/PSME2/BAX/CDKN1A/PSME1/PTPN6	7
GO:2000045	regulation of G1/S transition of mitotic cell cycle	7/182	18/749	0,120701146	0,999954951	0,999954951	ADAMTS1/MUC1/PSME2/BAX/CDKN1A/PSME1/PTPN6	7
GO:0009743	response to carbohydrate	7/182	19/749	0,153463492	0,999954951	0,999954951	HIF1A/MAPK13/ICAM1/TGFB1/XBP1/EGR1/NOX4	7
GO:0046486	glycerolipid metabolic process	7/182	19/749	0,153463492	0,999954951	0,999954951	PLAAT4/C3/SOCS3/SOCS1/LDLR/APOE/RGN	7
GO:0046890	regulation of lipid biosynthetic process	7/182	19/749	0,153463492	0,999954951	0,999954951	C3/TNFRSF1A/LDLR/EGR1/APOE/NFKB1/RGN	7
GO:0050818	regulation of coagulation	7/182	19/749	0,153463492	0,999954951	0,999954951	SERPING1/FCER1G/TLR4/APOE/SERPINE1/THBD/PLAUR	7
GO:0051341	regulation of oxidoreductase activity	7/182	19/749	0,153463492	0,999954951	0,999954951	HIF1A/HDAC6/AGT/SLAMF8/APOE/NFKB1/RGN	7
GO:0002479	antigen processing and presentation of exogenous peptide antig	7/182	20/749	0,189868017	0,999954951	0,999954951	PSMB8/PSME2/PSMB10/FCGR1A/TAP2/PSME1/TAP1	7
GO:0002576	platelet degranulation	7/182	20/749	0,189868017	0,999954951	0,999954951	SERPINA3/TIMP1/SERPING1/FCER1G/TGFB1/FN1/SERPINE1	7
GO:0046578	regulation of Ras protein signal transduction	7/182	20/749	0,189868017	0,999954951	0,999954951	BCL6/COL3A1/KITLG/APOE/ARHGDIB/FGD2/EPO	7
GO:1903169	regulation of calcium ion transmembrane transport	7/182	20/749	0,189868017	0,999954951	0,999954951	EHD3/TGFB1/BAX/FKBP1A/G6PD/RGN/PTPN6	7
GO:0006066	alcohol metabolic process	7/182	21/749	0,229390076	0,999954951	0,999954951	ALDH3A2/APOL1/LDLR/APOE/APOL2/G6PD/NFKB1	7
GO:0007219	Notch signaling pathway	7/182	22/749	0,271420266	0,999954951	0,999954951	BCL6/STAT3/STAT1/TGFB1/MYC/KRT19/IL6ST	7
GO:0034767	positive regulation of ion transmembrane transport	7/182	22/749	0,271420266	0,999954951	0,999954951	EHD3/ABCB1/BAX/AGT/CTSS/G6PD/RGN	7
GO:0009416	response to light stimulus	7/182	23/749	0,315300478	0,999954951	0,999954951	HIF1A/BCL3/MAPK13/MME/BAX/CDKN1A/MYC	7
GO:0031669	cellular response to nutrient levels	7/182	23/749	0,315300478	0,999954951	0,999954951	LCN2/COL1A1/ICAM1/XBP1/CDKN1A/TNC/ATF3	7
GO:0051056	regulation of small GTPase mediated signal transduction	7/182	23/749	0,315300478	0,999954951	0,999954951	BCL6/COL3A1/KITLG/APOE/ARHGDIB/FGD2/EPO	7
GO:0090257	regulation of muscle system process	7/182	24/749	0,360357513	0,999954951	0,999954951	TNFRSF1A/EHD3/AGT/TNFRSF1B/ERRFI1/G6PD/IL6ST	7
GO:0043409	negative regulation of MAPK cascade	7/182	25/749	0,405932445	0,999954951	0,999954951	TLR4/APOE/MYC/ERRFI1/ATF3/RNF149/PTPN6	7
GO:0043588	skin development	7/182	25/749	0,405932445	0,999954951	0,999954951	PLAAT4/KRT8/COL1A1/COL3A1/KRT19/ERRFI1/LTB	7
GO:2000107	negative regulation of leukocyte apoptotic process	7/182	25/749	0,405932445	0,999954951	0,999954951	HIF1A/BCL6/FCER1G/JAK3/KITLG/IRF7/LILRB1	7
GO:0007611	learning or memory	7/182	26/749	0,451404671	0,999954951	0,999954951	HIF1A/MME/AGT/LDLR/APOE/TLR2/LILRB2	7
GO:0015711	organic anion transport	7/182	26/749	0,451404671	0,999954951	0,999954951	ABCB1/AGT/LDLR/APOE/SLC11A1/ABCC2/ADORA2A	7
GO:0061900	glial cell activation	7/182	26/749	0,451404671	0,999954951	0,999954951	C1QA/LDLR/TLR2/ITGB2/C5AR1/IFNGR1/ADORA2A	7
GO:0001659	temperature homeostasis	7/182	27/749	0,496210222	0,999954951	0,999954951	STAT3/LCN2/IL4R/STAT6/EGR1/TLR4/CMKLR1	7
GO:0007584	response to nutrient	7/182	27/749	0,496210222	0,999954951	0,999954951	COL1A1/STAT1/TGFB1/XBP1/VCAM1/TNC/EPO	7
GO:0030856	regulation of epithelial cell differentiation	7/182	27/749	0,496210222	0,999954951	0,999954951	PLAAT4/CD24/TNFRSF1A/STAT1/ERRFI1/SERPINE1/GATA3	7
GO:0071383	cellular response to steroid hormone stimulus	7/182	27/749	0,496210222	0,999954951	0,999954951	HDAC6/NR4A1/ICAM1/TGFB1/RORC/ABCC2/ERRFI1	7
GO:0001649	osteoblast differentiation	7/182	28/749	0,539854411	0,999954951	0,999954951	LTF/IFITM1/COL1A1/TNC/VCAN/IL6R/IL6ST	7
GO:0001776	leukocyte homeostasis	7/182	28/749	0,539854411	0,999954951	0,999954951	HIF1A/FCER1G/JAK3/TGFB1/BAX/KITLG/TNFSF14	7
GO:0032677	regulation of interleukin-8 production	7/182	28/749	0,539854411	0,999954951	0,999954951	BCL3/TLR4/MYD88/TLR2/CD58/CD14/SERPINE1	7
GO:0034504	protein localization to nucleus	7/182	28/749	0,539854411	0,999954951	0,999954951	BCL3/STAT3/GBP2/COL1A1/TGFB1/CDKN1A/AGT	7
GO:0030168	platelet activation	7/182	29/749	0,581919243	0,999954951	0,999954951	FCER1G/COL1A1/COL3A1/TLR4/APOE/THBD/PTPN6	7
GO:0090066	regulation of anatomical structure size	7/182	29/749	0,581919243	0,999954951	0,999954951	SOD2/ICAM1/AGT/SLC12A3/APOE/FN1/ADORA2A	7
GO:0008406	gonad development	7/182	30/749	0,622066263	0,999954951	0,999954951	ADAMTS1/ICAM1/BAX/KITLG/TNFSF10/TIPARP/GATA3	7
GO:0032637	interleukin-8 production	7/182	30/749	0,622066263	0,999954951	0,999954951	BCL3/TLR4/MYD88/TLR2/CD58/CD14/SERPINE1	7
GO:0042133	neurotransmitter metabolic process	7/182	30/749	0,622066263	0,999954951	0,999954951	ICAM1/AGT/TLR4/TLR2/ITGB2/SLC22A2/RGN	7
GO:0045137	development of primary sexual characteristics	7/182	30/749	0,622066263	0,999954951	0,999954951	ADAMTS1/ICAM1/BAX/KITLG/TNFSF10/TIPARP/GATA3	7
GO:0070588	calcium ion transmembrane transport	7/182	30/749	0,622066263	0,999954951	0,999954951	EHD3/TGFB1/BAX/FKBP1A/G6PD/RGN/PTPN6	7
GO:0032613	interleukin-10 production	7/182	31/749	0,660035629	0,999954951	0,999954951	BCL3/FCER1G/JAK3/TLR4/TLR2/LILRB1/ISG15	7
GO:0045639	positive regulation of myeloid cell differentiation	7/182	31/749	0,660035629	0,999954951	0,999954951	HIF1A/STAT3/STAT1/TGFB1/KITLG/TRIB1/ISG15	7
GO:1903409	reactive oxygen species biosynthetic process	7/182	31/749	0,660035629	0,999954951	0,999954951	STAT3/ICAM1/AGT/TLR4/ITGB2/NOX4/RGN	7
GO:0048638	regulation of developmental growth	7/182	33/749	0,728769608	0,999954951	0,999954951	STAT3/CDKN1A/APOE/MAPK11/G6PD/FN1/GDF15	7
GO:0048738	cardiac muscle tissue development	7/182	33/749	0,728769608	0,999954951	0,999954951	TGFB1/AGT/FKBP1A/MAPK11/NOX4/G6PD/MYOM2	7
GO:0051260	protein homooligomerization	7/182	33/749	0,728769608	0,999954951	0,999954951	SOD2/C9/FCER1G/EHD3/LCN2/BAX/APOE	7
GO:0031960	response to corticosteroid	7/182	34/749	0,759362575	0,999954951	0,999954951	COL1A1/ICAM1/TGFB1/CDKN1A/ABCC2/ERRFI1/EPO	7
GO:0042509	regulation of tyrosine phosphorylation of STAT protein	7/182	34/749	0,759362575	0,999954951	0,999954951	STAT3/SOCS3/TNFRSF1A/SOCS1/IL6R/EPO/IL6ST	7
GO:0043542	endothelial cell migration	7/182	35/749	0,787418862	0,999954951	0,999954951	HIF1A/NR4A1/TGFB1/AGT/APOE/ITGB2/GATA3	7
GO:0006325	chromatin organization	7/182	36/749	0,812980625	0,999954951	0,999954951	BCL6/MUC1/HDAC6/TGFB1/DNMT1/MYC/GATA3	7
GO:0007268	chemical synaptic transmission	7/182	36/749	0,812980625	0,999954951	0,999954951	STAT3/MME/AGT/APOE/LILRB2/SLC22A2/ADORA2A	7
GO:0045664	regulation of neuron differentiation	7/182	36/749	0,812980625	0,999954951	0,999954951	S100A9/BCL6/AGT/APOE/FN1/EPO/GATA3	7
GO:0051493	regulation of cytoskeleton organization	7/182	36/749	0,812980625	0,999954951	0,999954951	S100A9/S100A8/HDAC6/ICAM1/TGFB1/ARHGDIB/NOX4	7
GO:0098916	anterograde trans-synaptic signaling	7/182	36/749	0,812980625	0,999954951	0,999954951	STAT3/MME/AGT/APOE/LILRB2/SLC22A2/ADORA2A	7
GO:0007548	sex differentiation	7/182	37/749	0,83612603	0,999954951	0,999954951	ADAMTS1/ICAM1/BAX/KITLG/TNFSF10/TIPARP/GATA3	7
GO:0042108	positive regulation of cytokine biosynthetic process	7/182	37/749	0,83612603	0,999954951	0,999954951	BCL3/STAT3/CD276/EGR1/TLR4/TBK1/LTB	7
GO:0043254	regulation of protein-containing complex assembly	7/182	37/749	0,83612603	0,999954951	0,999954951	SOST/HDAC6/ICAM1/TGFB1/BAX/TLR4/APOE	7
GO:0099537	trans-synaptic signaling	7/182	37/749	0,83612603	0,999954951	0,999954951	STAT3/MME/AGT/APOE/LILRB2/SLC22A2/ADORA2A	7

GO:0006914	autophagy	7/182	38/749	0,856961299	0,999954951	0,999954951	S100A9/HIF1A/STAT3/S100A8/HDAC6/XBP1/TBK1	7
GO:0061919	process utilizing autophagic mechanism	7/182	38/749	0,856961299	0,999954951	0,999954951	S100A9/HIF1A/STAT3/S100A8/HDAC6/XBP1/TBK1	7
GO:0071674	mononuclear cell migration	7/182	38/749	0,856961299	0,999954951	0,999954951	TGFB1/SLAMF8/IL6R/S100A12/CMKLR1/C5AR1/SERPINE1	7
GO:0099536	synaptic signaling	7/182	38/749	0,856961299	0,999954951	0,999954951	STAT3/MME/AGT/APOE/LILRB2/SLC22A2/ADORA2A	7
GO:0060485	mesenchyme development	7/182	39/749	0,875613407	0,999954951	0,999954951	HIF1A/COL1A1/STAT1/TGFB1/KITLG/MYC/FN1	7
GO:0120035	regulation of plasma membrane bounded cell projection organiz	7/182	39/749	0,875613407	0,999954951	0,999954951	S100A9/ICAM1/AGT/APOE/FN1/EPO/GATA3	7
GO:0007276	gamete generation	7/182	40/749	0,892223575	0,999954951	0,999954951	BCL6/ADAMTS1/IL4R/TGFB1/BAX/BIRC3/RGN	7
GO:0032729	positive regulation of interferon-gamma production	7/182	40/749	0,892223575	0,999954951	0,999954951	BCL3/IL1R1/CD276/TLR4/IL6R/SLC11A1/CD14	7
GO:0060562	epithelial tube morphogenesis	7/182	40/749	0,892223575	0,999954951	0,999954951	HIF1A/COL4A1/TGFB1/AGT/TNC/MYC/GATA3	7
GO:0030593	neutrophil chemotaxis	7/182	41/749	0,90694162	0,999954951	0,999954951	S100A9/S100A8/FCER1G/CXCL1/S100A12/ITGB2/C5AR1	7
GO:0050679	positive regulation of epithelial cell proliferation	7/182	42/749	0,919921177	0,999954951	0,999954951	HIF1A/STAT3/NR4A1/TGFB1/XBP1/MYC/C5AR1	7
GO:0051276	chromosome organization	7/182	42/749	0,919921177	0,999954951	0,999954951	BCL6/MUC1/HDAC6/TGFB1/DNMT1/MYC/GATA3	7
GO:0048568	embryonic organ development	7/182	45/749	0,949945526	0,999954951	0,999954951	HIF1A/SOCS3/KRT8/TGFB1/KITLG/KRT19/GATA3	7
GO:0048598	embryonic morphogenesis	7/182	48/749	0,969542439	0,999954951	0,999954951	HIF1A/SOCS3/TGFB1/BAX/ITGB2/FN1/GATA3	7
GO:0002456	T cell mediated immunity	7/182	50/749	0,978428883	0,999954951	0,999954951	IL1R1/ICAM1/SLC11A1/PVR/TNFRSF1B/LILRB1/GATA3	7
GO:0002708	positive regulation of lymphocyte mediated immunity	7/182	51/749	0,981917282	0,999954951	0,999954951	C3/FCER1G/IL1R1/TGFB1/STAT6/PVR/GATA3	7
GO:0070374	positive regulation of ERK1 and ERK2 cascade	7/182	54/749	0,989504502	0,999954951	0,999954951	ICAM1/TGFB1/TLR4/APOE/NOX4/C5AR1/EPO	7
GO:0050920	regulation of chemotaxis	7/182	55/749	0,991286433	0,999954951	0,999954951	TGFB1/SLAMF8/IL6R/CMKLR1/C5AR1/SERPINE1/TNFSF14	7
GO:0009792	embryo development ending in birth or egg hatching	7/182	58/749	0,995080031	0,999954951	0,999954951	HIF1A/SOCS3/KRT8/COL1A1/TGFB1/KRT19/GATA3	7
GO:0043009	chordate embryonic development	7/182	58/749	0,995080031	0,999954951	0,999954951	HIF1A/SOCS3/KRT8/COL1A1/TGFB1/KRT19/GATA3	7
GO:0010332	response to gamma radiation	6/182	12/749	0,04655142	0,999954951	0,999954951	BAX/CDKN1A/EGR1/MYC/NOX4/GATA3	6
GO:0032608	interferon-beta production	6/182	12/749	0,04655142	0,999954951	0,999954951	RELB/TLR4/TLR2/IRF7/TBK1/LILRB1	6
GO:0032648	regulation of interferon-beta production	6/182	12/749	0,04655142	0,999954951	0,999954951	RELB/TLR4/TLR2/IRF7/TBK1/LILRB1	6
GO:0044106	cellular amine metabolic process	6/182	12/749	0,04655142	0,999954951	0,999954951	PSMB8/PSME2/PSMB10/ABCC2/ITGB2/PSME1	6
GO:0061013	regulation of mRNA catabolic process	6/182	12/749	0,04655142	0,999954951	0,999954951	PSMB8/PSME2/PSMB10/MYD88/SLC11A1/PSME1	6
GO:0098754	detoxification	6/182	12/749	0,04655142	0,999954951	0,999954951	S100A9/SOD2/MT1A/APOE/ABCC2/RGN	6
GO:1903311	regulation of mRNA metabolic process	6/182	12/749	0,04655142	0,999954951	0,999954951	PSMB8/PSME2/PSMB10/MYD88/SLC11A1/PSME1	6
GO:0002762	negative regulation of myeloid leukocyte differentiation	6/182	13/749	0,06941507	0,999954951	0,999954951	LTF/TLR4/MYC/TRIB1/LILRB1/TMEM178A	6
GO:0015893	drug transport	6/182	13/749	0,06941507	0,999954951	0,999954951	AGT/SLC11A1/MYC/ABCC2/SLC22A2/ADORA2A	6
GO:0043627	response to estrogen	6/182	13/749	0,06941507	0,999954951	0,999954951	CD24/IL4R/KRT19/ABCC2/EPO/GATA3	6
GO:0051186	cofactor metabolic process	6/182	13/749	0,06941507	0,999954951	0,999954951	STAT3/NNMT/HDAC6/G6PD/ALAS1/RGN	6
GO:0071230	cellular response to amino acid stimulus	6/182	13/749	0,06941507	0,999954951	0,999954951	COL4A1/COL1A1/COL3A1/SOCS1/XBP1/DNMT1	6
GO:0090263	positive regulation of canonical Wnt signaling pathway	6/182	13/749	0,06941507	0,999954951	0,999954951	PSMB8/COL1A1/PSME2/PSMB10/PSME1/NFKB1	6
GO:2000736	regulation of stem cell differentiation	6/182	13/749	0,06941507	0,999954951	0,999954951	STAT3/PSMB8/PSME2/PSMB10/PSME1/GATA3	6
GO:0002756	MyD88-independent toll-like receptor signaling pathway	6/182	14/749	0,097683986	0,999954951	0,999954951	LY96/TLR4/IRF7/TBK1/BIRC3/CD14	6
GO:0006801	superoxide metabolic process	6/182	14/749	0,097683986	0,999954951	0,999954951	SOD2/TGFB1/AGT/ITGB2/NOX4/RGN	6
GO:0006970	response to osmotic stress	6/182	14/749	0,097683986	0,999954951	0,999954951	MAPK13/ABCB1/RELB/BAX/ERRFI1/EPO	6
GO:0035666	TRIF-dependent toll-like receptor signaling pathway	6/182	14/749	0,097683986	0,999954951	0,999954951	LY96/TLR4/IRF7/TBK1/BIRC3/CD14	6
GO:0061045	negative regulation of wound healing	6/182	14/749	0,097683986	0,999954951	0,999954951	SERPING1/CDKN1A/APOE/SERPINE1/THBD/PLAUR	6
GO:1903035	negative regulation of response to wounding	6/182	14/749	0,097683986	0,999954951	0,999954951	SERPING1/CDKN1A/APOE/SERPINE1/THBD/PLAUR	6
GO:0002755	MyD88-dependent toll-like receptor signaling pathway	6/182	15/749	0,131128058	0,999954951	0,999954951	LY96/TLR4/MYD88/TLR2/IRF7/CD14	6
GO:0006402	mRNA catabolic process	6/182	15/749	0,131128058	0,999954951	0,999954951	PSMB8/PSME2/PSMB10/MYD88/SLC11A1/PSME1	6
GO:0010611	regulation of cardiac muscle hypertrophy	6/182	15/749	0,131128058	0,999954951	0,999954951	TNFRSF1A/AGT/TNFRSF1B/ERRFI1/G6PD/IL6ST	6
GO:0014743	regulation of muscle hypertrophy	6/182	15/749	0,131128058	0,999954951	0,999954951	TNFRSF1A/AGT/TNFRSF1B/ERRFI1/G6PD/IL6ST	6
GO:0019218	regulation of steroid metabolic process	6/182	15/749	0,131128058	0,999954951	0,999954951	AGT/LDLR/EGR1/APOE/RORC/NFKB1	6
GO:0033627	cell adhesion mediated by integrin	6/182	15/749	0,131128058	0,999954951	0,999954951	ITGB6/MUC1/ICAM1/ITGB2/SERPINE1/PTPN6	6
GO:0045598	regulation of fat cell differentiation	6/182	15/749	0,131128058	0,999954951	0,999954951	HDAC6/TGFB1/XBP1/RORC/CMKLR1/GATA3	6
GO:0045638	negative regulation of myeloid cell differentiation	6/182	15/749	0,131128058	0,999954951	0,999954951	LTF/TLR4/MYC/TRIB1/LILRB1/TMEM178A	6
GO:0009749	response to glucose	6/182	16/749	0,169265235	0,999954951	0,999954951	HIF1A/ICAM1/TGFB1/XBP1/EGR1/NOX4	6
GO:0032869	cellular response to insulin stimulus	6/182	16/749	0,169265235	0,999954951	0,999954951	SOCS3/STAT1/SOCS1/XBP1/AGT/ERRFI1	6
GO:0033500	carbohydrate homeostasis	6/182	16/749	0,169265235	0,999954951	0,999954951	HIF1A/STAT3/ICAM1/XBP1/HNF1A/NOX4	6
GO:0042593	glucose homeostasis	6/182	16/749	0,169265235	0,999954951	0,999954951	HIF1A/STAT3/ICAM1/XBP1/HNF1A/NOX4	6
GO:0072080	nephron tubule development	6/182	16/749	0,169265235	0,999954951	0,999954951	CD24/STAT1/TGFB1/AGT/MYC/GATA3	6
GO:2001243	negative regulation of intrinsic apoptotic signaling pathway	6/182	16/749	0,169265235	0,999954951	0,999954951	HIF1A/SOD2/MUC1/XBP1/EPO/PLAUR	6
GO:0008630	intrinsic apoptotic signaling pathway in response to DNA damag	6/182	17/749	0,211418097	0,999954951	0,999954951	BCL3/MUC1/TNFRSF1A/BAX/CDKN1A/TNFRSF1B	6
GO:0009746	response to hexose	6/182	17/749	0,211418097	0,999954951	0,999954951	HIF1A/ICAM1/TGFB1/XBP1/EGR1/NOX4	6
GO:0032412	regulation of ion transmembrane transporter activity	6/182	17/749	0,211418097	0,999954951	0,999954951	EHD3/ABCB1/FKBP1A/CTSS/EPO/RGN	6
GO:0034284	response to monosaccharide	6/182	17/749	0,211418097	0,999954951	0,999954951	HIF1A/ICAM1/TGFB1/XBP1/EGR1/NOX4	6
GO:0043502	regulation of muscle adaptation	6/182	17/749	0,211418097	0,999954951	0,999954951	TNFRSF1A/AGT/TNFRSF1B/ERRFI1/G6PD/IL6ST	6

GO:1901655	cellular response to ketone	6/182	17/749	0,211418097	0,999954951	0,999954951	ICAM1/TGFB1/AHR/TNC/ABCC2/ERRFI1	6
GO:0003300	cardiac muscle hypertrophy	6/182	18/749	0,256776573	0,999954951	0,999954951	TNFRSF1A/AGT/TNFRSF1B/ERRFI1/G6PD/IL6ST	6
GO:0006109	regulation of carbohydrate metabolic process	6/182	18/749	0,256776573	0,999954951	0,999954951	HIF1A/STAT3/TGFB1/RORC/NFKB1/RGN	6
GO:0014896	muscle hypertrophy	6/182	18/749	0,256776573	0,999954951	0,999954951	TNFRSF1A/AGT/TNFRSF1B/ERRFI1/G6PD/IL6ST	6
GO:0014897	striated muscle hypertrophy	6/182	18/749	0,256776573	0,999954951	0,999954951	TNFRSF1A/AGT/TNFRSF1B/ERRFI1/G6PD/IL6ST	6
GO:0022898	regulation of transmembrane transporter activity	6/182	18/749	0,256776573	0,999954951	0,999954951	EHD3/ABCB1/FKBP1A/CTSS/EPO/RGN	6
GO:0032409	regulation of transporter activity	6/182	18/749	0,256776573	0,999954951	0,999954951	EHD3/ABCB1/FKBP1A/CTSS/EPO/RGN	6
GO:0090596	sensory organ morphogenesis	6/182	18/749	0,256776573	0,999954951	0,999954951	HIF1A/STAT3/MEGF11/BAX/FJX1/GATA3	6
GO:1903362	regulation of cellular protein catabolic process	6/182	18/749	0,256776573	0,999954951	0,999954951	PSME2/LDLR/APOE/TRIB1/PSME1/RGN	6
GO:0007569	cell aging	6/182	19/749	0,304459533	0,999954951	0,999954951	BCL6/MME/ICAM1/CDKN1A/NOX4/SERPINE1	6
GO:0007623	circadian rhythm	6/182	19/749	0,304459533	0,999954951	0,999954951	RELB/AHR/EGR1/RORC/SERPINE1/ADORA2A	6
GO:0009411	response to UV	6/182	19/749	0,304459533	0,999954951	0,999954951	BCL3/MAPK13/MME/BAX/CDKN1A/MYC	6
GO:0010498	proteasomal protein catabolic process	6/182	19/749	0,304459533	0,999954951	0,999954951	PSMB8/PSME2/PSMB10/APOE/TRIB1/PSME1	6
GO:0014074	response to purine-containing compound	6/182	19/749	0,304459533	0,999954951	0,999954951	COL1A1/STAT1/AHR/NOX4/ADORA2A/THBD	6
GO:0016053	organic acid biosynthetic process	6/182	19/749	0,304459533	0,999954951	0,999954951	TGFB1/XBP1/ALOX5/VCAN/NFKB1/RGN	6
GO:0018205	peptidyl-lysine modification	6/182	19/749	0,304459533	0,999954951	0,999954951	MUC1/HDAC6/TGFB1/DNMT1/EGR1/GATA3	6
GO:0046394	carboxylic acid biosynthetic process	6/182	19/749	0,304459533	0,999954951	0,999954951	TGFB1/XBP1/ALOX5/VCAN/NFKB1/RGN	6
GO:0046636	negative regulation of alpha-beta T cell activation	6/182	19/749	0,304459533	0,999954951	0,999954951	BCL6/JAK3/IL4R/SOCS1/LILRB1/ADORA2A	6
GO:0006091	generation of precursor metabolites and energy	6/182	20/749	0,353570182	0,999954951	0,999954951	HIF1A/STAT3/MYC/NOX4/G6PD/IL6ST	6
GO:0006631	fatty acid metabolic process	6/182	20/749	0,353570182	0,999954951	0,999954951	ALDH3A2/C3/TNFRSF1A/XBP1/ALOX5/RGN	6
GO:0010565	regulation of cellular ketone metabolic process	6/182	20/749	0,353570182	0,999954951	0,999954951	PSMB8/PSME2/PSMB10/EGR1/PSME1/RGN	6
GO:0032757	positive regulation of interleukin-8 production	6/182	20/749	0,353570182	0,999954951	0,999954951	TLR4/MYD88/TLR2/CD58/CD14/SERPINE1	6
GO:0045778	positive regulation of ossification	6/182	20/749	0,353570182	0,999954951	0,999954951	LTF/IFITM1/TGFB1/IL6R/IL6ST/ISG15	6
GO:0048144	fibroblast proliferation	6/182	20/749	0,353570182	0,999954951	0,999954951	TGFB1/BAX/CDKN1A/AGT/MYC/FN1	6
GO:0048145	regulation of fibroblast proliferation	6/182	20/749	0,353570182	0,999954951	0,999954951	TGFB1/BAX/CDKN1A/AGT/MYC/FN1	6
GO:1904064	positive regulation of cation transmembrane transport	6/182	20/749	0,353570182	0,999954951	0,999954951	EHD3/BAX/AGT/CTSS/G6PD/RGN	6
GO:0003018	vascular process in circulatory system	6/182	21/749	0,403242225	0,999954951	0,999954951	SOD2/ICAM1/TGFB1/AGT/APOE/ADORA2A	6
GO:0008625	extrinsic apoptotic signaling pathway via death domain receptor	6/182	21/749	0,403242225	0,999954951	0,999954951	TNFRSF1A/ICAM1/BAX/TNFSF10/ATF3/SERPINE1	6
GO:0030879	mammary gland development	6/182	21/749	0,403242225	0,999954951	0,999954951	HIF1A/TGFB1/XBP1/BAX/STAT6/GATA3	6
GO:0033044	regulation of chromosome organization	6/182	21/749	0,403242225	0,999954951	0,999954951	BCL6/MUC1/TGFB1/DNMT1/MYC/GATA3	6
GO:0042180	cellular ketone metabolic process	6/182	21/749	0,403242225	0,999954951	0,999954951	PSMB8/PSME2/PSMB10/EGR1/PSME1/RGN	6
GO:1903556	negative regulation of tumor necrosis factor superfamily cytokin	6/182	21/749	0,403242225	0,999954951	0,999954951	LTF/BCL3/TLR4/ERRFI1/LILRB1/PTPN6	6
GO:0001774	microglial cell activation	6/182	22/749	0,452675459	0,999954951	0,999954951	C1QA/LDLR/TLR2/ITGB2/C5AR1/IFNGR1	6
GO:0002269	leukocyte activation involved in inflammatory response	6/182	22/749	0,452675459	0,999954951	0,999954951	C1QA/LDLR/TLR2/ITGB2/C5AR1/IFNGR1	6
GO:0006958	complement activation, classical pathway	6/182	22/749	0,452675459	0,999954951	0,999954951	C1QB/C1S/C3/SERPING1/C9/C1QA	6
GO:0043524	negative regulation of neuron apoptotic process	6/182	22/749	0,452675459	0,999954951	0,999954951	HIF1A/SOD2/BAX/APOE/C5AR1/ADORA2A	6
GO:0045732	positive regulation of protein catabolic process	6/182	22/749	0,452675459	0,999954951	0,999954951	LDLR/APOE/TNFRSF1B/TRIB1/TIPARP/RGN	6
GO:0051149	positive regulation of muscle cell differentiation	6/182	22/749	0,452675459	0,999954951	0,999954951	SOD2/IL4R/TGFB1/MAPK11/GDF15/TNFSF14	6
GO:0051604	protein maturation	6/182	22/749	0,452675459	0,999954951	0,999954951	MME/FKBP1A/CTSS/IL1R2/BIRC3/SERPINE1	6
GO:0072009	nephron epithelium development	6/182	22/749	0,452675459	0,999954951	0,999954951	CD24/STAT1/TGFB1/AGT/MYC/GATA3	6
GO:0032722	positive regulation of chemokine production	6/182	23/749	0,501160747	0,999954951	0,999954951	HIF1A/IL4R/EGR1/TLR4/TLR2/IL6R	6
GO:0043500	muscle adaptation	6/182	23/749	0,501160747	0,999954951	0,999954951	TNFRSF1A/AGT/TNFRSF1B/ERRFI1/G6PD/IL6ST	6
GO:0002312	B cell activation involved in immune response	6/182	24/749	0,548095158	0,999954951	0,999954951	BCL3/BCL6/TGFB1/XBP1/STAT6/TLR4	6
GO:0002712	regulation of B cell mediated immunity	6/182	24/749	0,548095158	0,999954951	0,999954951	BCL6/C3/FCER1G/TGFB1/STAT6/PTPN6	6
GO:0002889	regulation of immunoglobulin mediated immune response	6/182	24/749	0,548095158	0,999954951	0,999954951	BCL6/C3/FCER1G/TGFB1/STAT6/PTPN6	6
GO:0051961	negative regulation of nervous system development	6/182	24/749	0,548095158	0,999954951	0,999954951	STAT3/COL3A1/TGFB1/LDLR/APOE/TLR2	6
GO:0110110	positive regulation of animal organ morphogenesis	6/182	24/749	0,548095158	0,999954951	0,999954951	TGFB1/XBP1/BAX/AGT/MYC/GATA3	6
GO:0002832	negative regulation of response to biotic stimulus	6/182	25/749	0,592988647	0,999954951	0,999954951	LTF/SERPING1/SIGIRR/SLAMF8/TRIB1/LILRB1	6
GO:0043280	positive regulation of cysteine-type endopeptidase activity invol	6/182	25/749	0,592988647	0,999954951	0,999954951	S100A9/S100A8/BAX/MYC/TNFSF10/CASP4	6
GO:0051153	regulation of striated muscle cell differentiation	6/182	25/749	0,592988647	0,999954951	0,999954951	IL4R/TGFB1/XBP1/G6PD/GDF15/TNFSF14	6
GO:0016570	histone modification	6/182	26/749	0,63546384	0,999954951	0,999954951	BCL6/MUC1/HDAC6/TGFB1/DNMT1/GATA3	6
GO:0031099	regeneration	6/182	26/749	0,63546384	0,999954951	0,999954951	NNMT/TGFB1/CDKN1A/TNC/C5AR1/RGN	6
GO:0031346	positive regulation of cell projection organization	6/182	26/749	0,63546384	0,999954951	0,999954951	S100A9/LCN2/AGT/APOE/FN1/EPO	6
GO:0035051	cardiocyte differentiation	6/182	26/749	0,63546384	0,999954951	0,999954951	TGFB1/VCAM1/AGT/NOX4/G6PD/MYOM2	6
GO:0071236	cellular response to antibiotic	6/182	26/749	0,63546384	0,999954951	0,999954951	MAPK13/LCN2/HDAC6/AHR/STAT6/EGR1	6
GO:0038095	Fc-epsilon receptor signaling pathway	6/182	27/749	0,675250588	0,999954951	0,999954951	FCER1G/PSMB8/PSME2/PSMB10/PSME1/NFKB1	6
GO:0043370	regulation of CD4-positive, alpha-beta T cell differentiation	6/182	27/749	0,675250588	0,999954951	0,999954951	BCL6/JAK3/IL4R/NFKBIZ/SOCS1/GATA3	6
GO:0045666	positive regulation of neuron differentiation	6/182	27/749	0,675250588	0,999954951	0,999954951	S100A9/BCL6/AGT/APOE/FN1/EPO	6
GO:0045834	positive regulation of lipid metabolic process	6/182	27/749	0,675250588	0,999954951	0,999954951	TNFRSF1A/TGFB1/AGT/LDLR/APOE/RGN	6

GO:1903426	regulation of reactive oxygen species biosynthetic process	6/182	27/749	0,675250588	0,999954951	0,999954951	STAT3/ICAM1/AGT/TLR4/ITGB2/RGN	6
GO:2001056	positive regulation of cysteine-type endopeptidase activity	6/182	27/749	0,675250588	0,999954951	0,999954951	S100A9/S100A8/BAX/MYC/TNFSF10/CASP4	6
GO:0007600	sensory perception	6/182	28/749	0,712176818	0,999954951	0,999954951	MME/COL1A1/ICAM1/TGFBI/C5AR1/ADORA2A	6
GO:0010975	regulation of neuron projection development	6/182	28/749	0,712176818	0,999954951	0,999954951	S100A9/AGT/APOE/FN1/EPO/GATA3	6
GO:0016569	covalent chromatin modification	6/182	28/749	0,712176818	0,999954951	0,999954951	BCL6/MUC1/HDAC6/TGFB1/DNMT1/GATA3	6
GO:0030316	osteoclast differentiation	6/182	28/749	0,712176818	0,999954951	0,999954951	LTF/FCER1G/TGFB1/TLR4/LILRB1/TMEM178A	6
GO:0046209	nitric oxide metabolic process	6/182	28/749	0,712176818	0,999954951	0,999954951	ICAM1/AGT/TLR4/TLR2/ITGB2/RGN	6
GO:0051216	cartilage development	6/182	28/749	0,712176818	0,999954951	0,999954951	HIF1A/TIMP1/COL1A1/MAF/TGFB1/TGFBI	6
GO:0060348	bone development	6/182	28/749	0,712176818	0,999954951	0,999954951	LTF/COL1A1/TGFB1/LILRB1/RGN/PTPN6	6
GO:2001057	reactive nitrogen species metabolic process	6/182	28/749	0,712176818	0,999954951	0,999954951	ICAM1/AGT/TLR4/TLR2/ITGB2/RGN	6
GO:0032760	positive regulation of tumor necrosis factor production	6/182	29/749	0,746157055	0,999954951	0,999954951	FCER1G/LY96/TLR4/TLR2/CD14/IFNGR1	6
GO:0042136	neurotransmitter biosynthetic process	6/182	29/749	0,746157055	0,999954951	0,999954951	ICAM1/AGT/TLR4/ITGB2/SLC22A2/RGN	6
GO:0050804	modulation of chemical synaptic transmission	6/182	29/749	0,746157055	0,999954951	0,999954951	STAT3/MME/AGT/APOE/LILRB2/ADORA2A	6
GO:0051384	response to glucocorticoid	6/182	29/749	0,746157055	0,999954951	0,999954951	ICAM1/TGFB1/CDKN1A/ABCC2/ERRFI1/EPO	6
GO:0061138	morphogenesis of a branching epithelium	6/182	29/749	0,746157055	0,999954951	0,999954951	COL4A1/SOCS3/TGFB1/AGT/TNC/MYC	6
GO:0072073	kidney epithelium development	6/182	29/749	0,746157055	0,999954951	0,999954951	CD24/STAT1/TGFB1/AGT/MYC/GATA3	6
GO:0099177	regulation of trans-synaptic signaling	6/182	29/749	0,746157055	0,999954951	0,999954951	STAT3/MME/AGT/APOE/LILRB2/ADORA2A	6
GO:0032653	regulation of interleukin-10 production	6/182	30/749	0,777179753	0,999954951	0,999954951	BCL3/FCER1G/JAK3/TLR4/TLR2/LILRB1	6
GO:0042531	positive regulation of tyrosine phosphorylation of STAT protein	6/182	31/749	0,805294342	0,999954951	0,999954951	STAT3/SOCS3/TNFRSF1A/IL6R/EPO/IL6ST	6
GO:0042542	response to hydrogen peroxide	6/182	31/749	0,805294342	0,999954951	0,999954951	MAPK13/LCN2/HDAC6/COL1A1/STAT1/STAT6	6
GO:0046637	regulation of alpha-beta T cell differentiation	6/182	31/749	0,805294342	0,999954951	0,999954951	BCL6/JAK3/IL4R/NFKBIZ/SOCS1/GATA3	6
GO:1903557	positive regulation of tumor necrosis factor superfamily cytokine	6/182	31/749	0,805294342	0,999954951	0,999954951	FCER1G/LY96/TLR4/TLR2/CD14/IFNGR1	6
GO:0001763	morphogenesis of a branching structure	6/182	32/749	0,830598678	0,999954951	0,999954951	COL4A1/SOCS3/TGFB1/AGT/TNC/MYC	6
GO:0006518	peptide metabolic process	6/182	32/749	0,830598678	0,999954951	0,999954951	BCL3/STAT3/MME/APOE/G6PD/IFNGR1	6
GO:0022604	regulation of cell morphogenesis	6/182	32/749	0,830598678	0,999954951	0,999954951	ICAM1/APOE/LST1/FGD2/ITGB2/FN1	6
GO:0043087	regulation of GTPase activity	6/182	32/749	0,830598678	0,999954951	0,999954951	BCL6/ICAM1/ARHGDIB/FGD2/ERRFI1/RGN	6
GO:0048762	mesenchymal cell differentiation	6/182	32/749	0,830598678	0,999954951	0,999954951	HIF1A/COL1A1/STAT1/TGFB1/KITLG/FN1	6
GO:0001935	endothelial cell proliferation	6/182	35/749	0,891118286	0,999954951	0,999954951	HIF1A/STAT3/NR4A1/STAT1/XBP1/APOE	6
GO:0003007	heart morphogenesis	6/182	36/749	0,906745818	0,999954951	0,999954951	HIF1A/ADAMTS1/TGFB1/FKBP1A/MYOM2/GATA3	6
GO:0034614	cellular response to reactive oxygen species	6/182	36/749	0,906745818	0,999954951	0,999954951	SOD2/MAPK13/LCN2/HDAC6/STAT6/RGN	6
GO:1903531	negative regulation of secretion by cell	6/182	36/749	0,906745818	0,999954951	0,999954951	TNFRSF1A/APOE/TNFRSF1B/IL1R2/FN1/LILRB1	6
GO:2000514	regulation of CD4-positive, alpha-beta T cell activation	6/182	36/749	0,906745818	0,999954951	0,999954951	BCL6/JAK3/IL4R/NFKBIZ/SOCS1/GATA3	6
GO:0002688	regulation of leukocyte chemotaxis	6/182	37/749	0,920414164	0,999954951	0,999954951	SLAMF8/IL6R/CMKLR1/C5AR1/SERPINE1/TNFSF14	6
GO:0032990	cell part morphogenesis	6/182	37/749	0,920414164	0,999954951	0,999954951	HDAC6/BAX/APOE/FN1/GATA3/ADORA2A	6
GO:0051048	negative regulation of secretion	6/182	37/749	0,920414164	0,999954951	0,999954951	TNFRSF1A/APOE/TNFRSF1B/IL1R2/FN1/LILRB1	6
GO:2001236	regulation of extrinsic apoptotic signaling pathway	6/182	38/749	0,932311576	0,999954951	0,999954951	LTBR/ICAM1/AGT/TNFSF10/ATF3/SERPINE1	6
GO:0018105	peptidyl-serine phosphorylation	6/182	39/749	0,942620334	0,999954951	0,999954951	MAPK13/HDAC6/TGFB1/BAX/TBK1/EPO	6
GO:0018209	peptidyl-serine modification	6/182	39/749	0,942620334	0,999954951	0,999954951	MAPK13/HDAC6/TGFB1/BAX/TBK1/EPO	6
GO:0034622	cellular protein-containing complex assembly	6/182	40/749	0,95151388	0,999954951	0,999954951	HDAC6/ICAM1/TGFB1/FKBP1A/TLR4/MYOM2	6
GO:0010632	regulation of epithelial cell migration	6/182	43/749	0,971268595	0,999954951	0,999954951	HIF1A/HDAC6/TGFB1/AGT/APOE/GATA3	6
GO:0048771	tissue remodeling	6/182	43/749	0,971268595	0,999954951	0,999954951	HIF1A/TIMP1/TGFB1/BAX/AGT/NOX4	6
GO:0050871	positive regulation of B cell activation	6/182	43/749	0,971268595	0,999954951	0,999954951	BCL6/TGFB1/XBP1/CDKN1A/STAT6/TLR4	6
GO:0007178	transmembrane receptor protein serine/threonine kinase signal	6/182	45/749	0,980013363	0,999954951	0,999954951	SOST/COL3A1/TGFB1/EGR1/FKBP1A/GDF15	6
GO:0050921	positive regulation of chemotaxis	6/182	46/749	0,983395995	0,999954951	0,999954951	TGFB1/IL6R/CMKLR1/C5AR1/SERPINE1/TNFSF14	6
GO:0007420	brain development	6/182	51/749	0,993665522	0,999954951	0,999954951	HIF1A/COL4A1/COL3A1/BAX/C5AR1/G6PD	6
GO:0006879	cellular iron ion homeostasis	5/182	10/749	0,068878288	0,999954951	0,999954951	HIF1A/LTF/LCN2/SLC11A1/MYC	5
GO:0008203	cholesterol metabolic process	5/182	10/749	0,068878288	0,999954951	0,999954951	APOL1/LDLR/APOE/APOL2/G6PD	5
GO:0016125	sterol metabolic process	5/182	10/749	0,068878288	0,999954951	0,999954951	APOL1/LDLR/APOE/APOL2/G6PD	5
GO:0035456	response to interferon-beta	5/182	10/749	0,068878288	0,999954951	0,999954951	IFITM3/IFITM2/IFITM1/IFNAR2/STAT1	5
GO:0051926	negative regulation of calcium ion transport	5/182	10/749	0,068878288	0,999954951	0,999954951	ICAM1/TGFB1/LILRB2/LILRB1/EPO	5
GO:0055067	monovalent inorganic cation homeostasis	5/182	10/749	0,068878288	0,999954951	0,999954951	AGT/SLC12A3/SLAMF8/SLC11A1/ADORA2A	5
GO:0071322	cellular response to carbohydrate stimulus	5/182	10/749	0,068878288	0,999954951	0,999954951	HIF1A/MAPK13/ICAM1/XBP1/NOX4	5
GO:0071479	cellular response to ionizing radiation	5/182	10/749	0,068878288	0,999954951	0,999954951	TGFB1/CDKN1A/EGR1/NOX4/GATA3	5
GO:0071548	response to dexamethasone	5/182	10/749	0,068878288	0,999954951	0,999954951	ICAM1/TGFB1/ABCC2/ERRFI1/EPO	5
GO:0072111	cell proliferation involved in kidney development	5/182	10/749	0,068878288	0,999954951	0,999954951	STAT1/EGR1/IL6R/MYC/GATA3	5
GO:1902652	secondary alcohol metabolic process	5/182	10/749	0,068878288	0,999954951	0,999954951	APOL1/LDLR/APOE/APOL2/G6PD	5
GO:0030195	negative regulation of blood coagulation	5/182	11/749	0,102085117	0,999954951	0,999954951	SERPING1/APOE/SERPINE1/THBD/PLAUR	5
GO:0034644	cellular response to UV	5/182	11/749	0,102085117	0,999954951	0,999954951	MAPK13/MME/BAX/CDKN1A/MYC	5
GO:0035023	regulation of Rho protein signal transduction	5/182	11/749	0,102085117	0,999954951	0,999954951	BCL6/COL3A1/APOE/ARHGDIB/FGD2	5

GO:0050819	negative regulation of coagulation	5/182	11/749	0,102085117	0,999954951	0,999954951	SERPING1/APOE/SERPINE1/THBD/PLAUR	5
GO:0060218	hematopoietic stem cell differentiation	5/182	11/749	0,102085117	0,999954951	0,999954951	PSMB8/PSME2/PSMB10/PSME1/GATA3	5
GO:0061082	myeloid leukocyte cytokine production	5/182	11/749	0,102085117	0,999954951	0,999954951	BCL6/FCER1G/TGFB1/TLR4/LILRB1	5
GO:0061180	mammary gland epithelium development	5/182	11/749	0,102085117	0,999954951	0,999954951	HIF1A/TGFB1/BAX/STAT6/GATA3	5
GO:0071482	cellular response to light stimulus	5/182	11/749	0,102085117	0,999954951	0,999954951	MAPK13/MME/BAX/CDKN1A/MYC	5
GO:1900047	negative regulation of hemostasis	5/182	11/749	0,102085117	0,999954951	0,999954951	SERPING1/APOE/SERPINE1/THBD/PLAUR	5
GO:1900407	regulation of cellular response to oxidative stress	5/182	11/749	0,102085117	0,999954951	0,999954951	HIF1A/SOD2/HDAC6/TLR4/RGN	5
GO:1902882	regulation of response to oxidative stress	5/182	11/749	0,102085117	0,999954951	0,999954951	HIF1A/SOD2/HDAC6/TLR4/RGN	5
GO:0010575	positive regulation of vascular endothelial growth factor product	5/182	12/749	0,141752044	0,999954951	0,999954951	HIF1A/C3/TGFB1/C5AR1/IL6ST	5
GO:0016051	carbohydrate biosynthetic process	5/182	12/749	0,141752044	0,999954951	0,999954951	TGFB1/G6PD/ATF3/NFKB1/RGN	5
GO:0030258	lipid modification	5/182	12/749	0,141752044	0,999954951	0,999954951	ALDH3A2/SOCS3/SOCS1/AGT/APOE	5
GO:0048143	astrocyte activation	5/182	12/749	0,141752044	0,999954951	0,999954951	C1QA/LDLR/C5AR1/IFNGR1/ADORA2A	5
GO:0061333	renal tubule morphogenesis	5/182	12/749	0,141752044	0,999954951	0,999954951	COL4A1/TGFB1/AGT/MYC/GATA3	5
GO:0072088	nephron epithelium morphogenesis	5/182	12/749	0,141752044	0,999954951	0,999954951	STAT1/TGFB1/AGT/MYC/GATA3	5
GO:0006694	steroid biosynthetic process	5/182	13/749	0,186962653	0,999954951	0,999954951	HSD11B1/EGR1/APOE/G6PD/NFKB1	5
GO:0010573	vascular endothelial growth factor production	5/182	13/749	0,186962653	0,999954951	0,999954951	HIF1A/C3/TGFB1/C5AR1/IL6ST	5
GO:0010574	regulation of vascular endothelial growth factor production	5/182	13/749	0,186962653	0,999954951	0,999954951	HIF1A/C3/TGFB1/C5AR1/IL6ST	5
GO:0033619	membrane protein proteolysis	5/182	13/749	0,186962653	0,999954951	0,999954951	TIMP1/TGFB1/APOE/TNFRSF1B/NFKB1	5
GO:0045685	regulation of glial cell differentiation	5/182	13/749	0,186962653	0,999954951	0,999954951	TGFB1/LDLR/TLR2/TNFRSF1B/IL6ST	5
GO:0055072	iron ion homeostasis	5/182	13/749	0,186962653	0,999954951	0,999954951	HIF1A/LTF/LCN2/SLC11A1/MYC	5
GO:1903050	regulation of proteolysis involved in cellular protein catabolic pr	5/182	13/749	0,186962653	0,999954951	0,999954951	PSME2/APOE/TRIB1/PSME1/RGN	5
GO:1904705	regulation of vascular smooth muscle cell proliferation	5/182	13/749	0,186962653	0,999954951	0,999954951	SOD2/ADAMTS1/DNMT1/CDKN1A/AGT	5
GO:1990874	vascular smooth muscle cell proliferation	5/182	13/749	0,186962653	0,999954951	0,999954951	SOD2/ADAMTS1/DNMT1/CDKN1A/AGT	5
GO:2000378	negative regulation of reactive oxygen species metabolic proces	5/182	13/749	0,186962653	0,999954951	0,999954951	HIF1A/STAT3/HDAC6/G6PD/RGN	5
GO:0001961	positive regulation of cytokine-mediated signaling pathway	5/182	14/749	0,236561912	0,999954951	0,999954951	HIF1A/IL1R1/IRF7/TBK1/CASP4	5
GO:0006367	transcription initiation from RNA polymerase II promoter	5/182	14/749	0,236561912	0,999954951	0,999954951	NR4A1/BAX/CDKN1A/RORC/HNF1A	5
GO:0014910	regulation of smooth muscle cell migration	5/182	14/749	0,236561912	0,999954951	0,999954951	ADAMTS1/AGT/TRIB1/NOX4/SERPINE1	5
GO:0035150	regulation of tube size	5/182	14/749	0,236561912	0,999954951	0,999954951	SOD2/ICAM1/AGT/APOE/ADORA2A	5
GO:0035296	regulation of tube diameter	5/182	14/749	0,236561912	0,999954951	0,999954951	SOD2/ICAM1/AGT/APOE/ADORA2A	5
GO:0042036	negative regulation of cytokine biosynthetic process	5/182	14/749	0,236561912	0,999954951	0,999954951	BCL3/SIGIRR/ERRFI1/LILRB1/NFKB1	5
GO:0043271	negative regulation of ion transport	5/182	14/749	0,236561912	0,999954951	0,999954951	ICAM1/TGFB1/LILRB2/LILRB1/EPO	5
GO:0061097	regulation of protein tyrosine kinase activity	5/182	14/749	0,236561912	0,999954951	0,999954951	CD24/FCGR1A/AGT/ERRFI1/NOX4	5
GO:0070265	necrotic cell death	5/182	14/749	0,236561912	0,999954951	0,999954951	BAX/LY96/TLR4/BIRC3/CD14	5
GO:0070670	response to interleukin-4	5/182	14/749	0,236561912	0,999954951	0,999954951	JAK3/IL4R/XBP1/STAT6/GATA3	5
GO:0071353	cellular response to interleukin-4	5/182	14/749	0,236561912	0,999954951	0,999954951	JAK3/IL4R/XBP1/STAT6/GATA3	5
GO:0072028	nephron morphogenesis	5/182	14/749	0,236561912	0,999954951	0,999954951	STAT1/TGFB1/AGT/MYC/GATA3	5
GO:0097300	programmed necrotic cell death	5/182	14/749	0,236561912	0,999954951	0,999954951	BAX/LY96/TLR4/BIRC3/CD14	5
GO:0097746	regulation of blood vessel diameter	5/182	14/749	0,236561912	0,999954951	0,999954951	SOD2/ICAM1/AGT/APOE/ADORA2A	5
GO:0000209	protein polyubiquitination	5/182	15/749	0,289278838	0,999954951	0,999954951	PSMB8/HDAC6/PSME2/PSMB10/PSME1	5
GO:0014909	smooth muscle cell migration	5/182	15/749	0,289278838	0,999954951	0,999954951	ADAMTS1/AGT/TRIB1/NOX4/SERPINE1	5
GO:0031058	positive regulation of histone modification	5/182	15/749	0,289278838	0,999954951	0,999954951	BCL6/MUC1/TGFB1/DNMT1/GATA3	5
GO:0031100	animal organ regeneration	5/182	15/749	0,289278838	0,999954951	0,999954951	NNMT/TGFB1/CDKN1A/C5AR1/RGN	5
GO:0048146	positive regulation of fibroblast proliferation	5/182	15/749	0,289278838	0,999954951	0,999954951	TGFB1/CDKN1A/AGT/MYC/FN1	5
GO:0048167	regulation of synaptic plasticity	5/182	15/749	0,289278838	0,999954951	0,999954951	MME/AGT/APOE/LILRB2/ADORA2A	5
GO:0050680	negative regulation of epithelial cell proliferation	5/182	15/749	0,289278838	0,999954951	0,999954951	STAT1/TGFB1/APOE/GATA3/RGN	5
GO:0051591	response to cAMP	5/182	15/749	0,289278838	0,999954951	0,999954951	COL1A1/STAT1/AHR/NOX4/THBD	5
GO:0060993	kidney morphogenesis	5/182	15/749	0,289278838	0,999954951	0,999954951	STAT1/TGFB1/AGT/MYC/GATA3	5
GO:1905269	positive regulation of chromatin organization	5/182	15/749	0,289278838	0,999954951	0,999954951	BCL6/MUC1/TGFB1/DNMT1/GATA3	5
GO:2001252	positive regulation of chromosome organization	5/182	15/749	0,289278838	0,999954951	0,999954951	BCL6/MUC1/TGFB1/DNMT1/GATA3	5
GO:0006520	cellular amino acid metabolic process	5/182	16/749	0,343830476	0,999954951	0,999954951	PSMB8/PSME2/PSMB10/NOX4/PSME1	5
GO:0010822	positive regulation of mitochondrion organization	5/182	16/749	0,343830476	0,999954951	0,999954951	HIF1A/HDAC6/BAX/TNFSF10/PLAUR	5
GO:0014812	muscle cell migration	5/182	16/749	0,343830476	0,999954951	0,999954951	ADAMTS1/AGT/TRIB1/NOX4/SERPINE1	5
GO:0048592	eye morphogenesis	5/182	16/749	0,343830476	0,999954951	0,999954951	HIF1A/STAT3/MEGF11/BAX/FJX1	5
GO:0060041	retina development in camera-type eye	5/182	16/749	0,343830476	0,999954951	0,999954951	HIF1A/COL4A1/MEGF11/BAX/FJX1	5
GO:0007266	Rho protein signal transduction	5/182	17/749	0,399002301	0,999954951	0,999954951	BCL6/COL3A1/APOE/ARHGDIB/FGD2	5
GO:0019693	ribose phosphate metabolic process	5/182	17/749	0,399002301	0,999954951	0,999954951	HIF1A/STAT3/IMPDH1/TGFB1/G6PD	5
GO:0031330	negative regulation of cellular catabolic process	5/182	17/749	0,399002301	0,999954951	0,999954951	TIMP1/STAT3/MYD88/SLC11A1/RGN	5
GO:0043161	proteasome-mediated ubiquitin-dependent protein catabolic pr	5/182	17/749	0,399002301	0,999954951	0,999954951	PSMB8/PSME2/PSMB10/TRIB1/PSME1	5
GO:0050829	defense response to Gram-negative bacterium	5/182	17/749	0,399002301	0,999954951	0,999954951	LTF/TLR4/IL6R/SLC11A1/SERPINE1	5

GO:0120031	plasma membrane bounded cell projection assembly	5/182	17/749	0,399002301	0,999954951	0,999954951	EHD3/HDAC6/ICAM1/EMP3/FGD2	5
GO:0002260	lymphocyte homeostasis	5/182	18/749	0,453704504	0,999954951	0,999954951	HIF1A/JAK3/TGFB1/BAX/TNFSF14	5
GO:0031056	regulation of histone modification	5/182	18/749	0,453704504	0,999954951	0,999954951	BCL6/MUC1/TGFB1/DNMT1/GATA3	5
GO:0046683	response to organophosphorus	5/182	18/749	0,453704504	0,999954951	0,999954951	COL1A1/STAT1/AHR/NOX4/THBD	5
GO:0006352	DNA-templated transcription, initiation	5/182	19/749	0,50700628	0,999954951	0,999954951	NR4A1/BAX/CDKN1A/RORC/HNF1A	5
GO:0006919	activation of cysteine-type endopeptidase activity involved in ap	5/182	19/749	0,50700628	0,999954951	0,999954951	S100A9/S100A8/BAX/TNFSF10/CASP4	5
GO:0008584	male gonad development	5/182	19/749	0,50700628	0,999954951	0,999954951	ICAM1/BAX/KITLG/TNFSF10/GATA3	5
GO:0008585	female gonad development	5/182	19/749	0,50700628	0,999954951	0,999954951	ADAMTS1/ICAM1/BAX/KITLG/TIPARP	5
GO:0010522	regulation of calcium ion transport into cytosol	5/182	19/749	0,50700628	0,999954951	0,999954951	TGFB1/BAX/FKBP1A/EPO/PTPN6	5
GO:0010821	regulation of mitochondrion organization	5/182	19/749	0,50700628	0,999954951	0,999954951	HIF1A/HDAC6/BAX/TNFSF10/PLAUR	5
GO:0010976	positive regulation of neuron projection development	5/182	19/749	0,50700628	0,999954951	0,999954951	S100A9/AGT/APOE/FN1/EPO	5
GO:0017038	protein import	5/182	19/749	0,50700628	0,999954951	0,999954951	STAT3/TGFB1/CDKN1A/AGT/APOE	5
GO:0030308	negative regulation of cell growth	5/182	19/749	0,50700628	0,999954951	0,999954951	BCL6/TGFB1/CDKN1A/AGT/G6PD	5
GO:0032720	negative regulation of tumor necrosis factor production	5/182	19/749	0,50700628	0,999954951	0,999954951	BCL3/TLR4/ERRFI1/LILRB1/PTPN6	5
GO:0032963	collagen metabolic process	5/182	19/749	0,50700628	0,999954951	0,999954951	HIF1A/COL1A1/TGFB1/CTSS/ERRFI1	5
GO:0046545	development of primary female sexual characteristics	5/182	19/749	0,50700628	0,999954951	0,999954951	ADAMTS1/ICAM1/BAX/KITLG/TIPARP	5
GO:0046546	development of primary male sexual characteristics	5/182	19/749	0,50700628	0,999954951	0,999954951	ICAM1/BAX/KITLG/TNFSF10/GATA3	5
GO:0048588	developmental cell growth	5/182	19/749	0,50700628	0,999954951	0,999954951	HDAC6/AGT/APOE/G6PD/FN1	5
GO:0055007	cardiac muscle cell differentiation	5/182	19/749	0,50700628	0,999954951	0,999954951	TGFB1/AGT/NOX4/G6PD/MYOM2	5
GO:0071675	regulation of mononuclear cell migration	5/182	19/749	0,50700628	0,999954951	0,999954951	TGFB1/SLAMF8/CMKLR1/C5AR1/SERPINE1	5
GO:0071897	DNA biosynthetic process	5/182	19/749	0,50700628	0,999954951	0,999954951	CDKN1A/MYC/NOX4/ISG15/RGN	5
GO:0090068	positive regulation of cell cycle process	5/182	19/749	0,50700628	0,999954951	0,999954951	ADAMTS1/MUC1/TGFB1/BAX/CDKN1A	5
GO:1902275	regulation of chromatin organization	5/182	19/749	0,50700628	0,999954951	0,999954951	BCL6/MUC1/TGFB1/DNMT1/GATA3	5
GO:0008360	regulation of cell shape	5/182	20/749	0,558151645	0,999954951	0,999954951	ICAM1/LST1/FGD2/ITGB2/FN1	5
GO:0015849	organic acid transport	5/182	20/749	0,558151645	0,999954951	0,999954951	AGT/APOE/SLC11A1/ABCC2/ADORA2A	5
GO:0046942	carboxylic acid transport	5/182	20/749	0,558151645	0,999954951	0,999954951	AGT/APOE/SLC11A1/ABCC2/ADORA2A	5
GO:0050830	defense response to Gram-positive bacterium	5/182	20/749	0,558151645	0,999954951	0,999954951	GBP2/MYD88/TLR2/TBK1/C5AR1	5
GO:0055123	digestive system development	5/182	20/749	0,558151645	0,999954951	0,999954951	HIF1A/COL3A1/TGFB1/XBP1/CDKN1A	5
GO:0060560	developmental growth involved in morphogenesis	5/182	20/749	0,558151645	0,999954951	0,999954951	HDAC6/TGFB1/TNC/APOE/FN1	5
GO:0072331	signal transduction by p53 class mediator	5/182	20/749	0,558151645	0,999954951	0,999954951	BCL3/MUC1/BAX/CDKN1A/MAPK11	5
GO:0106106	cold-induced thermogenesis	5/182	20/749	0,558151645	0,999954951	0,999954951	LCN2/IL4R/STAT6/TLR4/CMKLR1	5
GO:0120161	regulation of cold-induced thermogenesis	5/182	20/749	0,558151645	0,999954951	0,999954951	LCN2/IL4R/STAT6/TLR4/CMKLR1	5
GO:1990845	adaptive thermogenesis	5/182	20/749	0,558151645	0,999954951	0,999954951	LCN2/IL4R/STAT6/TLR4/CMKLR1	5
GO:0001890	placenta development	5/182	21/749	0,606560839	0,999954951	0,999954951	HIF1A/SOCS3/MME/KRT8/KRT19	5
GO:0016485	protein processing	5/182	21/749	0,606560839	0,999954951	0,999954951	MME/CTSS/IL1R2/BIRC3/SERPINE1	5
GO:0030031	cell projection assembly	5/182	21/749	0,606560839	0,999954951	0,999954951	EHD3/HDAC6/ICAM1/EMP3/FGD2	5
GO:0034248	regulation of cellular amide metabolic process	5/182	21/749	0,606560839	0,999954951	0,999954951	BCL3/STAT3/TNFRSF1A/APOE/IFNGR1	5
GO:0097028	dendritic cell differentiation	5/182	21/749	0,606560839	0,999954951	0,999954951	LTBR/RELB/TGFB1/LILRB2/LILRB1	5
GO:0002823	negative regulation of adaptive immune response based on som	5/182	22/749	0,651821245	0,999954951	0,999954951	BCL6/JAK3/IL4R/LILRB1/PTPN6	5
GO:0002825	regulation of T-helper 1 type immune response	5/182	22/749	0,651821245	0,999954951	0,999954951	IL1R1/JAK3/IL4R/IL6R/SLC11A1	5
GO:0006753	nucleoside phosphate metabolic process	5/182	22/749	0,651821245	0,999954951	0,999954951	HIF1A/STAT3/NNMT/IMPDH1/TGFB1	5
GO:0009117	nucleotide metabolic process	5/182	22/749	0,651821245	0,999954951	0,999954951	HIF1A/STAT3/NNMT/IMPDH1/TGFB1	5
GO:0030100	regulation of endocytosis	5/182	22/749	0,651821245	0,999954951	0,999954951	C3/APOE/LILRB1/CD14/SERPINE1	5
GO:0046660	female sex differentiation	5/182	22/749	0,651821245	0,999954951	0,999954951	ADAMTS1/ICAM1/BAX/KITLG/TIPARP	5
GO:0090407	organophosphate biosynthetic process	5/182	22/749	0,651821245	0,999954951	0,999954951	STAT3/NNMT/IMPDH1/TGFB1/G6PD	5
GO:0006470	protein dephosphorylation	5/182	23/749	0,693671384	0,999954951	0,999954951	TGFB1/FKBP1A/LILRB2/RGN/PTPN6	5
GO:0042476	odontogenesis	5/182	23/749	0,693671384	0,999954951	0,999954951	COL1A1/TGFB1/BAX/TNC/SERPINE1	5
GO:0045428	regulation of nitric oxide biosynthetic process	5/182	23/749	0,693671384	0,999954951	0,999954951	ICAM1/AGT/TLR4/ITGB2/RGN	5
GO:0046661	male sex differentiation	5/182	23/749	0,693671384	0,999954951	0,999954951	ICAM1/BAX/KITLG/TNFSF10/GATA3	5
GO:0050764	regulation of phagocytosis	5/182	23/749	0,693671384	0,999954951	0,999954951	C3/FCER1G/TGFB1/TLR2/SLC11A1	5
GO:0050768	negative regulation of neurogenesis	5/182	23/749	0,693671384	0,999954951	0,999954951	STAT3/COL3A1/TGFB1/LDLR/APOE	5
GO:0051640	organelle localization	5/182	23/749	0,693671384	0,999954951	0,999954951	HIF1A/FCER1G/HDAC6/IL4R/CD59	5
GO:0055002	striated muscle cell development	5/182	23/749	0,693671384	0,999954951	0,999954951	KRT8/AGT/KRT19/G6PD/MYOM2	5
GO:2000117	negative regulation of cysteine-type endopeptidase activity	5/182	23/749	0,693671384	0,999954951	0,999954951	LTF/BIRC3/ADORA2A/TNFSF14/PLAUR	5
GO:0003206	cardiac chamber morphogenesis	5/182	24/749	0,731980917	0,999954951	0,999954951	HIF1A/ADAMTS1/TGFB1/FKBP1A/GATA3	5
GO:0007050	cell cycle arrest	5/182	24/749	0,731980917	0,999954951	0,999954951	MUC1/TGFB1/BAX/CDKN1A/MYC	5
GO:0008544	epidermis development	5/182	24/749	0,731980917	0,999954951	0,999954951	ALDH3A2/PLAAT4/KRT8/KRT19/ERRFI1	5
GO:0022612	gland morphogenesis	5/182	24/749	0,731980917	0,999954951	0,999954951	TGFB1/XBP1/BAX/STAT6/TNC	5
GO:0048754	branching morphogenesis of an epithelial tube	5/182	24/749	0,731980917	0,999954951	0,999954951	COL4A1/TGFB1/AGT/TNC/MYC	5

GO:0051054	positive regulation of DNA metabolic process	5/182	24/749	0,731980917	0,999954951	0,999954951	TGFB1/BAX/STAT6/MYC/NOX4	5
GO:0055086	nucleobase-containing small molecule metabolic process	5/182	24/749	0,731980917	0,999954951	0,999954951	HIF1A/STAT3/NNMT/IMPDH1/TGFB1	5
GO:0070925	organelle assembly	5/182	24/749	0,731980917	0,999954951	0,999954951	EHD3/KRT8/HDAC6/KRT19/MYOM2	5
GO:1901216	positive regulation of neuron death	5/182	24/749	0,731980917	0,999954951	0,999954951	C1QA/BAX/EGR1/TLR4/ITGB2	5
GO:0002720	positive regulation of cytokine production involved in immune re	5/182	25/749	0,766728984	0,999954951	0,999954951	FCER1G/IL1R1/TLR4/LILRB1/GATA3	5
GO:0002820	negative regulation of adaptive immune response	5/182	25/749	0,766728984	0,999954951	0,999954951	BCL6/JAK3/IL4R/LILRB1/PTPN6	5
GO:0003205	cardiac chamber development	5/182	25/749	0,766728984	0,999954951	0,999954951	HIF1A/ADAMTS1/TGFB1/FKBP1A/GATA3	5
GO:0009895	negative regulation of catabolic process	5/182	25/749	0,766728984	0,999954951	0,999954951	TIMP1/STAT3/MYD88/SLC11A1/RGN	5
GO:0014013	regulation of gliogenesis	5/182	25/749	0,766728984	0,999954951	0,999954951	TGFB1/LDLR/TLR2/TNFRSF1B/IL6ST	5
GO:0031214	biomineral tissue development	5/182	25/749	0,766728984	0,999954951	0,999954951	HIF1A/LTF/COL1A1/TGFB1/ISG15	5
GO:0035821	modulation of process of other organism	5/182	25/749	0,766728984	0,999954951	0,999954951	S100A9/LTF/SLPI/TGFB1/APOE	5
GO:0042092	type 2 immune response	5/182	25/749	0,766728984	0,999954951	0,999954951	BCL3/BCL6/IL4R/STAT6/GATA3	5
GO:0055001	muscle cell development	5/182	25/749	0,766728984	0,999954951	0,999954951	KRT8/AGT/KRT19/G6PD/MYOM2	5
GO:0110148	biomineralization	5/182	25/749	0,766728984	0,999954951	0,999954951	HIF1A/LTF/COL1A1/TGFB1/ISG15	5
GO:1902903	regulation of supramolecular fiber organization	5/182	25/749	0,766728984	0,999954951	0,999954951	HDAC6/ICAM1/LDLR/APOE/NOX4	5
GO:0000187	activation of MAPK activity	5/182	26/749	0,79798259	0,999954951	0,999954951	FPR1/TLR4/MAPK11/C5AR1/GDF15	5
GO:0002064	epithelial cell development	5/182	27/749	0,825876227	0,999954951	0,999954951	HIF1A/TNFRSF1A/ICAM1/XBP1/CDKN1A	5
GO:0006644	phospholipid metabolic process	5/182	27/749	0,825876227	0,999954951	0,999954951	PLAAT4/SOCS3/TGFB1/SOCS1/LDLR	5
GO:0006809	nitric oxide biosynthetic process	5/182	27/749	0,825876227	0,999954951	0,999954951	ICAM1/AGT/TLR4/ITGB2/RGN	5
GO:0045787	positive regulation of cell cycle	5/182	27/749	0,825876227	0,999954951	0,999954951	ADAMTS1/MUC1/TGFB1/BAX/CDKN1A	5
GO:0048661	positive regulation of smooth muscle cell proliferation	5/182	28/749	0,850593489	0,999954951	0,999954951	ADAMTS1/STAT1/DNMT1/AGT/IL6R	5
GO:0060401	cytosolic calcium ion transport	5/182	28/749	0,850593489	0,999954951	0,999954951	TGFB1/BAX/FKBP1A/EPO/PTPN6	5
GO:0060402	calcium ion transport into cytosol	5/182	28/749	0,850593489	0,999954951	0,999954951	TGFB1/BAX/FKBP1A/EPO/PTPN6	5
GO:0009914	hormone transport	5/182	29/749	0,87235108	0,999954951	0,999954951	HIF1A/AGT/ABCC2/HNF1A/GATA3	5
GO:0051099	positive regulation of binding	5/182	29/749	0,87235108	0,999954951	0,999954951	TGFB1/FKBP1A/APOE/GATA3/PLAUR	5
GO:1901222	regulation of NIK/NF-kappaB signaling	5/182	29/749	0,87235108	0,999954951	0,999954951	BCL3/TLR4/TLR2/CD14/TNFSF14	5
GO:0002637	regulation of immunoglobulin production	5/182	30/749	0,891385345	0,999954951	0,999954951	BCL6/IL4R/TGFB1/XBP1/STAT6	5
GO:0010506	regulation of autophagy	5/182	30/749	0,891385345	0,999954951	0,999954951	HIF1A/STAT3/HDAC6/XBP1/TBK1	5
GO:0010594	regulation of endothelial cell migration	5/182	30/749	0,891385345	0,999954951	0,999954951	HIF1A/TGFB1/AGT/APOE/GATA3	5
GO:0016311	dephosphorylation	5/182	30/749	0,891385345	0,999954951	0,999954951	TGFB1/FKBP1A/LILRB2/RGN/PTPN6	5
GO:0001936	regulation of endothelial cell proliferation	5/182	31/749	0,907941307	0,999954951	0,999954951	HIF1A/STAT3/NR4A1/STAT1/APOE	5
GO:0031334	positive regulation of protein-containing complex assembly	5/182	31/749	0,907941307	0,999954951	0,999954951	HDAC6/ICAM1/TGFB1/BAX/TLR4	5
GO:0032945	negative regulation of mononuclear cell proliferation	5/182	31/749	0,907941307	0,999954951	0,999954951	TGFB1/LILRB2/LST1/LILRB1/PTPN6	5
GO:0043393	regulation of protein binding	5/182	31/749	0,907941307	0,999954951	0,999954951	SLPI/BAX/FKBP1A/APOE/IFIT1	5
GO:0050672	negative regulation of lymphocyte proliferation	5/182	31/749	0,907941307	0,999954951	0,999954951	TGFB1/LILRB2/LST1/LILRB1/PTPN6	5
GO:0061564	axon development	5/182	31/749	0,907941307	0,999954951	0,999954951	HDAC6/TNC/APOE/FN1/GATA3	5
GO:0002690	positive regulation of leukocyte chemotaxis	5/182	32/749	0,922264017	0,999954951	0,999954951	IL6R/CMKLR1/C5AR1/SERPINE1/TNFSF14	5
GO:0070664	negative regulation of leukocyte proliferation	5/182	32/749	0,922264017	0,999954951	0,999954951	TGFB1/LILRB2/LST1/LILRB1/PTPN6	5
GO:0010634	positive regulation of epithelial cell migration	5/182	33/749	0,934591991	0,999954951	0,999954951	HIF1A/HDAC6/TGFB1/AGT/GATA3	5
GO:0048812	neuron projection morphogenesis	5/182	33/749	0,934591991	0,999954951	0,999954951	HDAC6/APOE/FN1/GATA3/ADORA2A	5
GO:0090287	regulation of cellular response to growth factor stimulus	5/182	34/749	0,945152465	0,999954951	0,999954951	HIF1A/SOST/TGFB1/AGT/GATA3	5
GO:0048858	cell projection morphogenesis	5/182	35/749	0,954158162	0,999954951	0,999954951	HDAC6/APOE/FN1/GATA3/ADORA2A	5
GO:0072594	establishment of protein localization to organelle	5/182	35/749	0,954158162	0,999954951	0,999954951	STAT3/TGFB1/BAX/CDKN1A/AGT	5
GO:0120039	plasma membrane bounded cell projection morphogenesis	5/182	35/749	0,954158162	0,999954951	0,999954951	HDAC6/APOE/FN1/GATA3/ADORA2A	5
GO:0002709	regulation of T cell mediated immunity	5/182	37/749	0,968272681	0,999954951	0,999954951	IL1R1/PVR/TNFRSF1B/LILRB1/GATA3	5
GO:0001894	tissue homeostasis	5/182	38/749	0,973721333	0,999954951	0,999954951	SERPINA3/LTF/BAX/TLR4/NOX4	5
GO:0001701	in utero embryonic development	5/182	39/749	0,978295009	0,999954951	0,999954951	HIF1A/SOCS3/KRT8/KRT19/GATA3	5
GO:0002377	immunoglobulin production	5/182	40/749	0,982120885	0,999954951	0,999954951	BCL6/IL4R/TGFB1/XBP1/STAT6	5
GO:0042100	B cell proliferation	5/182	40/749	0,982120885	0,999954951	0,999954951	BCL6/BAX/AHR/CDKN1A/TLR4	5
GO:0071559	response to transforming growth factor beta	5/182	40/749	0,982120885	0,999954951	0,999954951	COL1A1/COL3A1/TGFB1/NOX4/GDF15	5
GO:0071560	cellular response to transforming growth factor beta stimulus	5/182	40/749	0,982120885	0,999954951	0,999954951	COL1A1/COL3A1/TGFB1/NOX4/GDF15	5
GO:0045165	cell fate commitment	5/182	42/749	0,987961537	0,999954951	0,999954951	STAT3/STAT6/SLAMF8/RORC/GATA3	5
GO:0000904	cell morphogenesis involved in differentiation	5/182	44/749	0,991972548	0,999954951	0,999954951	HDAC6/APOE/FN1/GATA3/PTPN6	5
GO:0001909	leukocyte mediated cytotoxicity	5/182	45/749	0,993467446	0,999954951	0,999954951	ICAM1/PVR/LILRB1/KIR3DL1/PTPN6	5
GO:0051897	positive regulation of protein kinase B signaling	5/182	47/749	0,99570218	0,999954951	0,999954951	TGFB1/KITLG/NOX4/GDF15/GATA3	5
GO:0051896	regulation of protein kinase B signaling	5/182	50/749	0,997741682	0,999954951	0,999954951	TGFB1/KITLG/NOX4/GDF15/GATA3	5
GO:0043491	protein kinase B signaling	5/182	58/749	0,999625543	0,999954951	0,999954951	TGFB1/KITLG/NOX4/GDF15/GATA3	5
GO:0010927	cellular component assembly involved in morphogenesis	4/182	10/749	0,206742596	0,999954951	0,999954951	KRT8/TLR2/KRT19/MYOM2	4
GO:0032350	regulation of hormone metabolic process	4/182	10/749	0,206742596	0,999954951	0,999954951	HIF1A/EGR1/GATA3/NFKB1	4

GO:0032411	positive regulation of transporter activity	4/182	10/749	0,206742596	0,999954951	0,999954951	EHD3/ABCB1/CTSS/RGN	4
GO:0032414	positive regulation of ion transmembrane transporter activity	4/182	10/749	0,206742596	0,999954951	0,999954951	EHD3/ABCB1/CTSS/RGN	4
GO:0032480	negative regulation of type I interferon production	4/182	10/749	0,206742596	0,999954951	0,999954951	RELB/TBK1/LILRB1/ISG15	4
GO:0035567	non-canonical Wnt signaling pathway	4/182	10/749	0,206742596	0,999954951	0,999954951	PSMB8/PSME2/PSMB10/PSME1	4
GO:0036473	cell death in response to oxidative stress	4/182	10/749	0,206742596	0,999954951	0,999954951	HIF1A/SOD2/HDAC6/TLR4	4
GO:0042632	cholesterol homeostasis	4/182	10/749	0,206742596	0,999954951	0,999954951	CD24/XBP1/LDLR/APOE	4
GO:0045064	T-helper 2 cell differentiation	4/182	10/749	0,206742596	0,999954951	0,999954951	BCL3/BCL6/IL4R/GATA3	4
GO:0045671	negative regulation of osteoclast differentiation	4/182	10/749	0,206742596	0,999954951	0,999954951	LTF/TLR4/LILRB1/TMEM178A	4
GO:0045687	positive regulation of glial cell differentiation	4/182	10/749	0,206742596	0,999954951	0,999954951	TGFB1/TLR2/TNFRSF1B/IL6ST	4
GO:0051353	positive regulation of oxidoreductase activity	4/182	10/749	0,206742596	0,999954951	0,999954951	HIF1A/AGT/APOE/RGN	4
GO:0055092	sterol homeostasis	4/182	10/749	0,206742596	0,999954951	0,999954951	CD24/XBP1/LDLR/APOE	4
GO:0060338	regulation of type I interferon-mediated signaling pathway	4/182	10/749	0,206742596	0,999954951	0,999954951	IFNAR2/IRF7/TBK1/PTPN6	4
GO:0060675	ureteric bud morphogenesis	4/182	10/749	0,206742596	0,999954951	0,999954951	TGFB1/AGT/MYC/GATA3	4
GO:0070757	interleukin-35-mediated signaling pathway	4/182	10/749	0,206742596	0,999954951	0,999954951	JAK1/STAT3/STAT1/IL6ST	4
GO:0072078	nephron tubule morphogenesis	4/182	10/749	0,206742596	0,999954951	0,999954951	TGFB1/AGT/MYC/GATA3	4
GO:0072171	mesonephric tubule morphogenesis	4/182	10/749	0,206742596	0,999954951	0,999954951	TGFB1/AGT/MYC/GATA3	4
GO:0072332	intrinsic apoptotic signaling pathway by p53 class mediator	4/182	10/749	0,206742596	0,999954951	0,999954951	BCL3/MUC1/BAX/CDKN1A	4
GO:0090322	regulation of superoxide metabolic process	4/182	10/749	0,206742596	0,999954951	0,999954951	TGFB1/AGT/ITGB2/RGN	4
GO:1903510	mucopolysaccharide metabolic process	4/182	10/749	0,206742596	0,999954951	0,999954951	LYVE1/TGFB1/VCAN/NFKB1	4
GO:1905314	semi-lunar valve development	4/182	10/749	0,206742596	0,999954951	0,999954951	TNFRSF1A/TGFB1/TNFRSF1B/GATA3	4
GO:2001238	positive regulation of extrinsic apoptotic signaling pathway	4/182	10/749	0,206742596	0,999954951	0,999954951	LTBR/AGT/TNFSF10/ATF3	4
GO:0001678	cellular glucose homeostasis	4/182	11/749	0,26654687	0,999954951	0,999954951	HIF1A/ICAM1/XBP1/NOX4	4
GO:0002888	positive regulation of myeloid leukocyte mediated immunity	4/182	11/749	0,26654687	0,999954951	0,999954951	C3/FCER1G/IL4R/ITGB2	4
GO:0006836	neurotransmitter transport	4/182	11/749	0,26654687	0,999954951	0,999954951	FCER1G/LILRB1/SLC22A2/ADORA2A	4
GO:0007093	mitotic cell cycle checkpoint	4/182	11/749	0,26654687	0,999954951	0,999954951	MUC1/TGFB1/BAX/CDKN1A	4
GO:0030216	keratinocyte differentiation	4/182	11/749	0,26654687	0,999954951	0,999954951	PLAAT4/KRT8/KRT19/ERRFI1	4
GO:0032727	positive regulation of interferon-alpha production	4/182	11/749	0,26654687	0,999954951	0,999954951	STAT1/TLR4/IRF7/TBK1	4
GO:0035967	cellular response to topologically incorrect protein	4/182	11/749	0,26654687	0,999954951	0,999954951	HDAC6/XBP1/BAX/ATF3	4
GO:0042446	hormone biosynthetic process	4/182	11/749	0,26654687	0,999954951	0,999954951	HIF1A/HSD11B1/EGR1/NFKB1	4
GO:0045591	positive regulation of regulatory T cell differentiation	4/182	11/749	0,26654687	0,999954951	0,999954951	BCL6/TGFB1/SOCS1/LILRB2	4
GO:0046639	negative regulation of alpha-beta T cell differentiation	4/182	11/749	0,26654687	0,999954951	0,999954951	BCL6/JAK3/IL4R/SOCS1	4
GO:0055088	lipid homeostasis	4/182	11/749	0,26654687	0,999954951	0,999954951	CD24/XBP1/LDLR/APOE	4
GO:0010742	macrophage derived foam cell differentiation	4/182	12/749	0,328944283	0,999954951	0,999954951	STAT1/TGFB1/AGT/NFKB1	4
GO:0031664	regulation of lipopolysaccharide-mediated signaling pathway	4/182	12/749	0,328944283	0,999954951	0,999954951	LTF/SIGIRR/LY96/TRIB1	4
GO:0033032	regulation of myeloid cell apoptotic process	4/182	12/749	0,328944283	0,999954951	0,999954951	FCER1G/KITLG/IRF7/EPO	4
GO:0038111	interleukin-7-mediated signaling pathway	4/182	12/749	0,328944283	0,999954951	0,999954951	JAK1/STAT3/JAK3/SOCS1	4
GO:0043029	T cell homeostasis	4/182	12/749	0,328944283	0,999954951	0,999954951	JAK3/TGFB1/BAX/TNFSF14	4
GO:0044070	regulation of anion transport	4/182	12/749	0,328944283	0,999954951	0,999954951	ABCB1/AGT/APOE/ADORA2A	4
GO:0045063	T-helper 1 cell differentiation	4/182	12/749	0,328944283	0,999954951	0,999954951	RELB/JAK3/IL4R/STAT6	4
GO:0046889	positive regulation of lipid biosynthetic process	4/182	12/749	0,328944283	0,999954951	0,999954951	TNFRSF1A/LDLR/APOE/RGN	4
GO:0048593	camera-type eye morphogenesis	4/182	12/749	0,328944283	0,999954951	0,999954951	HIF1A/MEGF11/BAX/FJX1	4
GO:0051279	regulation of release of sequestered calcium ion into cytosol	4/182	12/749	0,328944283	0,999954951	0,999954951	TGFB1/BAX/FKBP1A/PTPN6	4
GO:0061136	regulation of proteasomal protein catabolic process	4/182	12/749	0,328944283	0,999954951	0,999954951	PSME2/APOE/TRIB1/PSME1	4
GO:0071385	cellular response to glucocorticoid stimulus	4/182	12/749	0,328944283	0,999954951	0,999954951	ICAM1/TGFB1/ABCC2/ERRFI1	4
GO:0090077	foam cell differentiation	4/182	12/749	0,328944283	0,999954951	0,999954951	STAT1/TGFB1/AGT/NFKB1	4
GO:0090303	positive regulation of wound healing	4/182	12/749	0,328944283	0,999954951	0,999954951	XBP1/TLR4/SERPINE1/THBD	4
GO:0098656	anion transmembrane transport	4/182	12/749	0,328944283	0,999954951	0,999954951	ABCB1/AGT/APOL1/SLC12A3	4
GO:1900015	regulation of cytokine production involved in inflammatory resp	4/182	12/749	0,328944283	0,999954951	0,999954951	IL17RA/TLR4/MYD88/IL1R2	4
GO:1903036	positive regulation of response to wounding	4/182	12/749	0,328944283	0,999954951	0,999954951	XBP1/TLR4/SERPINE1/THBD	4
GO:1904646	cellular response to amyloid-beta	4/182	12/749	0,328944283	0,999954951	0,999954951	ICAM1/VCAM1/TLR4/CASP4	4
GO:0000075	cell cycle checkpoint	4/182	13/749	0,39204723	0,999954951	0,999954951	MUC1/TGFB1/BAX/CDKN1A	4
GO:0002675	positive regulation of acute inflammatory response	4/182	13/749	0,39204723	0,999954951	0,999954951	OSMR/C3/FCER1G/IL6ST	4
GO:0006022	aminoglycan metabolic process	4/182	13/749	0,39204723	0,999954951	0,999954951	LYVE1/TGFB1/VCAN/NFKB1	4
GO:0009165	nucleotide biosynthetic process	4/182	13/749	0,39204723	0,999954951	0,999954951	STAT3/NNMT/IMPDH1/TGFB1	4
GO:0016042	lipid catabolic process	4/182	13/749	0,39204723	0,999954951	0,999954951	ALDH3A2/PLAAT4/LDLR/APOE	4
GO:0019835	cytolysis	4/182	13/749	0,39204723	0,999954951	0,999954951	C9/TGFB1/APOL1/LILRB1	4
GO:0030203	glycosaminoglycan metabolic process	4/182	13/749	0,39204723	0,999954951	0,999954951	LYVE1/TGFB1/VCAN/NFKB1	4
GO:0032607	interferon-alpha production	4/182	13/749	0,39204723	0,999954951	0,999954951	STAT1/TLR4/IRF7/TBK1	4
GO:0032647	regulation of interferon-alpha production	4/182	13/749	0,39204723	0,999954951	0,999954951	STAT1/TLR4/IRF7/TBK1	4

GO:0035304	regulation of protein dephosphorylation	4/182	13/749	0,39204723	0,999954951	0,999954951	TGFB1/FKBP1A/LILRB2/RGN	4
GO:0035966	response to topologically incorrect protein	4/182	13/749	0,39204723	0,999954951	0,999954951	HDAC6/XBP1/BAX/ATF3	4
GO:0048839	inner ear development	4/182	13/749	0,39204723	0,999954951	0,999954951	C1QB/MAF/TGFB1/GATA3	4
GO:0070266	necroptotic process	4/182	13/749	0,39204723	0,999954951	0,999954951	LY96/TLR4/BIRC3/CD14	4
GO:0071384	cellular response to corticosteroid stimulus	4/182	13/749	0,39204723	0,999954951	0,999954951	ICAM1/TGFB1/ABCC2/ERRFI1	4
GO:0098760	response to interleukin-7	4/182	13/749	0,39204723	0,999954951	0,999954951	JAK1/STAT3/JAK3/SOCS1	4
GO:0098761	cellular response to interleukin-7	4/182	13/749	0,39204723	0,999954951	0,999954951	JAK1/STAT3/JAK3/SOCS1	4
GO:1901293	nucleoside phosphate biosynthetic process	4/182	13/749	0,39204723	0,999954951	0,999954951	STAT3/NNMT/IMPDH1/TGFB1	4
GO:1904427	positive regulation of calcium ion transmembrane transport	4/182	13/749	0,39204723	0,999954951	0,999954951	EHD3/BAX/G6PD/RGN	4
GO:2001257	regulation of cation channel activity	4/182	13/749	0,39204723	0,999954951	0,999954951	EHD3/FKBP1A/CTSS/EPO	4
GO:0001562	response to protozoan	4/182	14/749	0,454241357	0,999954951	0,999954951	BCL3/GBP2/IL4R/SLC11A1	4
GO:0001836	release of cytochrome c from mitochondria	4/182	14/749	0,454241357	0,999954951	0,999954951	SOD2/BAX/TNFSF10/PLAUR	4
GO:0001892	embryonic placenta development	4/182	14/749	0,454241357	0,999954951	0,999954951	HIF1A/SOCS3/KRT8/KRT19	4
GO:0002534	cytokine production involved in inflammatory response	4/182	14/749	0,454241357	0,999954951	0,999954951	IL17RA/TLR4/MYD88/IL1R2	4
GO:0002886	regulation of myeloid leukocyte mediated immunity	4/182	14/749	0,454241357	0,999954951	0,999954951	C3/FCER1G/IL4R/ITGB2	4
GO:0006650	glycerophospholipid metabolic process	4/182	14/749	0,454241357	0,999954951	0,999954951	PLAAT4/SOCS3/SOCS1/LDLR	4
GO:0007173	epidermal growth factor receptor signaling pathway	4/182	14/749	0,454241357	0,999954951	0,999954951	TGFB1/AGT/ERRFI1/PLAUR	4
GO:0010830	regulation of myotube differentiation	4/182	14/749	0,454241357	0,999954951	0,999954951	IL4R/XBP1/GDF15/TNFSF14	4
GO:0030330	DNA damage response, signal transduction by p53 class mediatc	4/182	14/749	0,454241357	0,999954951	0,999954951	BCL3/MUC1/BAX/CDKN1A	4
GO:0032259	methylation	4/182	14/749	0,454241357	0,999954951	0,999954951	NNMT/DNMT1/MYC/GATA3	4
GO:0033028	myeloid cell apoptotic process	4/182	14/749	0,454241357	0,999954951	0,999954951	FCER1G/KITLG/IRF7/EPO	4
GO:0033273	response to vitamin	4/182	14/749	0,454241357	0,999954951	0,999954951	COL1A1/TGFB1/TNC/EPO	4
GO:0042533	tumor necrosis factor biosynthetic process	4/182	14/749	0,454241357	0,999954951	0,999954951	BCL3/TLR4/ERRFI1/LILRB1	4
GO:0042534	regulation of tumor necrosis factor biosynthetic process	4/182	14/749	0,454241357	0,999954951	0,999954951	BCL3/TLR4/ERRFI1/LILRB1	4
GO:0042832	defense response to protozoan	4/182	14/749	0,454241357	0,999954951	0,999954951	BCL3/GBP2/IL4R/SLC11A1	4
GO:0050806	positive regulation of synaptic transmission	4/182	14/749	0,454241357	0,999954951	0,999954951	MME/APOE/LILRB2/ADORA2A	4
GO:0072330	monocarboxylic acid biosynthetic process	4/182	14/749	0,454241357	0,999954951	0,999954951	XBP1/ALOX5/VCAN/RGN	4
GO:1903305	regulation of regulated secretory pathway	4/182	14/749	0,454241357	0,999954951	0,999954951	FCER1G/IL4R/ITGB2/ADORA2A	4
GO:1903364	positive regulation of cellular protein catabolic process	4/182	14/749	0,454241357	0,999954951	0,999954951	LDLR/APOE/TRIB1/RGN	4
GO:0001764	neuron migration	4/182	15/749	0,514227727	0,999954951	0,999954951	STAT3/COL3A1/BAX/GATA3	4
GO:0002673	regulation of acute inflammatory response	4/182	15/749	0,514227727	0,999954951	0,999954951	OSMR/C3/FCER1G/IL6ST	4
GO:0002719	negative regulation of cytokine production involved in immune r	4/182	15/749	0,514227727	0,999954951	0,999954951	BCL6/JAK3/TGFB1/LILRB1	4
GO:0009913	epidermal cell differentiation	4/182	15/749	0,514227727	0,999954951	0,999954951	PLAAT4/KRT8/KRT19/ERRFI1	4
GO:0042770	signal transduction in response to DNA damage	4/182	15/749	0,514227727	0,999954951	0,999954951	BCL3/MUC1/BAX/CDKN1A	4
GO:0045807	positive regulation of endocytosis	4/182	15/749	0,514227727	0,999954951	0,999954951	C3/APOE/CD14/SERPINE1	4
GO:0072089	stem cell proliferation	4/182	15/749	0,514227727	0,999954951	0,999954951	HIF1A/ABCB1/TGFB1/KITLG	4
GO:1904645	response to amyloid-beta	4/182	15/749	0,514227727	0,999954951	0,999954951	ICAM1/VCAM1/TLR4/CASP4	4
GO:2000278	regulation of DNA biosynthetic process	4/182	15/749	0,514227727	0,999954951	0,999954951	CDKN1A/MYC/NOX4/RGN	4
GO:0003208	cardiac ventricle morphogenesis	4/182	16/749	0,57102681	0,999954951	0,999954951	HIF1A/TGFB1/FKBP1A/GATA3	4
GO:0009150	purine ribonucleotide metabolic process	4/182	16/749	0,57102681	0,999954951	0,999954951	HIF1A/STAT3/IMPDH1/TGFB1	4
GO:0009259	ribonucleotide metabolic process	4/182	16/749	0,57102681	0,999954951	0,999954951	HIF1A/STAT3/IMPDH1/TGFB1	4
GO:0030500	regulation of bone mineralization	4/182	16/749	0,57102681	0,999954951	0,999954951	HIF1A/LTF/TGFB1/ISG15	4
GO:0038127	ERBB signaling pathway	4/182	16/749	0,57102681	0,999954951	0,999954951	TGFB1/AGT/ERRFI1/PLAUR	4
GO:0043583	ear development	4/182	16/749	0,57102681	0,999954951	0,999954951	C1QB/MAF/TGFB1/GATA3	4
GO:0045669	positive regulation of osteoblast differentiation	4/182	16/749	0,57102681	0,999954951	0,999954951	LTF/IFITM1/IL6R/IL6ST	4
GO:0051155	positive regulation of striated muscle cell differentiation	4/182	16/749	0,57102681	0,999954951	0,999954951	IL4R/TGFB1/GDF15/TNFSF14	4
GO:0072659	protein localization to plasma membrane	4/182	16/749	0,57102681	0,999954951	0,999954951	FCER1G/TNFRSF1A/EHD3/TGFB1	4
GO:1901617	organic hydroxy compound biosynthetic process	4/182	16/749	0,57102681	0,999954951	0,999954951	APOE/G6PD/GATA3/NFKB1	4
GO:0001656	metanephros development	4/182	17/749	0,623957429	0,999954951	0,999954951	STAT1/EGR1/MYC/GATA3	4
GO:0002714	positive regulation of B cell mediated immunity	4/182	17/749	0,623957429	0,999954951	0,999954951	C3/FCER1G/TGFB1/STAT6	4
GO:0002891	positive regulation of immunoglobulin mediated immune respor	4/182	17/749	0,623957429	0,999954951	0,999954951	C3/FCER1G/TGFB1/STAT6	4
GO:0005996	monosaccharide metabolic process	4/182	17/749	0,623957429	0,999954951	0,999954951	RORC/G6PD/ATF3/RGN	4
GO:0006006	glucose metabolic process	4/182	17/749	0,623957429	0,999954951	0,999954951	RORC/G6PD/ATF3/RGN	4
GO:0006606	protein import into nucleus	4/182	17/749	0,623957429	0,999954951	0,999954951	STAT3/TGFB1/CDKN1A/AGT	4
GO:0010469	regulation of signaling receptor activity	4/182	17/749	0,623957429	0,999954951	0,999954951	HDAC6/IL18BP/ERRFI1/SERPINE1	4
GO:0017157	regulation of exocytosis	4/182	17/749	0,623957429	0,999954951	0,999954951	FCER1G/IL4R/ITGB2/ADORA2A	4
GO:0019318	hexose metabolic process	4/182	17/749	0,623957429	0,999954951	0,999954951	RORC/G6PD/ATF3/RGN	4
GO:0032535	regulation of cellular component size	4/182	17/749	0,623957429	0,999954951	0,999954951	ICAM1/SLC12A3/APOE/FN1	4
GO:0044262	cellular carbohydrate metabolic process	4/182	17/749	0,623957429	0,999954951	0,999954951	STAT3/RORC/IL6ST/RGN	4

GO:0045429	positive regulation of nitric oxide biosynthetic process	4/182	17/749	0,623957429	0,999954951	0,999954951	ICAM1/AGT/TLR4/ITGB2	4
GO:0045581	negative regulation of T cell differentiation	4/182	17/749	0,623957429	0,999954951	0,999954951	BCL6/JAK3/IL4R/SOCS1	4
GO:0045620	negative regulation of lymphocyte differentiation	4/182	17/749	0,623957429	0,999954951	0,999954951	BCL6/JAK3/IL4R/SOCS1	4
GO:0050688	regulation of defense response to virus	4/182	17/749	0,623957429	0,999954951	0,999954951	STAT1/BIRC3/LILRB1/IFIT1	4
GO:0060415	muscle tissue morphogenesis	4/182	17/749	0,623957429	0,999954951	0,999954951	COL3A1/TGFB1/FKBP1A/MYOM2	4
GO:0061844	antimicrobial humoral immune response mediated by antimicro	4/182	17/749	0,623957429	0,999954951	0,999954951	S100A9/LTF/CXCL1/S100A12	4
GO:0071774	response to fibroblast growth factor	4/182	17/749	0,623957429	0,999954951	0,999954951	COL1A1/NR4A1/TNC/GATA3	4
GO:0120162	positive regulation of cold-induced thermogenesis	4/182	17/749	0,623957429	0,999954951	0,999954951	LCN2/IL4R/STAT6/CMKLR1	4
GO:1902041	regulation of extrinsic apoptotic signaling pathway via death dor	4/182	17/749	0,623957429	0,999954951	0,999954951	ICAM1/TNFSF10/ATF3/SERPINE1	4
GO:1903428	positive regulation of reactive oxygen species biosynthetic proce	4/182	17/749	0,623957429	0,999954951	0,999954951	ICAM1/AGT/TLR4/ITGB2	4
GO:1904407	positive regulation of nitric oxide metabolic process	4/182	17/749	0,623957429	0,999954951	0,999954951	ICAM1/AGT/TLR4/ITGB2	4
GO:1990778	protein localization to cell periphery	4/182	17/749	0,623957429	0,999954951	0,999954951	FCER1G/TNFRSF1A/EHD3/TGFB1	4
GO:0001657	ureteric bud development	4/182	18/749	0,672601122	0,999954951	0,999954951	TGFB1/AGT/MYC/GATA3	4
GO:0001823	mesonephros development	4/182	18/749	0,672601122	0,999954951	0,999954951	TGFB1/AGT/MYC/GATA3	4
GO:0002701	negative regulation of production of molecular mediator of imm	4/182	18/749	0,672601122	0,999954951	0,999954951	BCL6/JAK3/TGFB1/LILRB1	4
GO:0003170	heart valve development	4/182	18/749	0,672601122	0,999954951	0,999954951	TNFRSF1A/TGFB1/TNFRSF1B/GATA3	4
GO:0006911	phagocytosis, engulfment	4/182	18/749	0,672601122	0,999954951	0,999954951	C3/FCER1G/FCGR1A/ITGB2	4
GO:0007283	spermatogenesis	4/182	18/749	0,672601122	0,999954951	0,999954951	BCL6/BAX/BIRC3/RGN	4
GO:0008637	apoptotic mitochondrial changes	4/182	18/749	0,672601122	0,999954951	0,999954951	SOD2/BAX/TNFSF10/PLAUR	4
GO:0010324	membrane invagination	4/182	18/749	0,672601122	0,999954951	0,999954951	C3/FCER1G/FCGR1A/ITGB2	4
GO:0031647	regulation of protein stability	4/182	18/749	0,672601122	0,999954951	0,999954951	HDAC6/XBP1/CDKN1A/RNF149	4
GO:0048644	muscle organ morphogenesis	4/182	18/749	0,672601122	0,999954951	0,999954951	COL3A1/TGFB1/FKBP1A/MYOM2	4
GO:0051170	import into nucleus	4/182	18/749	0,672601122	0,999954951	0,999954951	STAT3/TGFB1/CDKN1A/AGT	4
GO:0070167	regulation of biomineral tissue development	4/182	18/749	0,672601122	0,999954951	0,999954951	HIF1A/LTF/TGFB1/ISG15	4
GO:0072163	mesonephric epithelium development	4/182	18/749	0,672601122	0,999954951	0,999954951	TGFB1/AGT/MYC/GATA3	4
GO:0072164	mesonephric tubule development	4/182	18/749	0,672601122	0,999954951	0,999954951	TGFB1/AGT/MYC/GATA3	4
GO:0099024	plasma membrane invagination	4/182	18/749	0,672601122	0,999954951	0,999954951	C3/FCER1G/FCGR1A/ITGB2	4
GO:0110149	regulation of biomineralization	4/182	18/749	0,672601122	0,999954951	0,999954951	HIF1A/LTF/TGFB1/ISG15	4
GO:1903725	regulation of phospholipid metabolic process	4/182	18/749	0,672601122	0,999954951	0,999954951	SOCS3/TGFB1/SOCS1/LDLR	4
GO:1905952	regulation of lipid localization	4/182	18/749	0,672601122	0,999954951	0,999954951	C3/AGT/APOE/NFKB1	4
GO:0001889	liver development	4/182	19/749	0,716759746	0,999954951	0,999954951	TGFB1/XBP1/HNF1A/RGN	4
GO:0002437	inflammatory response to antigenic stimulus	4/182	19/749	0,716759746	0,999954951	0,999954951	C3/FCER1G/ICAM1/GATA3	4
GO:0006163	purine nucleotide metabolic process	4/182	19/749	0,716759746	0,999954951	0,999954951	HIF1A/STAT3/IMPDH1/TGFB1	4
GO:0009100	glycoprotein metabolic process	4/182	19/749	0,716759746	0,999954951	0,999954951	HIF1A/MUC1/JAK3/VCAN	4
GO:0031032	actomyosin structure organization	4/182	19/749	0,716759746	0,999954951	0,999954951	KRT8/KRT19/NOX4/MYOM2	4
GO:0034976	response to endoplasmic reticulum stress	4/182	19/749	0,716759746	0,999954951	0,999954951	XBP1/BAX/ATF3/CASP4	4
GO:0035303	regulation of dephosphorylation	4/182	19/749	0,716759746	0,999954951	0,999954951	TGFB1/FKBP1A/LILRB2/RGN	4
GO:0040029	regulation of gene expression, epigenetic	4/182	19/749	0,716759746	0,999954951	0,999954951	STAT3/HDAC6/TGFB1/DNMT1	4
GO:0048565	digestive tract development	4/182	19/749	0,716759746	0,999954951	0,999954951	HIF1A/COL3A1/TGFB1/CDKN1A	4
GO:0061008	hepaticobiliary system development	4/182	19/749	0,716759746	0,999954951	0,999954951	TGFB1/XBP1/HNF1A/RGN	4
GO:0072521	purine-containing compound metabolic process	4/182	19/749	0,716759746	0,999954951	0,999954951	HIF1A/STAT3/IMPDH1/TGFB1	4
GO:0002639	positive regulation of immunoglobulin production	4/182	20/749	0,756411771	0,999954951	0,999954951	IL4R/TGFB1/XBP1/STAT6	4
GO:0003231	cardiac ventricle development	4/182	20/749	0,756411771	0,999954951	0,999954951	HIF1A/TGFB1/FKBP1A/GATA3	4
GO:0006968	cellular defense response	4/182	20/749	0,756411771	0,999954951	0,999954951	LY96/LILRB2/C5AR1/ADORA2A	4
GO:0007519	skeletal muscle tissue development	4/182	20/749	0,756411771	0,999954951	0,999954951	TGFB1/EGR1/MYOM2/ATF3	4
GO:0014015	positive regulation of gliogenesis	4/182	20/749	0,756411771	0,999954951	0,999954951	TGFB1/TLR2/TNFRSF1B/IL6ST	4
GO:0014902	myotube differentiation	4/182	20/749	0,756411771	0,999954951	0,999954951	IL4R/XBP1/GDF15/TNFSF14	4
GO:0032733	positive regulation of interleukin-10 production	4/182	20/749	0,756411771	0,999954951	0,999954951	BCL3/FCER1G/TLR4/TLR2	4
GO:0048232	male gamete generation	4/182	20/749	0,756411771	0,999954951	0,999954951	BCL6/BAX/BIRC3/RGN	4
GO:0051208	sequestering of calcium ion	4/182	20/749	0,756411771	0,999954951	0,999954951	TGFB1/BAX/FKBP1A/PTPN6	4
GO:0051209	release of sequestered calcium ion into cytosol	4/182	20/749	0,756411771	0,999954951	0,999954951	TGFB1/BAX/FKBP1A/PTPN6	4
GO:0051282	regulation of sequestering of calcium ion	4/182	20/749	0,756411771	0,999954951	0,999954951	TGFB1/BAX/FKBP1A/PTPN6	4
GO:0051283	negative regulation of sequestering of calcium ion	4/182	20/749	0,756411771	0,999954951	0,999954951	TGFB1/BAX/FKBP1A/PTPN6	4
GO:0002828	regulation of type 2 immune response	4/182	21/749	0,791670814	0,999954951	0,999954951	BCL6/IL4R/STAT6/GATA3	4
GO:0010595	positive regulation of endothelial cell migration	4/182	21/749	0,791670814	0,999954951	0,999954951	HIF1A/TGFB1/AGT/GATA3	4
GO:0030282	bone mineralization	4/182	21/749	0,791670814	0,999954951	0,999954951	HIF1A/LTF/TGFB1/ISG15	4
GO:0034113	heterotypic cell-cell adhesion	4/182	21/749	0,791670814	0,999954951	0,999954951	VCAM1/LILRB2/CD58/ITGB2	4
GO:0043154	negative regulation of cysteine-type endopeptidase activity invo	4/182	21/749	0,791670814	0,999954951	0,999954951	BIRC3/ADORA2A/TNFSF14/PLAUR	4
GO:0045066	regulatory T cell differentiation	4/182	21/749	0,791670814	0,999954951	0,999954951	BCL6/TGFB1/SOCS1/LILRB2	4

GO:0045589	regulation of regulatory T cell differentiation	4/182	21/749	0,791670814	0,999954951	0,999954951	BCL6/TGFB1/SOCS1/LILRB2	4
GO:0045622	regulation of T-helper cell differentiation	4/182	21/749	0,791670814	0,999954951	0,999954951	BCL6/JAK3/IL4R/NFKBIZ	4
GO:0045667	regulation of osteoblast differentiation	4/182	21/749	0,791670814	0,999954951	0,999954951	LTF/IFITM1/IL6R/IL6ST	4
GO:0051656	establishment of organelle localization	4/182	21/749	0,791670814	0,999954951	0,999954951	HIF1A/FCER1G/IL4R/CD59	4
GO:0060538	skeletal muscle organ development	4/182	21/749	0,791670814	0,999954951	0,999954951	TGFB1/EGR1/MYOM2/ATF3	4
GO:0070301	cellular response to hydrogen peroxide	4/182	21/749	0,791670814	0,999954951	0,999954951	MAPK13/LCN2/HDAC6/STAT6	4
GO:0090288	negative regulation of cellular response to growth factor stimulu	4/182	21/749	0,791670814	0,999954951	0,999954951	SOST/TGFB1/AGT/GATA3	4
GO:1903320	regulation of protein modification by small protein conjugation c	4/182	21/749	0,791670814	0,999954951	0,999954951	EGR1/FKBP1A/BIRC3/ISG15	4
GO:0030307	positive regulation of cell growth	4/182	22/749	0,822748496	0,999954951	0,999954951	S100A9/S100A8/CXCL16/FN1	4
GO:0043534	blood vessel endothelial cell migration	4/182	22/749	0,822748496	0,999954951	0,999954951	HIF1A/NR4A1/TGFB1/APOE	4
GO:0045670	regulation of osteoclast differentiation	4/182	22/749	0,822748496	0,999954951	0,999954951	LTF/TLR4/LILRB1/TMEM178A	4
GO:0062013	positive regulation of small molecule metabolic process	4/182	22/749	0,822748496	0,999954951	0,999954951	HIF1A/STAT3/NFKB1/RGN	4
GO:0070228	regulation of lymphocyte apoptotic process	4/182	22/749	0,822748496	0,999954951	0,999954951	HIF1A/BCL6/JAK3/BAX	4
GO:0097553	calcium ion transmembrane import into cytosol	4/182	22/749	0,822748496	0,999954951	0,999954951	TGFB1/BAX/FKBP1A/PTPN6	4
GO:0002704	negative regulation of leukocyte mediated immunity	4/182	23/749	0,849922625	0,999954951	0,999954951	BCL6/JAK3/LILRB1/PTPN6	4
GO:0006913	nucleocytoplasmic transport	4/182	23/749	0,849922625	0,999954951	0,999954951	STAT3/TGFB1/CDKN1A/AGT	4
GO:0032355	response to estradiol	4/182	23/749	0,849922625	0,999954951	0,999954951	STAT3/COL1A1/TGFB1/AGT	4
GO:0033135	regulation of peptidyl-serine phosphorylation	4/182	23/749	0,849922625	0,999954951	0,999954951	HDAC6/TGFB1/BAX/TBK1	4
GO:0051101	regulation of DNA binding	4/182	23/749	0,849922625	0,999954951	0,999954951	BCL3/TGFB1/GATA3/PLAUR	4
GO:0051169	nuclear transport	4/182	23/749	0,849922625	0,999954951	0,999954951	STAT3/TGFB1/CDKN1A/AGT	4
GO:0002369	T cell cytokine production	4/182	24/749	0,873510966	0,999954951	0,999954951	IL1R1/SLC11A1/TNFRSF1B/GATA3	4
GO:0044282	small molecule catabolic process	4/182	24/749	0,873510966	0,999954951	0,999954951	ALDH3A2/LYVE1/TGFB1/APOE	4
GO:0071772	response to BMP	4/182	24/749	0,873510966	0,999954951	0,999954951	SOST/EGR1/GDF15/GATA3	4
GO:0071773	cellular response to BMP stimulus	4/182	24/749	0,873510966	0,999954951	0,999954951	SOST/EGR1/GDF15/GATA3	4
GO:1901224	positive regulation of NIK/NF-kappaB signaling	4/182	24/749	0,873510966	0,999954951	0,999954951	TLR4/TLR2/CD14/TNFSF14	4
GO:0032388	positive regulation of intracellular transport	4/182	25/749	0,893850392	0,999954951	0,999954951	FCER1G/IL4R/TGFB1/ITGB2	4
GO:0032735	positive regulation of interleukin-12 production	4/182	25/749	0,893850392	0,999954951	0,999954951	TLR4/TLR2/MAPK11/LTB	4
GO:0042130	negative regulation of T cell proliferation	4/182	25/749	0,893850392	0,999954951	0,999954951	TGFB1/LILRB2/LILRB1/PTPN6	4
GO:0042267	natural killer cell mediated cytotoxicity	4/182	25/749	0,893850392	0,999954951	0,999954951	PVR/LILRB1/KIR3DL1/PTPN6	4
GO:0045123	cellular extravasation	4/182	25/749	0,893850392	0,999954951	0,999954951	IL1R1/ICAM1/VCAM1/ITGB2	4
GO:0048705	skeletal system morphogenesis	4/182	25/749	0,893850392	0,999954951	0,999954951	LTF/COL1A1/TGFB1/TIPARP	4
GO:0051928	positive regulation of calcium ion transport	4/182	25/749	0,893850392	0,999954951	0,999954951	EHD3/BAX/G6PD/RGN	4
GO:0002548	monocyte chemotaxis	4/182	26/749	0,911280901	0,999954951	0,999954951	SLAMF8/IL6R/S100A12/SERPINE1	4
GO:0007409	axonogenesis	4/182	26/749	0,911280901	0,999954951	0,999954951	HDAC6/APOE/FN1/GATA3	4
GO:0043547	positive regulation of GTPase activity	4/182	26/749	0,911280901	0,999954951	0,999954951	ICAM1/ARHGDIB/ERRFI1/RGN	4
GO:0070227	lymphocyte apoptotic process	4/182	26/749	0,911280901	0,999954951	0,999954951	HIF1A/BCL6/JAK3/BAX	4
GO:0098742	cell-cell adhesion via plasma-membrane adhesion molecules	4/182	27/749	0,926133888	0,999954951	0,999954951	ICAM1/VCAM1/PVR/ITGB2	4
GO:0002228	natural killer cell mediated immunity	4/182	28/749	0,938723954	0,999954951	0,999954951	PVR/LILRB1/KIR3DL1/PTPN6	4
GO:0046879	hormone secretion	4/182	28/749	0,938723954	0,999954951	0,999954951	HIF1A/AGT/HNF1A/GATA3	4
GO:0050709	negative regulation of protein secretion	4/182	28/749	0,938723954	0,999954951	0,999954951	APOE/IL1R2/FN1/LILRB1	4
GO:1901343	negative regulation of vasculature development	4/182	28/749	0,938723954	0,999954951	0,999954951	STAT1/DNMT1/AGT/SERPINE1	4
GO:0014065	phosphatidylinositol 3-kinase signaling	4/182	29/749	0,949343614	0,999954951	0,999954951	AGT/EPO/GATA3/PTPN6	4
GO:0032956	regulation of actin cytoskeleton organization	4/182	29/749	0,949343614	0,999954951	0,999954951	ICAM1/TGFB1/ARHGDIB/NOX4	4
GO:0048667	cell morphogenesis involved in neuron differentiation	4/182	29/749	0,949343614	0,999954951	0,999954951	HDAC6/APOE/FN1/GATA3	4
GO:0002792	negative regulation of peptide secretion	4/182	30/749	0,958260271	0,999954951	0,999954951	APOE/IL1R2/FN1/LILRB1	4
GO:0030324	lung development	4/182	30/749	0,958260271	0,999954951	0,999954951	MME/HSD11B1/TNC/ERRFI1	4
GO:0030888	regulation of B cell proliferation	4/182	30/749	0,958260271	0,999954951	0,999954951	BCL6/AHR/CDKN1A/TLR4	4
GO:0032652	regulation of interleukin-1 production	4/182	30/749	0,958260271	0,999954951	0,999954951	EGR1/TLR4/IL1R2/ERRFI1	4
GO:0048015	phosphatidylinositol-mediated signaling	4/182	30/749	0,958260271	0,999954951	0,999954951	AGT/EPO/GATA3/PTPN6	4
GO:0048017	inositol lipid-mediated signaling	4/182	30/749	0,958260271	0,999954951	0,999954951	AGT/EPO/GATA3/PTPN6	4
GO:0051224	negative regulation of protein transport	4/182	30/749	0,958260271	0,999954951	0,999954951	APOE/IL1R2/FN1/LILRB1	4
GO:1904950	negative regulation of establishment of protein localization	4/182	30/749	0,958260271	0,999954951	0,999954951	APOE/IL1R2/FN1/LILRB1	4
GO:0007254	JNK cascade	4/182	31/749	0,965714932	0,999954951	0,999954951	LTBR/TLR4/FGD2/TRIB1	4
GO:0010810	regulation of cell-substrate adhesion	4/182	31/749	0,965714932	0,999954951	0,999954951	BCL6/COL1A1/FN1/SERPINE1	4
GO:0030323	respiratory tube development	4/182	31/749	0,965714932	0,999954951	0,999954951	MME/HSD11B1/TNC/ERRFI1	4
GO:0032970	regulation of actin filament-based process	4/182	31/749	0,965714932	0,999954951	0,999954951	ICAM1/TGFB1/ARHGDIB/NOX4	4
GO:1904019	epithelial cell apoptotic process	4/182	31/749	0,965714932	0,999954951	0,999954951	KRT8/ICAM1/SERPINE1/GATA3	4
GO:0032872	regulation of stress-activated MAPK cascade	4/182	32/749	0,971922206	0,999954951	0,999954951	LTBR/TLR4/MYC/FGD2	4
GO:0070302	regulation of stress-activated protein kinase signaling cascade	4/182	32/749	0,971922206	0,999954951	0,999954951	LTBR/TLR4/MYC/FGD2	4

GO:0032386	regulation of intracellular transport	4/182	33/749	0,977071201	0,999954951	0,999954951	FCER1G/IL4R/TGFB1/ITGB2	4
GO:0032612	interleukin-1 production	4/182	33/749	0,977071201	0,999954951	0,999954951	EGR1/TLR4/IL1R2/ERRFI1	4
GO:0090092	regulation of transmembrane receptor protein serine/threonine	4/182	33/749	0,977071201	0,999954951	0,999954951	SOST/TGFB1/FKBP1A/GDF15	4
GO:0045927	positive regulation of growth	4/182	34/749	0,981327033	0,999954951	0,999954951	S100A9/S100A8/CXCL16/FN1	4
GO:0046635	positive regulation of alpha-beta T cell activation	4/182	34/749	0,981327033	0,999954951	0,999954951	IL4R/NFKBIZ/SOCS1/IL6R	4
GO:0060541	respiratory system development	4/182	34/749	0,981327033	0,999954951	0,999954951	MME/HSD11B1/TNC/ERRFI1	4
GO:0072676	lymphocyte migration	4/182	39/749	0,993555524	0,999954951	0,999954951	ICAM1/CXCL16/GATA3/TNFSF14	4
GO:0070098	chemokine-mediated signaling pathway	4/182	40/749	0,99482604	0,999954951	0,999954951	HIF1A/CXCL1/ACKR1/CMKLR1	4
GO:1990868	response to chemokine	4/182	41/749	0,995854735	0,999954951	0,999954951	HIF1A/CXCL1/ACKR1/CMKLR1	4
GO:1990869	cellular response to chemokine	4/182	41/749	0,995854735	0,999954951	0,999954951	HIF1A/CXCL1/ACKR1/CMKLR1	4
GO:0019722	calcium-mediated signaling	4/182	44/749	0,997893352	0,999954951	0,999954951	VCAM1/FKBP1A/CMKLR1/RGN	4
GO:0031341	regulation of cell killing	4/182	48/749	0,999167149	0,999954951	0,999954951	ICAM1/CD59/PVR/LILRB1	4
GO:0000768	syncytium formation by plasma membrane fusion	3/182	10/749	0,453644039	0,999954951	0,999954951	IL4R/GDF15/TNFSF14	3
GO:0001974	blood vessel remodeling	3/182	10/749	0,453644039	0,999954951	0,999954951	TGFB1/BAX/AGT	3
GO:0002088	lens development in camera-type eye	3/182	10/749	0,453644039	0,999954951	0,999954951	MAF/TGFB1/GATA3	3
GO:0002374	cytokine secretion involved in immune response	3/182	10/749	0,453644039	0,999954951	0,999954951	TLR2/TNFRSF1B/LILRB1	3
GO:0006164	purine nucleotide biosynthetic process	3/182	10/749	0,453644039	0,999954951	0,999954951	STAT3/IMPDH1/TGFB1	3
GO:0006475	internal protein amino acid acetylation	3/182	10/749	0,453644039	0,999954951	0,999954951	MUC1/TGFB1/GATA3	3
GO:0006476	protein deacetylation	3/182	10/749	0,453644039	0,999954951	0,999954951	BCL6/HDAC6/TGFB1	3
GO:0006633	fatty acid biosynthetic process	3/182	10/749	0,453644039	0,999954951	0,999954951	XBP1/ALOX5/RGN	3
GO:0006949	syncytium formation	3/182	10/749	0,453644039	0,999954951	0,999954951	IL4R/GDF15/TNFSF14	3
GO:0007520	myoblast fusion	3/182	10/749	0,453644039	0,999954951	0,999954951	IL4R/GDF15/TNFSF14	3
GO:0007589	body fluid secretion	3/182	10/749	0,453644039	0,999954951	0,999954951	HIF1A/XBP1/SLC22A2	3
GO:0016573	histone acetylation	3/182	10/749	0,453644039	0,999954951	0,999954951	MUC1/TGFB1/GATA3	3
GO:0018393	internal peptidyl-lysine acetylation	3/182	10/749	0,453644039	0,999954951	0,999954951	MUC1/TGFB1/GATA3	3
GO:0018394	peptidyl-lysine acetylation	3/182	10/749	0,453644039	0,999954951	0,999954951	MUC1/TGFB1/GATA3	3
GO:0032634	interleukin-5 production	3/182	10/749	0,453644039	0,999954951	0,999954951	IL17RA/GATA3/IL17RB	3
GO:0032674	regulation of interleukin-5 production	3/182	10/749	0,453644039	0,999954951	0,999954951	IL17RA/GATA3/IL17RB	3
GO:0032736	positive regulation of interleukin-13 production	3/182	10/749	0,453644039	0,999954951	0,999954951	IL17RA/GATA3/IL17RB	3
GO:0035601	protein deacylation	3/182	10/749	0,453644039	0,999954951	0,999954951	BCL6/HDAC6/TGFB1	3
GO:0042982	amyloid precursor protein metabolic process	3/182	10/749	0,453644039	0,999954951	0,999954951	FKBP1A/APOE/IFNGR1	3
GO:0042987	amyloid precursor protein catabolic process	3/182	10/749	0,453644039	0,999954951	0,999954951	FKBP1A/APOE/IFNGR1	3
GO:0043114	regulation of vascular permeability	3/182	10/749	0,453644039	0,999954951	0,999954951	TGFB1/APOE/ADORA2A	3
GO:0043371	negative regulation of CD4-positive, alpha-beta T cell differentia	3/182	10/749	0,453644039	0,999954951	0,999954951	BCL6/JAK3/IL4R	3
GO:0048864	stem cell development	3/182	10/749	0,453644039	0,999954951	0,999954951	HIF1A/KITLG/FN1	3
GO:0050810	regulation of steroid biosynthetic process	3/182	10/749	0,453644039	0,999954951	0,999954951	EGR1/APOE/NFKB1	3
GO:0051057	positive regulation of small GTPase mediated signal transductior	3/182	10/749	0,453644039	0,999954951	0,999954951	COL3A1/KITLG/EPO	3
GO:0060443	mammary gland morphogenesis	3/182	10/749	0,453644039	0,999954951	0,999954951	TGFB1/BAX/STAT6	3
GO:0062208	positive regulation of pattern recognition receptor signaling patl	3/182	10/749	0,453644039	0,999954951	0,999954951	LTF/TLR4/TLR2	3
GO:0072522	purine-containing compound biosynthetic process	3/182	10/749	0,453644039	0,999954951	0,999954951	STAT3/IMPDH1/TGFB1	3
GO:0072606	interleukin-8 secretion	3/182	10/749	0,453644039	0,999954951	0,999954951	TLR2/CD58/CD14	3
GO:0090199	regulation of release of cytochrome c from mitochondria	3/182	10/749	0,453644039	0,999954951	0,999954951	BAX/TNFSF10/PLAUR	3
GO:0098732	macromolecule deacylation	3/182	10/749	0,453644039	0,999954951	0,999954951	BCL6/HDAC6/TGFB1	3
GO:0140253	cell-cell fusion	3/182	10/749	0,453644039	0,999954951	0,999954951	IL4R/GDF15/TNFSF14	3
GO:1904036	negative regulation of epithelial cell apoptotic process	3/182	10/749	0,453644039	0,999954951	0,999954951	ICAM1/SERPINE1/GATA3	3
GO:1904707	positive regulation of vascular smooth muscle cell proliferation	3/182	10/749	0,453644039	0,999954951	0,999954951	ADAMTS1/DNMT1/AGT	3
GO:1905954	positive regulation of lipid localization	3/182	10/749	0,453644039	0,999954951	0,999954951	C3/APOE/NFKB1	3
GO:0006338	chromatin remodeling	3/182	11/749	0,523805477	0,999954951	0,999954951	DNMT1/MYC/GATA3	3
GO:0006473	protein acetylation	3/182	11/749	0,523805477	0,999954951	0,999954951	MUC1/TGFB1/GATA3	3
GO:0006986	response to unfolded protein	3/182	11/749	0,523805477	0,999954951	0,999954951	XBP1/BAX/ATF3	3
GO:0007566	embryo implantation	3/182	11/749	0,523805477	0,999954951	0,999954951	TIMP1/ARHGDIB/EPO	3
GO:0007612	learning	3/182	11/749	0,523805477	0,999954951	0,999954951	HIF1A/AGT/TLR2	3
GO:0008361	regulation of cell size	3/182	11/749	0,523805477	0,999954951	0,999954951	SLC12A3/APOE/FN1	3
GO:0010758	regulation of macrophage chemotaxis	3/182	11/749	0,523805477	0,999954951	0,999954951	SLAMF8/CMKLR1/C5AR1	3
GO:0014031	mesenchymal cell development	3/182	11/749	0,523805477	0,999954951	0,999954951	HIF1A/KITLG/FN1	3
GO:0014033	neural crest cell differentiation	3/182	11/749	0,523805477	0,999954951	0,999954951	HIF1A/KITLG/FN1	3
GO:0015980	energy derivation by oxidation of organic compounds	3/182	11/749	0,523805477	0,999954951	0,999954951	HIF1A/MYC/IL6ST	3
GO:0019915	lipid storage	3/182	11/749	0,523805477	0,999954951	0,999954951	C3/APOE/NFKB1	3
GO:0021987	cerebral cortex development	3/182	11/749	0,523805477	0,999954951	0,999954951	HIF1A/COL3A1/BAX	3

GO:0030858	positive regulation of epithelial cell differentiation	3/182	11/749	0,523805477	0,999954951	0,999954951	PLAAT4/CD24/SERPINE1	3
GO:0032418	lysosome localization	3/182	11/749	0,523805477	0,999954951	0,999954951	FCER1G/HDAC6/IL4R	3
GO:0032768	regulation of monooxygenase activity	3/182	11/749	0,523805477	0,999954951	0,999954951	HIF1A/APOE/NFKB1	3
GO:0032964	collagen biosynthetic process	3/182	11/749	0,523805477	0,999954951	0,999954951	COL1A1/TGFB1/ERRFI1	3
GO:0033628	regulation of cell adhesion mediated by integrin	3/182	11/749	0,523805477	0,999954951	0,999954951	MUC1/SERPINE1/PTPN6	3
GO:0043011	myeloid dendritic cell differentiation	3/182	11/749	0,523805477	0,999954951	0,999954951	LTBR/RELB/TGFB1	3
GO:0043414	macromolecule methylation	3/182	11/749	0,523805477	0,999954951	0,999954951	DNMT1/MYC/GATA3	3
GO:0043543	protein acylation	3/182	11/749	0,523805477	0,999954951	0,999954951	MUC1/TGFB1/GATA3	3
GO:0045577	regulation of B cell differentiation	3/182	11/749	0,523805477	0,999954951	0,999954951	XBP1/SLAMF8/PTPN6	3
GO:0045913	positive regulation of carbohydrate metabolic process	3/182	11/749	0,523805477	0,999954951	0,999954951	HIF1A/NFKB1/RGN	3
GO:0045921	positive regulation of exocytosis	3/182	11/749	0,523805477	0,999954951	0,999954951	FCER1G/IL4R/ITGB2	3
GO:0048662	negative regulation of smooth muscle cell proliferation	3/182	11/749	0,523805477	0,999954951	0,999954951	SOD2/CDKN1A/TRIB1	3
GO:0051053	negative regulation of DNA metabolic process	3/182	11/749	0,523805477	0,999954951	0,999954951	BCL6/GATA3/RGN	3
GO:0070373	negative regulation of ERK1 and ERK2 cascade	3/182	11/749	0,523805477	0,999954951	0,999954951	TLR4/ERRFI1/ATF3	3
GO:0072091	regulation of stem cell proliferation	3/182	11/749	0,523805477	0,999954951	0,999954951	HIF1A/TGFB1/KITLG	3
GO:1902807	negative regulation of cell cycle G1/S phase transition	3/182	11/749	0,523805477	0,999954951	0,999954951	MUC1/BAX/CDKN1A	3
GO:2000134	negative regulation of G1/S transition of mitotic cell cycle	3/182	11/749	0,523805477	0,999954951	0,999954951	MUC1/BAX/CDKN1A	3
GO:2001244	positive regulation of intrinsic apoptotic signaling pathway	3/182	11/749	0,523805477	0,999954951	0,999954951	S100A9/S100A8/BAX	3
GO:0001838	embryonic epithelial tube formation	3/182	12/749	0,588759209	0,999954951	0,999954951	HIF1A/TGFB1/GATA3	3
GO:0006690	icosanoid metabolic process	3/182	12/749	0,588759209	0,999954951	0,999954951	TNFRSF1A/ALOX5/TLR2	3
GO:0007272	ensheathment of neurons	3/182	12/749	0,588759209	0,999954951	0,999954951	TGFB1/TLR2/TNFRSF1B	3
GO:0008286	insulin receptor signaling pathway	3/182	12/749	0,588759209	0,999954951	0,999954951	SOCS3/SOCS1/AGT	3
GO:0008366	axon ensheathment	3/182	12/749	0,588759209	0,999954951	0,999954951	TGFB1/TLR2/TNFRSF1B	3
GO:0010657	muscle cell apoptotic process	3/182	12/749	0,588759209	0,999954951	0,999954951	SOD2/DNMT1/AGT	3
GO:0010660	regulation of muscle cell apoptotic process	3/182	12/749	0,588759209	0,999954951	0,999954951	SOD2/DNMT1/AGT	3
GO:0010812	negative regulation of cell-substrate adhesion	3/182	12/749	0,588759209	0,999954951	0,999954951	BCL6/COL1A1/SERPINE1	3
GO:0016050	vesicle organization	3/182	12/749	0,588759209	0,999954951	0,999954951	CD59/TAP2/TAP1	3
GO:0022617	extracellular matrix disassembly	3/182	12/749	0,588759209	0,999954951	0,999954951	TIMP1/TGFB1/CTSS	3
GO:0030279	negative regulation of ossification	3/182	12/749	0,588759209	0,999954951	0,999954951	HIF1A/SOST/TGFB1	3
GO:0030501	positive regulation of bone mineralization	3/182	12/749	0,588759209	0,999954951	0,999954951	LTF/TGFB1/ISG15	3
GO:0031016	pancreas development	3/182	12/749	0,588759209	0,999954951	0,999954951	XBP1/IL6R/HNF1A	3
GO:0031623	receptor internalization	3/182	12/749	0,588759209	0,999954951	0,999954951	FCER1G/ITGB2/LILRB1	3
GO:0042058	regulation of epidermal growth factor receptor signaling pathwa	3/182	12/749	0,588759209	0,999954951	0,999954951	AGT/ERRFI1/PLAUR	3
GO:0042552	myelination	3/182	12/749	0,588759209	0,999954951	0,999954951	TGFB1/TLR2/TNFRSF1B	3
GO:0043300	regulation of leukocyte degranulation	3/182	12/749	0,588759209	0,999954951	0,999954951	FCER1G/IL4R/ITGB2	3
GO:0045576	mast cell activation	3/182	12/749	0,588759209	0,999954951	0,999954951	FCER1G/IL4R/S100A12	3
GO:0051148	negative regulation of muscle cell differentiation	3/182	12/749	0,588759209	0,999954951	0,999954951	XBP1/DNMT1/G6PD	3
GO:0055013	cardiac muscle cell development	3/182	12/749	0,588759209	0,999954951	0,999954951	AGT/G6PD/MYOM2	3
GO:0060349	bone morphogenesis	3/182	12/749	0,588759209	0,999954951	0,999954951	LTF/COL1A1/TGFB1	3
GO:0061383	trabecula morphogenesis	3/182	12/749	0,588759209	0,999954951	0,999954951	ADAMTS1/COL1A1/FKBP1A	3
GO:0070169	positive regulation of biomineral tissue development	3/182	12/749	0,588759209	0,999954951	0,999954951	LTF/TGFB1/ISG15	3
GO:0070542	response to fatty acid	3/182	12/749	0,588759209	0,999954951	0,999954951	LDLR/TNC/TLR2	3
GO:0098657	import into cell	3/182	12/749	0,588759209	0,999954951	0,999954951	LTF/AGT/SLC12A3	3
GO:0110151	positive regulation of biomineralization	3/182	12/749	0,588759209	0,999954951	0,999954951	LTF/TGFB1/ISG15	3
GO:1901568	fatty acid derivative metabolic process	3/182	12/749	0,588759209	0,999954951	0,999954951	TNFRSF1A/ALOX5/TLR2	3
GO:1902042	negative regulation of extrinsic apoptotic signaling pathway via (	3/182	12/749	0,588759209	0,999954951	0,999954951	ICAM1/TNFSF10/SERPINE1	3
GO:1902622	regulation of neutrophil migration	3/182	12/749	0,588759209	0,999954951	0,999954951	IL1R1/SLAMF8/C5AR1	3
GO:0002065	columnar/cuboidal epithelial cell differentiation	3/182	13/749	0,647772803	0,999954951	0,999954951	HIF1A/CDKN1A/SERPINE1	3
GO:0002753	cytoplasmic pattern recognition receptor signaling pathway	3/182	13/749	0,647772803	0,999954951	0,999954951	TLR4/IRF7/BIRC3	3
GO:0008593	regulation of Notch signaling pathway	3/182	13/749	0,647772803	0,999954951	0,999954951	BCL6/STAT3/IL6ST	3
GO:0009267	cellular response to starvation	3/182	13/749	0,647772803	0,999954951	0,999954951	XBP1/CDKN1A/ATF3	3
GO:0010675	regulation of cellular carbohydrate metabolic process	3/182	13/749	0,647772803	0,999954951	0,999954951	STAT3/RORC/RGN	3
GO:0016241	regulation of macroautophagy	3/182	13/749	0,647772803	0,999954951	0,999954951	HIF1A/HDAC6/TBK1	3
GO:0016331	morphogenesis of embryonic epithelium	3/182	13/749	0,647772803	0,999954951	0,999954951	HIF1A/TGFB1/GATA3	3
GO:0031640	killing of cells of other organism	3/182	13/749	0,647772803	0,999954951	0,999954951	LTF/APOL1/S100A12	3
GO:0032368	regulation of lipid transport	3/182	13/749	0,647772803	0,999954951	0,999954951	AGT/APOE/NFKB1	3
GO:0032656	regulation of interleukin-13 production	3/182	13/749	0,647772803	0,999954951	0,999954951	IL17RA/GATA3/IL17RB	3
GO:0042594	response to starvation	3/182	13/749	0,647772803	0,999954951	0,999954951	XBP1/CDKN1A/ATF3	3
GO:0043388	positive regulation of DNA binding	3/182	13/749	0,647772803	0,999954951	0,999954951	TGFB1/GATA3/PLAUR	3

GO:0046034	ATP metabolic process	3/182	13/749	0,647772803	0,999954951	0,999954951	HIF1A/STAT3/TGFB1	3
GO:0050803	regulation of synapse structure or activity	3/182	13/749	0,647772803	0,999954951	0,999954951	APOE/TLR2/LILRB2	3
GO:0050807	regulation of synapse organization	3/182	13/749	0,647772803	0,999954951	0,999954951	APOE/TLR2/LILRB2	3
GO:0055006	cardiac cell development	3/182	13/749	0,647772803	0,999954951	0,999954951	AGT/G6PD/MYOM2	3
GO:0070229	negative regulation of lymphocyte apoptotic process	3/182	13/749	0,647772803	0,999954951	0,999954951	HIF1A/BCL6/JAK3	3
GO:0072175	epithelial tube formation	3/182	13/749	0,647772803	0,999954951	0,999954951	HIF1A/TGFB1/GATA3	3
GO:1901136	carbohydrate derivative catabolic process	3/182	13/749	0,647772803	0,999954951	0,999954951	LYVE1/TGFB1/VCAN	3
GO:1901184	regulation of ERBB signaling pathway	3/182	13/749	0,647772803	0,999954951	0,999954951	AGT/ERRFI1/PLAUR	3
GO:1902895	positive regulation of pri-miRNA transcription by RNA polymeras	3/182	13/749	0,647772803	0,999954951	0,999954951	HIF1A/STAT3/TGFB1	3
GO:2000241	regulation of reproductive process	3/182	13/749	0,647772803	0,999954951	0,999954951	TIMP1/ARHGDIB/RGN	3
GO:0001541	ovarian follicle development	3/182	14/749	0,700554106	0,999954951	0,999954951	ICAM1/BAX/KITLG	3
GO:0009101	glycoprotein biosynthetic process	3/182	14/749	0,700554106	0,999954951	0,999954951	MUC1/JAK3/VCAN	3
GO:0010803	regulation of tumor necrosis factor-mediated signaling pathway	3/182	14/749	0,700554106	0,999954951	0,999954951	TNFRSF1A/BIRC3/CASP4	3
GO:0016358	dendrite development	3/182	14/749	0,700554106	0,999954951	0,999954951	HDAC6/APOE/LST1	3
GO:0016458	gene silencing	3/182	14/749	0,700554106	0,999954951	0,999954951	STAT3/TGFB1/DNMT1	3
GO:0032616	interleukin-13 production	3/182	14/749	0,700554106	0,999954951	0,999954951	IL17RA/GATA3/IL17RB	3
GO:0034250	positive regulation of cellular amide metabolic process	3/182	14/749	0,700554106	0,999954951	0,999954951	BCL3/TNFRSF1A/IFNGR1	3
GO:0035306	positive regulation of dephosphorylation	3/182	14/749	0,700554106	0,999954951	0,999954951	TGFB1/LILRB2/RGN	3
GO:0035722	interleukin-12-mediated signaling pathway	3/182	14/749	0,700554106	0,999954951	0,999954951	JAK1/SOD2/PSME2	3
GO:0043401	steroid hormone mediated signaling pathway	3/182	14/749	0,700554106	0,999954951	0,999954951	HDAC6/NR4A1/RORC	3
GO:0045191	regulation of isotype switching	3/182	14/749	0,700554106	0,999954951	0,999954951	BCL6/TGFB1/STAT6	3
GO:0051145	smooth muscle cell differentiation	3/182	14/749	0,700554106	0,999954951	0,999954951	SOD2/TGFB1/DNMT1	3
GO:0051302	regulation of cell division	3/182	14/749	0,700554106	0,999954951	0,999954951	TGFB1/MYC/KLHL13	3
GO:0051606	detection of stimulus	3/182	14/749	0,700554106	0,999954951	0,999954951	LY96/TLR4/TLR2	3
GO:2000515	negative regulation of CD4-positive, alpha-beta T cell activation	3/182	14/749	0,700554106	0,999954951	0,999954951	BCL6/JAK3/IL4R	3
GO:0001773	myeloid dendritic cell activation	3/182	15/749	0,747135701	0,999954951	0,999954951	LTBR/RELB/TGFB1	3
GO:0002827	positive regulation of T-helper 1 type immune response	3/182	15/749	0,747135701	0,999954951	0,999954951	IL1R1/IL6R/SLC11A1	3
GO:0003014	renal system process	3/182	15/749	0,747135701	0,999954951	0,999954951	AGT/HNF1A/ADORA2A	3
GO:0007492	endoderm development	3/182	15/749	0,747135701	0,999954951	0,999954951	TGFB1/ITGB2/FN1	3
GO:0009755	hormone-mediated signaling pathway	3/182	15/749	0,747135701	0,999954951	0,999954951	HDAC6/NR4A1/RORC	3
GO:0010508	positive regulation of autophagy	3/182	15/749	0,747135701	0,999954951	0,999954951	HIF1A/HDAC6/TBK1	3
GO:0030225	macrophage differentiation	3/182	15/749	0,747135701	0,999954951	0,999954951	TGFB1/TLR2/TRIB1	3
GO:0032091	negative regulation of protein binding	3/182	15/749	0,747135701	0,999954951	0,999954951	SLPI/BAX/IFIT1	3
GO:0035850	epithelial cell differentiation involved in kidney development	3/182	15/749	0,747135701	0,999954951	0,999954951	CD24/STAT1/GATA3	3
GO:0042475	odontogenesis of dentin-containing tooth	3/182	15/749	0,747135701	0,999954951	0,999954951	BAX/TNC/SERPINE1	3
GO:0043551	regulation of phosphatidylinositol 3-kinase activity	3/182	15/749	0,747135701	0,999954951	0,999954951	SOCS3/TGFB1/SOCS1	3
GO:0044344	cellular response to fibroblast growth factor stimulus	3/182	15/749	0,747135701	0,999954951	0,999954951	COL1A1/NR4A1/GATA3	3
GO:0048844	artery morphogenesis	3/182	15/749	0,747135701	0,999954951	0,999954951	COL3A1/LDLR/APOE	3
GO:0061005	cell differentiation involved in kidney development	3/182	15/749	0,747135701	0,999954951	0,999954951	CD24/STAT1/GATA3	3
GO:0070671	response to interleukin-12	3/182	15/749	0,747135701	0,999954951	0,999954951	JAK1/SOD2/PSME2	3
GO:0071349	cellular response to interleukin-12	3/182	15/749	0,747135701	0,999954951	0,999954951	JAK1/SOD2/PSME2	3
GO:0071901	negative regulation of protein serine/threonine kinase activity	3/182	15/749	0,747135701	0,999954951	0,999954951	CDKN1A/APOE/PTPN6	3
GO:0090150	establishment of protein localization to membrane	3/182	15/749	0,747135701	0,999954951	0,999954951	CD24/BAX/ITGB2	3
GO:1902893	regulation of pri-miRNA transcription by RNA polymerase II	3/182	15/749	0,747135701	0,999954951	0,999954951	HIF1A/STAT3/TGFB1	3
GO:0000018	regulation of DNA recombination	3/182	16/749	0,787776214	0,999954951	0,999954951	BCL6/TGFB1/STAT6	3
GO:0007187	G protein-coupled receptor signaling pathway, coupled to cyclic	3/182	16/749	0,787776214	0,999954951	0,999954951	FPR1/AGT/ADORA2A	3
GO:0008217	regulation of blood pressure	3/182	16/749	0,787776214	0,999954951	0,999954951	SOD2/MME/AGT	3
GO:0009791	post-embryonic development	3/182	16/749	0,787776214	0,999954951	0,999954951	BAX/TIPARP/GATA3	3
GO:0016236	macroautophagy	3/182	16/749	0,787776214	0,999954951	0,999954951	HIF1A/HDAC6/TBK1	3
GO:0021543	pallium development	3/182	16/749	0,787776214	0,999954951	0,999954951	HIF1A/COL3A1/BAX	3
GO:0042104	positive regulation of activated T cell proliferation	3/182	16/749	0,787776214	0,999954951	0,999954951	CD24/IL6R/EPO	3
GO:0043550	regulation of lipid kinase activity	3/182	16/749	0,787776214	0,999954951	0,999954951	SOCS3/TGFB1/SOCS1	3
GO:0048246	macrophage chemotaxis	3/182	16/749	0,787776214	0,999954951	0,999954951	SLAMF8/CMKLR1/C5AR1	3
GO:0055008	cardiac muscle tissue morphogenesis	3/182	16/749	0,787776214	0,999954951	0,999954951	TGFB1/FKBP1A/MYOM2	3
GO:0055024	regulation of cardiac muscle tissue development	3/182	16/749	0,787776214	0,999954951	0,999954951	TGFB1/MAPK11/G6PD	3
GO:0060840	artery development	3/182	16/749	0,787776214	0,999954951	0,999954951	COL3A1/LDLR/APOE	3
GO:0061351	neural precursor cell proliferation	3/182	16/749	0,787776214	0,999954951	0,999954951	HIF1A/TGFB1/C5AR1	3
GO:0061756	leukocyte adhesion to vascular endothelial cell	3/182	16/749	0,787776214	0,999954951	0,999954951	ICAM1/VCAM1/ITGB2	3
GO:0071604	transforming growth factor beta production	3/182	16/749	0,787776214	0,999954951	0,999954951	HIF1A/CD24/ITGB6	3

GO:0071634	regulation of transforming growth factor beta production	3/182	16/749	0,787776214	0,999954951	0,999954951	HIF1A/CD24/ITGB6	3
GO:0072539	T-helper 17 cell differentiation	3/182	16/749	0,787776214	0,999954951	0,999954951	STAT3/NFKBIZ/RORC	3
GO:1905521	regulation of macrophage migration	3/182	16/749	0,787776214	0,999954951	0,999954951	SLAMF8/CMKLR1/C5AR1	3
GO:0002062	chondrocyte differentiation	3/182	17/749	0,822880239	0,999954951	0,999954951	MAF/TGFB1/TGFBI	3
GO:0002495	antigen processing and presentation of peptide antigen via MHC	3/182	17/749	0,822880239	0,999954951	0,999954951	FCER1G/IFI30/CTSS	3
GO:0016054	organic acid catabolic process	3/182	17/749	0,822880239	0,999954951	0,999954951	ALDH3A2/LYVE1/TGFB1	3
GO:0019886	antigen processing and presentation of exogenous peptide antig	3/182	17/749	0,822880239	0,999954951	0,999954951	FCER1G/IFI30/CTSS	3
GO:0019935	cyclic-nucleotide-mediated signaling	3/182	17/749	0,822880239	0,999954951	0,999954951	AHR/APOE/ADORA2A	3
GO:0031396	regulation of protein ubiquitination	3/182	17/749	0,822880239	0,999954951	0,999954951	FKBP1A/BIRC3/ISG15	3
GO:0032689	negative regulation of interferon-gamma production	3/182	17/749	0,822880239	0,999954951	0,999954951	TLR4/LILRB1/GATA3	3
GO:0035148	tube formation	3/182	17/749	0,822880239	0,999954951	0,999954951	HIF1A/TGFB1/GATA3	3
GO:0045824	negative regulation of innate immune response	3/182	17/749	0,822880239	0,999954951	0,999954951	SERPING1/SLAMF8/LILRB1	3
GO:0046395	carboxylic acid catabolic process	3/182	17/749	0,822880239	0,999954951	0,999954951	ALDH3A2/LYVE1/TGFB1	3
GO:0051301	cell division	3/182	17/749	0,822880239	0,999954951	0,999954951	TGFB1/MYC/KLHL13	3
GO:0051702	interaction with symbiont	3/182	17/749	0,822880239	0,999954951	0,999954951	LTF/APOE/FN1	3
GO:0055017	cardiac muscle tissue growth	3/182	17/749	0,822880239	0,999954951	0,999954951	AGT/MAPK11/G6PD	3
GO:0072538	T-helper 17 type immune response	3/182	17/749	0,822880239	0,999954951	0,999954951	STAT3/NFKBIZ/RORC	3
GO:1902905	positive regulation of supramolecular fiber organization	3/182	17/749	0,822880239	0,999954951	0,999954951	ICAM1/APOE/NOX4	3
GO:1903522	regulation of blood circulation	3/182	17/749	0,822880239	0,999954951	0,999954951	EHD3/ICAM1/AGT	3
GO:0002204	somatic recombination of immunoglobulin genes involved in imı	3/182	18/749	0,852936063	0,999954951	0,999954951	BCL6/TGFB1/STAT6	3
GO:0002208	somatic diversification of immunoglobulins involved in immune	3/182	18/749	0,852936063	0,999954951	0,999954951	BCL6/TGFB1/STAT6	3
GO:0002504	antigen processing and presentation of peptide or polysaccharid	3/182	18/749	0,852936063	0,999954951	0,999954951	FCER1G/IFI30/CTSS	3
GO:0002724	regulation of T cell cytokine production	3/182	18/749	0,852936063	0,999954951	0,999954951	IL1R1/TNFRSF1B/GATA3	3
GO:0006936	muscle contraction	3/182	18/749	0,852936063	0,999954951	0,999954951	EHD3/AGT/MYOM2	3
GO:0016447	somatic recombination of immunoglobulin gene segments	3/182	18/749	0,852936063	0,999954951	0,999954951	BCL6/TGFB1/STAT6	3
GO:0043030	regulation of macrophage activation	3/182	18/749	0,852936063	0,999954951	0,999954951	IL4R/LDLR/TLR4	3
GO:0043372	positive regulation of CD4-positive, alpha-beta T cell differentiat	3/182	18/749	0,852936063	0,999954951	0,999954951	IL4R/NFKBIZ/SOCS1	3
GO:0045190	isotype switching	3/182	18/749	0,852936063	0,999954951	0,999954951	BCL6/TGFB1/STAT6	3
GO:0050766	positive regulation of phagocytosis	3/182	18/749	0,852936063	0,999954951	0,999954951	C3/FCER1G/SLC11A1	3
GO:0050848	regulation of calcium-mediated signaling	3/182	18/749	0,852936063	0,999954951	0,999954951	FKBP1A/CMKLR1/RGN	3
GO:0061614	pri-miRNA transcription by RNA polymerase II	3/182	18/749	0,852936063	0,999954951	0,999954951	HIF1A/STAT3/TGFB1	3
GO:0002431	Fc receptor mediated stimulatory signaling pathway	3/182	19/749	0,878469122	0,999954951	0,999954951	FCER1G/FCGR1A/FCGR2A	3
GO:0002707	negative regulation of lymphocyte mediated immunity	3/182	19/749	0,878469122	0,999954951	0,999954951	BCL6/LILRB1/PTPN6	3
GO:0003015	heart process	3/182	19/749	0,878469122	0,999954951	0,999954951	EHD3/AGT/NOX4	3
GO:0009408	response to heat	3/182	19/749	0,878469122	0,999954951	0,999954951	CDKN1A/ABCC2/CD14	3
GO:0016445	somatic diversification of immunoglobulins	3/182	19/749	0,878469122	0,999954951	0,999954951	BCL6/TGFB1/STAT6	3
GO:0038034	signal transduction in absence of ligand	3/182	19/749	0,878469122	0,999954951	0,999954951	LCN2/BAX/KITLG	3
GO:0038094	Fc-gamma receptor signaling pathway	3/182	19/749	0,878469122	0,999954951	0,999954951	FCER1G/FCGR1A/FCGR2A	3
GO:0045216	cell-cell junction organization	3/182	19/749	0,878469122	0,999954951	0,999954951	TGFB1/AGT/PVR	3
GO:0060419	heart growth	3/182	19/749	0,878469122	0,999954951	0,999954951	AGT/MAPK11/G6PD	3
GO:0070231	T cell apoptotic process	3/182	19/749	0,878469122	0,999954951	0,999954951	HIF1A/JAK3/BAX	3
GO:0097192	extrinsic apoptotic signaling pathway in absence of ligand	3/182	19/749	0,878469122	0,999954951	0,999954951	LCN2/BAX/KITLG	3
GO:2000351	regulation of endothelial cell apoptotic process	3/182	19/749	0,878469122	0,999954951	0,999954951	ICAM1/SERPINE1/GATA3	3
GO:0010639	negative regulation of organelle organization	3/182	20/749	0,900008655	0,999954951	0,999954951	HDAC6/DNMT1/LILRB2	3
GO:0032620	interleukin-17 production	3/182	20/749	0,900008655	0,999954951	0,999954951	TGFB1/TLR4/MYD88	3
GO:0032660	regulation of interleukin-17 production	3/182	20/749	0,900008655	0,999954951	0,999954951	TGFB1/TLR4/MYD88	3
GO:0032835	glomerulus development	3/182	20/749	0,900008655	0,999954951	0,999954951	CD24/EGR1/IL6R	3
GO:0042391	regulation of membrane potential	3/182	20/749	0,900008655	0,999954951	0,999954951	EHD3/BAX/ADORA2A	3
GO:0002381	immunoglobulin production involved in immunoglobulin mediat	3/182	21/749	0,918064962	0,999954951	0,999954951	BCL6/TGFB1/STAT6	3
GO:0030890	positive regulation of B cell proliferation	3/182	21/749	0,918064962	0,999954951	0,999954951	BCL6/CDKN1A/TLR4	3
GO:0033138	positive regulation of peptidyl-serine phosphorylation	3/182	21/749	0,918064962	0,999954951	0,999954951	HDAC6/TGFB1/TBK1	3
GO:0043535	regulation of blood vessel endothelial cell migration	3/182	21/749	0,918064962	0,999954951	0,999954951	HIF1A/TGFB1/APOE	3
GO:0046330	positive regulation of JNK cascade	3/182	21/749	0,918064962	0,999954951	0,999954951	LTBR/TLR4/FGD2	3
GO:0072577	endothelial cell apoptotic process	3/182	21/749	0,918064962	0,999954951	0,999954951	ICAM1/SERPINE1/GATA3	3
GO:1905517	macrophage migration	3/182	21/749	0,918064962	0,999954951	0,999954951	SLAMF8/CMKLR1/C5AR1	3
GO:0016202	regulation of striated muscle tissue development	3/182	22/749	0,93311493	0,999954951	0,999954951	TGFB1/MAPK11/G6PD	3
GO:0034103	regulation of tissue remodeling	3/182	22/749	0,93311493	0,999954951	0,999954951	TGFB1/BAX/AGT	3
GO:0046006	regulation of activated T cell proliferation	3/182	22/749	0,93311493	0,999954951	0,999954951	CD24/IL6R/EPO	3
GO:0046638	positive regulation of alpha-beta T cell differentiation	3/182	22/749	0,93311493	0,999954951	0,999954951	IL4R/NFKBIZ/SOCS1	3

GO:0048634	regulation of muscle organ development	3/182	22/749	0,93311493	0,999954951	0,999954951	TGFB1/MAPK11/G6PD	3
GO:0050710	negative regulation of cytokine secretion	3/182	22/749	0,93311493	0,999954951	0,999954951	IL1R2/FN1/LILRB1	3
GO:0051100	negative regulation of binding	3/182	22/749	0,93311493	0,999954951	0,999954951	SLPI/BAX/IFIT1	3
GO:1901861	regulation of muscle tissue development	3/182	22/749	0,93311493	0,999954951	0,999954951	TGFB1/MAPK11/G6PD	3
GO:0002562	somatic diversification of immune receptors via germline recom	3/182	23/749	0,945593776	0,999954951	0,999954951	BCL6/TGFB1/STAT6	3
GO:0002763	positive regulation of myeloid leukocyte differentiation	3/182	23/749	0,945593776	0,999954951	0,999954951	TGFB1/KITLG/TRIB1	3
GO:0014068	positive regulation of phosphatidylinositol 3-kinase signaling	3/182	23/749	0,945593776	0,999954951	0,999954951	AGT/EPO/PTPN6	3
GO:0016444	somatic cell DNA recombination	3/182	23/749	0,945593776	0,999954951	0,999954951	BCL6/TGFB1/STAT6	3
GO:0016525	negative regulation of angiogenesis	3/182	23/749	0,945593776	0,999954951	0,999954951	STAT1/AGT/SERPINE1	3
GO:0030509	BMP signaling pathway	3/182	23/749	0,945593776	0,999954951	0,999954951	SOST/EGR1/GDF15	3
GO:0050798	activated T cell proliferation	3/182	23/749	0,945593776	0,999954951	0,999954951	CD24/IL6R/EPO	3
GO:0051817	modulation of process of other organism involved in symbiotic ir	3/182	23/749	0,945593776	0,999954951	0,999954951	LTF/TGFB1/APOE	3
GO:2000181	negative regulation of blood vessel morphogenesis	3/182	23/749	0,945593776	0,999954951	0,999954951	STAT1/AGT/SERPINE1	3
GO:2000516	positive regulation of CD4-positive, alpha-beta T cell activation	3/182	23/749	0,945593776	0,999954951	0,999954951	IL4R/NFKBIZ/SOCS1	3
GO:0001837	epithelial to mesenchymal transition	3/182	24/749	0,95589132	0,999954951	0,999954951	HIF1A/COL1A1/TGFB1	3
GO:0001938	positive regulation of endothelial cell proliferation	3/182	24/749	0,95589132	0,999954951	0,999954951	HIF1A/STAT3/NR4A1	3
GO:0002200	somatic diversification of immune receptors	3/182	24/749	0,95589132	0,999954951	0,999954951	BCL6/TGFB1/STAT6	3
GO:0021537	telencephalon development	3/182	24/749	0,95589132	0,999954951	0,999954951	HIF1A/COL3A1/BAX	3
GO:0032874	positive regulation of stress-activated MAPK cascade	3/182	24/749	0,95589132	0,999954951	0,999954951	LTBR/TLR4/FGD2	3
GO:0043433	negative regulation of DNA-binding transcription factor activity	3/182	24/749	0,95589132	0,999954951	0,999954951	SIGIRR/CMKLR1/TRIB1	3
GO:0070304	positive regulation of stress-activated protein kinase signaling ca	3/182	24/749	0,95589132	0,999954951	0,999954951	LTBR/TLR4/FGD2	3
GO:1904035	regulation of epithelial cell apoptotic process	3/182	24/749	0,95589132	0,999954951	0,999954951	ICAM1/SERPINE1/GATA3	3
GO:0009266	response to temperature stimulus	3/182	25/749	0,964351447	0,999954951	0,999954951	CDKN1A/ABCC2/CD14	3
GO:0035265	organ growth	3/182	25/749	0,964351447	0,999954951	0,999954951	AGT/MAPK11/G6PD	3
GO:0046328	regulation of JNK cascade	3/182	25/749	0,964351447	0,999954951	0,999954951	LTBR/TLR4/FGD2	3
GO:0007015	actin filament organization	3/182	26/749	0,971273692	0,999954951	0,999954951	ICAM1/NOX4/MYOM2	3
GO:0014066	regulation of phosphatidylinositol 3-kinase signaling	3/182	26/749	0,971273692	0,999954951	0,999954951	AGT/EPO/PTPN6	3
GO:0032651	regulation of interleukin-1 beta production	3/182	26/749	0,971273692	0,999954951	0,999954951	EGR1/TLR4/ERRFI1	3
GO:0043604	amide biosynthetic process	3/182	26/749	0,971273692	0,999954951	0,999954951	BCL3/STAT3/TNFRSF1A	3
GO:0072678	T cell migration	3/182	26/749	0,971273692	0,999954951	0,999954951	ICAM1/CXCL16/TNFSF14	3
GO:0002711	positive regulation of T cell mediated immunity	3/182	27/749	0,976916167	0,999954951	0,999954951	IL1R1/PVR/GATA3	3
GO:0006310	DNA recombination	3/182	27/749	0,976916167	0,999954951	0,999954951	BCL6/TGFB1/STAT6	3
GO:0034329	cell junction assembly	3/182	27/749	0,976916167	0,999954951	0,999954951	AGT/TLR2/FN1	3
GO:0031294	lymphocyte costimulation	3/182	28/749	0,981499233	0,999954951	0,999954951	CD24/TNFSF14/PTPN6	3
GO:0031295	T cell costimulation	3/182	28/749	0,981499233	0,999954951	0,999954951	CD24/TNFSF14/PTPN6	3
GO:0032611	interleukin-1 beta production	3/182	29/749	0,985209496	0,999954951	0,999954951	EGR1/TLR4/ERRFI1	3
GO:0007179	transforming growth factor beta receptor signaling pathway	3/182	30/749	0,988203855	0,999954951	0,999954951	COL3A1/TGFB1/GDF15	3
GO:0032623	interleukin-2 production	3/182	30/749	0,988203855	0,999954951	0,999954951	FCER1G/SLC11A1/GATA3	3
GO:0045058	T cell selection	3/182	30/749	0,988203855	0,999954951	0,999954951	STAT3/STAT6/GATA3	3
GO:1903829	positive regulation of cellular protein localization	3/182	30/749	0,988203855	0,999954951	0,999954951	FCER1G/TGFB1/ITGB2	3
GO:2001237	negative regulation of extrinsic apoptotic signaling pathway	3/182	30/749	0,988203855	0,999954951	0,999954951	ICAM1/TNFSF10/SERPINE1	3
GO:0030101	natural killer cell activation	3/182	31/749	0,990613382	0,999954951	0,999954951	IL6R/HNF1A/ITGB2	3
GO:0007389	pattern specification process	3/182	33/749	0,994094527	0,999954951	0,999954951	HIF1A/C3/C1QA	3
GO:0030900	forebrain development	3/182	33/749	0,994094527	0,999954951	0,999954951	HIF1A/COL3A1/BAX	3
GO:0001910	regulation of leukocyte mediated cytotoxicity	3/182	38/749	0,998207347	0,999954951	0,999954951	ICAM1/PVR/LILRB1	3
GO:1903827	regulation of cellular protein localization	3/182	43/749	0,999478047	0,999954951	0,999954951	FCER1G/TGFB1/ITGB2	3
GO:0001782	B cell homeostasis	2/182	10/749	0,741695722	0,999954951	0,999954951	HIF1A/BAX	2
GO:0001841	neural tube formation	2/182	10/749	0,741695722	0,999954951	0,999954951	HIF1A/TGFB1	2
GO:0002279	mast cell activation involved in immune response	2/182	10/749	0,741695722	0,999954951	0,999954951	FCER1G/IL4R	2
GO:0002448	mast cell mediated immunity	2/182	10/749	0,741695722	0,999954951	0,999954951	FCER1G/IL4R	2
GO:0007405	neuroblast proliferation	2/182	10/749	0,741695722	0,999954951	0,999954951	HIF1A/TGFB1	2
GO:0010712	regulation of collagen metabolic process	2/182	10/749	0,741695722	0,999954951	0,999954951	TGFB1/ERRFI1	2
GO:0010743	regulation of macrophage derived foam cell differentiation	2/182	10/749	0,741695722	0,999954951	0,999954951	AGT/NFKB1	2
GO:0014020	primary neural tube formation	2/182	10/749	0,741695722	0,999954951	0,999954951	HIF1A/TGFB1	2
GO:0031398	positive regulation of protein ubiquitination	2/182	10/749	0,741695722	0,999954951	0,999954951	FKBP1A/BIRC3	2
GO:0032570	response to progesterone	2/182	10/749	0,741695722	0,999954951	0,999954951	TGFB1/TLR2	2
GO:0032700	negative regulation of interleukin-17 production	2/182	10/749	0,741695722	0,999954951	0,999954951	TGFB1/TLR4	2
GO:0033559	unsaturated fatty acid metabolic process	2/182	10/749	0,741695722	0,999954951	0,999954951	TNFRSF1A/ALOX5	2
GO:0035195	gene silencing by miRNA	2/182	10/749	0,741695722	0,999954951	0,999954951	STAT3/TGFB1	2

GO:0038110	interleukin-2-mediated signaling pathway	2/182	10/749	0,741695722	0,999954951	0,999954951	JAK1/JAK3	2
GO:0042033	chemokine biosynthetic process	2/182	10/749	0,741695722	0,999954951	0,999954951	SIGIRR/EGR1	2
GO:0042752	regulation of circadian rhythm	2/182	10/749	0,741695722	0,999954951	0,999954951	RORC/ADORA2A	2
GO:0043279	response to alkaloid	2/182	10/749	0,741695722	0,999954951	0,999954951	ICAM1/ADORA2A	2
GO:0043303	mast cell degranulation	2/182	10/749	0,741695722	0,999954951	0,999954951	FCER1G/IL4R	2
GO:0043407	negative regulation of MAP kinase activity	2/182	10/749	0,741695722	0,999954951	0,999954951	APOE/PTPN6	2
GO:0045414	regulation of interleukin-8 biosynthetic process	2/182	10/749	0,741695722	0,999954951	0,999954951	BCL3/TLR4	2
GO:0045830	positive regulation of isotype switching	2/182	10/749	0,741695722	0,999954951	0,999954951	TGFB1/STAT6	2
GO:0045911	positive regulation of DNA recombination	2/182	10/749	0,741695722	0,999954951	0,999954951	TGFB1/STAT6	2
GO:0050755	chemokine metabolic process	2/182	10/749	0,741695722	0,999954951	0,999954951	SIGIRR/EGR1	2
GO:0060688	regulation of morphogenesis of a branching structure	2/182	10/749	0,741695722	0,999954951	0,999954951	TGFB1/AGT	2
GO:0070669	response to interleukin-2	2/182	10/749	0,741695722	0,999954951	0,999954951	JAK1/JAK3	2
GO:0071352	cellular response to interleukin-2	2/182	10/749	0,741695722	0,999954951	0,999954951	JAK1/JAK3	2
GO:0090398	cellular senescence	2/182	10/749	0,741695722	0,999954951	0,999954951	BCL6/CDKN1A	2
GO:0097306	cellular response to alcohol	2/182	10/749	0,741695722	0,999954951	0,999954951	AHR/TNC	2
GO:1904994	regulation of leukocyte adhesion to vascular endothelial cell	2/182	10/749	0,741695722	0,999954951	0,999954951	ICAM1/ITGB2	2
GO:1990138	neuron projection extension	2/182	10/749	0,741695722	0,999954951	0,999954951	APOE/FN1	2
GO:2000573	positive regulation of DNA biosynthetic process	2/182	10/749	0,741695722	0,999954951	0,999954951	MYC/NOX4	2
GO:2000725	regulation of cardiac muscle cell differentiation	2/182	10/749	0,741695722	0,999954951	0,999954951	TGFB1/G6PD	2
GO:0002483	antigen processing and presentation of endogenous peptide ant	2/182	11/749	0,790115776	0,999954951	0,999954951	TAP2/TAP1	2
GO:0002544	chronic inflammatory response	2/182	11/749	0,790115776	0,999954951	0,999954951	S100A8/VCAM1	2
GO:0002691	regulation of cellular extravasation	2/182	11/749	0,790115776	0,999954951	0,999954951	IL1R1/ICAM1	2
GO:0002726	positive regulation of T cell cytokine production	2/182	11/749	0,790115776	0,999954951	0,999954951	IL1R1/GATA3	2
GO:0008643	carbohydrate transport	2/182	11/749	0,790115776	0,999954951	0,999954951	C3/HNF1A	2
GO:0010463	mesenchymal cell proliferation	2/182	11/749	0,790115776	0,999954951	0,999954951	STAT1/MYC	2
GO:0010596	negative regulation of endothelial cell migration	2/182	11/749	0,790115776	0,999954951	0,999954951	TGFB1/APOE	2
GO:0010906	regulation of glucose metabolic process	2/182	11/749	0,790115776	0,999954951	0,999954951	RORC/RGN	2
GO:0015908	fatty acid transport	2/182	11/749	0,790115776	0,999954951	0,999954951	APOE/ABCC2	2
GO:0016052	carbohydrate catabolic process	2/182	11/749	0,790115776	0,999954951	0,999954951	HIF1A/STAT3	2
GO:0016441	posttranscriptional gene silencing	2/182	11/749	0,790115776	0,999954951	0,999954951	STAT3/TGFB1	2
GO:0019885	antigen processing and presentation of endogenous peptide ant	2/182	11/749	0,790115776	0,999954951	0,999954951	TAP2/TAP1	2
GO:0030224	monocyte differentiation	2/182	11/749	0,790115776	0,999954951	0,999954951	IRF7/MYC	2
GO:0031047	gene silencing by RNA	2/182	11/749	0,790115776	0,999954951	0,999954951	STAT3/TGFB1	2
GO:0032692	negative regulation of interleukin-1 production	2/182	11/749	0,790115776	0,999954951	0,999954951	IL1R2/ERRFI1	2
GO:0034763	negative regulation of transmembrane transport	2/182	11/749	0,790115776	0,999954951	0,999954951	TGFB1/EPO	2
GO:0035194	post-transcriptional gene silencing by RNA	2/182	11/749	0,790115776	0,999954951	0,999954951	STAT3/TGFB1	2
GO:0035924	cellular response to vascular endothelial growth factor stimulus	2/182	11/749	0,790115776	0,999954951	0,999954951	NR4A1/VCAM1	2
GO:0042228	interleukin-8 biosynthetic process	2/182	11/749	0,790115776	0,999954951	0,999954951	BCL3/TLR4	2
GO:0042698	ovulation cycle	2/182	11/749	0,790115776	0,999954951	0,999954951	ADAMTS1/EGR1	2
GO:0043467	regulation of generation of precursor metabolites and energy	2/182	11/749	0,790115776	0,999954951	0,999954951	HIF1A/STAT3	2
GO:0045072	regulation of interferon-gamma biosynthetic process	2/182	11/749	0,790115776	0,999954951	0,999954951	CD276/LILRB1	2
GO:0045661	regulation of myoblast differentiation	2/182	11/749	0,790115776	0,999954951	0,999954951	TGFB1/TNFSF14	2
GO:0045682	regulation of epidermis development	2/182	11/749	0,790115776	0,999954951	0,999954951	PLAAT4/ERRFI1	2
GO:0045931	positive regulation of mitotic cell cycle	2/182	11/749	0,790115776	0,999954951	0,999954951	ADAMTS1/TGFB1	2
GO:0046902	regulation of mitochondrial membrane permeability	2/182	11/749	0,790115776	0,999954951	0,999954951	STAT3/BAX	2
GO:0048259	regulation of receptor-mediated endocytosis	2/182	11/749	0,790115776	0,999954951	0,999954951	C3/SERPINE1	2
GO:0048260	positive regulation of receptor-mediated endocytosis	2/182	11/749	0,790115776	0,999954951	0,999954951	C3/SERPINE1	2
GO:0050954	sensory perception of mechanical stimulus	2/182	11/749	0,790115776	0,999954951	0,999954951	COL1A1/ICAM1	2
GO:0055021	regulation of cardiac muscle tissue growth	2/182	11/749	0,790115776	0,999954951	0,999954951	MAPK11/G6PD	2
GO:0061025	membrane fusion	2/182	11/749	0,790115776	0,999954951	0,999954951	TAP2/TAP1	2
GO:0061384	heart trabecula morphogenesis	2/182	11/749	0,790115776	0,999954951	0,999954951	ADAMTS1/FKBP1A	2
GO:0061437	renal system vasculature development	2/182	11/749	0,790115776	0,999954951	0,999954951	EGR1/IL6R	2
GO:0061440	kidney vasculature development	2/182	11/749	0,790115776	0,999954951	0,999954951	EGR1/IL6R	2
GO:0070206	protein trimerization	2/182	11/749	0,790115776	0,999954951	0,999954951	LCN2/COL1A1	2
GO:0071677	positive regulation of mononuclear cell migration	2/182	11/749	0,790115776	0,999954951	0,999954951	TGFB1/SERPINE1	2
GO:0072012	glomerulus vasculature development	2/182	11/749	0,790115776	0,999954951	0,999954951	EGR1/IL6R	2
GO:1902624	positive regulation of neutrophil migration	2/182	11/749	0,790115776	0,999954951	0,999954951	IL1R1/C5AR1	2
GO:1903131	mononuclear cell differentiation	2/182	11/749	0,790115776	0,999954951	0,999954951	IRF7/MYC	2
GO:1903322	positive regulation of protein modification by small protein conj	2/182	11/749	0,790115776	0,999954951	0,999954951	FKBP1A/BIRC3	2

GO:1905523	positive regulation of macrophage migration	2/182	11/749	0,790115776	0,999954951	0,999954951	CMKLR1/C5AR1	2
GO:0002295	T-helper cell lineage commitment	2/182	12/749	0,83038709	0,999954951	0,999954951	STAT3/STAT6	2
GO:0002577	regulation of antigen processing and presentation	2/182	12/749	0,83038709	0,999954951	0,999954951	LILRB2/SLC11A1	2
GO:0002861	regulation of inflammatory response to antigenic stimulus	2/182	12/749	0,83038709	0,999954951	0,999954951	C3/FCER1G	2
GO:0003229	ventricular cardiac muscle tissue development	2/182	12/749	0,83038709	0,999954951	0,999954951	TGFB1/FKBP1A	2
GO:0007006	mitochondrial membrane organization	2/182	12/749	0,83038709	0,999954951	0,999954951	STAT3/BAX	2
GO:0007017	microtubule-based process	2/182	12/749	0,83038709	0,999954951	0,999954951	HIF1A/HDAC6	2
GO:0007188	adenylate cyclase-modulating G protein-coupled receptor signal	2/182	12/749	0,83038709	0,999954951	0,999954951	FPR1/ADORA2A	2
GO:0008016	regulation of heart contraction	2/182	12/749	0,83038709	0,999954951	0,999954951	EHD3/AGT	2
GO:0010633	negative regulation of epithelial cell migration	2/182	12/749	0,83038709	0,999954951	0,999954951	TGFB1/APOE	2
GO:0010769	regulation of cell morphogenesis involved in differentiation	2/182	12/749	0,83038709	0,999954951	0,999954951	APOE/FN1	2
GO:0014855	striated muscle cell proliferation	2/182	12/749	0,83038709	0,999954951	0,999954951	STAT3/MAPK11	2
GO:0015718	monocarboxylic acid transport	2/182	12/749	0,83038709	0,999954951	0,999954951	APOE/ABCC2	2
GO:0019933	cAMP-mediated signaling	2/182	12/749	0,83038709	0,999954951	0,999954951	AHR/ADORA2A	2
GO:0032693	negative regulation of interleukin-10 production	2/182	12/749	0,83038709	0,999954951	0,999954951	JAK3/LILRB1	2
GO:0032740	positive regulation of interleukin-17 production	2/182	12/749	0,83038709	0,999954951	0,999954951	TGFB1/MYD88	2
GO:0035264	multicellular organism growth	2/182	12/749	0,83038709	0,999954951	0,999954951	STAT3/GDF15	2
GO:0035872	nucleotide-binding domain, leucine rich repeat containing recep	2/182	12/749	0,83038709	0,999954951	0,999954951	TLR4/BIRC3	2
GO:0036037	CD8-positive, alpha-beta T cell activation	2/182	12/749	0,83038709	0,999954951	0,999954951	SOCS1/LILRB1	2
GO:0042095	interferon-gamma biosynthetic process	2/182	12/749	0,83038709	0,999954951	0,999954951	CD276/LILRB1	2
GO:0048010	vascular endothelial growth factor receptor signaling pathway	2/182	12/749	0,83038709	0,999954951	0,999954951	HIF1A/MAPK11	2
GO:0055010	ventricular cardiac muscle tissue morphogenesis	2/182	12/749	0,83038709	0,999954951	0,999954951	TGFB1/FKBP1A	2
GO:0061035	regulation of cartilage development	2/182	12/749	0,83038709	0,999954951	0,999954951	MAF/TGFB1	2
GO:0070423	nucleotide-binding oligomerization domain containing signaling	2/182	12/749	0,83038709	0,999954951	0,999954951	TLR4/BIRC3	2
GO:0001570	vasculogenesis	2/182	13/749	0,863589688	0,999954951	0,999954951	TGFB1/TIPARP	2
GO:0001885	endothelial cell development	2/182	13/749	0,863589688	0,999954951	0,999954951	TNFRSF1A/ICAM1	2
GO:0002830	positive regulation of type 2 immune response	2/182	13/749	0,863589688	0,999954951	0,999954951	IL4R/GATA3	2
GO:0006396	RNA processing	2/182	13/749	0,863589688	0,999954951	0,999954951	TGFB1/ISG20	2
GO:0010818	T cell chemotaxis	2/182	13/749	0,863589688	0,999954951	0,999954951	CXCL16/TNFSF14	2
GO:0019883	antigen processing and presentation of endogenous antigen	2/182	13/749	0,863589688	0,999954951	0,999954951	TAP2/TAP1	2
GO:0043373	CD4-positive, alpha-beta T cell lineage commitment	2/182	13/749	0,863589688	0,999954951	0,999954951	STAT3/STAT6	2
GO:0048524	positive regulation of viral process	2/182	13/749	0,863589688	0,999954951	0,999954951	APOE/IFIT1	2
GO:0051851	modulation by host of symbiont process	2/182	13/749	0,863589688	0,999954951	0,999954951	LTF/APOE	2
GO:0060420	regulation of heart growth	2/182	13/749	0,863589688	0,999954951	0,999954951	MAPK11/G6PD	2
GO:1901796	regulation of signal transduction by p53 class mediator	2/182	13/749	0,863589688	0,999954951	0,999954951	MUC1/MAPK11	2
GO:1905207	regulation of cardiocyte differentiation	2/182	13/749	0,863589688	0,999954951	0,999954951	TGFB1/G6PD	2
GO:2000177	regulation of neural precursor cell proliferation	2/182	13/749	0,863589688	0,999954951	0,999954951	HIF1A/TGFB1	2
GO:0001960	negative regulation of cytokine-mediated signaling pathway	2/182	14/749	0,890762285	0,999954951	0,999954951	SIGIRR/IL1R2	2
GO:0002363	alpha-beta T cell lineage commitment	2/182	14/749	0,890762285	0,999954951	0,999954951	STAT3/STAT6	2
GO:0006417	regulation of translation	2/182	14/749	0,890762285	0,999954951	0,999954951	BCL3/STAT3	2
GO:0010921	regulation of phosphatase activity	2/182	14/749	0,890762285	0,999954951	0,999954951	FKBP1A/RGN	2
GO:0021915	neural tube development	2/182	14/749	0,890762285	0,999954951	0,999954951	HIF1A/TGFB1	2
GO:0032526	response to retinoic acid	2/182	14/749	0,890762285	0,999954951	0,999954951	COL1A1/TNC	2
GO:0032715	negative regulation of interleukin-6 production	2/182	14/749	0,890762285	0,999954951	0,999954951	TLR4/PTPN6	2
GO:0032753	positive regulation of interleukin-4 production	2/182	14/749	0,890762285	0,999954951	0,999954951	FCER1G/GATA3	2
GO:0035270	endocrine system development	2/182	14/749	0,890762285	0,999954951	0,999954951	IL6R/GATA3	2
GO:0043369	CD4-positive or CD8-positive, alpha-beta T cell lineage commitm	2/182	14/749	0,890762285	0,999954951	0,999954951	STAT3/STAT6	2
GO:0045624	positive regulation of T-helper cell differentiation	2/182	14/749	0,890762285	0,999954951	0,999954951	IL4R/NFKBIZ	2
GO:0050691	regulation of defense response to virus by host	2/182	14/749	0,890762285	0,999954951	0,999954951	STAT1/LILRB1	2
GO:0070232	regulation of T cell apoptotic process	2/182	14/749	0,890762285	0,999954951	0,999954951	HIF1A/JAK3	2
GO:0090559	regulation of membrane permeability	2/182	14/749	0,890762285	0,999954951	0,999954951	STAT3/BAX	2
GO:2001020	regulation of response to DNA damage stimulus	2/182	14/749	0,890762285	0,999954951	0,999954951	MUC1/MYC	2
GO:0003179	heart valve morphogenesis	2/182	15/749	0,912858682	0,999954951	0,999954951	TGFB1/GATA3	2
GO:0006839	mitochondrial transport	2/182	15/749	0,912858682	0,999954951	0,999954951	STAT3/BAX	2
GO:0007292	female gamete generation	2/182	15/749	0,912858682	0,999954951	0,999954951	ADAMTS1/IL4R	2
GO:0010862	positive regulation of pathway-restricted SMAD protein phospho	2/182	15/749	0,912858682	0,999954951	0,999954951	TGFB1/GDF15	2
GO:0035743	CD4-positive, alpha-beta T cell cytokine production	2/182	15/749	0,912858682	0,999954951	0,999954951	IL1R1/GATA3	2
GO:0043536	positive regulation of blood vessel endothelial cell migration	2/182	15/749	0,912858682	0,999954951	0,999954951	HIF1A/TGFB1	2
GO:0045445	myoblast differentiation	2/182	15/749	0,912858682	0,999954951	0,999954951	TGFB1/TNFSF14	2

GO:0050704	regulation of interleukin-1 secretion	2/182	15/749	0,912858682	0,999954951	0,999954951	TLR4/IL1R2	2
GO:0060047	heart contraction	2/182	15/749	0,912858682	0,999954951	0,999954951	EHD3/AGT	2
GO:0060761	negative regulation of response to cytokine stimulus	2/182	15/749	0,912858682	0,999954951	0,999954951	SIGIRR/IL1R2	2
GO:0071622	regulation of granulocyte chemotaxis	2/182	15/749	0,912858682	0,999954951	0,999954951	CMKLR1/C5AR1	2
GO:0090101	negative regulation of transmembrane receptor protein serine/t	2/182	15/749	0,912858682	0,999954951	0,999954951	SOST/TGFB1	2
GO:0150077	regulation of neuroinflammatory response	2/182	15/749	0,912858682	0,999954951	0,999954951	LDLR/TNFRSF1B	2
GO:0001704	formation of primary germ layer	2/182	16/749	0,930727606	0,999954951	0,999954951	ITGB2/FN1	2
GO:0030073	insulin secretion	2/182	16/749	0,930727606	0,999954951	0,999954951	HIF1A/HNF1A	2
GO:0032092	positive regulation of protein binding	2/182	16/749	0,930727606	0,999954951	0,999954951	FKBP1A/APOE	2
GO:0046620	regulation of organ growth	2/182	16/749	0,930727606	0,999954951	0,999954951	MAPK11/G6PD	2
GO:0046849	bone remodeling	2/182	16/749	0,930727606	0,999954951	0,999954951	TGFB1/NOX4	2
GO:0051495	positive regulation of cytoskeleton organization	2/182	16/749	0,930727606	0,999954951	0,999954951	ICAM1/NOX4	2
GO:1905477	positive regulation of protein localization to membrane	2/182	16/749	0,930727606	0,999954951	0,999954951	TGFB1/ITGB2	2
GO:0002360	T cell lineage commitment	2/182	17/749	0,945107255	0,999954951	0,999954951	STAT3/STAT6	2
GO:0002507	tolerance induction	2/182	17/749	0,945107255	0,999954951	0,999954951	TGFB1/LILRB2	2
GO:0032673	regulation of interleukin-4 production	2/182	17/749	0,945107255	0,999954951	0,999954951	FCER1G/GATA3	2
GO:0032731	positive regulation of interleukin-1 beta production	2/182	17/749	0,945107255	0,999954951	0,999954951	EGR1/TLR4	2
GO:0050701	interleukin-1 secretion	2/182	17/749	0,945107255	0,999954951	0,999954951	TLR4/IL1R2	2
GO:0060393	regulation of pathway-restricted SMAD protein phosphorylation	2/182	17/749	0,945107255	0,999954951	0,999954951	TGFB1/GDF15	2
GO:0060395	SMAD protein signal transduction	2/182	17/749	0,945107255	0,999954951	0,999954951	TGFB1/GDF15	2
GO:0110053	regulation of actin filament organization	2/182	17/749	0,945107255	0,999954951	0,999954951	ICAM1/NOX4	2
GO:0002433	immune response-regulating cell surface receptor signaling path	2/182	18/749	0,956628654	0,999954951	0,999954951	FCGR1A/FCGR2A	2
GO:0032732	positive regulation of interleukin-1 production	2/182	18/749	0,956628654	0,999954951	0,999954951	EGR1/TLR4	2
GO:0038096	Fc-gamma receptor signaling pathway involved in phagocytosis	2/182	18/749	0,956628654	0,999954951	0,999954951	FCGR1A/FCGR2A	2
GO:0060389	pathway-restricted SMAD protein phosphorylation	2/182	18/749	0,956628654	0,999954951	0,999954951	TGFB1/GDF15	2
GO:1901888	regulation of cell junction assembly	2/182	18/749	0,956628654	0,999954951	0,999954951	AGT/TLR2	2
GO:0032633	interleukin-4 production	2/182	19/749	0,965823893	0,999954951	0,999954951	FCER1G/GATA3	2
GO:0090100	positive regulation of transmembrane receptor protein serine/tl	2/182	19/749	0,965823893	0,999954951	0,999954951	TGFB1/GDF15	2
GO:1905475	regulation of protein localization to membrane	2/182	19/749	0,965823893	0,999954951	0,999954951	TGFB1/ITGB2	2
GO:0001952	regulation of cell-matrix adhesion	2/182	20/749	0,973136697	0,999954951	0,999954951	BCL6/SERPINE1	2
GO:0030072	peptide hormone secretion	2/182	20/749	0,973136697	0,999954951	0,999954951	HIF1A/HNF1A	2
GO:0042269	regulation of natural killer cell mediated cytotoxicity	2/182	20/749	0,973136697	0,999954951	0,999954951	PVR/LILRB1	2
GO:0090316	positive regulation of intracellular protein transport	2/182	20/749	0,973136697	0,999954951	0,999954951	TGFB1/ITGB2	2
GO:0001914	regulation of T cell mediated cytotoxicity	2/182	21/749	0,978933722	0,999954951	0,999954951	PVR/LILRB1	2
GO:0002040	sprouting angiogenesis	2/182	21/749	0,978933722	0,999954951	0,999954951	JAK1/NR4A1	2
GO:0006412	translation	2/182	21/749	0,978933722	0,999954951	0,999954951	BCL3/STAT3	2
GO:0010717	regulation of epithelial to mesenchymal transition	2/182	21/749	0,978933722	0,999954951	0,999954951	COL1A1/TGFB1	2
GO:0010718	positive regulation of epithelial to mesenchymal transition	2/182	21/749	0,978933722	0,999954951	0,999954951	COL1A1/TGFB1	2
GO:0043043	peptide biosynthetic process	2/182	21/749	0,978933722	0,999954951	0,999954951	BCL3/STAT3	2
GO:0046883	regulation of hormone secretion	2/182	21/749	0,978933722	0,999954951	0,999954951	HIF1A/AGT	2
GO:0002715	regulation of natural killer cell mediated immunity	2/182	22/749	0,983515602	0,999954951	0,999954951	PVR/LILRB1	2
GO:0043368	positive T cell selection	2/182	22/749	0,983515602	0,999954951	0,999954951	STAT3/STAT6	2
GO:0048562	embryonic organ morphogenesis	2/182	22/749	0,983515602	0,999954951	0,999954951	HIF1A/GATA3	2
GO:0007369	gastrulation	2/182	23/749	0,987127204	0,999954951	0,999954951	ITGB2/FN1	2
GO:0033157	regulation of intracellular protein transport	2/182	23/749	0,987127204	0,999954951	0,999954951	TGFB1/ITGB2	2
GO:0001913	T cell mediated cytotoxicity	2/182	25/749	0,992194254	0,999954951	0,999954951	PVR/LILRB1	2
GO:0003002	regionalization	2/182	25/749	0,992194254	0,999954951	0,999954951	C3/C1QA	2
GO:0022412	cellular process involved in reproduction in multicellular organis	2/182	25/749	0,992194254	0,999954951	0,999954951	TGFB1/BAX	2
GO:0045446	endothelial cell differentiation	2/182	26/749	0,993937635	0,999954951	0,999954951	TNFRSF1A/ICAM1	2
GO:0048247	lymphocyte chemotaxis	2/182	26/749	0,993937635	0,999954951	0,999954951	CXCL16/TNFSF14	2
GO:0003158	endothelium development	2/182	29/749	0,99718693	0,999954951	0,999954951	TNFRSF1A/ICAM1	2
GO:0050853	B cell receptor signaling pathway	2/182	32/749	0,998711459	0,999954951	0,999954951	BAX/PTPN6	2
GO:0001911	negative regulation of leukocyte mediated cytotoxicity	1/182	10/749	0,939388651	0,999954951	0,999954951	LILRB1	1
GO:0002468	dendritic cell antigen processing and presentation	1/182	10/749	0,939388651	0,999954951	0,999954951	SLC11A1	1
GO:0002604	regulation of dendritic cell antigen processing and presentation	1/182	10/749	0,939388651	0,999954951	0,999954951	SLC11A1	1
GO:0002686	negative regulation of leukocyte migration	1/182	10/749	0,939388651	0,999954951	0,999954951	SLAMF8	1
GO:0006605	protein targeting	1/182	10/749	0,939388651	0,999954951	0,999954951	ITGB2	1
GO:0006925	inflammatory cell apoptotic process	1/182	10/749	0,939388651	0,999954951	0,999954951	IRF7	1
GO:0006941	striated muscle contraction	1/182	10/749	0,939388651	0,999954951	0,999954951	EHD3	1

GO:0007088	regulation of mitotic nuclear division	1/182	10/749	0,939388651	0,999954951	0,999954951	TGFB1	1
GO:0008154	actin polymerization or depolymerization	1/182	10/749	0,939388651	0,999954951	0,999954951	ICAM1	1
GO:0009855	determination of bilateral symmetry	1/182	10/749	0,939388651	0,999954951	0,999954951	HIF1A	1
GO:0014888	striated muscle adaptation	1/182	10/749	0,939388651	0,999954951	0,999954951	ERRFI1	1
GO:0019080	viral gene expression	1/182	10/749	0,939388651	0,999954951	0,999954951	IFITM3	1
GO:0019083	viral transcription	1/182	10/749	0,939388651	0,999954951	0,999954951	IFITM3	1
GO:0030038	contractile actin filament bundle assembly	1/182	10/749	0,939388651	0,999954951	0,999954951	NOX4	1
GO:0030512	negative regulation of transforming growth factor beta receptor	1/182	10/749	0,939388651	0,999954951	0,999954951	TGFB1	1
GO:0032231	regulation of actin filament bundle assembly	1/182	10/749	0,939388651	0,999954951	0,999954951	NOX4	1
GO:0032271	regulation of protein polymerization	1/182	10/749	0,939388651	0,999954951	0,999954951	ICAM1	1
GO:0032330	regulation of chondrocyte differentiation	1/182	10/749	0,939388651	0,999954951	0,999954951	MAF	1
GO:0042177	negative regulation of protein catabolic process	1/182	10/749	0,939388651	0,999954951	0,999954951	TIMP1	1
GO:0042246	tissue regeneration	1/182	10/749	0,939388651	0,999954951	0,999954951	CDKN1A	1
GO:0043149	stress fiber assembly	1/182	10/749	0,939388651	0,999954951	0,999954951	NOX4	1
GO:0044003	modulation by symbiont of host process	1/182	10/749	0,939388651	0,999954951	0,999954951	TGFB1	1
GO:0045727	positive regulation of translation	1/182	10/749	0,939388651	0,999954951	0,999954951	BCL3	1
GO:0046824	positive regulation of nucleocytoplasmic transport	1/182	10/749	0,939388651	0,999954951	0,999954951	TGFB1	1
GO:0046887	positive regulation of hormone secretion	1/182	10/749	0,939388651	0,999954951	0,999954951	HIF1A	1
GO:0048008	platelet-derived growth factor receptor signaling pathway	1/182	10/749	0,939388651	0,999954951	0,999954951	TIPARP	1
GO:0048546	digestive tract morphogenesis	1/182	10/749	0,939388651	0,999954951	0,999954951	HIF1A	1
GO:0050716	positive regulation of interleukin-1 secretion	1/182	10/749	0,939388651	0,999954951	0,999954951	TLR4	1
GO:0050718	positive regulation of interleukin-1 beta secretion	1/182	10/749	0,939388651	0,999954951	0,999954951	TLR4	1
GO:0050869	negative regulation of B cell activation	1/182	10/749	0,939388651	0,999954951	0,999954951	BCL6	1
GO:0055025	positive regulation of cardiac muscle tissue development	1/182	10/749	0,939388651	0,999954951	0,999954951	TGFB1	1
GO:0070585	protein localization to mitochondrion	1/182	10/749	0,939388651	0,999954951	0,999954951	BAX	1
GO:0071103	DNA conformation change	1/182	10/749	0,939388651	0,999954951	0,999954951	DNMT1	1
GO:0071276	cellular response to cadmium ion	1/182	10/749	0,939388651	0,999954951	0,999954951	MT1A	1
GO:0072540	T-helper 17 cell lineage commitment	1/182	10/749	0,939388651	0,999954951	0,999954951	STAT3	1
GO:0072655	establishment of protein localization to mitochondrion	1/182	10/749	0,939388651	0,999954951	0,999954951	BAX	1
GO:0090713	immunological memory process	1/182	10/749	0,939388651	0,999954951	0,999954951	BCL6	1
GO:0140014	mitotic nuclear division	1/182	10/749	0,939388651	0,999954951	0,999954951	TGFB1	1
GO:1903076	regulation of protein localization to plasma membrane	1/182	10/749	0,939388651	0,999954951	0,999954951	TGFB1	1
GO:1903672	positive regulation of sprouting angiogenesis	1/182	10/749	0,939388651	0,999954951	0,999954951	JAK1	1
GO:1904375	regulation of protein localization to cell periphery	1/182	10/749	0,939388651	0,999954951	0,999954951	TGFB1	1
GO:2000316	regulation of T-helper 17 type immune response	1/182	10/749	0,939388651	0,999954951	0,999954951	NFKBIZ	1
GO:0002042	cell migration involved in sprouting angiogenesis	1/182	11/749	0,954315939	0,999954951	0,999954951	NR4A1	1
GO:0002643	regulation of tolerance induction	1/182	11/749	0,954315939	0,999954951	0,999954951	LILRB2	1
GO:0002710	negative regulation of T cell mediated immunity	1/182	11/749	0,954315939	0,999954951	0,999954951	LILRB1	1
GO:0007043	cell-cell junction assembly	1/182	11/749	0,954315939	0,999954951	0,999954951	AGT	1
GO:0007044	cell-substrate junction assembly	1/182	11/749	0,954315939	0,999954951	0,999954951	FN1	1
GO:0009799	specification of symmetry	1/182	11/749	0,954315939	0,999954951	0,999954951	HIF1A	1
GO:0030510	regulation of BMP signaling pathway	1/182	11/749	0,954315939	0,999954951	0,999954951	SOST	1
GO:0030857	negative regulation of epithelial cell differentiation	1/182	11/749	0,954315939	0,999954951	0,999954951	STAT1	1
GO:0031345	negative regulation of cell projection organization	1/182	11/749	0,954315939	0,999954951	0,999954951	APOE	1
GO:0032703	negative regulation of interleukin-2 production	1/182	11/749	0,954315939	0,999954951	0,999954951	GATA3	1
GO:0032816	positive regulation of natural killer cell activation	1/182	11/749	0,954315939	0,999954951	0,999954951	IL6R	1
GO:0035904	aorta development	1/182	11/749	0,954315939	0,999954951	0,999954951	COL3A1	1
GO:0045061	thymic T cell selection	1/182	11/749	0,954315939	0,999954951	0,999954951	GATA3	1
GO:0045665	negative regulation of neuron differentiation	1/182	11/749	0,954315939	0,999954951	0,999954951	APOE	1
GO:0045995	regulation of embryonic development	1/182	11/749	0,954315939	0,999954951	0,999954951	GATA3	1
GO:0048538	thymus development	1/182	11/749	0,954315939	0,999954951	0,999954951	GATA3	1
GO:0050796	regulation of insulin secretion	1/182	11/749	0,954315939	0,999954951	0,999954951	HIF1A	1
GO:0050901	leukocyte tethering or rolling	1/182	11/749	0,954315939	0,999954951	0,999954951	VCAM1	1
GO:0050922	negative regulation of chemotaxis	1/182	11/749	0,954315939	0,999954951	0,999954951	SLAMF8	1
GO:0051017	actin filament bundle assembly	1/182	11/749	0,954315939	0,999954951	0,999954951	NOX4	1
GO:0051289	protein homotetramerization	1/182	11/749	0,954315939	0,999954951	0,999954951	SOD2	1
GO:0051783	regulation of nuclear division	1/182	11/749	0,954315939	0,999954951	0,999954951	TGFB1	1
GO:0061572	actin filament bundle organization	1/182	11/749	0,954315939	0,999954951	0,999954951	NOX4	1
GO:0150115	cell-substrate junction organization	1/182	11/749	0,954315939	0,999954951	0,999954951	FN1	1

GO:1900182	positive regulation of protein localization to nucleus	1/182	11/749	0,954315939	0,999954951	0,999954951	TGFB1	1
GO:1903845	negative regulation of cellular response to transforming growth	1/182	11/749	0,954315939	0,999954951	0,999954951	TGFB1	1
GO:1904888	cranial skeletal system development	1/182	11/749	0,954315939	0,999954951	0,999954951	TGFB1	1
GO:2000108	positive regulation of leukocyte apoptotic process	1/182	11/749	0,954315939	0,999954951	0,999954951	BAX	1
GO:2000778	positive regulation of interleukin-6 secretion	1/182	11/749	0,954315939	0,999954951	0,999954951	XBP1	1
GO:0006281	DNA repair	1/182	12/749	0,965582198	0,999954951	0,999954951	ISG15	1
GO:0007163	establishment or maintenance of cell polarity	1/182	12/749	0,965582198	0,999954951	0,999954951	GATA3	1
GO:0010256	endomembrane system organization	1/182	12/749	0,965582198	0,999954951	0,999954951	EHD3	1
GO:0030902	hindbrain development	1/182	12/749	0,965582198	0,999954951	0,999954951	C5AR1	1
GO:0031342	negative regulation of cell killing	1/182	12/749	0,965582198	0,999954951	0,999954951	LILRB1	1
GO:0034105	positive regulation of tissue remodeling	1/182	12/749	0,965582198	0,999954951	0,999954951	BAX	1
GO:0034405	response to fluid shear stress	1/182	12/749	0,965582198	0,999954951	0,999954951	TGFB1	1
GO:0034605	cellular response to heat	1/182	12/749	0,965582198	0,999954951	0,999954951	CDKN1A	1
GO:0043552	positive regulation of phosphatidylinositol 3-kinase activity	1/182	12/749	0,965582198	0,999954951	0,999954951	TGFB1	1
GO:0045844	positive regulation of striated muscle tissue development	1/182	12/749	0,965582198	0,999954951	0,999954951	TGFB1	1
GO:0048636	positive regulation of muscle organ development	1/182	12/749	0,965582198	0,999954951	0,999954951	TGFB1	1
GO:0050856	regulation of T cell receptor signaling pathway	1/182	12/749	0,965582198	0,999954951	0,999954951	PTPN6	1
GO:0060021	roof of mouth development	1/182	12/749	0,965582198	0,999954951	0,999954951	TIPARP	1
GO:0110020	regulation of actomyosin structure organization	1/182	12/749	0,965582198	0,999954951	0,999954951	NOX4	1
GO:1901623	regulation of lymphocyte chemotaxis	1/182	12/749	0,965582198	0,999954951	0,999954951	TNFSF14	1
GO:1901863	positive regulation of muscle tissue development	1/182	12/749	0,965582198	0,999954951	0,999954951	TGFB1	1
GO:0000280	nuclear division	1/182	13/749	0,974081574	0,999954951	0,999954951	TGFB1	1
GO:0002407	dendritic cell chemotaxis	1/182	13/749	0,974081574	0,999954951	0,999954951	SLAMF8	1
GO:0003281	ventricular septum development	1/182	13/749	0,974081574	0,999954951	0,999954951	GATA3	1
GO:0010524	positive regulation of calcium ion transport into cytosol	1/182	13/749	0,974081574	0,999954951	0,999954951	BAX	1
GO:0030326	embryonic limb morphogenesis	1/182	13/749	0,974081574	0,999954951	0,999954951	BAX	1
GO:0035113	embryonic appendage morphogenesis	1/182	13/749	0,974081574	0,999954951	0,999954951	BAX	1
GO:0036336	dendritic cell migration	1/182	13/749	0,974081574	0,999954951	0,999954951	SLAMF8	1
GO:0045601	regulation of endothelial cell differentiation	1/182	13/749	0,974081574	0,999954951	0,999954951	TNFRSF1A	1
GO:0046686	response to cadmium ion	1/182	13/749	0,974081574	0,999954951	0,999954951	MT1A	1
GO:0048305	immunoglobulin secretion	1/182	13/749	0,974081574	0,999954951	0,999954951	XBP1	1
GO:0051023	regulation of immunoglobulin secretion	1/182	13/749	0,974081574	0,999954951	0,999954951	XBP1	1
GO:0090218	positive regulation of lipid kinase activity	1/182	13/749	0,974081574	0,999954951	0,999954951	TGFB1	1
GO:1903727	positive regulation of phospholipid metabolic process	1/182	13/749	0,974081574	0,999954951	0,999954951	TGFB1	1
GO:2000406	positive regulation of T cell migration	1/182	13/749	0,974081574	0,999954951	0,999954951	TNFSF14	1
GO:0003151	outflow tract morphogenesis	1/182	14/749	0,98049075	0,999954951	0,999954951	HIF1A	1
GO:0019233	sensory perception of pain	1/182	14/749	0,98049075	0,999954951	0,999954951	MME	1
GO:0035107	appendage morphogenesis	1/182	14/749	0,98049075	0,999954951	0,999954951	BAX	1
GO:0035108	limb morphogenesis	1/182	14/749	0,98049075	0,999954951	0,999954951	BAX	1
GO:0043525	positive regulation of neuron apoptotic process	1/182	14/749	0,98049075	0,999954951	0,999954951	BAX	1
GO:0045453	bone resorption	1/182	14/749	0,98049075	0,999954951	0,999954951	NOX4	1
GO:0046822	regulation of nucleocytoplasmic transport	1/182	14/749	0,98049075	0,999954951	0,999954951	TGFB1	1
GO:0048706	embryonic skeletal system development	1/182	14/749	0,98049075	0,999954951	0,999954951	COL1A1	1
GO:0048736	appendage development	1/182	14/749	0,98049075	0,999954951	0,999954951	BAX	1
GO:0050706	regulation of interleukin-1 beta secretion	1/182	14/749	0,98049075	0,999954951	0,999954951	TLR4	1
GO:0051258	protein polymerization	1/182	14/749	0,98049075	0,999954951	0,999954951	ICAM1	1
GO:0060173	limb development	1/182	14/749	0,98049075	0,999954951	0,999954951	BAX	1
GO:0072604	interleukin-6 secretion	1/182	14/749	0,98049075	0,999954951	0,999954951	XBP1	1
GO:0090276	regulation of peptide hormone secretion	1/182	14/749	0,98049075	0,999954951	0,999954951	HIF1A	1
GO:1900180	regulation of protein localization to nucleus	1/182	14/749	0,98049075	0,999954951	0,999954951	TGFB1	1
GO:1903670	regulation of sprouting angiogenesis	1/182	14/749	0,98049075	0,999954951	0,999954951	JAK1	1
GO:0032088	negative regulation of NF-kappaB transcription factor activity	1/182	15/749	0,985321612	0,999954951	0,999954951	CMKLR1	1
GO:0032814	regulation of natural killer cell activation	1/182	15/749	0,985321612	0,999954951	0,999954951	IL6R	1
GO:0045954	positive regulation of natural killer cell mediated cytotoxicity	1/182	15/749	0,985321612	0,999954951	0,999954951	PVR	1
GO:0051262	protein tetramerization	1/182	15/749	0,985321612	0,999954951	0,999954951	SOD2	1
GO:0007411	axon guidance	1/182	16/749	0,988961212	0,999954951	0,999954951	GATA3	1
GO:0010811	positive regulation of cell-substrate adhesion	1/182	16/749	0,988961212	0,999954951	0,999954951	FN1	1
GO:0017015	regulation of transforming growth factor beta receptor signaling	1/182	16/749	0,988961212	0,999954951	0,999954951	TGFB1	1
GO:0048285	organelle fission	1/182	16/749	0,988961212	0,999954951	0,999954951	TGFB1	1

GO:0050702	interleukin-1 beta secretion	1/182	16/749	0,988961212	0,999954951	0,999954951	TLR4	1
GO:0097485	neuron projection guidance	1/182	16/749	0,988961212	0,999954951	0,999954951	GATA3	1
GO:2000403	positive regulation of lymphocyte migration	1/182	16/749	0,988961212	0,999954951	0,999954951	TNFSF14	1
GO:2000404	regulation of T cell migration	1/182	16/749	0,988961212	0,999954951	0,999954951	TNFSF14	1
GO:0001916	positive regulation of T cell mediated cytotoxicity	1/182	17/749	0,991702084	0,999954951	0,999954951	PVR	1
GO:0002717	positive regulation of natural killer cell mediated immunity	1/182	17/749	0,991702084	0,999954951	0,999954951	PVR	1
GO:0072132	mesenchyme morphogenesis	1/182	17/749	0,991702084	0,999954951	0,999954951	MYC	1
GO:1903844	regulation of cellular response to transforming growth factor be	1/182	17/749	0,991702084	0,999954951	0,999954951	TGFB1	1
GO:0003279	cardiac septum development	1/182	18/749	0,993765227	0,999954951	0,999954951	GATA3	1
GO:0007281	germ cell development	1/182	18/749	0,993765227	0,999954951	0,999954951	BAX	1
GO:0019827	stem cell population maintenance	1/182	18/749	0,993765227	0,999954951	0,999954951	STAT3	1
GO:0050854	regulation of antigen receptor-mediated signaling pathway	1/182	20/749	0,996484936	0,999954951	0,999954951	PTPN6	1
GO:0071248	cellular response to metal ion	1/182	20/749	0,996484936	0,999954951	0,999954951	MT1A	1
GO:0098727	maintenance of cell number	1/182	20/749	0,996484936	0,999954951	0,999954951	STAT3	1
GO:0048639	positive regulation of developmental growth	1/182	21/749	0,997362496	0,999954951	0,999954951	FN1	1
GO:2000401	regulation of lymphocyte migration	1/182	21/749	0,997362496	0,999954951	0,999954951	TNFSF14	1
GO:0071241	cellular response to inorganic substance	1/182	22/749	0,998021872	0,999954951	0,999954951	MT1A	1
GO:0033077	T cell differentiation in thymus	1/182	24/749	0,998888835	0,999954951	0,999954951	GATA3	1
GO:0008037	cell recognition	1/182	25/749	0,999167775	0,999954951	0,999954951	VCAN	1
GO:0032663	regulation of interleukin-2 production	1/182	25/749	0,999167775	0,999954951	0,999954951	GATA3	1
GO:0046777	protein autophosphorylation	1/182	27/749	0,999533813	0,999954951	0,999954951	ERRFI1	1
GO:0001912	positive regulation of leukocyte mediated cytotoxicity	1/182	30/749	0,999805231	0,999954951	0,999954951	PVR	1
GO:0031343	positive regulation of cell killing	1/182	35/749	0,999954951	0,999954951	0,999954951	PVR	1
