# Supplementary material for: Can Gene Expression Analysis in Zero-Time Biopsies Predict Kidney Transplant Rejection?
Source: Front Med (Lausanne). 2022 Mar 30;9:793744. doi: 10.3389/fmed.2022.793744 (PMC9005644; doi:10.3389/fmed.2022.793744)
Supplement: Supplementary file 7 [file Table_7.pdf]

## Supplementary data 7: Pathway enrincment GO rejected vs non-rejected

| ID | Description | GeneRatio | BgRatio | pvalue | p.adjust | qvalue | geneID | Count |
|---|---|---|---|---|---|---|---|---|
| GO:0006911 | phagocytosis, engulfment | 8/11 | 18/749 | 2,94E-12 | 3,83E-10 | 3,27E-10 | IGHG2/IGHG3/IGKC/IGHG4/IGHA1/IGHG1/IGLC1/IGHM | 8 |
| GO:0010324 | membrane invagination | 8/11 | 18/749 | 2,94E-12 | 3,83E-10 | 3,27E-10 | IGHG2/IGHG3/IGKC/IGHG4/IGHA1/IGHG1/IGLC1/IGHM | 8 |
| GO:0099024 | plasma membrane invagination | 8/11 | 18/749 | 2,94E-12 | 3,83E-10 | 3,27E-10 | IGHG2/IGHG3/IGKC/IGHG4/IGHA1/IGHG1/IGLC1/IGHM | 8 |
| GO:0006958 | complement activation, classical pathway | 8/11 | 22/749 | 2,12E-11 | 2,07E-09 | 1,77E-09 | IGHG2/IGHG3/IGKC/IGHG4/IGHA1/IGHG1/IGLC1/IGHM | 8 |
| GO:0008037 | cell recognition | 8/11 | 25/749 | 7,09E-11 | 5,53E-09 | 4,73E-09 | IGHG2/IGHG3/IGKC/IGHG4/IGHA1/IGHG1/IGLC1/IGHM | 8 |
| GO:0002455 | humoral immune response mediated by circ | 8/11 | 30/749 | 3,77E-10 | 2,45E-08 | 2,09E-08 | IGHG2/IGHG3/IGKC/IGHG4/IGHA1/IGHG1/IGLC1/IGHM | 8 |
| GO:0006956 | complement activation | 8/11 | 31/749 | 5,06E-10 | 2,82E-08 | 2,41E-08 | IGHG2/IGHG3/IGKC/IGHG4/IGHA1/IGHG1/IGLC1/IGHM | 8 |
| GO:0050853 | B cell receptor signaling pathway | 8/11 | 32/749 | 6,72E-10 | 3,28E-08 | 2,80E-08 | IGHG2/IGHG3/IGKC/IGHG4/IGHA1/IGHG1/IGLC1/IGHM | 8 |
| GO:0061024 | membrane organization | 9/11 | 64/749 | 6,77E-09 | 2,93E-07 | 2,51E-07 | IGHG2/IGHG3/IGKC/IGHG4/IGHA1/IGHG1/IGLC1/IGHM/CD24 | 9 |
| GO:0050871 | positive regulation of B cell activation | 8/11 | 43/749 | 8,89E-09 | 3,47E-07 | 2,97E-07 | IGHG2/IGHG3/IGKC/IGHG4/IGHA1/IGHG1/IGLC1/IGHM | 8 |
| GO:0002433 | immune response-regulating cell surface re | 6/11 | 18/749 | 3,33E-08 | 1,08E-06 | 9,25E-07 | IGHG2/IGHG3/IGKC/IGHG4/IGHG1/IGLC1 | 6 |
| GO:0038096 | Fc-gamma receptor signaling pathway invol | 6/11 | 18/749 | 3,33E-08 | 1,08E-06 | 9,25E-07 | IGHG2/IGHG3/IGKC/IGHG4/IGHG1/IGLC1 | 6 |
| GO:0002431 | Fc receptor mediated stimulatory signaling | 6/11 | 19/749 | 4,84E-08 | 1,35E-06 | 1,15E-06 | IGHG2/IGHG3/IGKC/IGHG4/IGHG1/IGLC1 | 6 |
| GO:0038094 | Fc-gamma receptor signaling pathway | 6/11 | 19/749 | 4,84E-08 | 1,35E-06 | 1,15E-06 | IGHG2/IGHG3/IGKC/IGHG4/IGHG1/IGLC1 | 6 |
| GO:0006909 | phagocytosis | 8/11 | 55/749 | 7,13E-08 | 1,85E-06 | 1,59E-06 | IGHG2/IGHG3/IGKC/IGHG4/IGHA1/IGHG1/IGLC1/IGHM | 8 |
| GO:0050864 | regulation of B cell activation | 8/11 | 57/749 | 9,61E-08 | 2,34E-06 | 2,00E-06 | IGHG2/IGHG3/IGKC/IGHG4/IGHA1/IGHG1/IGLC1/IGHM | 8 |
| GO:0016064 | immunoglobulin mediated immune respons | 8/11 | 58/749 | 1,11E-07 | 2,55E-06 | 2,18E-06 | IGHG2/IGHG3/IGKC/IGHG4/IGHA1/IGHG1/IGLC1/IGHM | 8 |
| GO:0019724 | B cell mediated immunity | 8/11 | 60/749 | 1,47E-07 | 3,19E-06 | 2,73E-06 | IGHG2/IGHG3/IGKC/IGHG4/IGHA1/IGHG1/IGLC1/IGHM | 8 |
| GO:0030449 | regulation of complement activation | 6/11 | 25/749 | 3,05E-07 | 6,25E-06 | 5,35E-06 | IGHG2/IGHG3/IGKC/IGHG4/IGHG1/IGLC1 | 6 |
| GO:0006897 | endocytosis | 8/11 | 67/749 | 3,65E-07 | 6,78E-06 | 5,80E-06 | IGHG2/IGHG3/IGKC/IGHG4/IGHA1/IGHG1/IGLC1/IGHM | 8 |
| GO:0042742 | defense response to bacterium | 8/11 | 67/749 | 3,65E-07 | 6,78E-06 | 5,80E-06 | IGHG2/IGHG3/IGKC/IGHG4/IGHA1/IGHG1/IGLC1/IGHM | 8 |
| GO:0050851 | antigen receptor-mediated signaling pathw | 8/11 | 74/749 | 8,21E-07 | 1,45E-05 | 1,24E-05 | IGHG2/IGHG3/IGKC/IGHG4/IGHA1/IGHG1/IGLC1/IGHM | 8 |
| GO:0002764 | immune response-regulating signaling path | 9/11 | 110/749 | 1,01E-06 | 1,64E-05 | 1,40E-05 | IGHG2/IGHG3/IGKC/IGHG4/IGHA1/IGHG1/IGLC1/IGHM/CD24 | 9 |
| GO:0002768 | immune response-regulating cell surface re | 9/11 | 110/749 | 1,01E-06 | 1,64E-05 | 1,40E-05 | IGHG2/IGHG3/IGKC/IGHG4/IGHA1/IGHG1/IGLC1/IGHM/CD24 | 9 |
| GO:0002920 | regulation of humoral immune response | 6/11 | 32/749 | 1,50E-06 | 2,33E-05 | 2,00E-05 | IGHG2/IGHG3/IGKC/IGHG4/IGHG1/IGLC1 | 6 |
| GO:0006959 | humoral immune response | 8/11 | 85/749 | 2,51E-06 | 3,62E-05 | 3,10E-05 | IGHG2/IGHG3/IGKC/IGHG4/IGHA1/IGHG1/IGLC1/IGHM | 8 |
| GO:0042113 | B cell activation | 8/11 | 85/749 | 2,51E-06 | 3,62E-05 | 3,10E-05 | IGHG2/IGHG3/IGKC/IGHG4/IGHA1/IGHG1/IGLC1/IGHM | 8 |
| GO:0051251 | positive regulation of lymphocyte activatior | 9/11 | 131/749 | 4,84E-06 | 6,74E-05 | 5,77E-05 | IGHG2/IGHG3/IGKC/IGHG4/IGHA1/IGHG1/IGLC1/IGHM/CD24 | 9 |
| GO:0002429 | immune response-activating cell surface rec | 8/11 | 94/749 | 5,60E-06 | 7,28E-05 | 6,23E-05 | IGHG2/IGHG3/IGKC/IGHG4/IGHA1/IGHG1/IGLC1/IGHM | 8 |
| GO:0002757 | immune response-activating signal transduc | 8/11 | 94/749 | 5,60E-06 | 7,28E-05 | 6,23E-05 | IGHG2/IGHG3/IGKC/IGHG4/IGHA1/IGHG1/IGLC1/IGHM | 8 |
| GO:0038093 | Fc receptor signaling pathway | 6/11 | 42/749 | 8,16E-06 | 0,000102603 | 8,78E-05 | IGHG2/IGHG3/IGKC/IGHG4/IGHG1/IGLC1 | 6 |
| GO:0002696 | positive regulation of leukocyte activation | 9/11 | 141/749 | 9,31E-06 | 0,000113434 | 9,71E-05 | IGHG2/IGHG3/IGKC/IGHG4/IGHA1/IGHG1/IGLC1/IGHM/CD24 | 9 |
| GO:0050867 | positive regulation of cell activation | 9/11 | 145/749 | 1,19E-05 | 0,000140886 | 0,000120542 | IGHG2/IGHG3/IGKC/IGHG4/IGHA1/IGHG1/IGLC1/IGHM/CD24 | 9 |
| GO:0002449 | lymphocyte mediated immunity | 8/11 | 113/749 | 2,38E-05 | 0,000273385 | 0,000233908 | IGHG2/IGHG3/IGKC/IGHG4/IGHA1/IGHG1/IGLC1/IGHM | 8 |
| GO:0002253 | activation of immune response | 8/11 | 114/749 | 2,55E-05 | 0,000284446 | 0,000243372 | IGHG2/IGHG3/IGKC/IGHG4/IGHA1/IGHG1/IGLC1/IGHM | 8 |
| GO:0009617 | response to bacterium | 9/11 | 170/749 | 4,81E-05 | 0,000516428 | 0,000441856 | IGHG2/IGHG3/IGKC/IGHG4/IGHA1/IGHG1/IGLC1/IGHM/CD24 | 9 |
| GO:0002460 | adaptive immune response based on somat | 8/11 | 124/749 | 4,90E-05 | 0,000516428 | 0,000441856 | IGHG2/IGHG3/IGKC/IGHG4/IGHA1/IGHG1/IGLC1/IGHM | 8 |
| GO:0051249 | regulation of lymphocyte activation | 9/11 | 172/749 | 5,33E-05 | 0,000546774 | 0,00046782 | IGHG2/IGHG3/IGKC/IGHG4/IGHA1/IGHG1/IGLC1/IGHM/CD24 | 9 |
| GO:0002443 | leukocyte mediated immunity | 9/11 | 182/749 | 8,70E-05 | 0,000847881 | 0,000725447 | IGHG2/IGHG3/IGKC/IGHG4/IGHA1/IGHG1/IGLC1/IGHM/SERPINA3 | 9 |
| GO:0016192 | vesicle-mediated transport | 9/11 | 182/749 | 8,70E-05 | 0,000847881 | 0,000725447 | IGHG2/IGHG3/IGKC/IGHG4/IGHA1/IGHG1/IGLC1/IGHM/SERPINA3 | 9 |
| GO:0002694 | regulation of leukocyte activation | 9/11 | 184/749 | 9,56E-05 | 0,000909117 | 0,000777841 | IGHG2/IGHG3/IGKC/IGHG4/IGHA1/IGHG1/IGLC1/IGHM/CD24 | 9 |
| GO:0050865 | regulation of cell activation | 9/11 | 192/749 | 0,000137901 | 0,001280512 | 0,001095607 | IGHG2/IGHG3/IGKC/IGHG4/IGHA1/IGHG1/IGLC1/IGHM/CD24 | 9 |
| GO:0045087 | innate immune response | 9/11 | 229/749 | 0,000617018 | 0,005596214 | 0,004788123 | IGHG2/IGHG3/IGKC/IGHG4/IGHA1/IGHG1/IGLC1/IGHM/CIITA | 9 |
| GO:0046649 | lymphocyte activation | 9/11 | 235/749 | 0,000766509 | 0,006794058 | 0,005813 | IGHG2/IGHG3/IGKC/IGHG4/IGHA1/IGHG1/IGLC1/IGHM/CD24 | 9 |
| GO:0045321 | leukocyte activation | 10/11 | 306/749 | 0,000829514 | 0,007189117 | 0,006151013 | IGHG2/IGHG3/IGKC/IGHG4/IGHA1/IGHG1/IGLC1/IGHM/CD24/SERPINA3 | 10 |
| GO:0002250 | adaptive immune response | 8/11 | 185/749 | 0,000996517 | 0,008448727 | 0,007228735 | IGHG2/IGHG3/IGKC/IGHG4/IGHA1/IGHG1/IGLC1/IGHM | 8 |
| GO:0050778 | positive regulation of immune response | 8/11 | 191/749 | 0,001257851 | 0,010437487 | 0,008930319 | IGHG2/IGHG3/IGKC/IGHG4/IGHA1/IGHG1/IGLC1/IGHM | 8 |
| GO:0001894 | tissue homeostasis | 4/11 | 38/749 | 0,001443879 | 0,011731516 | 0,010037491 | IGHG3/IGKC/IGHA1/SERPINA3 | 4 |

| | | | | | | | | |
|---|---|---|---|---|---|---|---|---|
| GO:0002252 | immune effector process | 9/11 | 261/749 | 0,001831986 | 0,014458746 | 0,012370911 | IGHG2/IGHG3/IGKC/IGHG4/IGHA1/IGHG1/IGLC1/IGHM/SERPINA3 | 9 |
| GO:0001775 | cell activation | 10/11 | 333/749 | 0,001853685 | 0,014458746 | 0,012370911 | IGHG2/IGHG3/IGKC/IGHG4/IGHA1/IGHG1/IGLC1/IGHM/CD24/SERPINA3 | 10 |
| GO:0043207 | response to external biotic stimulus | 10/11 | 337/749 | 0,002075115 | 0,015563364 | 0,013316022 | IGHG2/IGHG3/IGKC/IGHG4/IGHA1/IGHG1/IGLC1/IGHM/CD24/CIITA | 10 |
| GO:0051707 | response to other organism | 10/11 | 337/749 | 0,002075115 | 0,015563364 | 0,013316022 | IGHG2/IGHG3/IGKC/IGHG4/IGHA1/IGHG1/IGLC1/IGHM/CD24/CIITA | 10 |
| GO:0009607 | response to biotic stimulus | 10/11 | 338/749 | 0,00213398 | 0,015702872 | 0,013435386 | IGHG2/IGHG3/IGKC/IGHG4/IGHA1/IGHG1/IGLC1/IGHM/CD24/CIITA | 10 |
| GO:0098542 | defense response to other organism | 9/11 | 268/749 | 0,002276686 | 0,016442735 | 0,014068413 | IGHG2/IGHG3/IGKC/IGHG4/IGHA1/IGHG1/IGLC1/IGHM/CIITA | 9 |
| GO:0050776 | regulation of immune response | 9/11 | 269/749 | 0,002347188 | 0,016643696 | 0,014240355 | IGHG2/IGHG3/IGKC/IGHG4/IGHA1/IGHG1/IGLC1/IGHM/CD24 | 9 |
| GO:0002684 | positive regulation of immune system proce | 9/11 | 273/749 | 0,002648178 | 0,018442666 | 0,015779555 | IGHG2/IGHG3/IGKC/IGHG4/IGHA1/IGHG1/IGLC1/IGHM/CD24 | 9 |
| GO:0006810 | transport | 10/11 | 349/749 | 0,002885153 | 0,019740522 | 0,016890001 | IGHG2/IGHG3/IGKC/IGHG4/IGHA1/IGHG1/IGLC1/IGHM/CD24/SERPINA3 | 10 |
| GO:0051234 | establishment of localization | 10/11 | 351/749 | 0,003044206 | 0,02046966 | 0,017513852 | IGHG2/IGHG3/IGKC/IGHG4/IGHA1/IGHG1/IGLC1/IGHM/CD24/SERPINA3 | 10 |
| GO:0060249 | anatomical structure homeostasis | 4/11 | 47/749 | 0,00325311 | 0,02150361 | 0,0183985 | IGHG3/IGKC/IGHA1/SERPINA3 | 4 |
| GO:0006955 | immune response | 11/11 | 460/749 | 0,004474565 | 0,029084674 | 0,024884863 | IGHG2/IGHG3/IGKC/IGHG4/IGHA1/IGHG1/IGLC1/IGHM/CD24/SERPINA3/CIITA | 11 |
| GO:0044419 | interspecies interaction between organisms | 10/11 | 383/749 | 0,006864811 | 0,043889778 | 0,037552117 | IGHG2/IGHG3/IGKC/IGHG4/IGHA1/IGHG1/IGLC1/IGHM/CD24/CIITA | 10 |
| GO:0006952 | defense response | 10/11 | 386/749 | 0,007378112 | 0,046410704 | 0,039709023 | IGHG2/IGHG3/IGKC/IGHG4/IGHA1/IGHG1/IGLC1/IGHM/SERPINA3/CIITA | 10 |
| GO:0002697 | regulation of immune effector process | 6/11 | 141/749 | 0,008121511 | 0,05027602 | 0,04301619 | IGHG2/IGHG3/IGKC/IGHG4/IGHG1/IGLC1 | 6 |
| GO:0006950 | response to stress | 11/11 | 495/749 | 0,010111096 | 0,061614494 | 0,052717394 | IGHG2/IGHG3/IGKC/IGHG4/IGHA1/IGHG1/IGLC1/IGHM/CD24/SERPINA3/CIITA | 11 |
| GO:0048871 | multicellular organismal homeostasis | 4/11 | 66/749 | 0,011320887 | 0,06792532 | 0,05811694 | IGHG3/IGKC/IGHA1/SERPINA3 | 4 |
| GO:0016043 | cellular component organization | 9/11 | 329/749 | 0,01177829 | 0,069598984 | 0,059548929 | IGHG2/IGHG3/IGKC/IGHG4/IGHA1/IGHG1/IGLC1/IGHM/CD24 | 9 |
| GO:0071840 | cellular component organization or biogene | 9/11 | 331/749 | 0,012348979 | 0,071882114 | 0,061502376 | IGHG2/IGHG3/IGKC/IGHG4/IGHA1/IGHG1/IGLC1/IGHM/CD24 | 9 |
| GO:0006898 | receptor-mediated endocytosis | 3/11 | 40/749 | 0,017287262 | 0,099147532 | 0,084830682 | IGKC/IGHA1/IGLC1 | 3 |
| GO:0002682 | regulation of immune system process | 9/11 | 362/749 | 0,024587442 | 0,138167386 | 0,11821609 | IGHG2/IGHG3/IGKC/IGHG4/IGHA1/IGHG1/IGLC1/IGHM/CD24 | 9 |
| GO:0009605 | response to external stimulus | 10/11 | 441/749 | 0,024799274 | 0,138167386 | 0,11821609 | IGHG2/IGHG3/IGKC/IGHG4/IGHA1/IGHG1/IGLC1/IGHM/CD24/CIITA | 10 |
| GO:0051179 | localization | 10/11 | 448/749 | 0,028543121 | 0,156786158 | 0,134146322 | IGHG2/IGHG3/IGKC/IGHG4/IGHA1/IGHG1/IGLC1/IGHM/CD24/SERPINA3 | 10 |
| GO:0007166 | cell surface receptor signaling pathway | 10/11 | 465/749 | 0,039693046 | 0,215003997 | 0,183957536 | IGHG2/IGHG3/IGKC/IGHG4/IGHA1/IGHG1/IGLC1/IGHM/CD24/CIITA | 10 |
| GO:0048584 | positive regulation of response to stimulus | 9/11 | 387/749 | 0,040518931 | 0,216471001 | 0,185212706 | IGHG2/IGHG3/IGKC/IGHG4/IGHA1/IGHG1/IGLC1/IGHM/CD24 | 9 |
| GO:0019730 | antimicrobial humoral response | 2/11 | 26/749 | 0,052586929 | 0,277147329 | 0,237127404 | IGHA1/IGHM | 2 |
| GO:0038095 | Fc-epsilon receptor signaling pathway | 2/11 | 27/749 | 0,0563384 | 0,292959682 | 0,250656462 | IGKC/IGLC1 | 2 |
| GO:0050900 | leukocyte migration | 4/11 | 139/749 | 0,129147479 | 0,662730483 | 0,567032559 | IGKC/IGHA1/IGLC1/IGHM | 4 |
| GO:0042592 | homeostatic process | 5/11 | 197/749 | 0,13497851 | 0,674240635 | 0,576880651 | IGHG3/IGKC/IGHA1/CD24/SERPINA3 | 5 |
| GO:0010712 | regulation of collagen metabolic process | 1/11 | 10/749 | 0,138305771 | 0,674240635 | 0,576880651 | CIITA | 1 |
| GO:0042632 | cholesterol homeostasis | 1/11 | 10/749 | 0,138305771 | 0,674240635 | 0,576880651 | CD24 | 1 |
| GO:0055092 | sterol homeostasis | 1/11 | 10/749 | 0,138305771 | 0,674240635 | 0,576880651 | CD24 | 1 |
| GO:0030858 | positive regulation of epithelial cell differen | 1/11 | 11/749 | 0,151132072 | 0,710138653 | 0,607595015 | CD24 | 1 |
| GO:0032964 | collagen biosynthetic process | 1/11 | 11/749 | 0,151132072 | 0,710138653 | 0,607595015 | CIITA | 1 |
| GO:0055088 | lipid homeostasis | 1/11 | 11/749 | 0,151132072 | 0,710138653 | 0,607595015 | CD24 | 1 |
| GO:0043627 | response to estrogen | 1/11 | 13/749 | 0,176265403 | 0,818375084 | 0,700202163 | CD24 | 1 |
| GO:0061097 | regulation of protein tyrosine kinase activity | 1/11 | 14/749 | 0,188576653 | 0,855173196 | 0,731686648 | CD24 | 1 |
| GO:0090184 | positive regulation of kidney development | 1/11 | 14/749 | 0,188576653 | 0,855173196 | 0,731686648 | CD24 | 1 |
| GO:0003014 | renal system process | 1/11 | 15/749 | 0,200720404 | 0,864088504 | 0,739314591 | IGHA1 | 1 |
| GO:0035850 | epithelial cell differentiation involved in kid | 1/11 | 15/749 | 0,200720404 | 0,864088504 | 0,739314591 | CD24 | 1 |
| GO:0061005 | cell differentiation involved in kidney develo | 1/11 | 15/749 | 0,200720404 | 0,864088504 | 0,739314591 | CD24 | 1 |
| GO:0090150 | establishment of protein localization to mer | 1/11 | 15/749 | 0,200720404 | 0,864088504 | 0,739314591 | CD24 | 1 |
| GO:0048583 | regulation of response to stimulus | 9/11 | 490/749 | 0,206421386 | 0,864088504 | 0,739314591 | IGHG2/IGHG3/IGKC/IGHG4/IGHA1/IGHG1/IGLC1/IGHM/CD24 | 9 |
| GO:0006953 | acute-phase response | 1/11 | 16/749 | 0,212698709 | 0,864088504 | 0,739314591 | SERPINA3 | 1 |
| GO:0042104 | positive regulation of activated T cell prolife | 1/11 | 16/749 | 0,212698709 | 0,864088504 | 0,739314591 | CD24 | 1 |
| GO:0071604 | transforming growth factor beta productior | 1/11 | 16/749 | 0,212698709 | 0,864088504 | 0,739314591 | CD24 | 1 |
| GO:0071634 | regulation of transforming growth factor be | 1/11 | 16/749 | 0,212698709 | 0,864088504 | 0,739314591 | CD24 | 1 |
| GO:0072080 | nephron tubule development | 1/11 | 16/749 | 0,212698709 | 0,864088504 | 0,739314591 | CD24 | 1 |
| GO:0016477 | cell migration | 5/11 | 229/749 | 0,221675379 | 0,884447509 | 0,756733766 | IGKC/IGHA1/IGLC1/IGHM/CD24 | 5 |
| GO:0050829 | defense response to Gram-negative bacteri | 1/11 | 17/749 | 0,224513599 | 0,884447509 | 0,756733766 | IGHM | 1 |

| | | | | | | | | |
|---|---|---|---|---|---|---|---|---|
| GO:0061326 | renal tubule development | 1/11 | 17/749 | 0,224513599 | 0,884447509 | 0,756733766 | CD24 | 1 |
| GO:0090183 | regulation of kidney development | 1/11 | 18/749 | 0,236167083 | 0,906353859 | 0,775476851 | CD24 | 1 |
| GO:0048870 | cell motility | 5/11 | 234/749 | 0,237046394 | 0,906353859 | 0,775476851 | IGKC/IGHA1/IGLC1/IGHM/CD24 | 5 |
| GO:0051674 | localization of cell | 5/11 | 234/749 | 0,237046394 | 0,906353859 | 0,775476851 | IGKC/IGHA1/IGLC1/IGHM/CD24 | 5 |
| GO:0032963 | collagen metabolic process | 1/11 | 19/749 | 0,247661148 | 0,937746096 | 0,802336066 | CIITA | 1 |
| GO:0002576 | platelet degranulation | 1/11 | 20/749 | 0,258997761 | 0,953965388 | 0,816213301 | SERPINA3 | 1 |
| GO:0032835 | glomerulus development | 1/11 | 20/749 | 0,258997761 | 0,953965388 | 0,816213301 | CD24 | 1 |
| GO:0006928 | movement of cell or subcellular component | 5/11 | 241/749 | 0,2592829 | 0,953965388 | 0,816213301 | IGKC/IGHA1/IGLC1/IGHM/CD24 | 5 |
| GO:0040011 | locomotion | 5/11 | 244/749 | 0,269052737 | 0,980659507 | 0,839052804 | IGKC/IGHA1/IGLC1/IGHM/CD24 | 5 |
| GO:0046006 | regulation of activated T cell proliferation | 1/11 | 22/749 | 0,281206382 | 0,999976816 | 0,855580704 | CD24 | 1 |
| GO:0072009 | nephron epithelium development | 1/11 | 22/749 | 0,281206382 | 0,999976816 | 0,855580704 | CD24 | 1 |
| GO:0050798 | activated T cell proliferation | 1/11 | 23/749 | 0,292082214 | 0,999976816 | 0,855580704 | CD24 | 1 |
| GO:0030856 | regulation of epithelial cell differentiation | 1/11 | 27/749 | 0,334105987 | 0,999976816 | 0,855580704 | CD24 | 1 |
| GO:0031294 | lymphocyte costimulation | 1/11 | 28/749 | 0,344251187 | 0,999976816 | 0,855580704 | CD24 | 1 |
| GO:0031295 | T cell costimulation | 1/11 | 28/749 | 0,344251187 | 0,999976816 | 0,855580704 | CD24 | 1 |
| GO:0072073 | kidney epithelium development | 1/11 | 29/749 | 0,354255676 | 0,999976816 | 0,855580704 | CD24 | 1 |
| GO:0072006 | nephron development | 1/11 | 31/749 | 0,373849541 | 0,999976816 | 0,855580704 | CD24 | 1 |
| GO:0072657 | protein localization to membrane | 1/11 | 34/749 | 0,402228352 | 0,999976816 | 0,855580704 | CD24 | 1 |
| GO:0002526 | acute inflammatory response | 1/11 | 36/749 | 0,420492523 | 0,999976816 | 0,855580704 | SERPINA3 | 1 |
| GO:0010466 | negative regulation of peptidase activity | 1/11 | 36/749 | 0,420492523 | 0,999976816 | 0,855580704 | SERPINA3 | 1 |
| GO:0010951 | negative regulation of endopeptidase activi | 1/11 | 36/749 | 0,420492523 | 0,999976816 | 0,855580704 | SERPINA3 | 1 |
| GO:0097193 | intrinsic apoptotic signaling pathway | 1/11 | 37/749 | 0,429433031 | 0,999976816 | 0,855580704 | CD24 | 1 |
| GO:0002377 | immunoglobulin production | 1/11 | 40/749 | 0,455507482 | 0,999976816 | 0,855580704 | IGKC | 1 |
| GO:0045861 | negative regulation of proteolysis | 1/11 | 43/749 | 0,480494141 | 0,999976816 | 0,855580704 | SERPINA3 | 1 |
| GO:0065008 | regulation of biological quality | 5/11 | 304/749 | 0,483538474 | 0,999976816 | 0,855580704 | IGHG3/IGKC/IGHA1/CD24/SERPINA3 | 5 |
| GO:0060333 | interferon-gamma-mediated signaling pathv | 1/11 | 44/749 | 0,488588425 | 0,999976816 | 0,855580704 | CIITA | 1 |
| GO:0006886 | intracellular protein transport | 1/11 | 45/749 | 0,496567896 | 0,999976816 | 0,855580704 | CD24 | 1 |
| GO:0016055 | Wnt signaling pathway | 1/11 | 45/749 | 0,496567896 | 0,999976816 | 0,855580704 | CD24 | 1 |
| GO:0198738 | cell-cell signaling by wnt | 1/11 | 45/749 | 0,496567896 | 0,999976816 | 0,855580704 | CD24 | 1 |
| GO:0019216 | regulation of lipid metabolic process | 1/11 | 47/749 | 0,512188256 | 0,999976816 | 0,855580704 | SERPINA3 | 1 |
| GO:0001959 | regulation of cytokine-mediated signaling p | 1/11 | 48/749 | 0,519832029 | 0,999976816 | 0,855580704 | CD24 | 1 |
| GO:0051346 | negative regulation of hydrolase activity | 1/11 | 50/749 | 0,534793856 | 0,999976816 | 0,855580704 | SERPINA3 | 1 |
| GO:0043406 | positive regulation of MAP kinase activity | 1/11 | 51/749 | 0,542114697 | 0,999976816 | 0,855580704 | CD24 | 1 |
| GO:0060759 | regulation of response to cytokine stimulus | 1/11 | 51/749 | 0,542114697 | 0,999976816 | 0,855580704 | CD24 | 1 |
| GO:1905114 | cell surface receptor signaling pathway invc | 1/11 | 51/749 | 0,542114697 | 0,999976816 | 0,855580704 | CD24 | 1 |
| GO:0001822 | kidney development | 1/11 | 52/749 | 0,549330654 | 0,999976816 | 0,855580704 | CD24 | 1 |
| GO:0072001 | renal system development | 1/11 | 53/749 | 0,556443083 | 0,999976816 | 0,855580704 | CD24 | 1 |
| GO:0007204 | positive regulation of cytosolic calcium ion c | 1/11 | 54/749 | 0,563453321 | 0,999976816 | 0,855580704 | CD24 | 1 |
| GO:0071902 | positive regulation of protein serine/threon | 1/11 | 55/749 | 0,570362693 | 0,999976816 | 0,855580704 | CD24 | 1 |
| GO:0001655 | urogenital system development | 1/11 | 56/749 | 0,577172506 | 0,999976816 | 0,855580704 | CD24 | 1 |
| GO:0051480 | regulation of cytosolic calcium ion concentr | 1/11 | 56/749 | 0,577172506 | 0,999976816 | 0,855580704 | CD24 | 1 |
| GO:0003008 | system process | 2/11 | 129/749 | 0,590497844 | 0,999976816 | 0,855580704 | IGHA1/SERPINA3 | 2 |
| GO:0050731 | positive regulation of peptidyl-tyrosine pho: | 1/11 | 59/749 | 0,597017449 | 0,999976816 | 0,855580704 | CD24 | 1 |
| GO:0046677 | response to antibiotic | 1/11 | 60/749 | 0,603441809 | 0,999976816 | 0,855580704 | CIITA | 1 |
| GO:0042102 | positive regulation of T cell proliferation | 1/11 | 61/749 | 0,609772926 | 0,999976816 | 0,855580704 | CD24 | 1 |
| GO:0043405 | regulation of MAP kinase activity | 1/11 | 61/749 | 0,609772926 | 0,999976816 | 0,855580704 | CD24 | 1 |
| GO:0001666 | response to hypoxia | 1/11 | 63/749 | 0,622160301 | 0,999976816 | 0,855580704 | CD24 | 1 |
| GO:0036293 | response to decreased oxygen levels | 1/11 | 63/749 | 0,622160301 | 0,999976816 | 0,855580704 | CD24 | 1 |
| GO:0019221 | cytokine-mediated signaling pathway | 4/11 | 275/749 | 0,622271521 | 0,999976816 | 0,855580704 | IGHG4/IGHG1/CD24/CIITA | 4 |
| GO:0006874 | cellular calcium ion homeostasis | 1/11 | 65/749 | 0,634189162 | 0,999976816 | 0,855580704 | CD24 | 1 |

| | | | | | | | | |
|---|---|---|---|---|---|---|---|---|
| GO:0055074 | calcium ion homeostasis | 1/11 | 65/749 | 0,634189162 | 0,999976816 | 0,855580704 | CD24 | 1 |
| GO:0070482 | response to oxygen levels | 1/11 | 68/749 | 0,651580666 | 0,999976816 | 0,855580704 | CD24 | 1 |
| GO:0002283 | neutrophil activation involved in immune re | 1/11 | 69/749 | 0,657208585 | 0,999976816 | 0,855580704 | SERPINA3 | 1 |
| GO:0043312 | neutrophil degranulation | 1/11 | 69/749 | 0,657208585 | 0,999976816 | 0,855580704 | SERPINA3 | 1 |
| GO:0046907 | intracellular transport | 1/11 | 69/749 | 0,657208585 | 0,999976816 | 0,855580704 | CD24 | 1 |
| GO:0052548 | regulation of endopeptidase activity | 1/11 | 69/749 | 0,657208585 | 0,999976816 | 0,855580704 | SERPINA3 | 1 |
| GO:0002446 | neutrophil mediated immunity | 1/11 | 70/749 | 0,66275374 | 0,999976816 | 0,855580704 | SERPINA3 | 1 |
| GO:0050730 | regulation of peptidyl-tyrosine phosphoryla | 1/11 | 70/749 | 0,66275374 | 0,999976816 | 0,855580704 | CD24 | 1 |
| GO:0071346 | cellular response to interferon-gamma | 1/11 | 70/749 | 0,66275374 | 0,999976816 | 0,855580704 | CIITA | 1 |
| GO:0072503 | cellular divalent inorganic cation homeostas | 1/11 | 70/749 | 0,66275374 | 0,999976816 | 0,855580704 | CD24 | 1 |
| GO:0071900 | regulation of protein serine/threonine kinas | 1/11 | 71/749 | 0,668217229 | 0,999976816 | 0,855580704 | CD24 | 1 |
| GO:0072507 | divalent inorganic cation homeostasis | 1/11 | 71/749 | 0,668217229 | 0,999976816 | 0,855580704 | CD24 | 1 |
| GO:0030855 | epithelial cell differentiation | 1/11 | 72/749 | 0,673600135 | 0,999976816 | 0,855580704 | CD24 | 1 |
| GO:0000122 | negative regulation of transcription by RNA | 1/11 | 74/749 | 0,684128473 | 0,999976816 | 0,855580704 | CIITA | 1 |
| GO:0042119 | neutrophil activation | 1/11 | 75/749 | 0,689276009 | 0,999976816 | 0,855580704 | SERPINA3 | 1 |
| GO:0052547 | regulation of peptidase activity | 1/11 | 75/749 | 0,689276009 | 0,999976816 | 0,855580704 | SERPINA3 | 1 |
| GO:0006629 | lipid metabolic process | 1/11 | 76/749 | 0,694347172 | 0,999976816 | 0,855580704 | SERPINA3 | 1 |
| GO:0050671 | positive regulation of lymphocyte proliferat | 1/11 | 76/749 | 0,694347172 | 0,999976816 | 0,855580704 | CD24 | 1 |
| GO:0002440 | production of molecular mediator of immur | 1/11 | 77/749 | 0,699342984 | 0,999976816 | 0,855580704 | IGKC | 1 |
| GO:0032946 | positive regulation of mononuclear cell prol | 1/11 | 77/749 | 0,699342984 | 0,999976816 | 0,855580704 | CD24 | 1 |
| GO:0034341 | response to interferon-gamma | 1/11 | 77/749 | 0,699342984 | 0,999976816 | 0,855580704 | CIITA | 1 |
| GO:0036230 | granulocyte activation | 1/11 | 77/749 | 0,699342984 | 0,999976816 | 0,855580704 | SERPINA3 | 1 |
| GO:0006875 | cellular metal ion homeostasis | 1/11 | 79/749 | 0,709112576 | 0,999976816 | 0,855580704 | CD24 | 1 |
| GO:0070665 | positive regulation of leukocyte proliferatio | 1/11 | 79/749 | 0,709112576 | 0,999976816 | 0,855580704 | CD24 | 1 |
| GO:0002275 | myeloid cell activation involved in immune r | 1/11 | 81/749 | 0,718592717 | 0,999976816 | 0,855580704 | SERPINA3 | 1 |
| GO:0043299 | leukocyte degranulation | 1/11 | 81/749 | 0,718592717 | 0,999976816 | 0,855580704 | SERPINA3 | 1 |
| GO:0002444 | myeloid leukocyte mediated immunity | 1/11 | 82/749 | 0,723226669 | 0,999976816 | 0,855580704 | SERPINA3 | 1 |
| GO:0030003 | cellular cation homeostasis | 1/11 | 82/749 | 0,723226669 | 0,999976816 | 0,855580704 | CD24 | 1 |
| GO:0045860 | positive regulation of protein kinase activity | 1/11 | 82/749 | 0,723226669 | 0,999976816 | 0,855580704 | CD24 | 1 |
| GO:0006873 | cellular ion homeostasis | 1/11 | 83/749 | 0,727791147 | 0,999976816 | 0,855580704 | CD24 | 1 |
| GO:0042129 | regulation of T cell proliferation | 1/11 | 84/749 | 0,732287089 | 0,999976816 | 0,855580704 | CD24 | 1 |
| GO:0034613 | cellular protein localization | 1/11 | 85/749 | 0,736715423 | 0,999976816 | 0,855580704 | CD24 | 1 |
| GO:0043086 | negative regulation of catalytic activity | 1/11 | 85/749 | 0,736715423 | 0,999976816 | 0,855580704 | SERPINA3 | 1 |
| GO:0070727 | cellular macromolecule localization | 1/11 | 85/749 | 0,736715423 | 0,999976816 | 0,855580704 | CD24 | 1 |
| GO:0033674 | positive regulation of kinase activity | 1/11 | 86/749 | 0,741077065 | 0,999976816 | 0,855580704 | CD24 | 1 |
| GO:0055065 | metal ion homeostasis | 1/11 | 86/749 | 0,741077065 | 0,999976816 | 0,855580704 | CD24 | 1 |
| GO:0001818 | negative regulation of cytokine production | 1/11 | 87/749 | 0,745372921 | 0,999976816 | 0,855580704 | CD24 | 1 |
| GO:0018108 | peptidyl-tyrosine phosphorylation | 1/11 | 87/749 | 0,745372921 | 0,999976816 | 0,855580704 | CD24 | 1 |
| GO:0018212 | peptidyl-tyrosine modification | 1/11 | 87/749 | 0,745372921 | 0,999976816 | 0,855580704 | CD24 | 1 |
| GO:0071345 | cellular response to cytokine stimulus | 4/11 | 314/749 | 0,749048498 | 0,999976816 | 0,855580704 | IGHG4/IGHG1/CD24/CIITA | 4 |
| GO:0055080 | cation homeostasis | 1/11 | 89/749 | 0,753770839 | 0,999976816 | 0,855580704 | CD24 | 1 |
| GO:0098771 | inorganic ion homeostasis | 1/11 | 90/749 | 0,757874659 | 0,999976816 | 0,855580704 | CD24 | 1 |
| GO:0010605 | negative regulation of macromolecule meta | 3/11 | 248/749 | 0,76299972 | 0,999976816 | 0,855580704 | CD24/SERPINA3/CIITA | 3 |
| GO:0051649 | establishment of localization in cell | 2/11 | 174/749 | 0,765836699 | 0,999976816 | 0,855580704 | CD24/SERPINA3 | 2 |
| GO:0045892 | negative regulation of transcription, DNA-te | 1/11 | 92/749 | 0,765896329 | 0,999976816 | 0,855580704 | CIITA | 1 |
| GO:0051347 | positive regulation of transferase activity | 1/11 | 92/749 | 0,765896329 | 0,999976816 | 0,855580704 | CD24 | 1 |
| GO:0042098 | T cell proliferation | 1/11 | 93/749 | 0,769815873 | 0,999976816 | 0,855580704 | CD24 | 1 |
| GO:1903507 | negative regulation of nucleic acid-template | 1/11 | 93/749 | 0,769815873 | 0,999976816 | 0,855580704 | CIITA | 1 |
| GO:0055082 | cellular chemical homeostasis | 1/11 | 94/749 | 0,773675667 | 0,999976816 | 0,855580704 | CD24 | 1 |
| GO:1902679 | negative regulation of RNA biosynthetic pro | 1/11 | 94/749 | 0,773675667 | 0,999976816 | 0,855580704 | CIITA | 1 |

| | | | | | | | | |
|---|---|---|---|---|---|---|---|---|
| GO:0030162 | regulation of proteolysis | 1/11 | 95/749 | 0,777476534 | 0,999976816 | 0,855580704 | SERPINA3 | 1 |
| GO:0043410 | positive regulation of MAPK cascade | 1/11 | 95/749 | 0,777476534 | 0,999976816 | 0,855580704 | CD24 | 1 |
| GO:2000113 | negative regulation of cellular macromolecu | 1/11 | 96/749 | 0,781219284 | 0,999976816 | 0,855580704 | CIITA | 1 |
| GO:0050801 | ion homeostasis | 1/11 | 97/749 | 0,784904717 | 0,999976816 | 0,855580704 | CD24 | 1 |
| GO:0034097 | response to cytokine | 4/11 | 328/749 | 0,787765199 | 0,999976816 | 0,855580704 | IGHG4/IGHG1/CD24/CIITA | 4 |
| GO:0045055 | regulated exocytosis | 1/11 | 99/749 | 0,792106789 | 0,999976816 | 0,855580704 | SERPINA3 | 1 |
| GO:0009892 | negative regulation of metabolic process | 3/11 | 259/749 | 0,793578614 | 0,999976816 | 0,855580704 | CD24/SERPINA3/CIITA | 3 |
| GO:0051253 | negative regulation of RNA metabolic proce | 1/11 | 100/749 | 0,795624982 | 0,999976816 | 0,855580704 | CIITA | 1 |
| GO:0019725 | cellular homeostasis | 1/11 | 101/749 | 0,799088966 | 0,999976816 | 0,855580704 | CD24 | 1 |
| GO:0032269 | negative regulation of cellular protein meta | 1/11 | 102/749 | 0,802499492 | 0,999976816 | 0,855580704 | SERPINA3 | 1 |
| GO:0050870 | positive regulation of T cell activation | 1/11 | 102/749 | 0,802499492 | 0,999976816 | 0,855580704 | CD24 | 1 |
| GO:0006887 | exocytosis | 1/11 | 104/749 | 0,809163142 | 0,999976816 | 0,855580704 | SERPINA3 | 1 |
| GO:0045934 | negative regulation of nucleobase-containir | 1/11 | 104/749 | 0,809163142 | 0,999976816 | 0,855580704 | CIITA | 1 |
| GO:0045859 | regulation of protein kinase activity | 1/11 | 105/749 | 0,812417724 | 0,999976816 | 0,855580704 | CD24 | 1 |
| GO:0051172 | negative regulation of nitrogen compound r | 2/11 | 190/749 | 0,81244118 | 0,999976816 | 0,855580704 | SERPINA3/CIITA | 2 |
| GO:0010558 | negative regulation of macromolecule biosy | 1/11 | 107/749 | 0,818775984 | 0,999976816 | 0,855580704 | CIITA | 1 |
| GO:0050670 | regulation of lymphocyte proliferation | 1/11 | 107/749 | 0,818775984 | 0,999976816 | 0,855580704 | CD24 | 1 |
| GO:0097190 | apoptotic signaling pathway | 1/11 | 107/749 | 0,818775984 | 0,999976816 | 0,855580704 | CD24 | 1 |
| GO:0032944 | regulation of mononuclear cell proliferation | 1/11 | 108/749 | 0,821881069 | 0,999976816 | 0,855580704 | CD24 | 1 |
| GO:1903039 | positive regulation of leukocyte cell-cell adh | 1/11 | 108/749 | 0,821881069 | 0,999976816 | 0,855580704 | CD24 | 1 |
| GO:0010629 | negative regulation of gene expression | 2/11 | 195/749 | 0,825438164 | 0,999976816 | 0,855580704 | CD24/CIITA | 2 |
| GO:0031327 | negative regulation of cellular biosynthetic | 1/11 | 110/749 | 0,827946595 | 0,999976816 | 0,855580704 | CIITA | 1 |
| GO:0031324 | negative regulation of cellular metabolic pr | 2/11 | 196/749 | 0,827951648 | 0,999976816 | 0,855580704 | SERPINA3/CIITA | 2 |
| GO:0070663 | regulation of leukocyte proliferation | 1/11 | 111/749 | 0,830908391 | 0,999976816 | 0,855580704 | CD24 | 1 |
| GO:0009890 | negative regulation of biosynthetic process | 1/11 | 113/749 | 0,836693369 | 0,999976816 | 0,855580704 | CIITA | 1 |
| GO:0043549 | regulation of kinase activity | 1/11 | 113/749 | 0,836693369 | 0,999976816 | 0,855580704 | CD24 | 1 |
| GO:0022409 | positive regulation of cell-cell adhesion | 1/11 | 114/749 | 0,839517854 | 0,999976816 | 0,855580704 | CD24 | 1 |
| GO:0002237 | response to molecule of bacterial origin | 1/11 | 115/749 | 0,84229786 | 0,999976816 | 0,855580704 | CD24 | 1 |
| GO:0051641 | cellular localization | 2/11 | 203/749 | 0,844765828 | 0,999976816 | 0,855580704 | CD24/SERPINA3 | 2 |
| GO:0043408 | regulation of MAPK cascade | 1/11 | 117/749 | 0,847726949 | 0,999976816 | 0,855580704 | CD24 | 1 |
| GO:0051248 | negative regulation of protein metabolic pr | 1/11 | 118/749 | 0,850377271 | 0,999976816 | 0,855580704 | SERPINA3 | 1 |
| GO:0051338 | regulation of transferase activity | 1/11 | 119/749 | 0,852985591 | 0,999976816 | 0,855580704 | CD24 | 1 |
| GO:0044092 | negative regulation of molecular function | 1/11 | 120/749 | 0,855552509 | 0,999976816 | 0,855580704 | SERPINA3 | 1 |
| GO:0060429 | epithelium development | 1/11 | 120/749 | 0,855552509 | 0,999976816 | 0,855580704 | CD24 | 1 |
| GO:0002274 | myeloid leukocyte activation | 1/11 | 121/749 | 0,858078618 | 0,999976816 | 0,855580704 | SERPINA3 | 1 |
| GO:0048878 | chemical homeostasis | 1/11 | 125/749 | 0,867786543 | 0,999976816 | 0,855580704 | CD24 | 1 |
| GO:0046651 | lymphocyte proliferation | 1/11 | 127/749 | 0,872410505 | 0,999976816 | 0,855580704 | CD24 | 1 |
| GO:0006954 | inflammatory response | 2/11 | 217/749 | 0,874499678 | 0,999976816 | 0,855580704 | SERPINA3/CIITA | 2 |
| GO:0032943 | mononuclear cell proliferation | 1/11 | 128/749 | 0,87466691 | 0,999976816 | 0,855580704 | CD24 | 1 |
| GO:0051336 | regulation of hydrolase activity | 1/11 | 129/749 | 0,876886981 | 0,999976816 | 0,855580704 | SERPINA3 | 1 |
| GO:1903037 | regulation of leukocyte cell-cell adhesion | 1/11 | 129/749 | 0,876886981 | 0,999976816 | 0,855580704 | CD24 | 1 |
| GO:0050790 | regulation of catalytic activity | 2/11 | 221/749 | 0,882099099 | 0,999976816 | 0,855580704 | CD24/SERPINA3 | 2 |
| GO:0045785 | positive regulation of cell adhesion | 1/11 | 132/749 | 0,883334424 | 0,999976816 | 0,855580704 | CD24 | 1 |
| GO:0018193 | peptidyl-amino acid modification | 1/11 | 133/749 | 0,885414361 | 0,999976816 | 0,855580704 | CD24 | 1 |
| GO:0050863 | regulation of T cell activation | 1/11 | 134/749 | 0,887460533 | 0,999976816 | 0,855580704 | CD24 | 1 |
| GO:0070661 | leukocyte proliferation | 1/11 | 134/749 | 0,887460533 | 0,999976816 | 0,855580704 | CD24 | 1 |
| GO:0006508 | proteolysis | 1/11 | 136/749 | 0,891453552 | 0,999976816 | 0,855580704 | SERPINA3 | 1 |
| GO:0007159 | leukocyte cell-cell adhesion | 1/11 | 139/749 | 0,897201986 | 0,999976816 | 0,855580704 | CD24 | 1 |
| GO:0009628 | response to abiotic stimulus | 1/11 | 141/749 | 0,900879017 | 0,999976816 | 0,855580704 | CD24 | 1 |
| GO:0045944 | positive regulation of transcription by RNA | 1/11 | 141/749 | 0,900879017 | 0,999976816 | 0,855580704 | CIITA | 1 |

| | | | | | | | | |
|---|---|---|---|---|---|---|---|---|
| GO:0002263 | cell activation involved in immune response | 1/11 | 143/749 | 0,904436087 | 0,999976816 | 0,855580704 | SERPINA3 | 1 |
| GO:0002366 | leukocyte activation involved in immune res | 1/11 | 143/749 | 0,904436087 | 0,999976816 | 0,855580704 | SERPINA3 | 1 |
| GO:0022407 | regulation of cell-cell adhesion | 1/11 | 143/749 | 0,904436087 | 0,999976816 | 0,855580704 | CD24 | 1 |
| GO:0000165 | MAPK cascade | 1/11 | 149/749 | 0,914422418 | 0,999976816 | 0,855580704 | CD24 | 1 |
| GO:0023014 | signal transduction by protein phosphorylat | 1/11 | 149/749 | 0,914422418 | 0,999976816 | 0,855580704 | CD24 | 1 |
| GO:0016310 | phosphorylation | 2/11 | 246/749 | 0,921596647 | 0,999976816 | 0,855580704 | CD24/CIITA | 2 |
| GO:0035295 | tube development | 1/11 | 154/749 | 0,922009021 | 0,999976816 | 0,855580704 | CD24 | 1 |
| GO:0045597 | positive regulation of cell differentiation | 1/11 | 154/749 | 0,922009021 | 0,999976816 | 0,855580704 | CD24 | 1 |
| GO:0043085 | positive regulation of catalytic activity | 1/11 | 157/749 | 0,926262119 | 0,999976816 | 0,855580704 | CD24 | 1 |
| GO:0015031 | protein transport | 1/11 | 160/749 | 0,930303312 | 0,999976816 | 0,855580704 | CD24 | 1 |
| GO:0001934 | positive regulation of protein phosphorylati | 1/11 | 162/749 | 0,932884447 | 0,999976816 | 0,855580704 | CD24 | 1 |
| GO:0015833 | peptide transport | 1/11 | 164/749 | 0,935378389 | 0,999976816 | 0,855580704 | CD24 | 1 |
| GO:0045184 | establishment of protein localization | 1/11 | 164/749 | 0,935378389 | 0,999976816 | 0,855580704 | CD24 | 1 |
| GO:0045893 | positive regulation of transcription, DNA-te | 1/11 | 164/749 | 0,935378389 | 0,999976816 | 0,855580704 | CIITA | 1 |
| GO:0042327 | positive regulation of phosphorylation | 1/11 | 165/749 | 0,936593496 | 0,999976816 | 0,855580704 | CD24 | 1 |
| GO:1902680 | positive regulation of RNA biosynthetic pro | 1/11 | 165/749 | 0,936593496 | 0,999976816 | 0,855580704 | CIITA | 1 |
| GO:1903508 | positive regulation of nucleic acid-template | 1/11 | 165/749 | 0,936593496 | 0,999976816 | 0,855580704 | CIITA | 1 |
| GO:0042886 | amide transport | 1/11 | 166/749 | 0,937787796 | 0,999976816 | 0,855580704 | CD24 | 1 |
| GO:0051254 | positive regulation of RNA metabolic proces | 1/11 | 168/749 | 0,940115258 | 0,999976816 | 0,855580704 | CIITA | 1 |
| GO:1902533 | positive regulation of intracellular signal tra | 1/11 | 169/749 | 0,941249048 | 0,999976816 | 0,855580704 | CD24 | 1 |
| GO:0008284 | positive regulation of cell population prolife | 1/11 | 173/749 | 0,945591746 | 0,999976816 | 0,855580704 | CD24 | 1 |
| GO:0010562 | positive regulation of phosphorus metaboli | 1/11 | 173/749 | 0,945591746 | 0,999976816 | 0,855580704 | CD24 | 1 |
| GO:0031401 | positive regulation of protein modification | 1/11 | 173/749 | 0,945591746 | 0,999976816 | 0,855580704 | CD24 | 1 |
| GO:0045937 | positive regulation of phosphate metabolic | 1/11 | 173/749 | 0,945591746 | 0,999976816 | 0,855580704 | CD24 | 1 |
| GO:0044267 | cellular protein metabolic process | 3/11 | 347/749 | 0,946487245 | 0,999976816 | 0,855580704 | IGHA1/CD24/SERPINA3 | 3 |
| GO:0032268 | regulation of cellular protein metabolic pro | 2/11 | 268/749 | 0,946643159 | 0,999976816 | 0,855580704 | CD24/SERPINA3 | 2 |
| GO:0071310 | cellular response to organic substance | 4/11 | 418/749 | 0,946702722 | 0,999976816 | 0,855580704 | IGHG4/IGHG1/CD24/CIITA | 4 |
| GO:0006357 | regulation of transcription by RNA polymera | 1/11 | 176/749 | 0,948654958 | 0,999976816 | 0,855580704 | CIITA | 1 |
| GO:0098609 | cell-cell adhesion | 1/11 | 176/749 | 0,948654958 | 0,999976816 | 0,855580704 | CD24 | 1 |
| GO:0006796 | phosphate-containing compound metabolic | 2/11 | 272/749 | 0,950383137 | 0,999976816 | 0,855580704 | CD24/CIITA | 2 |
| GO:0006793 | phosphorus metabolic process | 2/11 | 273/749 | 0,951282887 | 0,999976816 | 0,855580704 | CD24/CIITA | 2 |
| GO:0009888 | tissue development | 1/11 | 179/749 | 0,951560577 | 0,999976816 | 0,855580704 | CD24 | 1 |
| GO:0030155 | regulation of cell adhesion | 1/11 | 179/749 | 0,951560577 | 0,999976816 | 0,855580704 | CD24 | 1 |
| GO:0042110 | T cell activation | 1/11 | 179/749 | 0,951560577 | 0,999976816 | 0,855580704 | CD24 | 1 |
| GO:0045935 | positive regulation of nucleobase-containin | 1/11 | 179/749 | 0,951560577 | 0,999976816 | 0,855580704 | CIITA | 1 |
| GO:0071705 | nitrogen compound transport | 1/11 | 180/749 | 0,952495373 | 0,999976816 | 0,855580704 | CD24 | 1 |
| GO:0065009 | regulation of molecular function | 2/11 | 275/749 | 0,953041251 | 0,999976816 | 0,855580704 | CD24/SERPINA3 | 2 |
| GO:0006366 | transcription by RNA polymerase II | 1/11 | 182/749 | 0,954315939 | 0,999976816 | 0,855580704 | CIITA | 1 |
| GO:0051241 | negative regulation of multicellular organis | 1/11 | 186/749 | 0,957768128 | 0,999976816 | 0,855580704 | CD24 | 1 |
| GO:0001932 | regulation of protein phosphorylation | 1/11 | 193/749 | 0,963243871 | 0,999976816 | 0,855580704 | CD24 | 1 |
| GO:0008104 | protein localization | 1/11 | 195/749 | 0,964685147 | 0,999976816 | 0,855580704 | CD24 | 1 |
| GO:0010557 | positive regulation of macromolecule biosy | 1/11 | 195/749 | 0,964685147 | 0,999976816 | 0,855580704 | CIITA | 1 |
| GO:0006464 | cellular protein modification process | 2/11 | 292/749 | 0,965943375 | 0,999976816 | 0,855580704 | IGHA1/CD24 | 2 |
| GO:0036211 | protein modification process | 2/11 | 292/749 | 0,965943375 | 0,999976816 | 0,855580704 | IGHA1/CD24 | 2 |
| GO:0044093 | positive regulation of molecular function | 1/11 | 197/749 | 0,966074862 | 0,999976816 | 0,855580704 | CD24 | 1 |
| GO:0051246 | regulation of protein metabolic process | 2/11 | 293/749 | 0,966597557 | 0,999976816 | 0,855580704 | CD24/SERPINA3 | 2 |
| GO:0071702 | organic substance transport | 1/11 | 199/749 | 0,967414682 | 0,999976816 | 0,855580704 | CD24 | 1 |
| GO:0043412 | macromolecule modification | 2/11 | 295/749 | 0,967873915 | 0,999976816 | 0,855580704 | IGHA1/CD24 | 2 |
| GO:0042325 | regulation of phosphorylation | 1/11 | 200/749 | 0,968066388 | 0,999976816 | 0,855580704 | CD24 | 1 |
| GO:0051094 | positive regulation of developmental proces | 1/11 | 202/749 | 0,969334383 | 0,999976816 | 0,855580704 | CD24 | 1 |

| | | | | | | | | |
|---|---|---|---|---|---|---|---|---|
| GO:0044260 | cellular macromolecule metabolic process | 4/11 | 446/749 | 0,969510695 | 0,999976816 | 0,855580704 | IGHA1/CD24/SERPINA3/CIITA | 4 |
| GO:0032940 | secretion by cell | 1/11 | 204/749 | 0,970556441 | 0,999976816 | 0,855580704 | SERPINA3 | 1 |
| GO:0140352 | export from cell | 1/11 | 204/749 | 0,970556441 | 0,999976816 | 0,855580704 | SERPINA3 | 1 |
| GO:0070887 | cellular response to chemical stimulus | 4/11 | 448/749 | 0,970785483 | 0,999976816 | 0,855580704 | IGHG4/IGHG1/CD24/CIITA | 4 |
| GO:0031328 | positive regulation of cellular biosynthetic p | 1/11 | 205/749 | 0,971150715 | 0,999976816 | 0,855580704 | CIITA | 1 |
| GO:0009891 | positive regulation of biosynthetic process | 1/11 | 208/749 | 0,972868712 | 0,999976816 | 0,855580704 | CIITA | 1 |
| GO:0019220 | regulation of phosphate metabolic process | 1/11 | 209/749 | 0,973420365 | 0,999976816 | 0,855580704 | CD24 | 1 |
| GO:0051174 | regulation of phosphorus metabolic process | 1/11 | 209/749 | 0,973420365 | 0,999976816 | 0,855580704 | CD24 | 1 |
| GO:0010033 | response to organic substance | 4/11 | 453/749 | 0,973790111 | 0,999976816 | 0,855580704 | IGHG4/IGHG1/CD24/CIITA | 4 |
| GO:0007267 | cell-cell signaling | 1/11 | 210/749 | 0,973961802 | 0,999976816 | 0,855580704 | CD24 | 1 |
| GO:0001817 | regulation of cytokine production | 1/11 | 211/749 | 0,974493194 | 0,999976816 | 0,855580704 | CD24 | 1 |
| GO:0031399 | regulation of protein modification process | 1/11 | 211/749 | 0,974493194 | 0,999976816 | 0,855580704 | CD24 | 1 |
| GO:0019538 | protein metabolic process | 3/11 | 386/749 | 0,974893475 | 0,999976816 | 0,855580704 | IGHA1/CD24/SERPINA3 | 3 |
| GO:0033036 | macromolecule localization | 1/11 | 212/749 | 0,975014709 | 0,999976816 | 0,855580704 | CD24 | 1 |
| GO:0046903 | secretion | 1/11 | 214/749 | 0,976028766 | 0,999976816 | 0,855580704 | SERPINA3 | 1 |
| GO:1902531 | regulation of intracellular signal transductio | 1/11 | 216/749 | 0,977005269 | 0,999976816 | 0,855580704 | CD24 | 1 |
| GO:0032270 | positive regulation of cellular protein metab | 1/11 | 217/749 | 0,977479832 | 0,999976816 | 0,855580704 | CD24 | 1 |
| GO:0009893 | positive regulation of metabolic process | 3/11 | 395/749 | 0,97925651 | 0,999976816 | 0,855580704 | IGHA1/CD24/CIITA | 3 |
| GO:0045595 | regulation of cell differentiation | 1/11 | 226/749 | 0,981367154 | 0,999976816 | 0,855580704 | CD24 | 1 |
| GO:0019222 | regulation of metabolic process | 4/11 | 468/749 | 0,98136873 | 0,999976816 | 0,855580704 | IGHA1/CD24/SERPINA3/CIITA | 4 |
| GO:0051171 | regulation of nitrogen compound metabolic | 3/11 | 400/749 | 0,981396791 | 0,999976816 | 0,855580704 | CD24/SERPINA3/CIITA | 3 |
| GO:0001816 | cytokine production | 1/11 | 229/749 | 0,982520447 | 0,999976816 | 0,855580704 | CD24 | 1 |
| GO:0080090 | regulation of primary metabolic process | 3/11 | 404/749 | 0,982974544 | 0,999976816 | 0,855580704 | CD24/SERPINA3/CIITA | 3 |
| GO:0051173 | positive regulation of nitrogen compound m | 2/11 | 329/749 | 0,984021182 | 0,999976816 | 0,855580704 | CD24/CIITA | 2 |
| GO:0007155 | cell adhesion | 1/11 | 234/749 | 0,98429923 | 0,999976816 | 0,855580704 | CD24 | 1 |
| GO:0006355 | regulation of transcription, DNA-templated | 1/11 | 235/749 | 0,984634586 | 0,999976816 | 0,855580704 | CIITA | 1 |
| GO:0006468 | protein phosphorylation | 1/11 | 235/749 | 0,984634586 | 0,999976816 | 0,855580704 | CD24 | 1 |
| GO:0022610 | biological adhesion | 1/11 | 235/749 | 0,984634586 | 0,999976816 | 0,855580704 | CD24 | 1 |
| GO:1903506 | regulation of nucleic acid-templated transcr | 1/11 | 235/749 | 0,984634586 | 0,999976816 | 0,855580704 | CIITA | 1 |
| GO:0051247 | positive regulation of protein metabolic pro | 1/11 | 236/749 | 0,984963418 | 0,999976816 | 0,855580704 | CD24 | 1 |
| GO:2001141 | regulation of RNA biosynthetic process | 1/11 | 237/749 | 0,98528584 | 0,999976816 | 0,855580704 | CIITA | 1 |
| GO:0006351 | transcription, DNA-templated | 1/11 | 238/749 | 0,985601964 | 0,999976816 | 0,855580704 | CIITA | 1 |
| GO:0097659 | nucleic acid-templated transcription | 1/11 | 238/749 | 0,985601964 | 0,999976816 | 0,855580704 | CIITA | 1 |
| GO:0031323 | regulation of cellular metabolic process | 3/11 | 412/749 | 0,985798728 | 0,999976816 | 0,855580704 | CD24/SERPINA3/CIITA | 3 |
| GO:0051252 | regulation of RNA metabolic process | 1/11 | 239/749 | 0,985911902 | 0,999976816 | 0,855580704 | CIITA | 1 |
| GO:0032774 | RNA biosynthetic process | 1/11 | 240/749 | 0,986215763 | 0,999976816 | 0,855580704 | CIITA | 1 |
| GO:0031325 | positive regulation of cellular metabolic pro | 2/11 | 338/749 | 0,986877103 | 0,999976816 | 0,855580704 | CD24/CIITA | 2 |
| GO:1901564 | organonitrogen compound metabolic proce | 3/11 | 418/749 | 0,987651021 | 0,999976816 | 0,855580704 | IGHA1/CD24/SERPINA3 | 3 |
| GO:0009967 | positive regulation of signal transduction | 1/11 | 246/749 | 0,987917208 | 0,999976816 | 0,855580704 | CD24 | 1 |
| GO:0006915 | apoptotic process | 1/11 | 247/749 | 0,988181444 | 0,999976816 | 0,855580704 | CD24 | 1 |
| GO:0016070 | RNA metabolic process | 1/11 | 249/749 | 0,988694219 | 0,999976816 | 0,855580704 | CIITA | 1 |
| GO:2000112 | regulation of cellular macromolecule biosyn | 1/11 | 250/749 | 0,988942946 | 0,999976816 | 0,855580704 | CIITA | 1 |
| GO:0048519 | negative regulation of biological process | 3/11 | 423/749 | 0,989036322 | 0,999976816 | 0,855580704 | CD24/SERPINA3/CIITA | 3 |
| GO:0012501 | programmed cell death | 1/11 | 251/749 | 0,989186689 | 0,999976816 | 0,855580704 | CD24 | 1 |
| GO:0019219 | regulation of nucleobase-containing compo | 1/11 | 251/749 | 0,989186689 | 0,999976816 | 0,855580704 | CIITA | 1 |
| GO:0042127 | regulation of cell population proliferation | 1/11 | 254/749 | 0,989888904 | 0,999976816 | 0,855580704 | CD24 | 1 |
| GO:0042221 | response to chemical | 4/11 | 493/749 | 0,990042186 | 0,999976816 | 0,855580704 | IGHG4/IGHG1/CD24/CIITA | 4 |
| GO:0034654 | nucleobase-containing compound biosynthe | 1/11 | 256/749 | 0,990333737 | 0,999976816 | 0,855580704 | CIITA | 1 |
| GO:0018130 | heterocycle biosynthetic process | 1/11 | 258/749 | 0,990760708 | 0,999976816 | 0,855580704 | CIITA | 1 |
| GO:0019438 | aromatic compound biosynthetic process | 1/11 | 258/749 | 0,990760708 | 0,999976816 | 0,855580704 | CIITA | 1 |

| | | | | | | | | |
|---|---|---|---|---|---|---|---|---|
| GO:2000026 | regulation of multicellular organismal devel | 1/11 | 258/749 | 0,990760708 | 0,999976816 | 0,855580704 | CD24 | 1 |
| GO:1901362 | organic cyclic compound biosynthetic proce | 1/11 | 262/749 | 0,991563633 | 0,999976816 | 0,855580704 | CIITA | 1 |
| GO:0044238 | primary metabolic process | 4/11 | 500/749 | 0,99176341 | 0,999976816 | 0,855580704 | IGHA1/CD24/SERPINA3/CIITA | 4 |
| GO:0008219 | cell death | 1/11 | 266/749 | 0,992302616 | 0,999976816 | 0,855580704 | CD24 | 1 |
| GO:0090304 | nucleic acid metabolic process | 1/11 | 266/749 | 0,992302616 | 0,999976816 | 0,855580704 | CIITA | 1 |
| GO:0010556 | regulation of macromolecule biosynthetic p | 1/11 | 268/749 | 0,992649584 | 0,999976816 | 0,855580704 | CIITA | 1 |
| GO:0034645 | cellular macromolecule biosynthetic proces | 1/11 | 268/749 | 0,992649584 | 0,999976816 | 0,855580704 | CIITA | 1 |
| GO:0010468 | regulation of gene expression | 2/11 | 371/749 | 0,993921717 | 0,999976816 | 0,855580704 | CD24/CIITA | 2 |
| GO:0010647 | positive regulation of cell communication | 1/11 | 278/749 | 0,99418063 | 0,999976816 | 0,855580704 | CD24 | 1 |
| GO:0023056 | positive regulation of signaling | 1/11 | 278/749 | 0,99418063 | 0,999976816 | 0,855580704 | CD24 | 1 |
| GO:0044271 | cellular nitrogen compound biosynthetic pr | 1/11 | 278/749 | 0,99418063 | 0,999976816 | 0,855580704 | CIITA | 1 |
| GO:0006139 | nucleobase-containing compound metaboli | 1/11 | 279/749 | 0,994316539 | 0,999976816 | 0,855580704 | CIITA | 1 |
| GO:0031326 | regulation of cellular biosynthetic process | 1/11 | 281/749 | 0,994579737 | 0,999976816 | 0,855580704 | CIITA | 1 |
| GO:0008283 | cell population proliferation | 1/11 | 282/749 | 0,994707137 | 0,999976816 | 0,855580704 | CD24 | 1 |
| GO:0009889 | regulation of biosynthetic process | 1/11 | 284/749 | 0,994953804 | 0,999976816 | 0,855580704 | CIITA | 1 |
| GO:0046483 | heterocycle metabolic process | 1/11 | 284/749 | 0,994953804 | 0,999976816 | 0,855580704 | CIITA | 1 |
| GO:0006725 | cellular aromatic compound metabolic proc | 1/11 | 285/749 | 0,995073176 | 0,999976816 | 0,855580704 | CIITA | 1 |
| GO:0051240 | positive regulation of multicellular organism | 1/11 | 286/749 | 0,995189976 | 0,999976816 | 0,855580704 | CD24 | 1 |
| GO:0010604 | positive regulation of macromolecule metal | 2/11 | 381/749 | 0,995262324 | 0,999976816 | 0,855580704 | CD24/CIITA | 2 |
| GO:0060255 | regulation of macromolecule metabolic pro | 3/11 | 456/749 | 0,995303178 | 0,999976816 | 0,855580704 | CD24/SERPINA3/CIITA | 3 |
| GO:0009059 | macromolecule biosynthetic process | 1/11 | 288/749 | 0,995416057 | 0,999976816 | 0,855580704 | CIITA | 1 |
| GO:0010628 | positive regulation of gene expression | 1/11 | 288/749 | 0,995416057 | 0,999976816 | 0,855580704 | CIITA | 1 |
| GO:0048523 | negative regulation of cellular process | 2/11 | 386/749 | 0,995829804 | 0,999976816 | 0,855580704 | SERPINA3/CIITA | 2 |
| GO:0050793 | regulation of developmental process | 1/11 | 297/749 | 0,996318676 | 0,999976816 | 0,855580704 | CD24 | 1 |
| GO:1901360 | organic cyclic compound metabolic process | 1/11 | 297/749 | 0,996318676 | 0,999976816 | 0,855580704 | CIITA | 1 |
| GO:0010467 | gene expression | 2/11 | 393/749 | 0,996524054 | 0,999976816 | 0,855580704 | CD24/CIITA | 2 |
| GO:0034641 | cellular nitrogen compound metabolic proc | 1/11 | 305/749 | 0,996981949 | 0,999976816 | 0,855580704 | CIITA | 1 |
| GO:0035556 | intracellular signal transduction | 1/11 | 307/749 | 0,997129804 | 0,999976816 | 0,855580704 | CD24 | 1 |
| GO:1901576 | organic substance biosynthetic process | 1/11 | 319/749 | 0,997887062 | 0,999976816 | 0,855580704 | CIITA | 1 |
| GO:0044249 | cellular biosynthetic process | 1/11 | 321/749 | 0,997993905 | 0,999976816 | 0,855580704 | CIITA | 1 |
| GO:0009058 | biosynthetic process | 1/11 | 327/749 | 0,998285648 | 0,999976816 | 0,855580704 | CIITA | 1 |
| GO:0009966 | regulation of signal transduction | 1/11 | 334/749 | 0,998576947 | 0,999976816 | 0,855580704 | CD24 | 1 |
| GO:0048513 | animal organ development | 1/11 | 341/749 | 0,998822532 | 0,999976816 | 0,855580704 | CD24 | 1 |
| GO:0030154 | cell differentiation | 1/11 | 355/749 | 0,999201953 | 0,999976816 | 0,855580704 | CD24 | 1 |
| GO:0048869 | cellular developmental process | 1/11 | 359/749 | 0,999287725 | 0,999976816 | 0,855580704 | CD24 | 1 |
| GO:0010646 | regulation of cell communication | 1/11 | 368/749 | 0,999450917 | 0,999976816 | 0,855580704 | CD24 | 1 |
| GO:0023051 | regulation of signaling | 1/11 | 369/749 | 0,99946677 | 0,999976816 | 0,855580704 | CD24 | 1 |
| GO:0051239 | regulation of multicellular organismal proce | 1/11 | 376/749 | 0,999566598 | 0,999976816 | 0,855580704 | CD24 | 1 |
| GO:0048731 | system development | 1/11 | 411/749 | 0,999855652 | 0,999976816 | 0,855580704 | CD24 | 1 |
| GO:0007275 | multicellular organism development | 1/11 | 425/749 | 0,999910008 | 0,999976816 | 0,855580704 | CD24 | 1 |
| GO:0048856 | anatomical structure development | 1/11 | 444/749 | 0,999954206 | 0,999976816 | 0,855580704 | CD24 | 1 |
| GO:0032502 | developmental process | 1/11 | 462/749 | 0,999976816 | 0,999976816 | 0,855580704 | CD24 | 1 |
